# Supplementary material for: Lewis acid-catalyzed asymmetric reactions of β,γ-unsaturated 2-acyl imidazoles
Source: Nat Commun. 2020 Aug 3;11:3869. doi: 10.1038/s41467-020-17681-9 (PMC7398931; doi:10.1038/s41467-020-17681-9)
Supplement: Supplementary file 1 — Supplementary Information [file 41467_2020_17681_MOESM1_ESM.pdf]

Supplementary Information

Lewis Acid-Catalyzed Asymmetric Reactions of β,γ-Unsaturated 2-Acyl Imidazoles

Kang et al.

General Information 3
General procedures for the synthesis of substrates 3
Optimization of the reaction conditions 4
General procedure for the derivatization of the products 8
Determination of the absolute configuration of **2j**, **4r** and **11** by X-ray crystallography 10
Mechanisitic studies 11
Spectral characterization data for the substrates and products 13
Copy of ^{1}H, ^{13}C NMR spectra of substrates and products 62
Copy of CD spectra in MeOH 164
Supplementary References 172

Supplementary Methods

General Information

Unless otherwise noted, all commercially available compounds were used as provided without further purification. $Y(OTf)_3$ was purchased from Alfa Aesar. Toluene used in reactions were distilled from Na. Enantiomeric excesses (ee) were determined by HPLC or SFC analysis using the corresponding commercial chiral column as stated in the experimental procedures at 23 °C with UV detector at 254 nm. ^{1}H NMR, ^{13}C NMR and ^{19}F NMR were recorded on a Bruker AMX-400 spectrometer in $CDCl_3$. Data for ^{1}H NMR are reported as follows: chemical shift in reference to residual $CHCl_3$ at 7.26 ppm (δ ppm), multiplicity (s = singlet, br s = broad singlet, d = doublet, t = triplet, q = quartet, dd = doublet of doublets, td = triplet of doublets, m = multiplet), coupling constants (J) are in Hertz (Hz), and integration. Data for ^{13}C NMR are reported in terms of chemical shift in reference to the $CDCl_3$ solvent signal (77.16 ppm) and are reported relative to the solvent residual peaks. HRMS was recorded on a commercial apparatus (ESI Source). The chiral HPLC methods were calibrated with the corresponding racemic mixtures. Optical rotations were measured on a Rudolph Autopol V automatic polarimeter and are reported as follows: $[\alpha]^T_D$ (c g/100 mL, in solvent).

General procedures for the synthesis of substrates

General procedures for the synthesis of aryl substituted β,γ unsaturated ketones[1]

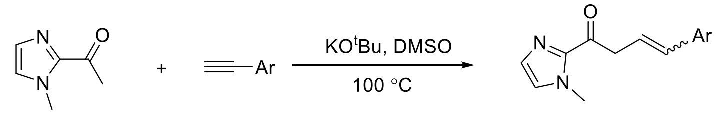

1

To a solution of ketone (10.0 mmol, 1.0 equiv) in DMSO (0.5 M) was added KO^{t}Bu (11.0 mmol, 1.1 equiv) and alkyne (1.1 equiv) in sequence at rt, the mixture was stirred at 100 °C for 0.5 – 1.0 h (determined by TLC), which was cooled down to rt, quenched by H_2O (10.0 mL) and 1 N NH_4Cl (30.0 mL). Then the mixture was extracted with Et_2O (4 × 15 mL) and dried over anhydrous Na_2SO_4. After filtration and concentration under reduced pressure to provide the crude *Z/E* mixture of aryl substituted β,γ-unsaturated ketones **1** which was then purified by flash chromatography (Pet/AcOEt = 4:1 to 3:1) to provide the aryl substituted β,γ-unsaturated ketones *E*-**1** as a pale yellow oil or yellow powder. (Usually a high ratio of *Z*-**1** was predominated when the reaction time was controlled in 5-6 minutes)

General procedures for the synthesis of alkyl substituted β,γ unsaturated ketones[2]

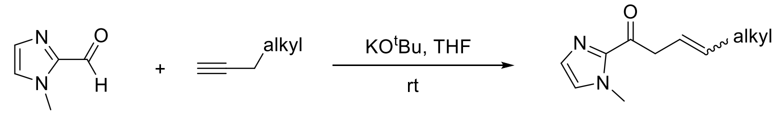

1

To a solution of 1-methyl-1*H*-imidazole-2-carbaldehyde (5.0 mmol, 1.0 equiv) in THF (0.5 M) was added KO^{t}Bu (5.0 mmol, 1.0 equiv) and alkyne (1.1 equiv) in sequence under N_2 atmosphere at rt, the mixture was stirred at rt for 0.5 h (determined by TLC), which was quenched by 1 N NH_4Cl (15.0 mL). Then the mixture was extracted with Et_2O (4 × 15.0 mL) and dried over anhydrous Na_2SO_4. After filtration and concentration under reduced pressure, the residue was purified by flash chromatography (Pet/AcOEt = 4:1 to 3:1) to provide the *Z/E* mixture of alkyl substituted β,γ-unsaturated ketones **1** as a pale yellow oil.

General procedures for the synthesis of alkyl substituted α,β-unsaturated ketone 10

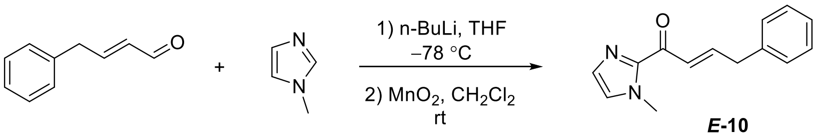

***E*-10**

To a solution of *N*-Methyl imidazole (7.7 mmol, 1.1 equiv) in THF (20.0 mL) was added n-BuLi (7.7 mmol, 1.1 equiv) under N_2 atmosphere at –78 °C, the mixture was stirred at this tempereture for 10 min and then at rt for 20 min. (*E*)-4-phenylbut-2-enal (7.0 mmol, 1.0 equiv) was added at –78 °C and stirred for 1 h (determined by TLC), which was quenched by 1 N NH_4Cl (15.0 mL). Then the mixture was extracted with Et_2O (4 × 15.0 mL) and dried over anhydrous Na_2SO_4. After filtration and concentration under reduced pressure to afford the alcohol intermediate which was dissolved in CH_2Cl_2 and treated with activated MnO_2 (80.0 mmol), the mixture was stirred at 25 °C for 0.5 h (determined by TLC). After the alcohol intermediate was oxidized completely, the mixture was filtrated through celite and the filtrate was concentrated under reduced pressure and purified by flash chromatography (Pet/AcOEt = 4:1 to 3:1) to provide the α,β-unsaturated ketones **10** as a colorless oil.

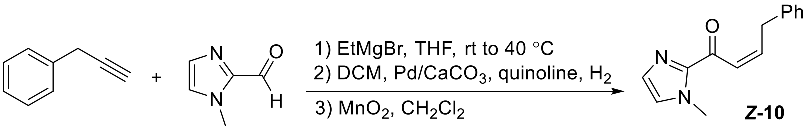

***Z*-10**

To a solution of 3-Phenyl-1-propyne (10.0 mmol, 1.0 equiv) in THF (0.5 M) was added EtMgBr (11.0 mmol, 1.1 equiv) under N_2 atmosphere at rt, the mixture was then stirred at 40 °C for 40 min. After cooling to rt, 1-methyl-1*H*-imidazole-2-carbaldehyde (10.0 mmol, 1.0 equiv) was added to

the above reaction mixture and sitrred at the same tepmeperature overnight. The mixture was quenched with 1 N NH_4Cl (30.0 mL), extracted with EA (4 × 15 mL) and dried over anhydrous Na_2SO_4. After filtration and concentration under reduced pressure to afford the alcohol intermediate which was dissolved in CH_2Cl_2 (30.0 mL) and treated with Pd/$CaCO_3$ (100.0 mg, 5% mol Pd on $CaCO_3$, poisoned with lead) and quinoline (1.0 mmol) under H_2 atmosphere (1 atm). The reaction mixture was stirred at rt for 1 h (determined by TLC) and filtrated through celite to obtain the alcohol intermediate which was treated with activated MnO_2 (100.0 mmol). After the alcohol intermediate was oxidized completely (determined by TLC, about 10.0 min), the mixture was filtrated through celite and the filtrate was concentrated under reduced pressure which was purified by flash chromatography (Pet/AcOEt = 4:1 to 3:1) to provide the α,β-unsaturated ketones ***Z*-10** as a colorless oil. (*The enone* ***Z*-10** *should be used as soon as possible due to its instability thus transforming into* ***E*-1a**).

Optimization of the reaction conditions

Optimization of the reaction conditions for α-Michael addition of β,γ-unsaturated ketone.
Supplementary Table 1: Screening of metal salts and ligands.

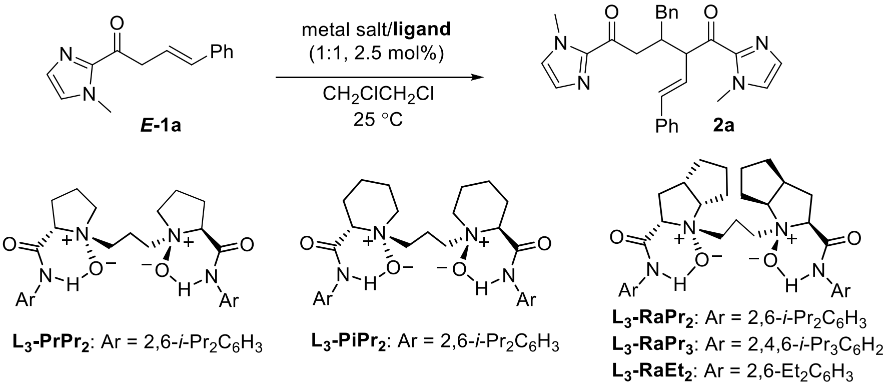

Entry[a]	metal salt	**ligand**	yield [%][b]	dr[c]	ee [%][d]
1	$Sc(OTf)_3$	**L_3-RaPr$_2$**	trace	-	-
2	$Ni(OTf)_2$	**L_3-RaPr$_2$**	trace	-	-
3	$Mg(OTf)_2$	**L_3-RaPr$_2$**	trace	-	-
4	$Zn(NTf_2)_2$	**L_3-RaPr$_2$**	trace	-	-
5	$La(OTf)_3$	**L_3-RaPr$_2$**	58	2.7:1	92/63
6	$Y(OTf)_3$	**L_3-RaPr$_2$**	60	2.2:1	96/−34
7	$Gd(OTf)_3$	**L_3-RaPr$_2$**	55	1.7:1	87/31
8	$Nd(OTf)_3$	**L_3-RaPr$_2$**	58	1.7:1	80/40
9	$Dy(OTf)_3$	**L_3-RaPr$_2$**	48	2.0:1	87/9
10	$Yb(OTf)_3$	**L_3-RaPr$_2$**	46	2.2:1	94/13
11	$Y(OTf)_3$	**L_3-PrPr$_2$**	59	2.8:1	90/10
12	$Y(OTf)_3$	**L_3-PiPr$_2$**	50	2.0:1	88/−16
13	$Y(OTf)_3$	**L_3-RaPr$_3$**	48	2.4:1	96/51
14	$Y(OTf)_3$	**L_3-RaEt$_2$**	56	2.0:1	95/53

[a] Unless otherwise noted, all reactions were performed with metal salt/**ligand** (1:1, 2.5 mol%), ***E*-1a** (0.20 mmol) in CH_2ClCH_2Cl (1.0 mL) at 25 °C under N_2 atmosphere for 24 h. [b] Isolated yield of major isomer. [c] Determined by ^1H NMR analysis. [d] Determined by HPLC analysis.

Supplementary Table 2: Screening of solvent and additive.

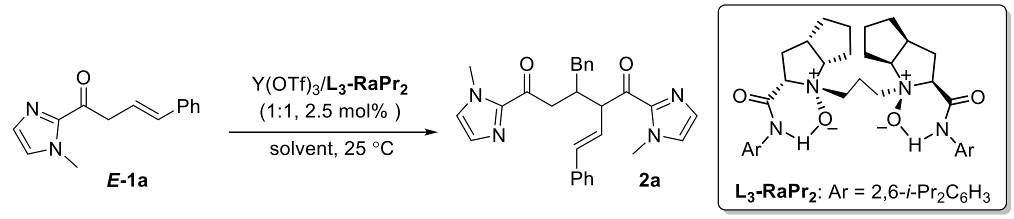

Entry[a]	solvent	additive	yield [%][b]	dr[c]	ee [%][d]
1	MeCN	-	60	1.7:1	84/67
2	EA	-	65	2.6:1	94/0
3	THF	-	54	2.0:1	95/−50
4	CH_2Cl_2	-	61	2.0:1	88/−10
5	$CHCl_3$	-	67	3.0:1	90/−22
6	Toluene	-	73	5.2:1	97/0
7[e]	*m*-Xylene	-	41	5.5:1	98/32
8[e]	*p*-Xylene	-	48	6:1	99/39
9[e]	Mesitylene	-	11	3:1	99/70

10[e]	Chlorobenzene	-	70	4.5:1	94/–60
11[f]	Toluene	NEt_3	74	10:1	98/N.D.

[a] Unless otherwise noted, all reactions were performed with $Y(OTf)_3$/**L_3-RaPr$_2$** (1:1, 2.5 mol%), ***E*-1a** (0.20 mmol) in solvent (1.0 mL) at 25 °C under N_2 atmosphere for 24 h. [b] Isolated yield of major isomer. [c] Determined by ^{1}H NMR analysis. [d] Determined by HPLC analysis. [e] The reaction time was 24 h. [f] With 10 mol% NEt_3 for 12 h.

Supplementary Table 3: Screening of other privileged ligands.

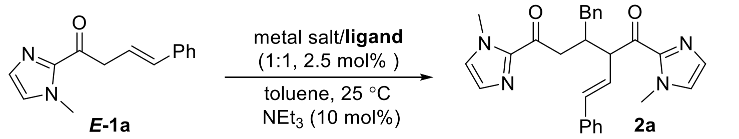

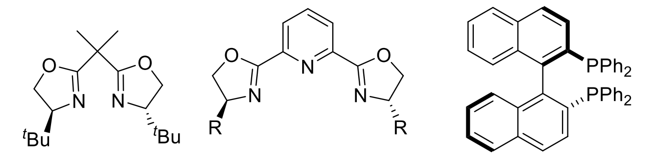

Entry[a]	metal salt	ligand	yield [%][b]	dr[c]	ee [%][d]
1	$Y(OTf)_3$	**tBu-Box**	<5	-	-
2	$Y(OTf)_3$	**Ph-PyBox**	<5	-	-
3	$Y(OTf)_3$	**BINAP**	12	5:1	60/40
4	$Cu(OTf)_2$	**tBu-Box**	trace	-	-
5	$Cu(OTf)_2$	**Ph-PyBox**	trace	-	-
6	$Cu(OTf)_2$	**iPr-PyBox**	trace	-	-
7	$Cu(MeCN)_2PF_6$	**BINAP**	32	2:1	53/40

[a] Unless otherwise noted, all reactions were performed with metal salt/**ligand** (1:1, 2.5 mol%), ***E*-1a** (0.20 mmol) in toluene (1.0 mL) at 25 °C under N_2 atmosphere for 24 h. [b] Isolated yield of major isomer. [c] Determined by ^{1}H NMR analysis. [d] Determined by HPLC analysis.

Typical procedure for the asymmetric α-Michael addition of β,γ-unsaturated ketones.

A dry reaction tube was charged with **L_3-RaPr$_2$** (3.5 mg, 2.5 mol%), $Y(OTf)_3$ (2.7 mg, 2.5 mol%), ***E*-1a** (45.2 mg, 0.20 mmol) under an N_2 atmosphere. Toluene (1.0 mL) and NEt_3 (10 mol%) were added and the mixture was stirred at 25 °C for 12 h. After the β,γ-unsaturated ketone **1a** was consumed (detected by TLC), the residue was purified by column chromatography on silica gel to afford the product **2a** (Pet/EtOAc = 1:1 as eluent) as a white foam. The enantiomeric excess (ee) was determined by high-performance liquid chromatography (HPLC) with Daicel chiralcel IE.

General procedure for the preparation of the racemic products.

The racemic products were prepared with the same procedure of the catalytic asymmetric reaction with racemic ligand (±)-**L_3-PiPr$_2$** instead of chiral ligand **L_3-RaPr$_2$.**

Typical procedure for gram-scale experiment of 2a.

An oven-dried 50 mL round bottom flask was charged with **L_3-RaPr$_2$** (0.175 mmol, 122.5 mg, 2.5 mol%), $Y(OTf)_3$ (0.175 mmol, 93.8 mg, 2.5 mol%), NEt_3 (0.7 mmol, 97.1 mg, 10 mol%) and model substrate ***E*-1a** (7.0 mmol, 1.58 g). The flask was then sealed with a stopper. The vessel was briefly evacuated and backfilled with nitrogen (repeated for three times) before 30.0 mL toluene was added. Then the reaction mixture stirred at 25 °C for 12 h. After removing most of toluene under vaccum, it was directly purified by flash chromatography on silica gel (eluent: Pet : EtOAc = 1:1) to afford the desired product **2a** (1.115 g, 70% yield, 98% ee).

Optimization of the reaction conditions for direct Mannich reaction.
Supplementary Table 4: Optimization of the reaction conditions for direct Mannich reaction of β,γ-unsaturated ketones to isatin-derived ketimines.

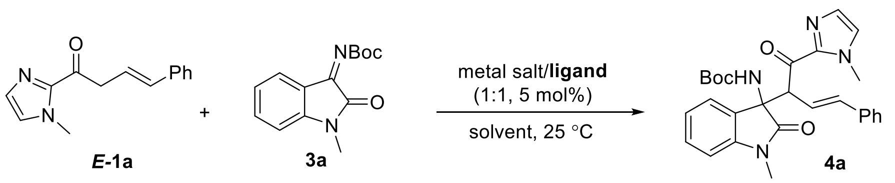

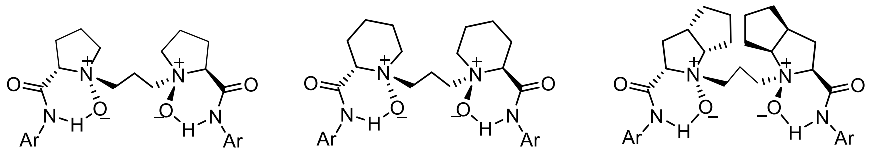

L_3-PrPr$_2$: Ar = 2,6-*i*-$Pr_2C_6H_3$

L_3-PiPr$_2$: Ar = 2,6-*i*-$Pr_2C_6H_3$
L_3-PiPr$_3$: Ar = 2,4,6-*i*-$Pr_3C_6H_2$
L_3-Pi^{t}Bu: Ar = ^{t}Bu
L_3-PiCy: Ar = Cy

L_3-RaPr$_2$: Ar = 2,6-*i*-$Pr_2C_6H_3$

Entry[a]	metal salt	**ligand**	solvent	yield [%]	dr[b]	ee [%][b]
1	$Y(OTf)_3$	**L_3-RaPr$_2$**	CH_2ClCH_2Cl	97	1:1	31/9
2	$Sc(OTf)_3$	**L_3-RaPr$_2$**	CH_2ClCH_2Cl	88	1:3	0/0
3	$Ni(BF_4)_2$•$6H_2O$	**L_3-RaPr$_2$**	CH_2ClCH_2Cl	98	1:2	15/5
4	$Zn(NTf_2)_2$	**L_3-RaPr$_2$**	CH_2ClCH_2Cl	98	1:1	0/0
5	$Mg(OTf)_2$	**L_3-RaPr$_2$**	CH_2ClCH_2Cl	95	4:1	27/0
6	$La(OTf)_3$	**L_3-RaPr$_2$**	CH_2ClCH_2Cl	98	4.3:1	−32/0
7	$Yb(OTf)_3$	**L_3-RaPr$_2$**	CH_2ClCH_2Cl	98	1:1	10/13
8	$Eu(OTf)_3$	**L_3-RaPr$_2$**	CH_2ClCH_2Cl	99	1.4: 1	−18/−55
9	$Gd(OTf)_3$	**L_3-RaPr$_2$**	CH_2ClCH_2Cl	87	1.4:1	−14/−32
10	$Er(OTf)_3$	**L_3-RaPr$_2$**	CH_2ClCH_2Cl	99	1:1	19/-30
11	$La(OTf)_3$	**L_3-PiPr$_2$**	CH_2ClCH_2Cl	88	19:1	−56/-
12	$La(OTf)_3$	**L_3-PrPr$_2$**	CH_2ClCH_2Cl	93	13:1	−40/-
13	$La(OTf)_3$	**L_3-Pi^{t}Bu**	CH_2ClCH_2Cl	98	8:1	−72/42
14	$La(OTf)_3$	**L_3-Pi^{t}Cy**	CH_2ClCH_2Cl	99	2.5:1	17/0
15	$La(OTf)_3$	**L_3-Pi^{t}Bu**	CH_2Cl_2	96	7.3:1	−72/56
16	$La(OTf)_3$	**L_3-Pi^{t}Bu**	$CHCl_3$	68	7.3:1	−53/8
17	$La(OTf)_3$	**L_3-Pi^{t}Bu**	$CH_2ClCHCl_2$	99	15.6:1	−75/-
18	$La(OTf)_3$	**L_3-Pi^{t}Bu**	THF	79	1:1	0/0
19[c]	$La(OTf)_3$	**L_3-Pi^{t}Bu**	$CH_2ClCHCl_2$	70	>19:1	−77/-
20[c,d]	$La(OTf)_3$	**L_3-Pi^{t}Bu**	$CH_2ClCHCl_2$	95	>19:1	−91/-

[a] Unless otherwise noted, all reactions were performed with metal salt/**ligand** (1:1, 5 mol%), ***E*-1a** (0.12 mmol) and **4a** (0.10 mmol) in solvent (1.0 mL) at 25 °C for 2 h.
[b] Determined by HPLC analysis. [c] At 0 °C for 12 h. [d] 3 Å MS (30 mg) was added.

Typical procedure for direct Mannich reaction of β,γ-unsaturated ketones to imines.

With isatin-derived ketimines: A dry reaction tube was charged with **L_3-Pi^{t}Bu** (2.2 mg, 5 mol%), $La(OTf)_3$ (2.9 mg, 5 mol%), 3 Å M.S. (30 mg) and ***E*-1a** (27.1 mg, 0.12 mmol) in $CH_2ClCHCl_2$ (1.0 mL). The mixture was stirred at 30 °C for 30 min, then **3a** (0.10 mmol, 26.0 mg) was added at 0 °C. After **3a** was consumed (detected by TLC), the residue was purified by column chromatography on silica gel to afford the product **4a** (Pet/EtOAc = 1:1 as eluent) as a white foam. The enantiomeric excess (ee) was determined by **UPC^2** (Chiral **IC3**).
With pyrazolinone-derived ketimines: A dry reaction tube was charged with **L_3-RaPr$_2$** (3.5 mg, 5 mol%), $La(OTf)_3$ (2.9 mg, 5 mol%), ***E*-1a** (24.9 mg, 0.11 mmol) and pyrazolinone-derived ketimine (34.9 mg, 0.10 mmol) in $CHCl_3$ (1.0 mL). The mixture was stirred at 0 °C for 8 h. After ketimine was consumed (detected by TLC), the residue was purified by column chromatography on silica gel to afford the product **4i** (Pet/EtOAc = 2:1 as eluent) as a white foam. The enantiomeric excess (ee) was determined by **UPC^2** (Chiral **IB3**).
With aldimines: A dry reaction tube was charged with **L_3-RaPr$_2$** (7.0 mg, 10 mol%), $La(OTf)_3$ (5.9 mg, 10 mol%), ***E*-1a** (24.9 mg, 0.10 mmol), 4 Å M.S. (20 mg) and benzaldehyde-dervived aldimine (30.8 mg, 0.15 mmol) in $CH_2ClCHCl_2$ (1.0 mL). The mixture was stirred at 35 °C for 10 h. After ***E*-1a** was consumed (detected by TLC), the residue was purified by column chromatography on silica gel to afford the product **4q** (Pet/EtOAc = 2:1 as eluent) as a colorless oil. The enantiomeric excess (ee) was determined by chiral HPLC (**IA**).

Optimization of the reaction conditions for tandem isomerization/sulfur Michael addition.
Supplementary Table 5: Optimization of the reaction conditions.

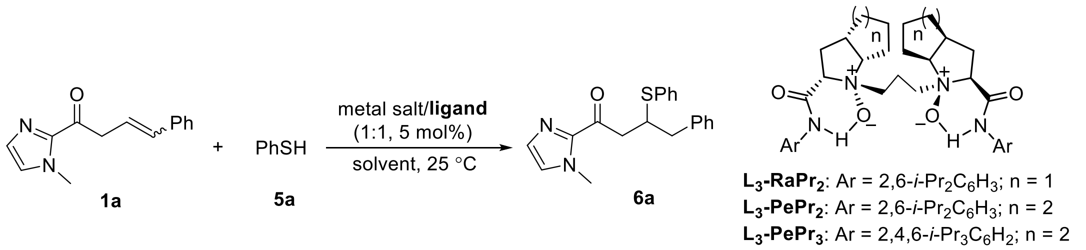

Entry[a]	metal salt	ligand	*E*-1a or *Z*-1a	solvent	yield [%]	ee [%][b]
1	$Y(OTf)_3$	**L_3-RaPr$_2$**	***E*-1a**	CH_2ClCH_2Cl	trace	-
2	$La(OTf)_3$	**L_3-RaPr$_2$**	***E*-1a**	CH_2ClCH_2Cl	trace	-
3	$Dy(OTf)_3$	**L_3-RaPr$_2$**	***E*-1a**	CH_2ClCH_2Cl	trace	-
4	$Y(OTf)_3$	**L_3-RaPr$_2$**	***Z*-1a**	CH_2ClCH_2Cl	41	72
5	$La(OTf)_3$	**L_3-RaPr$_2$**	***Z*-1a**	CH_2ClCH_2Cl	45	73
6	$Dy(OTf)_3$	**L_3-RaPr$_2$**	***Z*-1a**	CH_2ClCH_2Cl	50	72
7	$Yb(OTf)_3$	**L_3-RaPr$_2$**	***Z*-1a**	CH_2ClCH_2Cl	32	71
8	$Dy(OTf)_3$	**L_3-PePr$_2$**	***Z*-1a**	CH_2ClCH_2Cl	54	73
9	$Dy(OTf)_3$	**L_3-PePr$_3$**	***Z*-1a**	CH_2ClCH_2Cl	65	84
10[c]	$Dy(OTf)_3$	**L_3-PePr$_3$**	***Z*-1a**	$CH_2ClCHCl_2$	73	90
11[c,d]	$Dy(OTf)_3$	**L_3-PePr$_3$**	***Z*-1a**	$CH_2ClCHCl_2$	89	90

[a] Unless otherwise noted, all reactions were performed with metal salt/**ligand** (1:1, 5 mol%), **1a** (0.15 mmol) and PhSH (0.10 mmol) in solvent (1.0 mL) at 25 °C for 12 h. [b] Determined by HPLC analysis. [c] $CH_2ClCHCl_2$ was used as solvent. [d] 2.5 equiv ***Z*-1a** was used for 17 h.

Typical procedure for tandem isomerization/sulfur Michael addition.

A dry reaction tube was charged with **L_3-PePr$_3$** (4.2 mg, 5 mol%), $Dy(OTf)_3$ (3.0 mg, 5 mol%), ***Z*-1a** (56.5 mg, 0.25 mmol) in $CH_2ClCHCl_2$ (1.0 mL). PhSH (0.10 mmol) was added and the mixture was stirred at 25 °C for 12 h. After PhSH was consumed (detected by TLC), the residue was purified by column chromatography on silica gel to afford the product **6a** (Pet/EtOAc = 3:1 as eluent) as a pale yellow oil. The enantiomeric excess (ee) was determined by **UPC^2** (Chiral **OJ-3**).

Optimization of the reaction conditions for *syn*-selectivity of 2a.
Supplementary Table 6: Optimization of the reaction conditions for *syn*-2a.

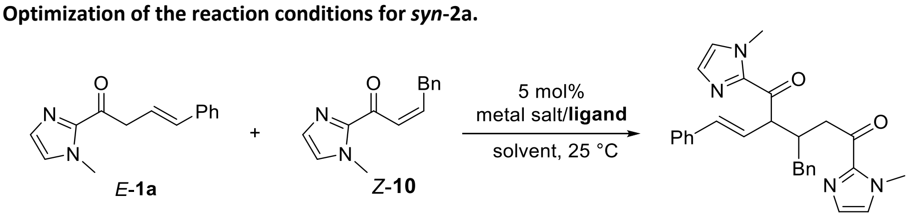

Entry[a]	metal salt	ligand	solvent	time (h)	yield (%)[b]	dr[c]	ee (%)[d]
1	$Y(OTf)_3$	**L_3-RaPr$_2$**	toluene	1	53	6.2:1	99
2[e]	$Y(OTf)_3$	**L_3-RaPr$_2$**	toluene	2	48	2.6:1	97
3[f]	$Y(OTf)_3$	**L_3-RaPr$_2$**	toluene	2	57	3.4:1	98
4	$Sc(OTf)_3$	**L_3-RaPr$_2$**	toluene	2	<5	-	-
5	$La(OTf)_3$	**L_3-RaPr$_2$**	toluene	2	56	8:1	99
6	$Dy(OTf)_3$	**L_3-RaPr$_2$**	toluene	2	55	4.7:1	98
7	$Yb(OTf)_3$	**L_3-RaPr$_2$**	toluene	2	40	3:1	97
8	$La(OTf)_3$	**L_3-RaPr$_2$**	*m*-xylene	2	50	8.1:1	>99
9	$Y(OTf)_3$	**L_3-RaPr$_2$**	*m*-xylene	2	67	8:1	>99
10	$Y(OTf)_3$	**L_3-RaPr$_2$**	*p*-xylene	2	38	3.5:1	>99
11	$Y(OTf)_3$	**L_3-RaPr$_2$**	Mesitylene	2	trace	-	-
12	$Y(OTf)_3$	**L_3-RaPr$_2$**	Chlorobenzene	2	68	2.4:1	98
13	$Y(OTf)_3$	**L_3-RaPr$_2$**	THF	2	63	4.9:1	98
14	$Y(OTf)_3$	***ent*-L_3-RaPr$_2$**	*m*-xylene	2	69	9:1	>99

[a] Unless otherwise noted, all reactions were performed with metal salt/**ligand** (1:1, 5 mol%), *E*-**1a** (0.10 mmol), *Z*-**10** (0.10 mmol) in solvent (1.0 mL) at 25 °C under N_2 atmosphere. [b] The yield was based on isolated *syn*-isomer. [c] The dr value was determined by ^{1}H NMR. [d] The ee value was determined by HPLC analysis on chiral stationary phases. [e] 10 mol% of catalyst was used. [f] Toluene (0.2M) was used.

Michael reaction

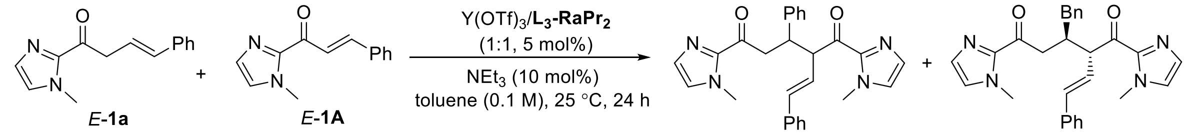

2A **2a**

5:1 mixture of **2A**:**2a** by ^{1}H NMR
can't be isolated on column chromatography

other Michael acceptors:

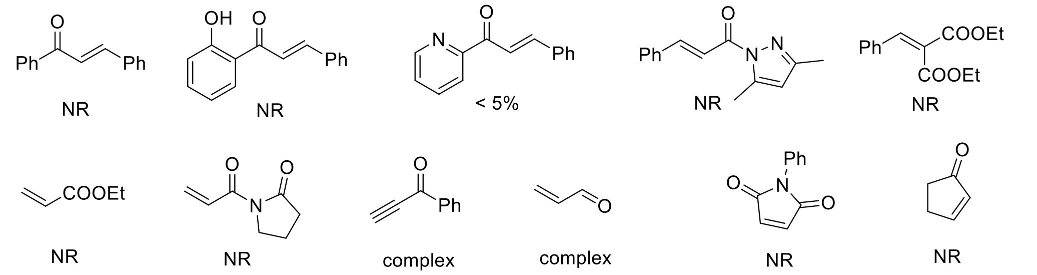

Mannich reaction

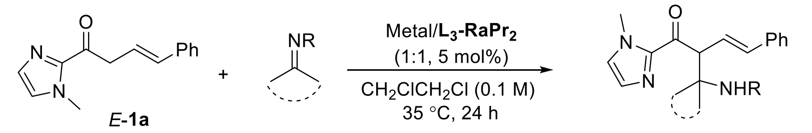

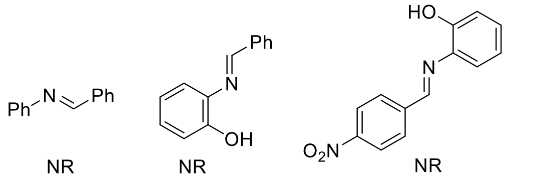

Supplementary Figure 1. Unsuccessful substrates.

General procedure for the derivatization of the products

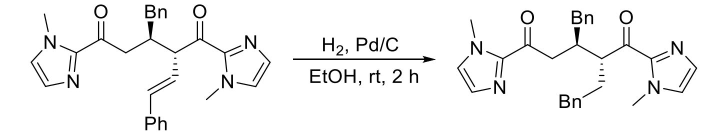

2a, >19:1 dr, 98% ee **7**, 98%, 98% ee

To a stirred solution of **2a** (1.0 mmol) in EtOH (20 mL) at rt was added Pd/C (10% mol Pd on carbon) (30 mg), the reaction mixture was stirred at rt under H_2 atmosphere for 2 h. Then, the mixture was filtered and the filtrate was concentrated under reduced pressure to give the crude product and was subsequently purified by flash column chromatography on silica gel (Pet/AcOEt = 1:1) to afford **7** in 98% yield with 98% ee.

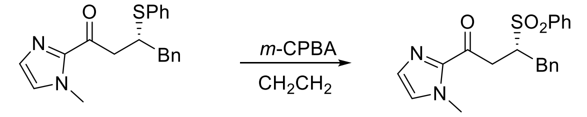

6a, 90% ee **8**, 85%, 90% ee

To a stirred solution of **6a** (0.3 mmol) in CH_2Cl_2 (10 mL) at 0 °C was added *m*-CPBA (3.0 euqiv), and the reaction mixture was stirred at rt for 1 h. Then, the reaction mixture was quenched with saturated $NaHCO_3$ and extracted with CH_2Cl_2 (3 × 10 mL). The combined organic layer was washed with saturated NaCl and dried over $NaSO_4$, filtered and concentrated under reduced pressure to give the crude product and was subsequently purified by flash column chromatography on silica gel (Pet/AcOEt = 1:1) to afford **8** in 85% yield with 90% ee.

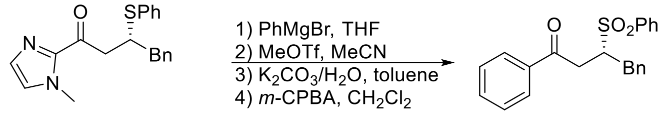

6a, 90% ee **9**, 50%, 85% ee

To a stirred solution of **6a** (0.5 mmol) in THF (5.0 mL) at 0 °C was added PhMgBr (2.0 euqiv) under N_2 atmosphere, and the reaction mixture was stirred at 0 °C for 5 h. Then, the reaction mixture was quenched with saturated H_2O (1.0 mL). The resulting solution was diluted with 25.0 mL diethyl ether and 30.0 mL of 1N HCl. The bilayer system as stirred vigorously for 10 min. The organic layer was removed and the resulting queous layer was brought to a pH > 9 with solid Na_2CO_3. The aqueous layer was ubsequently extracted with 3x15 mL of Et_2O and extracted with Et_2O (3 × 10 mL). The combined organic layer was washed with saturated NaCl and dried over $NaSO_4$, filtered and concentrated under reduced pressure to give the crude product and then it was subsequently dissolved in 3.0 mL MeCN and MeOTf (1.0 mmol) was added at rt. After stirring ar rt for 1 h (determined by TLC), the excess MeOTf and MeCN were removed under reduced pressure before 3.0 mL of toluene and 1.0 mL of a 10% wt aq. K_2CO_3 were added to the vial. The bilayer system was stirred vigorously for 1.5 h at 60 °C. The resulting solution was cooled to rt and diluted with 30.0 mL Et_2O. The organic layer was washed with 30.0 mL sat. $NaHCO_3$ and 30.0 ml brine. The organic layer was dried with Na_2SO_4. The drying agent was removed by filtration and reaction concentrated *in vacuo* and purified by flash column chromatography on silica gel (Pet/AcOEt = 15:1) to afford **9** in 50% yield with 85% ee.

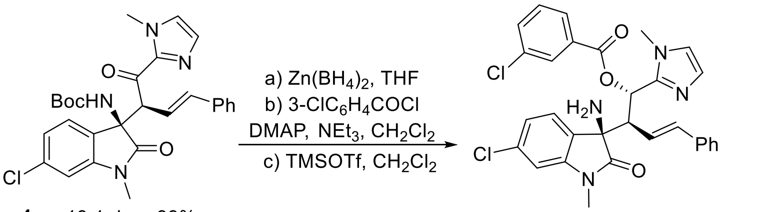

4e, >19:1 dr, >99% ee (after recrystallization)

11, 70% yield for major 5:1 dr, >99% ee

To a stirred solution of **4e** (0.2 mmol, >19:1 dr, >99% ee) in THF (5 mL) at 0 °C was added $Zn(BH_4)_2$ (5.0 euqiv, 0.5 mol/L THF solution), and the reaction mixture was stirred at 0 °C for 5 h. Then the reaction mixture was quenched with saturated NH_4Cl (10 mL) and extracted with EA (3 × 10 mL). The combined organic layer was washed with saturated NaCl and dried over $NaSO_4$, filtered and concentrated under reduced pressure to give the crude product which was dissolved in 4 mL CH_2Cl_2 and 3-ClC_6H_4COCl (0.30 mmol), NEt_3 (0.30 mmol), DMAP (0.04 mmol) were added in this sequence at 0 °C. After the alcohol material was consumed (determined by TLC), the reaction mixture was quenched with saturated $NaHCO_3$ and extracted with CH_2Cl_2 (3 × 10 mL). The combined organic layer was washed with saturated NaCl and dried over $NaSO_4$, filtered and concentrated under reduced pressure to give the crude product which was dissolved in 3 mL CH_2Cl_2 and TMSOTf (1.2 mmol) was added at rt. After stirring at rt for 10 minutes (determined by TLC), the reaction mixture was quenched with saturated $NaHCO_3$ and extracted with CH_2Cl_2 (3 × 10 mL). The combined organic layer was washed with saturated NaCl and dried over $NaSO_4$, filtered and concentrated under reduced pressure to give the crude product which was subsequently purified by flash column chromatography on silica gel (Pet/AcOEt = 1:1 to 0:1) to afford **11** in 70% yield with >99% ee for major isomer.

Determination of the absolute configuration of 2j, 4r and 11 by X-ray crystallography

Good quality crystal of **2j**, **4r** and **11** were obtained by vaporization of a toluene/petroleum ether solution of compound **2j** and a dichloromethane/ethyl acetate/petroleum ether solution of compound **4r** and **11**. CCDC 1972987, 2001513 and 1972937 contains the supplementary crystallographic data for this paper. These data can be obtained free of charge from the Cambridge Crystallographic Data Centre via www.ccdc.cam.ac.uk/data_request/cif.

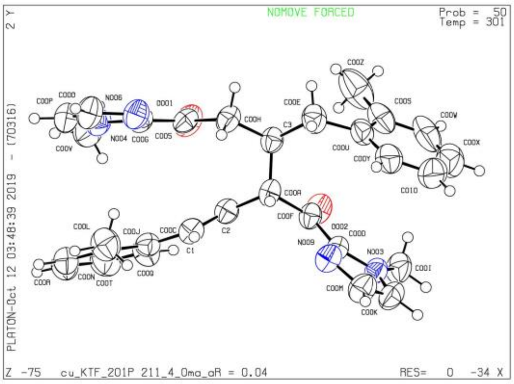

2j CCDC 1972987

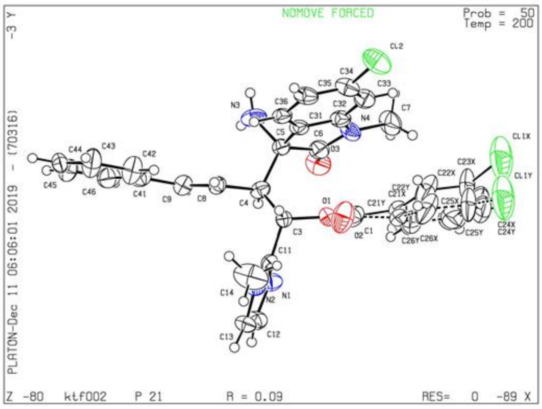

11 CCDC 1972937

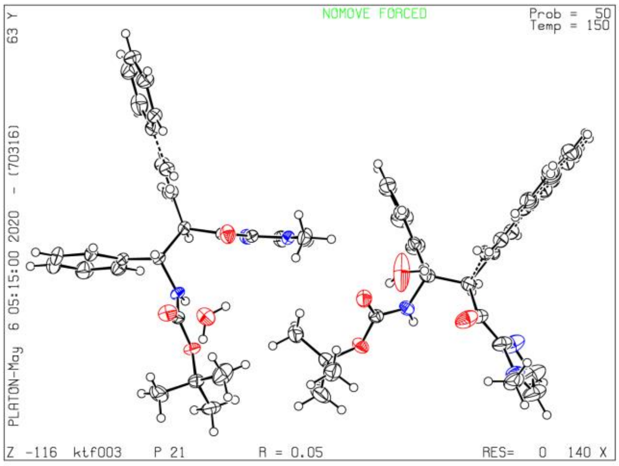

4r CCDC 2001513

Supplementary Figure 2. Determination of the absolute configuration of **2j**, **4r** and **11**

Mechanistic studies

Control experiments

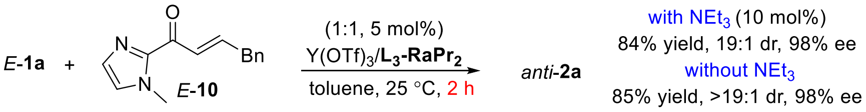

Supplementary Note 1. Firstly, α,β-unsaturated ketone *E*-**10** was synthezied to react with *E*-**1a**, and both afforded *anti*-**2a** in good yields (84-85%), excellent diastereoselectivities (19:1 to >19:1) and ee values (98%) within 2 h no matter with or without addition of NEt_3.

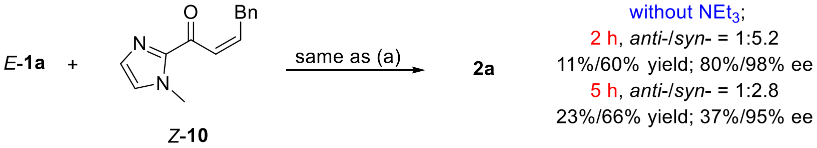

Supplementary Note 2. When α,β-unsaturated ketone *Z*-**10** was reacted with *E*-**1a**, 1： 5.2 *anti-/syn-* was obtained in the absence of NEt_3 for 2 h which was then decreased to 1:2.8 *anti-/syn-* when the reaction time was prolonged to 5 h due to the instability of *Z*-**10**.

Supplementary Note 3. The above mentioned control experiments (a) and (b) could confirm our hypothesis on isomerization of β,γ-unsaturated ketones **1** to α,β-conjugated ketones **10**. (Note: Trace amount of **10** was observed if the reaction of *E*-**1a** was performed under the standard conditions without addition of $Y(OTf)_3$/**L_3-RaPr$_2$** complex).

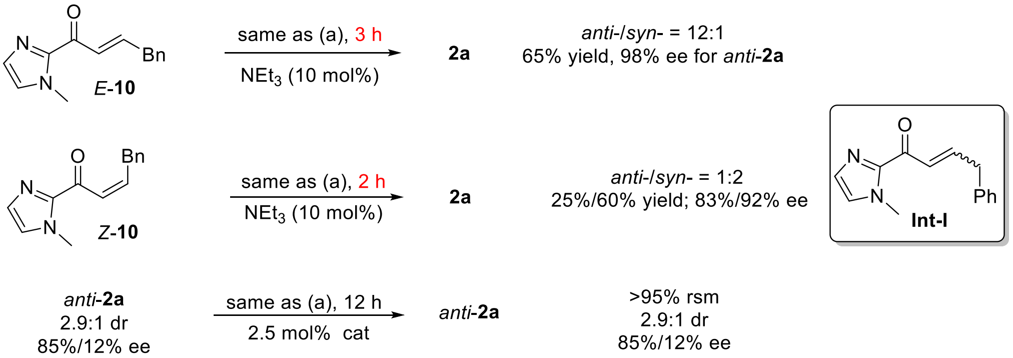

Supplementary Note 4. In addition, single *E*-**10** could also be transformed into **2a** with 12:1 *anti-/syn-* within 3 h, however, only 1:2 *anti-/syn-* was obtained when Z-**10** was utilized within 2 h (c and d). Also, the low *anti-/syn-* ratio could be attributed to the instability of Z-**10** and transforming into *E*-**1a** very easily. These experiments suggested β,γ-unsaturated ketones and α,β-unsaturated ketones can be interchangeable in this reaction, and the transformation of *E*-**1a** to **Int-I** was likely to be the rate determining step, and the addition of NEt_3 can facilitate this process.

Supplementary Note 5. On the other hand, we wondered why the addition of NEt_3 could improve the diastereoselectivity (Table 1, entry 6, in the manuscript). Treating the product *anti*-**2a** (2.9:1 dr, 85%/12% ee) under the standard conditions for 12 h, no loss of enantioselectivity and diastereoselectivity of recovered **2a** was observed (e), which ruled out the possibility that the diastereoselectivity was increased through epimerization of **2a** in the presence of NEt_3.

Supplementary Note 6. Overall the above control experiments, we could conclude that the additive of NEt_3 play a key role to enhance the *E*/*Z* ratio of **Int-I** of the isomerization process. In other words, the diastereoselectivity was very likely determined by the *E*/*Z* ratio of **Int-I**.

The operando IR experiments

Supplementary Note 7. To gain further insight into the mechanism, operando IR experiments were performed to interpret the process of the reaction. As depicted in Figure S1, **1a**, **2a** and **int.** were monitored by the operando IR spectrometer. The peaks at 968 cm^{-1} is related to **1a** gradually decreased in intensity and the peaks at 854 cm^{-1} is related to the intermediate **int.** gradually increased and then decreased in intensity. It was shown clearly that the amount of product **2a** (peak at 881 cm^{-1}) increased with the decrease of starting material. In Figure S2, it was found that the reaction rate was slow in the initial 2 h, and bacame faster as the formation of intermediate **int.**

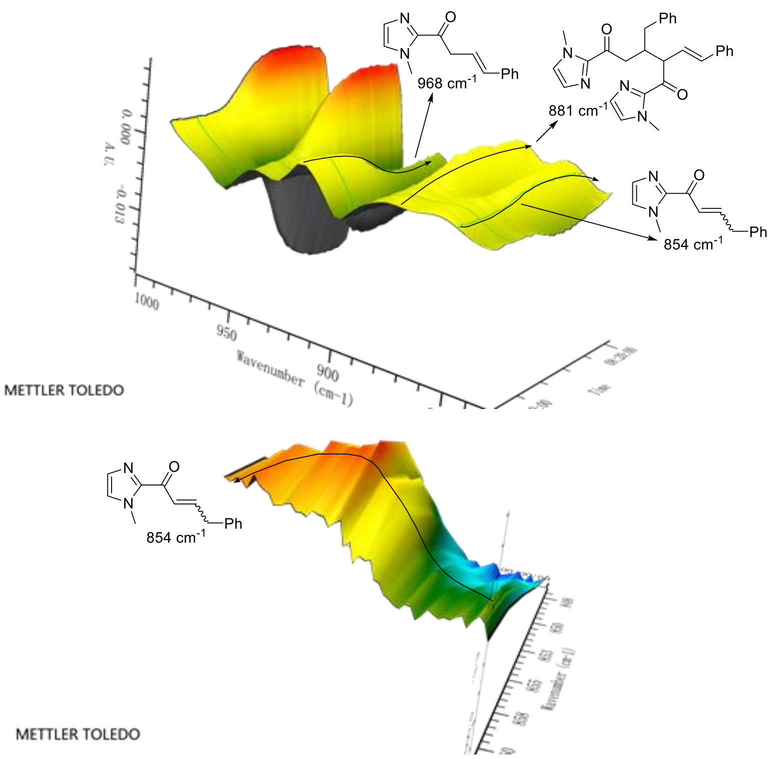

Supplementary Figure 3. The operando IR experimen

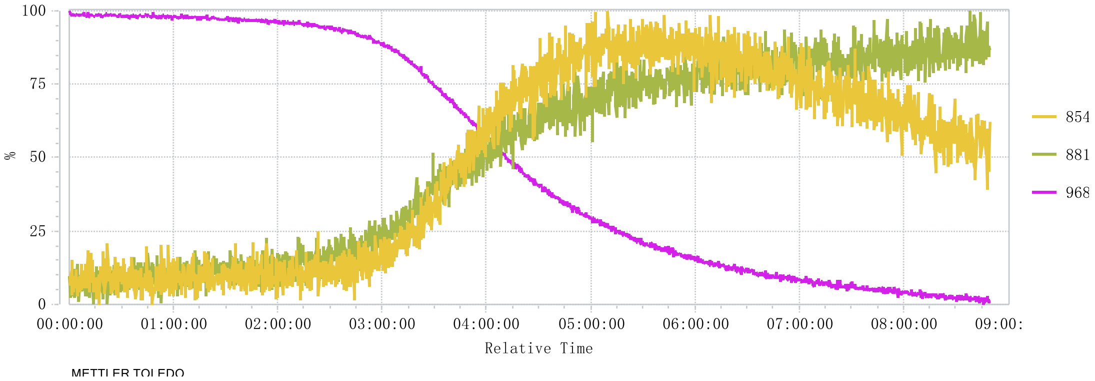

Supplementary Figure 4. The trend of each component (X axes: reaction time; Y axes: absorbance unit). **1a**: peak at 968 cm^{-1}; **2a**: peak at 881 cm^{-1}; intermediate: peak at 854 cm^{-1}.

Spectral characterization data for the substrates and products

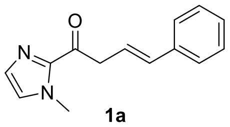

1a

(*E*)-1-(1-Methyl-1H-imidazol-2-yl)-4-phenylbut-3-en-1-one

^{1}H NMR (400 MHz, $CDCl_3$) δ 7.42 – 7.35 (m, 2H), 7.31 – 7.25 (m, 2H), 7.23 – 7.18 (m, 1H), 7.17 (d, *J* = 0.8 Hz, 1H), 7.04 (s, 1H), 6.58 (d, *J* = 16.0 Hz, 1H), 6.47 (dt, *J* = 16.0, 6.8 Hz, 1H), 4.04 (dd, *J* = 6.8, 0.8 Hz, 2H), 4.00 (s, 3H).

^{13}C NMR (100 MHz, $CDCl_3$) δ 190.5, 142.8, 137.3, 133.8, 129.4, 128.6, 127.5, 127.3, 126.4, 122.6, 43.3, 36.3.

HRMS (ESI-TOF) calcd for $C_{14}H_{15}N_2O^+$ ($[M+H^+]$) = 227.1179, Found 227.1178.

IR (film): 1673, 1402, 1288, 1154, 983, 914 cm^{-1}.

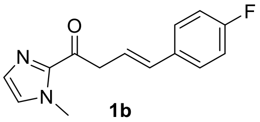

1b

(*E*)-4-(4-Fluorophenyl)-1-(1-methyl-1H-imidazol-2-yl)but-3-en-1-one

^{1}H NMR (400 MHz, $CDCl_3$) δ 7.36 – 7.29 (m, 2H), 7.15 (d, *J* = 0.8 Hz, 1H), 7.03 (s, 1H), 6.99 – 6.92 (m, 2H), 6.52 (d, *J* = 16.0 Hz, 1H), 6.37 (dt, *J* = 16.0, 6.8 Hz, 1H), 4.01 (dd, *J* = 6.8, 1.2 Hz, 2H), 3.98 (s, 3H).

^{13}C NMR (100 MHz, $CDCl_3$) δ 190.4, 162.3 (d, *J* = 245.0 Hz), 142.7, 133.4 (d, *J* = 4.0 Hz), 132.5, 129.4, 127.8 (d, *J* = 8.0 Hz), 127.3, 122.4 (d, *J* = 3.0 Hz), 115.4 (d, *J* = 21.0 Hz), 43.1, 36.2.

^{19}F NMR (376 MHz, CDCl3) δ −115.39 (s, 1F).

HRMS (ESI-TOF) calcd for $C_{14}H_{14}FN_2O^+$ ($[M+H^+]$) = 245.1085, Found 245.1090.

IR (film): 1675, 1507, 1404, 1224, 1156, 984, 915 cm^{-1}.

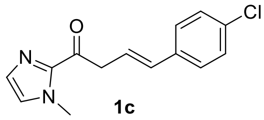

1c

(*E*)-4-(4-Chlorophenyl)-1-(1-methyl-1H-imidazol-2-yl)but-3-en-1-one

^{1}H NMR (400 MHz, $CDCl_3$) δ 7.31 – 7.27 (m, 2H), 7.25 – 7.20 (m, 2H), 7.15 (d, *J* = 0.4 Hz, 1H), 7.03 (s, 1H), 6.50 (d, *J* = 16.0 Hz, 1H), 6.43 (dt, *J* = 16.0, 6.4 Hz, 1H), 4.01 (d, *J* = 6.4 Hz, 2H), 3.98 (s, 3H).

^{13}C NMR (100 MHz, $CDCl_3$) δ 190.3, 142.7, 135.8, 133.1, 132.6, 129.4, 128.7, 127.6, 127.4, 123.4, 43.2, 36.3.

HRMS (ESI-TOF) calcd for $C_{14}H_{14}{}^{34.9689}ClN_2O^+$ ($[M+H^+]$) = 261.0789, Found 261.0794.

HRMS (ESI-TOF) calcd for $C_{14}H_{14}{}^{36.9659}ClN_2O^+$ ($[M+H^+]$) = 263.0760, Found 263.0762.

IR (film): 1674, 1490, 1403, 1285, 1090, 984, 914 cm^{-1}.

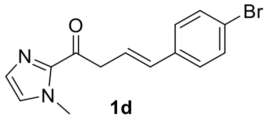

1d

(*E*)-4-(4-Bromophenyl)-1-(1-methyl-1H-imidazol-2-yl)but-3-en-1-one

^{1}H NMR (400 MHz, $CDCl_3$) δ 7.39 (d, *J* = 8.4 Hz, 2H), 7.23 (d, *J* = 9.2 Hz, 2H), 7.16 (s, 1H), 7.04 (s, 1H), 6.55 – 6.39 (m, 2H), 4.02 (d, *J* = 5.6 Hz, 2H), 3.98 (s, 3H).

^{13}C NMR (100 MHz, $CDCl_3$) δ 190.2, 142.7, 136.2, 132.6, 131.6, 129.4, 127.9, 127.4, 123.5, 121.2, 43.1, 36.3.

HRMS (ESI-TOF) calcd for $C_{14}H_{14}{}^{78.9183}BrN_2O^+$ ($[M+H^+]$) = 305.0284, Found 305.0294.

HRMS (ESI-TOF) calcd for $C_{14}H_{14}{}^{80.9163}BrN_2O^+$ ($[M+H^+]$) = 307.0264, Found 307.0272.

IR (film): 1673, 1485, 1403, 1286, 1154, 983, 914 cm^{-1}.

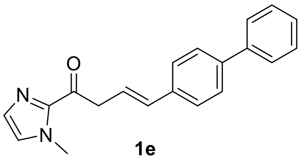

1e

(*E*)-4-([1,1'-Biphenyl]-4-yl)-1-(1-methyl-1H-imidazol-2-yl)but-3-en-1-one

^{1}H NMR (400 MHz, $CDCl_3$) δ 7.62 – 7.52 (m, 4H), 7.49 – 7.40 (m, 4H), 7.33 (t, *J* = 7.2 Hz, 1H), 7.19 (s, 1H), 7.06 (s, 1H), 6.62 (d, *J* = 16.0 Hz, 1H), 6.52 (dt, *J* = 16.0, 6.4 Hz, 1H), 4.10 – 4.05 (m, 2H), 4.02 (s, 3H).

^{13}C NMR (100 MHz, $CDCl_3$) δ 190.5, 142.8, 140.9, 140.2, 136.9, 136.3, 133.4, 129.4, 128.9, 127.4, 127.3, 127.1, 126.9, 122.8, 115.4, 43.4, 36.3.

HRMS (ESI-TOF) calcd for $C_{20}H_{19}N_2O^+$ ($[M+H^+]$) = 303.1492, Found 303.1497.

IR (film): 1675, 1467, 1269, 1154, 983, 915 cm^{-1}.

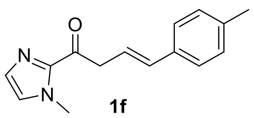

1f

(*E*)-1-(1-Methyl-1H-imidazol-2-yl)-4-(p-tolyl)but-3-en-1-one
^{1}H NMR (400 MHz, $CDCl_3$) δ 7.28 (d, *J* = 8.0 Hz, 2H), 7.16 (d, *J* = 0.8 Hz, 1H), 7.09 (d, *J* = 8.0 Hz, 2H), 7.03 (s, 1H), 6.54 (d, *J* = 16.0 Hz, 1H), 6.41 (dt, *J* = 15.6, 7.2 Hz, 1H), 4.02 (dd, *J* = 7.2, 1.2 Hz, 2H), 3.99 (s, 3H), 2.31 (s, 3H).
^{13}C NMR (100 MHz, $CDCl_3$) δ 190.6, 142.8, 137.2, 134.5, 133.7, 129.3, 129.3, 127.3, 126.3, 121.5, 43.3, 36.3, 21.3.
HRMS (ESI-TOF) calcd for $C_{15}H_{17}N_2O^+$ ($[M]+H^+$) = 241.1335, Found 241.1341.
IR (film): 1673, 1617, 1539, 1405, 1286, 984, 915 cm^{-1}.

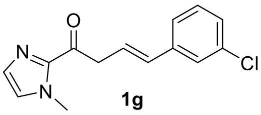

1g

(*E*)-4-(3-Chlorophenyl)-1-(1-methyl-1H-imidazol-2-yl)but-3-en-1-one
^{1}H NMR (400 MHz, $CDCl_3$) δ 7.37 (s, 1H), 7.28 – 7.20 (m, 2H), 7.20 – 7.15 (m, 2H), 7.05 (s, 1H), 6.56 – 6.43 (m, 2H), 4.06 – 4.03 (m, 2H), 4.00 (s, 3H).
^{13}C NMR (100 MHz, $CDCl_3$) δ 190.1, 142.7, 139.1, 134.5, 132.4, 129.8, 129.5, 127.4, 126.3, 124.6, 124.3, 43.1, 36.3.
HRMS (ESI-TOF) calcd for $C_{14}H_{14}{}^{34.9689}ClN_2O^+$ ($[M]+H^+$) = 261.0789, Found 261.0789.
HRMS (ESI-TOF) calcd for $C_{14}H_{14}{}^{36.9659}ClN_2O^+$ ($[M]+H^+$) = 263.0760, Found 263.0757.
IR (film): 1674, 1565, 1406, 1283, 1081, 984, 915 cm^{-1}.

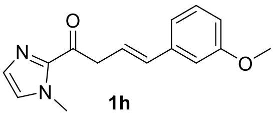

1h

(*E*)-4-(3-Methoxyphenyl)-1-(1-methyl-1H-imidazol-2-yl)but-3-en-1-one
^{1}H NMR (400 MHz, $CDCl_3$) δ 7.18 (t, *J* = 8.0 Hz, 1H), 7.14 (d, *J* = 0.8 Hz, 1H), 7.01 (s, 1H), 6.96 (d, *J* = 7.6 Hz, 1H), 6.93 – 6.89 (m, 1H), 6.75 (dd, *J* = 8.0, 2.4 Hz, 1H), 6.53 (d, *J* = 16.0 Hz, 1H), 6.45 (dt, *J* = 15.9, 6.4 Hz, 1H), 4.02 (d, *J* = 6.4 Hz, 2H), 3.96 (s, 3H), 3.77 (s, 3H).
^{13}C NMR (100 MHz, $CDCl_3$) δ 190.3, 159.8, 142.6, 138.6, 133.6, 129.5, 129.3, 127.3, 122.9, 119.0, 113.3, 111.4, 55.2, 43.1, 36.2.
HRMS (ESI-TOF) calcd for $C_{15}H_{17}N_2O_2^+$ ($[M]+H^+$) = 257.1285, Found 257.1279.
IR (film): 1673, 1464, 1403, 1260, 1155, 1043, 983, 914 cm^{-1}.

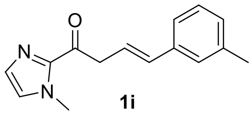

1i

(*E*)-1-(1-Methyl-1H-imidazol-2-yl)-4-(m-tolyl)but-3-en-1-one
^{1}H NMR (400 MHz, $CDCl_3$) δ 7.20 (s, 1H), 7.17 (d, *J* = 0.8 Hz, 1H), 7.17 – 7.16 (m, 1H), 7.15 (d, *J* = 0.8 Hz, 1H), 7.02 – 6.98 (m, 2H), 6.54 (d, *J* = 16.0 Hz, 1H), 6.50 – 6.41 (m, 1H), 4.04 (d, *J* = 0.8 Hz, 1H), 4.02 (d, *J* = 0.4 Hz, 1H), 3.96 (s, 3H), 2.31 (s, 3H).
^{13}C NMR (100 MHz, $CDCl_3$) δ 190.4, 142.6, 138.0, 137.1, 133.7, 129.2, 128.4, 128.2, 127.2, 126.9, 123.5, 122.3, 43.1, 36.1, 21.4.
HRMS (ESI-TOF) calcd for $C_{15}H_{17}N_2O^+$ ($[M]+H^+$) = 241.1335, Found 241.1337.
IR (film): 1673, 1471, 1403, 1287, 1154, 982, 913 cm^{-1}.

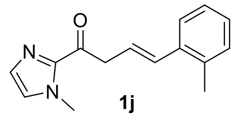

1j

(*E*)-1-(1-Methyl-1H-imidazol-2-yl)-4-(o-tolyl)but-3-en-1-one
^{1}H NMR (400 MHz, $CDCl_3$) δ 7.50 – 7.41 (m, 1H), 7.16 (s, 1H), 7.15 – 7.10 (m, 3H), 7.03 (s, 1H), 6.79 (d, *J* = 15.6 Hz, 1H), 6.35 (dt, *J* = 16.0, 6.8 Hz, 1H), 4.07 (d, *J* = 1.2 Hz, 1H), 4.05 (d, *J* = 1.2 Hz, 1H), 3.99 (s, 3H), 2.33 (s, 3H).
^{13}C NMR (100 MHz, $CDCl_3$) δ 190.5, 142.8, 136.3, 135.3, 131.7, 130.3, 129.3, 127.4, 127.3, 126.1, 125.8, 123.8, 43.6, 36.3, 19.9.
HRMS (ESI-TOF) calcd for $C_{15}H_{17}N_2O^+$ ($[M]+H^+$) = 241.1335, Found 241.1337.
IR (film): 1674, 1459, 1406, 1261, 1155, 984, 915 cm^{-1}.

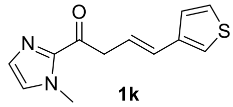

1k

(*E*)-4-(3-Methoxyphenyl)-1-(1-methyl-1H-imidazol-2-yl)but-3-en-1-one
^{1}H NMR (400 MHz, $CDCl_3$) δ 7.25 – 7.19 (m, 2H), 7.15 (d, *J* = 0.8 Hz, 1H), 7.13 – 7.09 (m, 1H), 7.03 (s, 1H), 6.57 (d, *J* = 15.6 Hz, 1H), 6.30 (dt, *J* = 16.0, 7.2 Hz, 1H), 4.00 (d, *J* = 1.6 Hz, 1H), 3.99 – 3.97 (m, 4H).
^{13}C NMR (100 MHz, $CDCl_3$) δ 190.5, 142.7, 139.9, 129.4, 128.0, 127.3, 125.9, 125.1, 122.4, 121.8, 43.1, 36.3.
HRMS (ESI-TOF) calcd for $C_{12}H_{13}N_2OS^+$ ($[M]+H^+$) = 233.0743, Found 233.0745.
IR (film): 1672, 1471, 1402, 1290, 1154, 983, 914 cm^{-1}.

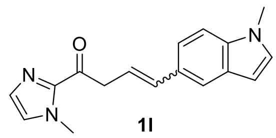

1l

1-(1-Methyl-1H-imidazol-2-yl)-4-(1-methyl-1H-indol-5-yl)but-3-en-1-one (2.5/1 *E*/*Z*)

^{1}H NMR (400 MHz, $CDCl_3$) for *E*-isomer: δ 7.59 (s, 1H), 7.34 (dd, *J* = 8.4, 1.2 Hz, 1H), 7.28 – 7.20 (m, 2H), 7.17 (d, *J* = 0.8 Hz, 1H), 7.05 – 6.96 (m, 2H), 6.69 (d, *J* = 16.0 Hz, 1H), 6.48 – 6.35 (m, 2H), 4.05 (dd, *J* = 7.2, 1.2 Hz, 2H), 3.99 (s, 3H), 3.75 (s, 3H).

^{13}C NMR (100 MHz, $CDCl_3$) for E-isomer δ 191.0, 142.9, 136.5, 134.9, 129.3, 129.0, 128.7, 127.2, 123.1, 121.2, 120.1, 119.4, 109.3, 101.3, 43.4, 36.3, 33.0.

HRMS (ESI-TOF) calcd for $C_{17}H_{18}N_3O^+$ ($[M]+H^+$) = 280.1444, Found 280.1444.

IR (film): 1673, 1484, 1405, 1291, 1246, 1154, 1081, 983, 915 cm^{-1}.

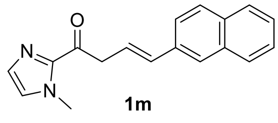

1m

(*E*)-1-(1-Methyl-1H-imidazol-2-yl)-4-(naphthalen-2-yl)but-3-en-1-one

^{1}H NMR (400 MHz, $CDCl_3$) δ 7.80 – 7.70 (m, 4H), 7.62 (dd, *J* = 8.8, 2.0 Hz, 1H), 7.47 – 7.38 (m, 2H), 7.18 (d, *J* = 0.8 Hz, 1H), 7.04 (s, 1H), 6.74 (d, *J* = 16.0 Hz, 1H), 6.61 (dt, *J* = 15.6, 7.2 Hz, 1H), 4.11 (d, *J* = 0.8 Hz, 1H), 4.09 (d, *J* = 1.2 Hz, 1H), 4.00 (s, 3H).

^{13}C NMR (100 MHz, $CDCl_3$) δ 190.5, 142.8, 134.8, 133.9, 133.7, 133.0, 129.4, 128.2, 128.1, 127.8, 127.4, 126.3, 126.2, 125.8, 123.7, 123.1, 43.4, 36.3.

HRMS (ESI-TOF) calcd for $C_{18}H_{17}N_2O^+$ ($[M]+H^+$) = 277.1335, Found 277.1338.

IR (film): 1675, 1467, 1406, 1269, 1154, 983, 915 cm^{-1}.

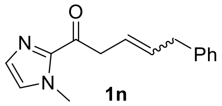

1n

1-(1-Methyl-1H-imidazol-2-yl)-5-phenylpent-3-en-1-one (8/1 *Z*/*E*)

^{1}H NMR (400 MHz, $CDCl_3$) δ 7.29 – 7.24 (m, 2H), 7.21 – 7.16 (m, 3H), 7.13 (d, *J* = 0.8 Hz, 1H), 7.01 (s, 1H), 5.81 – 5.76 (m, 2H), 3.97 (s, 3H), 3.90 – 3.85 (m, 2H), 3.40 (d, *J* = 3.2 Hz, 2H).

^{13}C NMR (100 MHz, $CDCl_3$) δ 190.9, 142.8, 140.4, 133.4, 129.3, 128.6, 128.5, 127.1, 126.1, 123.8, 42.7, 39.2, 36.2.

HRMS (ESI-TOF) calcd for $C_{15}H_{17}N_2O^+$ ($[M]+H^+$) = 241.1335, Found 241.1338.

IR (film): 1674, 1464, 1405, 1285, 1155, 977, 916 cm^{-1}.

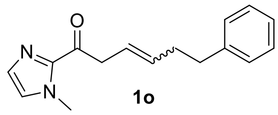

1o

1-(1-Methyl-1H-imidazol-2-yl)-6-phenylhex-3-en-1-one (3/1 *Z*/*E*)

^{1}H NMR (400 MHz, $CDCl_3$) δ 7.29 – 7.21 (m, 3H), 7.21 – 7.10 (m, 5H), 7.01 (s, 1H), 5.77 – 5.62 (m, 2H), 3.96 (s, 3H), 3.83 (d, *J* = 5.6 Hz, 2H), 2.73 – 2.65 (m, 2H), 2.40 – 2.33 (m, 2H).

^{13}C NMR (100 MHz, $CDCl_3$) δ 191.1, 142.7, 141.9, 134.1, 129.1, 128.5, 128.3, 127.1, 125.8, 122.8, 42.8, 36.2, 35.7, 34.5.

HRMS (ESI-TOF) calcd for $C_{16}H_{19}N_2O^+$ ($[M]+H^+$) = 255.1492, Found 255.1481.

IR (film): 1673, 1454, 1403, 1284, 1153, 1079, 970, 913 cm^{-1}.

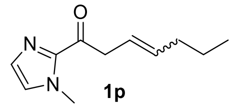

1p

1-(1-Methyl-1H-imidazol-2-yl)hept-3-en-1-one (3.3/1 *Z*/*E*)

^{1}H NMR (400 MHz, $CDCl_3$) δ 7.11 (s, 1H), 7.00 (s, 1H), 5.72 – 5.53 (m, 2H), 3.96 (d, *J* = 1.2 Hz, 3H), 3.79 (d, *J* = 5.2 Hz, 2H), 1.99 (dd, *J* = 12.8, 6.4 Hz, 2H), 1.41 – 1.31 (m, 2H), 0.90 – 0.82 (m, 3H).

^{13}C NMR (100 MHz, $CDCl_3$) δ 191.3, 142.8, 135.0, 129.2, 127.1, 122.2, 42.9, 34.8, 22.4, 13.7.

HRMS (ESI-TOF) calcd for $C_{11}H_{17}N_2O^+$ ($[M]+H^+$) = 193.1335, Found 193.1340.

IR (film): 1674, 1464, 1405, 1285, 1155, 977, 916 cm^{-1}.

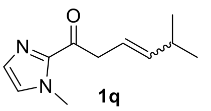

1q

5-Methyl-1-(1-methyl-1H-imidazol-2-yl)hex-3-en-1-one (5/1 *Z*/*E*)

^{1}H NMR (400 MHz, $CDCl_3$) δ 7.14 (d, *J* = 1.2 Hz, 1H), 7.05 (s, 1H), 5.65 – 5.55 (m, 2H), 4.00 (s, 3H), 3.85 – 3.80 (m, 2H), 2.37 – 2.26 (m, 1H), 1.00 (s, 3H), 0.98 (s, 3H), 0.96 (s, 1H).

^{13}C NMR (100 MHz, $CDCl_3$) δ 191.3, 142.7, 141.9, 129.0, 127.0, 119.1, 42.7, 36.1, 31.1, 22.3.

HRMS (ESI-TOF) calcd for $C_{11}H_{17}N_2O^+$ ([M]+H$^+$) = 193.1335, Found 193.1336.
IR (film): 1675, 1465, 1404, 1287, 1155, 976, 915 cm^{-1}.

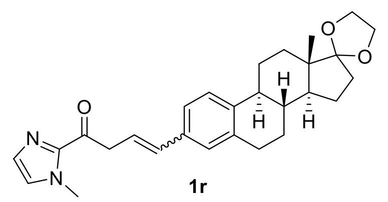

1r

1-(1-Methyl-1H-imidazol-2-yl)-4-((8R,9S,13S,14S)-13-methyl-6,7,8,9,11,12,13,14,15,16-decahydrospiro[cyclopenta[a]phenanthrene-17,2'-[1,3]dioxolan]-3-yl)but-3-en-1-one
^{1}**H NMR** (400 MHz, $CDCl_3$) ^{1}H NMR (400 MHz, $CDCl_3$) for *Z/E* isomer (*Z/E* = 2.5:1): δ 7.29 – 7.20 (m, 2H), 7.20 – 7.12 (m, 4H), 7.11 – 7.04 (m, 2H), 7.03 – 7.00 (m, 2H), 6.63 (d, *J* = 11.6 Hz, 1H) (*Z*-isomer), 6.51 (d, *J* = 16.0 Hz, 1H) (*E*-isomer), 6.41 (dt, *J* = 16.0, 6.8 Hz, 1H) (*E*-isomer), 5.98 (dt, *J* = 12.0, 6.8 Hz, 1H) (*Z*-isomer), 4.18 (dd, *J* = 7.2, 2.0 Hz, 2H), 4.03 – 4.00 (m, 2H), 4.00 – 3.97 (m, 5H), 3.96 – 3.86 (m, 8H), 2.88 – 2.81 (m, 4H), 2.34 – 2.21 (m, 5H), 2.08 – 1.98 (m, 2H), 1.92 – 1.74 (m, 8H), 1.69 – 1.58 (m, 2H), 1.56 – 1.33 (m, 10H), 0.88 (s, 3H), 0.87 (s, 3H).
^{13}**C NMR** (100 MHz, $CDCl_3$) for *Z*-isomer: δ 190.6, 142.8, 139.4, 136.7, 134.2, 132.1, 129.4, 129.2, 129.1, 128.3, 127.1, 126.0, 125.4, 123.0, 119.5, 65.3, 64.6, 49.5, 46.2, 44.1, 38.9, 34.3, 30.8, 29.6, 27.0, 26.0, 22.4, 14.4.
IR (film):2935, 2869, 1674, 1461, 1404, 1283, 1157, 1103, 1040, 913 cm^{-1}.

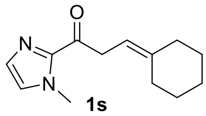

1s

3-Cyclohexylidene-1-(1-methyl-1H-imidazol-2-yl)propan-1-one
^{1}**H NMR** (400 MHz, $CDCl_3$) δ 7.14 (d, *J* = 0.8 Hz, 1H), 7.03 (s, 1H), 5.42 – 5.35 (m, 1H), 4.00 (s, 3H), 3.87 (d, *J* = 7.2 Hz, 2H), 2.22 – 2.18 (m, 2H), 2.17 – 2.13 (m, 2H), 1.57 – 1.49 (m, 6H).
^{13}**C NMR** (100 MHz, $CDCl_3$) δ 191.4, 143.8, 143.0, 129.1, 127.0, 112.6, 112.5, 37.8, 37.2, 36.3, 29.3, 28.6, 27.7, 26.8.
HRMS (ESI-TOF) calcd for $C_{13}H_{19}N_2O^+$ ([M]+H$^+$) = 219.1492, Found 209.1494.
IR (film): 1675, 1446, 1404, 1286, 1154, 1021, 975 cm^{-1}.

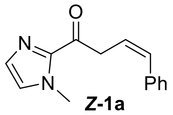

***Z*-1a**

(*Z*)-1-(1-Methyl-1H-imidazol-2-yl)-4-phenylbut-3-en-1-one
^{1}**H NMR** (400 MHz, $CDCl_3$) δ 7.37 – 7.28 (m, 4H), 7.23 – 7.18 (m, 1H), 7.11 (s, 1H), 6.98 (s, 1H), 6.68 (d, *J* = 11.6 Hz, 1H), 6.04 (dt, *J* = 11.6, 7.2 Hz, 1H), 4.17 (dd, *J* = 7.2, 1.6 Hz, 2H), 3.95 (s, 3H).
^{13}**C NMR** (100 MHz, $CDCl_3$) δ 190.2, 142.7, 136.8, 132.0, 129.1, 128.7, 128.3, 127.1, 127.0, 123.6, 38.6, 36.1.
HRMS (ESI-TOF) calcd for $C_{14}H_{15}N_2O^+$ ([M]+H$^+$) = 227.1179, Found 227.1180.
IR (film): 1674, 1472, 1404, 1287, 1155, 981, 915 cm^{-1}.

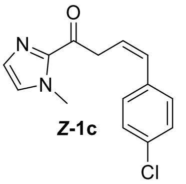

***Z*-1c**

(*Z*)-4-(4-Chlorophenyl)-1-(1-methyl-1H-imidazol-2-yl)but-3-en-1-one
^{1}**H NMR** (400 MHz, $CDCl_3$) δ 7.33 – 7.26 (m, 4H), 7.15 (s, 1H), 7.04 (s, 1H), 6.63 (d, *J* = 11.6 Hz, 1H), 6.07 (dt, *J* = 11.6, 7.2 Hz, 1H), 4.14 (dd, *J* = 7.2, 2.0 Hz, 2H), 4.01 (s, 3H).
^{13}**C NMR** (100 MHz, $CDCl_3$) δ 190.2, 142.8, 135.4, 132.9, 131.1, 130.1, 129.4, 128.6, 127.3, 124.5, 38.7, 36.3.
HRMS (ESI-TOF) calcd for $C_{14}H_{14}{}^{34.9689}ClN_2O^+$ ([M]+H$^+$) = 261.0789, Found 261.0791.
HRMS (ESI-TOF) calcd for $C_{14}H_{14}{}^{36.9659}ClN_2O^+$ ([M]+H$^+$) = 263.0760, Found 263.0759.
IR (film): 1676, 1489, 1406, 1287, 1155, 1089, 1041, 982, cm^{-1}.

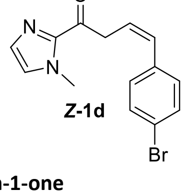

***Z*-1d**

(*Z*)-4-(4-Bromophenyl)-1-(1-methyl-1H-imidazol-2-yl)but-3-en-1-one
^{1}**H NMR** (400 MHz, $CDCl_3$) δ 7.49 – 7.42 (m, 2H), 7.23 (d, *J* = 8.4 Hz, 2H), 7.14 (s, 1H), 7.04 (s, 1H), 6.61 (d, *J* = 11.6 Hz, 1H), 6.07 (dt, *J* = 11.6, 7.2 Hz, 1H), 4.13 (dd, *J* = 7.2, 1.6 Hz, 2H), 4.00 (s, 3H).

^{13}C NMR (100 MHz, $CDCl_3$) δ 190.1, 142.8, 135.8, 131.6, 131.1, 130.5, 129.4, 127.3, 124.6, 121.1, 38.7, 36.3.
HRMS (ESI-TOF) calcd for $C_{14}H_{14}{}^{78.9183}BrN_2O^+$ ([M]+H$^+$) = 305.0284, Found 305.0285.
HRMS (ESI-TOF) calcd for $C_{14}H_{14}{}^{80.9163}BrN_2O^+$ ([M]+H$^+$) = 307.0264, Found 307.0264.
IR (film): 1675, 1483, 1405, 1286, 1155, 1071, 1005, cm^{-1}.

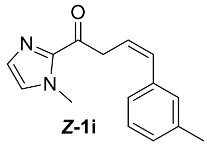

***Z*-1i**

(*Z*)-1-(1-Methyl-1H-imidazol-2-yl)-4-(m-tolyl)but-3-en-1-one
^{1}H NMR (400 MHz, $CDCl_3$) δ 7.27 – 7.12 (m, 4H), 7.08 – 6.99 (m, 2H), 6.66 (d, *J* = 11.6 Hz, 1H), 6.07 – 5.96 (m, 1H), 4.17 (dd, *J* = 7.2, 1.6 Hz, 2H), 4.02 –3.97 (m, 3H), 2.34 (s, 3H).
^{13}C NMR (100 MHz, $CDCl_3$) δ 190.6, 142.9, 137.9, 136.9, 132.3, 129.6, 129.3, 128.3, 127.9, 127.2, 125.8, 123.5, 38. 8, 36.3, 21.5.
HRMS (ESI-TOF) calcd for $C_{15}H_{17}N_2O^+$ ([M]+H$^+$) = 241.1335, Found 241.1337.
IR (film): 1670, 1613, 1405, 1287, 1154, 983, cm^{-1}.

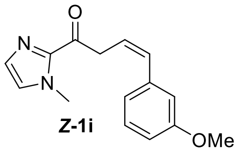

***Z*-1i**

(*Z*)-4-(3-Methoxyphenyl)-1-(1-methyl-1H-imidazol-2-yl)but-3-en-1-one
^{1}H NMR (400 MHz, $CDCl_3$) δ 7.25 (t, *J* = 7.6 Hz, 1H), 7.13 (d, *J* = 0.8 Hz, 1H), 7.02 (s, 1H), 6.95 (d, *J* = 8.0 Hz, 1H), 6.92 (d, *J* = 2.0 Hz, 1H), 6.79 (dd, *J* = 8.4, 2.4 Hz, 1H), 6.67 (d, *J* = 11.6 Hz, 1H), 6.08 – 5.99 (m, 1H), 4.17 (dd, *J* = 7.2, 1.6 Hz, 2H), 3.99 (s, 3H), 3.80 (s, 3H).
^{13}C NMR (100 MHz, $CDCl_3$) δ 190.4, 159.6, 142.8, 138.3, 132.2, 129.4, 129.3, 127.2, 124.0, 121.2, 114.2, 112.9, 55.3, 38.9, 36.2.
HRMS (ESI-TOF) calcd for $C_{15}H_{17}N_2O_2^+$ ([M]+H$^+$) = 257.1285, Found 257.1286.
IR (film): 1673, 1599, 1406, 1258, 1154, 1043, 985, cm^{-1}.

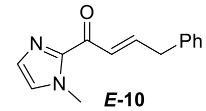

***E*-10**

(*E*)-1-(1-methyl-1H-imidazol-2-yl)-4-phenylbut-2-en-1-one
^{1}H NMR (400 MHz, $CDCl_3$) δ 7.50 – 7.43 (m, 1H), 7.34 – 7.27 (m, 2H), 7.25 – 7.18 (m, 4H), 7.17 (s, 1H), 7.04 (s, 1H), 4.03 (s, 3H), 3.64 (d, *J* = 7.2 Hz, 2H).
^{13}C NMR (100 MHz, $CDCl_3$) δ 180.7, 146.5, 143.8, 138.2, 129.4, 128.9, 128.8, 127.3, 127.2, 126.7, 39.1, 36.4.
HRMS (ESI-TOF) calcd for $C_{14}H_{15}N_2O^+$ ([M]+H$^+$) = 227.1179, Found 227.1181.
IR (film): 1664, 1617, 1406, 1288, 1156, 1017, 916 cm^{-1}.

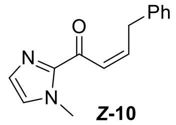

***Z*-10**

(*Z*)-1-(1-methyl-1H-imidazol-2-yl)-4-phenylbut-2-en-1-one
^{1}H NMR (400 MHz, $CDCl_3$) δ 7.44 (d, *J* = 11.2 Hz, 1H), 7.34 – 7.24 (m, 4H), 7.21 (t, *J* = 6.8 Hz, 1H), 7.15 (s, 1H), 7.02 (s, 1H), 6.48 (dt, *J* = 11.6, 7.6 Hz, 1H), 4.12 (d, *J* = 7.2 Hz, 2H), 4.06 (s, 3H).
^{13}C NMR (100 MHz, $CDCl_3$) δ 182.4, 148.1, 144.4, 139.9, 129.1, 128.7, 128.7, 127.2, 126.4, 124.6, 36.5, 35.9.
HRMS (ESI-TOF) calcd for $C_{14}H_{15}N_2O^+$ ([M]+H$^+$) = 227.1179, Found 227.1172.
IR (film): 1657, 1609, 1419, 1382, 1233, 1155, 995, 914 cm^{-1}.

(2*S*,3*R*)-3-Benzyl-1,5-bis(1-methyl-1H-imidazol-2-yl)-2-((*E*)-styryl)pentane-1,5-dione

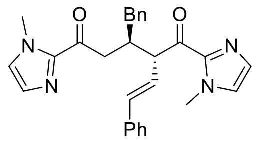

E-(2*S*,3*R*)-**2a**

Colourless foam. 74% yield (98% ee, 10:1 dr) was obtained according to the general procedure at 25 °C for 12 h.
HPLC (Chiral IE column), *i*-PrOH/*n*-Hexane = 30/70, Flow rate: 1.0 mL/min, 254 nm, t_r (minor) = 42.29 min, t_r (major) = 33.26 min.
^{1}H NMR (400 MHz, $CDCl_3$) δ 7.21 – 7.11 (m, 10H), 7.10 – 7.04 (m, 1H), 7.00 (s, 2H), 6.73 (s, 1H), 6.51 (d, *J* = 16.0 Hz, 1H), 6.20 (dd, *J* = 15.6, 10.0 Hz, 1H), 4.80 (t, *J* = 9.2 Hz, 1H), 3.90 (s, 3H), 3.56 (s, 3H), 3.52 – 3.36 (m, 2H), 2.94 (dd, *J* = 16.4, 5.2 Hz, 1H), 2.84 (dd, *J* = 13.6, 4.8 Hz, 1H), 2.60 (dd, *J* = 14.0, 9.2 Hz, 1H).
^{13}C NMR (100 MHz, $CDCl_3$) δ 192.6, 192.0, 143.3, 143.2, 140.0, 136.8, 134.2, 129.5, 129.3, 128.6, 128.3, 128.1, 127.6, 127.5, 127.5, 126.6, 126.4, 126.1, 40.7, 40.2, 39.5, 36.3, 35.8.
HRMS (ESI-FT) calculated for $C_{28}H_{29}N_4O_2^+$ ([M]+H$^+$) = 453.2285, Found 453.2285.

$[\alpha]_D^{26}$ = +46.2 (c = 0.61, in CH_2Cl_2).
IR (film): 1675, 1600, 1507, 1404, 1288, 1224, 1156, 984 cm^{-1}.
Chiral HPLC spectrum of (2*S*,3*R*)-**2a**:

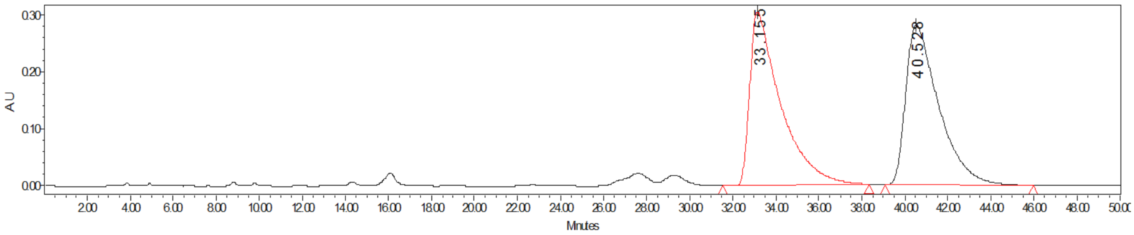

	Retention Time	Area	% Area
1	33.155	30308191	50.35
2	40.528	29890856	49.65

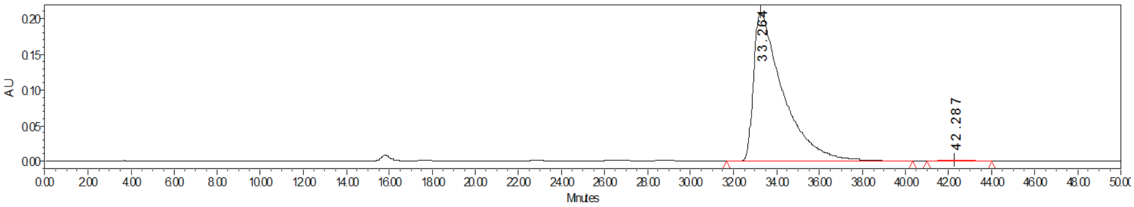

	Retention Time	Area	% Area
1	33.264	19829285	99.15
2	42.287	169096	0.85

(2*R*,3*S*)-3-Benzyl-1,5-bis(1-methyl-1H-imidazol-2-yl)-2-((E)-styryl)pentane-1,5-dione

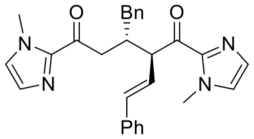

E-(2*R*,3*S*)-**2a**

Colourless foam. 84% yield (98% ee, 19:1 dr) was obtained according to the general procedure at 25 °C for 2 h.
HPLC (Chiral IE column), *i*-PrOH/*n*-Hexane = 30/70, Flow rate: 1.0 mL/min, 254 nm, t_r (minor) = 36.72 min, t_r (major) = 41.85 min.
^{1}H NMR (400 MHz, $CDCl_3$) δ 7.21 – 7.11 (m, 10H), 7.10 – 7.05 (m, 1H), 6.99 (d, J = 2.4 Hz, 2H), 6.73 (s, 1H), 6.51 (d, J = 16.0 Hz, 1H), 6.20 (dd, J = 15.6, 10.0 Hz, 1H), 4.81 (t, J = 9.2 Hz, 1H), 3.90 (s, 3H), 3.56 (s, 3H), 3.52 – 3.36 (m, 2H), 2.95 (dd, J = 16.4, 5.2 Hz, 1H), 2.84 (dd, J = 14.0, 4.8 Hz, 1H), 2.60 (dd, J = 13.6, 9.2 Hz, 1H).
^{13}C NMR (100 MHz, $CDCl_3$) δ 192.6, 192.0, 143.4, 143.2, 140.0, 136.8, 134.2, 129.5, 129.3, 128.6, 128.3, 128.0, 127.6, 127.5, 127.5, 126.5, 126.4, 126.1, 56.7, 40.7, 40.2, 39.5, 36.3, 35.8.
HRMS (ESI-FT) calculated for $C_{28}H_{29}N_4O_2^+$ ([M]+H$^+$) = 453.2285, Found 453.2290.
$[\alpha]_D^{23}$ = –40.8 (c = 1.18, in CH_2Cl_2).
IR (film): 1668, 1402, 1287, 1154, 986 cm^{-1}.
Chiral HPLC spectrum of (2*R*,3*S*)-**2a**:

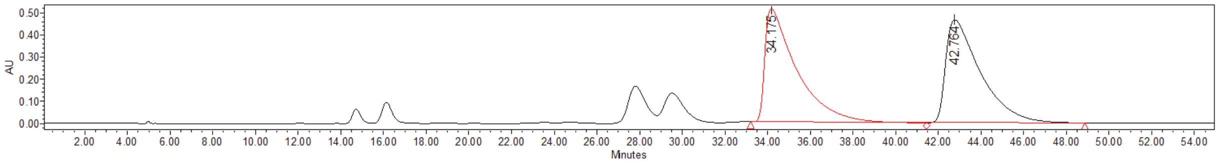

	Retention Time	Area	% Area
1	34.175	52319338	50.09
2	42.764	52129699	49.91

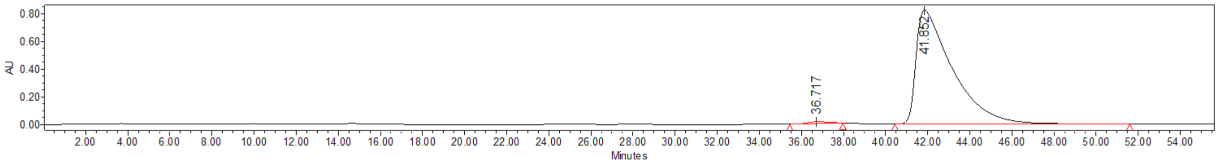

	Retention Time	Area	% Area
1	36.717	886611	0.89
2	41.852	98870573	99.11

(2*R*,3*R*)-3-Benzyl-1,5-bis(1-methyl-1H-imidazol-2-yl)-2-((*E*)-styryl)pentane-1,5-dione

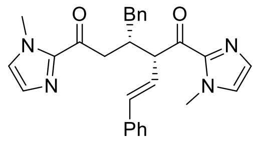

E-(2*S*,3*S*)-**2a**

Colourless foam. 67% yield (>99% ee, 8:1 dr) was obtained according to the general procedure at 25 °C for 2 h.
HPLC (Chiral IG column), *i*-PrOH/*n*-Hexane = 30/70, Flow rate: 1.0 mL/min, 254 nm, t_r (minor) = 23.50 min, t_r (major) = 20.94 min.
^{1}H NMR (400 MHz, $CDCl_3$) δ 7.31 (d, *J* = 7.6 Hz, 2H), 7.26 (t, *J* = 6.8 Hz, 2H), 7.21 – 7.10 (m, 6H), 7.09 – 7.04 (m, 1H), 6.99 (s, 1H), 6.95 (s, 1H), 6.87 (s, 1H), 6.65 (d, *J* = 16.0 Hz, 1H), 6.34 (dd, *J* = 16.0, 9.6 Hz, 1H), 4.89 (t, *J* = 8.0 Hz, 1H), 3.90 (s, 3H), 3.77 (s, 3H), 3.34 – 3.21 (m, 2H), 3.09 (dd, *J* = 13.2, 4.4 Hz, 2H), 2.68 – 2.59 (m, 1H).
^{13}C NMR (100 MHz, $CDCl_3$) δ 192.5, 191.8, 143.4, 143.0, 140.3, 137.1, 134.5, 129.7, 129.3, 128.7, 128.5, 128.1, 127.6, 127.4, 127.0, 126.6, 126.5, 125.9, 54.9, 40.7, 39.2, 38.5, 36.3, 36.1.
HRMS (ESI-FT) calculated for $C_{28}H_{29}N_4O_2^+$ ([M]+H$^+$) = 453.2285, Found 453.2295.
$[\alpha]_D^{23}$ = +71.7 (*c* = 1.33, in CH_2Cl_2).
IR (film): 1668, 1403, 1287, 1154, 976 cm^{-1}.
Chiral HPLC spectrum of (2*S*,3*S*)-**2a**:

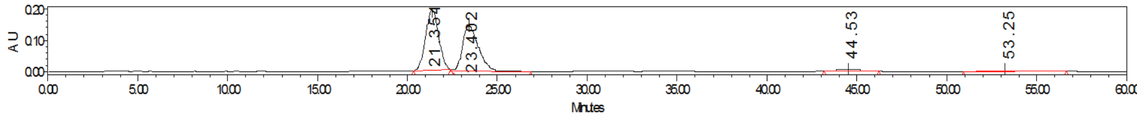

	Retention Time	Area	% Area
1	21.354	9705046	49.02
2	23.402	9061556	45.77
3	44.533	507920	2.57
4	53.259	522673	2.64

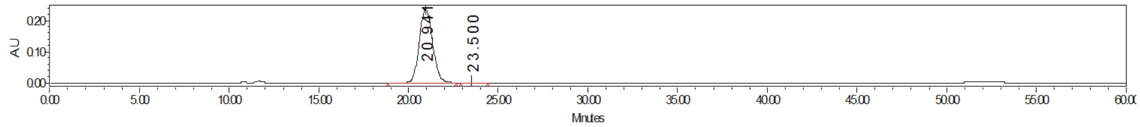

	Retention Time	Area	% Area
1	20.941	12102193	99.75
2	23.500	29872	0.25

(2*S*,3*S*)-3-Benzyl-1,5-bis(1-methyl-1H-imidazol-2-yl)-2-((*E*)-styryl)pentane-1,5-dione

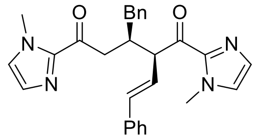

E-(2*R*,3*R*)-**2a**

Colourless foam. 69% yield (99% ee, 9:1 dr) was obtained according to the general procedure at 25 °C for 2 h.
HPLC (Chiral IG column), *i*-PrOH/*n*-Hexane = 30/70, Flow rate: 1.0 mL/min, 254 nm, t_r (minor) = 20.39 min, t_r (major) = 22.06 min.
^{1}H NMR (400 MHz, $CDCl_3$) δ 7.31 (d, *J* = 7.2 Hz, 2H), 7.26 (t, *J* = 6.8 Hz, 2H), 7.19 (d, *J* = 7.2 Hz, 1H), 7.17 – 7.10 (m, 5H), 7.09 – 7.04 (m, 1H), 6.99 (s, 1H), 6.95 (s, 1H), 6.87 (s, 1H), 6.65 (d, *J* = 16.0 Hz, 1H), 6.34 (dd, *J* = 15.6, 9.6 Hz, 1H), 4.96 – 4.89 (t, *J* = 8.0 Hz, 1H), 3.90 (s, 3H), 3.77 (s, 3H), 3.34 – 3.19 (m, 2H), 3.14 – 3.04 (m, 2H), 2.63 (dd, *J* = 14.0, 9.2 Hz, 1H).
^{13}C NMR (100 MHz, $CDCl_3$) δ 192.5, 191.8, 143.4, 143.0, 140.3, 137.1, 134.5, 129.7, 129.3, 128.7, 128.5, 128.1, 127.6, 127.4, 127.0, 126.6, 126.5, 125.9, 54.9, 40.7, 39.2, 38.5, 36.3, 36.1.
HRMS (ESI-FT) calculated for $C_{28}H_{29}N_4O_2^+$ ([M]+H$^+$) = 453.2285, Found 453.2292.
$[\alpha]_D^{23}$ = –64.8 (*c* = 1.34, in CH_2Cl_2).
IR (film): 1669, 1405, 1287, 1155, 977 cm^{-1}.
Chiral HPLC spectrum of (2*R*,3*R*)-**2a**:

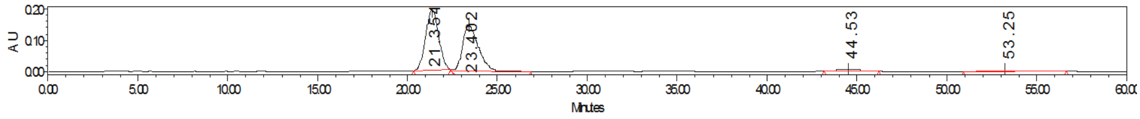

	Retention Time	Area	% Area
1	21.354	9705046	49.02
2	23.402	9061556	45.77
3	44.533	507920	2.57
4	53.259	522673	2.64

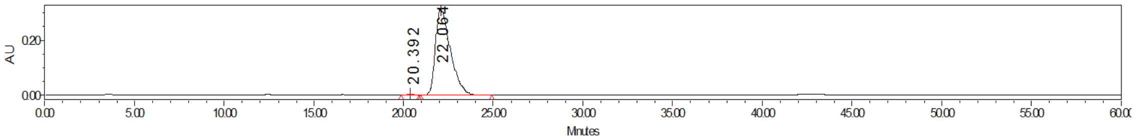

	Retention Time	Area	% Area
1	20.392	103983	0.56
2	22.064	18333201	99.44

(2*S*,3*R*)-3-(4-fluorobenzyl)-2-((*E*)-4-fluorostyryl)-1,5-bis(1-methyl-1H-imidazol-2-yl)pentane-1,5-dione

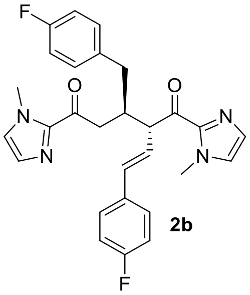

2b

Colourless foam. 74% yield (99% ee, 11:1 dr) was obtained according to the general procedure at 25 °C for 24 h.
HPLC (Chiral IE column), *i*-PrOH/*n*-Hexane = 30/70, Flow rate: 1.0 mL/min, 254 nm, t_r (minor) = 27.58 min, t_r (major) = 22.16 min.
^{1}H NMR (400 MHz, $CDCl_3$) δ 7.17 – 7.12 (m, 3H), 7.11 – 7.04 (m, 2H), 7.01 (s, 2H), 6.91 – 6.77 (m, 5H), 6.47 (d, *J* = 16.0 Hz, 1H), 6.10 (dd, *J* = 16.0, 10.0 Hz, 1H), 4.76 (t, *J* = 9.2 Hz, 1H), 3.91 (s, 3H), 3.63 (s, 3H), 3.47 – 3.30 (m, 2H), 2.94 (dd, *J* = 16.4, 5.6 Hz, 1H), 2.78 (dd, *J* = 14.0, 5.2 Hz, 1H), 2.58 (dd, *J* = 13.6, 8.8 Hz, 1H).
^{13}C NMR (100 MHz, $CDCl_3$) δ 192.4, 191.9, 163.1 (d, *J* = 90.0 Hz), 160.7 (d, *J* = 86.0 Hz), 143.3, 143.1, 135.5 (d, *J* = 3.0 Hz), 133.1, 132.98 (d, *J* = 3.0 Hz), 130.99 (d, *J* = 7.0 Hz), 129.41, 128.78, 127.90 (d, *J* = 8.0 Hz), 127.65, 127.14 (d, *J* = 2.0 Hz), 126.69, 115.21 (d, *J* = 22.0 Hz), 114.7 (d, *J* = 20.0 Hz), 56.4, 40.6, 39.5, 39.4, 36.3, 35.9.
^{19}F NMR (376 MHz, $CDCl_3$) δ –115.01 (s, 1F), –117.99 (s, 1F).
HRMS (ESI-FT) calculated for $C_{28}H_{27}F_2N_4O_2^+$ ([M]+H$^+$) = 489.2097, Found 489.2098.
$[\alpha]_D^{27}$ = +37.4 (*c* = 0.72, in CH_2Cl_2).
IR (film): 1667, 1506, 1400, 1287, 1222, 1156, 975, 831 cm^{-1}.
Chiral HPLC spectrum of **2b**:

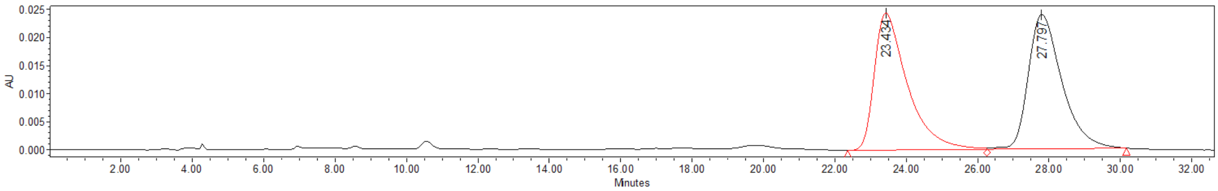

	Retention Time	Area	% Area
1	23.434	1572462	50.20
2	27.797	1560109	49.80

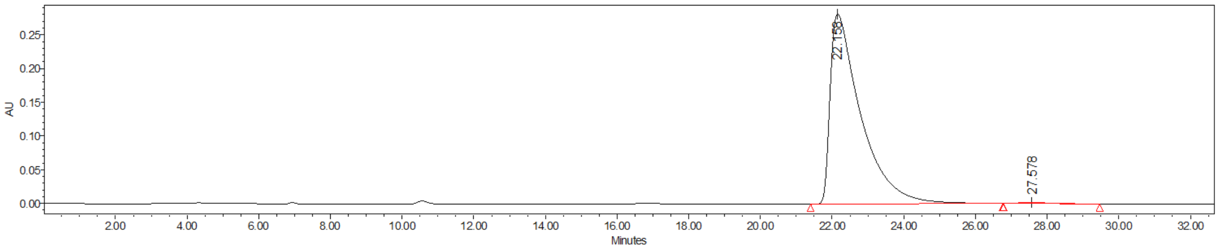

	Retention Time	Area	% Area
1	22.158	16683755	99.47
2	27.578	88881	0.53

(2*S*,3*R*)-3-(4-chlorobenzyl)-2-((*E*)-4-chlorostyryl)-1,5-bis(1-methyl-1H-imidazol-2-yl)pentane-1,5-dione

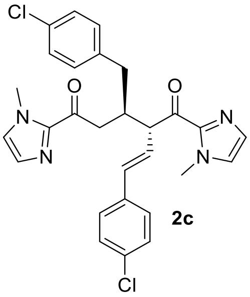

2c

Colourless foam. 72% yield (98% ee, 7.5:1 dr) was obtained according to the general procedure at 25 °C for 24 h.
HPLC (Chiral IE column), *i*-PrOH/*n*-Hexane = 30/70, Flow rate: 1.0 mL/min, 254 nm, t_r (minor) = 35.87 min, t_r (major) = 25.03 min.
^{1}H NMR (400 MHz, $CDCl_3$) δ 7.18 – 7.13 (m, 3H), 7.12 – 7.02 (m, 6H), 7.02 – 6.98 (m, 2H), 6.80 (s, 1H), 6.47 (d, *J* = 16.0 Hz, 1H), 6.16 (dd, *J* = 16.0, 10.0 Hz, 1H), 4.76 (t, *J* = 9.2 Hz, 1H), 3.89 (s, 3H), 3.63 (s, 3H), 3.43 – 3.30 (m, 2H), 3.02 – 2.91 (m, 1H), 2.76 (dd, *J* = 14.0, 5.2 Hz, 1H), 2.58 (dd, *J* = 13.6, 8.8 Hz, 1H).
^{13}C NMR (100 MHz, $CDCl_3$) δ 192.2, 191.8, 143.2, 143.1, 138.3, 135.3, 133.2, 133.1, 131.9, 131.0, 129.4, 128.8, 128.5, 128.1, 128.0, 127.7, 127.6, 126.7, 56.4, 40.7, 39.7, 39.4, 36.3, 35.9.
HRMS (ESI-FT) calculated for $C_{28}H_{27}{}^{34.9689}Cl_2N_4O_2^+$ ([M]+H$^+$) = 521.1506, Found 521.1506.
HRMS (ESI-FT) calculated for $C_{28}H_{27}{}^{36.9659}Cl_2N_4O_2^+$ ([M]+H$^+$) = 523.1476, Found 523.1478.
$[\alpha]_D^{27}$ = +43.0 (*c* = 1.56, in CH_2Cl_2).
IR (film): 1667, 1489, 1399, 1285, 1089, 975 cm^{-1}.
Chiral HPLC spectrum of **2c**:

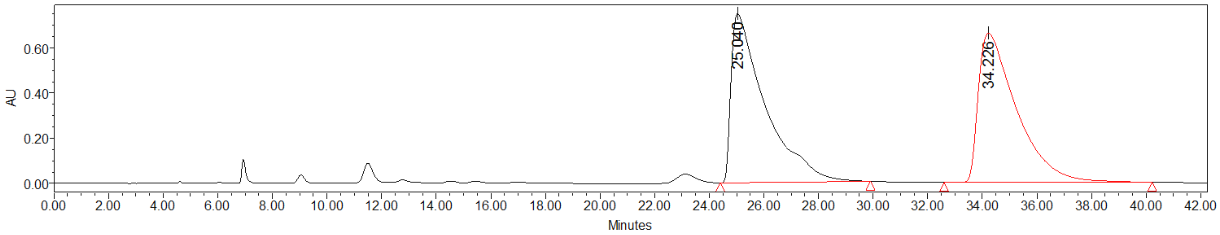

	Retention Time	Area	% Area
1	25.040	64808948	50.18
2	34.226	64338432	49.82

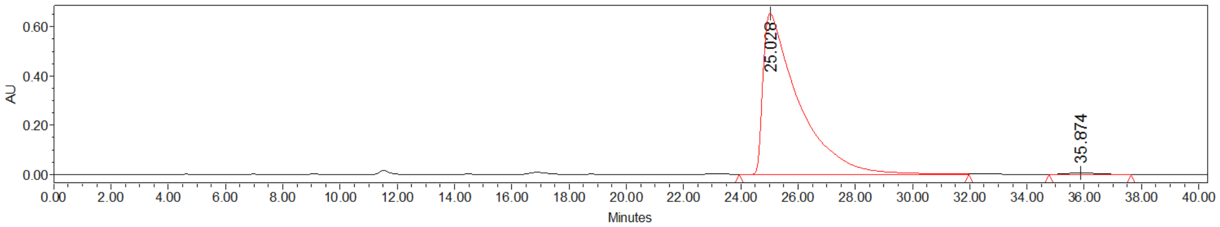

	Retention Time	Area	% Area
1	25.028	56129367	99.14
2	35.874	489544	0.86

(2*S*,3*R*)-3-(4-bromobenzyl)-2-((*E*)-4-bromostyryl)-1,5-bis(1-methyl-1H-imidazol-2-yl)pentane-1,5-dione

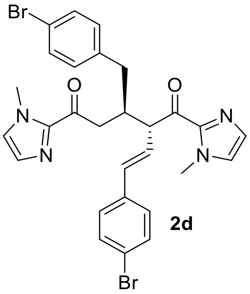

2d

Colourless foam. 73% yield (98% ee, 8:1 dr) was obtained according to the general procedure at 25 °C for 24 h.
HPLC (Chiral IE column), *i*-PrOH/*n*-Hexane = 30/70, Flow rate: 1.0 mL/min, 254 nm, t_r (minor) = 42.64 min, t_r (major) = 27.89 min.
^{1}H NMR (400 MHz, $CDCl_3$) δ 7.31 (d, *J* = 8.4 Hz, 2H), 7.21 (d, *J* = 8.4 Hz, 2H), 7.14 (s, 1H), 7.07 – 6.95 (m, 6H), 6.80 (s, 1H), 6.45 (d, *J* = 15.6 Hz, 1H), 6.18 (dd, *J* = 16.0, 10.0 Hz, 1H), 4.76 (t, *J* = 8.8 Hz, 1H), 3.89 (s, 3H), 3.64 (s, 3H), 3.44 – 3.31 (m, 2H), 3.03 – 2.92(m, 1H), 2.74 (dd, *J* = 14.0, 5.2 Hz, 1H), 2.57 (dd, *J* = 13.6, 8.8 Hz, 1H).
^{13}C NMR (100 MHz, $CDCl_3$) δ 192.1, 191.7, 143.2, 143.0, 138.8, 135.7, 133.2, 131.5, 131.4, 131.0, 129.5, 128.8, 128.3, 127.9, 127.7, 126.8, 121.3, 120.0, 56.4, 40.7, 39.8, 39.4, 36.3, 35.9.
HRMS (ESI-FT) calculated for $C_{28}H_{27}{}^{78.9183}Br_2N_4O_2^+$ ([M]+H$^+$) = 609.0495, Found 609.0492.

HRMS (ESI-FT) calculated for $C_{28}H_{27}{}^{80.9163}Br_2N_4O_2^+$ ([M]+H$^+$) = 611.0475, Found 611.0462.
$[\alpha]_D^{27}$ = +37.3 (c = 1.28, in CH_2Cl_2).
IR (film): 1668, 1485, 1402, 1286, 1071, 1007, 985 cm^{-1}.
Chiral HPLC spectrum of **2d**:

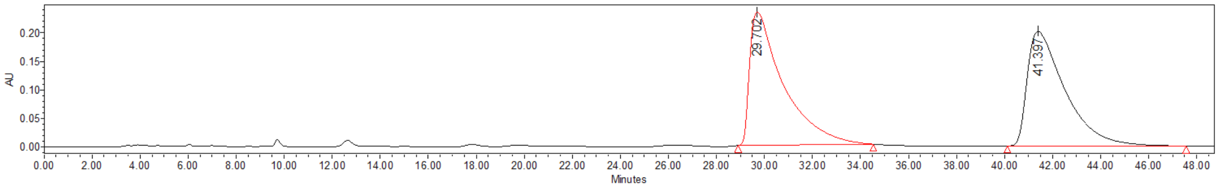

	Retention Time	Area	% Area
1	29.702	22754079	50.90
2	41.397	21946732	49.10

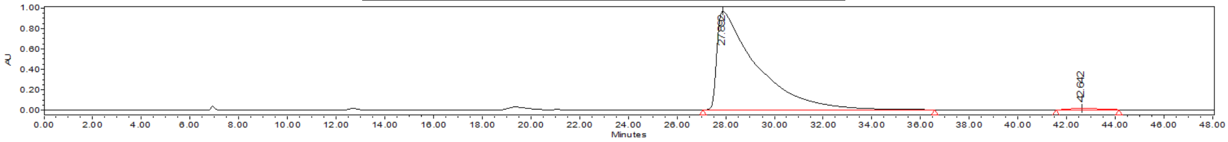

	Retention Time	Area	% Area
1	27.892	108918443	98.89
2	42.642	1217018	1.11

(2*S*,3*R*)-2-((*E*)-2-([1,1'-biphenyl]-4-yl)vinyl)-3-([1,1'-biphenyl]-4-ylmethyl)-1,5-bis(1-methyl-1H-imidazol-2-yl)pentane-1,5-dione

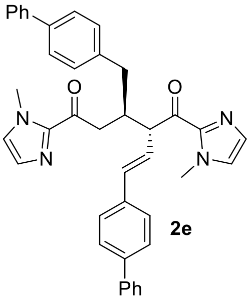

2e

Colourless foam. 81% yield (99% ee, 11:1 dr) was obtained according to the general procedure at 25 °C for 24 h.
HPLC (Chiral IA column), *i*-PrOH/*n*-Hexane = 30/70, Flow rate: 1.0 mL/min, 254 nm, t_r (minor) = 33.58 min, t_r (major) = 22.79 min.
^{1}**H NMR** (400 MHz, $CDCl_3$) δ 7.57 – 7.49 (m, 4H), 7.47 – 7.35 (m, 8H), 7.34 – 7.24 (m, 5H), 7.21 (d, J = 8.0 Hz, 2H), 7.16 (s, 1H), 7.00 (d, J = 14.8 Hz, 2H), 6.72 (s, 1H), 6.59 (d, J = 16.0 Hz, 1H), 6.27 (dd, J = 16.0, 10.0 Hz, 1H), 4.86 (t, J = 8.8 Hz, 1H), 3.88 (s, 3H), 3.57 (s, 3H), 3.53 – 3.43 (m, 2H), 3.06 (dd, J = 18.8, 8.4 Hz, 1H), 2.89 (dd, J = 13.6, 4.8 Hz, 1H), 2.68 (dd, J = 14.0, 8.8 Hz, 1H).
^{13}**C NMR** (100 MHz, $CDCl_3$) δ 192.5, 192.0, 143.3, 143.2, 141.0, 140.7, 140.2, 139.1, 138.8, 135.9, 133.8, 130.1, 129.3, 128.9, 128.8, 128.7, 127.8, 127.5, 127.4, 127.1, 127.0, 126.9, 126.8, 126.6, 126.6, 56.7, 40.9, 40.0, 39.6, 36.3, 35.8.
HRMS (ESI-FT) calculated for $C_{40}H_{37}N_4O_2^+$ ([M]+H$^+$) = 605.2911, Found 605.2907.
$[\alpha]_D^{27}$ = +57.4 (c = 0.98, in CH_2Cl_2).
IR (film): 1666, 1484, 1400, 1154, 975, 759 cm^{-1}.
Chiral HPLC spectrum of **2e**:

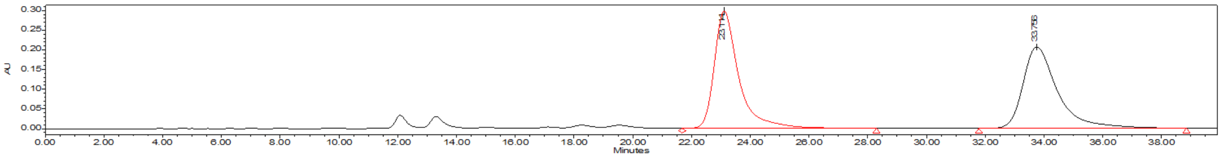

	Retention Time	Area	% Area
1	23.114	17272586	50.54
2	33.756	16904029	49.46

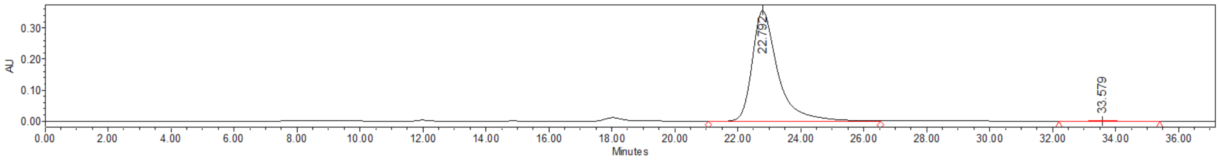

	Retention Time	Area	% Area
1	22.792	20259894	99.51
2	33.579	99123	0.49

(2*S*,3*R*)-1,5-bis(1-methyl-1H-imidazol-2-yl)-3-(4-methylbenzyl)-2-((*E*)-4-methylstyryl)pentane-1,5-dione

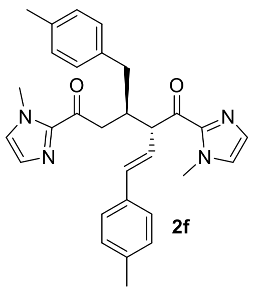

2f

Colourless foam. 78% yield (>99% ee, 8.5:1 dr) was obtained according to the general procedure at 25 °C for 24 h.
HPLC (Chiral IA column), *i*-PrOH/*n*-Hexane = 30/70, Flow rate: 1.0 mL/min, 254 nm, t_r (minor) = 65.98 min, t_r (major) = 38.14 min.
^{1}H NMR (400 MHz, $CDCl_3$) δ 7.13 (s, 1H), 7.08 (d, *J* = 8.0 Hz, 2H), 7.04 – 6.97 (m, 6H), 6.94 (d, *J* = 8.0 Hz, 2H), 6.74 (s, 1H), 6.48 (d, *J* = 16.0 Hz, 1H), 6.14 (dd, *J* = 16.0, 10.0 Hz, 1H), 4.77 (t, *J* = 9.2 Hz, 1H), 3.90 (s, 3H), 3.58 (s, 3H), 3.47 – 3.31 (m, 2H), 2.95 (dd, *J* = 16.4, 5.2 Hz, 1H), 2.79 (dd, *J* = 13.6, 4.8 Hz, 1H), 2.56 (dd, *J* = 13.6, 9.2 Hz, 1H), 2.27 (s, 3H), 2.23 (s, 3H).
^{13}C NMR (100 MHz, $CDCl_3$) δ 192.7, 192.1, 143.4, 143.2, 137.2, 136.8, 135.3, 134.1, 134.1, 129.5, 129.3, 129.0, 128.7, 128.6, 128.6, 127.4, 126.5, 126.3, 56.5, 40.7, 39.7, 39.6, 36.2, 35.8, 21.2, 21.0.
HRMS (ESI-FT) calculated for $C_{30}H_{33}N_4O_2^+$ ($[M]+H^+$) = 481.2598, Found 481.2598.
$[\alpha]_D^{27}$ = +46.2 (*c* = 0.75, in CH_2Cl_2).
IR (film): 1667, 1512, 1401, 1287, 1154, 975, 915 cm^{-1}.
Chiral HPLC spectrum of **2f**:

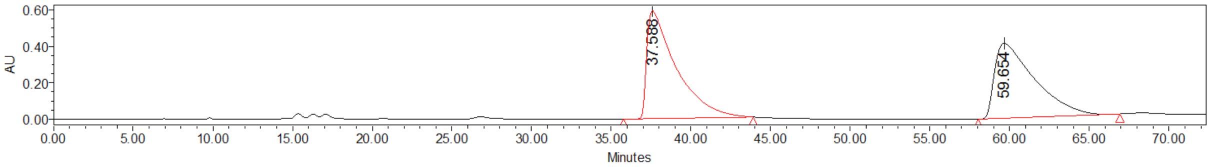

	Retention Time	Area	% Area
1	37.588	75894569	51.47
2	59.654	71572640	48.53

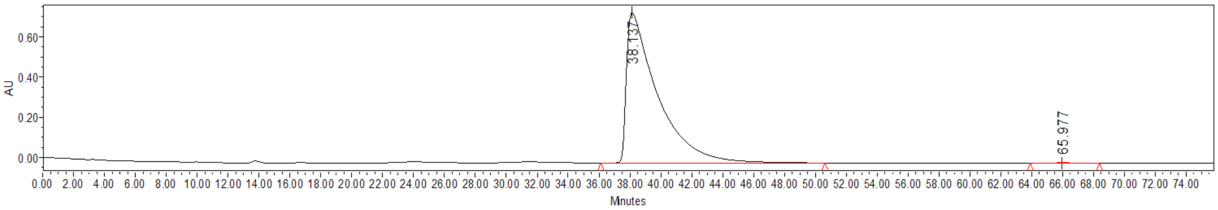

	Retention Time	Area	% Area
1	38.137	104540704	99.66
2	65.977	355522	0.34

(2*S*,3*R*)-3-(3-chlorobenzyl)-2-((*E*)-3-chlorostyryl)-1,5-bis(1-methyl-1H-imidazol-2-yl)pentane-1,5-dione

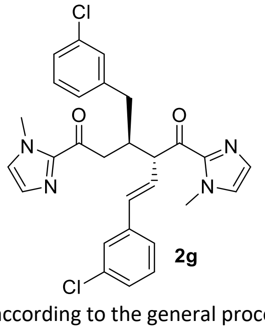

2g

Colourless foam. 68% yield (98% ee, 9:1 dr) was obtained according to the general procedure at 25 °C for 24 h.
HPLC (Chiral IA column), *i*-PrOH/*n*-Hexane = 20/80, Flow rate: 1.0 mL/min, 254 nm, t_r (minor) = 18.62 min, t_r (major) = 16.56 min.
^{1}H NMR (400 MHz, $CDCl_3$) δ 7.15 (d, *J* = 0.8 Hz, 1H), 7.13 – 7.03 (m, 8H), 7.02 (s, 2H), 6.79 (s, 1H), 6.45 (d, *J* = 16.0 Hz, 1H), 6.18 (dd, *J* = 16.0, 10.0 Hz, 1H), 4.78 (t, *J* = 9.2 Hz, 1H), 3.91 (s, 3H), 3.66 (s, 3H), 3.46 (dd, *J* = 16.4, 7.2 Hz, 1H), 3.42 – 3.32 (m, 1H), 2.91 (dd, *J* = 16.4, 5.2 Hz, 1H), 2.78 (dd, *J* = 13.6, 5.2 Hz, 1H), 2.59 (dd, *J* = 13.6, 8.8 Hz, 1H).
^{13}C NMR (100 MHz, $CDCl_3$) δ 192.0, 191.7, 143.2, 143.0, 142.0, 138.6, 134.3, 134.0, 133.0, 129.6, 129.5, 129.3, 129.0, 128.9, 128.8, 127.8, 127.8, 127.4, 126.9, 126.4, 126.1, 124.7, 56.4, 40.6, 39.8, 39.2, 36.3, 35.9.
HRMS (ESI-FT) calculated for $C_{28}H_{27}{}^{34.9689}Cl_2N_4O_2^+$ ($[M]+H^+$) = 521.1506, Found 521.1505.
HRMS (ESI-FT) calculated for $C_{28}H_{27}{}^{36.9659}Cl_2N_4O_2^+$ ($[M]+H^+$) = 523.1476, Found 523.1475.
$[\alpha]_D^{28}$ = +38.7 (*c* = 0.70, in CH_2Cl_2).
IR (film): 1667, 1593, 1471, 1401, 1285, 1155, 976, 883 cm^{-1}.

Chiral HPLC spectrum of **2g**:

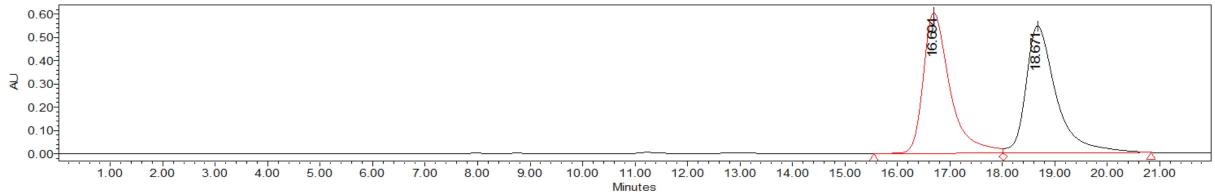

	Retention Time	Area	% Area
1	16.694	21414233	49.48
2	18.671	21865642	50.52

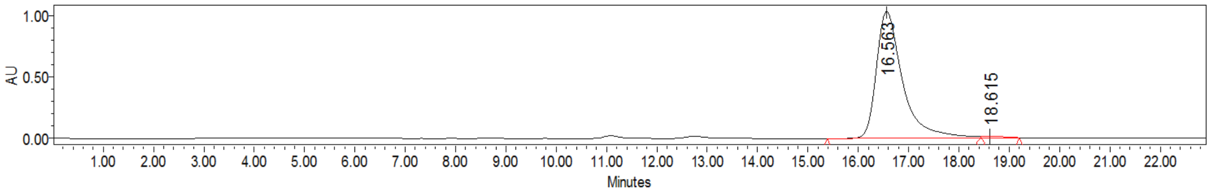

	Retention Time	Area	% Area
1	16.563	37398026	98.97
2	18.615	389831	1.03

(2*S*,3*R*)-3-(3-methoxybenzyl)-2-((*E*)-3-methoxystyryl)-1,5-bis(1-methyl-1H-imidazol-2-yl)pentane-1,5-dione

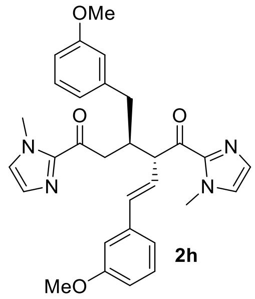

2h

Colourless foam. 65% yield (98% ee, 9:1 dr) was obtained according to the general procedure at 25 °C for 24 h.
UPC² (Chiral **OD-3**), CO_2/MeOH = 90/10, Flow rate: 1.0 mL/min, 254 nm, t_r (minor) = 22.27 min, t_r (major) = 20.31 min.
^{1}**H NMR** (400 MHz, $CDCl_3$) δ 7.14 (d, *J* = 0.8 Hz, 1H), 7.13 – 7.04 (m, 2H), 7.01 (s, 2H), 6.82 – 6.74 (m, 3H), 6.73 – 6.67(m, 3H), 6.64 (dd, *J* = 8.0, 2.4 Hz, 1H), 6.48 (d, *J* = 16.0 Hz, 1H), 6.20 (dd, *J* = 15.6, 10.0 Hz, 1H), 4.80 (t, *J* = 9.2 Hz, 1H), 3.93 (s, 3H), 3.76 (s, 3H), 3.71 (s, 3H), 3.61 (s, 3H), 3.49 – 3.37 (m, 2H), 3.02 – 2.91 (m, 1H), 2.82 (dd, *J* = 14.0, 5.2 Hz, 1H), 2.59 (dd, *J* = 13.6, 9.2 Hz, 1H).
^{13}**C NMR** (100 MHz, $CDCl_3$) δ 192.5, 192.0, 159.7, 159.5, 143.4, 143.2, 141.6, 138.3, 134.2, 129.4, 129.3, 129.1, 128. 7, 127.8, 127.6, 126.6, 122.0, 119.1, 114.5, 113.7, 112.3, 111.3, 56.5, 55.3, 55.2, 40.8, 40.3, 39.3, 36.4, 35.9.
HRMS (ESI-FT) calculated for $C_{30}H_{33}N_4O_4^+$ ([M]+H$^+$) = 513.2496, Found 513.2501.
$[\alpha]_D^{28}$ = +43.4 (*c* = 0.67, in CH_2Cl_2).
IR (film): 1670, 1599, 1404, 1261, 1156, 1044, 988, 915 cm^{-1}.
Chiral HPLC spectrum of **2h**:

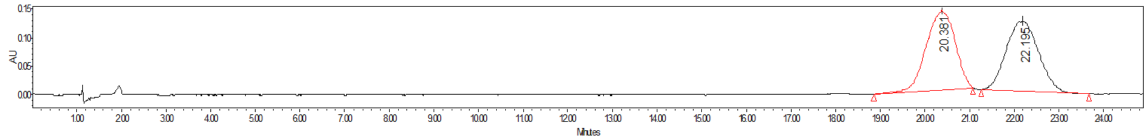

	Retention Time	Area	% Area
1	20.381	6266565	50.08
2	22.195	6246604	49.92

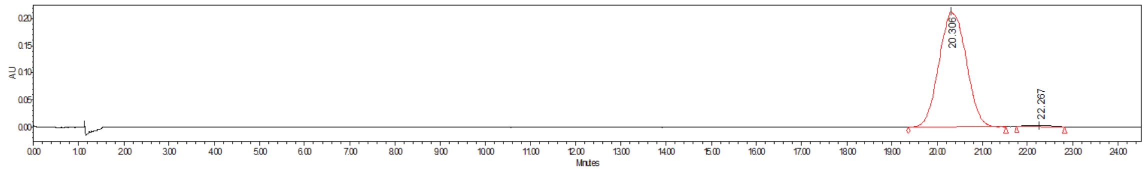

	Retention Time	Area	% Area
1	20.306	8936146	98.96
2	22.267	94180	1.04

(2*S*,3*R*)-1,5-bis(1-methyl-1H-imidazol-2-yl)-3-(3-methylbenzyl)-2-((*E*)-3-methylstyryl)pentane-1,5-dione

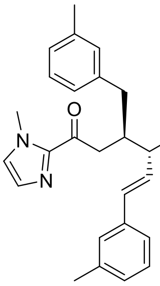

2i

Colourless foam. 80% yield (98% ee, 9:1 dr) was obtained according to the general procedure at 25 °C for 24 h.
HPLC (Chiral IA column), *i*-PrOH/*n*-Hexane = 30/70, Flow rate: 1.0 mL/min, 254 nm, t_r (minor) = 8.89 min, t_r (major) = 8.11 min.
^{1}H NMR (400 MHz, $CDCl_3$) δ 7.14 (s, 1H), 7.10 – 7.02 (m, 2H), 7.01 – 6.92 (m, 7H), 6.89 (d, *J* = 7.6 Hz, 1H), 6.73 (s, 1H), 6.48 (d, *J* = 16.0 Hz, 1H), 6.18 (dd, *J* = 16.0, 9.6 Hz, 1H), 4.78 (t, *J* = 9.2 Hz, 1H), 3.90 (s, 3H), 3.57 (s, 3H), 3.49 – 3.33 (m, 2H), 2.95 (dd, *J* = 16.4, 5.2 Hz, 1H), 2.80 (dd, *J* = 13.6, 4.8 Hz, 1H), 2.56 (dd, *J* = 13.6, 9.2 Hz, 1H), 2.26 (s, 3H), 2.20 (s, 3H).
^{13}C NMR (100 MHz, $CDCl_3$) δ 192.6, 192.0, 143.3, 143.2, 139.8, 137.8, 137.7, 136.7, 134.3, 130.3, 129.3, 128.6, 128.2, 128.2, 127.9, 127.5, 127.3, 126.9, 126.8, 126.6, 126.5, 123.6, 56.6, 40.6, 40.1, 39.5, 36.3, 35.8, 21.4, 21.3.
HRMS (ESI-FT) calculated for $C_{30}H_{33}N_4O_2^+$ ($[M]+H^+$) = 481.2598, Found 481.2593.
$[\alpha]^{27}_{D}$ = +36.7 (*c* = 0.85, in CH_2Cl_2).
IR (film): 1668, 1458, 1401, 1287, 977, 913 cm^{-1}.
Chiral HPLC spectrum of **2i**:

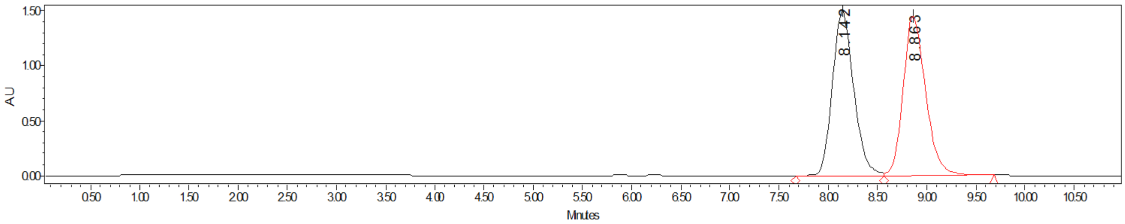

	Retention Time	Area	% Area
1	8.142	22595300	49.68
2	8.863	22883324	50.32

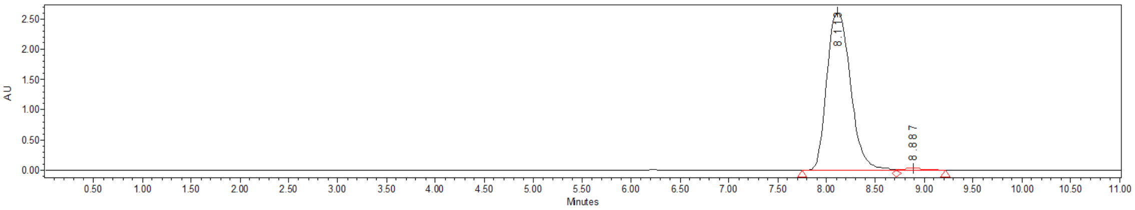

	Retention Time	Area	% Area
1	8.113	43808021	99.19
2	8.887	357632	0.81

(2*S*,3*R*)-1,5-bis(1-methyl-1H-imidazol-2-yl)-3-(2-methylbenzyl)-2-((*E*)-2-methylstyryl)pentane-1,5-dione

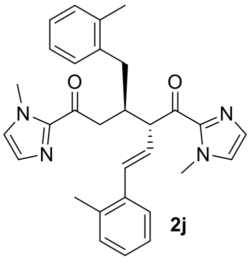

2j

Colourless foam. 81% yield (97% ee, 11:1 dr) was obtained according to the general procedure at 25 °C for 12 h.
HPLC (Chiral IA column), *i*-PrOH/*n*-Hexane = 30/70, Flow rate: 1.0 mL/min, 254 nm, t_r (minor) = 7.77 min, t_r (major) = 6.82 min.
^{1}H NMR (400 MHz, $CDCl_3$) δ 7.35 – 7.31 (m, 1H), 7.15 (d, *J* = 0.8 Hz, 1H), 7.07 – 6.94 (m, 8H), 6.87 (t, *J* = 7.6 Hz, 1H), 6.78 (d, *J* = 16.0 Hz, 1H), 6.74 (s, 1H), 6.11 (dd, *J* = 15.6, 10.0 Hz, 1H), 4.83 (t, *J* = 9.2, 1H), 3.91 (s, 3H), 3.55 (s, 3H), 3.52 – 3.39 (m, 2H), 3.02 (dd, *J* = 16.4, 5.2 Hz, 1H), 2.83 (dd, *J* = 13.6, 4.8 Hz, 1H), 2.64 (dd, *J* = 13.6, 9.6 Hz, 1H), 2.34 (s, 3H), 2.15 (s, 3H).
^{13}C NMR (100 MHz, $CDCl_3$) δ 192.4, 191.9, 143.3, 143.2, 137.9, 137.3, 135.7, 135.5, 132.0, 130.7, 130.2, 130.2, 129.2, 128.6, 128.6, 127.4, 127.4, 126.5, 126.3, 125.9, 125.4, 125.2, 57.3, 40.7, 38.2, 37.5, 36.4, 35.8, 19.7, 19.6.
HRMS (ESI-FT) calculated for $C_{30}H_{33}N_4O_2^+$ ($[M]+H^+$) = 481.2598, Found 481.2604.
$[\alpha]^{26}_{D}$ = +14.9 (*c* = 0.55, in CH_2Cl_2).
IR (film): 1670, 1462, 1404, 1287, 1155, 984, 916 cm^{-1}.

Chiral HPLC spectrum of **2j**:

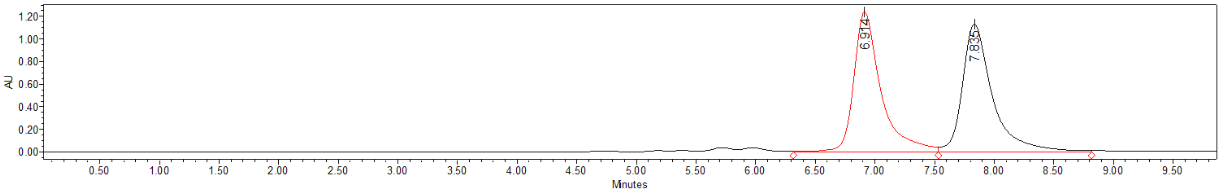

	Retention Time	Area	% Area
1	6.914	19725139	49.81
2	7.835	19873942	50.19

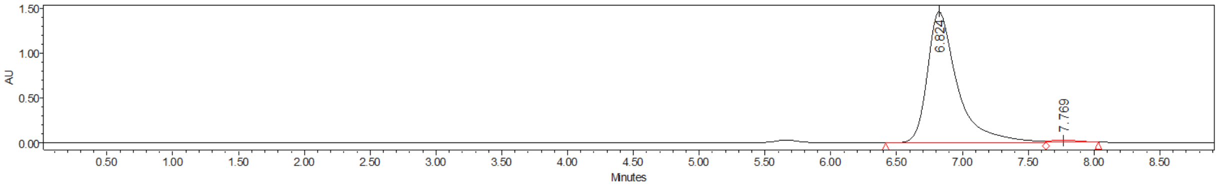

	Retention Time	Area	% Area
1	6.824	23066767	98.56
2	7.769	337643	1.44

(2*S*,3*R*)-1,5-bis(1-methyl-1H-imidazol-2-yl)-2-((*E*)-2-(thiophen-3-yl)vinyl)-3-(thiophen-3-ylmethyl)pentane-1,5-dione

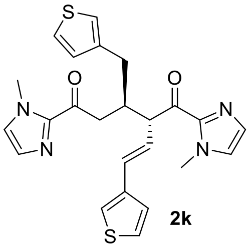

2k

Colourless foam. 60% yield (98% ee, 9:1 dr) was obtained according to the general procedure at 25 °C for 24 h.
UPC[2] (Chiral **OD-3**), CO_2/MeOH = 80/20, Flow rate: 1.0 mL/min, 254 nm, t_r (minor) = 6.74 min, t_r (major) = 7.43 min.
^{1}**H NMR** (400 MHz, $CDCl_3$) δ 7.17 – 7.11 (m, 3H), 7.06 (dd, *J* = 5.2, 1.2 Hz, 1H), 7.03 – 6.98 (m, 2H), 6.96 – 6.90 (m, 2H), 6.86 (d, *J* = 1.6 Hz, 1H), 6.80 (s, 1H), 6.51 (d, *J* = 15.6 Hz, 1H), 6.01 (dd, *J* = 16.0, 10.0 Hz, 1H), 4.73 (t, *J* = 9.2 Hz, 1H), 3.93 (s, 3H), 3.68 (s, 3H), 3.46 – 3.31 (m, 2H), 2.98 (dd, *J* = 16.0, 5.2 Hz, 1H), 2.79 (dd, *J* = 14.4, 5.2 Hz, 1H), 2.67 (dd, *J* = 14.4, 8.4 Hz, 1H).
^{13}**C NMR** (100 MHz, $CDCl_3$) δ 192.5, 192.0, 143.4, 143.2, 140.2, 139.5, 129.4, 129.0, 128.7, 128.6, 127.6, 127.2, 126.6, 125.7, 125.1, 125.0, 122.1, 122.0, 56.3, 41.0, 38.7, 36.4, 36.0, 34.6.
HRMS (ESI-FT) calculated for $C_{24}H_{25}N_4O_2S_2^+$ ([M]+H$^+$) = 465.1413, Found 465.1418.
$[\alpha]_D^{28}$ = +54.6 (*c* = 0.56, in CH_2Cl_2).
IR (film): 1665, 1399, 1287, 1154, 1080, 972, 915 cm^{-1}.
Chiral HPLC spectrum of **2k**:

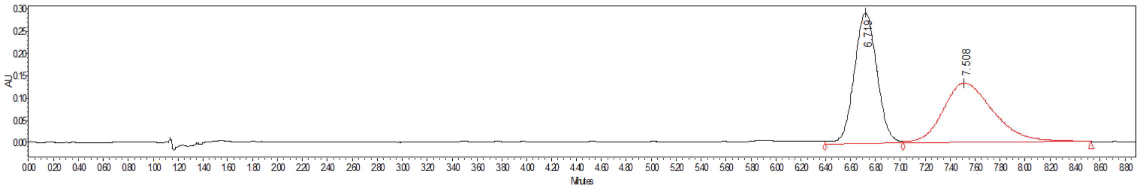

	Retention Time	Area	% Area
1	6.719	3760575	49.57
2	7.508	3825907	50.43

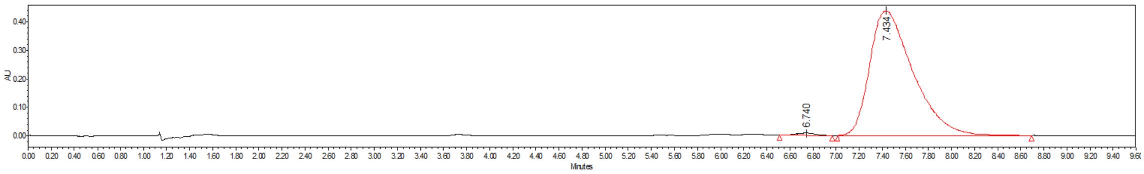

	Retention Time	Area	% Area
1	6.740	90822	0.79
2	7.434	11379294	99.21

(2*S*,3*R*)-1,5-bis(1-methyl-1H-imidazol-2-yl)-3-((1-methyl-1H-indol-5-yl)methyl)-2-((*E*)-2-(1-methyl-1H-indol-5-yl)vinyl)pentane-1,5-dione

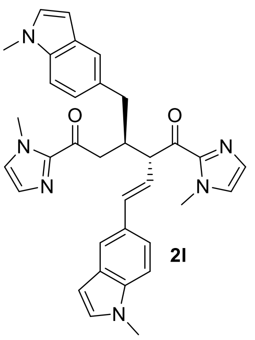

2l

Colourless foam. 72% yield (>99% ee, 10:1 dr) was obtained according to the general procedure at 25 °C for 36 h.
UPC[2] (Chiral **IC-3**), CO_2/MeOH = 70/30, Flow rate: 1.0 mL/min, 254 nm, t_r (minor) = 33.53 min, t_r (major) = 28.61 min.
^{1}H NMR (400 MHz, $CDCl_3$) δ 7.35 (s, 1H), 7.31 (s, 1H), 7.18 – 7.11 (m, 4H), 7.09 (dd, *J* = 8.4, 1.2 Hz, 1H), 6.99 – 6.95 (m, 3H), 6.93 (d, *J* = 2.8 Hz, 1H), 6.63 (d, *J* = 15.6 Hz, 2H), 6.37 (d, *J* = 2.8 Hz, 1H), 6.28 (d, *J* = 2.8 Hz, 1H), 6.13 (dd, *J* = 16.0, 10.0 Hz, 1H), 4.83 (t, *J* = 9.2 Hz, 1H), 3.85 (s, 3H), 3.72 (d, *J* = 5.6 Hz, 6H), 3.57 – 3.41 (m, 2H), 3.37 (s, 3H), 3.05 – 2.92 (m, 2H), 2.69 (dd, *J* = 13.6, 9.6 Hz, 1H).
^{13}C NMR (100 MHz, $CDCl_3$) δ 193.2, 192.4, 143.5, 143.5, 136.5, 135.6, 135.3, 130.9, 129.2, 128.7, 128.6, 128.5, 128.5, 128.4, 128.4, 127.4, 126.4, 124.8, 123.7, 121.6, 120.3, 119.4, 109.0, 108.9, 101.3, 100.9, 57.0, 40.9, 40.6, 40.5, 36.2, 35.6, 33.0, 32.9.
HRMS (ESI-FT) calculated for $C_{34}H_{35}N_6O_2^+$ ($[M]+H^+$) = 559.2816, Found 559.2819.
$[\alpha]_D^{26}$ = +50.4 (*c* = 0.81, in CH_2Cl_2).
IR (film): 1665, 1401, 1242, 1153, 1079, 973, 915 cm^{-1}.
Chiral HPLC spectrum of **2l**:

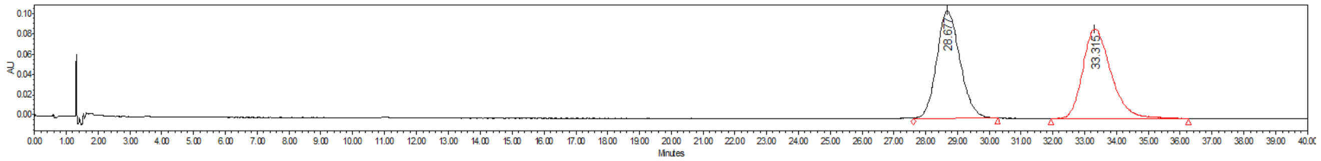

	Retention Time	Area	% Area
1	28.677	5402528	49.78
2	33.315	5450526	50.22

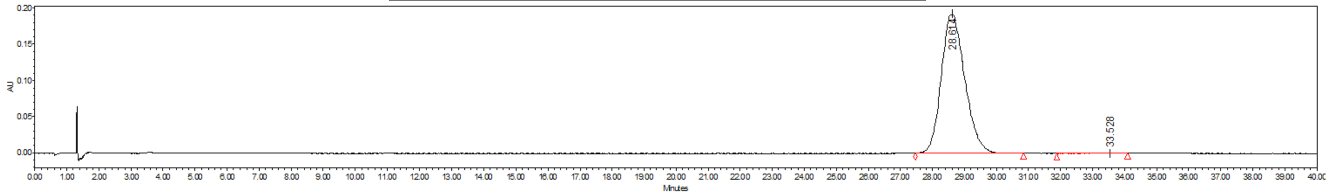

	Retention Time	Area	% Area
1	28.614	9913995	99.80
2	33.528	19815	0.20

(2*S*,3*R*)-1,5-bis(1-methyl-1H-imidazol-2-yl)-2-((*E*)-2-(naphthalen-2-yl)vinyl)-3-(naphthalen-2-ylmethyl)pentane-1,5-dione

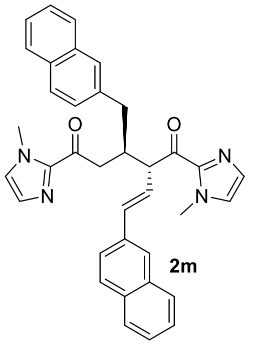

2m

Colourless foam. 81% yield (>99% ee, 12:1 dr) was obtained according to the general procedure at 25 °C for 24 h.
UPC[2] (Chiral **IA-3**), CO_2/MeOH = 75/25, Flow rate: 1.0 mL/min, 254 nm, t_r (minor) = 21.60 min, t_r (major) = 20.02 min.
^{1}H NMR (400 MHz, $CDCl_3$) δ 7.77 – 7.65 (m, 5H), 7.60 – 7.56 (m, 1H), 7.50 (d, *J* = 9.2 Hz, 2H), 7.47 – 7.32 (m, 6H), 7.18 (d, *J* = 0.8 Hz, 1H), 6.99 (d, *J* = 0.8 Hz, 1H), 6.93 (s, 1H), 6.72 (d, *J* = 16.0 Hz, 1H), 6.55 (s, 1H), 6.34 (dd, *J* = 15.6, 9.6 Hz, 1H), 4.93 (t, *J* = 9.6 Hz, 1H), 3.75 (s, 3H), 3.63 – 3.45 (m, 2H), 3.32 (s, 3H), 3.12 (dd, *J* = 16.4, 5.6 Hz, 1H), 3.02 (dd, *J* = 13.6, 5.6 Hz, 1H), 2.82 (dd, *J* = 13.6, 8.8 Hz, 1H).
^{13}C NMR (100 MHz, $CDCl_3$) δ 192.4, 191.8, 143.3, 143.3, 137.6, 134.4, 134.3, 133.5, 133.4, 133.0, 132.3, 129.4, 128.6, 128.6, 128.2, 128.2, 128.1, 128.1, 127.9, 127.7, 127.7, 127.7, 127.4, 126.6, 126.2, 125.8, 125.7, 125.3, 123.7, 56.9, 41.1, 40.9, 39.7, 36.1, 35.5.
HRMS (ESI-FT) calculated for $C_{36}H_{33}N_4O_2^+$ ($[M]+H^+$) = 553.2598, Found 553.2594.
$[\alpha]_D^{29}$ = +83.4 (*c* = 0.89, in CH_2Cl_2).
IR (film): 1669, 1404, 1267, 1154, 977, 912 cm^{-1}.

Chiral HPLC spectrum of **2m**:

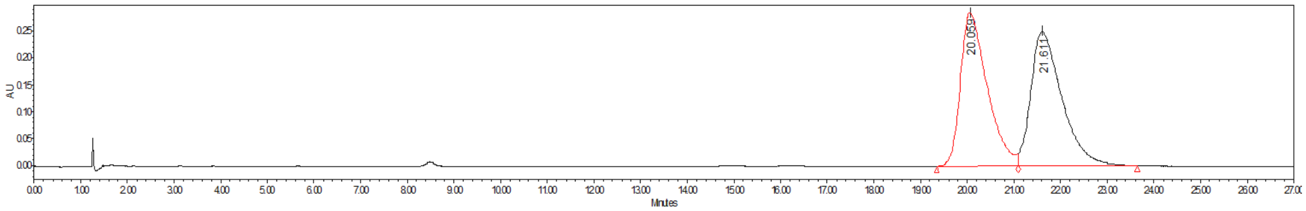

	Retention Time	Area	% Area
1	20.059	11317570	49.78
2	21.611	11416833	50.22

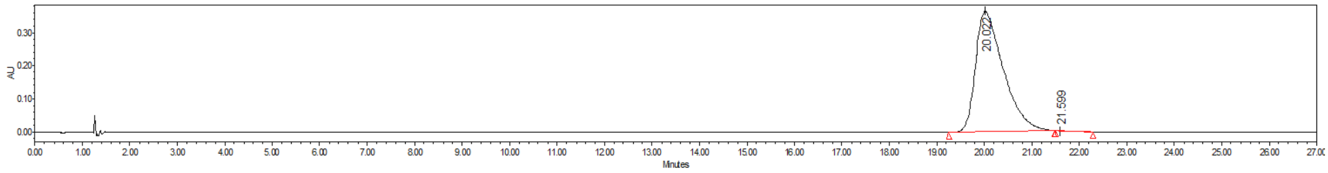

	Retention Time	Area	% Area
1	20.022	14828229	99.95
2	21.599	6805	0.05

(*E*)-1,5-bis(1-methyl-1H-imidazol-2-yl)-3-phenethyl-2-(3-phenylprop-1-en-1-yl)pentane-1,5-dione

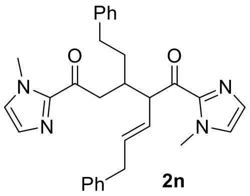

2n

Colourless foam. 80% yield (>99% ee, 9:1 dr) was obtained according to the general procedure at 25 °C for 36 h.
HPLC (Chiral IA column), *i*-PrOH/*n*-Hexane = 30/70, Flow rate: 1.0 mL/min, 254 nm, t_r (minor) = 7.77 min, t_r (major) = 6.82 min.
^{1}H NMR (400 MHz, $CDCl_3$) δ 7.22 – 7.16 (m, 4H), 7.16 – 7.10 (m, 4H), 7.09 – 7.00 (m, 5H), 6.98 (s, 1H), 5.86 (dt, *J* = 15.6, 6.8 Hz, 1H), 5.72 (dd, *J* = 15.2, 9.6 Hz, 1H), 4.79 (dd, *J* = 9.6, 7.2 Hz, 1H), 3.95 (d, *J* = 13.2 Hz, 6H), 3.43 (dd, *J* = 17.6, 5.2 Hz, 1H), 3.32 (d, *J* = 6.8 Hz, 2H), 3.21 (dd, *J* = 17.6, 7.2 Hz, 1H), 2.98 – 2.87 (m, 1H), 2.73 – 2.62 (m, 2H), 1.81 – 1.61 (m, 2H).
^{13}C NMR (100 MHz, $CDCl_3$) δ 193.6, 192.2, 143.3, 143.1, 142.6, 140.3, 134.4, 129.2, 129.0, 128.5, 128.5, 128.4, 128.3, 127.7, 127.4, 126.9, 126.0, 125.7, 54.0, 40.5, 39.2, 36.4, 36.3, 36.2, 35.4, 33.4.
HRMS (ESI-FT) calculated for $C_{30}H_{33}N_4O_2^+$ ($[M]+H^+$) = 481.2598, Found 481.2606.
$[\alpha]_D^{25}$ = +33.4 (*c* = 0.61, in CH_2Cl_2).
IR (film): 1670, 1456, 1403, 1286, 1154, 979, 914 cm^{-1}.
Chiral HPLC spectrum of **2n**:

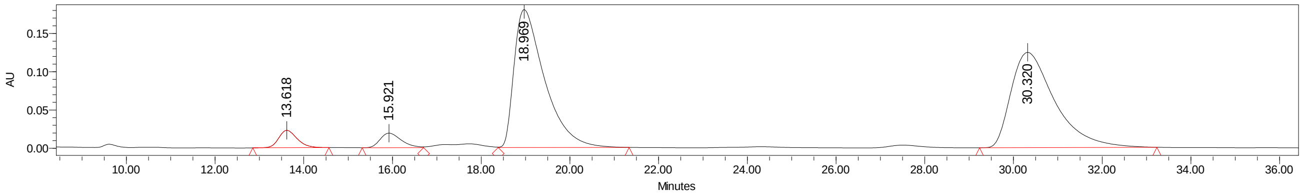

	Retention Time	Area	% Area
1	13.618	650129	3.53
2	15.921	628049	3.41
3	18.969	8596207	46.73
4	30.320	8520988	46.32

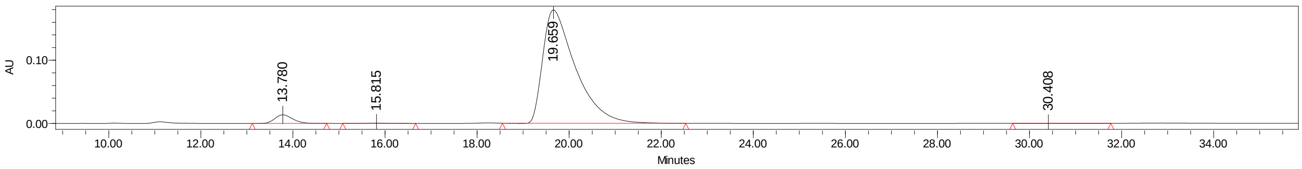

	Retention Time	Area	% Area
1	13.780	365325	3.98
2	15.815	14420	0.16
3	19.659	8805307	95.83
4	30.408	3220	0.04

(*E*)-1,5-Bis(1-methyl-1H-imidazol-2-yl)-2-(4-phenylbut-1-en-1-yl)-3-(3-phenylpropyl)pentane-1,5-dione

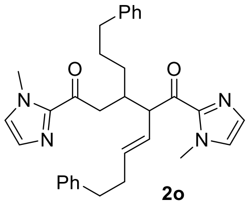

2o

Colourless foam. 84% yield (99% ee, 9:1 dr) was obtained according to the general procedure at 25 °C for 36 h.
HPLC (Chiral IH column), *i*-PrOH/*n*-Hexane = 10/90, Flow rate: 1.0 mL/min, 254 nm, t_r (minor) = 19.20 min, t_r (major) = 20.43 min.
^{1}H NMR (400 MHz, $CDCl_3$) δ 7.21 – 7.16 (m, 4H), 7.14 (d, *J* = 0.4 Hz, 1H), 7.13 – 7.09 (m, 3H), 7.08 – 7.04 (m, 4H), 7.01 (s, 1H), 6.98 (s, 1H), 5.66 (dt, *J* = 15.2, 6.8 Hz, 1H), 5.51 (dd, *J* = 15.2, 9.6 Hz, 1H), 4.60 (dd, *J* = 9.6, 7.6 Hz, 1H), 3.94 (s, 3H), 3.92 (s, 3H), 3.26 (dd, *J* = 17.2, 5.2 Hz, 1H), 3.05 (dd, *J* = 17.2, 7.2 Hz, 1H), 2.84 (dd, *J* = 12.8, 6.0 Hz, 1H), 2.52 (dd, *J* = 14.8, 7.2 Hz, 4H), 2.27 – 2.19 (m, 2H), 1.70 – 1.61 (m, 2H), 1.44 – 1.28 (m, 2H).
^{13}C NMR (100 MHz, $CDCl_3$) δ 193.8, 192.5, 143.4, 143.1, 142.6, 141.9, 135.1, 129.1, 128.9, 128.5, 128.5, 128.3, 128.1, 127.3, 127.0, 126.8, 125.7, 125.5, 54.3, 40.5, 36.3, 36.3, 35.9, 35.9, 35.6, 34.5, 32.7, 28.4.
HRMS (ESI-FT) calculated for $C_{32}H_{37}N_4O_2^+$ ([M]+H$^+$) = 509.2911, Found 509.2914.
$[\alpha]^{22}_{\lambda}$ = +77.1 (*c* = 0.81, in CH_2Cl_2, λ = 436 nm).
IR (film): 1668, 1455, 1402, 1285, 1154, 976, 914 cm^{-1}.
Chiral HPLC spectrum of **2o**:

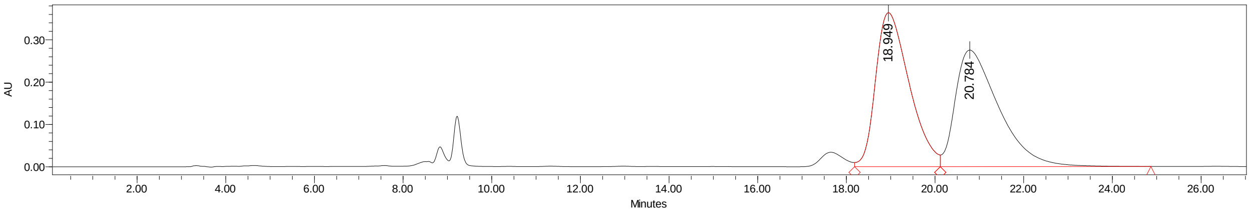

	Retention Time	Area	% Area
1	18.949	19183951	50.58
2	20.784	18743157	49.42

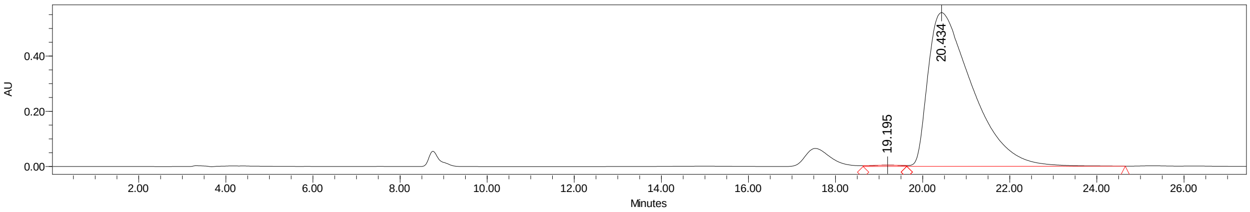

	Retention Time	Area	% Area
1	19.195	229632	0.58
2	20.434	39579752	99.42

(*E*)-3-Butyl-1,5-bis(1-methyl-1H-imidazol-2-yl)-2-(pent-1-en-1-yl)pentane-1,5-dione

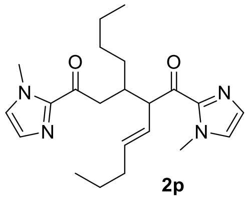

2p

Colourless foam. 63% yield (96% ee, 14:1 dr) was obtained according to the general procedure at 25 °C for 36 h.
HPLC (Chiral IE column), *i*-PrOH/*n*-Hexane = 20/80, Flow rate: 1.0 mL/min, 254 nm, t_r (minor) = 16.89 min, t_r (major) = 13.82 min.
^{1}H NMR (400 MHz, $CDCl_3$) δ 7.09 (d, *J* = 12.4 Hz, 2H), 6.98 (d, *J* = 9.6 Hz, 2H), 5.62 (dt, *J* = 15.6, 6.4 Hz, 1H), 5.49 (dd, *J* = 15.2, 9.6 Hz, 1H), 4.57 (dd, *J* = 9.6, 7.2 Hz, 1H), 3.96 (s, 6H), 3.32 (dd, *J* = 17.6, 5.6 Hz, 1H), 3.06 (dd, *J* = 17.6, 7.2 Hz, 1H), 2.84 – 2.70 (m, 1H), 1.91 – 1.84 (m, 2H), 1.41 – 1.15 (m, 8H), 0.81 – 0.72 (m, 6H).
^{13}C NMR (100 MHz, $CDCl_3$) δ 194.1, 192.7, 143.4, 143.2, 135.9, 129.1, 128.9, 127.2, 126.7, 126.3, 54.5, 40.6, 36.3, 36.3, 36.1, 34.8, 33.1, 29.1, 22.9, 22.4, 14.1, 13.7.
HRMS (ESI-FT) calculated for $C_{22}H_{33}N_4O_2^+$ ([M]+H$^+$) = 385.2598, Found 385.2606.
$[\alpha]^{26}_{D}$ = +33.3 (*c* = 0.48, in CH_2Cl_2).
IR (film): 1671, 1463, 1404, 1285, 1154, 978, 913 cm^{-1}.
Chiral HPLC spectrum of **2p**:

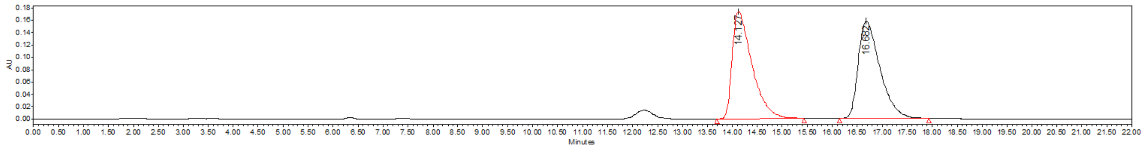

	Retention Time	Area	% Area
1	14.127	4798162	50.10
2	16.682	4778417	49.90

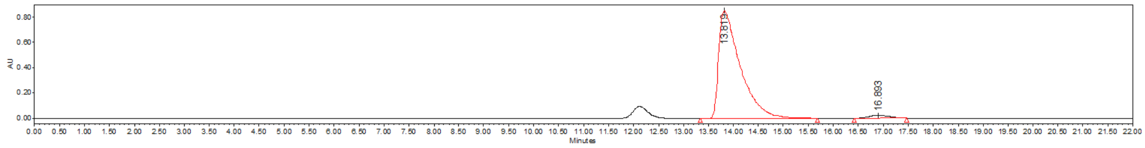

	Retention Time	Area	% Area
1	13.819	25301515	97.94
2	16.893	530910	2.06

(*E*)-3-isobutyl-1,5-bis(1-methyl-1H-imidazol-2-yl)-2-(3-methylbut-1-en-1-yl)pentane-1,5-dione

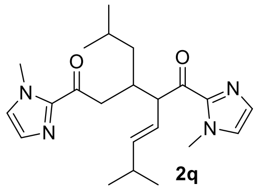

2q

Colourless foam. 76% yield (92% ee, >19:1 dr) was obtained according to the general procedure at 25 °C for 36 h.
HPLC (Chiral IE column), *i*-PrOH/*n*-Hexane = 20/80, Flow rate: 1.0 mL/min, 254 nm, t_r (minor) = 16.21 min, t_r (major) = 10.78 min.
^{1}H NMR (400 MHz, $CDCl_3$) δ 7.08 (d, *J* = 11.2 Hz, 2H), 6.98 (d, *J* = 11.2 Hz, 2H), 5.57 (dd, *J* = 15.6, 6.4 Hz, 1H), 5.50 – 5.41 (m, 1H), 4.54 (dd, *J* = 9.2, 6.4 Hz, 1H), 3.95 (d, *J* = 0.8 Hz, 6H), 3.34 (dd, *J* = 17.6, 6.0 Hz, 1H), 2.97 (dd, *J* = 18.0, 6.0 Hz, 1H), 2.89 – 2.79 (m, 1H), 2.22 – 2.09 (m, 1H), 1.71 – 1.57 (m, 1H), 1.21 (t, *J* = 7.2 Hz, 2H), 0.88 – 0.80 (m, 12H).
^{13}C NMR (100 MHz, $CDCl_3$) δ 194.3, 192.5, 143.4, 143.3, 142.8, 129.1, 128.9, 127.2, 126.7, 122.9, 54.4, 42.9, 40.7, 36.3, 36.3, 34.1, 31.2, 25.5, 23.1, 22.5, 22.4, 22.2.
HRMS (ESI-FT) calculated for $C_{22}H_{33}N_4O_2^+$ ([M]+H$^+$) = 385.2598, Found 385.2600.
$[\alpha]_D^{26}$ = +21.2 (*c* = 0.58, in CH_2Cl_2).
IR (film): 1671, 1465, 1401, 1287, 1154, 980, 916 cm^{-1}.
Chiral HPLC spectrum of **2q**:

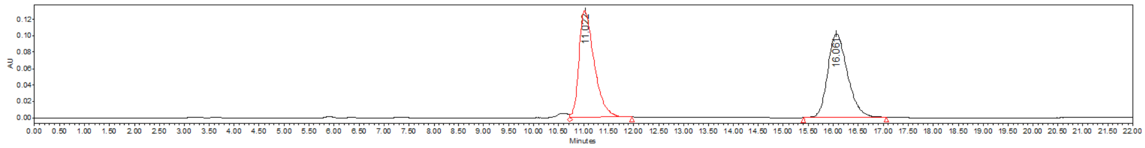

	Retention Time	Area	% Area
1	11.022	2781965	49.76
2	16.061	2808926	50.24

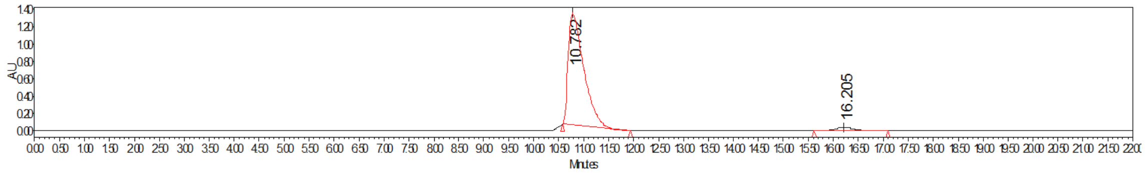

	Retention Time	Area	% Area
1	10.782	27926398	96.32
2	16.205	1068304	3.68

(*E*)-1,5-Bis(1-methyl-1H-imidazol-2-yl)-3-((13-methyl-6,7,8,9,11,12,13,14,15,16-decahydrospiro[cyclopenta[a]phenanthrene-17,2'-[1,3]dioxolan]-3-yl)methyl)-2-(2-(13-methyl-6,7,8,9,11,12,13,14,15,16-decahydrospiro[cyclopenta[a]phenanthrene-17,2'-[1,3]dioxolan]-3-yl)vinyl)pentane-1,5-dione

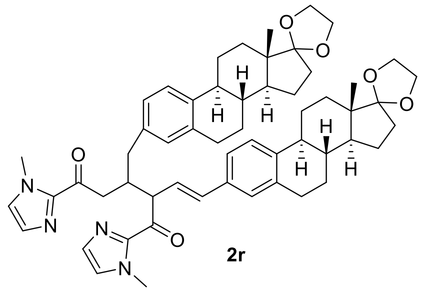

2r

Colourless foam. 69% yield (2.8:1 *E*/*Z*; 99% ee and >19:1 dr for *E*-isomer) was obtained according to the general procedure at 25 °C for 6 h.
HPLC (Chiral IH column), *i*-PrOH/*n*-Hexane = 30/70, Flow rate: 1.0 mL/min, 254 nm, t_r (minor) = 29.31 min, t_r (major) = 36.26 min.
^{1}H NMR (400 MHz, $CDCl_3$) δ 7.16 – 7.10 (m, 2H), 7.06 (d, *J* = 8.0 Hz, 1H), 7.02 (d, *J* = 0.4 Hz, 1H), 7.00 – 6.96 (m, 2H), 6.95 – 6.88 (m, 2H), 6.81 (s, 1H), 6.77 (s, 1H), 6.47 (d, *J* = 15.6 Hz, 1H), 6.15 (dd, *J* = 15.6, 10.0 Hz, 1H), 4.76 (t, *J* = 8.8, 1H), 3.98 – 3.86 (m, 11H), 3.63 (s, 3H), 3.45 – 3.34 (m,

2H), 3.02 (dd, J = 18.8, 8.0 Hz, 1H), 2.81 – 2.71 (m, 3H), 2.70 – 2.61 (m, 2H), 2.54 (dd, J = 13.6, 8.8 Hz, 1H), 2.34 – 2.12 (m, 4H), 2.05 – 1.97 (m, 2H), 1.90 – 1.70 (m, 9H), 1.66 – 1.57 (m, 2H), 1.56 – 1.49 (m, 2H), 1.44 – 1.22 (m, 8H), 0.86 (s, 6H).
^{13}C NMR (100 MHz, $CDCl_3$) δ 192.7, 192.2, 143.4, 143.2, 140.0, 137.9, 136.9, 136.7, 136.3, 134.3, 134.2, 130.3, 129.2, 128.6, 127.3, 127.0, 126.8, 126.6, 126.5, 125.4, 124.9, 123.8, 119.5, 119.5, 65.4, 64.7, 56.3, 49.6, 49.5, 46.2, 46.2, 44.2, 44.1, 40.7, 39.7, 39.2, 39.0, 36.3, 36.0, 34.3, 30.9, 30.8, 29.6, 29.4, 27.1, 27.0, 26.1, 26.0, 22.5, 14.4.
HRMS (ESI-FT) calculated for $C_{56}H_{69}N_4O_6^+$ ([M]+H$^+$) = 893.5212, Found 893.5211.
$[\alpha]_D^{26}$ = +47.7 (c = 1.24, in CH_2Cl_2).
IR (film): 1670, 1403, 1282, 1157,1104, 1040, 968 cm^{-1}.
Chiral HPLC spectrum of **2r**:

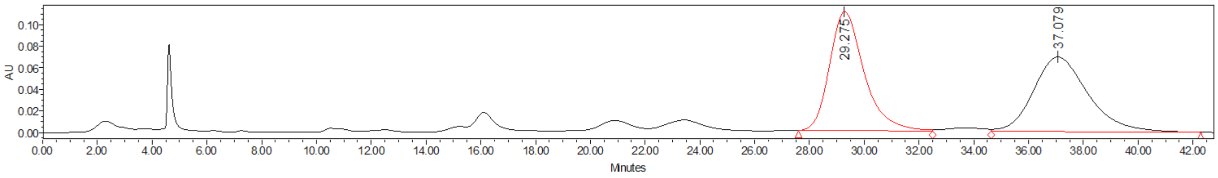

	Retention Time	Area	% Area
1	29.275	9667580	50.26
2	37.079	9568291	49.74

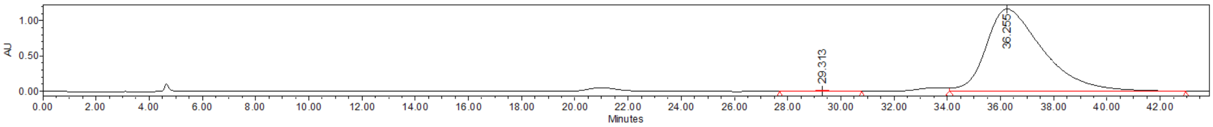

	Retention Time	Area	% Area
1	29.313	813257	0.46
2	36.255	174318935	99.54

(*E*)-3-Methyl-1,5-bis(1-methyl-1H-imidazol-2-yl)-2-styrylpentane-1,5-dione

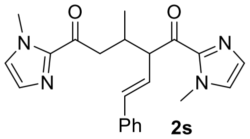

2s

Colourless foam. 63% yield (98% ee, 6:1 dr) was obtained according to the general procedure at 25 °C for 24 h.
UPC^2 (Chiral **AD-3**), CO_2/MeOH = 90/10, Flow rate: 1.0 mL/min, 254 nm, t_r (minor) = 9.08 min, t_r (major) = 10.35 min for major isomer.
^{1}H NMR (400 MHz, $CDCl_3$) δ 7.28 – 7.19 (m, 4H), 7.18 – 7.13 (m, 2H), 7.07 (s, 1H), 7.02 (s, 1H), 6.88 (s, 1H), 6.56 (d, J = 16.0 Hz, 1H), 6.24 (dd, J = 15.6, 9.6 Hz, 1H), 4.65 (t, J = 9.2 Hz, 1H), 4.00 (s, 3H), 3.81 (s, 3H), 3.49 – 3.38 (m, 1H), 3.06 – 2.94 (m, 2H), 1.03 (d, J = 6.4 Hz, 3H).
^{13}C NMR (100 MHz, $CDCl_3$) δ 193.0, 192.4, 143.5, 143.3, 136.9, 134.3, 129.4, 128.9, 128.4, 127.6, 127.5, 126.8, 126.5, 57.4, 43.6, 36.4, 36.1, 32.7, 19.2.
HRMS (ESI-FT) calculated for $C_{22}H_{25}N_4O_2^+$ ([M]+H$^+$) = 377.1972, Found 377.1964.
$[\alpha]_D^{24}$ = +53.3 (c = 0.4, in CH_2Cl_2).
IR (film): 1670, 1403, 1288, 1155, 997, cm^{-1}.
Chiral HPLC spectrum of **2s**:

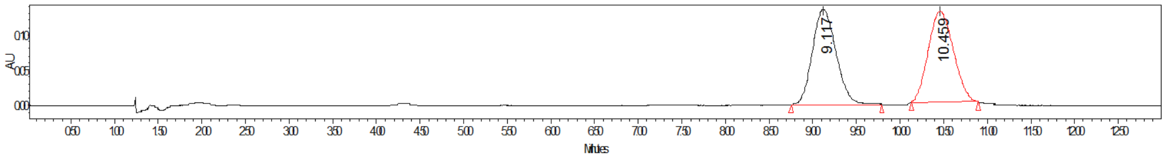

	Retention Time	Area	% Area
1	9.117	2529630	49.41
2	10.459	2590322	50.59

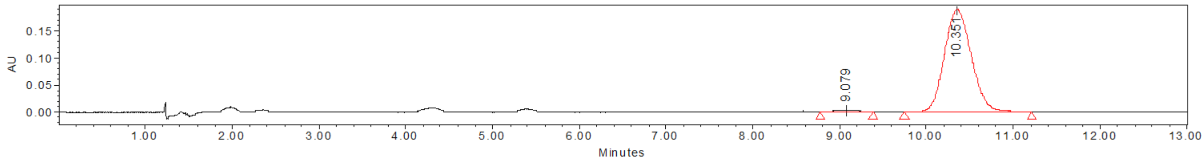

	Retention Time	Area	% Area
1	9.079	37253	0.91
2	10.351	4039645	99.09

(*E*)-1,5-bis(1-Methyl-1H-imidazol-2-yl)-3-phenethyl-2-styrylpentane-1,5-dione

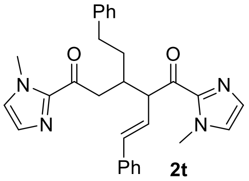

2t

Colourless foam. 60% yield (>99% ee, 6:1 dr) was obtained according to the general procedure at 25 °C for 24 h.

UPC² (Chiral **AD-3**), CO_2/MeOH = 90/10, Flow rate: 1.0 mL/min, 254 nm, t_r (minor) = 20.34 min, t_r (major) = 22.11 min for major isomer.

¹H NMR (400 MHz, $CDCl_3$) δ 7.23 – 7.17 (m, 6H), 7.17 – 7.09 (m, 3H), 7.09 – 7.03 (m, 3H), 7.02 (s, 1H), 6.83 (s, 1H), 6.54 (d, *J* = 16.0 Hz, 1H), 6.22 (dd, *J* = 16.0, 10.0 Hz, 1H), 4.94 – 4.83 (t, *J* = 8.8, 1H), 3.98 (s, 3H), 3.74 (s, 3H), 3.62 – 3.52 (m, 1H), 3.18 – 3.04 (m, 2H), 2.80 – 2.62 (m, 2H), 1.83 – 1.63 (m, 2H).

¹³C NMR (100 MHz, $CDCl_3$) δ 192.9, 192.2, 143.4, 143.1, 142.6, 136.9, 134.3, 129.4, 128.9, 128.5, 128.4, 128.3, 127.6, 127.5, 127.4, 126.8, 126.5, 125.7, 55.6, 40.9, 37.3, 36.4, 36.0, 35.5, 33.3.

HRMS (ESI-FT) calculated for $C_{29}H_{31}N_4O_2^+$ ([M]+H⁺) = 467.2442, Found 467.2433.

$[\alpha]_D^{23}$ = +47.9 (*c* = 0.52, in CH_2Cl_2).

IR (film): 1670, 1403, 1288, 1155, 997, cm^{-1}.

Chiral HPLC spectrum of **2t**:

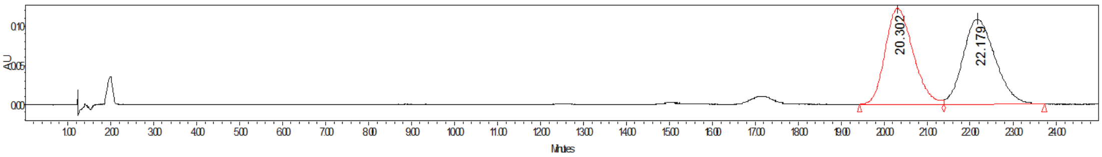

	Retention Time	Area	% Area
1	20.302	5526832	49.50
2	22.179	5638766	50.50

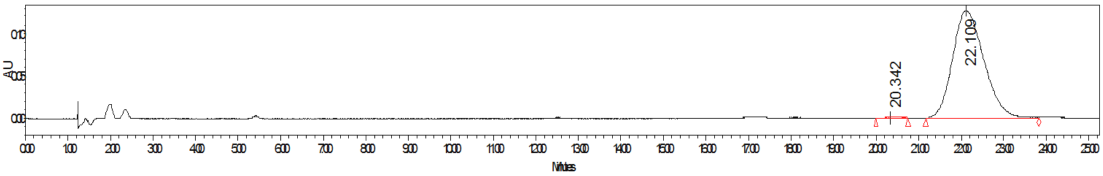

	Retention Time	Area	% Area
1	20.342	23715	0.36
2	22.109	6483396	99.64

(*E*)-1-(1-Methyl-1H-imidazol-2-yl)-2-styrylheptane-1,5-dione

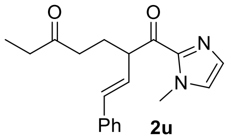

2u

Colourless oil. 71% yield (92% ee) was obtained according to the general procedure at 25 °C for 3 h.

HPLC (Chiral **IC** column), *i*-PrOH/Hexane = 30/70, Flow rate: 1.0 mL/min, 254 nm, t_r (minor) = 10.66 min, t_r (major) = 16.28 min.

¹H NMR (400 MHz, $CDCl_3$) δ 7.35 (d, *J* = 7.2 Hz, 2H), 7.31 – 7.25 (m, 2H), 7.20 (t, *J* = 7.2 Hz, 1H), 7.16 (s, 1H), 7.04 (s, 1H), 6.59 (d, *J* = 15.6 Hz, 1H), 6.27 (dd, *J* = 15.6, 8.8 Hz, 1H), 4.64 (dd, *J* = 15.2, 8.4 Hz, 1H), 3.99 (s, 3H), 2.57 – 2.44 (m, 2H), 2.44 – 2.32 (m, 2H), 2.23 – 2.13 (m, 1H), 2.13 – 1.97 (m, 1H), 1.01 (t, *J* = 7.2 Hz, 3H).

¹³C NMR (100 MHz, $CDCl_3$) δ 210.9, 192.7, 142.7, 137.0, 133.4, 129. 5, 128.6, 127.7, 127.67, 127.7, 126.5, 50.4, 40.0, 36.4, 36.1, 26.1, 7.9.

HRMS (ESI-FT) calculated for $C_{19}H_{23}N_2O_2^+$ ([M]+H⁺) = 311.1754, Found 311.1745.

$[\alpha]_D^{23}$ = +15.7 (*c* = 0.44, in CH_2Cl_2).

IR (film): 1711, 1671, 1449, 1403, 1288, 1155, 971 cm^{-1}.

Chiral HPLC spectrum of **2u**:

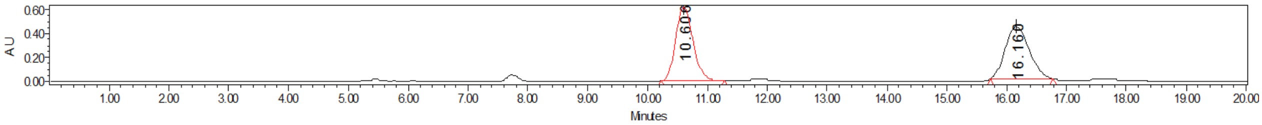

	Retention Time	Area	% Area
1	10.606	12220529	50.20
2	16.160	12125439	49.80

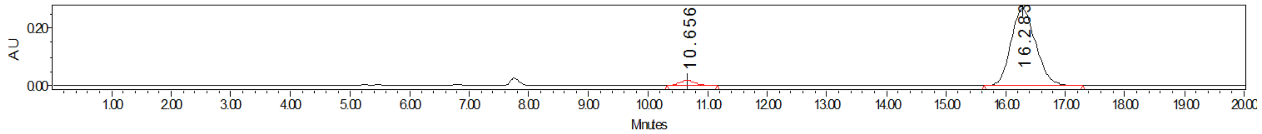

	Retention Time	Area	% Area
1	10.656	328512	4.09
2	16.283	7702419	95.91

(*E*)-2-(4-Fluorostyryl)-1-(1-methyl-1H-imidazol-2-yl)heptane-1,5-dione

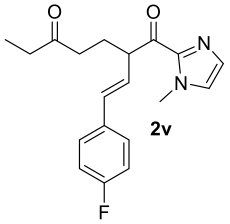

2v

Colourless oil. 68% yield (92% ee) was obtained according to the general procedure at 25 °C for 3 h.
HPLC (Chiral **IA** column), *i*-PrOH/Hexane = 20/80, Flow rate: 1.0 mL/min, 254 nm, t_r (minor) = 10.11 min, t_r (major) = 11.23 min.
^{1}H NMR (400 MHz, $CDCl_3$) δ 7.31 (dd, *J* = 8.8, 5.6 Hz, 2H), 7.16 (s, 1H), 7.04 (s, 1H), 6.96 (t, *J* = 8.4 Hz, 2H), 6.55 (d, *J* = 16.0 Hz, 1H), 6.18 (dd, *J* = 16.0, 9.2 Hz, 1H), 4.62 (dd, *J* = 15.2, 8.0 Hz, 1H), 3.99 (s, 3H), 2.54 – 2.44 (m, 2H), 2.38 (dd, *J* = 7.2, 14.8 Hz, 2H), 2.24 – 2.12 (m, 1H), 2.09 – 1.98 (m, 1H), 1.00 (t, *J* = 7.2 Hz, 3H).
^{13}C NMR (100 MHz, $CDCl_3$) δ 210.8, 192.6, 162.4 (d, *J* = 245.0 Hz), 142.6, 133.2 (d, *J* = 3.0 Hz), 132.1, 129.5, 128.0 (d, *J* = 8.0 Hz), 127.7, 127.5 (d, *J* = 2.0 Hz), 115.5 (d, *J* = 21.0 Hz), 50.3, 40.0, 36.4, 36.1, 26.2, 7.9.
^{19}F NMR (376 MHz, $CDCl_3$) δ –115.01 (s, 1F).
HRMS (ESI-FT) calculated for $C_{19}H_{22}FN_2O_2^+$ ([M]+H$^+$) = 329.1660, Found 329.1655.
$[\alpha]_D^{21}$ = +13.8 (*c* = 0.50, in CH_2Cl_2).
IR (film): 1712, 1671, 1601, 1508, 1403, 1288, 1226, 1155, 972 cm^{-1}.
Chiral HPLC spectrum of **2v**:

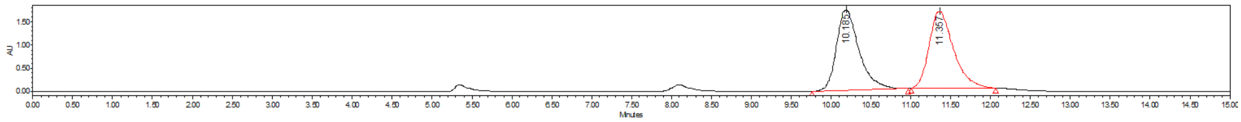

	Retention Time	Area	% Area
1	10.185	34024478	49.54
2	11.357	34655963	50.46

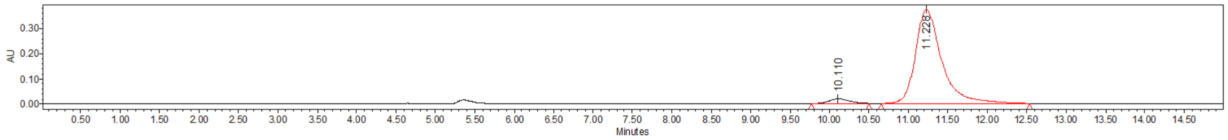

	Retention Time	Area	% Area
1	10.110	358197	3.96
2	11.228	8677071	96.04

(*E*)-1-(1-Methyl-1H-imidazol-2-yl)-2-(2-(naphthalen-2-yl)vinyl)heptane-1,5-dione

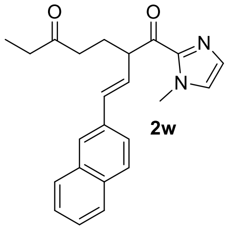

2w

Colourless oil. 61% yield (91% ee) was obtained according to the general procedure at 25 °C for 3 h.
HPLC (Chiral **IA** column), *i*-PrOH/Hexane = 20/80, Flow rate: 1.0 mL/min, 254 nm, t_r (minor) = 11.68 min, t_r (major) = 13.44 min.
^{1}H NMR (400 MHz, $CDCl_3$) δ 7.81 – 7.67 (m, 3H), 7.70 (s, 1H), 7.57 (dd, *J* = 8.8, 1.2 Hz, 1H), 7.47 – 7.37 (m, 2H), 7.18 (s, 1H), 7.04 (s, 1H), 6.76 (d, *J* = 15.6 Hz, 1H), 6.40 (dd, *J* = 15.6, 8.8 Hz, 1H), 4.71 (dd, *J* = 15.2, 8.4 Hz, 1H), 4.00 (s, 3H), 2.60 – 2.45 (m, 2H), 2.39 (dd, *J* = 14.4, 7.2 Hz, 2H), 2.29 – 2.18 (m, 1H), 2.15 – 2.03 (m, 1H), 1.01 (t, *J* = 7.2 Hz, 3H).
^{13}C NMR (100 MHz, $CDCl_3$) δ 210.8, 192.6, 142.7, 134.5, 133.7, 133.5, 133.1, 129.5, 128.2, 128.2, 128.1, 127.7, 127.7, 126.4, 126.3, 125.9, 123.6, 50.5, 40.0, 36.4, 36.1, 26.2, 7.9.
HRMS (ESI-FT) calculated for $C_{23}H_{25}N_2O_2^+$ ([M]+H$^+$) = 361.1911, Found 361.1906.
$[\alpha]_D^{22}$ = +24.2 (*c* = 0.48, in CH_2Cl_2).
IR (film): 1711, 1671, 1403, 1289, 1155, 970 cm^{-1}.
Chiral HPLC spectrum of **2w**:

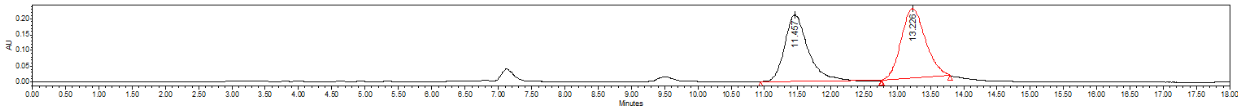

	Retention Time	Area	% Area

1	11.457	5196703	48.30
2	13.226	5562216	51.70

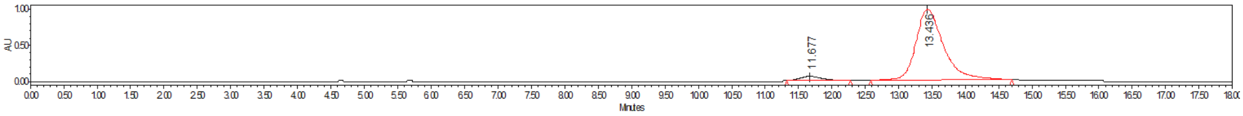

	Retention Time	Area	% Area
1	11.677	1371701	4.54
2	13.436	28850224	95.46

Tert-butyl ((*R*)-1-methyl-3-((*S*,*E*)-1-(1-methyl-1H-imidazol-2-yl)-1-oxo-4-phenylbut-3-en-2-yl)-2-oxoindolin-3-yl)carbamate

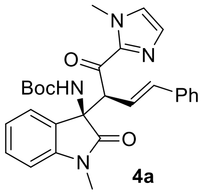

4a

Colourless foam. 95% yield (91% ee, >19:1 dr) was obtained according to the general procedure at 0 °C for 12 h.
UPC^2 (Chiral **IC-3**), CO_2/MeOH = 80/20, Flow rate: 1.0 mL/min, 254 nm, t_r (minor) = 6.76 min, t_r (major) = 6.21 min for major isomer.
^{1}H NMR (400 MHz, $CDCl_3$) δ 7.34 – 7.28 (m, 1H), 7.25 – 7.21 (m, 4H), 7.20 (s, 1H), 7.18 (d, *J* = 3.6 Hz, 1H), 7.17 – 7.12 (m, 2H), 7.05 (s, 1H), 6.94 (t, *J* = 7.6 Hz, 1H), 6.75 (d, *J* = 7.6 Hz, 1H), 6.46 – 6.30 (m, 2H), 4.92 (d, *J* = 8.8 Hz, 1H), 4.00 (s, 3H), 3.21 (s, 3H), 1.38 – 0.94 (m, 9H).
^{13}C NMR (100 MHz, $CDCl_3$) δ 188.5, 174.3, 154.0, 143.7, 143.2, 136.6, 136.1, 129.7, 129.0, 128.5, 128.2, 127.9, 126.7, 122. 7, 122.4, 121.6, 108.1, 80.1, 63.4, 54.9, 36.5, 28.1, 26.6.
HRMS (ESI-FT) calculated for $C_{28}H_{31}N_4O_4^+$ ($[M]+H^+$) = 487.2340, Found 487.2330.
$[\alpha]^{23}_{\lambda}$ = +122.8 (*c* = 0.93, in CH_2Cl_2, λ = 436 nm).
IR (film): 1711, 1666, 1611, 1471, 1399, 1468, 1252, 1162, 1023, 978 cm^{-1}.
UPC^2 spectrum of **4a**:

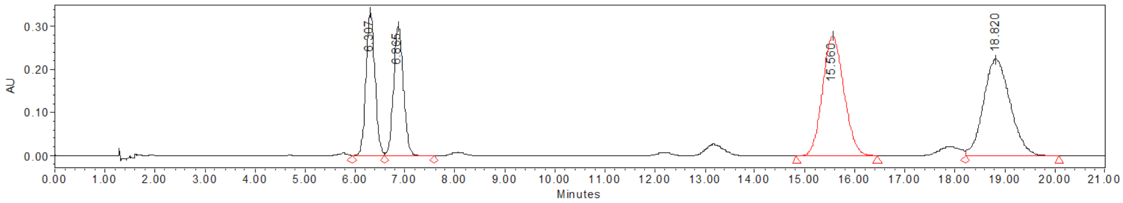

	Retention Time	Area	% Area
1	6.307	4229714	16.85
2	6.865	4177486	16.65
3	15.560	8315587	33.14
4	18.820	8371927	33.36

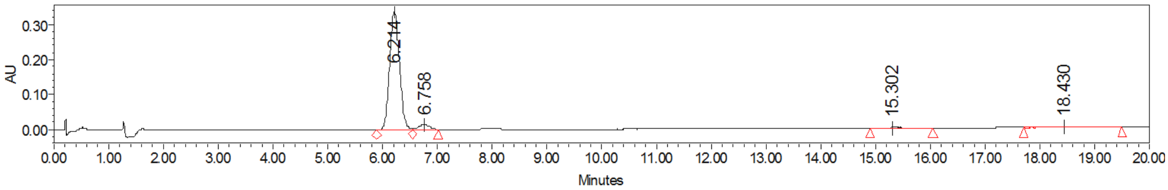

	Retention Time	Area	% Area
1	6.214	4213517	92.80
2	6.758	196020	4.32
3	15.302	39022	0.86
4	18.430	91923	2.02

Tert-butyl ((*R*)-5-chloro-1-methyl-3-((*S*,*E*)-1-(1-methyl-1H-imidazol-2-yl)-1-oxo-4-phenylbut-3-en-2-yl)-2-oxoindolin-3-yl) carbamate

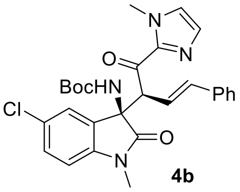

4b

Colourless foam. 87% yield (90% ee, >19:1 dr) was obtained according to the general procedure at 0 °C for 5 h.
UPC^2 (Chiral **IC-3**), CO_2/MeOH = 80/20, Flow rate: 1.0 mL/min, 254 nm, t_r (minor) = 5.90 min, t_r (major) = 5.18 min for major isomer.
^{1}H NMR (400 MHz, $CDCl_3$) δ 7.30 (s, 1H), 7.27 – 7.24 (m, 4H), 7.23 – 7.13 (m, 4H), 7.07 (s, 1H), 6.68 (d, *J* = 8.4 Hz, 1H), 6.39 (d, *J* = 5.2 Hz, 2H), 4.90 (dd, *J* = 6.0, 2.4 Hz, 1H), 4.02 (s, 3H), 3.20 (s, 3H), 1.39 – 1.03 (m, 9H).
^{13}C NMR (100 MHz, $CDCl_3$) δ 188.2, 173.9, 153.9, 143.1, 142.3, 136.5, 136.5, 129.8, 128.9, 128.6, 128.4, 128.0, 127.7, 126.7, 123.2, 121.0, 109.0, 80.4, 63.2, 54.6, 36.5, 28.2, 26.8.

HRMS (ESI-FT) calculated for $C_{28}H_{30}{}^{34.9689}ClN_4O_4^+$ ([M]+H$^+$) = 521.1950, Found 521.1957.
HRMS (ESI-FT) calculated for $C_{28}H_{30}{}^{36.9659}ClN_4O_4^+$ ([M]+H$^+$) = 523.1921, Found 523.1932.
$[\alpha]^{23}_{\lambda}$ = +48.1 (*c* = 0.64, in CH_2Cl_2, λ = 436 nm).
IR (film): 1713, 1670, 1610, 1401, 1364, 1255,1163, 1102, 968 cm^{-1}.
UPC^2 spectrum of **4b**:

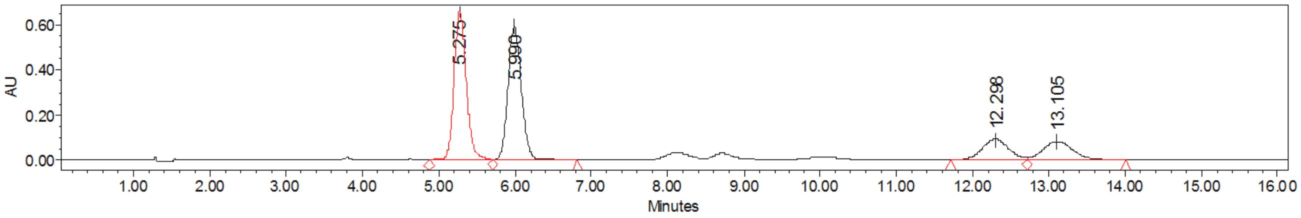

	Retention Time	Area	% Area
1	5.275	7432751	38.95
2	5.990	7248495	37.99
3	12.298	2203373	11.55
4	13.105	2197027	11.51

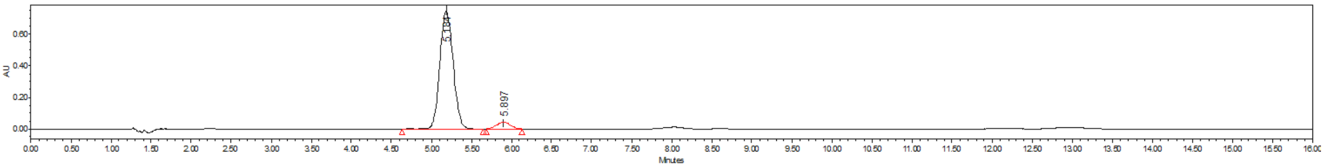

	Retention Time	Area	% Area
1	5.184	8589367	94.75
2	5.897	476242	5.25

Tert-butyl ((*R*)-5-iodo-1-methyl-3-((*S*,*E*)-1-(1-methyl-1H-imidazol-2-yl)-1-oxo-4-phenylbut-3-en-2-yl)-2-oxoindolin-3-yl) carbamate

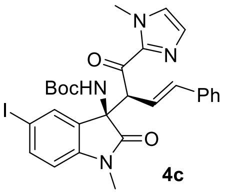

4c

Colourless foam. 90% yield (90% ee, >19:1 dr) was obtained according to the general procedure at 0 °C for 6 h.
UPC^2 (Chiral **IC-3**), CO_2/MeOH = 80/20, Flow rate: 1.0 mL/min, 254 nm, t_r (minor) = 8.39 min, t_r (major) = 7.24 min for major isomer.
^{1}H NMR (400 MHz, $CDCl_3$) δ 7.50 (d, *J* = 8.4 Hz, 1H), 7.45 – 7.38 (m, 1H), 7.30 – 7.23 (m, 5H), 7.22 – 7.17 (m, 1H), 7.14 (s, 1H), 7.08 (s, 1H), 6.53 (d, *J* = 8.5 Hz, 1H), 6.43 – 6.35 (m, 2H), 4.89 (d, *J* = 7.6 Hz, 1H), 4.03 (s, 3H), 3.19 (s, 3H), 1.42 – 1.03 (m, 9H).
^{13}C NMR (100 MHz, $CDCl_3$) δ 188.3, 173.6, 153.9, 143.4, 143.2, 137.7, 136.6, 136.5, 131.5, 129.8, 128.6, 128.4, 128.0, 126.7, 120.9, 110.2, 84.6, 80.5, 63.1, 54.5, 36.6, 28.2, 26.7.
HRMS (ESI-FT) calculated for $C_{28}H_{30}IN_4O_4^+$ ([M]+H$^+$) = 613.1306, Found 613.1316.
$[\alpha]^{25}_{\lambda}$ = –22.2 (*c* = 0.99, in CH_2Cl_2, λ = 436 nm).
IR (film): 1711, 1666, 1603, 1481, 1399, 1360, 1253,1160, 1097, 964 cm^{-1}.
UPC^2 spectrum of **4c**:

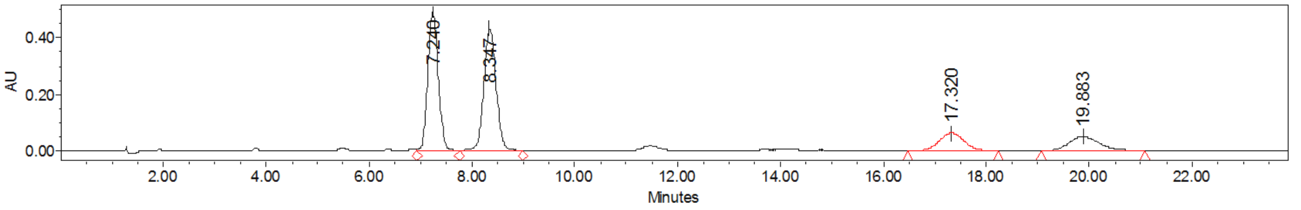

	Retention Time	Area	% Area
1	7.240	7034311	38.11
2	8.347	7232513	39.18
3	17.320	2122342	11.50
4	19.883	2070460	11.22

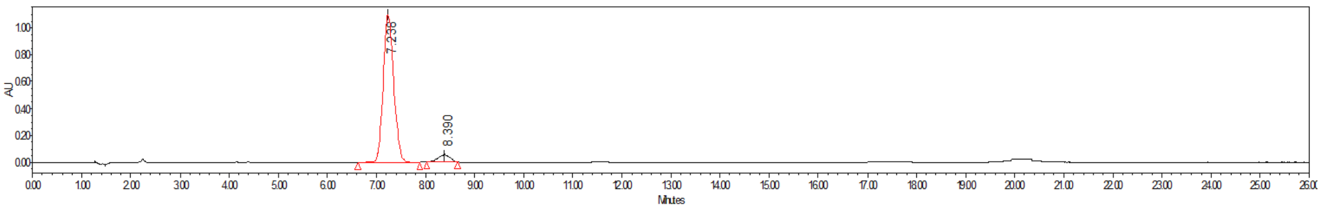

	Retention Time	Area	% Area
1	7.238	16327548	95.18
2	8.390	826516	4.82

Tert-butyl ((*R*)-1,5-dimethyl-3-((*S*,*E*)-1-(1-methyl-1H-imidazol-2-yl)-1-oxo-4-phenylbut-3-en-2-yl)-2-oxoindolin-3-yl)carbamate

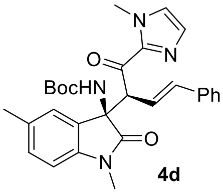

4d

Colourless foam. 75% yield (90% ee, >19:1 dr) was obtained according to the general procedure at 0 °C for 12 h.
UPC² (Chiral **IC-3**), CO_2/MeOH = 80/20, Flow rate: 1.0 mL/min, 254 nm, t_r (minor) = 7.27 min, t_r (major) = 6.63 min for major isomer.
¹H NMR (400 MHz, $CDCl_3$) δ 7.26 (s, 1H), 7.25 – 7.20 (m, 4H), 7.20 – 7.17 (m, 1H), 7.15 (s, 1H), 7.05 (s, 1H), 6.99 (d, *J* = 8.4 Hz, 2H), 6.63 (d, *J* = 8.0 Hz, 1H), 6.46 – 6.31 (m, 2H), 4.91 (d, *J* = 8.4 Hz, 1H), 4.00 (s, 3H), 2.24 (s, 3H), 1.35 – 0.98 (m, 9H).
¹³C NMR (100 MHz, $CDCl_3$) δ 189.3, 174.3, 154.1, 143.4, 141.4, 136.7, 136.1, 131.8, 129.7, 129.2, 128.5, 128.1, 127.9, 126.7, 123.6, 121.7, 107.8, 80.1, 63.4, 55.0, 36.5, 28.2, 26.7, 21.3.
HRMS (ESI-FT) calculated for $C_{29}H_{33}N_4O_4^+$ ($[M]+H^+$) = 501.2496, Found 501.2497.
$[\alpha]_D^{23}$ = +20.7 (*c* = 0.16, in CH_2Cl_2).
IR (film): 1711, 1667, 1497, 1400, 1361, 1252,1163, 968 cm^{-1}.
UPC² spectrum of **4d**:

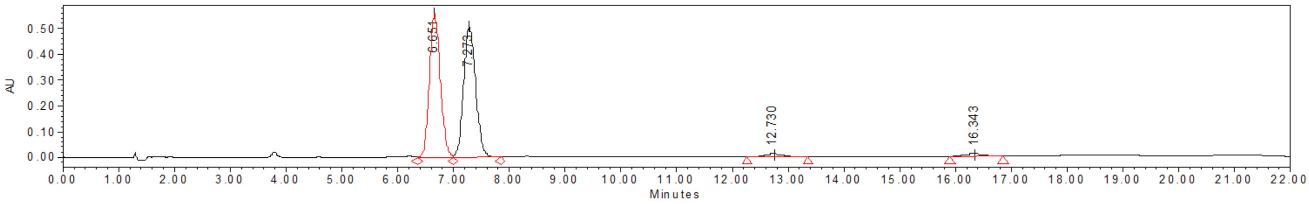

	Retention Time	Area	% Area
1	6.651	7596691	48.20
2	7.273	7621171	48.35
3	12.730	289738	1.84
4	16.343	254387	1.61

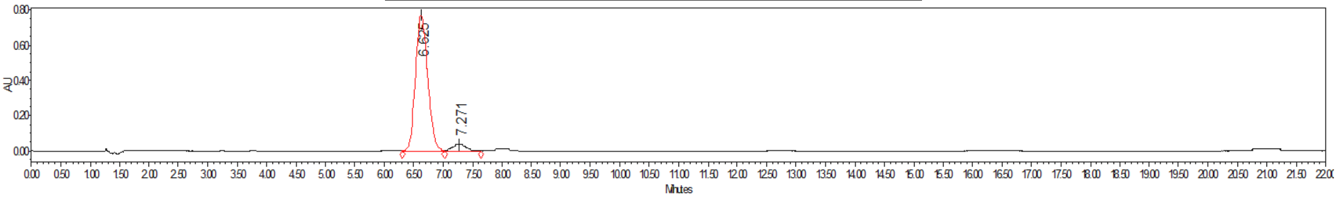

	Retention Time	Area	% Area
1	6.625	11169564	94.97
2	7.271	591064	5.03

Tert-butyl ((*R*)-6-chloro-1-methyl-3-((*S*,*E*)-1-(1-methyl-1H-imidazol-2-yl)-1-oxo-4-phenylbut-3-en-2-yl)-2-oxoindolin-3-yl) carbamate

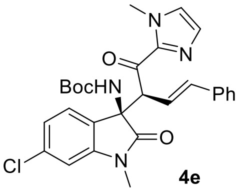

4e

Colourless foam. 88% yield (90% ee, >19:1 dr) was obtained according to the general procedure at 0 °C for 16 h.
UPC² (Chiral **IC-3**), CO_2/MeOH = 80/20, Flow rate: 1.0 mL/min, 254 nm, t_r (minor) = 6.37 min, t_r (major) = 5.43 min for major isomer.
¹H NMR (400 MHz, $CDCl_3$) δ 7.26 – 7.18 (m, 6H), 7.15 (s, 1H), 7.09 – 7.02 (m, 2H), 6.92 (d, *J* = 7.6 Hz, 1H), 6.77 (s, 1H), 6.44 – 6.36 (m, 2H), 4.93 – 4.88 (m, 1H), 4.00 (s, 3H), 3.20 (s, 3H), 1.38 – 1.06 (m, 9H).

^{13}C NMR (100 MHz, $CDCl_3$) δ 188.2, 174.3, 154.0, 145.0, 143.0, 136.5, 134.7, 129.8, 128.6, 128.4, 128.1, 126.7, 123.7, 122.2, 121.2, 109.0, 80.3, 62.8, 54.7, 36.5, 28.2, 26.7.
HRMS (ESI-FT) calculated for $C_{28}H_{30}{}^{34.9689}ClN_4O_4^+$ ([M]+H$^+$) = 521.1950, Found 521.1940.
HRMS (ESI-FT) calculated for $C_{28}H_{30}{}^{36.9659}ClN_4O_4^+$ ([M]+H$^+$) = 523.1921, Found 523.1916.
$[\alpha]_D^{24}$ = +19.9 (*c* = 0.91, in CH_2Cl_2).
IR (film): 1711, 1667, 1607, 1488, 1399, 1367, 1288, 1247, 1161, 1072, 978 cm^{-1}.
UPC^2 spectrum of **4e**:

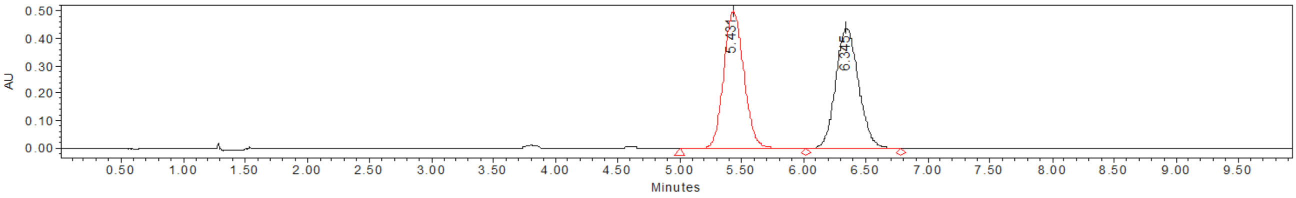

	Retention Time	Area	% Area
1	5.431	5653907	50.13
2	6.345	5623567	49.87

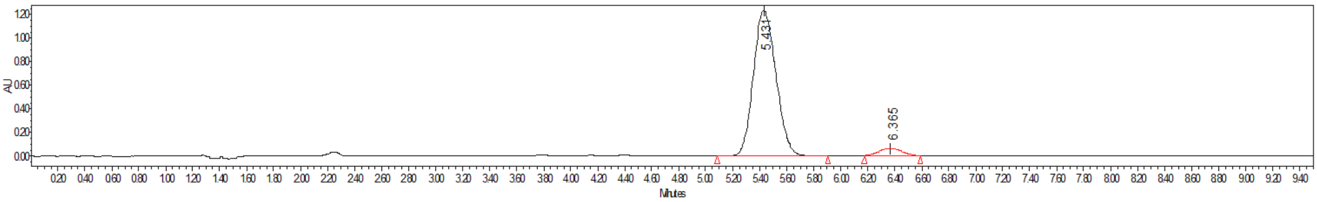

	Retention Time	Area	% Area
1	5.431	14347281	95.00
2	6.365	755908	5.00

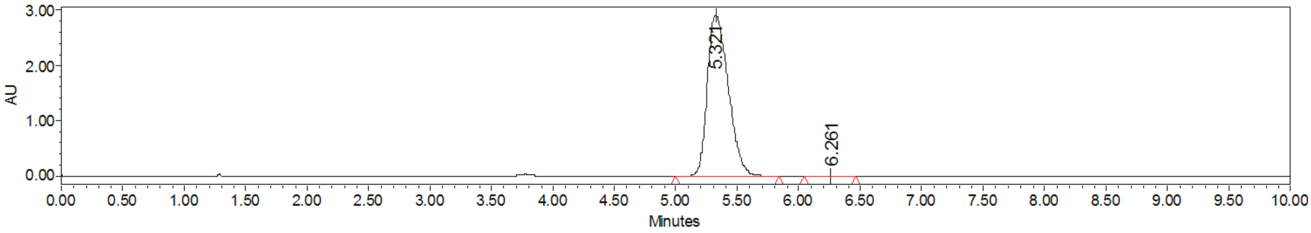

	Retention Time	Area	% Area
1	5.321	34884469	99.77
2	6.261	79095	0.23

Tert-butyl ((*R*)-6-fluoro-1-methyl-3-((*S*,*E*)-1-(1-methyl-1H-imidazol-2-yl)-1-oxo-4-phenylbut-3-en-2-yl)-2-oxoindolin-3-yl) carbamate

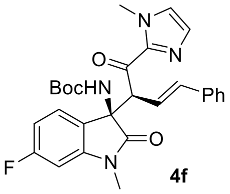

4f

Colourless foam. 98% yield (88% ee, 19:1 dr) was obtained according to the general procedure at 0 °C for 24 h.
UPC^2 (Chiral **IC-3**), CO_2/MeOH = 80/20, Flow rate: 1.0 mL/min, 254 nm, t_r (minor) = 4.83 min, t_r (major) = 4.23 min for major isomer.
^{1}H NMR (400 MHz, $CDCl_3$) δ 7.30 (s, 1H), 7.27 – 7.22 (m, 4H), 7.21 – 7.17 (m, 1H), 7.15 (s, 1H), 7.12 – 7.04 (m, 2H), 6.61 (t, *J* = 8.8 Hz, 1H), 6.51 (dd, *J* = 0.8 Hz, *J* = 8.8 Hz, 1H), 6.45 – 6.33 (m, 2H), 4.91 (d, *J* = 7.6 Hz, 1H), 4.00 (s, 3H), 3.19 (s, 3H), 1.42 – 1.00 (m, 9H).
^{13}C NMR (100 MHz, $CDCl_3$) δ 188.5, 174.6, 164.8, 162.3, 154.0, 145.3 (d, *J* = 11.0 Hz), 143.1, 136.5, 136.4, 129.8, 128.6, 128.3, 128.0, 126.7, 123.8 (d, *J* = 9.0 Hz), 121.3, 108.4 (d, *J* = 22.0 Hz), 97.0 (d, *J* = 25.0 Hz), 80.2, 62.8, 54.8, 36.6, 28.2, 26.8.
^{19}F NMR (376 MHz, $CDCl_3$) δ –111.28 (s, 1F).
HRMS (ESI-FT) calculated for $C_{28}H_{30}FN_4O_4^+$ ([M]+H$^+$) = 505.2246, Found 505.2243.
$[\alpha]^{23}_{\lambda}$ = +121.5 (*c* = 0.99, in CH_2Cl_2, λ = 436 nm).
IR (film): 1715, 1671, 1616, 1499, 1472, 1401, 1374, 1250, 1164, 1082, cm^{-1}.
UPC^2 spectrum of **4f**:

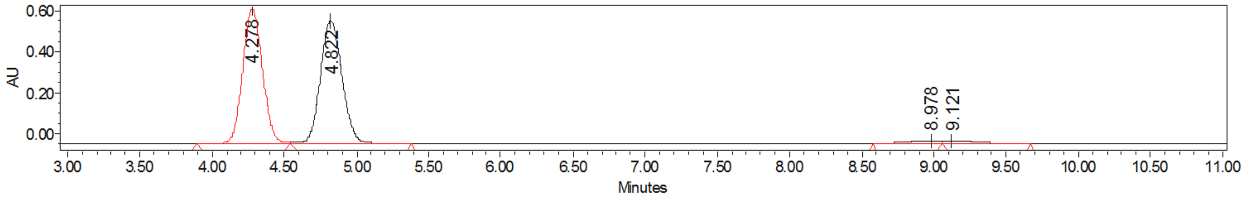

	Retention Time	Area	% Area
1	4.278	6233696	48.27
2	4.822	6240560	48.32
3	8.978	219146	1.70
4	9.121	222069	1.72

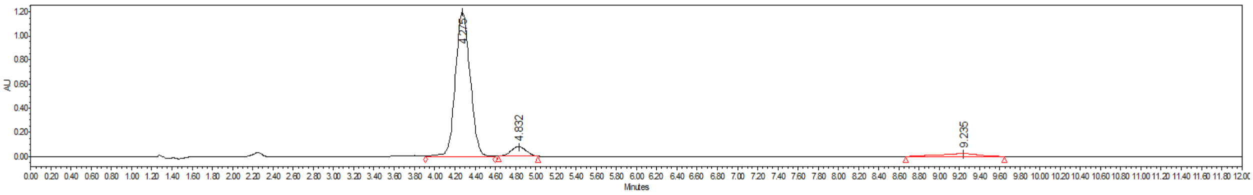

	Retention Time	Area	% Area
1	4.275	12147195	89.65
2	4.832	785735	5.80
3	9.235	616953	4.55

Tert-butyl ((*R*)-7-bromo-1-methyl-3-((*S*,*E*)-1-(1-methyl-1H-imidazol-2-yl)-1-oxo-4-phenylbut-3-en-2-yl)-2-oxoindolin-3-yl) carbamate

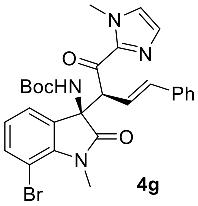

4g

Colourless foam. 95% yield (88% ee, 19:1 dr) was obtained according to the general procedure at 0 °C for 16 h.
UPC^2 (Chiral **IC-3**), CO_2/MeOH = 80/20, Flow rate: 1.0 mL/min, 254 nm, t_r (minor) = 7.87 min, t_r (major) = 6.89 min for major isomer.
^{1}H NMR (400 MHz, $CDCl_3$) δ 7.50 – 7.35 (m, 1H), 7.30 (d, *J* = 8.0 Hz, 1H), 7.28 – 7.19 (m, 5H), 7.16 (s, 1H), 7.14 – 7.06 (m, 2H), 6.85 – 6.72 (m, 1H), 6.41 – 6.27 (m, 2H), 4.85 (d, *J* = 8.0 Hz, 1H), 4.00 (s, 3H), 3.60 (s, 3H), 3.52 (s, 1H), 1.39 – 1.01 (m, 9H).
^{13}C NMR (100 MHz, $CDCl_3$) δ 188.7, 174.8, 153.9, 143.2, 141.0, 136.5, 134.6, 129.8, 128.6, 128.3, 128.0, 126.7, 123.5, 121.7, 121.2, 110.3, 103.4, 80.1, 63.1, 55.1, 36.6, 30.2, 28.2.
HRMS (ESI-FT) calculated for $^{78.9183}C_{28}H_{30}BrN_4O_4^+$ ($[M]+H^+$) = 565.1445, Found 565.1455.
HRMS (ESI-FT) calculated for $^{80.9163}C_{28}H_{30}BrN_4O_4^+$ ($[M]+H^+$) = 567.1424, Found 567.1416.
$[\alpha]^{23}_{\lambda}$ = +42.2 (*c* = 1.15, in CH_2Cl_2, λ = 436 nm).
IR (film): 1711, 1670, 1605, 1458, 1399, 1363, 1254, 1160, 1104, 1055, 964 cm^{-1}.
UPC^2 spectrum of **4g**:

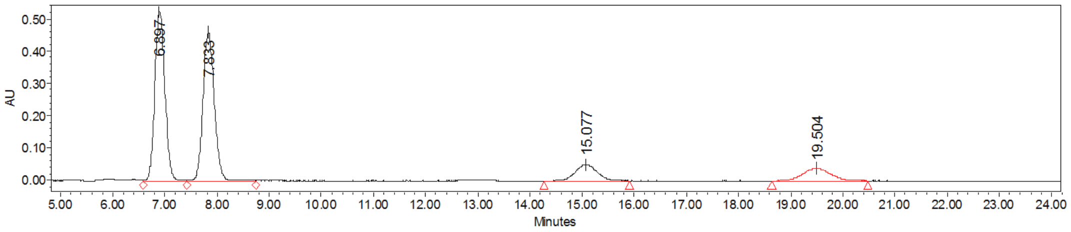

	Retention Time	Area	% Area
1	6.897	6924979	41.59
2	7.833	6895829	41.41
3	15.077	1415800	8.50
4	19.504	1414462	8.49

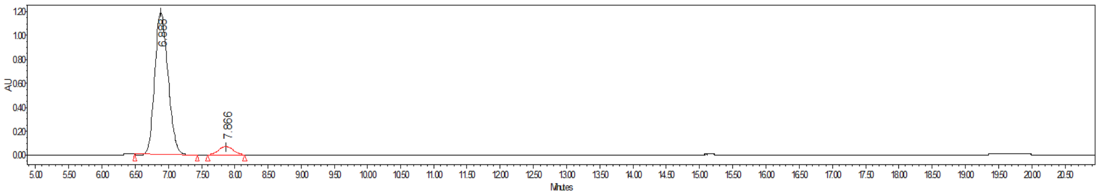

	Retention Time	Area	% Area
1	6.888	16453710	93.96
2	7.866	1058386	6.04

Tert-butyl ((*R*)-1-methyl-3-((*S*,*E*)-1-(1-methyl-1H-imidazol-2-yl)-4-(naphthalen-2-yl)-1-oxobut-3-en-2-yl)-2-oxoindolin-3-yl) carbamate

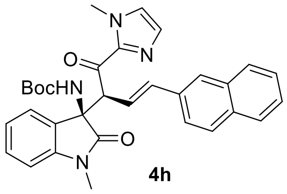

4h

Colourless foam. 99% yield (88% ee, 19:1 dr) was obtained according to the general procedure at 0 °C for 36 h.
UPC^2 (Chiral **IC-3**), CO_2/MeOH = 80/20, Flow rate: 1.0 mL/min, 254 nm, t_r (minor) = 17.73 min, t_r (major) = 14.81 min for major isomer.
^{1}H NMR (400 MHz, $CDCl_3$) δ 7.79 – 7.68 (m, 3H), 7.58 (s, 1H), 7.50 – 7.35 (m, 4H), 7.22 – 7.12 (m, 3H), 7.06 (s, 1H), 6.95 (t, *J* = 7.2 Hz, 1H), 6.75 (d, *J* = 7.6 Hz, 1H), 6.61 – 6.45 (m, 2H), 4.98 (d, *J* = 8.4 Hz, 1H), 4.02 (s, 3H), 3.22 (s, 3H), 1.43 – 0.98 (m, 9H).
^{13}C NMR (100 MHz, $CDCl_3$) δ 188.6, 174.4, 166.3, 154.1, 143.7, 143.3, 136.2, 134.1, 133.5, 133.2, 129.7, 129.0, 128.3, 128.2, 128.1, 127.7, 126.7, 126.3, 126.0, 123.8, 122.7, 122.4, 122.0, 108.2, 80.1, 63.5, 54.9, 36.6, 28.1, 26.6.
HRMS (ESI-FT) calculated for $C_{32}H_{33}N_4O_4^+$ ($[M]+H^+$) = 537.2496, Found 537.2492.
$[\alpha]^{23}_{\lambda}$ = +19.7 (*c* = 1.07, in CH_2Cl_2, λ = 436 nm).
IR (film): 1710, 1665, 1611, 1469, 1398, 1367, 1251, 1160, 1123, 1021, 962 cm^{-1}.
UPC^2 spectrum of **4h**:

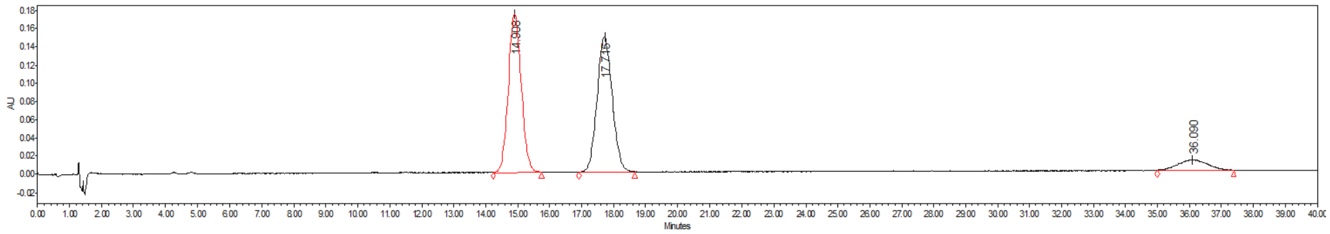

	Retention Time	Area	% Area
1	14.908	4930836	46.28
2	17.715	4936685	46.33
3	36.090	787596	7.39

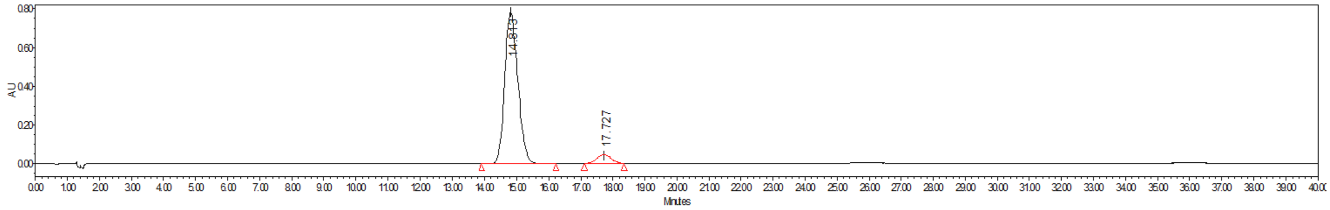

	Retention Time	Area	% Area
1	14.813	22372846	93.95
2	17.727	1441749	6.05

Tert-butyl (*E*)-(4-(1-(1-methyl-1H-imidazol-2-yl)-1-oxo-4-phenylbut-3-en-2-yl)-5-oxo-1,3-diphenyl-4,5-dihydro-1H-pyrazol-4-yl)carbamate

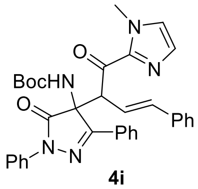

4i

Colourless foam. 94% yield (99% ee, >19:1 dr) was obtained according to the general procedure at 0 °C for 8 h.
UPC^2 (Chiral **IB3**), CO_2/MeOH = 90/10, Flow rate: 1.0 mL/min, 254 nm, t_r (minor) = 11.54 min, t_r (major) = 12.55 min.
^{1}H NMR (400 MHz, $CDCl_3$) δ 8.36 – 8.09 (m, 1H), 8.07 – 7.95 (m, 4H), 7.49 – 7.36 (m, 5H), 7.33 – 7.16 (m, 5H), 7.14 – 7.07 (m, 3H), 6.30 – 6.17 (m, 1H), 6.12 (d, *J* = 15.6 Hz, 1H), 5.03 (d, *J* = 9.6 Hz, 1H), 3.98 –3.88 (m, 3H), 1.34 (s, 4H), 1.13 (s, 5H).
^{13}C NMR (100 MHz, $CDCl_3$) δ 186.5/186.0 (rotamer), 170.5, 156.3/155.6 (rotamer), 154.1/153.3 (rotamer), 143.8, 138.3, 136.7/136.6 (rotamer), 136.2, 131.3/130.8 (rotamer), 130.5/130.2 (rotamer), 129.8/129.4 (rotamer), 129.0/128.8 (rotamer), 128.6, 128.4, 128.3, 128.1, 126.8, 126.7, 125.2, 120.0/119.8 (rotamer), 119.5/118.9 (rotamer), 82.0/80.7 (rotamer), 67.9/67.4 (rotamer), 53.8/53.6 (rotamer), 36.4, 28.4/28.0 (rotamer).
HRMS (ESI-FT) calculated for $C_{34}H_{33}N_5O_4Na^+$ ($[M]+Na^+$) = 598.2425, Found 598.2416.
$[\alpha]^{21}_{D}$ = –136.1 (*c* = 1.08, in CH_2Cl_2).
IR (film): 1713, 1676, 1596, 1494, 1398, 1284, 1157, 1109, 972 cm^{-1}.
UPC^2 spectrum of **4i**:

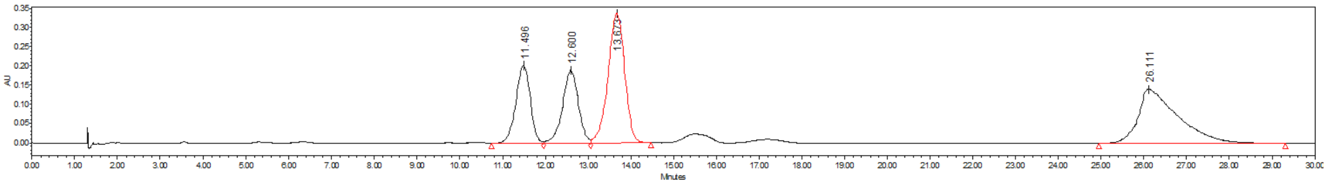

	Retention Time	Area	% Area
1	11.496	4736779	17.16
2	12.600	4802408	17.40
3	13.673	9094403	32.95
4	26.111	8969183	32.49

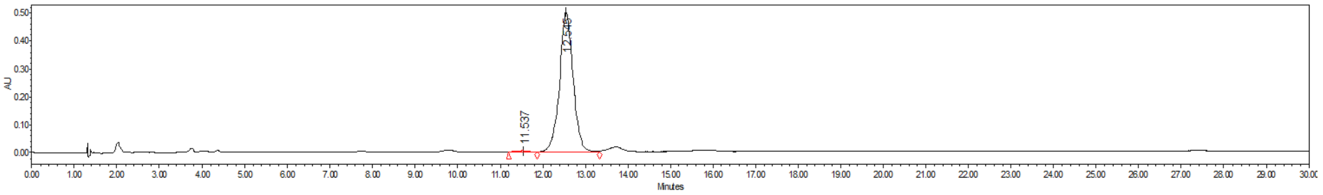

	Retention Time	Area	% Area
1	11.537	64401	0.58
2	12.545	10951797	99.42

Tert-butyl-(*E*)-(4-(4-(4-bromophenyl)-1-(1-methyl-1H-imidazol-2-yl)-1-oxobut-3-en-2-yl)-5-oxo-1,3-diphenyl-4,5-dihydro-1H-pyrazol-4-yl)carbamate

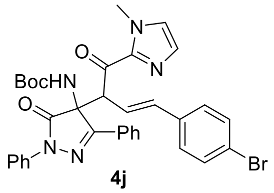

4j

Colourless foam. 98% yield (98% ee, 14:1 dr) was obtained according to the general procedure at 0 °C for 12 h.
UPC^2 (Chiral **IB3**), CO_2/MeOH = 85/15, Flow rate: 1.0 mL/min, 254 nm, t_r (minor) = 8.85 min, t_r (major) = 10.23 min.
^{1}**H NMR** (400 MHz, $CDCl_3$) δ 8.42 – 8.10 (m, 1H), 8.07 – 7.94 (m, 4H), 7.47 –7.37 (m, 5H), 7.32 (d, *J* = 8.4 Hz, 2H), 7.29 –7.16 (m, 2H), 7.09 (s, 1H), 6.96 (d, *J* = 8.4 Hz, 2H), 6.30 – 6.15 (m, 1H), 6.03 (d, *J* = 16.0 Hz, 1H), 5.07 – 4.93 (m, 1H), 3.96 – 3.90 (m, 3H), 1.34 (s, 4H), 1.12 (s, 5H).
^{13}**C NMR** (100 MHz, $CDCl_3$) δ 186.3/185.8 (rotamer), 170.4, 156.2/155.5 (rotamer), 154.0/153.2 (rotamer), 143.7, 138.2, 135.4/135.3 (rotamer), 135.1, 131.7, 130.7/130.5 (rotamer), 130.3/129.8 (rotamer), 129.5/129.4 (rotamer), 129.0/128.9 (rotamer), 128.6/128.4 (rotamer), 128.4/128.3 (rotamer), 128.2, 126.7, 125.3, 122.0, 120.9/120.7 (rotamer), 119.4/118.8 (rotamer), 82.1/80.7 (rotamer), 67.9/67.3 (rotamer), 53.7/53.4 (rotamer), 36.4, 28.3/27.9 (rotamer).
HRMS (ESI-FT) calculated for $C_{34}H_{33}{}^{78.9183}BrN_5O_4^+$ ($[M]+Na^+$) = 654.1710, Found 654.1701.
HRMS (ESI-FT) calculated for $C_{34}H_{33}{}^{80.9163}BrN_5O_4^+$ ($[M]+Na^+$) = 656.1690, Found 656.1684.
$[\alpha]_D^{20}$ = −114.6 (*c* = 1.33, in CH_2Cl_2).
IR (film): 1723, 1676, 1595, 1493, 1401, 1283, 1159, 1071, 974 cm^{-1}.
UPC^2 spectrum of **4j**:

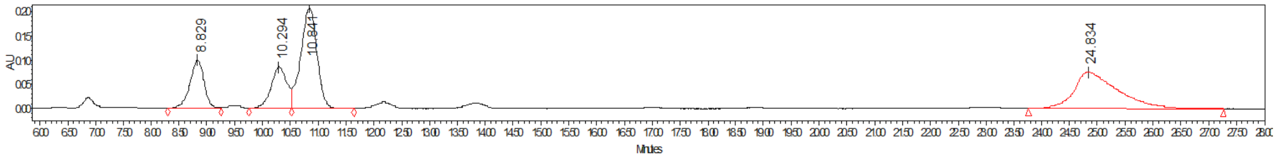

	Retention Time	Area	% Area
1	8.829	1742373	14.77
2	10.294	1688907	14.32
3	10.841	4222444	35.80
4	24.834	4139729	35.10

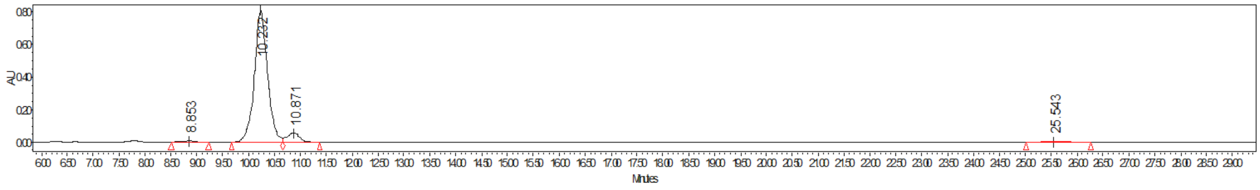

	Retention Time	Area	% Area
1	8.853	141393	0.92
2	10.232	14275504	92.40

3	10.871	957933	6.20
4	25.543	75233	0.49

Tert-butyl (*E*)-(4-(1-(1-methyl-1H-imidazol-2-yl)-1-oxo-4-(m-tolyl)but-3-en-2-yl)-5-oxo-1,3-diphenyl-4,5-dihydro-1H-pyrazol-4-yl)carbamate

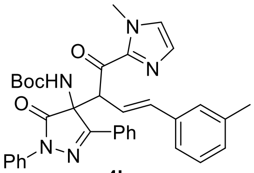

4k

Colourless foam. 99% yield (96% ee, 16:1 dr) was obtained according to the general procedure at 0 °C for 10 h.
UPC² (Chiral **IC3**), CO_2/MeOH = 80/20, Flow rate: 1.0 mL/min, 254 nm, t_r (minor) = 5.26 min, t_r (major) = 5.74 min.
¹H NMR (400 MHz, $CDCl_3$) δ 8.34 – 8.10 (m, 1H), 8.07 – 7.96 (m, 4H), 7.49 – 7.37 (m, 5H), 7.35 – 7.27 (m, 1H), 7.25 – 7.18 (m, 1H), 7.13 – 7.06 (m, 2H), 7.00 (d, *J* = 7.2 Hz, 1H), 6.95 (s, 1H), 6.90 (d, *J* = 7.6 Hz, 1H), 6.30 – 6.16 (m, 1H), 6.10 (d, *J* = 15.6 Hz, 1H), 5.03 (d, *J* = 10.0 Hz, 1H), 3.97 – 3.88 (m, 3H), 2.24 (s, 3H), 1.35 (s, 4H), 1.14 (s, 5H).
¹³C NMR (100 MHz, $CDCl_3$) δ 186.4/186.0 (rotamer), 170.4, 156.3/155.6 (rotamer), 154.0/153.3 (rotamer), 143.8, 138.3, 138.1, 136.8/136.7 (rotamer), 136.1, 131.2/130.8 (rotamer), 130.4/130.2 (rotamer), 129.7/129.4 (rotamer), 128.9, 128.8, 128.6, 128.4, 128.3, 127.3, 126.8, 125.2, 124.0, 119.8/119.6 (rotamer), 119.5/118.9 (rotamer), 82.0/80.7 (rotamer), 67.9/67.4 (rotamer), 53.8/53.6 (rotamer), 36.4, 28.3/28.0 (rotamer), 21.4.
HRMS (ESI-FT) calculated for $C_{35}H_{36}N_5O_4^+$ ([M]+H⁺) = 590.2762, Found 590.2759.
$[\alpha]_D^{23}$ = −125.8 (*c* = 1.16, in CH_2Cl_2).
IR (film): 1716, 1676, 1597, 1494, 1399, 1285, 1158, 1109, 973 cm^{-1}.
UPC² spectrum of **4k**:

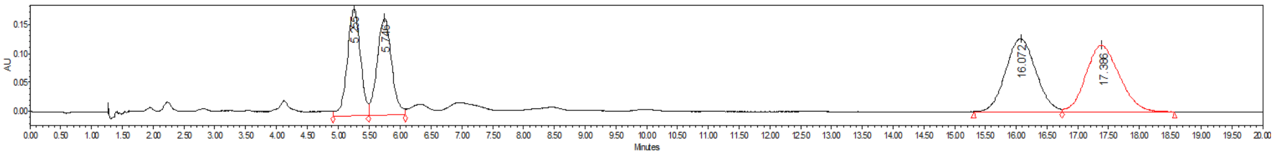

	Retention Time	Area	% Area
1	5.255	2722683	19.27
2	5.746	2746630	19.44
3	16.072	4309564	30.51
4	17.386	4347051	30.77

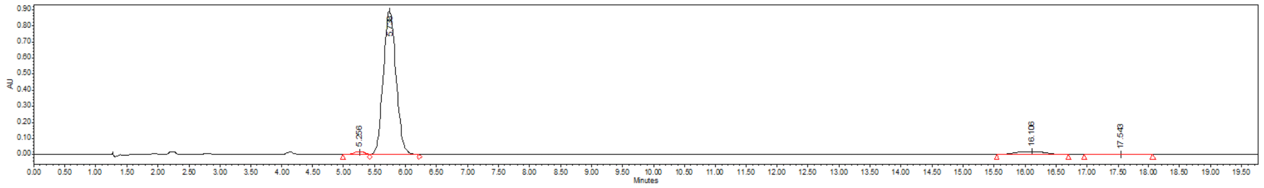

	Retention Time	Area	% Area
1	5.256	229335	1.67
2	5.738	12811240	93.01
3	16.106	612049	4.44
4	17.543	121069	0.88

Tert-butyl-(*E*)-(4-(4-(3-methoxyphenyl)-1-(1-methyl-1H-imidazol-2-yl)-1-oxobut-3-en-2-yl)-5-oxo-1,3-diphenyl-4,5-dihydro-1H-pyrazol-4-yl)carbamate

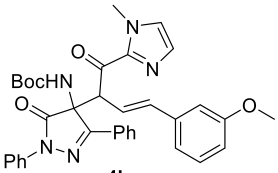

4l

Colourless foam. 97% yield (98% ee, >19:1 dr) was obtained according to the general procedure at 0 °C for 8 h.
UPC² (Chiral **OJH**), CO_2/MeOH = 90/10, Flow rate: 1.0 mL/min, 254 nm, t_r (minor) = 14.02 min, t_r (major) = 12.94 min.
¹H NMR (400 MHz, $CDCl_3$) δ 8.37 – 8.11 (m, 1H), 8.09 – 7.96 (m, 4H), 7.49 – 7.35 (m, 5H), 7.32 –7.27 (m, 1H), 7.25 – 7.17 (m, 1H), 7.14 – 7.07 (m, 2H), 6.74 (dd, *J* = 8.4, 2.4 Hz, 1H), 6.69 (d, *J* = 7.2 Hz, 1H), 6.63 (s, 1H), 6.29 – 6.16 (m, 1H), 6.10 (d, *J* = 16.0 Hz, 1H), 5.03 (d, *J* = 10.0 Hz, 1H), 3.97 – 3.89 (m, 3H), 3.66 (s, 3H), 1.34 (s, 4H), 1.13 (s, 5H).
¹³C NMR (100 MHz, $CDCl_3$) δ 186.3/185.9 (rotamer), 170.4, 159.7, 156.3/155.5 (rotamer), 154.0/153.2 (rotamer), 143.8, 138.3, 137.6, 136.6/136.5 (rotamer), 131.2/130.7 (rotamer), 130.5/130.2 (rotamer), 129.8, 129.5, 129.4, 129.0/128.9 (rotamer), 128.6, 128.4/128.3 (rotamer), 126.8, 125.2, 120.3/120.1 (rotamer), 119.5, 119.5/118.8 (rotamer), 114.3/111.3 (rotamer), 82.0/80.7 (rotamer), 67.9/67.4 (rotamer), 55.2, 53.7/53.5 (rotamer), 36.4, 28.3/27.9 (rotamer).
HRMS (ESI-FT) calculated for $C_{35}H_{36}N_5O_5^+$ ([M]+H⁺) = 606.2711, Found 606.2701.
$[\alpha]_D^{23}$ = −123.9 (*c* = 1.16, in CH_2Cl_2).

IR (film): 1714, 1676, 1596, 1493, 1399, 1260, 1158, 1046 cm^{-1}.
UPC^2 spectrum of **4l:**

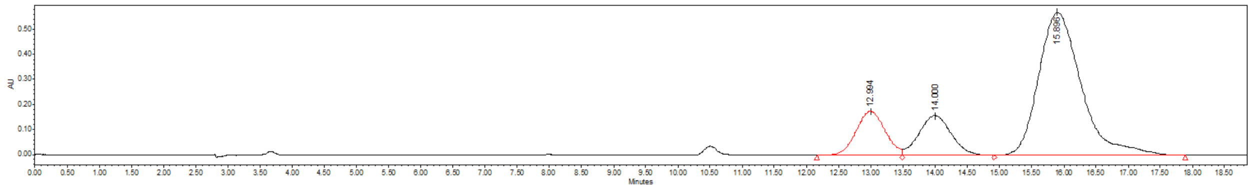

	Retention Time	Area	% Area
1	12.994	5461513	14.81
2	14.000	5428308	14.72
3	15.896	25988534	70.47

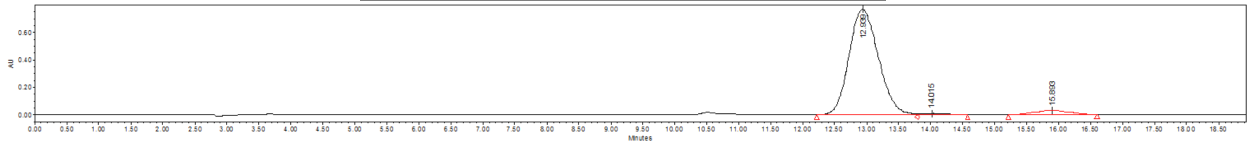

	Retention Time	Area	% Area
1	12.939	24347180	94.66
2	14.015	257327	1.00
3	15.893	1115171	4.34

Tert-butyl (*E*)-(4-(1-(1-methyl-1H-imidazol-2-yl)-1-oxo-4-(o-tolyl)but-3-en-2-yl)-5-oxo-1,3-diphenyl-4,5-dihydro-1H-pyrazol-4-yl)carbamate

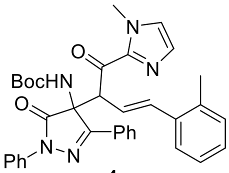

4m

Colourless foam. 98% yield (98% ee, >19:1 dr) was obtained according to the general procedure at 0 °C for 10 h.
UPC^2 (Chiral **ID3**), CO_2/MeOH = 95/5, Flow rate: 1.0 mL/min, 254 nm, t_r (minor) = 8.13 min, t_r (major) = 22.47 min.
^{1}H NMR (400 MHz, $CDCl_3$) δ 8.55 – 8.23 (m, 1H), 8.16 – 8.04 (m, 2H), 8.01 (d, *J* = 8.0 Hz, 2H), 7.48 – 7.37 (m, 5H), 7.33 – 7.18 (m, 3H), 7.13 – 6.99 (m, 4H), 6.34 (d, *J* = 15.6 Hz, 1H), 6.18 – 6.02 (m, 1H), 5.09 (t, *J* = 10.8 Hz, 1H), 4.03 – 3.94 (m, 3H), 1.88 (s, 3H), 1.33 (s, 4H), 1.12 (s, 5H).
^{13}C NMR (100 MHz, $CDCl_3$) δ 186.6/186.1 (rotamer), 170.5, 156.1/155.4 (rotamer), 154.1/153.3 (rotamer), 143.9, 138.3, 135.6, 135.3/135.2 (rotamer), 134.6, 131.1, 130.7/130.5 (rotamer), 130.2, 130.1, 129.7/129.3 (rotamer), 128.9, 128.8/128.7 (rotamer), 128.4/128.3, 128.0, 126.6, 126.3/126.1 (rotamer), 125.2, 121.0/120.9 (rotamer), 119.5/118.8 (rotamer), 82.0/80.6 (rotamer), 68.1/67.5 (rotamer), 54.3/54.1 (rotamer), 36.5, 28.3/27.9 (rotamer), 19.4.
HRMS (ESI-FT) calculated for $C_{35}H_{36}N_5O_4^+$ ($[M+H]^+$) = 590.2762, Found 590.2756.
$[\alpha]_D^{20}$ = –164.7 (*c* = 1.16, in CH_2Cl_2).
IR (film): 1724, 1678, 1596, 1495, 1401, 1286, 1160, 973 cm^{-1}.
UPC^2 spectrum of **4m**:

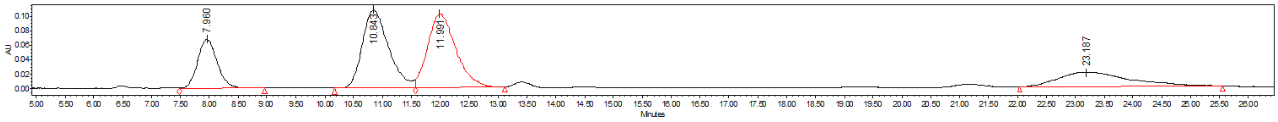

	Retention Time	Area	% Area
1	7.960	1620037	16.09
2	10.843	3403442	33.80
3	11.991	3401595	33.78
4	23.187	1643314	16.32

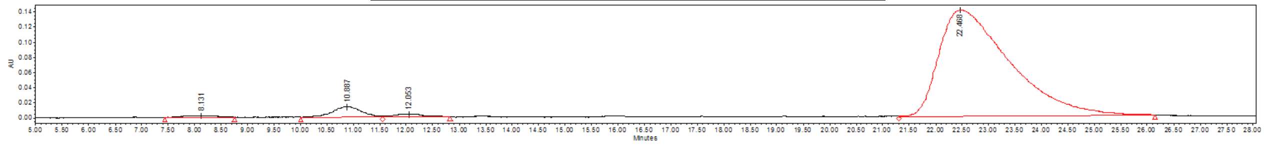

	Retention Time	Area	% Area
1	8.131	88818	0.64
2	10.887	536464	3.87
3	12.053	152922	1.10
4	22.468	13083416	94.39

Tert-butyl-(*E*)-(4-(1-(1-methyl-1H-imidazol-2-yl)-1-oxo-4-(thiophen-3-yl)but-3-en-2-yl)-5-oxo-1,3-diphenyl-4,5-dihydro-1H-pyrazol-4-yl)carbamate

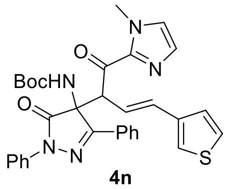

4n

Colourless foam. 99% yield (98% ee, >19:1 dr) was obtained according to the general procedure at 0 °C for 8 h.
UPC² (Chiral **ODH**), CO_2/MeOH = 85/15, Flow rate: 1.0 mL/min, 254 nm, t_r (minor) = 21.41 min, t_r (major) = 24.31 min.
¹H NMR (400 MHz, $CDCl_3$) δ 8.26 – 8.04 (m, 1H), 8.03 – 7.94 (m, 4H), 7.48 – 7.36 (m, 5H), 7.28 – 7.18 (m, 2H), 7.16 (dd, *J* = 4.8, 2.8 Hz, 1H), 7.07 (s, 1H), 6.99 (d, *J* = 3.6 Hz, 1H), 6.92 (d, *J* = 2.0 Hz, 1H), 6.19 – 6.00 (m, 2H), 4.99 (d, *J* = 9.6 Hz, 1H), 3.91 (s, 3H), 1.34 (s, 4H), 1.13 (s, 5H).
¹³C NMR (100 MHz, $CDCl_3$) δ 186.4/186.0 (rotamer), 170.4, 156.9/156.3 (rotamer), 154.0/153.2 (rotamer), 143.7, 138.7/138.3 (rotamer), 131.2, 130.7, 130.4/130.2 (rotamer), 129.8/129.4 (rotamer), 129.0, 128.8, 128.5, 128.4/128.3 (rotamer), 126.8, 126.1, 125.2, 123.1, 119.6, 119.5, 118.8, 82.0/80.7 (rotamer), 67.9/67.3 (rotamer), 53.8/53.5 (rotamer), 36.4, 28.3/28.0 (rotamer).
HRMS (ESI-FT) calculated for $C_{32}H_{32}N_5O_4^+S$ ($[M+H]^+$) = 582.2170, Found 582.2161.
$[\alpha]_D^{22}$ = –125.1 (*c* = 1.16, in CH_2Cl_2).
IR (film): 1714, 1676, 1596, 1494, 1398, 1256, 1158, 1106, 971 cm^{-1}.
UPC² spectrum of **4n**:

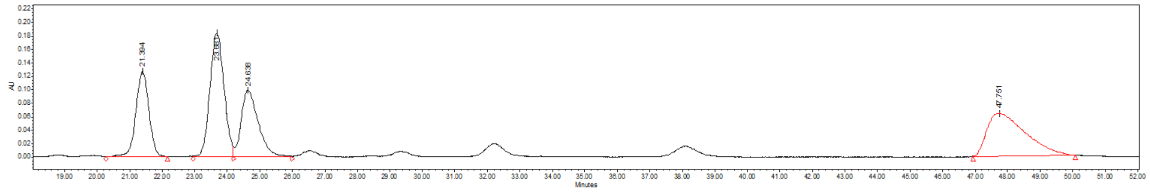

	Retention Time	Area	% Area
1	21.394	3542403	19.79
2	23.681	5546637	30.99
3	24.638	3603932	20.13
4	47.751	5207972	29.09

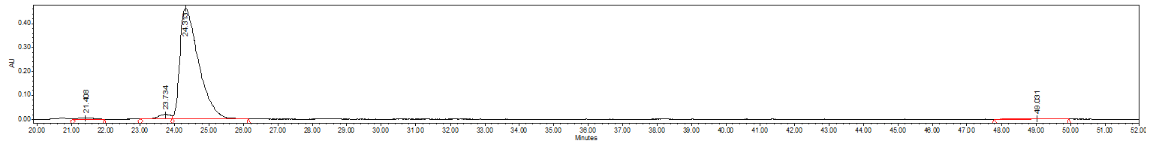

	Retention Time	Area	% Area
1	21.408	158890	0.87
2	23.734	514376	2.82
3	24.313	17477024	95.69
4	49.031	114024	0.62

Tert-butyl (*E*)-(4-(1-(1-methyl-1H-imidazol-2-yl)-1-oxo-5-phenylpent-3-en-2-yl)-5-oxo-1,3-diphenyl-4,5-dihydro-1H-pyrazol-4-yl)carbamate

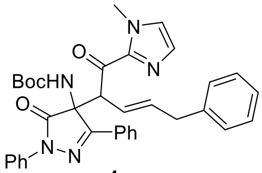

4o

Colourless foam. 96% yield (85% ee, 13:1 dr) was obtained according to the general procedure at 20 °C for 10 h.
UPC² (Chiral **IC3**), CO_2/MeOH = 85/15, Flow rate: 1.0 mL/min, 254 nm, t_r (minor) = 7.70 min, t_r (major) = 8.31 min.
¹H NMR (400 MHz, $CDCl_3$) δ 8.50 – 8.16 (m, 1H), 8.01 (d, *J* = 7.6 Hz, 4H), 7.46 – 7.36 (m, 5H), 7.27 – 7.18 (m, 3H), 7.16 – 7.10 (m, 3H), 7.09 (s, 1H), 6.92 – 6.83 (m, 2H), 5.65 – 5.52 (m, 1H), 5.42 – 5.32 (m, 1H), 4.90 (t, *J* = 10.4 Hz, 1H), 3.97 (s, 3H), 3.16 (d, *J* = 6.4 Hz, 2H), 1.29 (s, 4H), 1.09 (s, 5H).
¹³C NMR (100 MHz, $CDCl_3$) δ 186.8/186.3 (rotamer), 170.5, 156.0/155.3 (rotamer), 154.0/153.2 (rotamer), 144.0, 139.1/138.3 (rotamer), 136.8/136.7 (rotamer), 130.4/130.1 (rotamer), 129.7/129.2 (rotamer), 128.9, 128.9/128.8 (rotamer), 128.6, 128.6, 128.5, 128.4, 128.3/128.3 (rotamer), 126.5, 126.1, 125.1, 121.5, 119.4/118.8 (rotamer), 81.9/80.5, 67.8/67.3 (rotamer), 53.3, 38.9, 36.5, 28.3/27.9 (rotamer).
HRMS (ESI-FT) calculated for $C_{35}H_{36}N_5O_4^+$ ($[M+H]^+$) = 590.2762, Found 590.2756.
$[\alpha]_D^{25}$ = –132.4 (*c* = 1.11, in CH_2Cl_2).
IR (film): 1718, 1676, 1596, 1494, 1399, 1284, 1158, 1110, 973 cm^{-1}.
UPC² spectrum of **4o**:

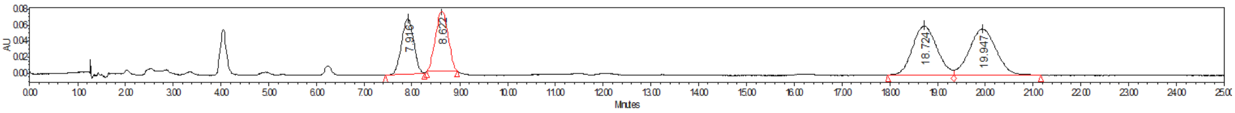

	Retention Time	Area	% Area
1	7.916	1216625	17.38

2	8.622	1368264	19.54
3	18.724	2161867	30.88
4	19.947	2254869	32.20

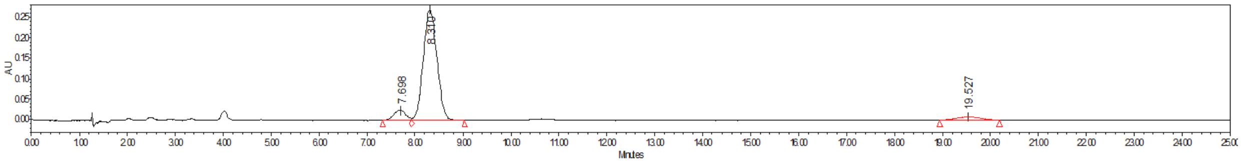

	Retention Time	Area	% Area
1	7.698	433694	7.14
2	8.310	5333524	87.81
3	19.527	306944	5.05

Tert-butyl (*E*)-(4-(1-(1-methyl-1H-imidazol-2-yl)-1-oxohept-3-en-2-yl)-5-oxo-1,3-diphenyl-4,5-dihydro-1H-pyrazol-4-yl)carbamate

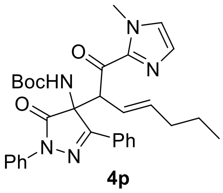

4p

Colourless foam. 97% yield (52% ee, 10:1 dr) was obtained according to the general procedure at 20 °C for 4 h.
UPC^2 (Chiral **OXH**), CO_2/MeOH = 85/15, Flow rate: 1.0 mL/min, 254 nm, t_r (minor) = 13.35 min, t_r (major) = 12.01 min.
^{1}H NMR (400 MHz, $CDCl_3$) δ 8.43 – 8.11 (m, 1H), 8.09 – 8.03 (m, 2H), 8.00 (d, *J* = 7.6 Hz, 2H), 7.45 – 7.39 (m, 5H), 7.25 – 7.15 (m, 2H), 7.08 (s, 1H), 5.52 – 5.45 (m, 1H), 5.28 – 5.20 (m, 1H), 4.85 (t, *J* = 10.8 Hz, 1H), 3.96 – 3.92 (m, 3H), 1.80 (dd, *J* = 14.0, 6.8 Hz, 2H), 1.44 – 1.35 (m, 1H), 1.30 (s, 4H), 1.09 (s, 5H), 0.92 – 0.85 (m, 1H), 0.69 (t, *J* = 7.2 Hz, 3H).
^{13}C NMR (100 MHz, $CDCl_3$) δ 187.1/186.7 (rotamer), 170.6, 169.59, 156.1/155.4 (rotamer), 154.0/153.3 (rotamer), 144.0, 138.5/138.3 (rotamer), 131.1/130.7 (rotamer), 130.4/130.1 (rotamer), 129.6/129.2 (rotamer), 128.9/128.8 (rotamer), 128.5, 128.2/128.1 (rotamer), 126.6, 126.5, 125.1, 120.1, 119.4/118.8 (rotamer), 81.8/80.4 (rotamer), 67.9/67.4 (rotamer), 53.6/53.5 (rotamer), 36.4, 34.5, 28.3/27.9 (rotamer), 22.0, 13.6.
HRMS (ESI-FT) calculated for $C_{31}H_{36}N_5O_4^+$ ([M]+H$^+$) = 542.2762, Found 542.2769.
$[\alpha]_D^{29}$ = –141.5 (*c* =1.06, in CH_2Cl_2).
IR (film): 1714, 1679, 1596, 1494, 1402, 1368, 1288, 1159, 1080 cm^{-1}.
UPC^2 spectrum of **4p**:

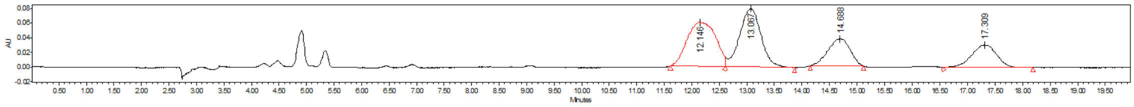

	Retention Time	Area	% Area
1	12.146	2147490	34.20
2	13.067	2137746	34.05
3	14.688	1035531	16.49
4	17.309	958239	15.26

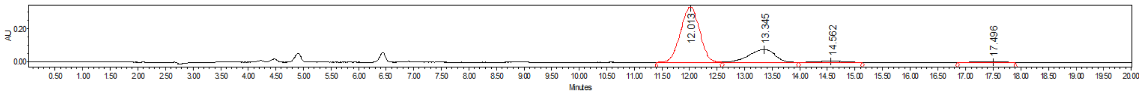

	Retention Time	Area	% Area
1	12.013	7863292	73.73
2	13.345	2455033	23.02
3	14.562	239168	2.24
4	17.496	107756	1.01

Tert-butyl (4-(3-cyclohexylidene-1-(1-methyl-1H-imidazol-2-yl)-1-oxopropan-2-yl)-5-oxo-1,3-diphenyl-4,5-dihydro-1H-pyrazol-4-yl)carbamate

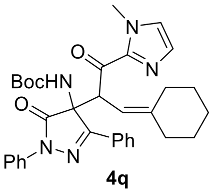

4q

Colourless foam. 75% yield (99% ee, >19:1 dr) was obtained according to the general procedure at 20 °C for 10 h.
UPC^2 (Chiral **OD3**), CO_2/MeOH = 90/10, Flow rate: 1.0 mL/min, 254 nm, t_r (minor) = 7.72 min, t_r (major) = 8.33 min.
^{1}H NMR (400 MHz, $CDCl_3$) δ 8.34 – 7.98 (m, 5H), 7.42 (s, 5H), 7.28 – 7.16 (m, 2H), 7.07 (s, 1H), 5.28 – 5.09 (m, 2H), 3.94 (s, 3H), 2.04 – 1.89 (m, 2H), 1.68 (t, *J* = 6.4 Hz, 2H), 1.47 – 1.38 (m, 1H), 1.36 – 1.22 (m, 7H), 1.10 (s, 6H), 0.68 – 0.55 (m, 1H).
^{13}C NMR (100 MHz, $CDCl_3$) δ 187.8/187.2 (rotamer), 170.7, 156.1/155.4 (rotamer), 154.1/153.3 (rotamer), 146.8/146.6 (rotamer), 144.0, 138.4, 131.1/130.7 (rotamer), 130.3/130.1 (rotamer), 129.6/129.2 (rotamer), 128.9/128.8 (rotamer), 128.5, 128.2/128.1 (rotamer), 126.6, 125.1,

119.5/118.8 (rotamer), 111.6, 81.8/80.5 (rotamer), 68.0/67.4 (rotamer), 47.7/47.6 (rotamer), 37.4, 36.5, 29.2, 28.4, 28.1/28.0 (rotamer), 26.8, 26.5.
HRMS (ESI-FT) calculated for $C_{33}H_{38}N_5O_4^+$ ([M]+H$^+$) = 568.2918, Found 568.2915.
$[\alpha]_D^{25}$ = –155.5 (*c* =0.82, in CH_2Cl_2).
IR (film): 1719, 1678, 1596, 1496, 1399, 1283, 1160, 1110, 967 cm^{-1}.
UPC^2 spectrum of **4q**:

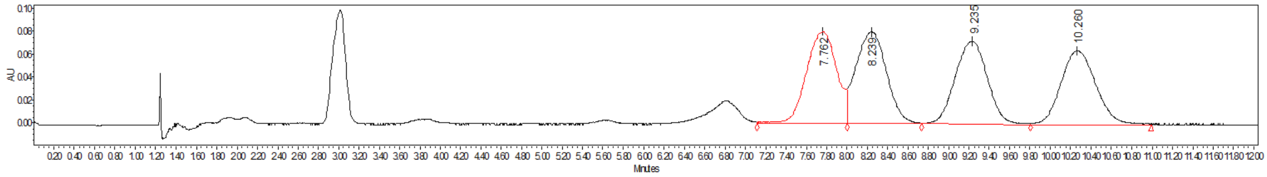

	Retention Time	Area	% Area
1	7.762	1676655	26.01
2	8.239	1680973	26.07
3	9.235	1581129	24.53
4	10.260	1508084	23.39

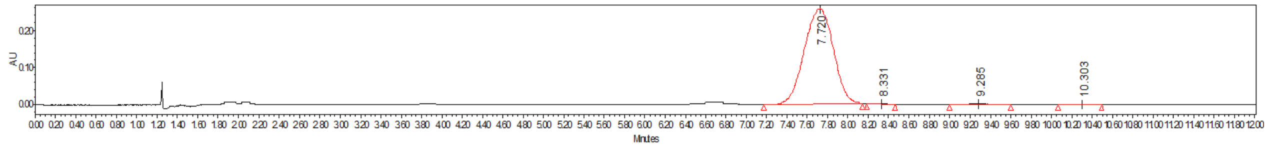

	Retention Time	Area	% Area
1	7.720	5061626	99.10
2	8.331	1463	0.03
3	9.285	36467	0.71
4	10.303	8072	0.16

Tert-butyl ((*1S,2R,E*)-2-(1-methyl-1H-imidazole-2-carbonyl)-1,4-diphenylbut-3-en-1-yl)carbamate

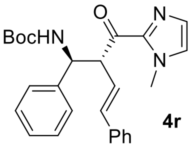

4r

Colourless oil. 73% yield (98% ee, >19:1 dr) was obtained according to the general procedure at 35 °C for 10 h.
HPLC (Chiral IA column), *i*-PrOH/*n*-Hexane = 20/80, Flow rate: 1.0 mL/min, 254 nm, t_r (minor) = 18.51 min, t_r (major) = 10.91 min.
^{1}**H NMR** (400 MHz, $CDCl_3$) δ 7.34 – 7.28 (m, 2H), 7.28 – 7.20 (m, 6H), 7.20 – 7.11 (m, 3H), 6.99 (s, 1H), 6.55 – 6.20 (m, 2H), 6.20 – 5.70 (m, 1H), 5.26 – 4.88 (m, 2H), 3.92 (s, 3H), 1.42 – 1.19 (m, 9H).
^{13}**C NMR** (100 MHz, $CDCl_3$) δ 191.8, 155.4, 143.3, 140.9, 136.9, 134.7, 129.7, 128.5, 128.4, 127.7, 127.3, 126.9, 126.5, 125.0, 79.4, 57.1, 55.2, 36.3, 28.4.
HRMS (ESI-FT) calculated for $C_{26}H_{30}N_3O_3^+$ ([M]+H$^+$) = 432.2282, Found 432.2281.
$[\alpha]_D^{21}$ = +41.5 (*c* = 0.58, in CH_2Cl_2).
IR (film): 1708, 1495, 1403, 1365, 1250, 1165, 969 cm^{-1}.
UPC^2 spectrum of **4r**:

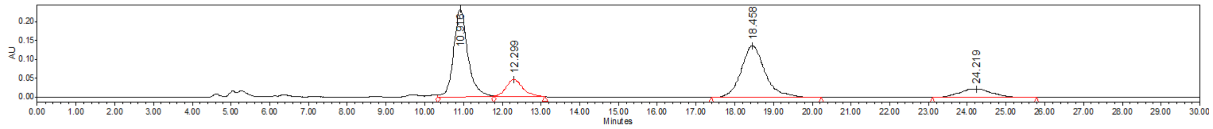

	Retention Time	Area	% Area
1	10.916	5870291	40.64
2	12.299	1362528	9.43
3	18.458	5886138	40.75
4	24.219	1325466	9.18

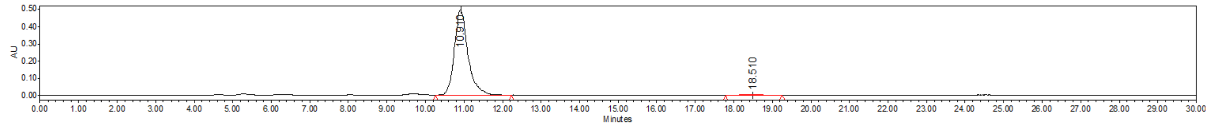

	Retention Time	Area	% Area
1	10.910	12134369	98.91
2	18.510	133165	1.09

Tert-butyl (*E*)-(1-(4-chlorophenyl)-2-(1-methyl-4,5-dihydro-1H-imidazole-2-carbonyl)-4-phenylbut-3-en-1-yl)carbamate

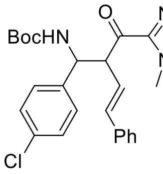

4s

Colourless oil. 77% yield (97% ee, >19:1 dr) was obtained according to the general procedure at 35 °C for 10 h.

HPLC (Chiral IA column), *i*-PrOH/*n*-Hexane = 20/80, Flow rate: 1.0 mL/min, 254 nm, t_r (minor) = 19.25 min, t_r (major) = 15.86 min.

^{1}H NMR (400 MHz, $CDCl_3$) δ 7.30 – 7.13 (m, 10H), 7.02 (s, 1H), 6.58 – 5.74 (m, 3H), 5.24 – 4.86 (m, 2H), 3.93 (s, 3H), 1.43 – 1.23 (m, 9H).

^{13}C NMR (100 MHz, $CDCl_3$) δ 191.5, 155.3, 143.1, 139.7, 136.7, 135.0, 133.0, 129.8, 128.6, 128.3, 128.0, 127.9, 126.6, 124.6, 79.7, 56.6, 54.9, 36.3, 28.4.

HRMS (ESI-FT) calculated for $C_{26}H_{29}{}^{34.9689}ClN_3O_3^+$ ([M]+H$^+$) = 466.1892, Found 466.1887.

HRMS (ESI-FT) calculated for $C_{26}H_{29}{}^{36.9659}ClN_3O_3^+$ ([M]+H$^+$) = 468.1862, Found 468.1858.

$[\alpha]_D^{26}$ = +41.5 (*c* = 0.48, in CH_2Cl_2).

IR (film): 1711, 1492, 1404, 1366, 1250, 1165, 1090, 1013 cm^{-1}.

UPC^2 spectrum of **4s**:

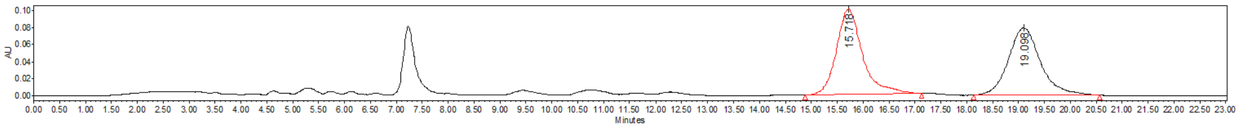

	Retention Time	Area	% Area
1	15.718	3485020	50.98
2	19.098	3351277	49.02

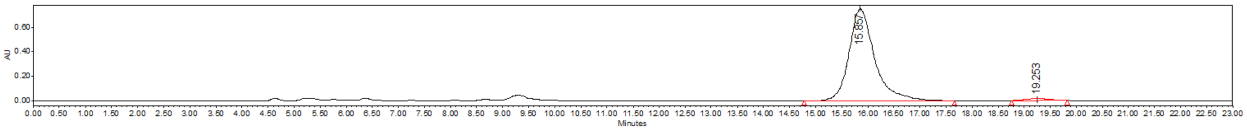

	Retention Time	Area	% Area
1	15.857	25260327	98.45
2	19.253	398941	1.55

Tert-butyl (*E*)-(2-(1-methyl-4,5-dihydro-1H-imidazole-2-carbonyl)-4-phenyl-1-(m-tolyl)but-3-en-1-yl)carbamate

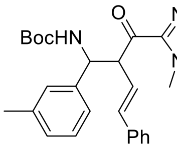

4t

Colourless oil. 70% yield (98% ee, >19:1 dr) was obtained according to the general procedure at 35 °C for 10 h.

HPLC (Chiral IA column), *i*-PrOH/ *n*-Hexane = 20/80, Flow rate: 1.0 mL/min, 254 nm, t_r (minor) = 12.94 min, t_r (major) = 8.68 min.

^{1}H NMR (400 MHz, $CDCl_3$) δ 7.29 – 7.17 (m, 5H), 7.16 – 7.07 (m, 4H), 7.03 – 6.93 (m, 2H), 6.67 – 6.20 (m, 2H), 6.13 – 5.65 (m, 1H), 5.23 – 4.88 (m, 2H), 3.92 (s, 3H), 2.28 (s, 3H), 1.42 – 1.23 (m, 9H).

^{13}C NMR (100 MHz, $CDCl_3$) δ 191.9, 155.4, 143.3, 140.8, 137.9, 137.0, 134.6, 129.7, 128.5, 128.3, 128.1, 127.7, 127.7, 126.5, 125.1, 123.9, 79.4, 57.0, 55.2, 36.3, 28.5, 21.6.

HRMS (ESI-FT) calculated for $C_{27}H_{32}N_3O_3^+$ ([M]+H$^+$) = 446.2438, Found 446.2431.

$[\alpha]_D^{21}$ = +38.3 (*c* = 0.40, in CH_2Cl_2).

IR (film): 1711, 1672, 1493, 1403, 1365, 1250, 1164, 967 cm^{-1}.

UPC^2 spectrum of **4t**:

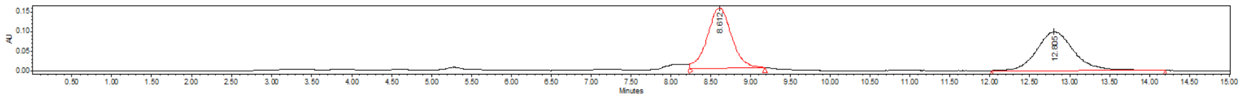

	Retention Time	Area	% Area
1	8.612	3241273	49.93
2	12.805	3250574	50.07

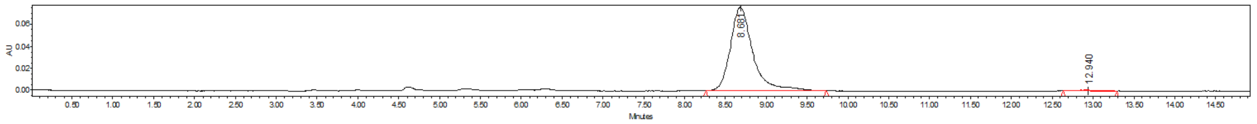

	Retention Time	Area	% Area
1	8.681	1474753	98.95
2	12.940	15581	1.05

Tert-butyl (E)-(1-(3-bromophenyl)-2-(1-methyl-4,5-dihydro-1H-imidazole-2-carbonyl)-4-phenylbut-3-en-1-yl)carbamate

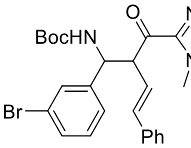

4u

Colourless oil. 57% yield (95% ee, >19:1 dr) was obtained according to the general procedure at 35 °C for 8 h.
HPLC (Chiral IA column), *i*-PrOH/*n*-Hexane = 20/80, Flow rate: 1.0 mL/min, 254 nm, t_r (minor) = 12.97 min, t_r (major) = 10.53 min.
^{1}H NMR (400 MHz, $CDCl_3$) δ 7.47 (s, 1H), 7.31 – 7.22 (m, 6H), 7.22 – 7.09 (m, 3H), 7.02 (s, 1H), 6.52 (d, *J* = 15.6 Hz, 1H), 6.34 – 5.70 (m, 2H), 5.23 – 4.83 (m, 2H), 3.94 (s, 3H), 1.43 – 1.22 (m, 9H).
^{13}C NMR (100 MHz, $CDCl_3$) δ 191.4, 155.3, 143.4, 143.1, 136.7, 135.1, 130.5, 130.0, 129.8, 128.6, 128.0, 127.9, 126.6, 125.8, 124.5, 122.6, 79.8, 56.7, 54.9, 36.3, 28.4.
HRMS (ESI-FT) calculated for $C_{26}H_{29}{}^{78.9183}BrN_3O_3^+$ ([M]+H$^+$) = 510.1387, Found 510.1381.
HRMS (ESI-FT) calculated for $C_{26}H_{29}{}^{80.9163}BrN_3O_3^+$ ([M]+H$^+$) = 512.1366, Found 512.1364.
$[\alpha]_D^{21}$ = +40.2 (*c* = 0.50, in CH_2Cl_2).
IR (film): 1712, 1494, 1404, 1366, 1249, 1164, 968 cm^{-1}.
UPC^2 spectrum of **4u**:

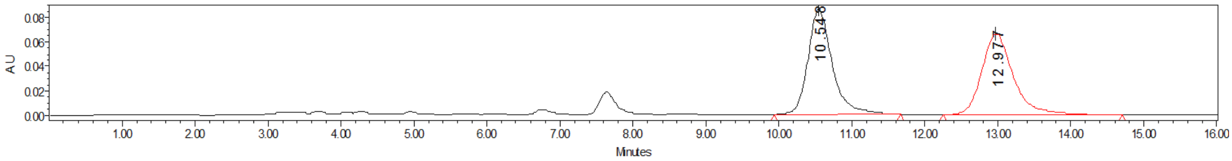

	Retention Time	Area	% Area
1	10.548	1989637	50.21
2	12.977	1973330	49.79

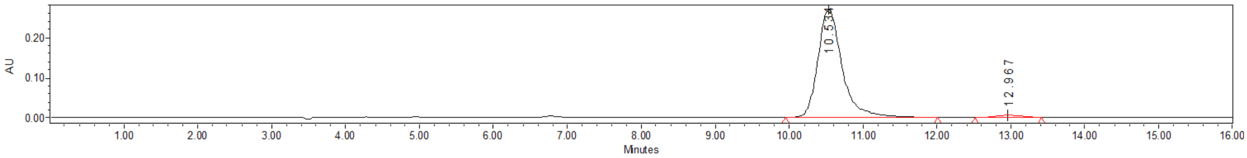

	Retention Time	Area	% Area
1	10.534	6178060	97.41
2	12.967	164584	2.59

Tert-butyl (*E*)-(2-(1-methyl-4,5-dihydro-1H-imidazole-2-carbonyl)-4-phenyl-1-(o-tolyl)but-3-en-1-yl)carbamate

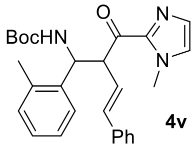

4v

Colourless oil. 55% yield (97% ee, >19:1 dr) was obtained according to the general procedure at 35 °C for 8 h.
HPLC (Chiral IA column), *i*-PrOH/*n*-Hexane = 20/80, Flow rate: 1.0 mL/min, 254 nm, t_r (minor) = 12.72 min, t_r (major) = 7.25 min.
^{1}H NMR (400 MHz, $CDCl_3$) δ 7.72 – 7.32 (m, 11H), 6.88 – 6.56 (m, 2H), 6.55 – 6.10 (m, 1H), 5.82 – 5.30 (m, 2H), 4.31 (s, 3H), 2.83 (s, 3H), 1.77 – 1.45 (m, 9H).
^{13}C NMR (100 MHz, $CDCl_3$) δ 192.0, 155.2, 143.5, 139.4, 136.9, 135.7, 134.4, 130.6, 129.7, 128.5, 127.7, 127.1, 126.5, 126.0, 125.9, 124.9, 79.4, 54.6, 53.6, 36.3, 28.4, 19.7.
HRMS (ESI-FT) calculated for $C_{27}H_{32}N_3O_3^+$ ([M]+H$^+$) = 446.2438, Found 446.2432.
$[\alpha]_D^{21}$ = +5.6 (*c* = 0.36, in CH_2Cl_2).
IR (film): 1711, 1672, 1493, 1404, 1365, 1287, 1249, 1167, 967 cm^{-1}.
UPC^2 spectrum of **4v**:

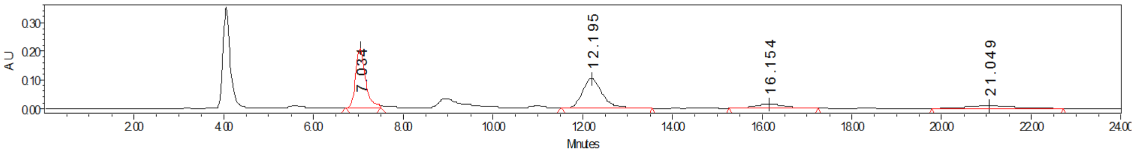

	Retention Time	Area	% Area
1	7.034	3224641	41.88
2	12.195	3198149	41.54
3	16.154	642517	8.34
4	21.049	634465	8.24

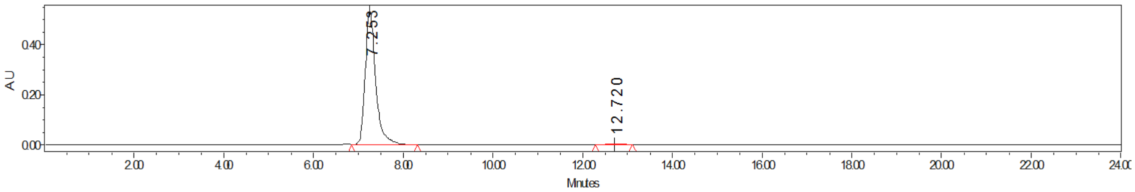

	Retention Time	Area	% Area
1	7.253	8706821	98.42
2	12.720	139487	1.58

Tert-butyl (*E*)-(2-(1-methyl-1H-imidazole-2-carbonyl)-1-phenyl-4-(o-tolyl)but-3-en-1-yl)carbamate

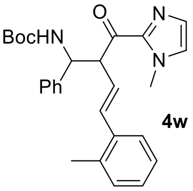

4w

Colourless oil. 67% yield (96% ee, >19:1 dr) was obtained according to the general procedure at 35 °C for 8 h.
HPLC (Chiral IA column), *i*-PrOH/*n*-Hexane = 20/80, Flow rate: 1.0 mL/min, 254 nm, t_r (minor) = 14.07 min, t_r (major) = 8.34 min.
^{1}**H NMR** (400 MHz, $CDCl_3$) δ 7.36 – 7.20 (m, 5H), 7.19 – 7.12 (m, 2H), 7.11 – 6.97 (m, 4H), 6.75 –6.35 (m, 1H), 6.23 – 5.60 (m, 2H), 5.26 – 4.87 (m, 2H), 3.93 (s, 3H), 2.16 – 1.92 (m, 3H), 1.41 – 1.20 (m, 9H).
^{13}**C NMR** (100 MHz, $CDCl_3$) δ 191.7, 155.3, 143.3, 141.0, 136.1, 135.5, 133.1, 130.1, 129.6, 128.4, 127.7, 127.6, 127.3, 127.0, 126.2, 126.0, 125.9, 79.4, 57.1, 55.7, 36.3, 28.4, 19.7.
HRMS (ESI-FT) calculated for $C_{27}H_{32}N_3O_3^+$ ($[M]+H^+$) = 446.2438, Found 446.2432.
$[\alpha]_D^{21}$ = +25.3 (*c* = 1.12, in CH_2Cl_2).
IR (film): 1710, 1671, 1493, 1403, 1365, 1287, 1249, 1167, 968 cm^{-1}.
UPC^2 spectrum of **4w**:

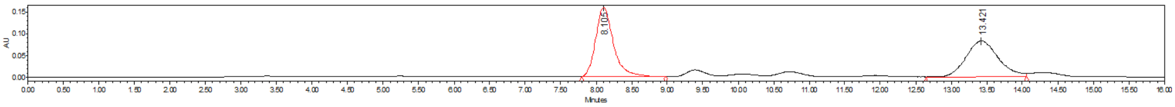

	Retention Time	Area	% Area
1	8.105	2721877	50.36
2	13.421	2682541	49.64

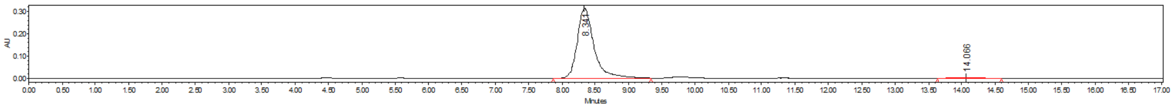

	Retention Time	Area	% Area
1	8.341	5421399	98.10
2	14.066	104996	1.90

Tert-butyl (*E*)-(4-(3-methoxyphenyl)-2-(1-methyl-1H-imidazole-2-carbonyl)-1-phenylbut-3-en-1-yl)carbamate

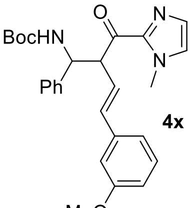

4x

Colourless oil. 62% yield (98% ee, >19:1 dr) was obtained according to the general procedure at 35 °C for 8 h.
HPLC (Chiral IA column), *i*-PrOH/ *n*-Hexane = 20/80, Flow rate: 1.0 mL/min, 254 nm, t_r (minor) = 15.57 min, t_r (major) = 9.41 min.
^{1}**H NMR** (400 MHz, $CDCl_3$) δ 7.33 – 7.21 (m, 4H), 7.18 – 6.94 (m, 7H), 6.55 – 6.20 (m, 2H), 6.18 – 5.68 (m, 1H), 5.26 – 4.84 (m, 2H), 3.92 (s, 3H), 2.28 (s, 3H), 1.44 – 1.20 (m, 9H).
^{13}**C NMR** (100 MHz, $CDCl_3$) δ 191.9, 155.4, 143.3, 141.0, 138.1, 136.9, 134.8, 129.7, 128.5, 128.4, 127.7, 127.3, 127.2, 126.9, 124.7, 123.8, 79.4, 57.1, 55.2, 36.3, 28.5, 21.4.
HRMS (ESI-FT) calculated for $C_{27}H_{32}N_3O_4^+$ ($[M]+H^+$) = 462.2387, Found 462.2383.
$[\alpha]_D^{22}$ = +44.8 (*c* = 0.40, in CH_2Cl_2).
IR (film): 1711, 1671, 1493, 1403, 1365, 1287, 1249, 1166, 966 cm^{-1}.
UPC^2 spectrum of **4x**:

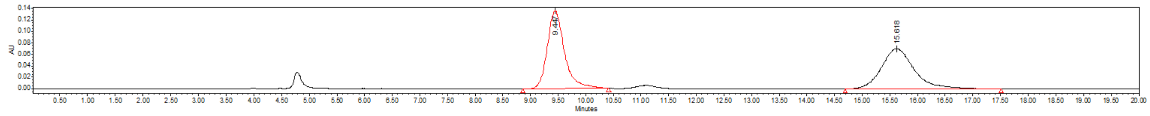

	Retention Time	Area	% Area
1	9.447	2874731	50.38
2	15.618	2831928	49.62

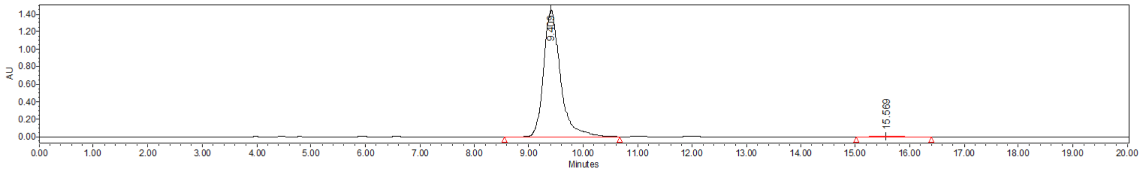

	Retention Time	Area	% Area
1	9.409	30877568	99.03
2	15.569	303400	0.97

Tert-butyl (*E*)-(4-(4-bromophenyl)-2-(1-methyl-1H-imidazole-2-carbonyl)-1-phenylbut-3-en-1-yl)carbamate

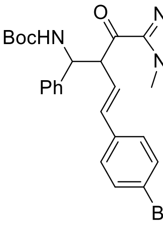

4y

Colourless oil. 72% yield (97% ee, >19:1 dr) was obtained according to the general procedure at 35 °C for 8 h.
HPLC (Chiral IA column), *i*-PrOH/*n*-Hexane = 20/80, Flow rate: 1.0 mL/min, 254 nm, t_r (minor) = 25.93 min, t_r (major) = 14.49 min.
^{1}H NMR (400 MHz, CDCl$_3$) δ 7.38 – 7.32 (m, 2H), 7.32 – 7.20 (m, 4H), 7.19 – 7.07 (m, 4H), 7.04 – 6.98 (m, 1H), 6.48 – 6.19 (m, 2H), 6.17 – 5.70 (m, 1H), 5.25 – 4.86 (m, 2H), 3.93 (s, 3H), 1.39 – 1.21 (m, 9H).
^{13}C NMR (100 MHz, CDCl$_3$) δ 191.7, 155.4, 143.2, 140.8, 135.9, 133.4, 131.6, 129.8, 128.4, 128.1, 127.8, 127.4, 126.8, 126.0, 121.5, 79.5, 57.1, 55.1, 36.3, 28.4.
HRMS (ESI-FT) calculated for $C_{26}H_{29}{}^{78.9183}BrN_3O_3^+$ ([M]+H$^+$) = 510.1387, Found 510.1382.
HRMS (ESI-FT) calculated for $C_{26}H_{29}{}^{80.9163}BrN_3O_3^+$ ([M]+H$^+$) = 512.1366, Found 512.1362.
$[\alpha]_D^{22}$ = +31.8 (*c* = 0.44, in CH$_2$Cl$_2$).
IR (film): 1711, 1671, 1488, 1403, 1365, 1248, 1166, 1073, 1008 cm^{-1}.
UPC^2 spectrum of **4y**:

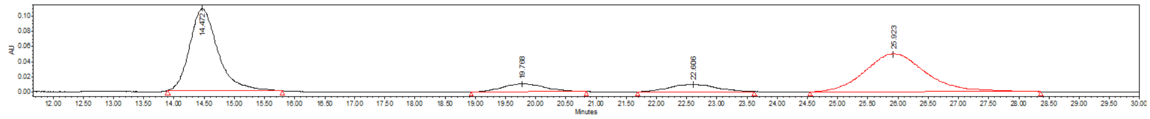

	Retention Time	Area	% Area
1	14.472	3472616	43.80
2	19.768	497319	6.27
3	22.606	493384	6.22
4	25.923	3465895	43.71

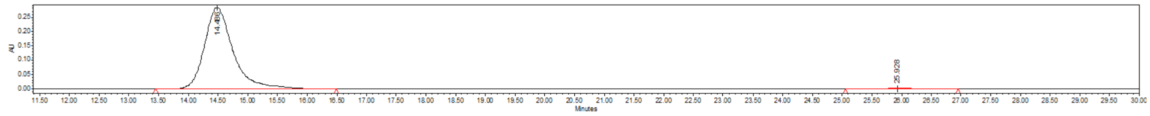

	Retention Time	Area	% Area
1	14.486	9455839	98.47
2	25.928	147397	1.53

Tert-butyl (2-(cyclohexylidenemethyl)-3-(1-methyl-1H-imidazol-2-yl)-3-oxo-1-phenylpropyl)carbamate

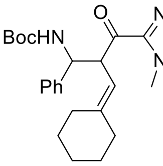

4z

Colourless oil. 81% yield (85% ee, >19:1 dr) was obtained according to the general procedure at 35 °C for 10 h.
HPLC (Chiral IA column), *i*-PrOH/*n*-Hexane = 15/85, Flow rate: 1.0 mL/min, 254 nm, t_r (minor) = 8.47 min, t_r (major) = 7.74 min.

¹H NMR (400 MHz, $CDCl_3$) δ 7.30 – 7.21 (m, 4H), 7.19 – 7.10 (m, 2H), 7.00 (s, 1H), 6.00 – 5.53 (m, 1H), 5.24 – 4.64 (m, 3H), 3.93 (s, 3H), 2.18 – 1.93 (m, 3H), 1.49 – 1.13 (m, 15H), 1.10 – 0.90 (m, 1H).
¹³C NMR (100 MHz, $CDCl_3$) δ 192.8, 155.3, 145.2, 143.6, 141.6, 129.4, 129.4, 128.2, 127.5, 127.0, 116.5, 79.2, 57.7, 50.5, 37.4, 36.3, 29.5, 28.4, 28.3, 27.4, 26.6.
HRMS (ESI-FT) calculated for $C_{25}H_{34}N_3O_3^+$ ($[M]+H^+$) = 424.2595, Found 424.2587.
$[\alpha]_D^{26}$ = +71.4 (*c* = 0.76, in CH_2Cl_2).
IR (film): 1711, 1677, 1494, 1451, 1404, 1365, 1250, 1168, 990 cm^{-1}.
UPC² spectrum of **4z**:

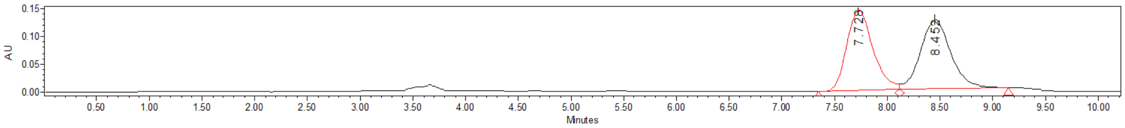

	Retention Time	Area	% Area
1	7.728	2518025	50.23
2	8.452	2494813	49.77

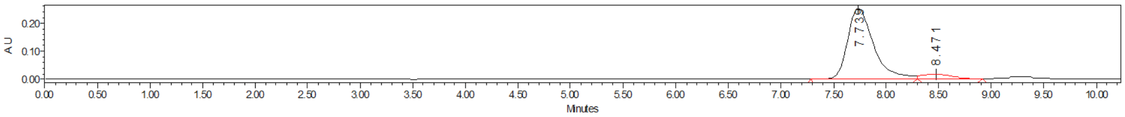

	Retention Time	Area	% Area
1	7.739	4327774	92.45
2	8.471	353658	7.55

(*R*)-1-(1-Methyl-1H-imidazol-2-yl)-4-phenyl-3-(phenylthio)butan-1-one

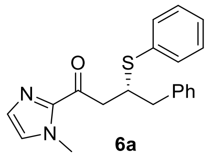

6a

Pale yellow oil. 89% yield and 90% ee was obtained according to the general procedure at 25 °C for 17 h.
UPC² (Chiral **OJ-3**), CO_2/MeOH = 90/10, Flow rate: 1.0 mL/min, 254 nm, t_r (minor) = 8.83 min, t_r (major) = 9.58 min.
¹H NMR (400 MHz, $CDCl_3$) δ 7.47 – 7.40 (m, 2H), 7.29 – 7.14 (m, 8H), 7.11 (d, *J* = 0.8 Hz, 1H), 6.97 (s, 1H), 4.14 – 4.03 (m, 1H), 3.87 (s, 3H), 3.49 (dd, *J* = 16.8, 7.6 Hz, 1H), 3.30 (dd, *J* = 16.8, 6.0 Hz, 1H), 3.06 (dd, *J* = 14.0, 6.4 Hz, 1H), 2.86 (dd, *J* = 13.6, 8.4 Hz, 1H).
¹³C NMR (100 MHz, $CDCl_3$) δ 190.5, 143.1, 138.8, 134.6, 132.7, 129.5, 129.2, 129.0, 128.4, 127.3, 127.1, 126.6, 45.7, 43.4, 41.8, 36.3.
HRMS (ESI-FT) calculated for $C_{20}H_{21}N_2OS^+$ ($[M]+H^+$) = 337.1369, Found 337.1369.
$[\alpha]_D^{25}$ = +4.5 (*c* = 0.69, in CH_2Cl_2).
IR (film): 1671, 1405, 1286, 1154, 1081, 1024, 985, 914 cm^{-1}.
UPC² spectrum of **6a**:

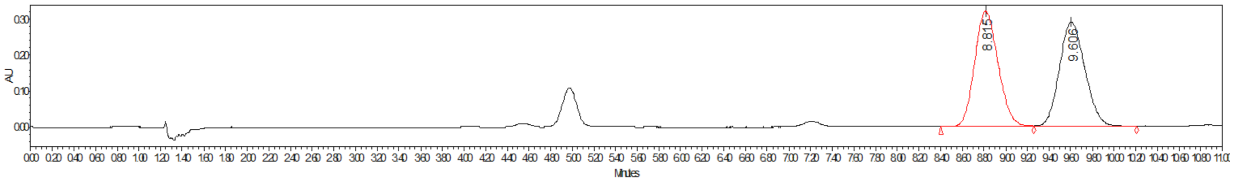

	Retention Time	Area	% Area
1	8.815	4853450	50.33
2	9.606	4789197	49.67

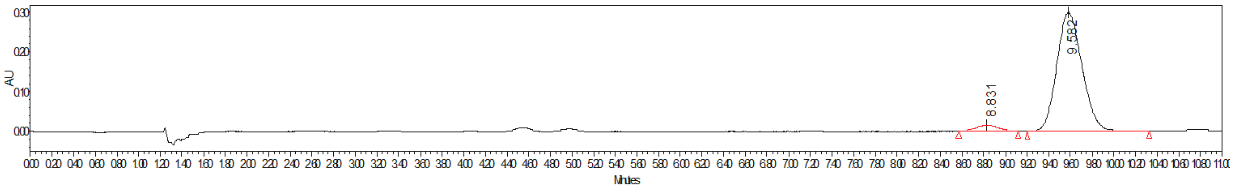

	Retention Time	Area	% Area
1	8.831	204749	4.06
2	9.582	4837895	95.94

3-((4-Bromophenyl)thio)-1-(1-methyl-1H-imidazol-2-yl)-4-phenylbutan-1-one

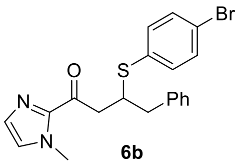

6b

Pale yellow oil. 94% yield and 80% ee was obtained according to the general procedure at 25 °C for 17 h.
UPC² (Chiral **OJ-3**), CO_2/MeOH = 90/10, Flow rate: 1.0 mL/min, 254 nm, t_r (minor) = 9.89 min, t_r (major) = 12.84 min.
^{1}H NMR (400 MHz, $CDCl_3$) δ 7.38 – 7.33 (m, 2H), 7.28 – 7.21 (m, 4H), 7.20 – 7.14 (m, 3H), 7.11 (d, *J* = 0.8 Hz, 1H), 6.98 (s, 1H), 4.10 – 4.01 (m, 1H), 3.86 (s, 3H), 3.50 (dd, *J* = 16.8, 8.0 Hz, 1H), 3.27 (dd, *J* = 16.8, 6.0 Hz, 1H), 3.02 (dd, *J* = 14.0, 6.8 Hz, 1H), 2.87 (dd, *J* = 13.8, 8.0 Hz, 1H).
^{13}C NMR (100 MHz, $CDCl_3$) δ 190.2, 143.0, 138.5, 134.2, 133.8, 131.9, 129.4, 129.2, 128.4, 127.2, 126.7, 121.4, 46.1, 43.5, 41.7, 36.2.
HRMS (ESI-FT) calculated for $C_{20}H_{20}{}^{78.9183}BrN_2OS^+$ ([M]+H$^+$) = 415.0474, Found 415.0473.
HRMS (ESI-FT) calculated for $C_{20}H_{20}{}^{80.9163}BrN_2OS^+$ ([M]+H$^+$) = 417.0454, Found 417.0450.
$[\alpha]^{25}_{\lambda}$ = +21.2 (*c* = 0.82, in CH_2Cl_2, λ = 436 nm).
IR (film): 1671, 1470, 1406, 1286, 1087, 1005, 913 cm^{-1}.
UPC² spectrum of **6b**:

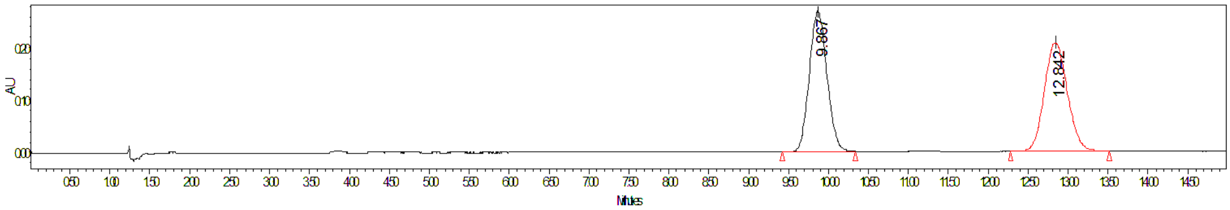

	Retention Time	Area	% Area
1	9.867	4141198	49.93
2	12.842	4153451	50.07

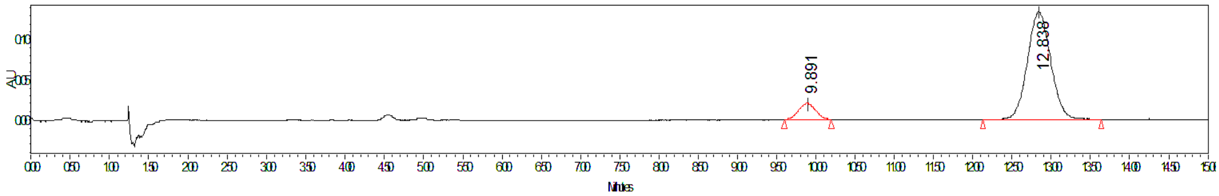

	Retention Time	Area	% Area
1	9.891	308980	10.08
2	12.838	2754932	89.92

3-((4-Chlorophenyl)thio)-1-(1-methyl-1H-imidazol-2-yl)-4-phenylbutan-1-one

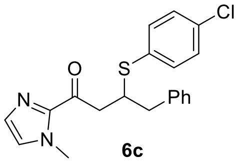

6c

Pale yellow oil. 90% yield and 88% ee was obtained according to the general procedure at 25 °C for 17 h.
UPC² (Chiral **OJ-3**), CO_2/MeOH = 90/10, Flow rate: 1.0 mL/min, 254 nm, t_r (minor) = 7.92 min, t_r (major) = 10.47 min.
^{1}H NMR (400 MHz, $CDCl_3$) δ 7.36 – 7.31 (m, 2H), 7.27 – 7.14 (m, 7H), 7.11 (d, *J* = 0.4 Hz, 1H), 6.98 (s, 1H), 4.08 – 4.01 (m, 1H), 3.87 (s, 3H), 3.50 (dd, *J* = 16.8, 8.0 Hz, 1H), 3.27 (dd, *J* = 16.8, 6.0 Hz, 1H), 3.02 (dd, *J* = 14.0, 6.4 Hz, 1H), 2.87 (dd, *J* = 14.0, 8.4 Hz, 1H).
^{13}C NMR (100 MHz, $CDCl_3$) δ 190.3, 143.0, 138.6, 134.1, 133.4, 133.1, 129.5, 129.3, 129.0, 128.4, 127.2, 126.7, 46.2, 43.5, 41.7, 36.2.
HRMS (ESI-FT) calculated for $C_{20}H_{20}{}^{34.9689}ClN_2OS^+$ ([M]+H$^+$) = 371.0979, Found 371.0974.
HRMS (ESI-FT) calculated for $C_{26}H_{29}{}^{36.9659}ClN_3O_3^+$ ([M]+H$^+$) = 373.0950, Found 373.0941.
$[\alpha]^{23}_{\lambda}$ = +11.4 (*c* = 0.92, in CH_2Cl_2, λ = 436 nm).
IR (film): 1671, 1474, 1407, 1286, 1154, 1092, 1010, 914 cm^{-1}.
UPC² spectrum of **6c**:

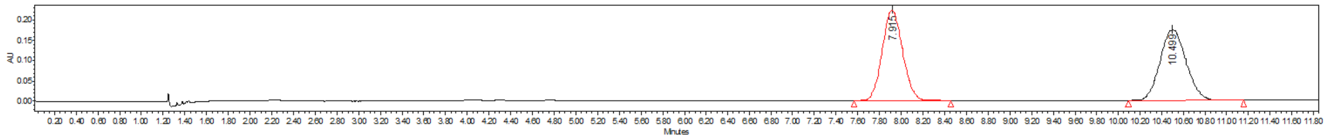

	Retention Time	Area	% Area
1	7.915	2928117	49.93
2	10.499	2936724	50.07

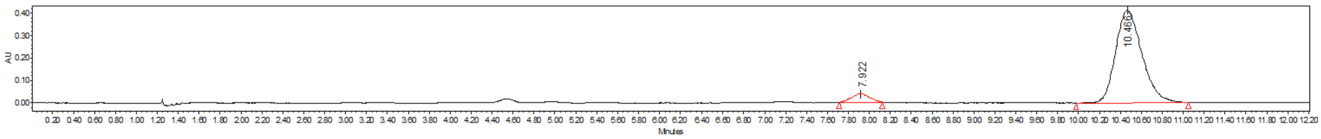

	Retention Time	Area	% Area
1	7.922	465072	6.05
2	10.466	7228424	93.95

1-(1-Methyl-1H-imidazol-2-yl)-4-phenyl-3-(p-tolylthio)butan-1-one

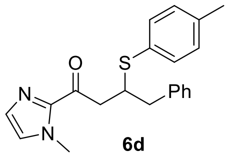

6d

Pale yellow oil. 64% yield and 88% ee was obtained according to the general procedure at 25 °C for 17 h.
UPC^2 (Chiral **OJ-3**), CO_2/MeOH = 90/10, Flow rate: 1.0 mL/min, 254 nm, t_r (minor) = 8.07 min, t_r (major) = 10.03 min.
^{1}H NMR (400 MHz, $CDCl_3$) δ 7.34 (d, *J* = 8.4 Hz, 2H), 7.26 – 7.15 (m, 5H), 7.11 (d, *J* = 0.8, 1H), 7.08 (d, *J* = 8.0, 2H), 6.97 (s, 1H), 4.04 – 3.96 (m, 1H), 3.87 (s, 3H), 3.48 (dd, *J* = 16.8, 8.0 Hz, 1H), 3.25 (dd, *J* = 16.8, 6.0 Hz, 1H), 3.05 (dd, *J* = 13.6, 6.0 Hz, 1H), 2.82 (dd, *J* = 13.6, 8.4 Hz, 1H), 2.31 (s, 3H).
^{13}C NMR (100 MHz, $CDCl_3$) δ 190.6, 143.2, 139.0, 137.6, 133.6, 130.5, 129.7, 129.5, 129.2, 128.4, 127.1, 126.6, 46.0, 43.3, 41.7, 36.2, 21.2.
HRMS (ESI-FT) calculated for $C_{21}H_{23}N_2OS^+$ ([M]+H$^+$) = 351.1526, Found 351.1526.
$[\alpha]^{26}_{\lambda}$ = +9.4 (*c* = 0.45, in CH_2Cl_2, λ = 436 nm).
IR (film): 1673, 1492, 1408, 1288, 1155, 1092, 987 cm^{-1}.
UPC^2 spectrum of **6d**:

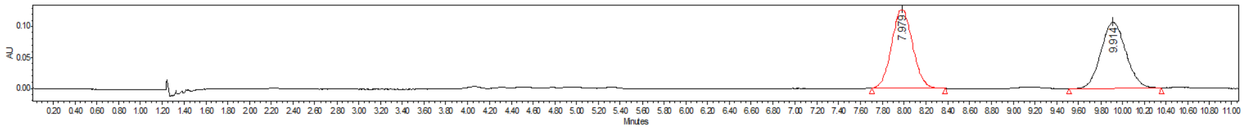

	Retention Time	Area	% Area
1	7.979	1686148	50.03
2	9.914	1684103	49.97

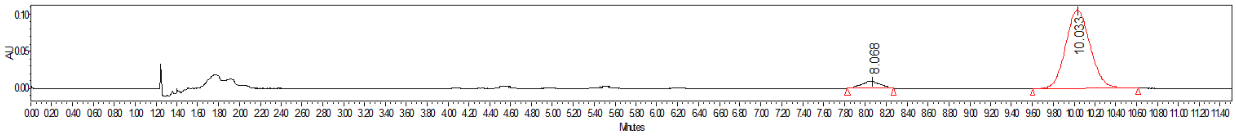

	Retention Time	Area	% Area
1	8.068	105812	5.85
2	10.033	1703752	94.15

1-(1-Methyl-1H-imidazol-2-yl)-3-(naphthalen-2-ylthio)-4-phenylbutan-1-one

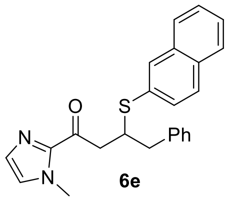

6e

Pale yellow oil. 95% yield and 83% ee was obtained according to the general procedure at 25 °C for 17 h.
UPC^2 (Chiral **OJ-3**), CO_2/MeOH = 80/20, Flow rate: 1.0 mL/min, 254 nm, t_r (minor) = 8.25 min, t_r (major) = 8.72 min.
^{1}H NMR (400 MHz, $CDCl_3$) δ 7.88 (s, 1H), 7.78 – 7.66 (m, 3H), 7.50 (dd, *J* = 8.4, 1.6 Hz, 1H), 7.47 – 7.39 (m, 2H), 7.26 – 7.19 (m, 4H), 7.18 – 7.13 (m, 1H), 7.09 (d, *J* = 0.4 Hz, 1H), 6.88 (s, 1H), 4.27 – 4.16 (m, 1H), 3.74 (s, 3H), 3.57 (dd, *J* = 16.8, 8.0 Hz, 1H), 3.31 (dd, *J* = 16.8, 6.0 Hz, 1H), 3.10 (dd, *J* = 13.6, 6.0 Hz, 1H), 2.91 (dd, *J* = 14.0, 8.4 Hz, 1H).
^{13}C NMR (100 MHz, $CDCl_3$) δ 190.4, 143.0, 138.7, 133.7, 132.4, 132.0, 131.4, 130.0, 129.5, 129.1, 128.4, 127.7, 127.5, 127.1, 126.6, 126.4, 126.2, 45.8, 43.6, 41.7, 36.0.
HRMS (ESI-FT) calculated for $C_{24}H_{23}N_2OS^+$ ([M]+H$^+$) = 387.1526, Found 387.1530.
$[\alpha]^{25}_{\lambda}$ = +22.8 (*c* = 0.79, in CH_2Cl_2, λ = 436 nm).
IR (film): 1670, 1496, 1405, 1285, 1154, 1076, 985 cm^{-1}.
UPC^2 spectrum of **6e**:

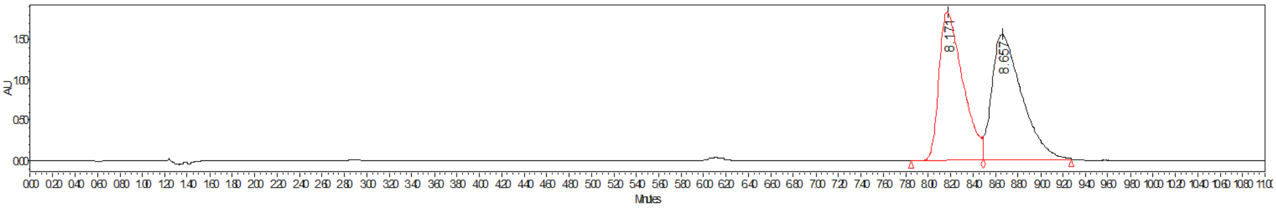

	Retention Time	Area	% Area
1	8.171	27157391	48.75
2	8.657	28548634	51.25

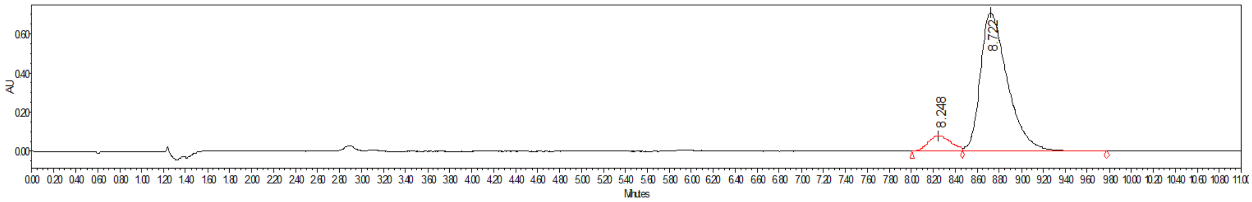

	Retention Time	Area	% Area
1	8.248	1147467	8.60
2	8.722	12190724	91.40

3-(Benzylthio)-1-(1-methyl-1H-imidazol-2-yl)-4-phenylbutan-1-one

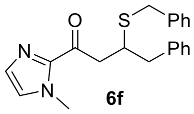

6f

Pale yellow oil. 60% yield and 78% ee was obtained according to the general procedure at 25 °C for 17 h.
UPC^2 (Chiral **IA-3**), CO_2/MeOH = 90/10, Flow rate: 1.0 mL/min, 254 nm, t_r (minor) = 10.24 min, t_r (major) = 9.52 min.
^{1}H NMR (400 MHz, $CDCl_3$) δ 7.28 – 7.17 (m, 8H), 7.16 – 7.12 (m, 2H), 7.11 (s, 1H), 6.99 (s, 1H), 3.92 (s, 3H), 3.75 – 3.64 (m, 2H), 3.56 – 3.45 (m, 2H), 3.24 (dd, *J* = 19.2, 8.4 Hz, 1H), 2.95 (dd, *J* = 13.6, 6.4 Hz, 1H), 2.87 (dd, *J* = 14.0, 7.6 Hz, 1H).
^{13}C NMR (100 MHz, $CDCl_3$) δ 190.6, 143.2, 139.2, 138.3, 129.5, 129.2, 129.1, 128.5, 128.4, 127.1, 127.0, 126.5, 43.7, 42.5, 42.5, 36.3, 36.0.
HRMS (ESI-FT) calculated for $C_{21}H_{23}N_2OS^+$ ([M]+H$^+$) = 351.1526, Found 351.1522.
$[\alpha]^{25}_{\lambda}$ = +16.5 (*c* = 0.31, in CH_2Cl_2, λ = 436 nm).
IR (film): 1672, 1453, 1407, 1286, 1154, 986, 914 cm^{-1}.
UPC^2 spectrum of **6f**:

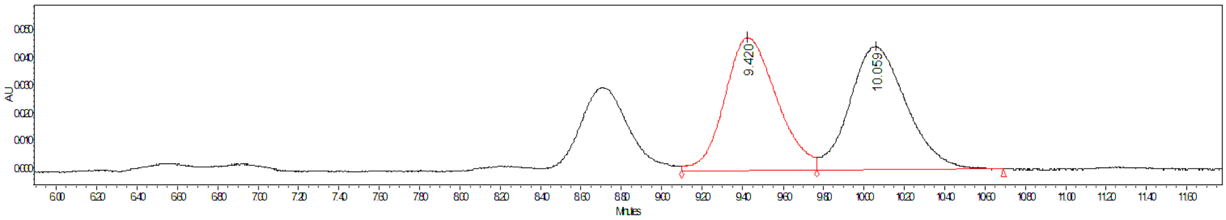

	Retention Time	Area	% Area
1	9.420	860130	49.67
2	10.059	871722	50.33

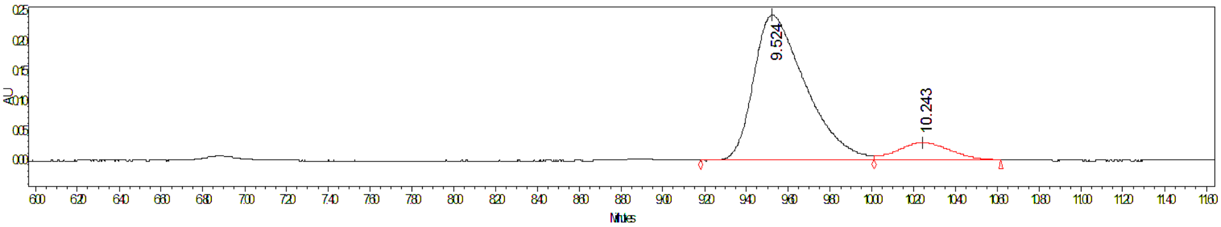

	Retention Time	Area	% Area
1	9.524	4203350	89.00
2	10.243	519698	11.00

3-(Butylthio)-1-(1-methyl-1H-imidazol-2-yl)-4-phenylbutan-1-one

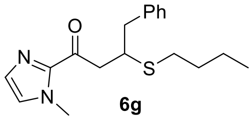

6g

Pale yellow oil. 95% yield and 70% ee was obtained according to the general procedure at 25 °C for 17 h.
HPLC (Chiral IB column), *i*-PrOH/*n*-Hexane = 5/95, Flow rate: 1.0 mL/min, 254 nm, t_r (minor) = 9.89 min, t_r (major) = 9.08 min.
^{1}H NMR (400 MHz, $CDCl_3$) δ 7.29 – 7.21 (m, 4H), 7.21 – 7.15 (m, 1H), 7.11 (d, *J* = 0.4 Hz, 1H), 6.99 (s, 1H), 3.93 (s, 3H), 3.62 – 3.46 (m, 2H), 3.24 (dd, *J* = 16.0, 5.2 Hz, 1H), 3.03 (dd, *J* = 13.6, 6.0 Hz, 1H), 2.90 (dd, *J* = 14.0, 8.0 Hz, 1H), 2.55 – 2.46 (m, 2H), 1.54 – 1.44 (m, 2H), 1.38 – 1.27 (m, 2H), 0.86 (t, *J* = 7.2 Hz, 3H).
^{13}C NMR (100 MHz, $CDCl_3$) δ 190.8, 143.3, 139.3, 129.5, 129.2, 128.4, 127.1, 126.5, 43.9, 42.5, 36.3, 31.8, 30.7, 22.1, 13.8.
HRMS (ESI-FT) calculated for $C_{18}H_{25}N_2OS^+$ ([M]+H^+) = 317.1682, Found 317.1677.
$[\alpha]^{25}_{\lambda}$ = –40.2 (*c* = 0.51, in CH_2Cl_2, λ = 436 nm).
IR (film): 2926, 1672, 1457, 1285, 1154, 986, 913 cm^{-1}.
Chiral HPLC spectrum of **6g**:

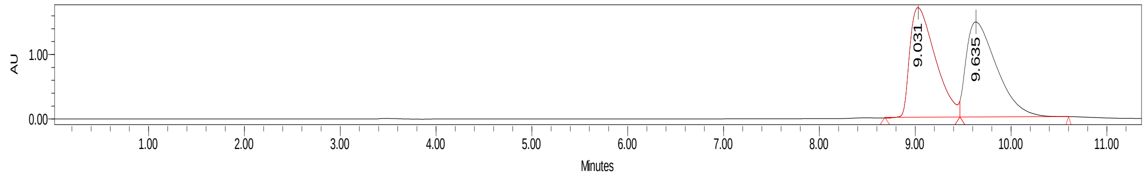

	Retention Time	Area	% Area
1	9.031	31583873	49.26
2	9.635	32536627	50.74

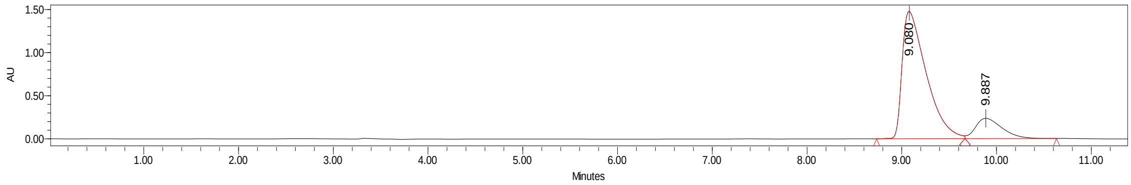

	Retention Time	Area	% Area
1	9.080	25995313	85.05
2	9.887	4569899	14.95

3-(Cyclohexylthio)-1-(1-methyl-1H-imidazol-2-yl)-4-phenylbutan-1-one

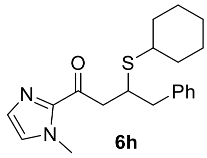

6h

Pale yellow oil. 39% yield and 85% ee was obtained according to the general procedure at 25 °C for 17 h.
UPC^2 (Chiral **IA-3**), CO_2/MeOH = 95/5, Flow rate: 1.0 mL/min, 254 nm, t_r (minor) = 13.94 min, t_r (major) = 12.91 min.
^{1}H NMR (400 MHz, $CDCl_3$) δ 7.28 – 7.21 (m, 4H), 7.21 – 7.15 (m, 1H), 7.11 (s, 1H), 6.99 (s, 1H), 3.93 (s, 3H), 3.68 – 3.55 (m, 1H), 3.46 (dd, *J* = 16.8, 8.0 Hz, 1H), 3.25 (dd, *J* = 16.8, 6.0 Hz, 1H), 3.02 (dd, *J* = 13.6, 6.8 Hz, 1H), 2.91 (dd, *J* = 14.0, 8.4 Hz, 1H), 2.63 – 2.50 (m, 1H), 1.96 – 1.80 (m, 2H), 1.75 – 1.65 (m, 2H), 1.49 – 1.58 (m, 1H), 1.31 – 1.14 (m, 5H).
^{13}C NMR (100 MHz, $CDCl_3$) δ 190.9, 143.3, 139.5, 129.6, 129.2, 128.3, 127.0, 126.5, 44.4, 43.6, 43.4, 41.0, 36.3, 34.1, 34.0, 26.2, 25.9.
HRMS (ESI-FT) calculated for $C_{20}H_{27}N_2OS^+$ ([M]+H^+) = 343.1839, Found 343.1836.
$[\alpha]^{25}_{\lambda}$ = –26.7 (*c* = 0.21, in CH_2Cl_2, λ = 436 nm).
IR (film): 2927, 2851, 1674, 1449, 1408, 1284, 988, 914 cm^{-1}.
UPC^2 spectrum of **6h**:

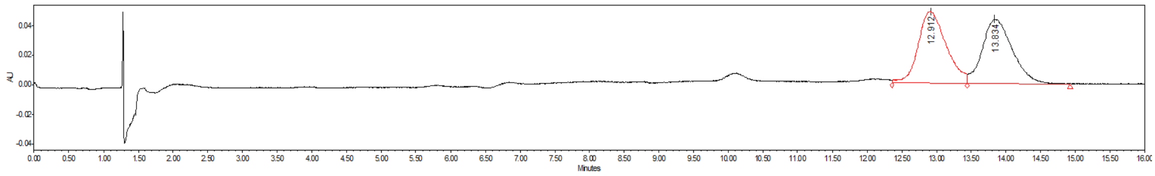

	Retention Time	Area	% Area
1	12.912	1333714	50.77
2	13.834	1293156	49.23

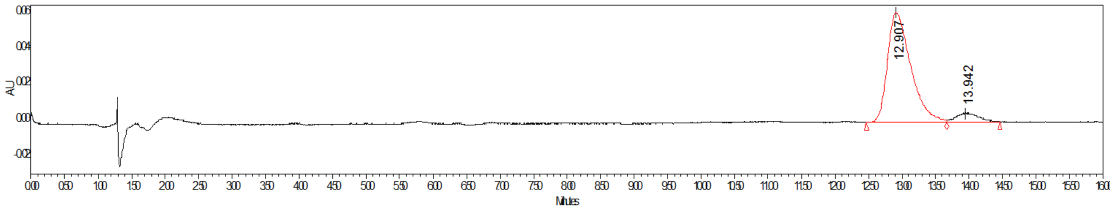

	Retention Time	Area	% Area
1	12.907	1464706	92.42

2	13.942	120135	7.58

Methyl 2-((4-(1-methyl-1H-imidazol-2-yl)-4-oxo-1-phenylbutan-2-yl)thio)acetate

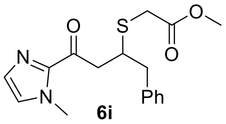

6i

Pale yellow oil. 74% yield and 93% ee was obtained according to the general procedure at 25 °C for 17 h.
HPLC (Chiral IF column), *i*-PrOH/*n*-Hexane = 20/80, Flow rate: 1.0 mL/min, 254 nm, t_r (minor) = 15.20 min, t_r (major) = 19.89 min.
^{1}H NMR (400 MHz, $CDCl_3$) δ 7.30 – 7.23 (m, 4H), 7.22 – 7.17 (m, 1H), 7.11 (d, *J* = 0.8 Hz, 1H), 7.00 (s, 1H), 3.94 (s, 3H), 3.70 (s, 3H), 3.55 (dd, *J* = 16.8, 8.0 Hz, 1H), 3.34 – 3.18 (m, 3H), 3.07 (dd, *J* = 13.6, 6.4 Hz, 1H), 2.92 (dd, *J* = 14.0, 8.4 Hz, 1H).
^{13}C NMR (100 MHz, $CDCl_3$) δ 190.2, 171.0, 143.1, 138.8, 129.5, 129.2, 128.5, 127.2, 126.7, 52.5, 43.4, 43.1, 42.1, 36.3, 33.2.
HRMS (ESI-FT) calculated for $C_{17}H_{21}N_2O_3S^+$ ([M]+H$^+$) = 333.1267, Found 333.1265.
$[\alpha]_D^{25}$ = –16.0 (*c* = 1.20, in CH_2Cl_2).
IR (film): 1734, 1673, 1408, 1281, 1154, 1006, 914 cm^{-1}.
Chiral HPLC spectrum of **6i**:

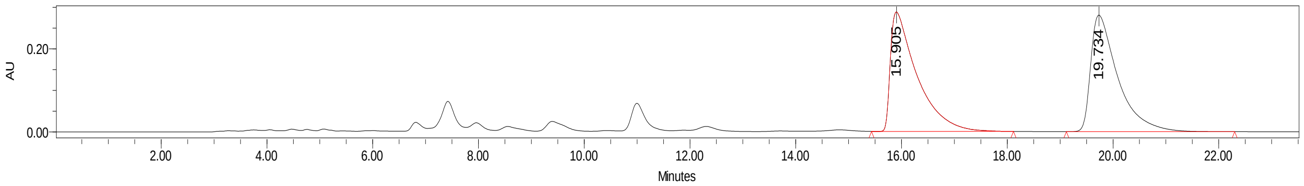

	Retention Time	Area	% Area
1	15.905	10017635	50.12
2	19.734	9969220	49.88

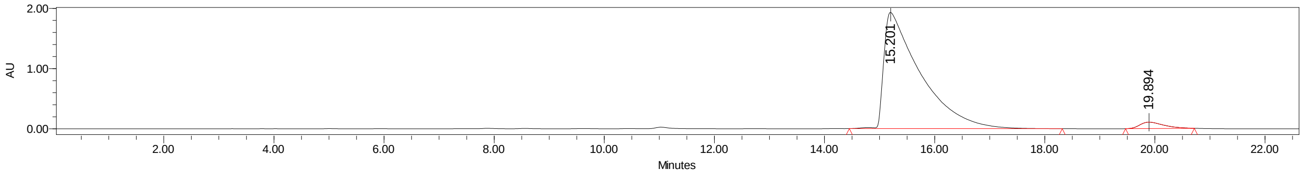

	Retention Time	Area	% Area
1	15.201	88850477	96.51
2	19.894	3213564	3.49

4-(4-Chlorophenyl)-1-(1-methyl-1H-imidazol-2-yl)-3-(phenylthio)butan-1-one

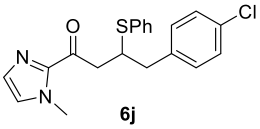

6j

Pale yellow oil. 87% yield and 91% ee was obtained according to the general procedure at 25 °C for 17 h.
UPC^2 (Chiral **OJ-3**), CO_2/MeOH = 90/10, Flow rate: 1.0 mL/min, 254 nm, t_r (minor) = 8.90 min, t_r (major) = 8.07 min.
^{1}H NMR (400 MHz, $CDCl_3$) δ 7.44 – 7.38(m, 2H), 7.29 – 7.21 (m, 3H), 7.20 – 7.15 (m, 2H), 7.15 – 7.08 (m, 3H), 6.99 (s, 1H), 4.09 – 4.01 (m, 1H), 3.88 (s, 3H), 3.44 (dd, *J* = 16.8, 7.6 Hz, 1H), 3.32 (dd, *J* = 16.8, 6.8 Hz, 1H), 2.99 (dd, *J* = 14.0, 6.4 Hz, 1H), 2.84 (dd, *J* = 14.0, 8.4 Hz, 1H).
^{13}C NMR (100 MHz, $CDCl_3$) δ 190.3, 143.0, 137.3, 134.4, 132.7, 132.4, 130.9, 129.3, 129.0, 128.4, 127.4, 127.2, 45.7, 43.5, 41.0, 36.2.
HRMS (ESI-FT) calculated for $C_{20}H_{20}{}^{34.9689}ClN_2OS^+$ ([M]+H$^+$) = 371.0979, Found 371.0974.
HRMS (ESI-FT) calculated for $C_{26}H_{29}{}^{36.9659}ClN_3O_3^+$ ([M]+H$^+$) = 373.0950, Found 373.0941.
$[\alpha]^{23}_{\lambda}$ = –29.5 (*c* = 0.60, in CH_2Cl_2, λ = 405 nm).
IR (film): 1671, 1486, 1407, 1286, 1155, 1090, 1018, 986 cm^{-1}.
UPC^2 spectrum of **6j**:

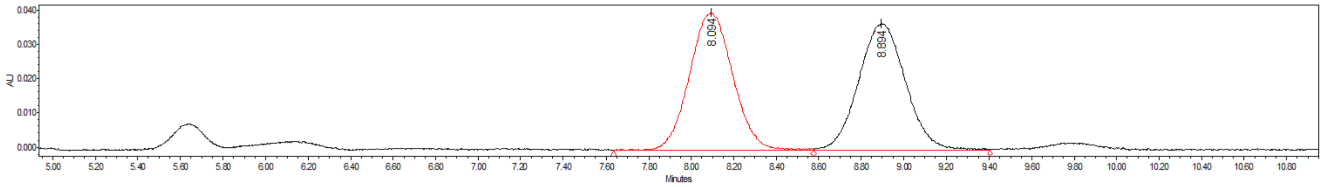

	Retention Time	Area	% Area
1	8.094	570277	50.17
2	8.894	566318	49.83

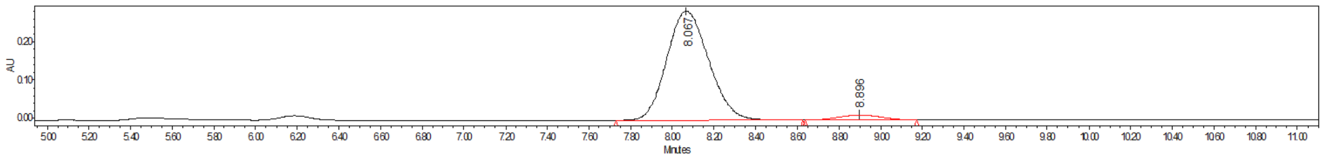

	Retention Time	Area	% Area
1	8.067	4017815	95.48
2	8.896	190120	4.52

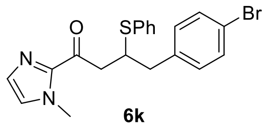

6k

Pale yellow oil. 74% yield and 85% ee was obtained according to the general procedure at 25 °C for 17 h.
UPC² (Chiral **OJ-3**), CO_2/MeOH = 90/10, Flow rate: 1.0 mL/min, 254 nm, t_r (minor) = 11.18 min, t_r (major) = 10.57 min.
^{1}H NMR (400 MHz, $CDCl_3$) δ 7.44 – 7.38 (m, 2H), 7.33 (d, *J* = 8.4 Hz, 2H), 7.29 – 7.21 (m, 3H), 7.12 (d, *J* = 0.8 Hz, 1H), 7.05 (d, *J* = 8.4 Hz, 2H), 6.99 (s, 1H), 4.09 – 4.00 (m, 1H), 3.88 (s, 3H), 3.43 (dd, *J* = 16.8, 7.2 Hz, 1H), 3.32 (dd, *J* = 16.8, 6.8 Hz, 1H), 2.98 (dd, *J* = 14.0, 6.4 Hz, 1H), 2.82 (dd, *J* = 13.6, 8.0 Hz, 1H).
^{13}C NMR (100 MHz, $CDCl_3$) δ 190.3, 143.0, 137.8, 134.4, 132.7, 131.4, 131.3, 129.3, 129.0, 127.4, 127.2, 120.5, 45.7, 43.5, 41.1, 36.2.
HRMS (ESI-FT) calculated for $C_{20}H_{20}{}^{78.9183}BrN_2OS^+$ ([M]+H$^+$) = 415.0474, Found 415.0472.
HRMS (ESI-FT) calculated for $C_{20}H_{20}{}^{80.9163}BrN_2OS^+$ ([M]+H$^+$) = 417.0454, Found 417.0449.
$[\alpha]^{23}_{\lambda}$ = –6.6 (*c* = 0.62, in CH_2Cl_2, λ = 546 nm).
IR (film): 1672, 1481, 1407, 1287, 1155, 1072, 1009cm^{-1}.
UPC² spectrum of **6k**:

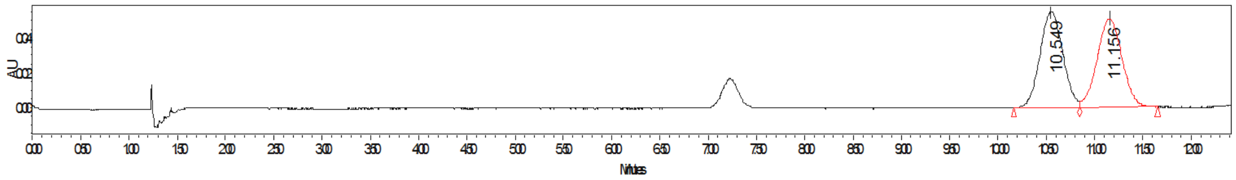

	Retention Time	Area	% Area
1	10.549	911399	49.92
2	11.156	914349	50.08

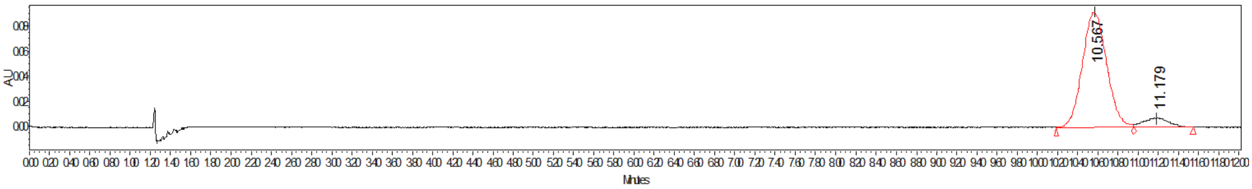

	Retention Time	Area	% Area
1	10.567	1533264	92.53
2	11.179	123775	7.47

1-(1-Methyl-1H-imidazol-2-yl)-3-(phenylthio)-4-(m-tolyl)butan-1-one

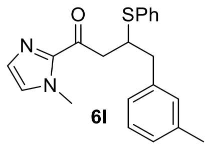

Pale yellow oil. 89% yield and 91% ee was obtained according to the general procedure at 25 °C for 17 h.
UPC² (Chiral **OJ-3**), CO_2/MeOH = 90/10, Flow rate: 1.0 mL/min, 254 nm, t_r (minor) = 15.28 min, t_r (major) = 14.12 min.
^{1}H NMR (400 MHz, $CDCl_3$) δ 7.44 (d, *J* = 7.2 Hz, 2H), 7.30 – 7.15 (m, 3H), 7.14 – 7.10 (m, 2H), 6.97 (d, *J* = 6.0 Hz, 4H), 4.11 – 4.03 (m, 1H), 3.87 (s, 3H), 3.49 (dd, *J* = 16.8, 8.0 Hz, 1H), 3.29 (dd, *J* = 16.8, 6.0 Hz, 1H), 3.03 (dd, *J* = 13.6, 6.0 Hz, 1H), 2.81 (dd, *J* = 14.0, 8.8 Hz, 1H), 2.26 (s, 3H).
^{13}C NMR (100 MHz, $CDCl_3$) δ 190.6, 143.2, 138.7, 138.0, 134.6, 132.7, 130.2, 129.2, 128.9, 128.3, 127.4, 127.2, 127.1, 126.5, 45.6, 43.4, 41.8, 36.2, 21.5.
HRMS (ESI-FT) calculated for $C_{21}H_{23}N_2OS^+$ ([M]+H$^+$) = 351.1526, Found 351.1522.
$[\alpha]^{26}_{\lambda}$ = +3.1 (*c* = 0.62, in CH_2Cl_2, λ = 546 nm).
IR (film): 1672, 1475, 1406, 1286, 1155, 987 cm^{-1}.
UPC² spectrum of **6l**:

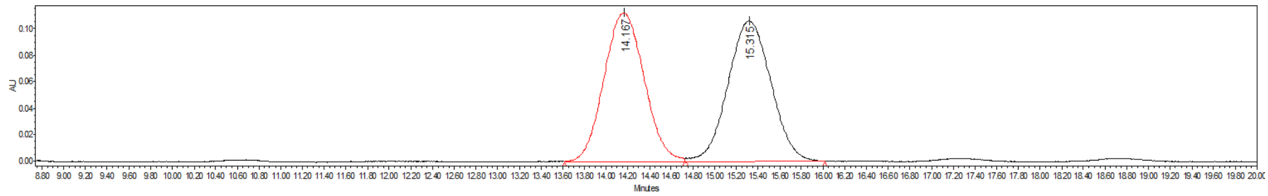

	Retention Time	Area	% Area
1	14.167	2884513	49.60
2	15.315	2931117	50.40

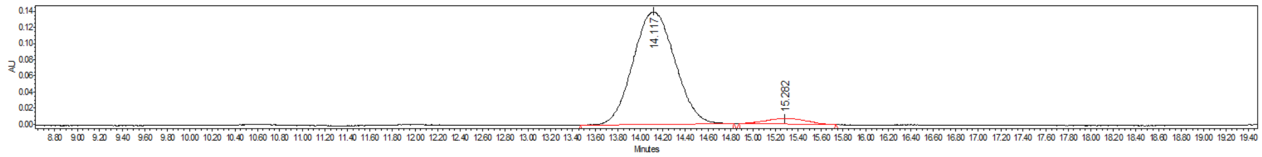

	Retention Time	Area	% Area
1	14.117	3575949	95.45
2	15.282	170352	4.55

4-(3-Methoxyphenyl)-1-(1-methyl-1H-imidazol-2-yl)-3-(phenylthio)butan-1-one

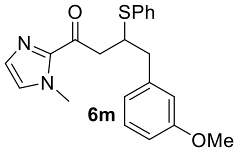

6m

Pale yellow oil. 92% yield and 91% ee was obtained according to the general procedure at 25 °C for 17 h.
UPC^2 (Chiral **OJ-3**), CO_2/MeOH = 90/10, Flow rate: 1.0 mL/min, 254 nm, t_r (minor) = 7.41 min, t_r (major) = 8.02 min.
^{1}H NMR (400 MHz, $CDCl_3$) δ 7.49 – 7.40 (m, 2H), 7.30 – 7.18 (m, 3H), 7.17 – 7.12 (m, 1H), 7.11 (s, 1H), 6.97 (s, 1H), 4.15 – 4.03 (m, 1H), 3.88 (s, 3H), 3.74 (s, 3H), 3.49 (dd, *J* = 16.8, 7.6 Hz, 1H), 3.30 (dd, *J* = 16.8, 6.0 Hz, 1H), 3.04 (dd, *J* = 14.0, 6.0 Hz, 1H), 2.83 (dd, *J* = 13.6, 8.4 Hz, 1H).
^{13}C NMR (100 MHz, $CDCl_3$) δ 190.4, 159.7, 143.1, 140.4, 134.6, 132.7, 129.4, 129.2, 128.9, 127.3, 127.1, 121.9, 115.0, 112.2, 55.2, 45.6, 43.4, 41.8, 36.2.
HRMS (ESI-FT) calculated for $C_{21}H_{23}N_2O_2S^+$ ([M]+H$^+$) = 367.1475, Found 367.1474.
$[\alpha]^{21}_{\lambda}$ = +5.1 (*c* = 0.67, in CH_2Cl_2, λ = 546 nm).
IR (film): 1672, 1588, 1408, 1261, 1155, 1045, 989 cm^{-1}.
UPC^2 spectrum of **6m**:

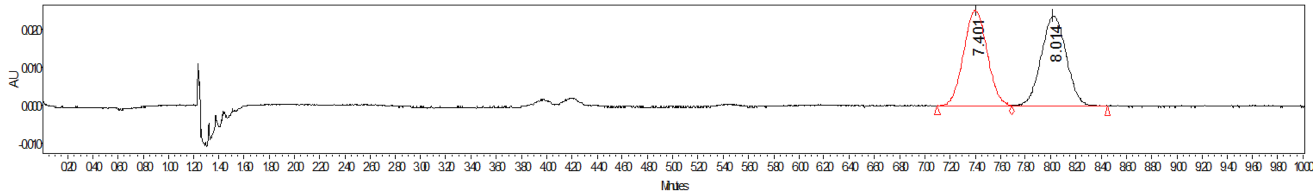

	Retention Time	Area	% Area
1	7.401	334731	49.87
2	8.014	336510	50.13

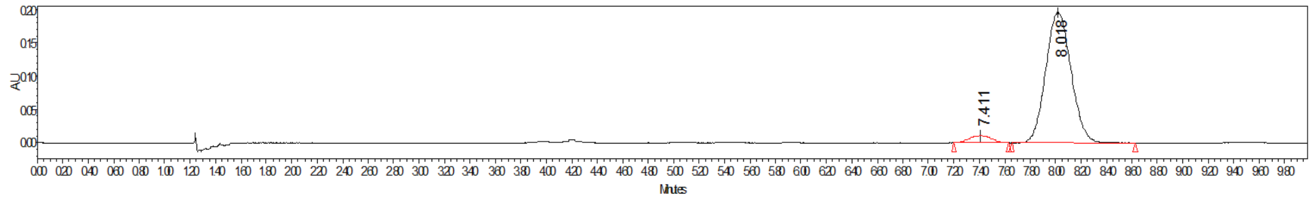

	Retention Time	Area	% Area
1	7.411	129097	4.53
2	8.018	2718431	95.47

1-(1-Methyl-1H-imidazol-2-yl)-3-(phenylthio)heptan-1-one

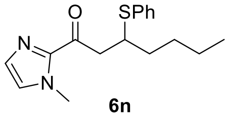

6n

Colorless oil. 76% yield and 80% ee was obtained according to the general procedure at 25 °C for 5 days.
UPC^2 (Chiral **ODH**), CO_2/MeOH = 95/5, Flow rate: 1.0 mL/min, 254 nm, t_r (minor) = 19.17 min, t_r (major) = 18.55 min.

^{1}H NMR (400 MHz, $CDCl_3$) δ 7.42 (d, *J* = 7.6 Hz, 2H), 7.27 – 7.14 (m, 3H), 7.13 (s, 1H), 7.00 (s, 1H), 3.94 (s, 3H), 3.86 – 3.77 (m, 1H), 3.47 – 3.32 (m, 2H), 1.68 – 1.60 (m, 3H), 1.56 – 1.41 (m, 2H), 1.36 – 1.26 (m, 2H), 0.87 (t, *J* = 7.2 Hz, 3H).
^{13}C NMR (100 MHz, $CDCl_3$) δ 191.0, 143.2, 134.9, 132.5, 129.2, 128.9, 127.1, 127.0, 44.8, 44.5, 36.3, 34.9, 29.2, 22.6, 14.1.
HRMS (ESI-FT) calculated for $C_{17}H_{23}N_2OS^+$ ([M]+H$^+$) = 303.1526, Found 303.1519.
$[\alpha]^{23}_{\lambda}$ = +24 (*c* = 0.60, in CH_2Cl_2, λ = 405 nm).
IR (film): 1672, 1466, 1408, 1289, 1155, 1025 cm^{-1}.
UPC^2 spectrum of **6n**:

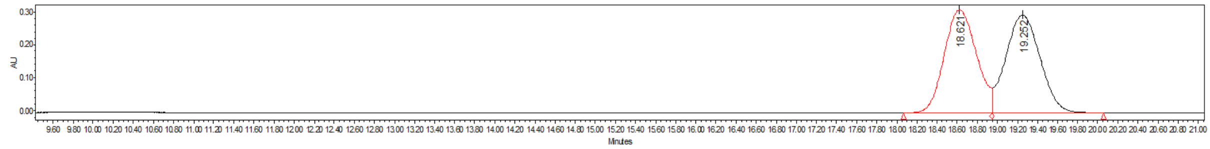

	Retention Time	Area	% Area
1	18.621	6962262	49.66
2	19.252	7057913	50.34

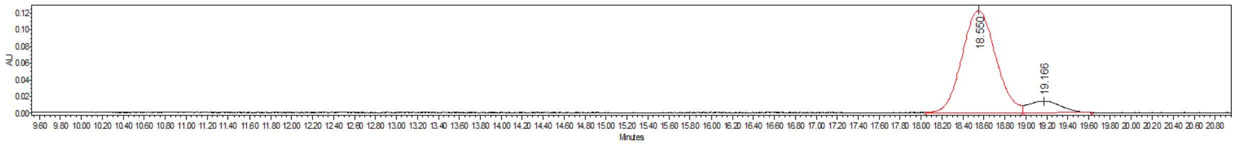

	Retention Time	Area	% Area
1	18.550	2742091	90.33
2	19.166	293709	9.67

Methyl 2-((4-(1-methyl-1H-imidazol-2-yl)-4-oxo-1-(m-tolyl)butan-2-yl)thio)acetate

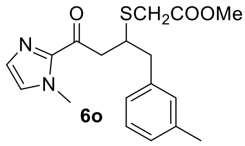

6o

Colorless oil. 60% yield and 90% ee was obtained according to the general procedure at 25 °C for 17 h.
UPC^2 (Chiral **OJ-3**), CO_2/MeOH = 95/5, Flow rate: 1.0 mL/min, 254 nm, t_r (minor) = 5.90 min, t_r (major) = 6.42 min.
^{1}H NMR (400 MHz, $CDCl_3$) δ 7.18 – 7.13 (m, 1H), 7.11 (d, *J* = 0.8 Hz, 1H), 7.06 – 6.98 (m, 4H), 3.94 (s, 3H), 3.71 (s, 3H), 3.54 (dd, *J* = 17.2, 8.4 Hz, 1H), 3.35 – 3.18 (m, 3H), 3.04 (dd, *J* = 13.6, 6.4 Hz, 1H), 2.87 (dd, *J* = 13.6, 8.4 Hz, 1H), 2.30 (s, 3H).
^{13}C NMR (100 MHz, $CDCl_3$) δ 190.3, 171.1, 143.2, 138.7, 138.1, 130.3, 129.2, 128.4, 127.5, 127.2, 126.5, 52.5, 43.4, 43.1, 42.1, 36.3, 33.2, 21.5.
HRMS (ESI-FT) calculated for $C_{18}H_{23}N_2O_3S^+$ ([M]+H$^+$) = 347.1424, Found 347.1417.
$[\alpha]^{24}_{\lambda}$ = –36.3 (*c* = 0.26, in CH_2Cl_2, λ = 436 nm).
IR (film): 1736, 1673, 1409, 1280, 1154, 1006 cm^{-1}.
UPC^2 spectrum of **6o**:

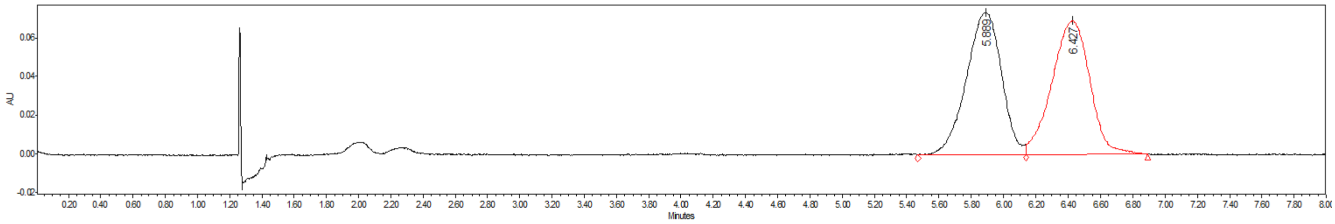

	Retention Time	Area	% Area
1	5.889	1120344	49.95
2	6.427	1122520	50.05

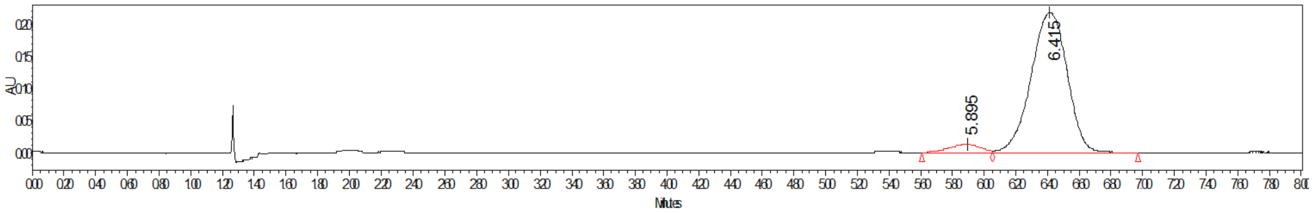

	Retention Time	Area	% Area
1	5.895	180661	4.95
2	6.415	3472013	95.05

Methyl 2-((1-(3-methoxyphenyl)-4-(1-methyl-1H-imidazol-2-yl)-4-oxobutan-2-yl)thio)acetate

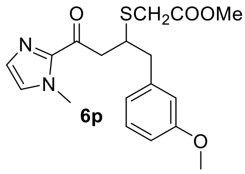

Colorless oil. 70% yield and 92% ee was obtained according to the general procedure at 25 °C for 17 h.
UPC² (Chiral **OJ-3**), CO_2/MeOH = 90/10, Flow rate: 1.0 mL/min, 254 nm, t_r (minor) = 4.06 min, t_r (major) = 4.38 min.
¹H NMR (400 MHz, $CDCl_3$) δ 7.17 (t, *J* = 8.0 Hz, 1H), 7.10 (d, *J* = 0.4 Hz, 1H), 7.00 (s, 1H), 6.83 (d, *J* = 7.6 Hz, 1H), 6.79 (s, 1H), 6.74 (dd, *J* = 8.0, 2.4 Hz, 1H), 3.94 (s, 3H), 3.77 (s, 3H), 3.70 (s, 3H), 3.53 (dd, *J* = 17.2, 8.4 Hz, 1H), 3.35 – 3.18 (m, 3H), 3.05 (dd, *J* = 14.0, 6.8 Hz, 1H), 2.89 (dd, *J* = 13.6, 8.4 Hz, 1H).
¹³C NMR (100 MHz, $CDCl_3$) δ 190.2, 171.0, 159.7, 143.1, 140.4, 129.5, 129.2, 127.2, 121.9, 115.0, 112.3, 55.33, 52.5, 43.4, 43.0, 42.2, 36.2, 33.2.
HRMS (ESI-FT) calculated for $C_{18}H_{23}N_2O_4S^+$ ([M]+H⁺) = 363.1373, Found 363.1364.
$[\alpha]^{23}_{\lambda}$ = –34.0 (*c* = 0.50, in CH_2Cl_2, λ = 436 nm).
IR (film): 1735, 1673, 1600, 1489, 1408, 1263, 1153, 1044, 1008 cm^{-1}.
UPC² spectrum of **6p**:

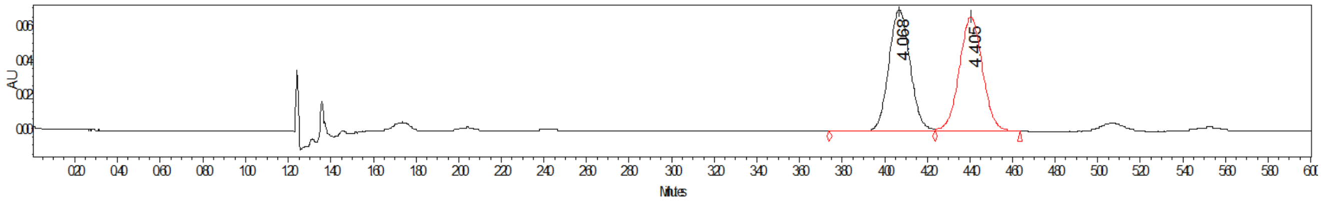

	Retention Time	Area	% Area
1	4.058	167387	4.19
2	4.384	3824971	95.81

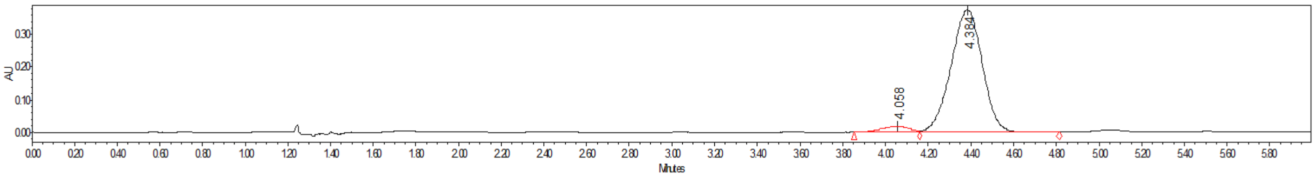

	Retention Time	Area	% Area
1	4.058	167387	4.19
2	4.384	3824971	95.81

(2*R*,3*R*)-3-benzyl-1,5-bis(1-methyl-1H-imidazol-2-yl)-2-phenethylpentane-1,5-dione

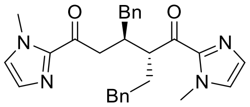

7

HPLC (Chiral IE column), *i*-PrOH/*n*-Hexane = 30/70, Flow rate: 1.0 mL/min, 254 nm, t_r (minor) = 15.54 min, t_r (major) = 17.13 min.
¹H NMR (400 MHz, $CDCl_3$) δ 7.25 – 7.18 (m, 2H), 7.17 – 7.04 (m, 8H), 6.99 (dd, *J* = 2.4, 0.8 Hz, 2H), 6.95 (s, 1H), 6.86 (s, 1H), 4.30 – 4.19 (m, 1H), 3.89 (s, 3H), 3.75 (s, 3H), 3.26 – 3.13 (m, 1H), 3.11 – 2.90 (m, 3H), 2.67 – 2.51 (m, 3H), 2.37 – 2.26 (m, 1H), 1.97 – 1.86 (m, 1H).
¹³C NMR (100 MHz, $CDCl_3$) δ 195.3, 191.7, 143.8, 143.2, 142.2, 140.1, 129.4, 128.8, 128.5, 128.4, 128.2, 128.0, 127.1, 126.5, 125.9, 125.7, 49.5, 39.6, 39.2, 38.5, 36.1, 35.9, 34.4, 30.0.
HRMS (ESI-FT) calculated for $C_{28}H_{31}N_4O_2^+$ ([M]+H⁺) = 455.2442, Found 455.2446.
$[\alpha]^{22}_{D}$ = –46.8 (*c* = 2.25, in CH_2Cl_2).
IR (film): 1666, 1455, 1407, 1287, 1154, 971 cm^{-1}.
Chiral HPLC spectrum of **7**:

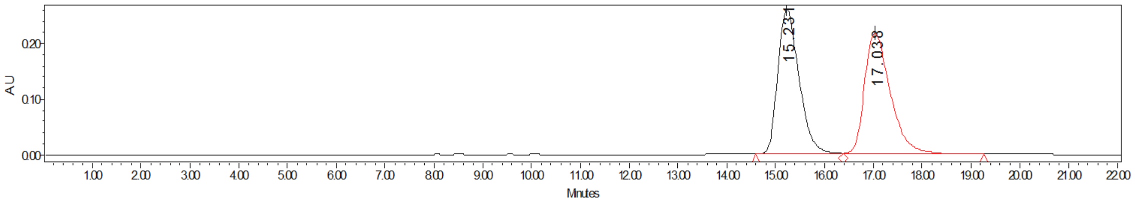

	Retention Time	Area	% Area
1	15.231	7919536	49.55
2	17.038	8064647	50.45

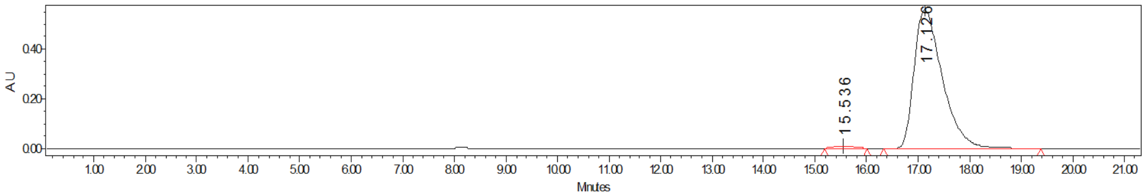

	Retention Time	Area	% Area
1	15.536	260150	1.22
2	17.126	21022434	98.78

(*R*)-1-(1-methyl-1H-imidazol-2-yl)-4-phenyl-3-(phenylsulfonyl)butan-1-one

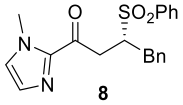

8

HPLC (Chiral IA column), *i*-PrOH/*n*-Hexane = 20/80, Flow rate: 1.0 mL/min, 254 nm, t_r (minor) = 22.81 min, t_r (major) = 30.10 min.
^{1}H NMR (400 MHz, $CDCl_3$) δ 7.97 – 7.87 (m, 2H), 7.66 – 7.58 (m, 1H), 7.51 (t, *J* = 8.0 Hz, 2H), 7.19 – 7.04 (m, 6H), 6.93 (s, 1H), 4.36 – 4.26 (m, 1H), 3.77 (dd, *J* = 17.6, 6.8 Hz, 1H), 3.68 (s, 3H), 3.46 (dd, *J* = 13.6, 4.0 Hz, 1H), 3.16 (dd, *J* = 17.6, 5.6 Hz, 1H), 2.71 (dd, *J* = 13.6, 11.2 Hz, 1H).
^{13}C NMR (100 MHz, $CDCl_3$) δ 187.4, 142.5, 137.5, 136.3, 133.8, 129.5, 129.4, 129.3, 129.2, 128.6, 127.2, 127.0, 61.7, 36.7, 36.0, 34.7.
HRMS (ESI-FT) calculated for $C_{20}H_{21}N_2O_3S^+$ ($[M+H^+]$) = 369.1267, Found 369.1272.
$[\alpha]_D^{25}$ = –32.8 (*c* = 2.84, in CH_2Cl_2).
IR (film): 1676, 1449, 1409, 1300, 1143, 1082, 990, 915 cm^{-1}.
Chiral HPLC spectrum of **8**:

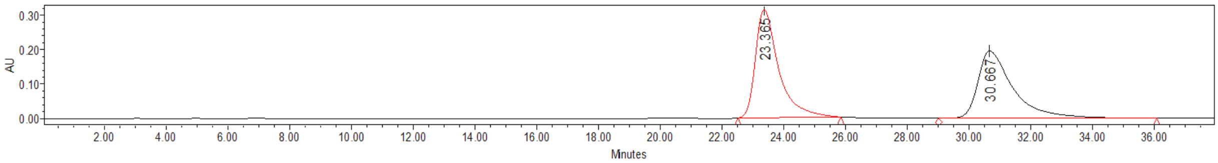

	Retention Time	Area	% Area
1	23.365	16413161	51.43
2	30.667	15497585	48.57

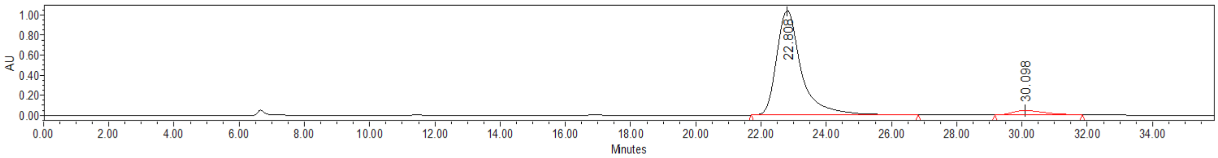

	Retention Time	Area	% Area
1	22.808	54659762	94.92
2	30.098	2928035	5.08

(*R*)-1,4-diphenyl-3-(phenylsulfonyl)butan-1-one

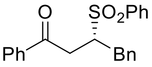

9

HPLC (Chiral ADH column), *i*-PrOH/*n*-Hexane = 30/70, Flow rate: 1.0 mL/min, 254 nm, t_r (minor) = 12.52 min, t_r (major) = 13.34 min.
^{1}H NMR (400 MHz, $CDCl_3$) δ 7.93 – 7.87 (m, 2H), 7.78 – 7.70 (m, 2H), 7.62 – 7.55 (m, 1H), 7.55 – 7.45 (m, 3H), 7.39 (t, *J* = 7.7 Hz, 2H), 7.20 – 7.06 (m, 5H), 4.48 – 4.34 (m, 1H), 3.70 (dd, *J* = 18.0, 6.0 Hz, 1H), 3.36 (dd, *J* = 14.0, 4.8 Hz, 1H), 3.07 (dd, *J* = 18.0, 5.6 Hz, 1H), 2.93 (dd, *J* = 14.4, 10.0 Hz, 1H).
^{13}C NMR (100 MHz, $CDCl_3$) δ 195.4, 137.9, 136.1, 133.9, 133.5, 129.3, 129.3, 128.8, 128.7, 128.61 128.0, 127.1, 60.9, 36.0, 34.5.
HRMS (ESI-FT) calculated for $C_{22}H_{21}O_3S^+$ ($[M+H^+]$) = 365.1206, Found 365.1210.
$[\alpha]^{18}_{\lambda}$ = –14.8 (*c* = 0.8, in CH_2Cl_2, λ = 436 nm).
IR (film): 1759, 1686, 1448, 1300, 1233, 1143, 1082, 994 cm^{-1}.
Chiral HPLC spectrum of **9**:

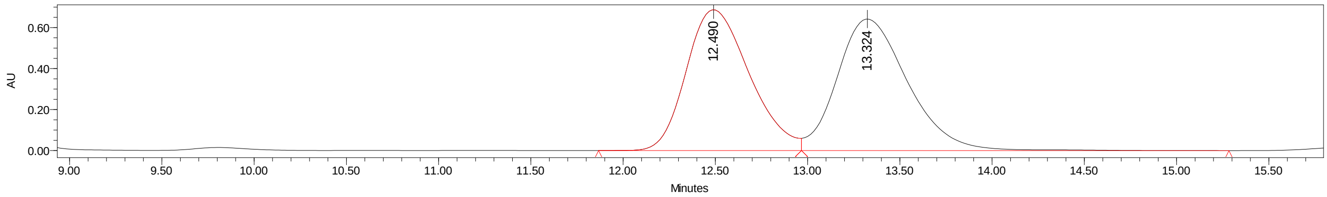

	Retention Time	Area	% Area
1	12.490	16355169	49.00

2	13.324	17025843	51.00

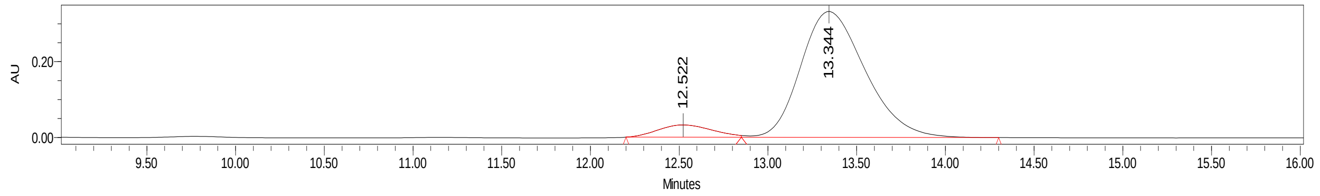

	Retention Time	Area	% Area
1	12.522	689193	7.57
2	13.344	8415349	92.43

(*1S*,*2S*,*E*)-2-((*R*)-3-amino-6-chloro-1-methyl-2-oxoindolin-3-yl)-1-(1-methyl-1H-imidazol-2-yl)-4-phenylbut-3-en-1-yl 3-chlorobenzoate

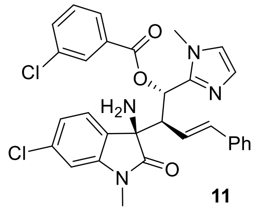

11

HPLC (Chiral IA column), *i*-PrOH/*n*-Hexane = 20/80, Flow rate: 1.0 mL/min, 254 nm, t_r (minor) = 22.81 min, t_r (major) = 30.10 min.
^{1}H NMR (400 MHz, $CDCl_3$) δ 7.95 (s, 1H), 7.87 (d, *J* = 7.6 Hz, 1H), 7.55 (d, *J* = 8.0 Hz, 1H), 7.42 – 7.34 (m, 2H), 7.34 – 7.18 (m, 6H), 7.10 (dd, *J* = 7.6, 1.2 Hz, 1H), 6.91 (s, 1H), 6.67 – 6.58 (m, 2H), 6.44 (d, *J* = 15.6 Hz, 1H), 6.08 (dd, *J* = 15.6, 10.0 Hz, 1H), 5.74 (d, *J* = 10.8 Hz, 1H), 4.04 (t, *J* = 10.4 Hz, 1H), 3.64 (s, 3H), 2.70 (s, 3H), 1.86 (s, 2H).
^{13}C NMR (100 MHz, $CDCl_3$) δ 178.9, 163.8, 145.1, 144.3, 137.8, 136.4, 135.6, 134.9, 133.7, 130.8, 130.2, 130.0, 128.8, 128.4, 128.3, 128.2, 128.2, 126.5, 125.2, 122.9, 121.4, 121.3, 109.5, 68.4, 61.4, 54.0, 32.9, 26.5.
HRMS (ESI-FT) calculated for $C_{30}H_{25}{}^{34.9689}Cl_2N_4O_3^-$ ([M]-H$^+$) = 559.1309, Found 559.1309.
HRMS (ESI-FT) calculated for $C_{30}H_{25}{}^{36.9659}Cl_2N_4O_3^-$ ([M]-H$^+$) = 561.1280, Found 561.1283.
$[\alpha]_D^{23}$ = +100.0 (*c* = 0.82, in CH_2Cl_2).
IR (film): 2361, 1721, 1607, 1493, 1372, 1282, 1253, 1119, 1074, 967 cm^{-1}.
Chiral HPLC spectrum of **11**:

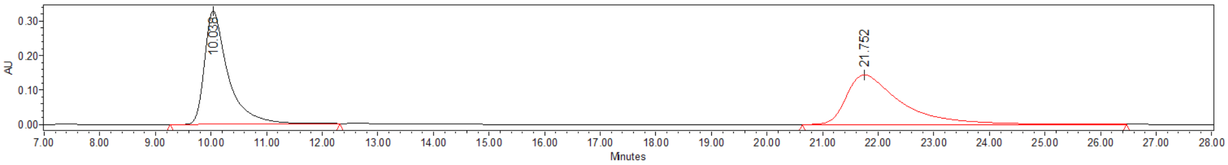

	Retention Time	Area	% Area
1	10.038	9682133	49.64
2	21.752	9820707	50.36

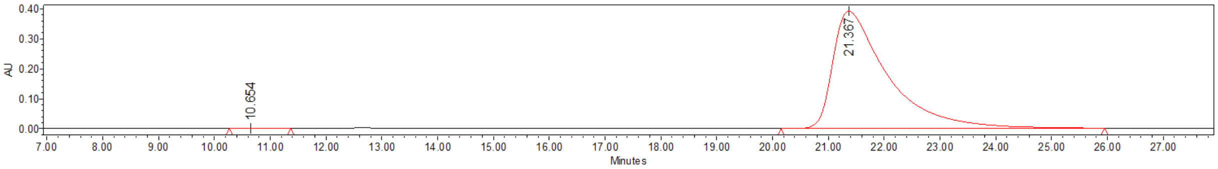

	Retention Time	Area	% Area
1	10.654	16738	0.06
2	21.367	26637232	99.94

Copy of 1H, 13C NMR spectra of substrates and products

1a

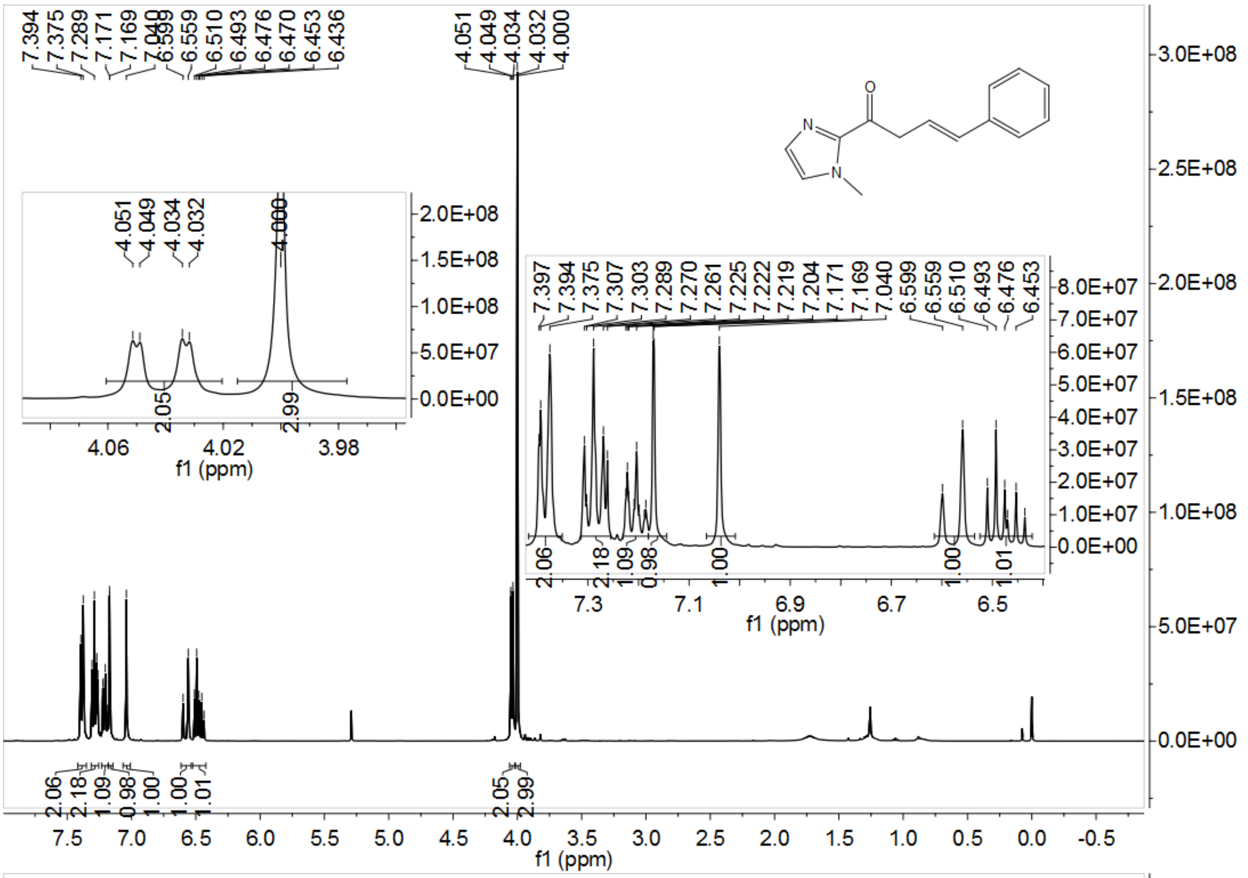

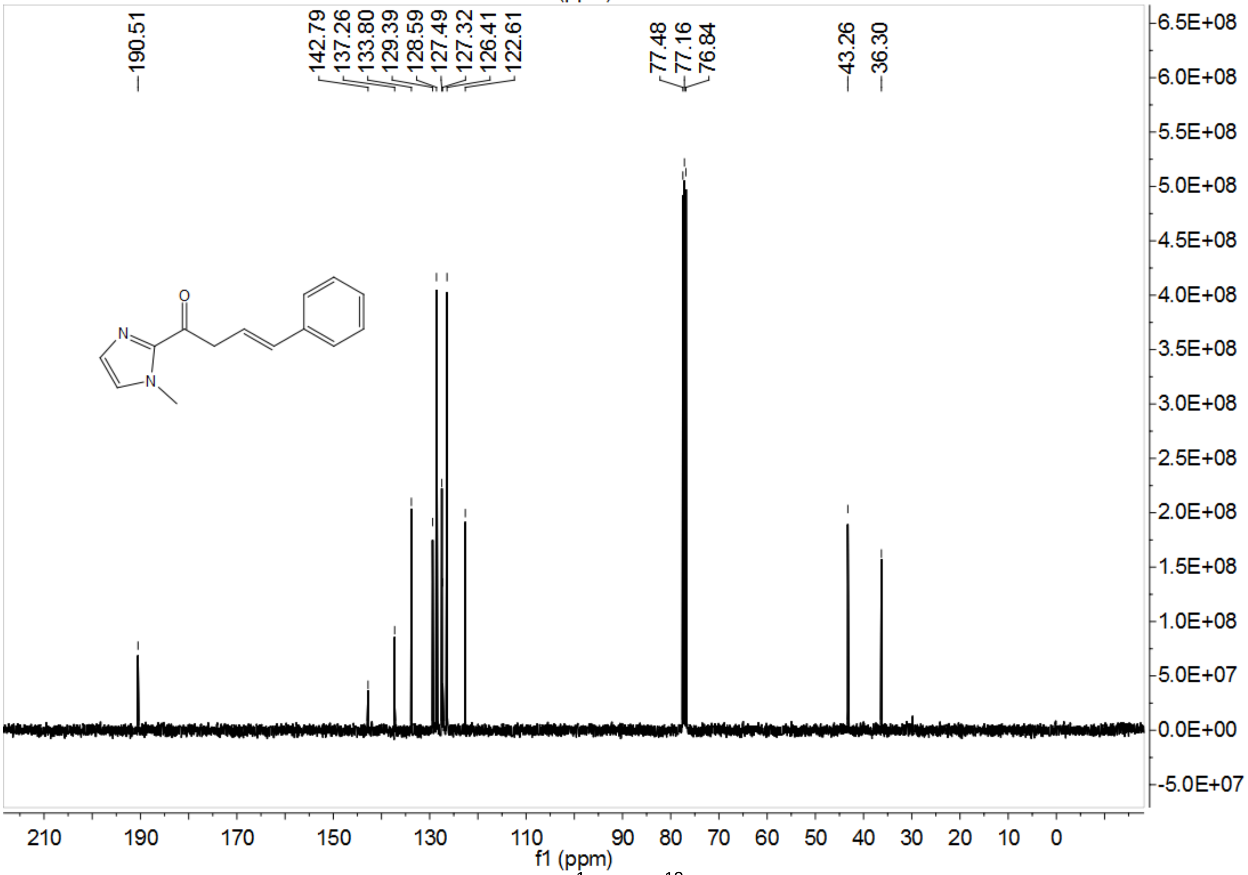

Supplementary Figure 5. ^{1}H and ^{13}C spectra for substrate **1a**

1b

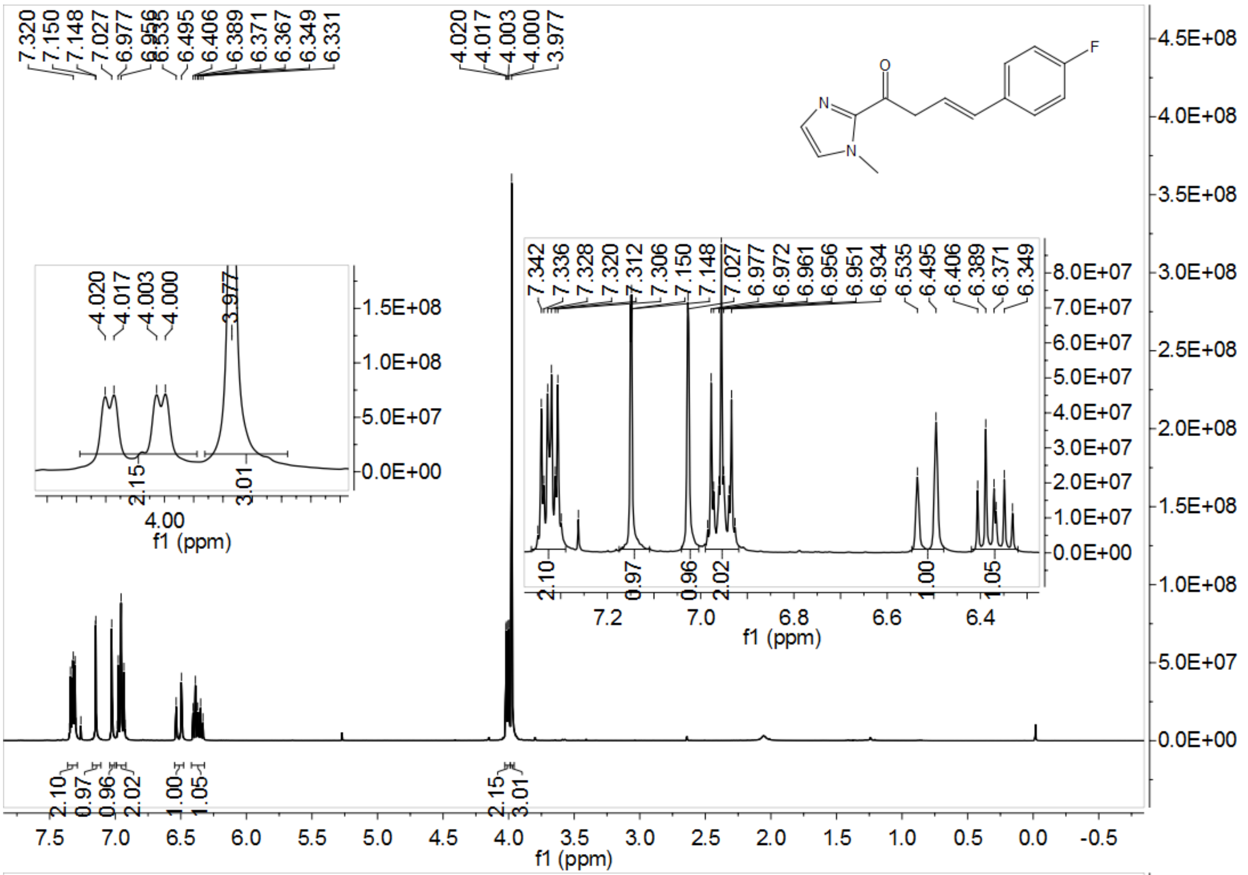

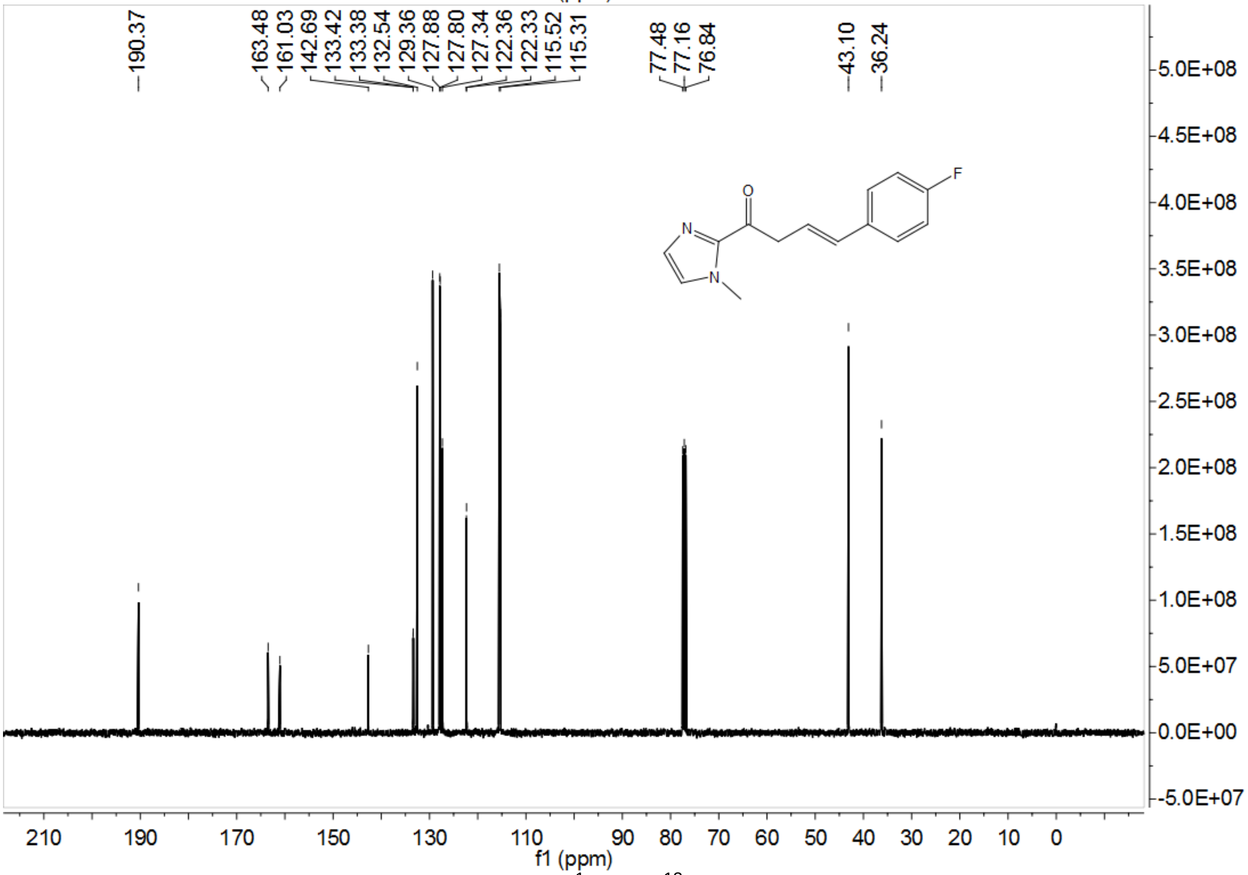

Supplementary Figure 6. ^{1}H and ^{13}C spectra for substrate **1b**

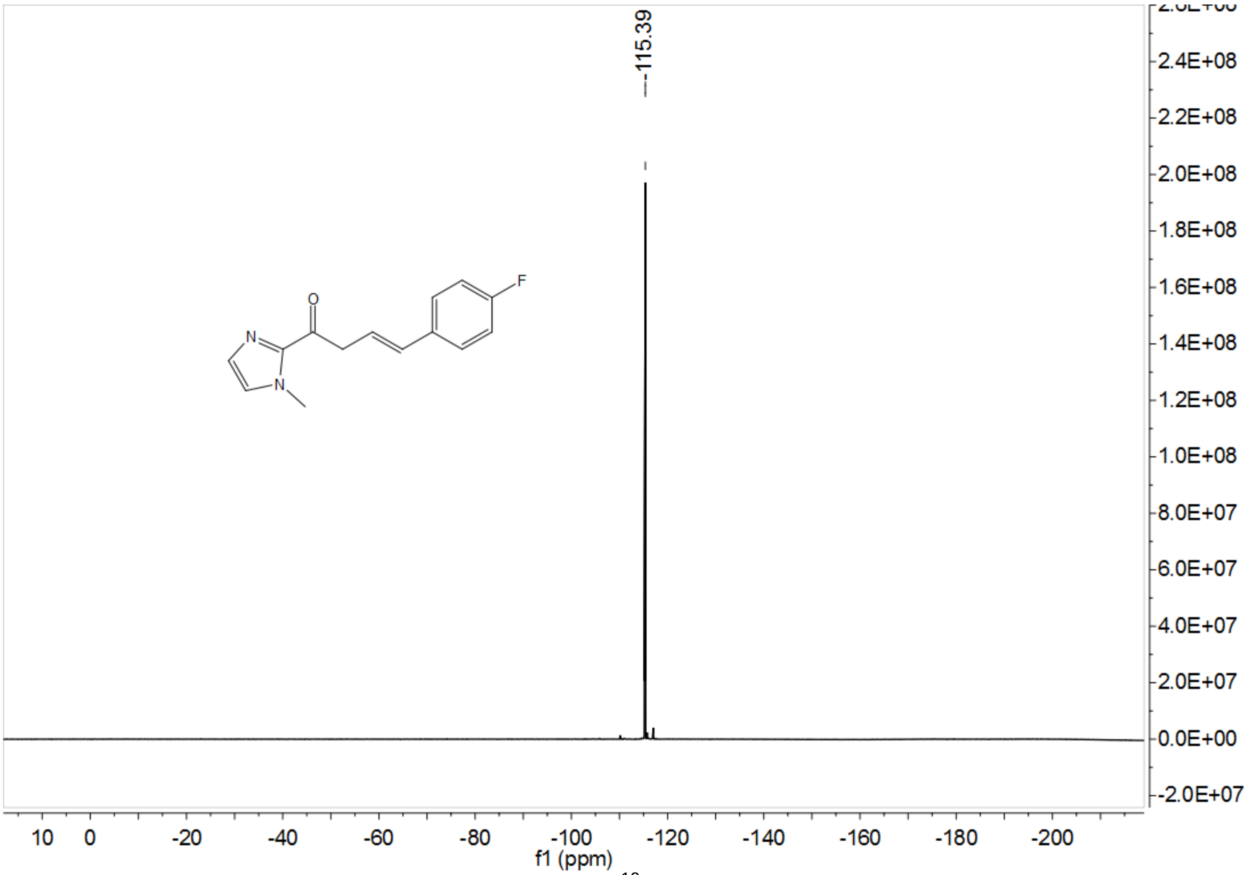

Supplementary Figure 7. ^{19}F spectra for substrate **1b**

1c

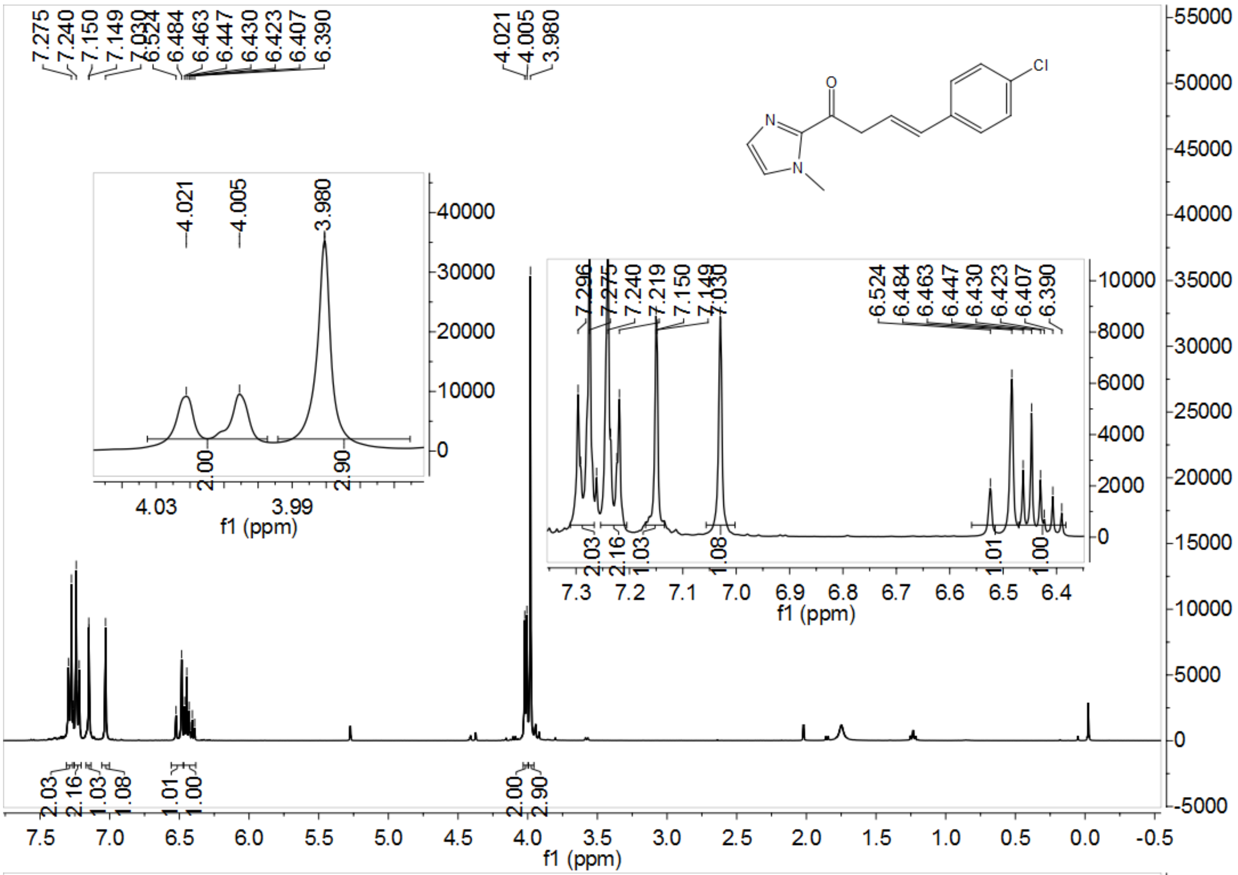

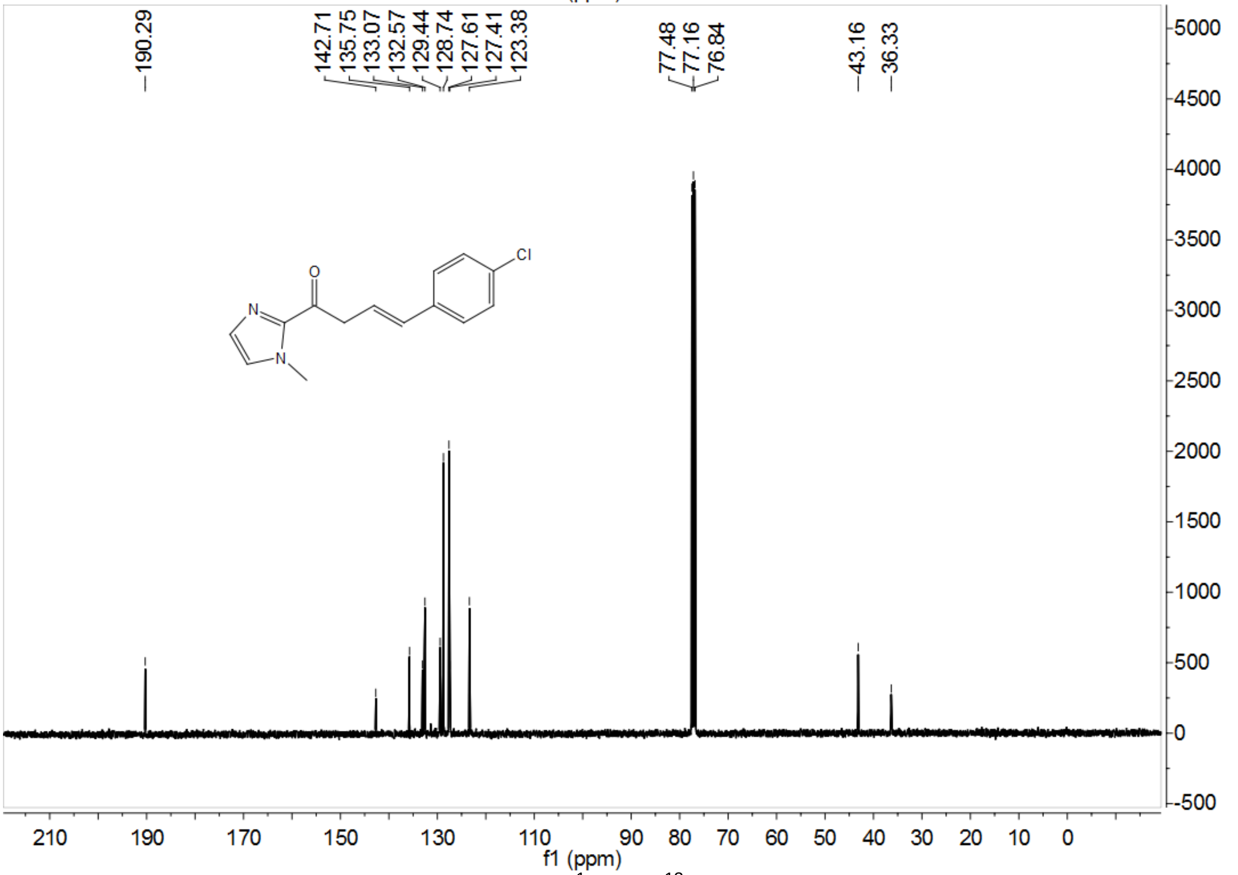

Supplementary Figure 8. ^{1}H and ^{13}C spectra for substrate **1c**

1d

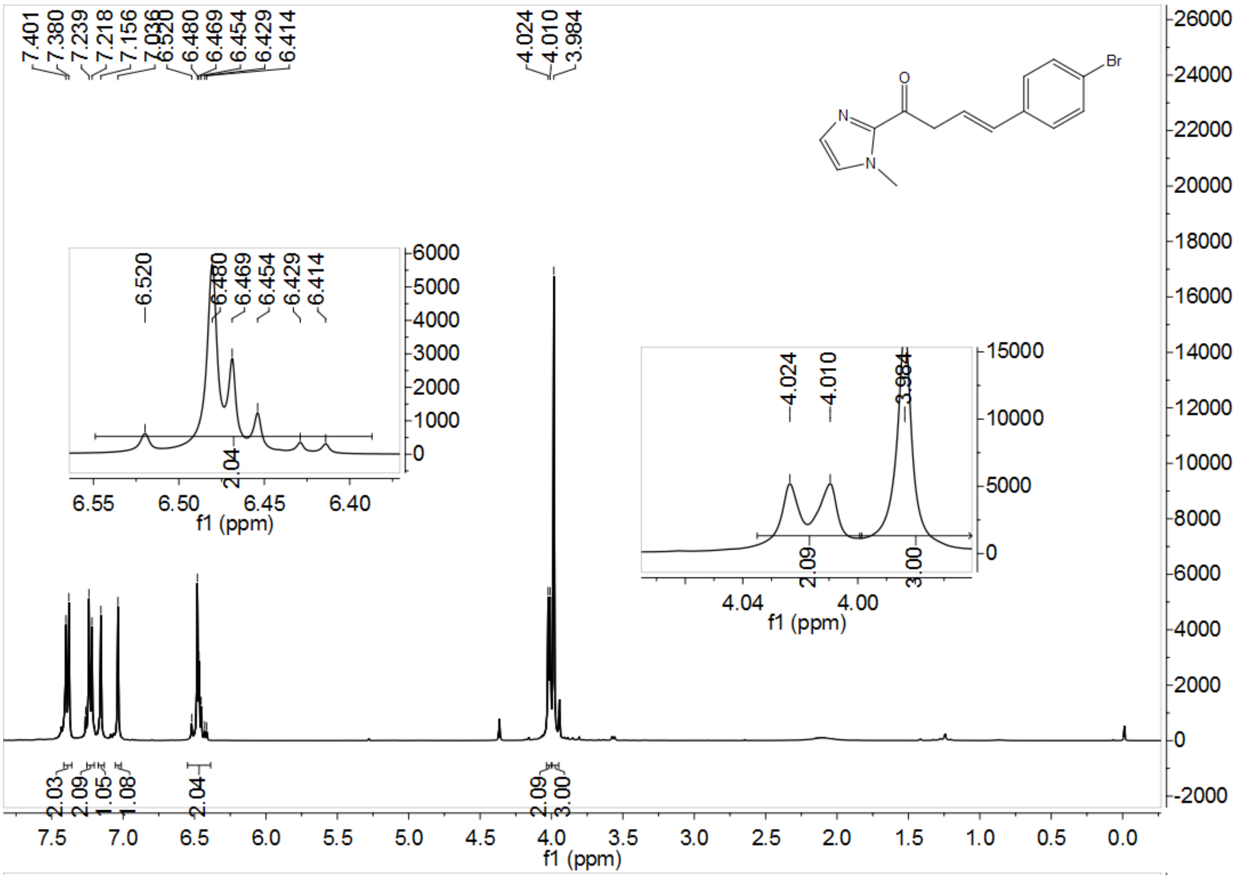

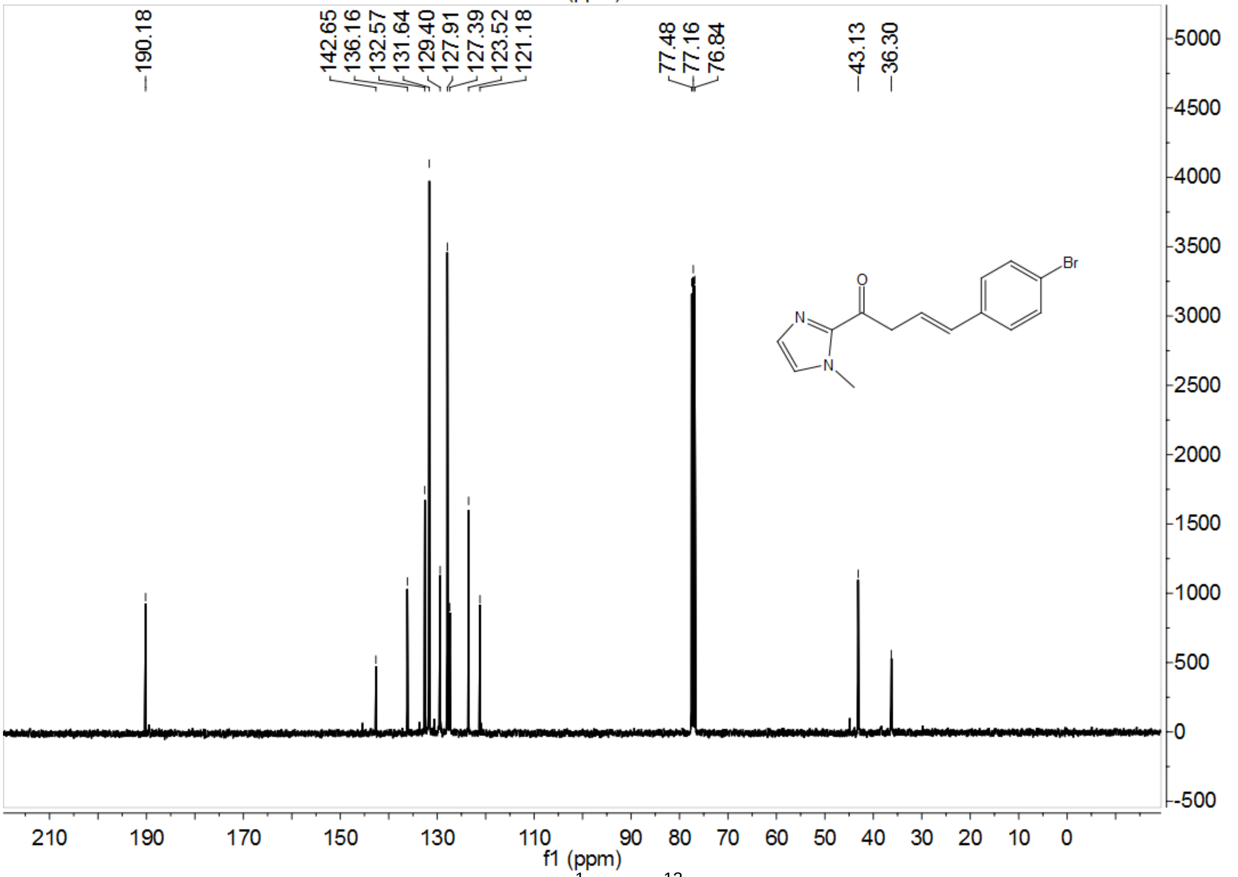

Supplementary Figure 9. ^{1}H and ^{13}C spectra for substrate **1d**

1e

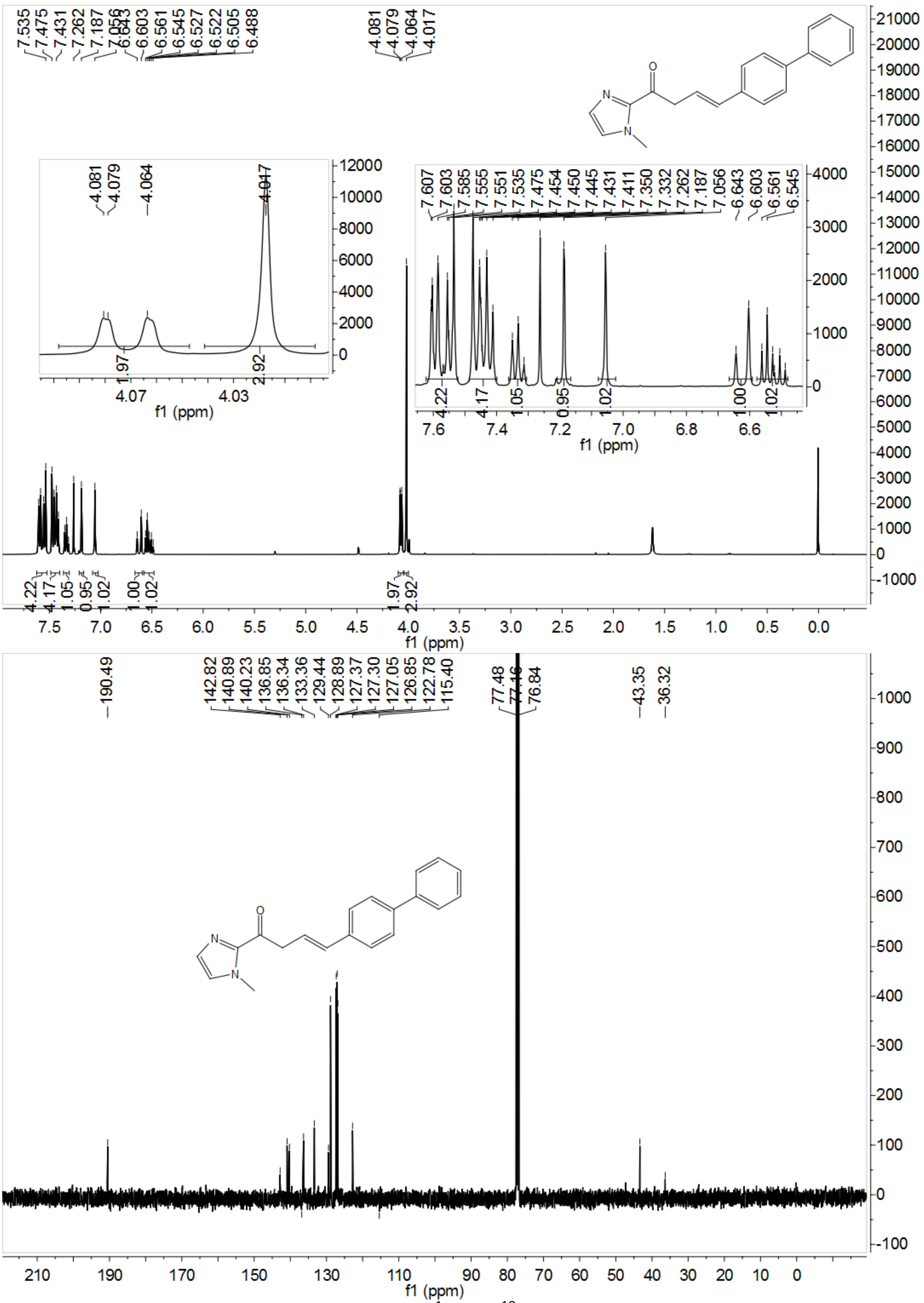

Supplementary Figure 10. ^{1}H and ^{13}C spectra for substrate **1e**

1f

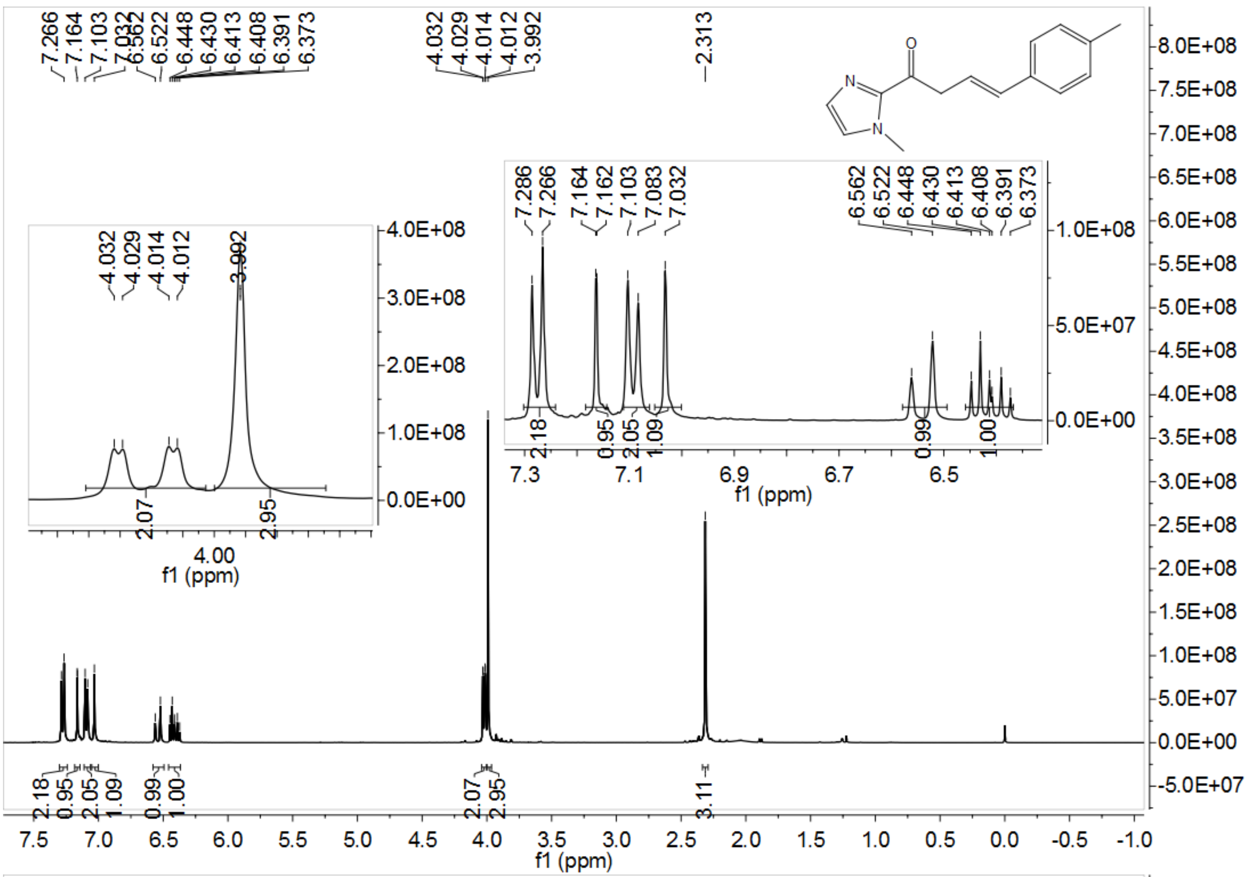

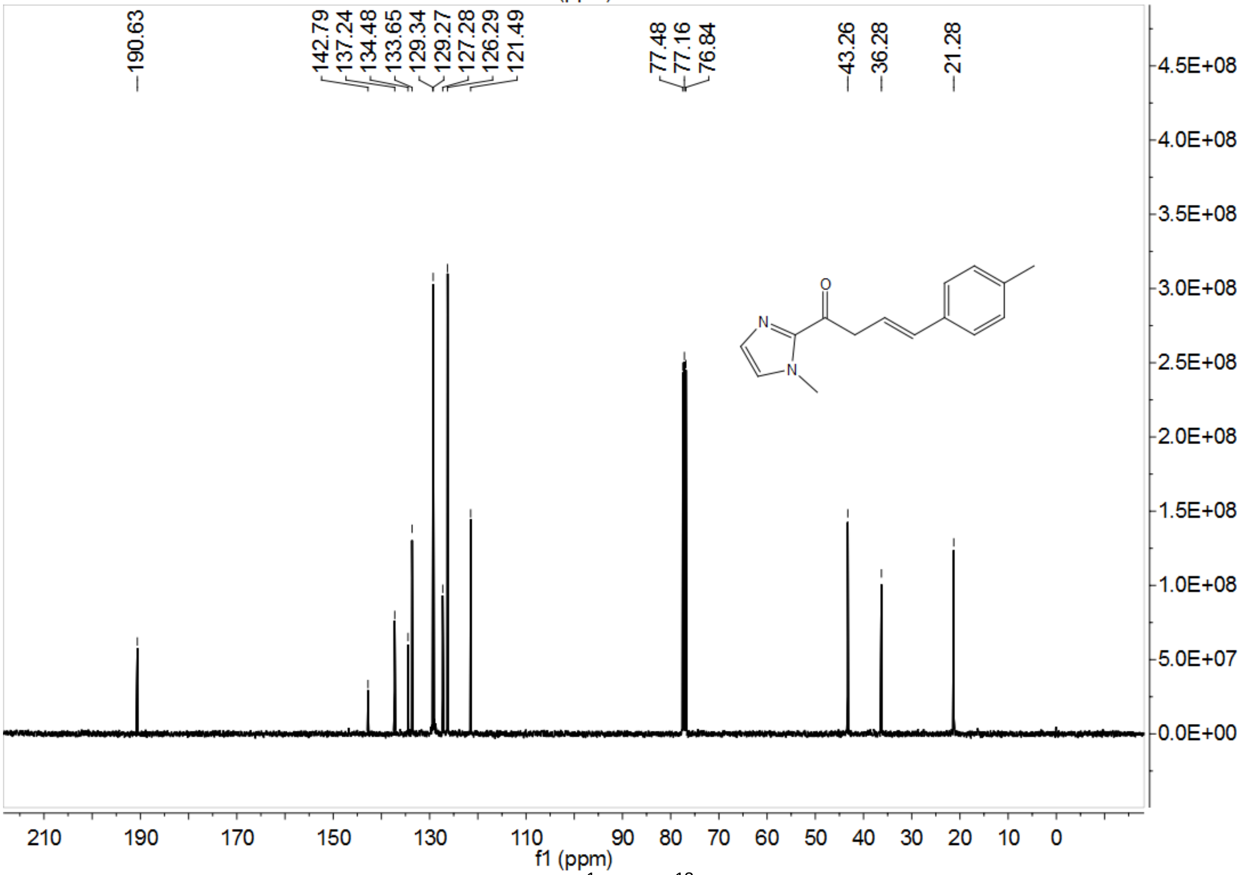

Supplementary Figure 11. ^{1}H and ^{13}C spectra for substrate **1f**

1g

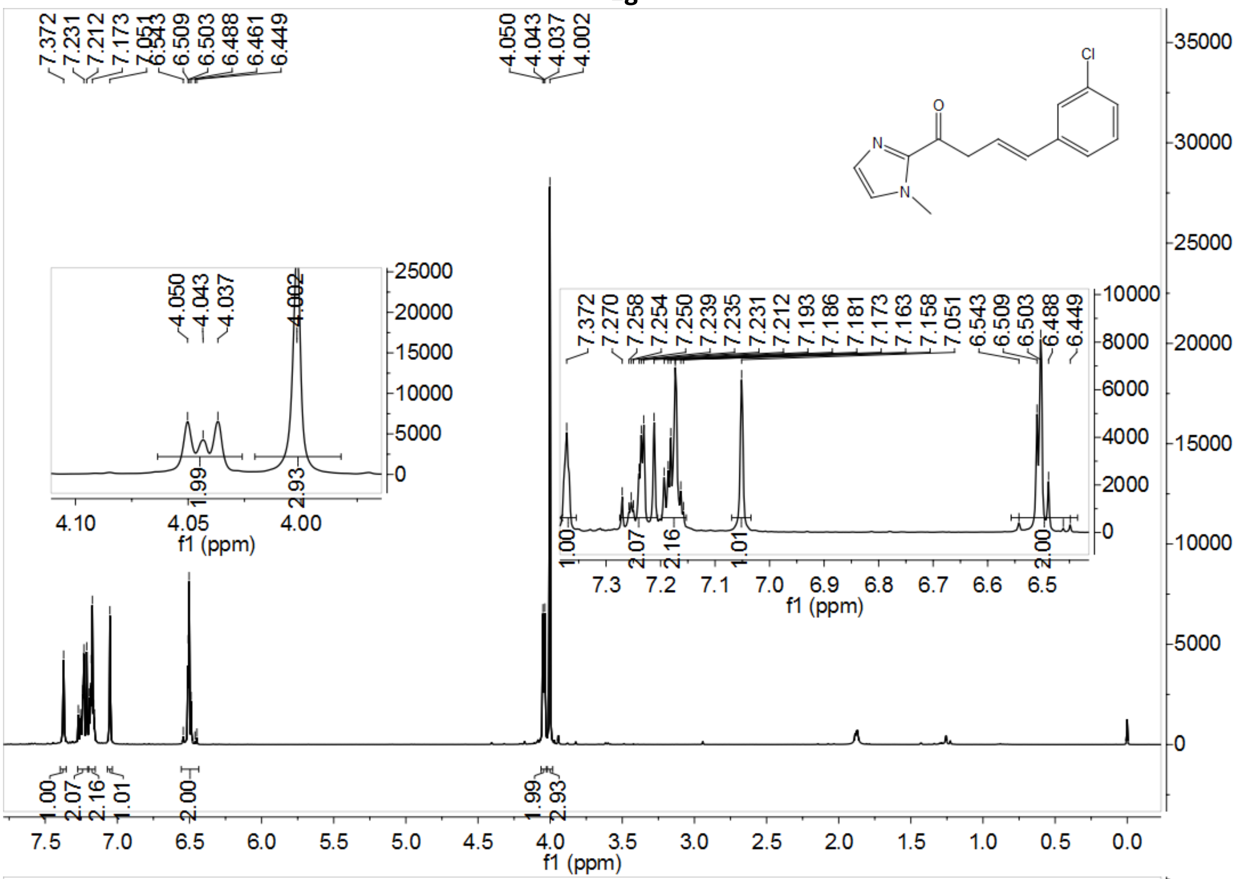

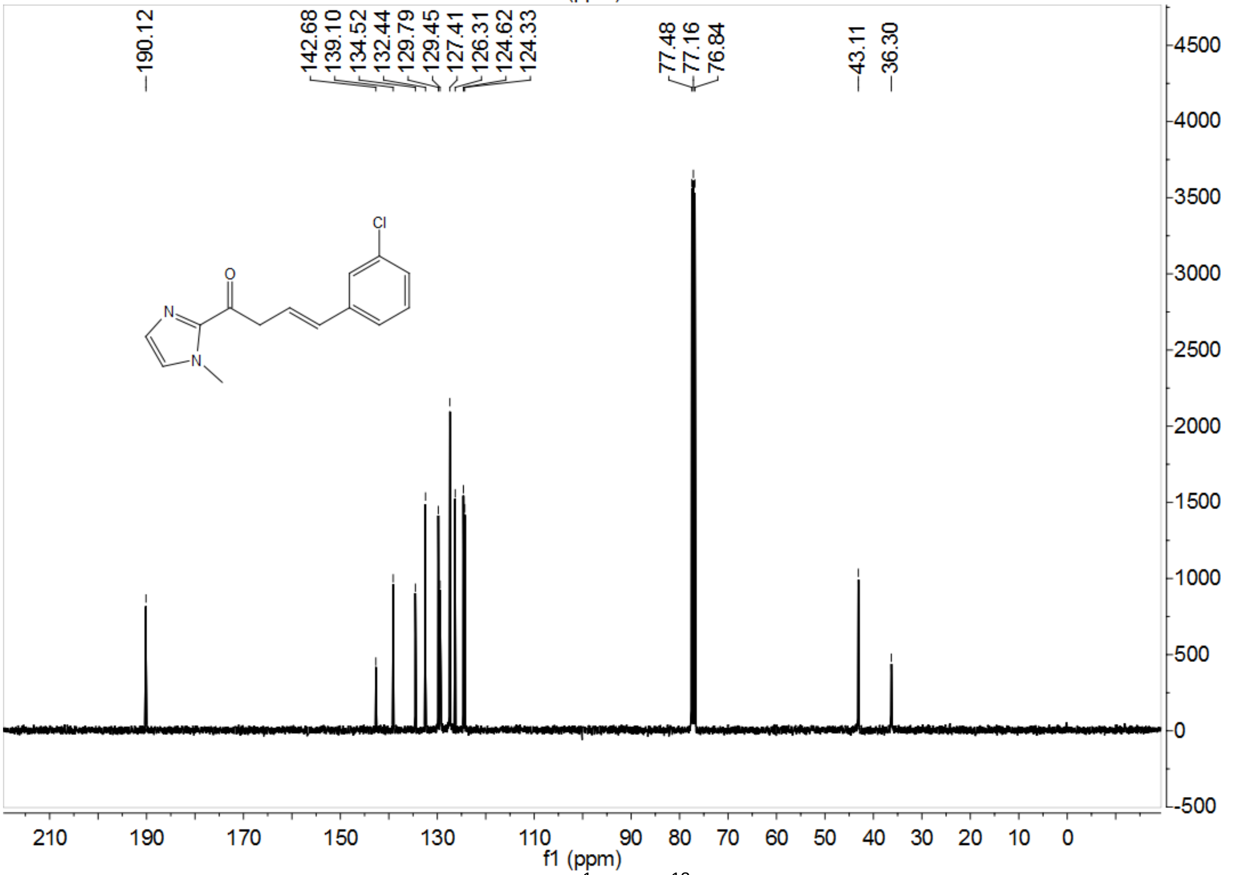

Supplementary Figure 12. ^{1}H and ^{13}C spectra for substrate **1g**

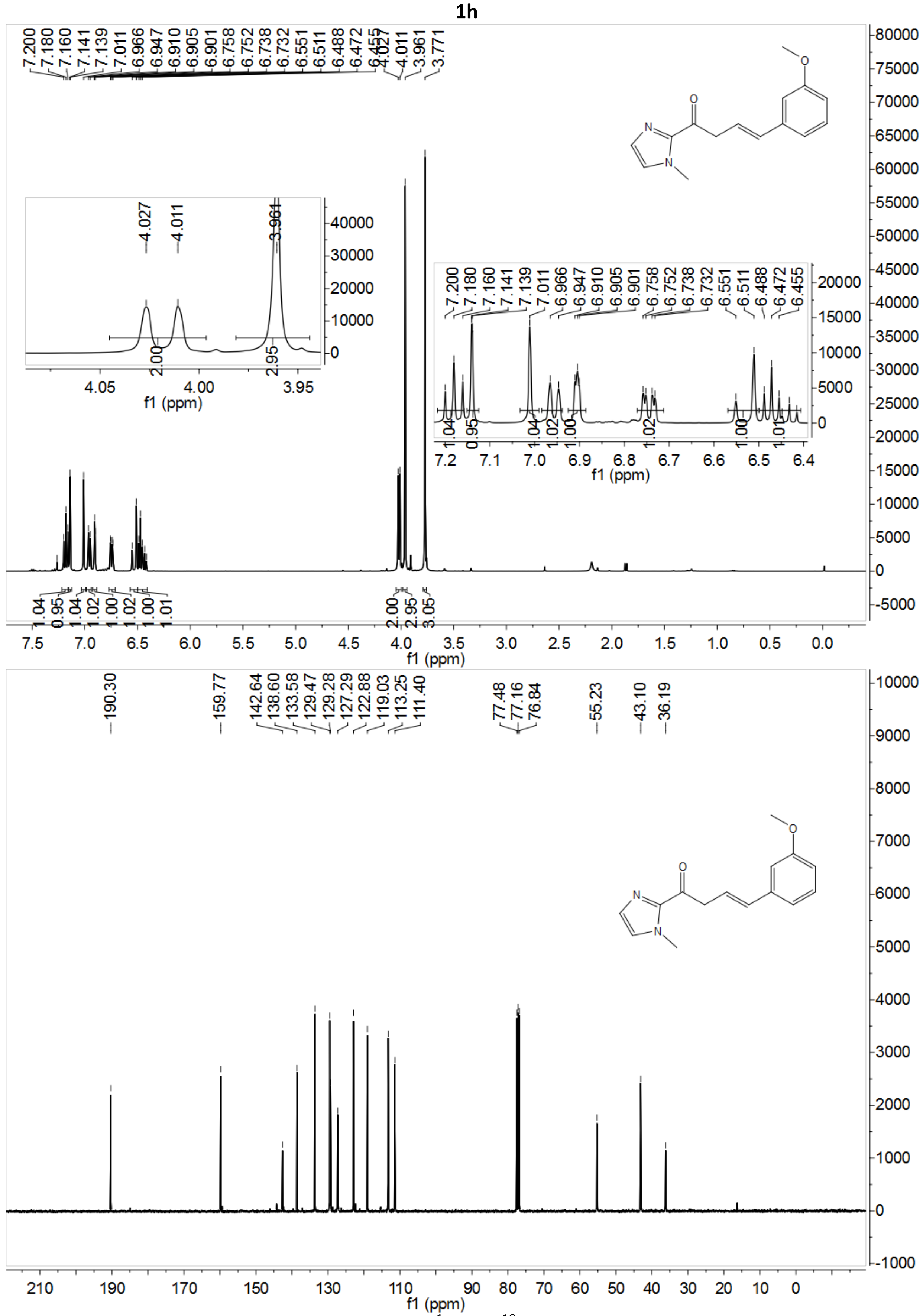

Supplementary Figure 13. ^{1}H and ^{13}C spectra for substrate **1h**

1i

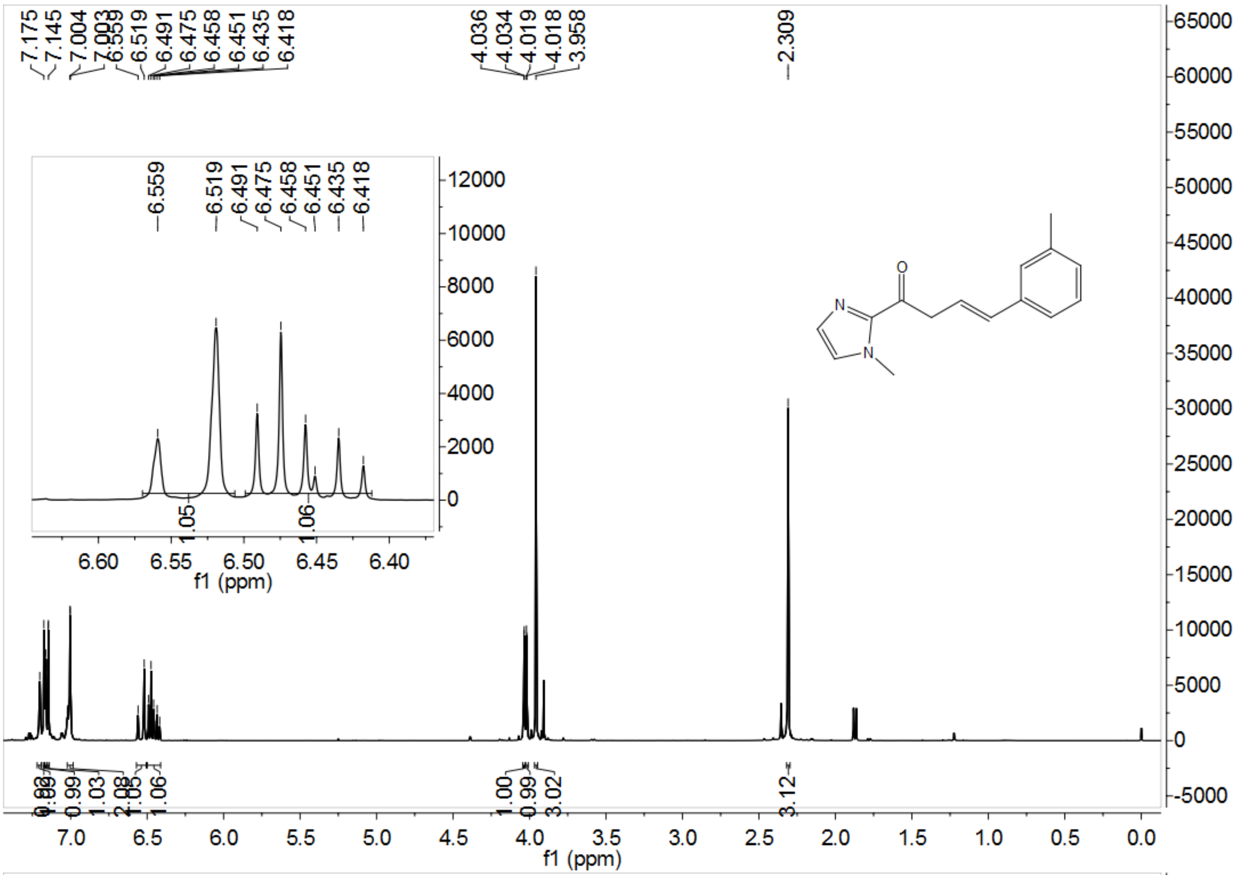

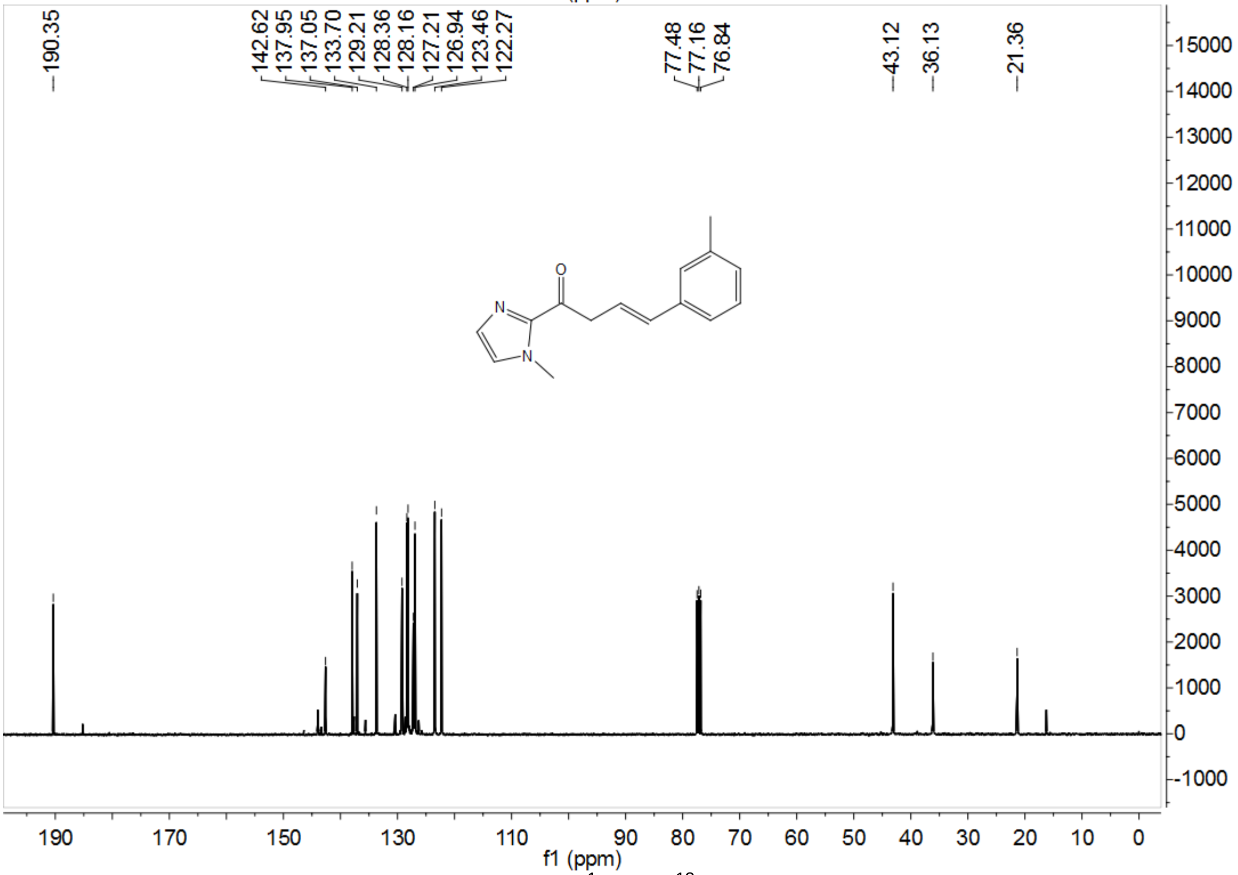

Supplementary Figure 14. ^{1}H and ^{13}C spectra for substrate **1i**

1j

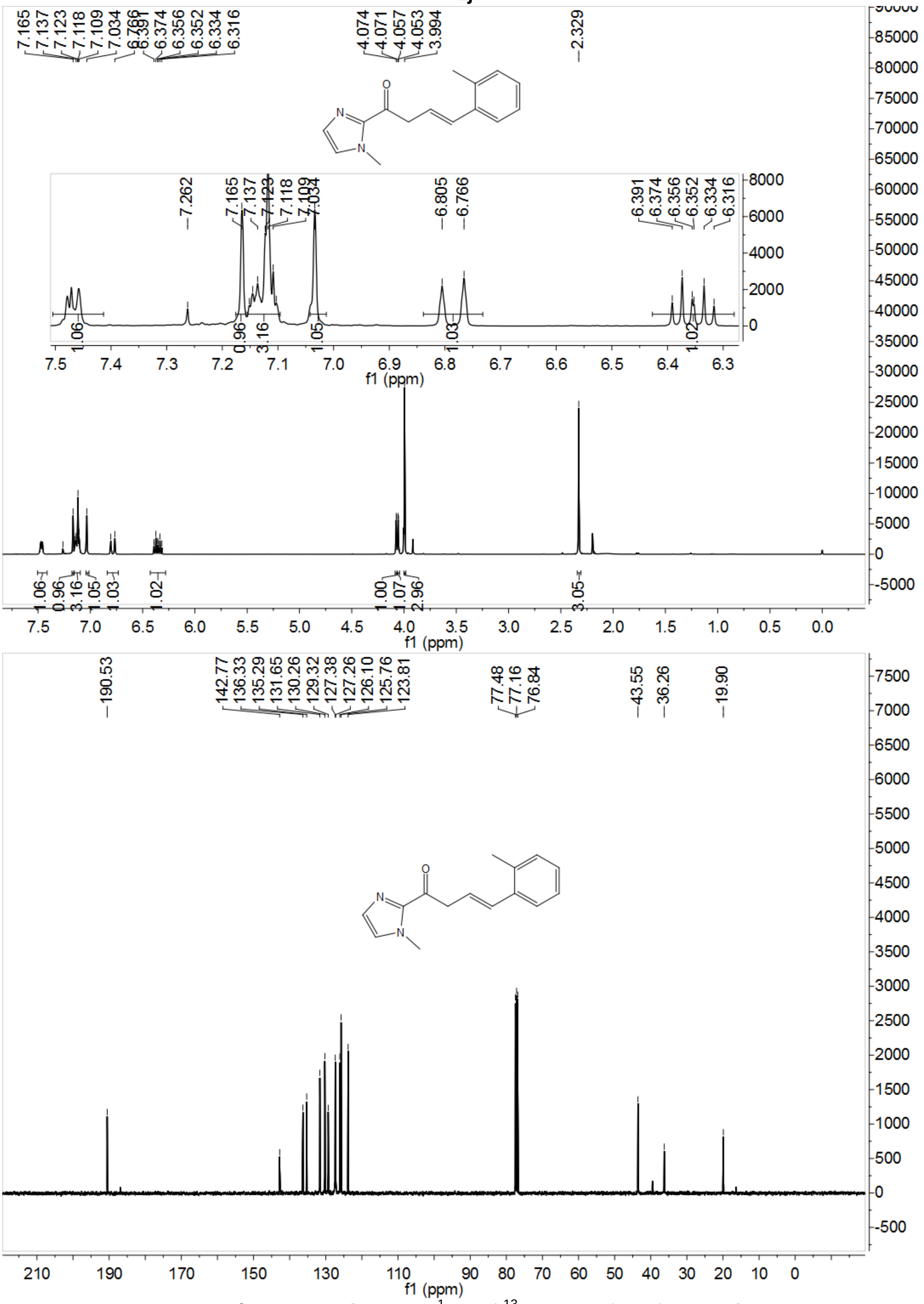

Supplementary Figure 15. ^{1}H and ^{13}C spectra for substrate **1j**

1k

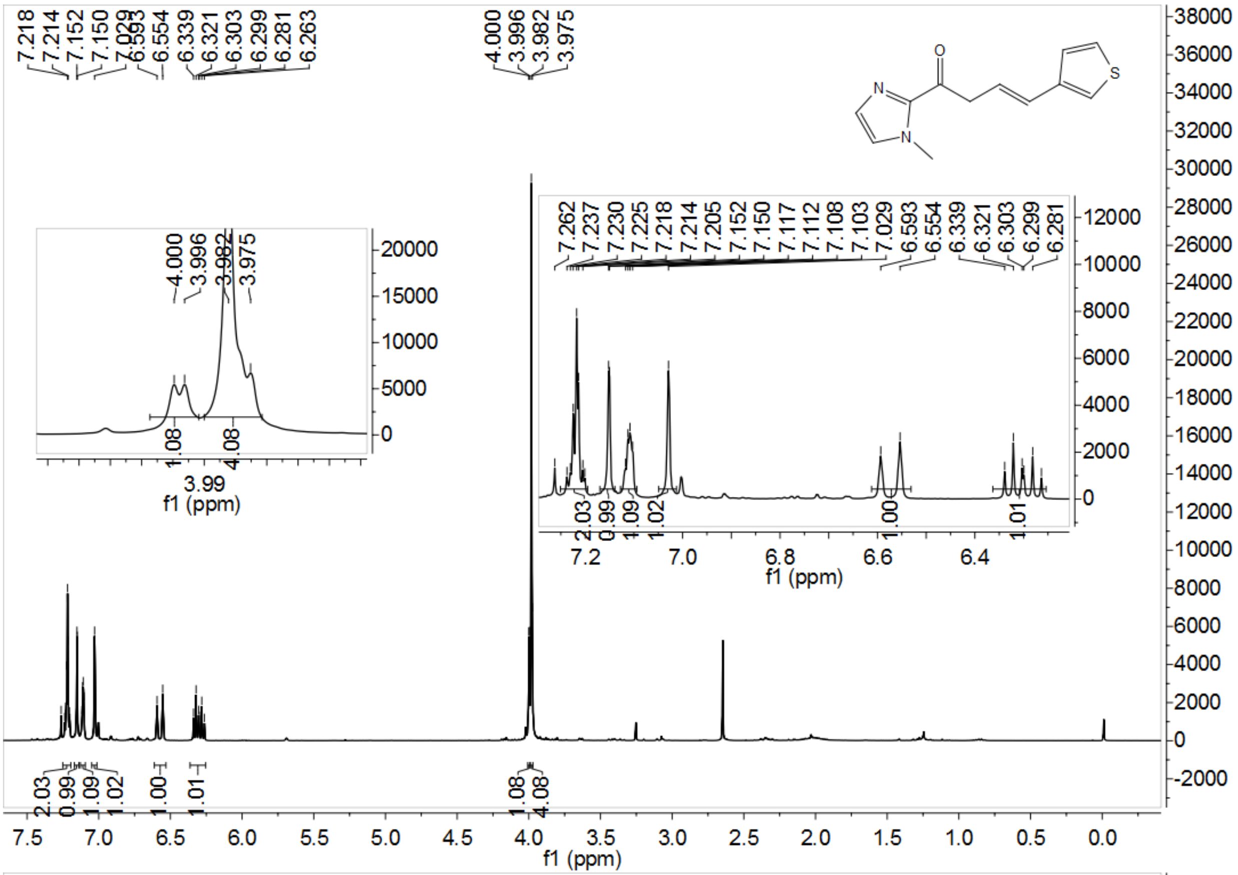

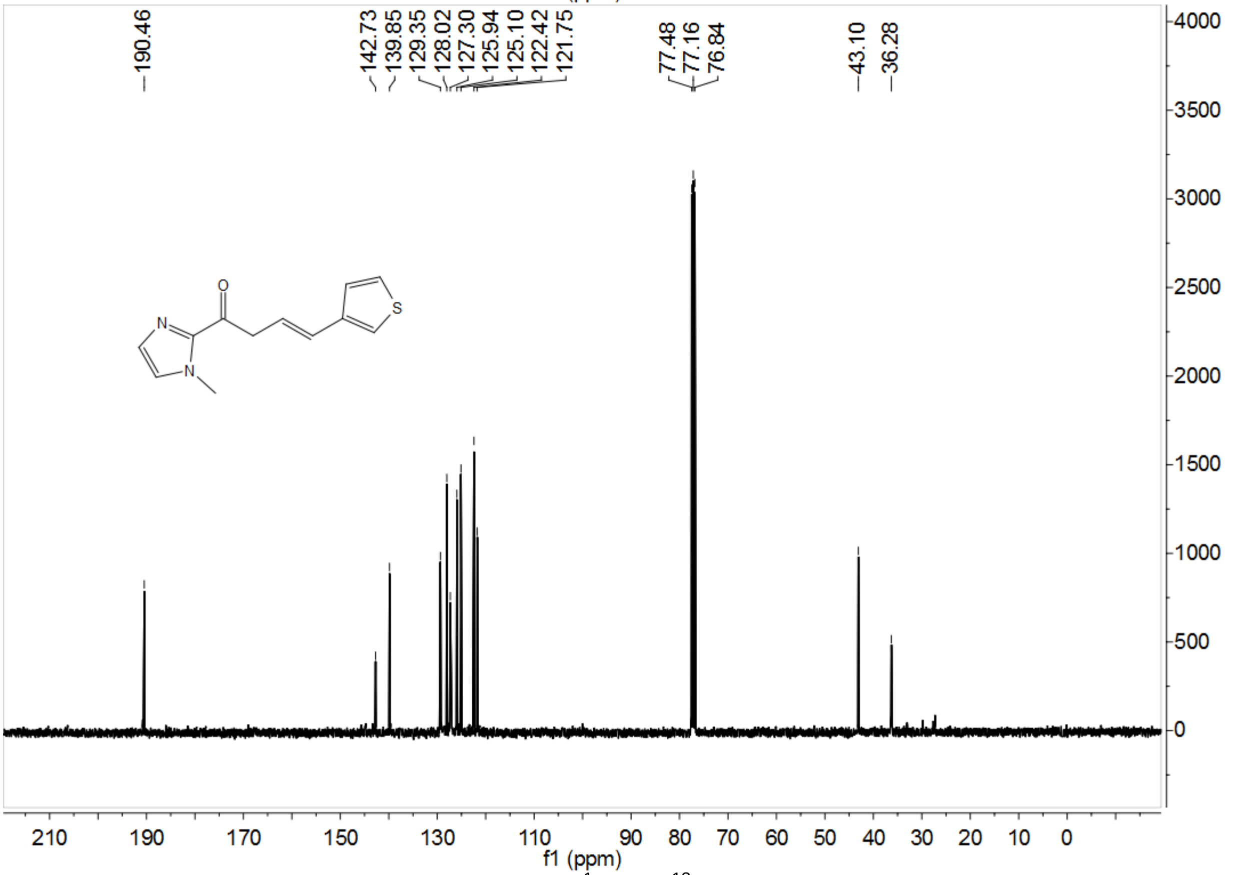

Supplementary Figure 16. ^{1}H and ^{13}C spectra for substrate **1k**

1l

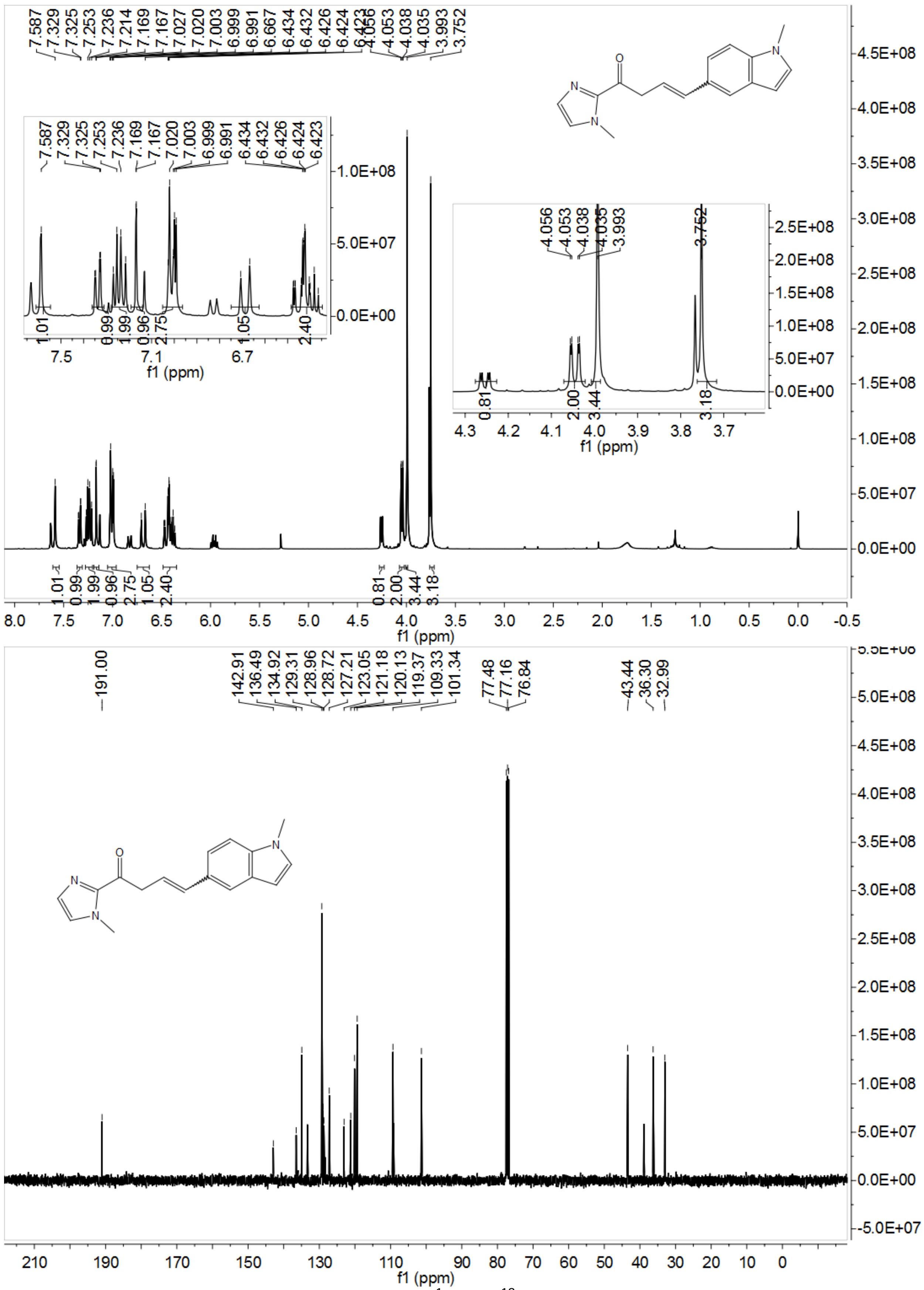

Supplementary Figure 17. ^{1}H and ^{13}C spectra for substrate **1l**

1m

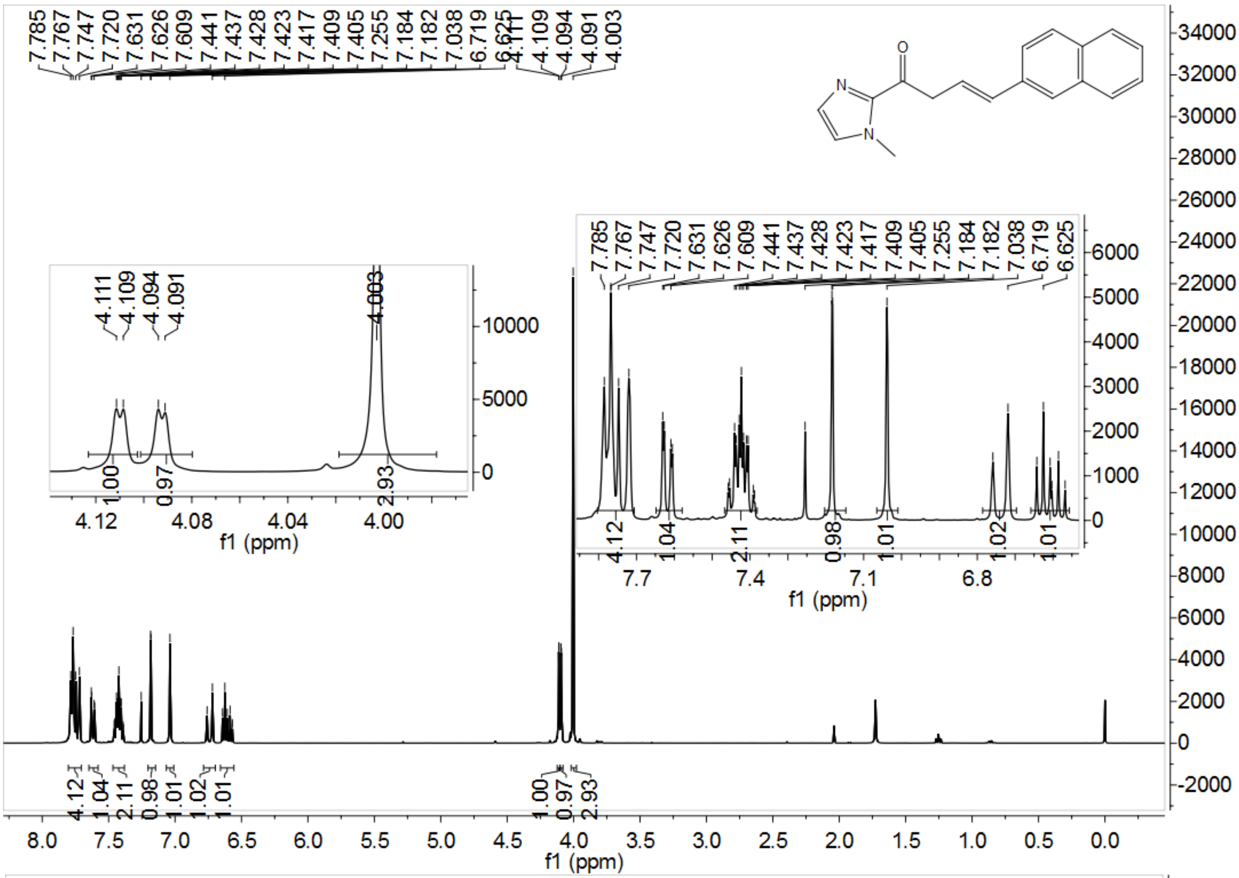

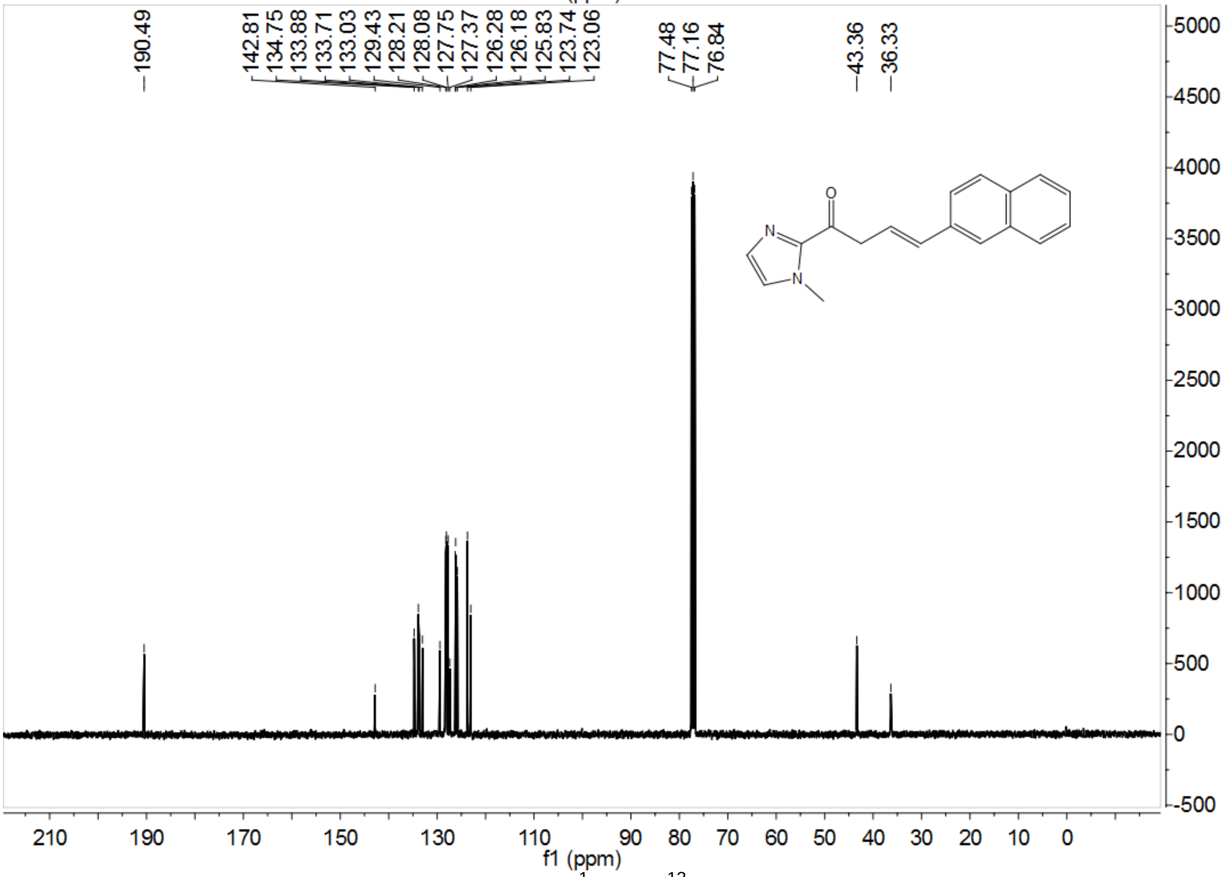

Supplementary Figure 18. ^{1}H and ^{13}C spectra for substrate **1m**

1n

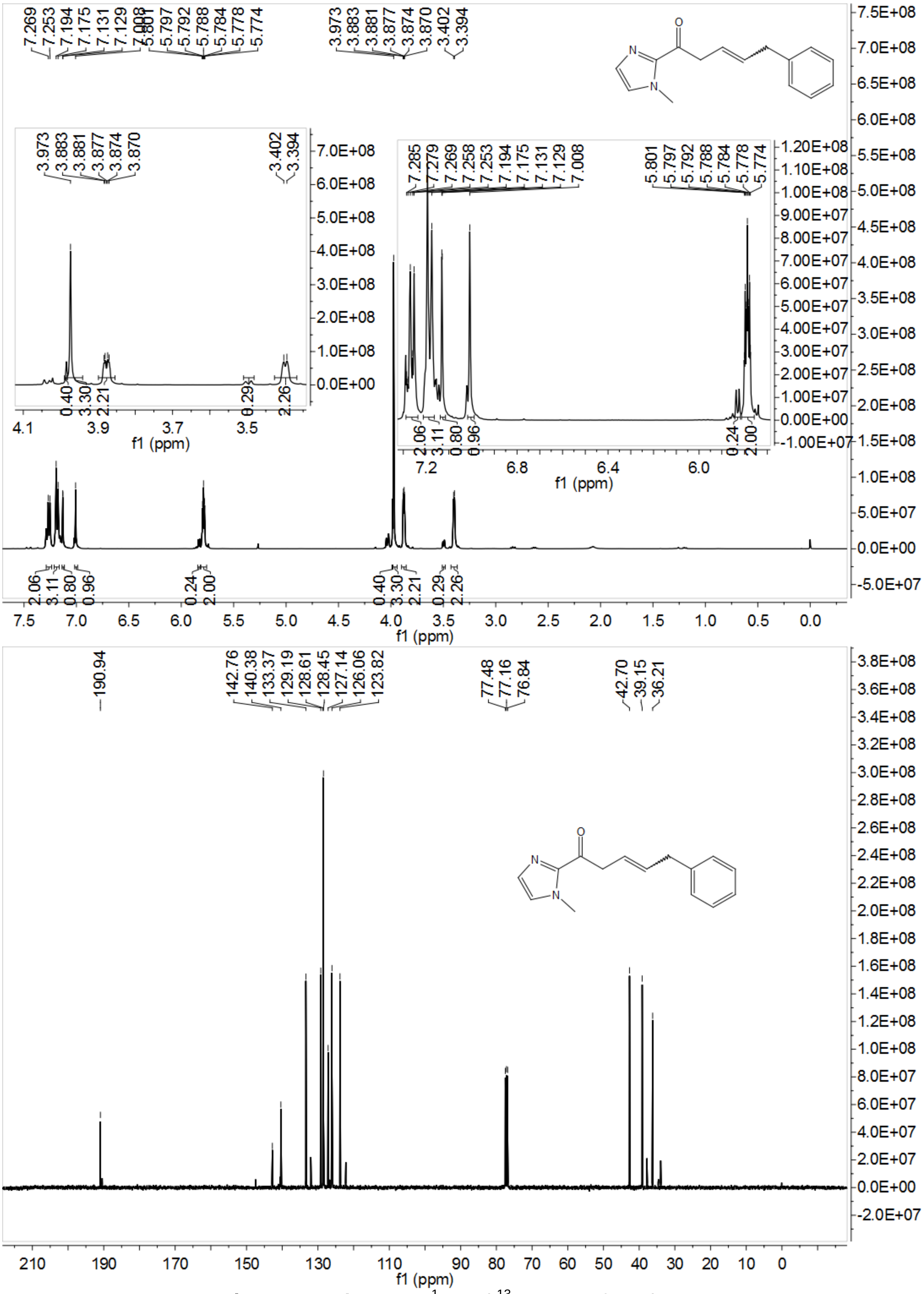

Supplementary Figure 19. ^{1}H and ^{13}C spectra for substrate **1n**

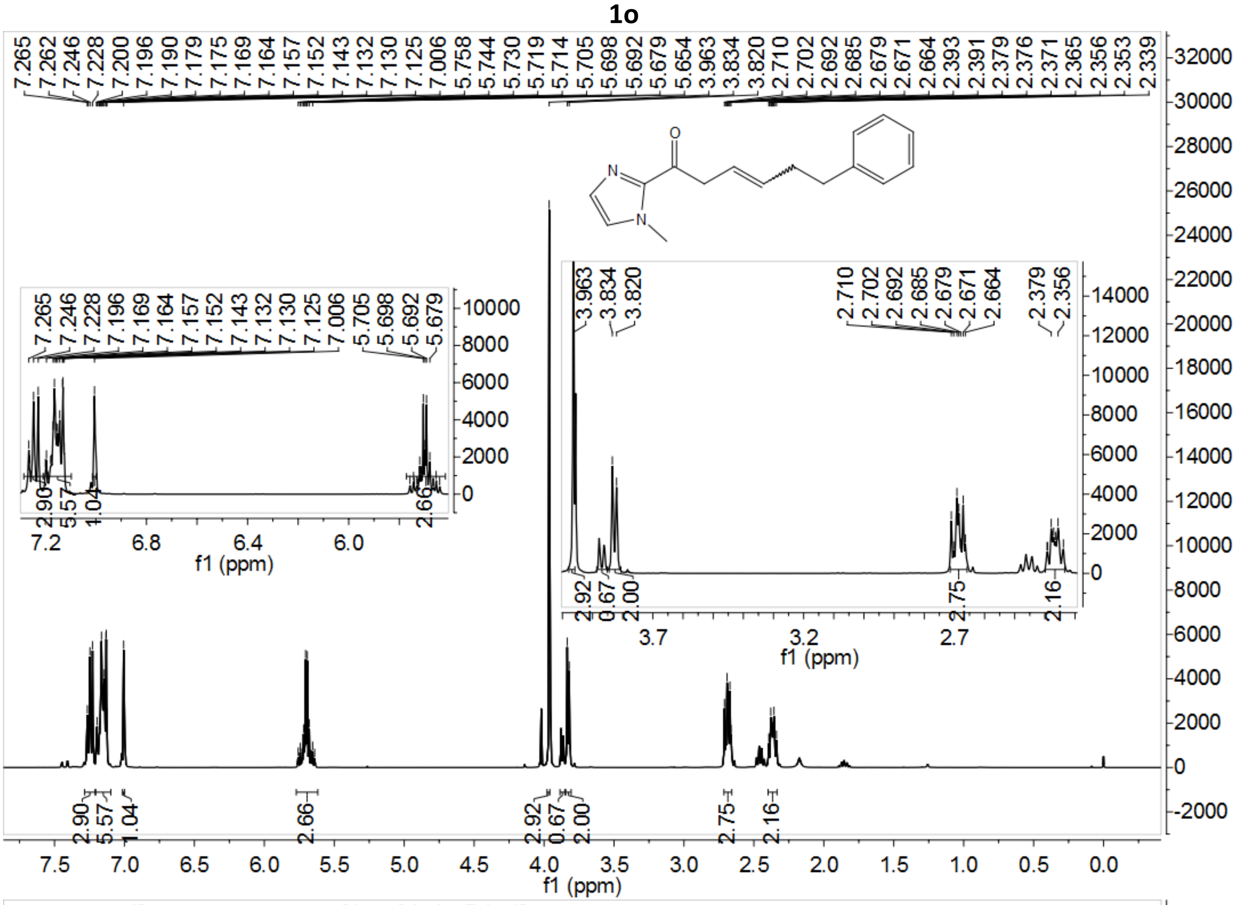

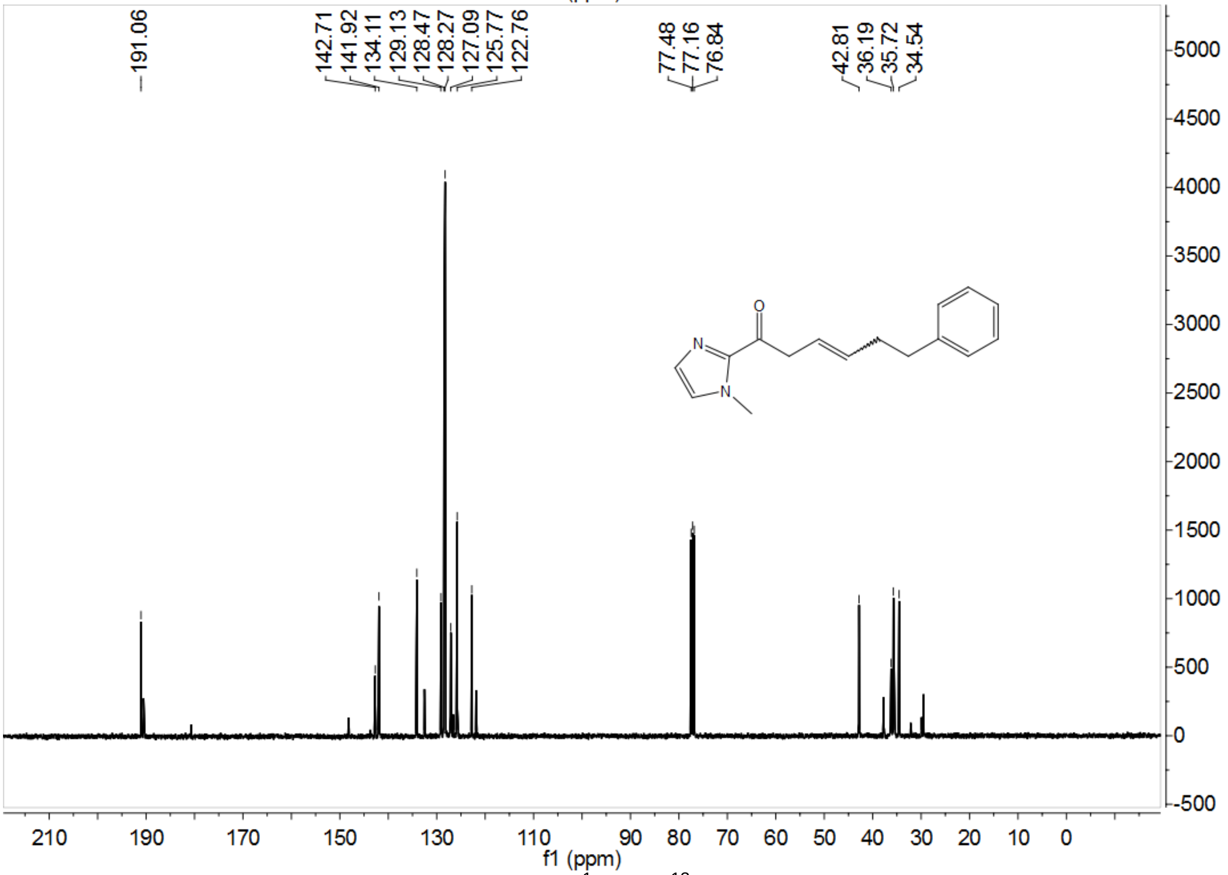

Supplementary Figure 20. ^{1}H and ^{13}C spectra for substrate **1o**

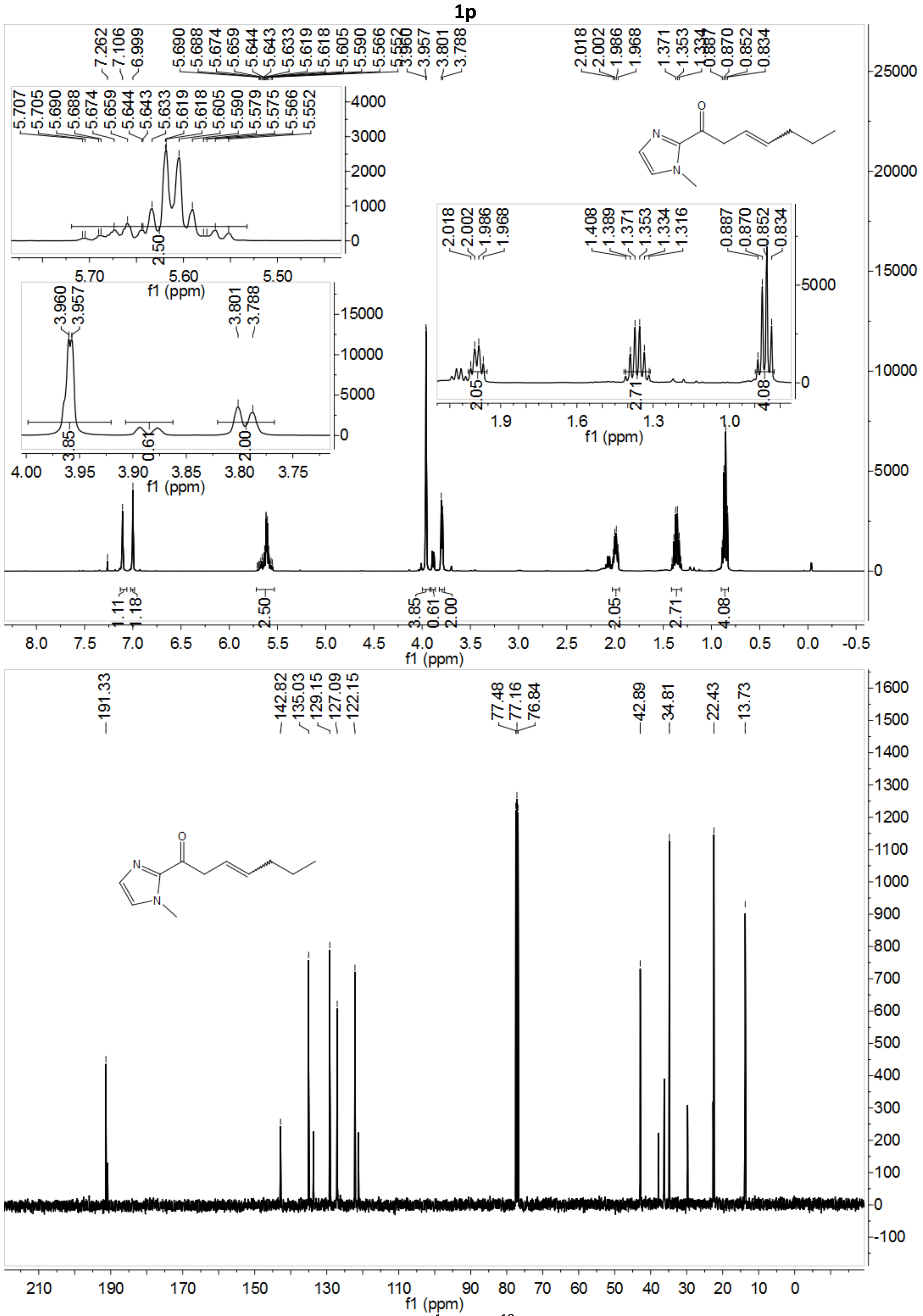

Supplementary Figure 21. ^{1}H and ^{13}C spectra for substrate **1p**

1q

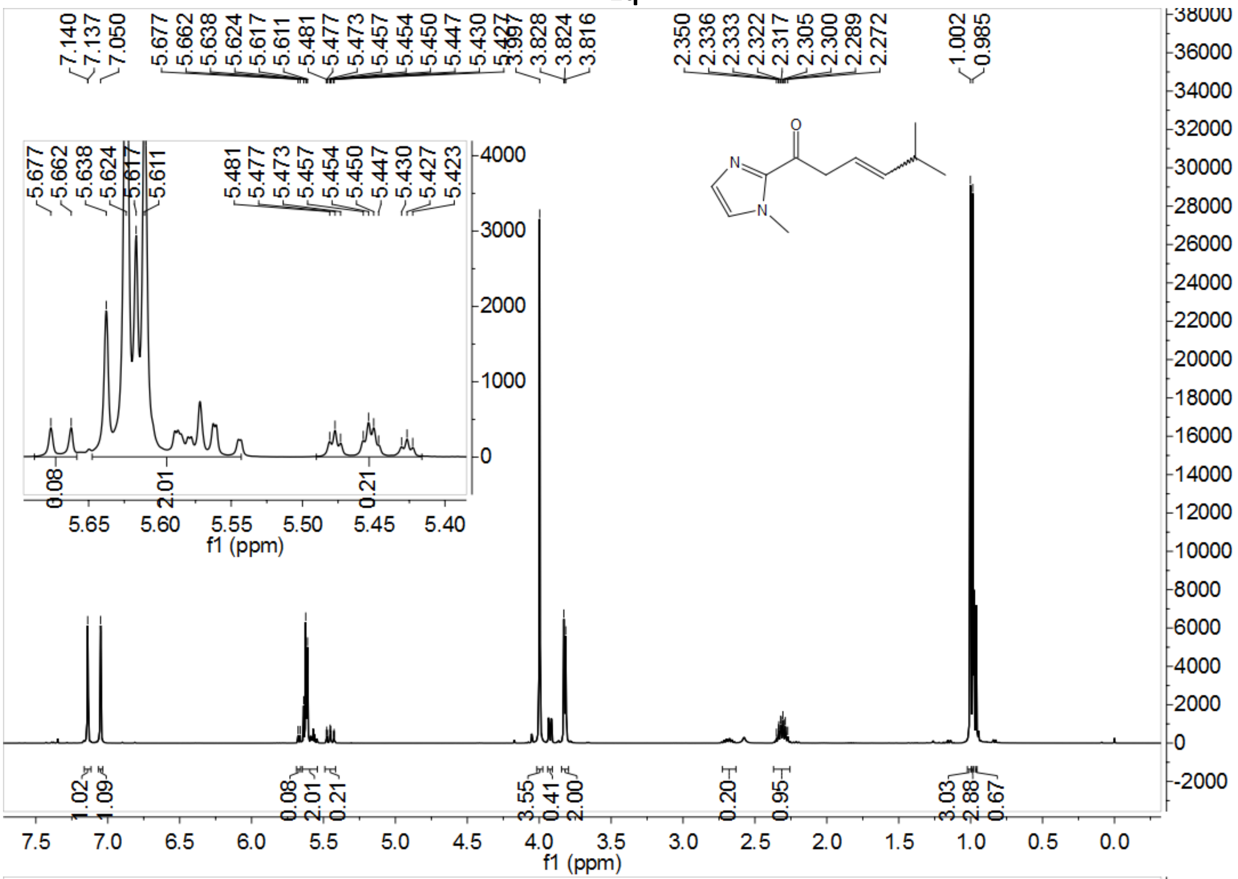

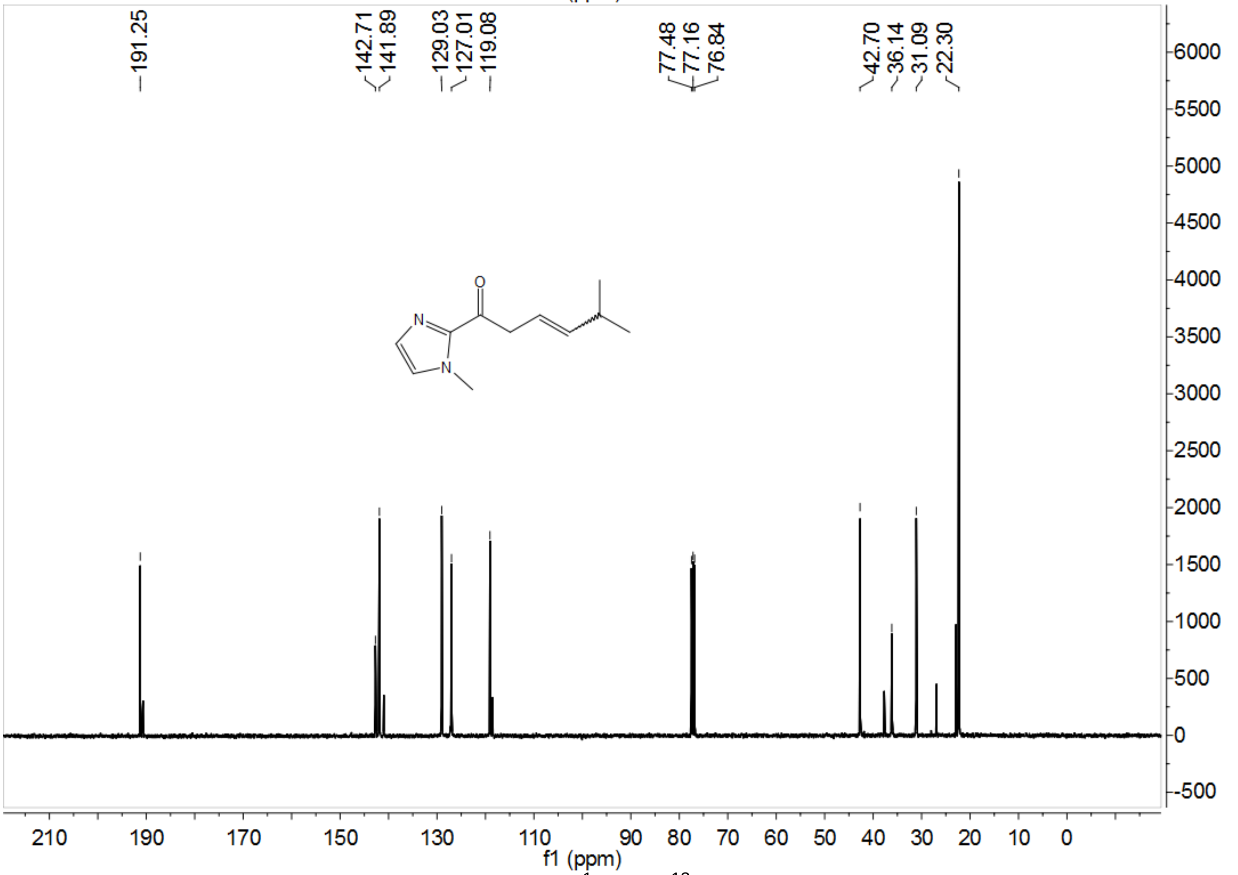

Supplementary Figure 22. ^{1}H and ^{13}C spectra for substrate **1q**

1r

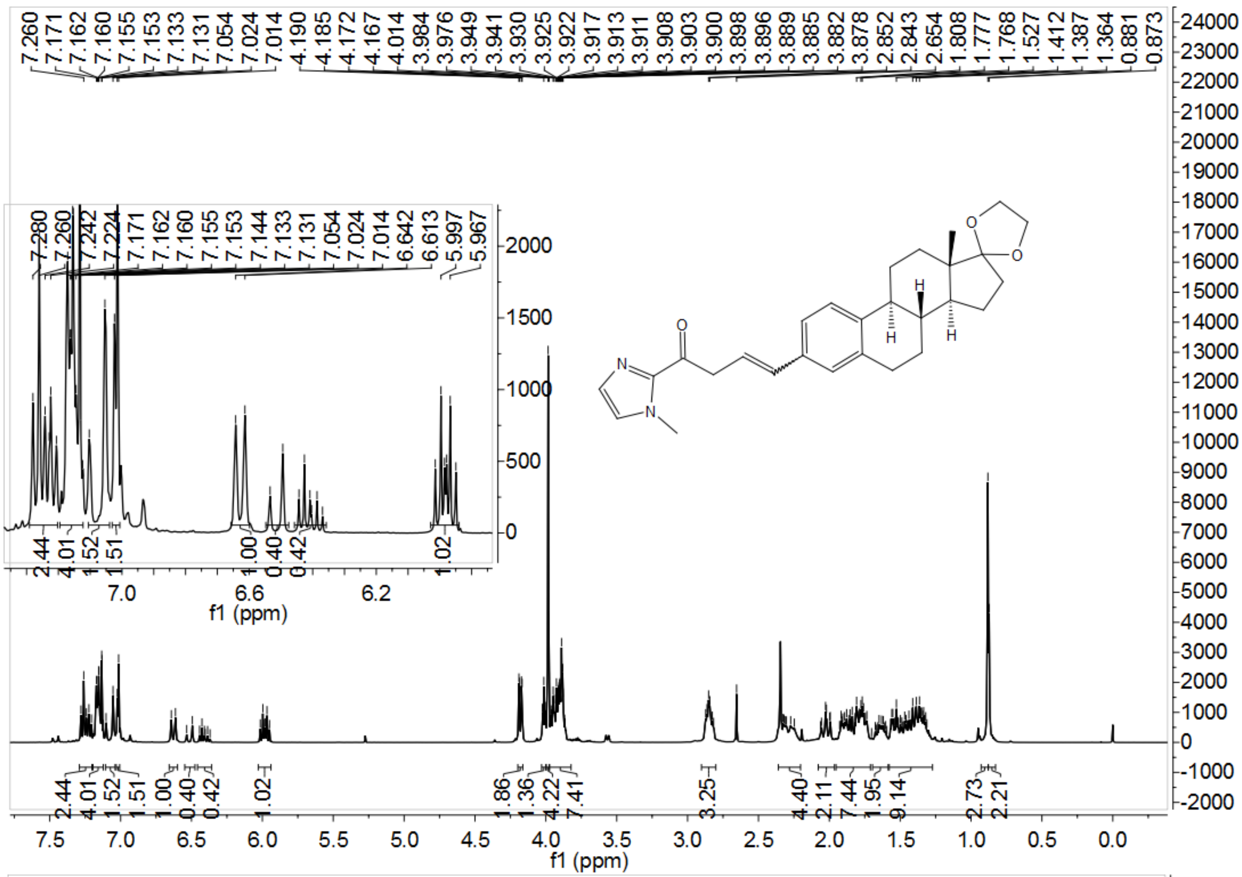

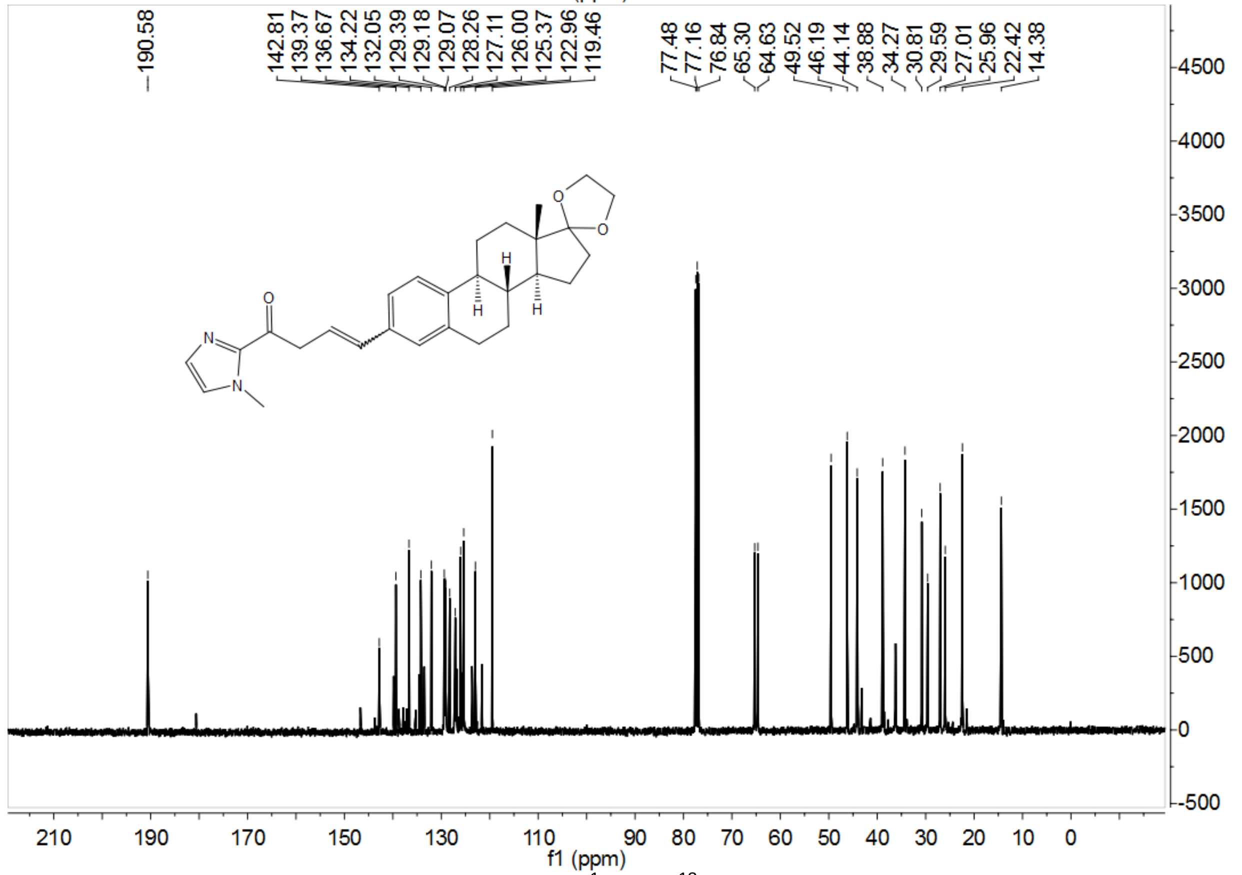

Supplementary Figure 23. ^{1}H and ^{13}C spectra for substrate **1r**

1s

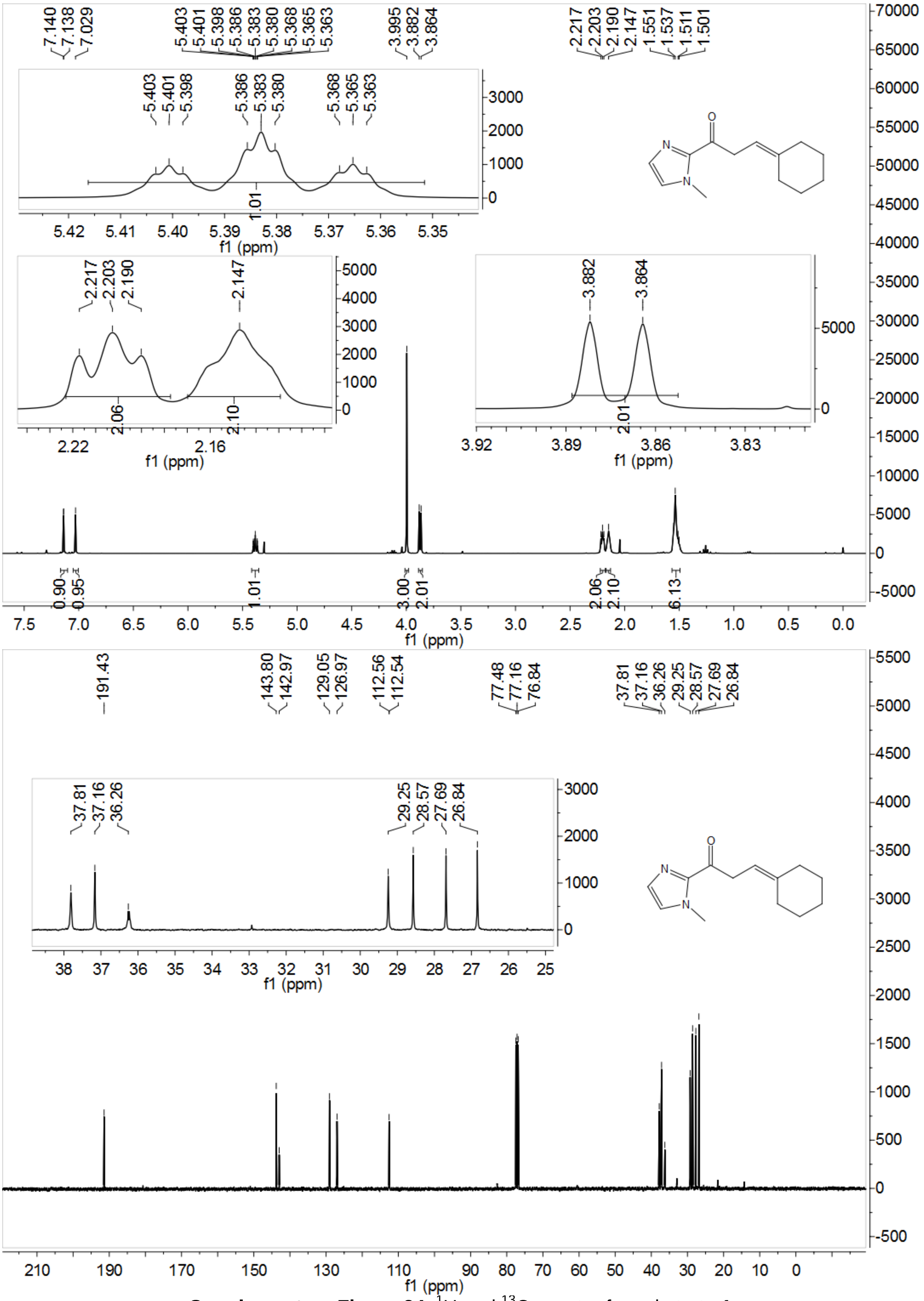

Supplementary Figure 24. ^{1}H and ^{13}C spectra for substrate **1s**

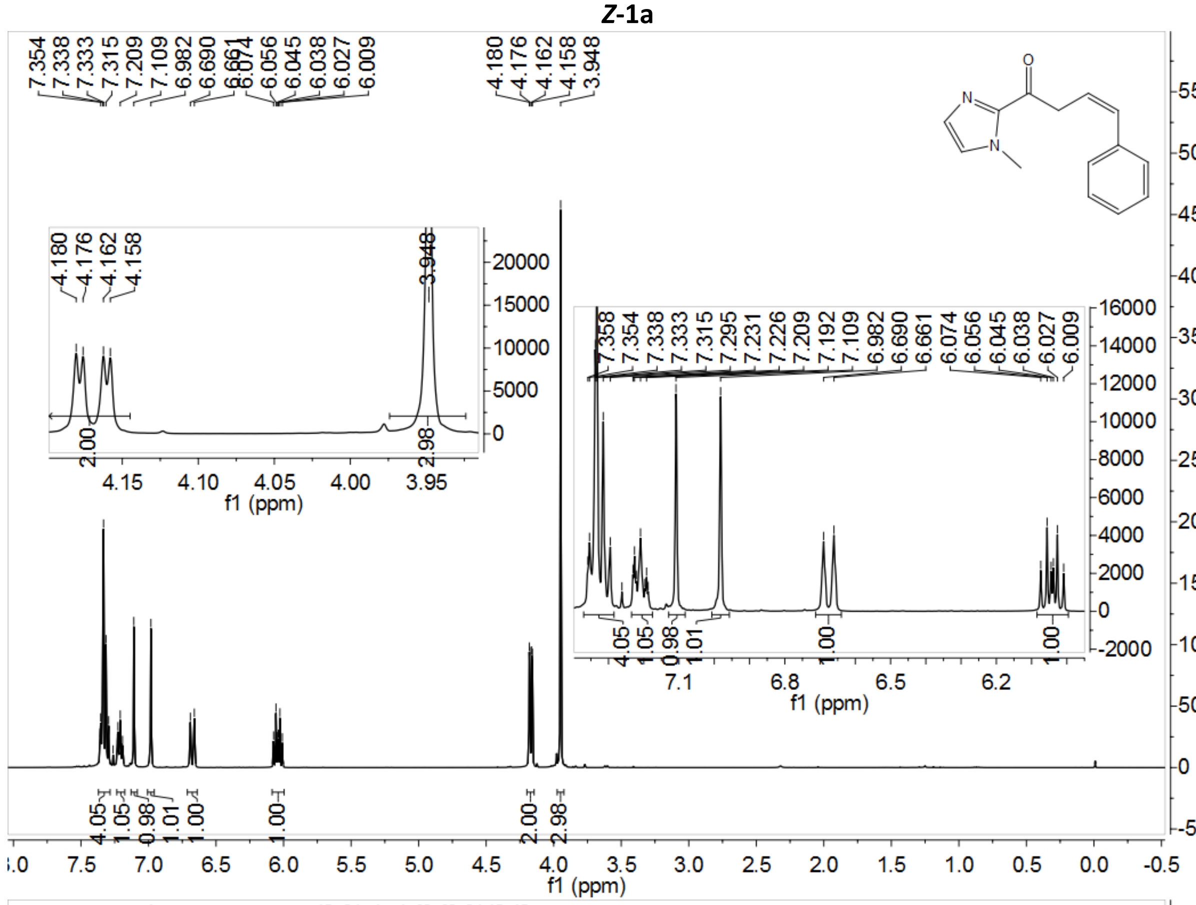

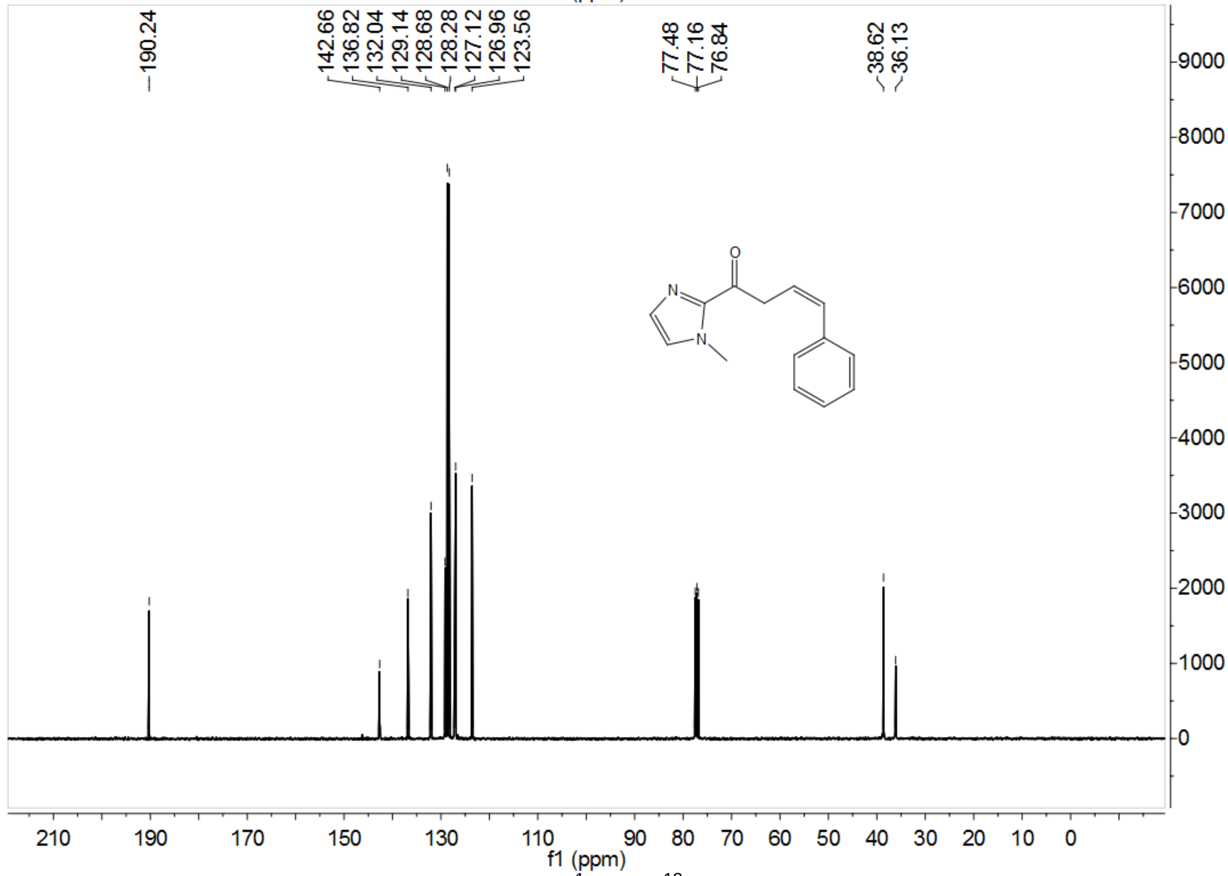

Supplementary Figure 25. ^{1}H and ^{13}C spectra for substrate ***Z*-1a**

***E*-10**

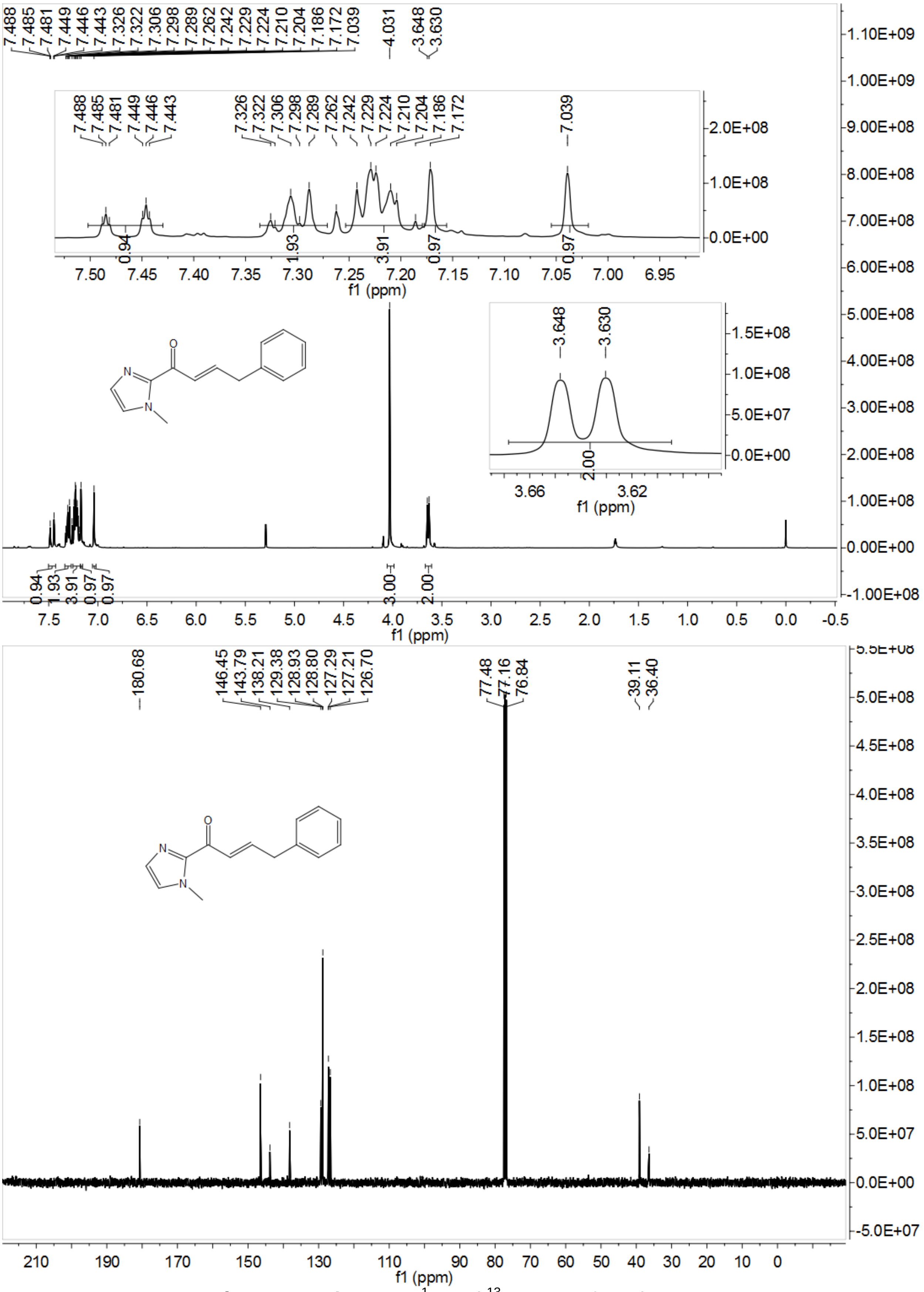

Supplementary Figure 26. ^{1}H and ^{13}C spectra for substrate ***E*-10**

Z-10

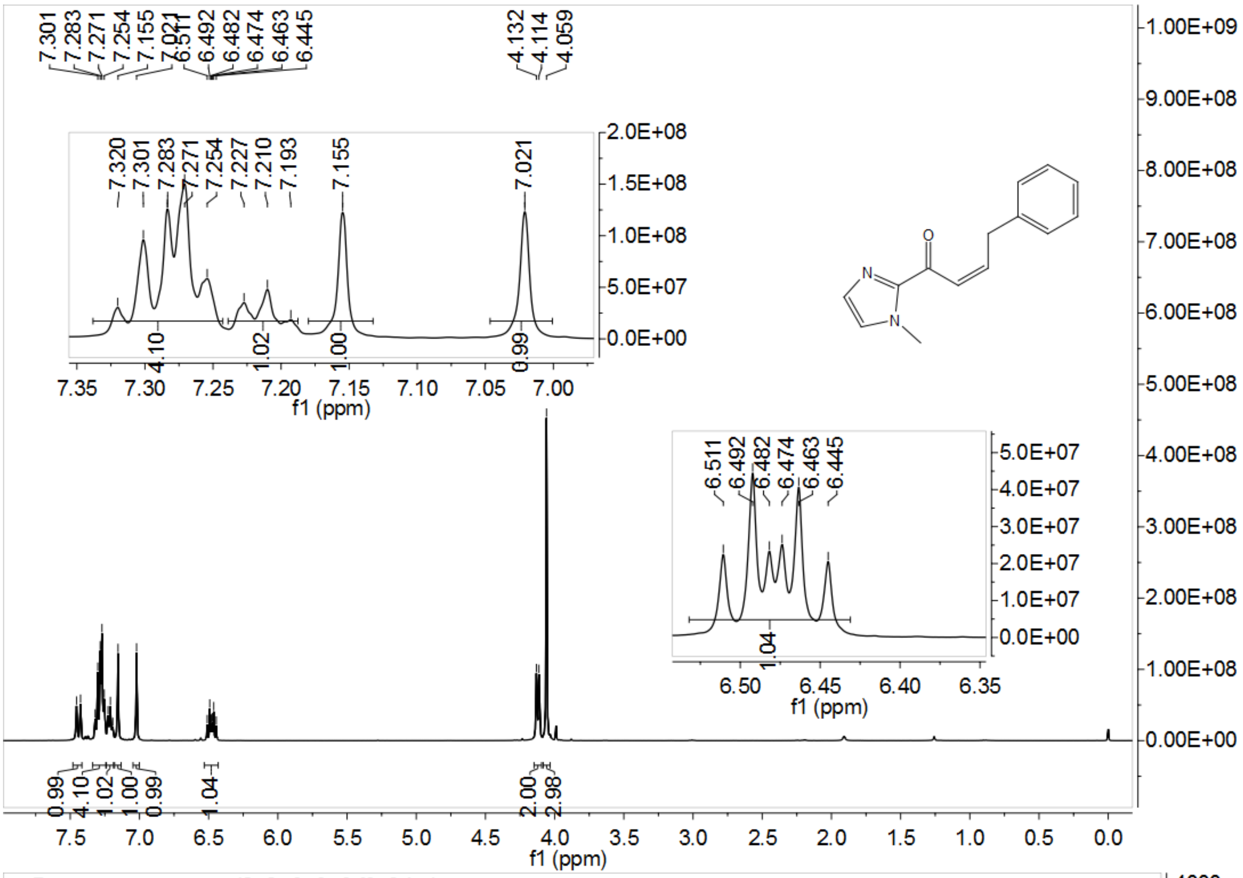

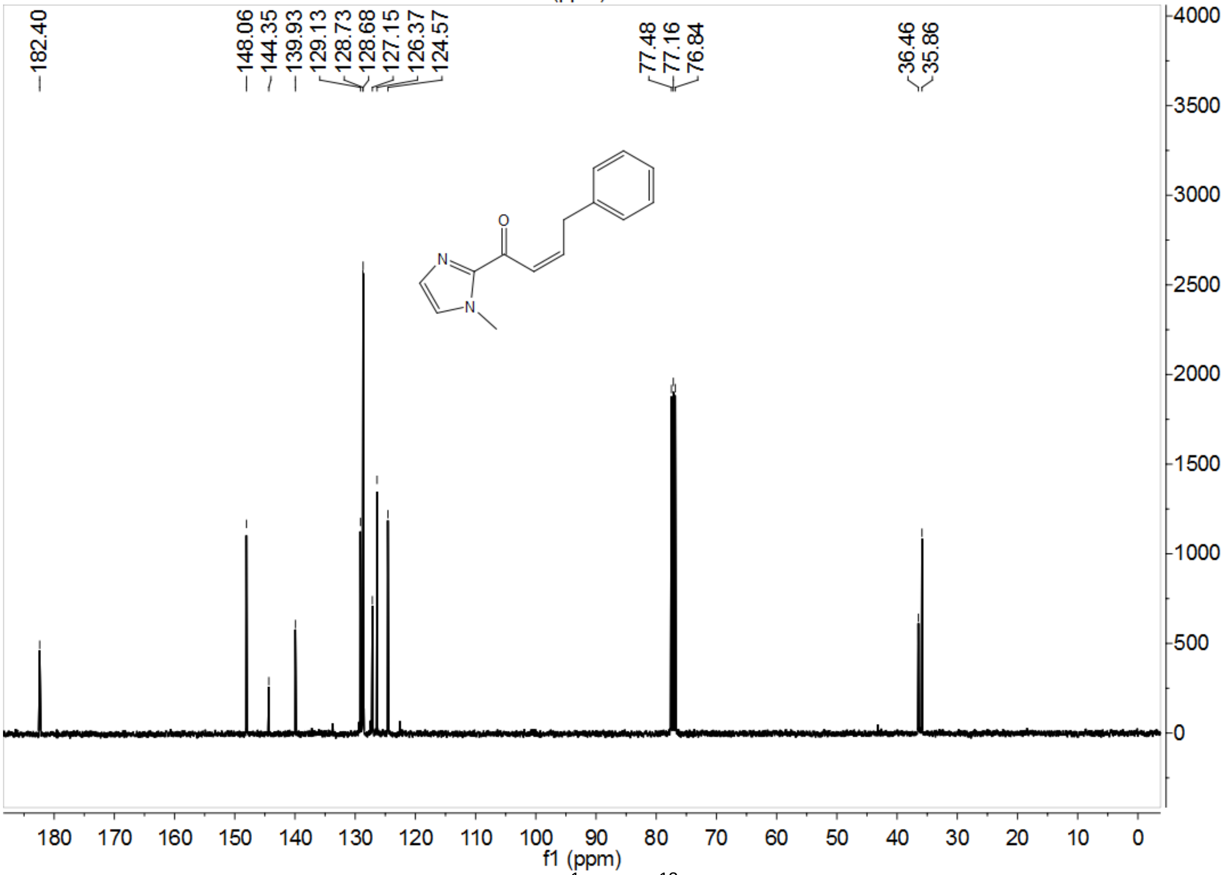

Supplementary Figure 27. ^{1}H and ^{13}C spectra for substrate ***Z*-10**

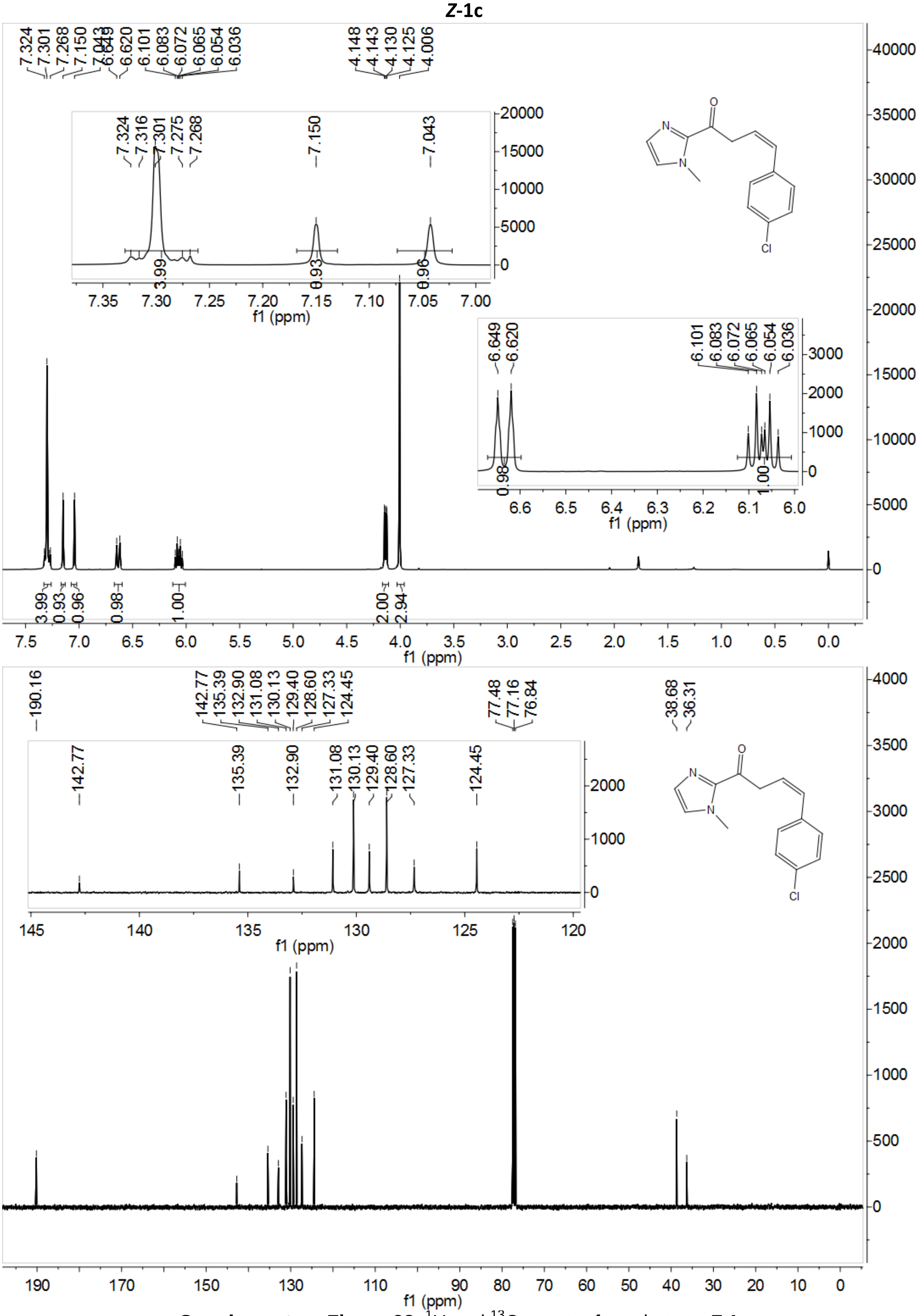

Supplementary Figure 28. ^{1}H and ^{13}C spectra for substrate ***Z*-1c**

***Z*-1d**

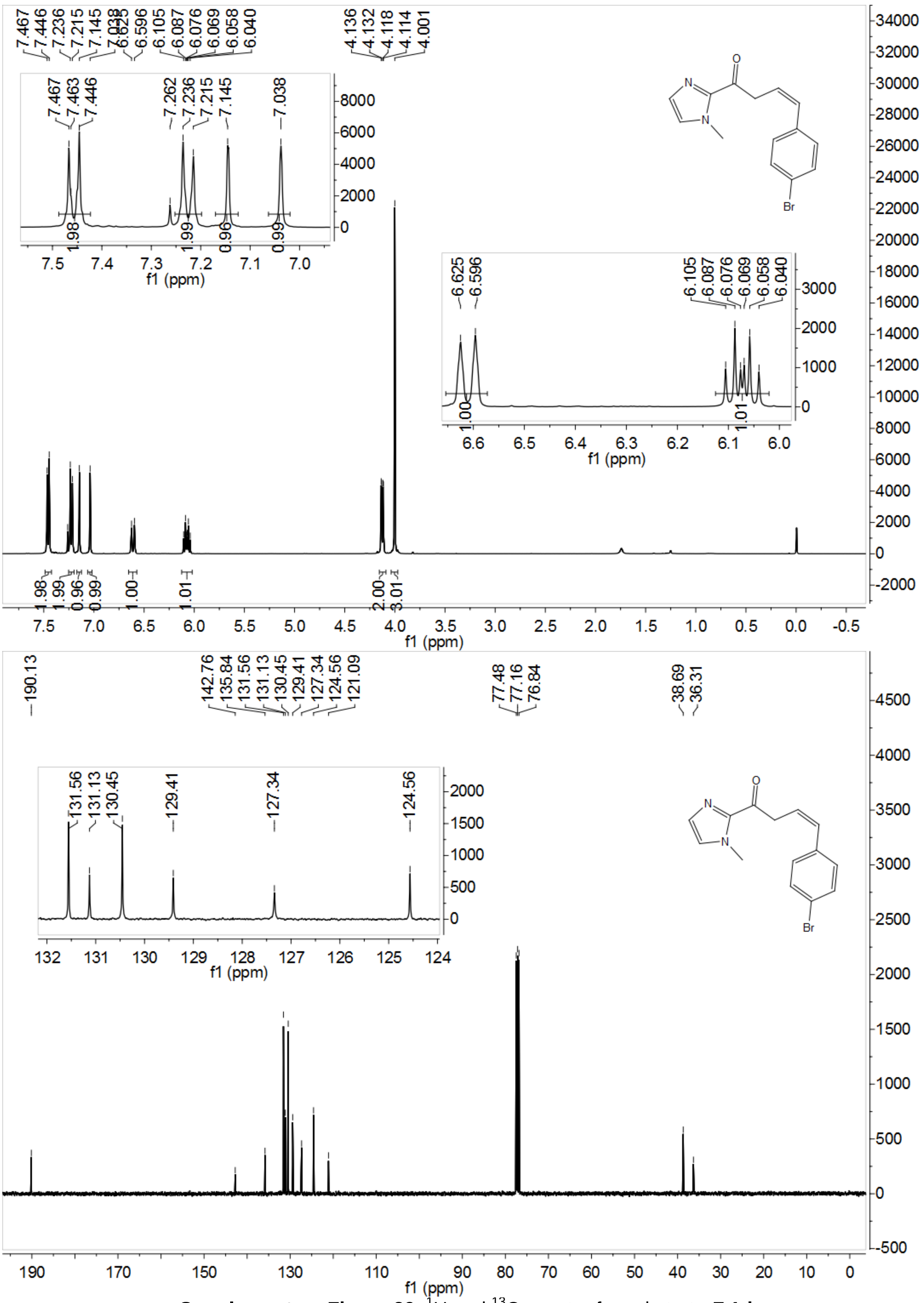

Supplementary Figure 29. ^{1}H and ^{13}C spectra for substrate ***Z*-1d**

***Z*-1h**

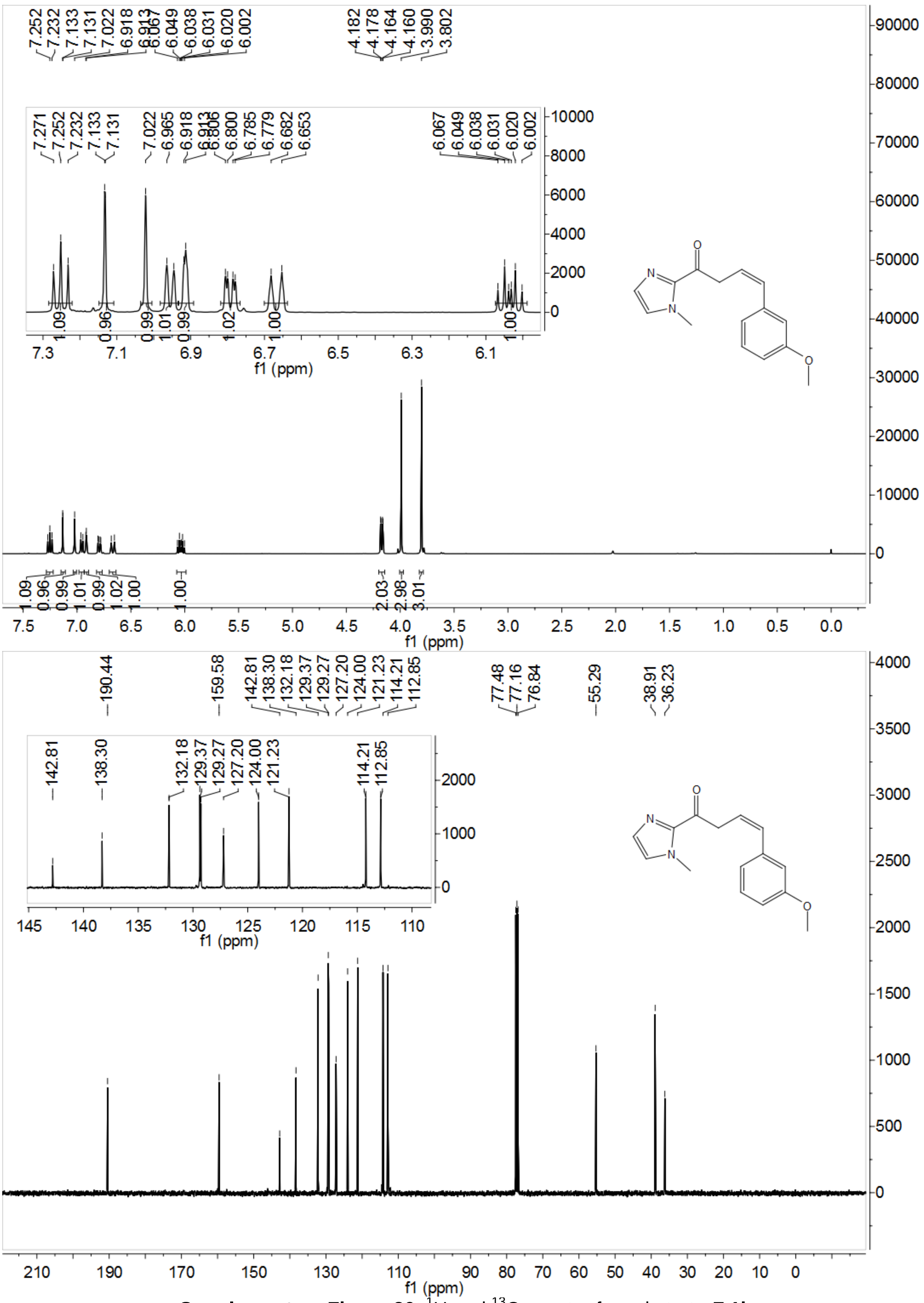

Supplementary Figure 30. ^{1}H and ^{13}C spectra for substrate ***Z*-1h**

***Z*-1i**

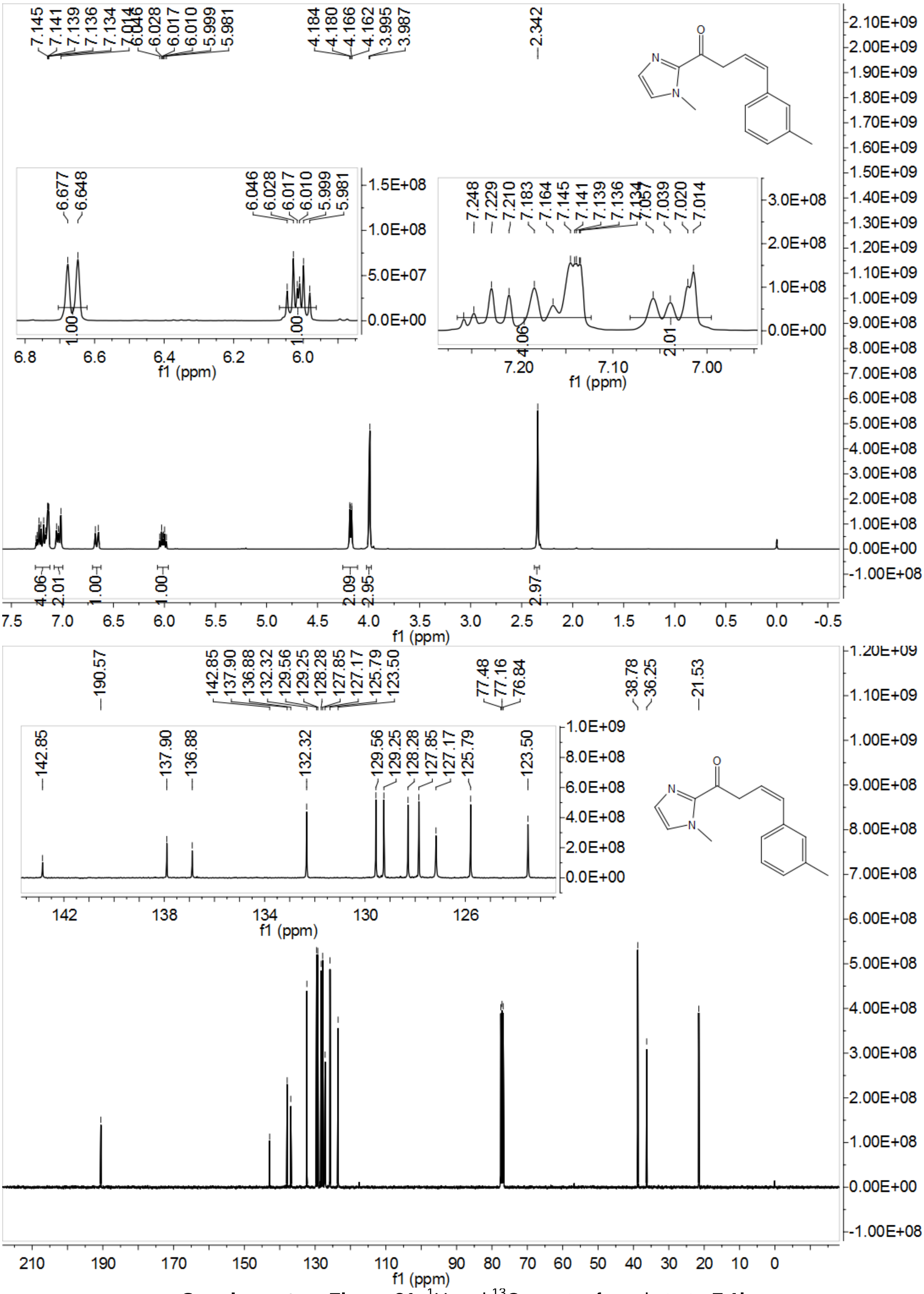

Supplementary Figure 31. ^{1}H and ^{13}C spectra for substrate ***Z*-1i**

(2*S*,3*R*)-2a

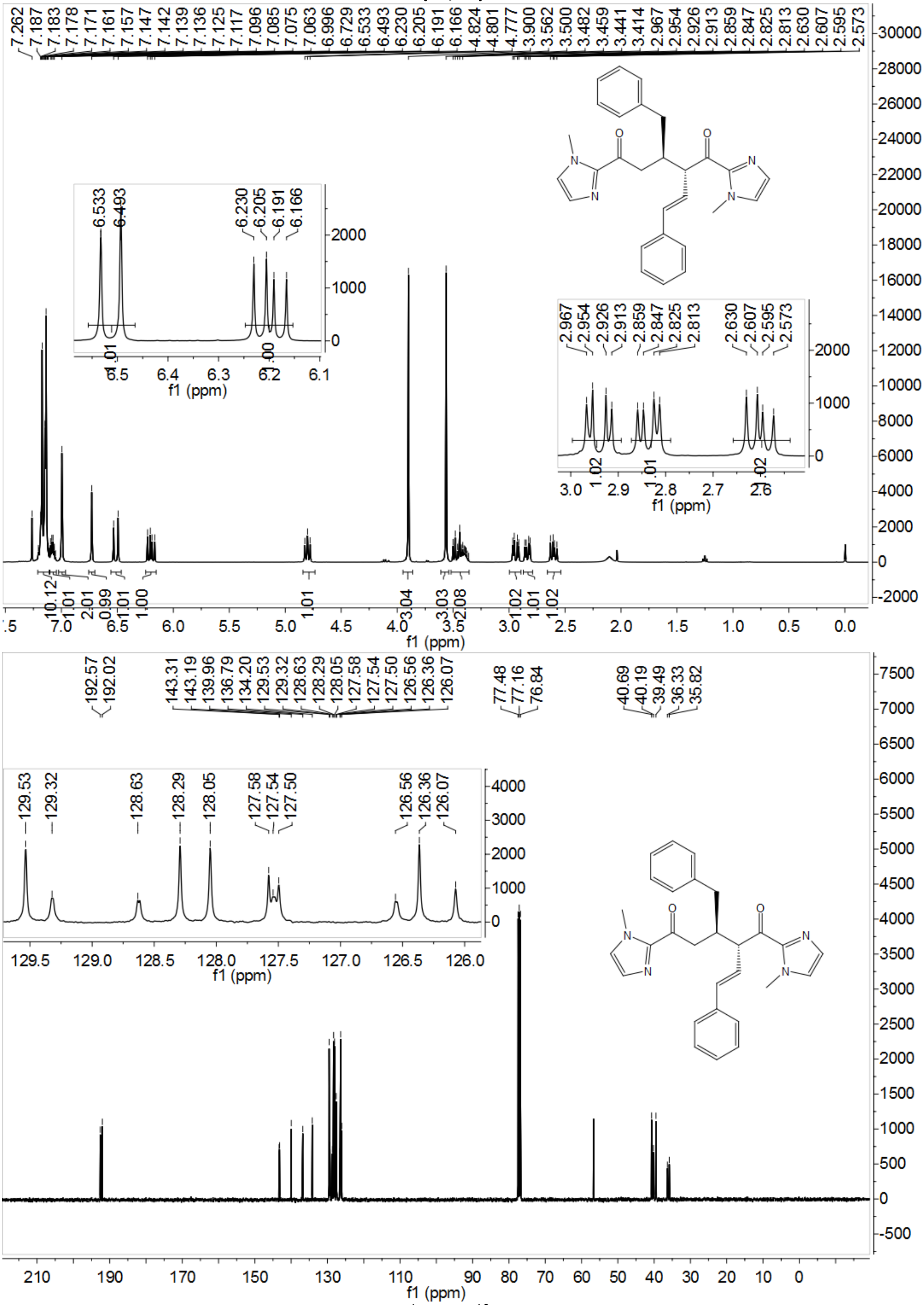

Supplementary Figure 32. ^{1}H and ^{13}C spectra for product **(2*S*,3*R*)-2a**

(2*R*,3*S*)-2a

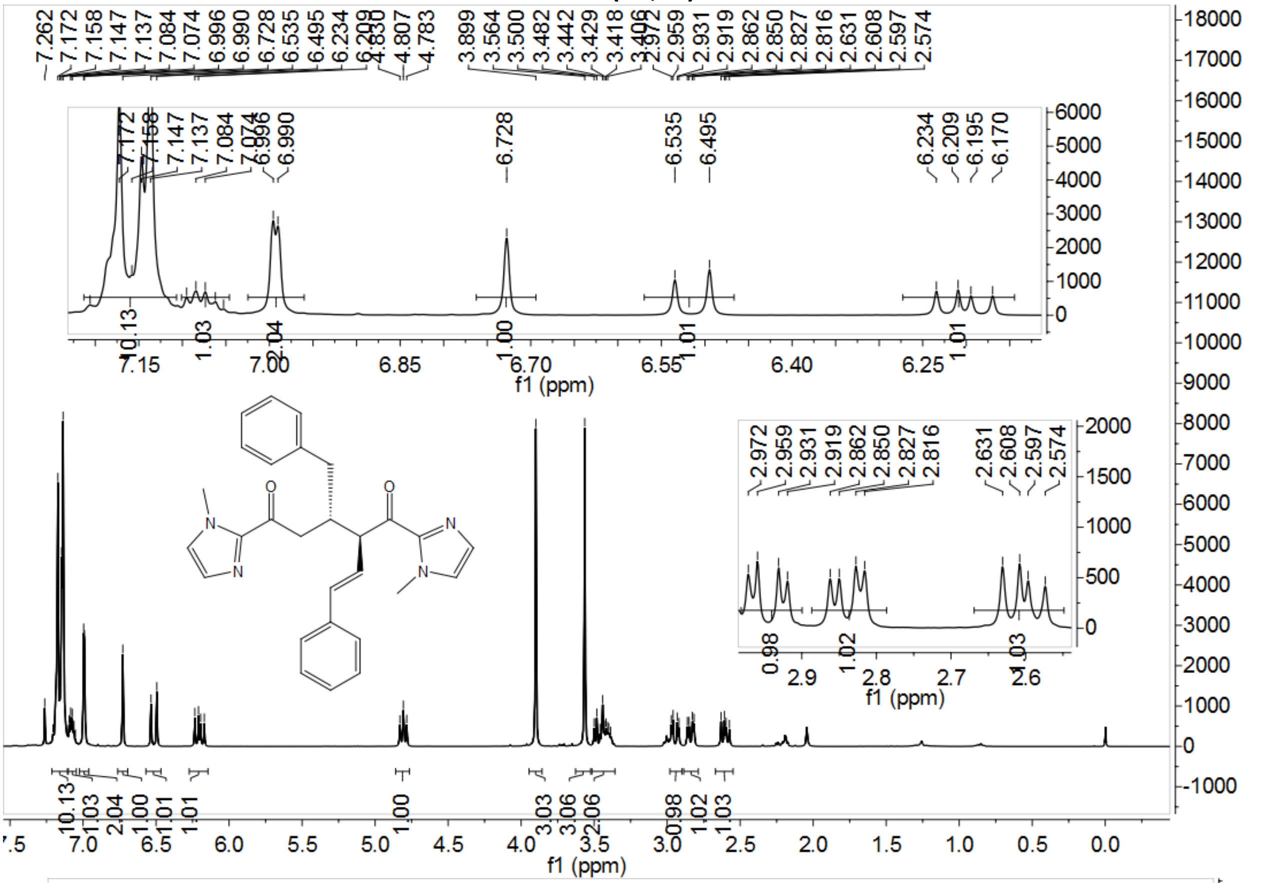

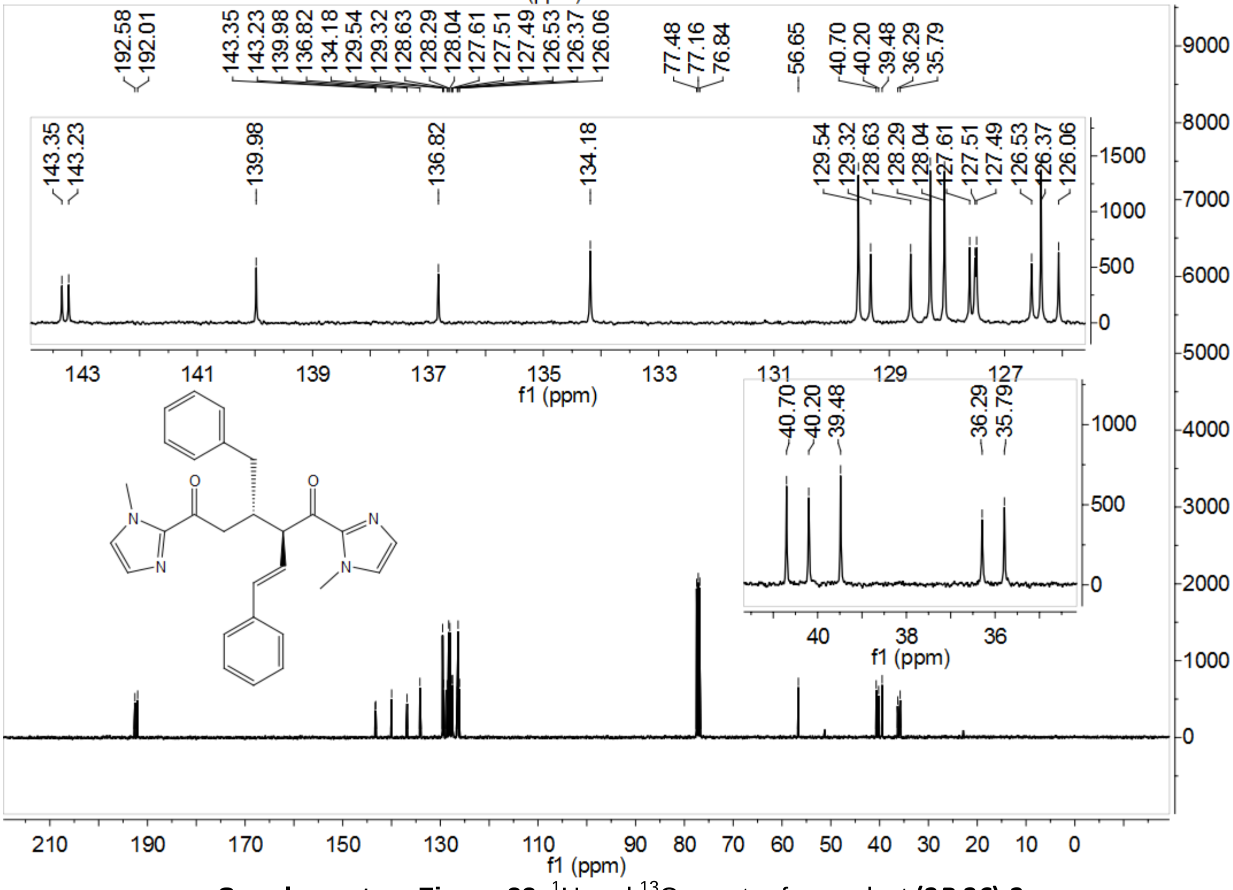

Supplementary Figure 33. ^{1}H and ^{13}C spectra for product **(2*R*,3*S*)-2a**

(2*S*,3*S*)-2a

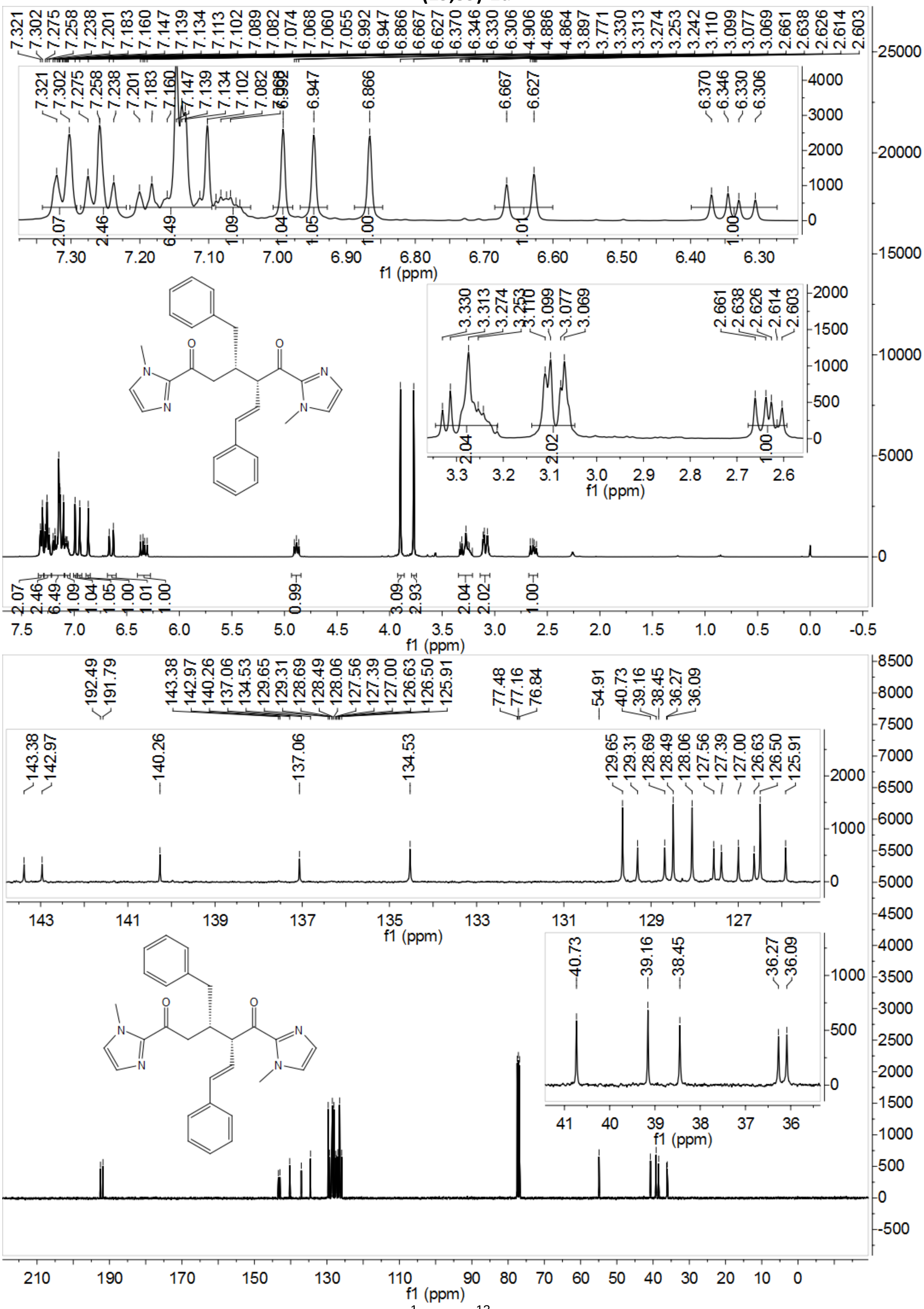

Supplementary Figure 34. ^{1}H and ^{13}C spectra for product **(2*S*,3*S*)-2a**

(2*R*,3*R*)-2a

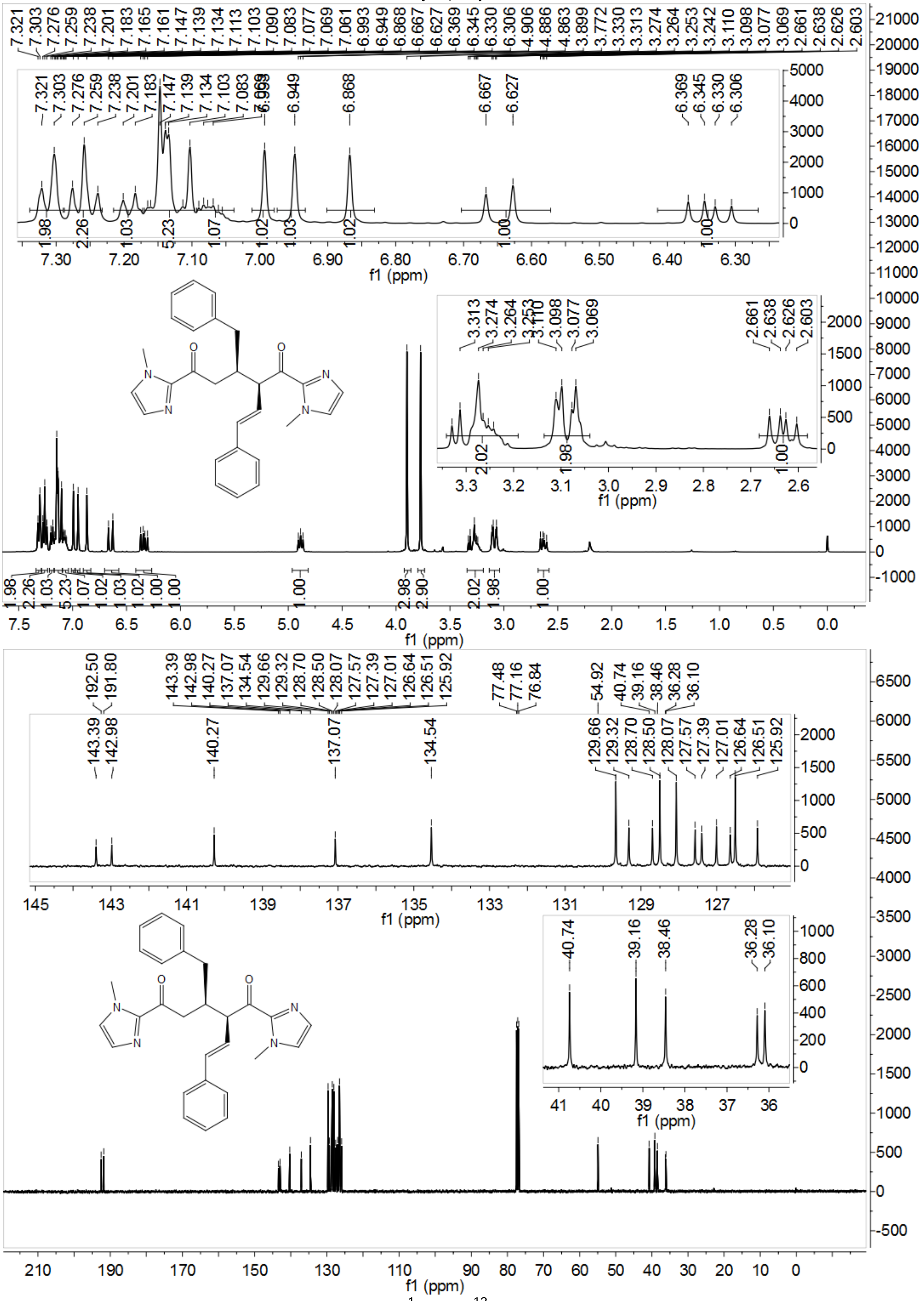

Supplementary Figure 35. ^{1}H and ^{13}C spectra for product **(2*R*,3*R*)-2a**

2b

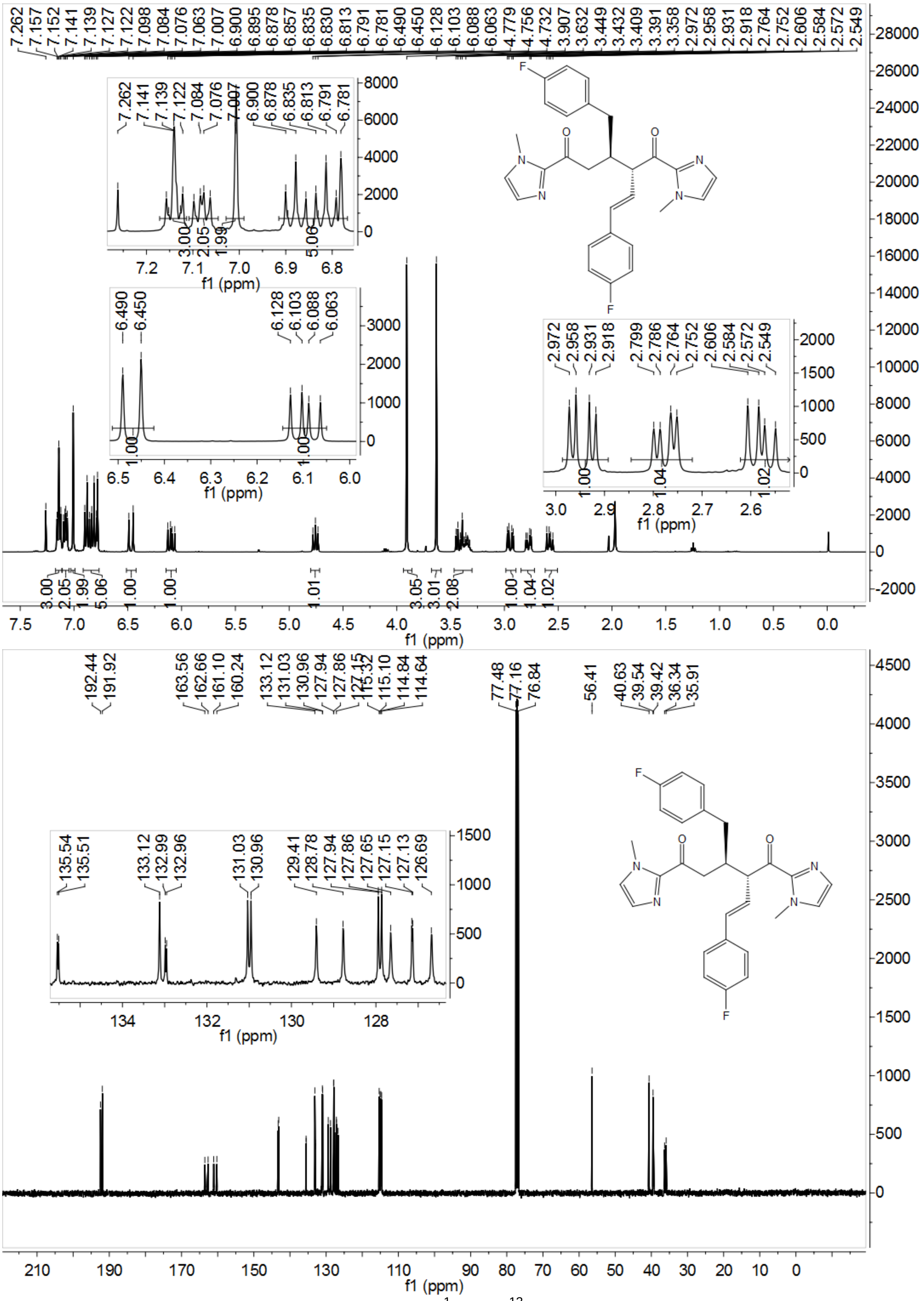

Supplementary Figure 36. ^{1}H and ^{13}C spectra for product **2b**

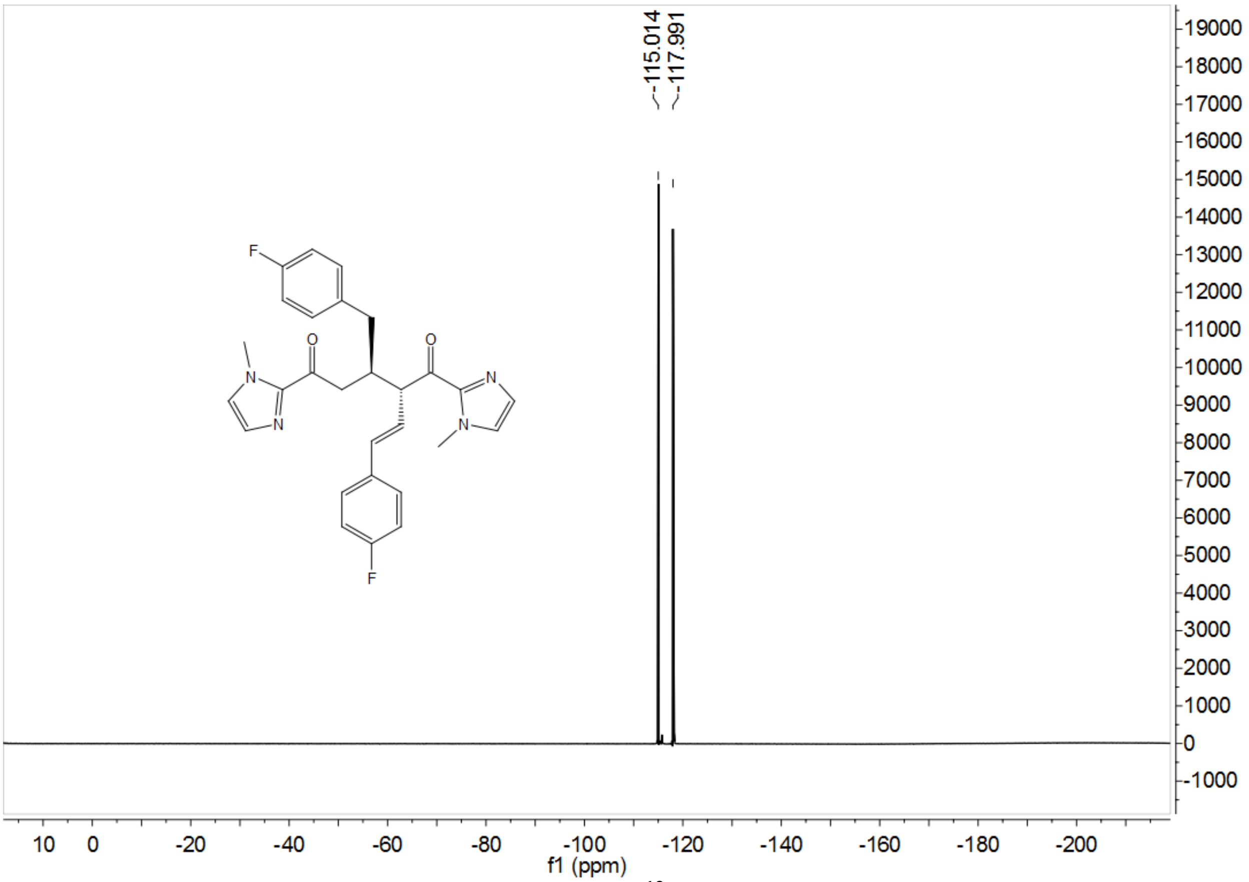

Supplementary Figure 37. ^{19}F spectra for product **2b**

2c

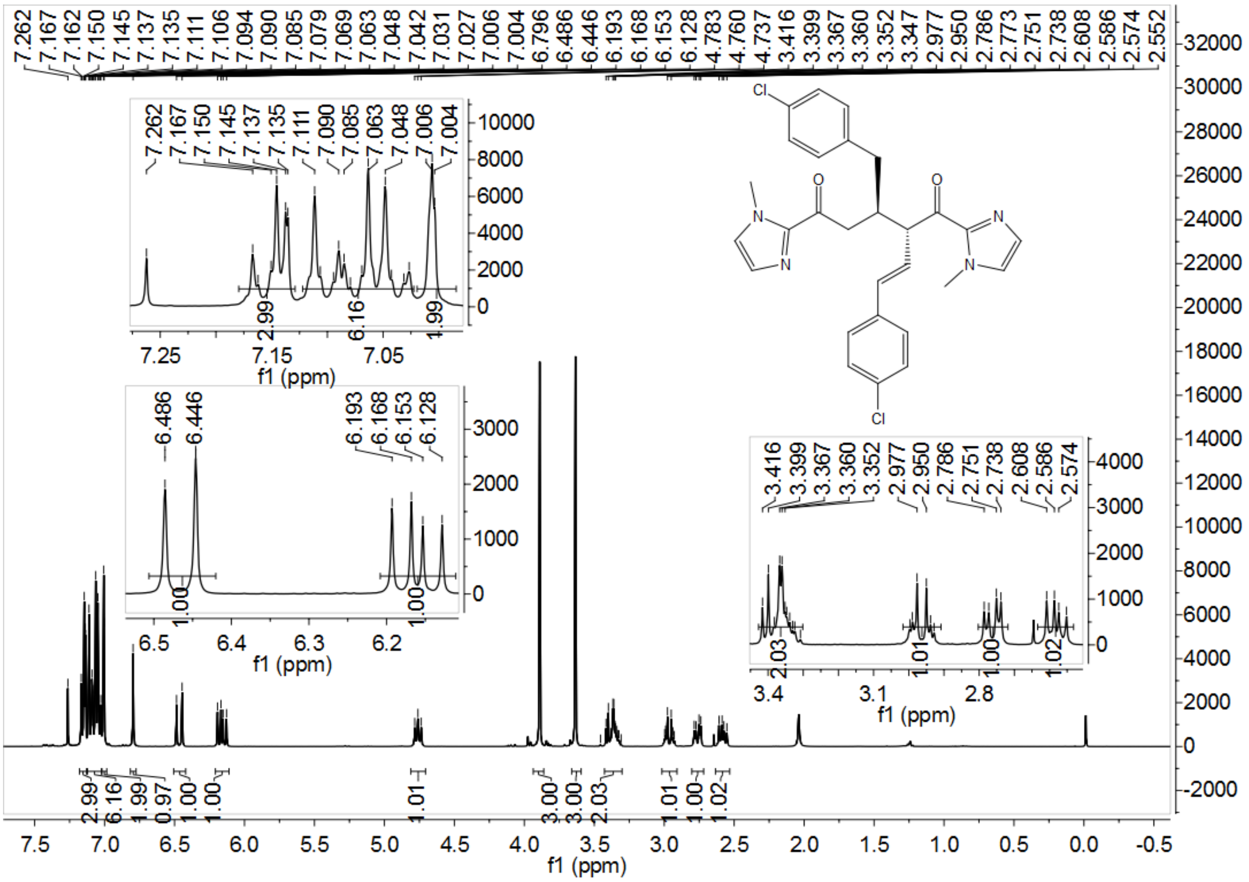

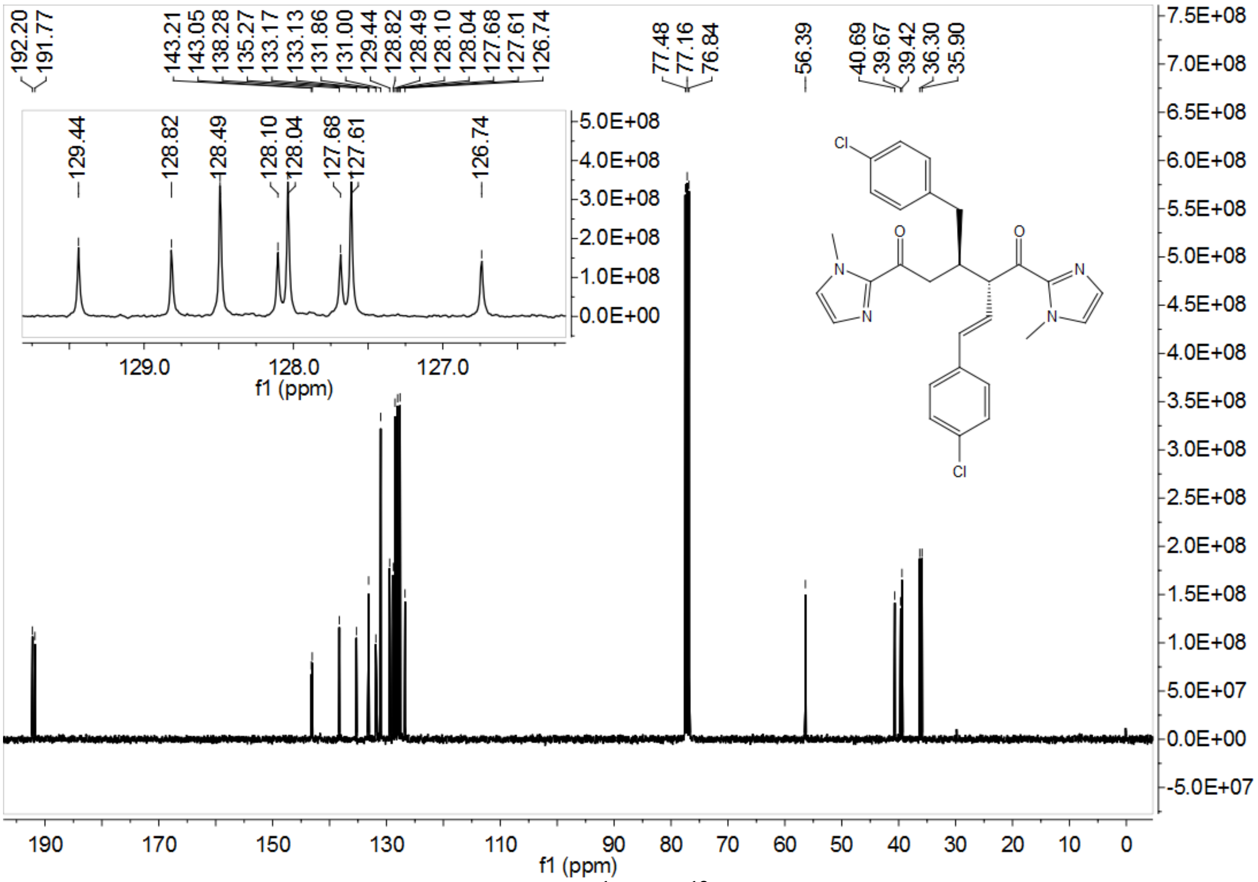

Supplementary Figure 38. ^{1}H and ^{13}C spectra for product **2c**

2d

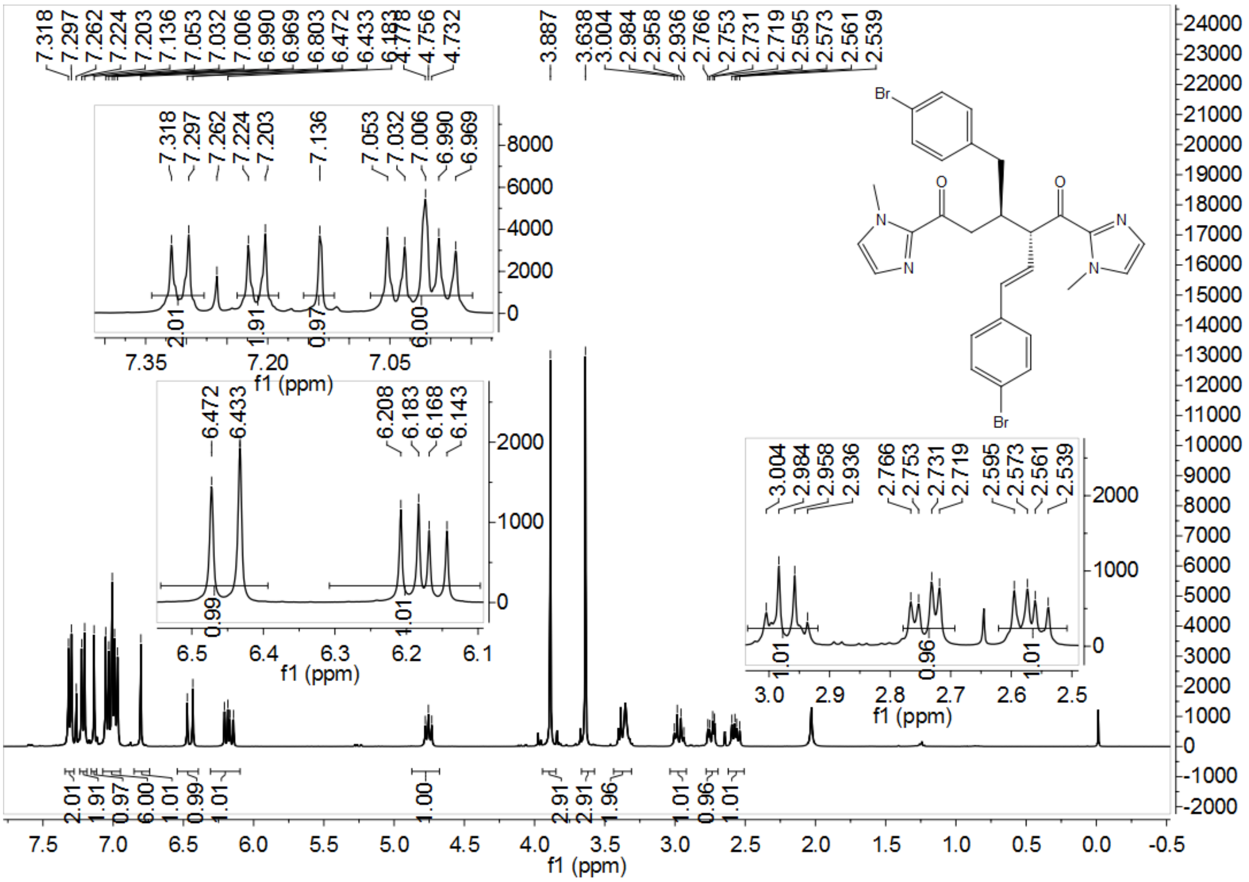

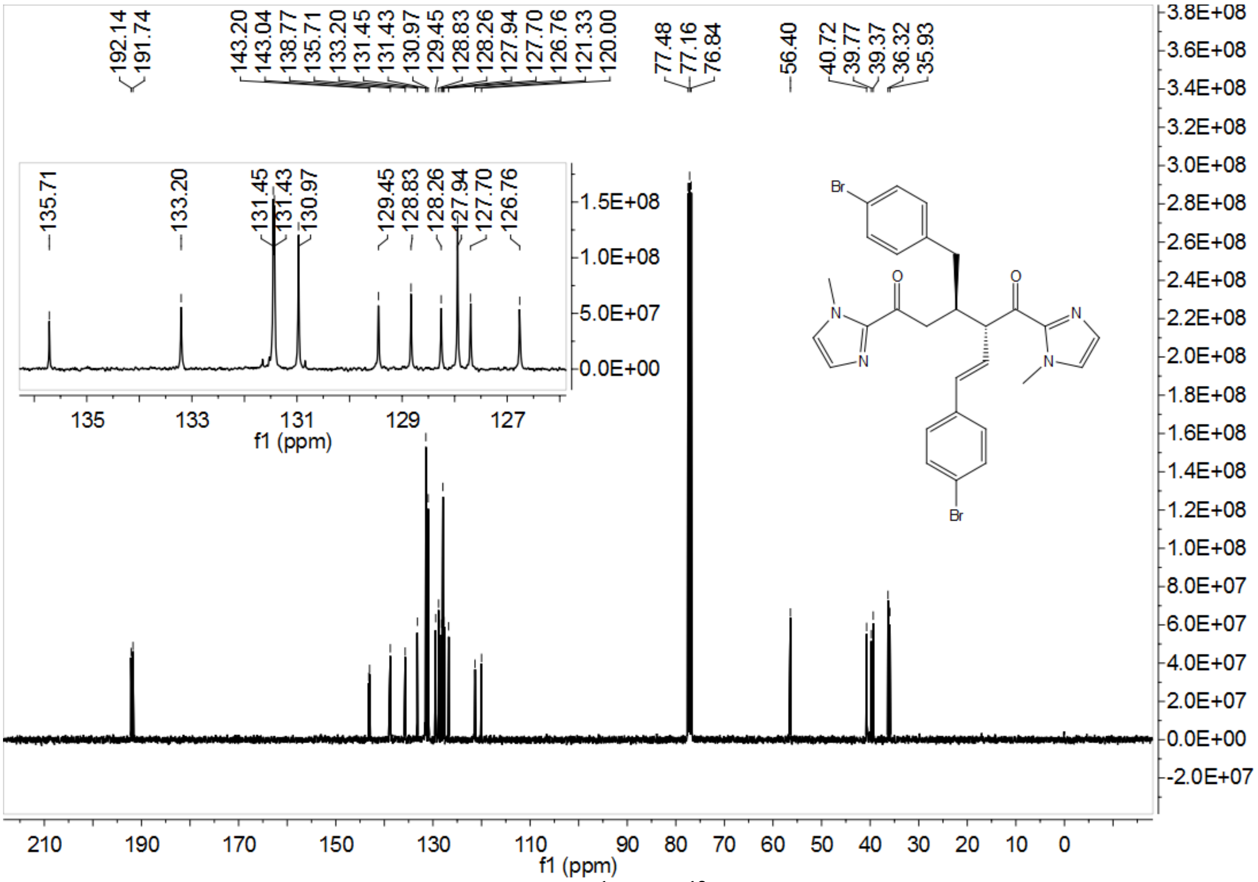

Supplementary Figure 39. ^{1}H and ^{13}C spectra for product **2d**

2e

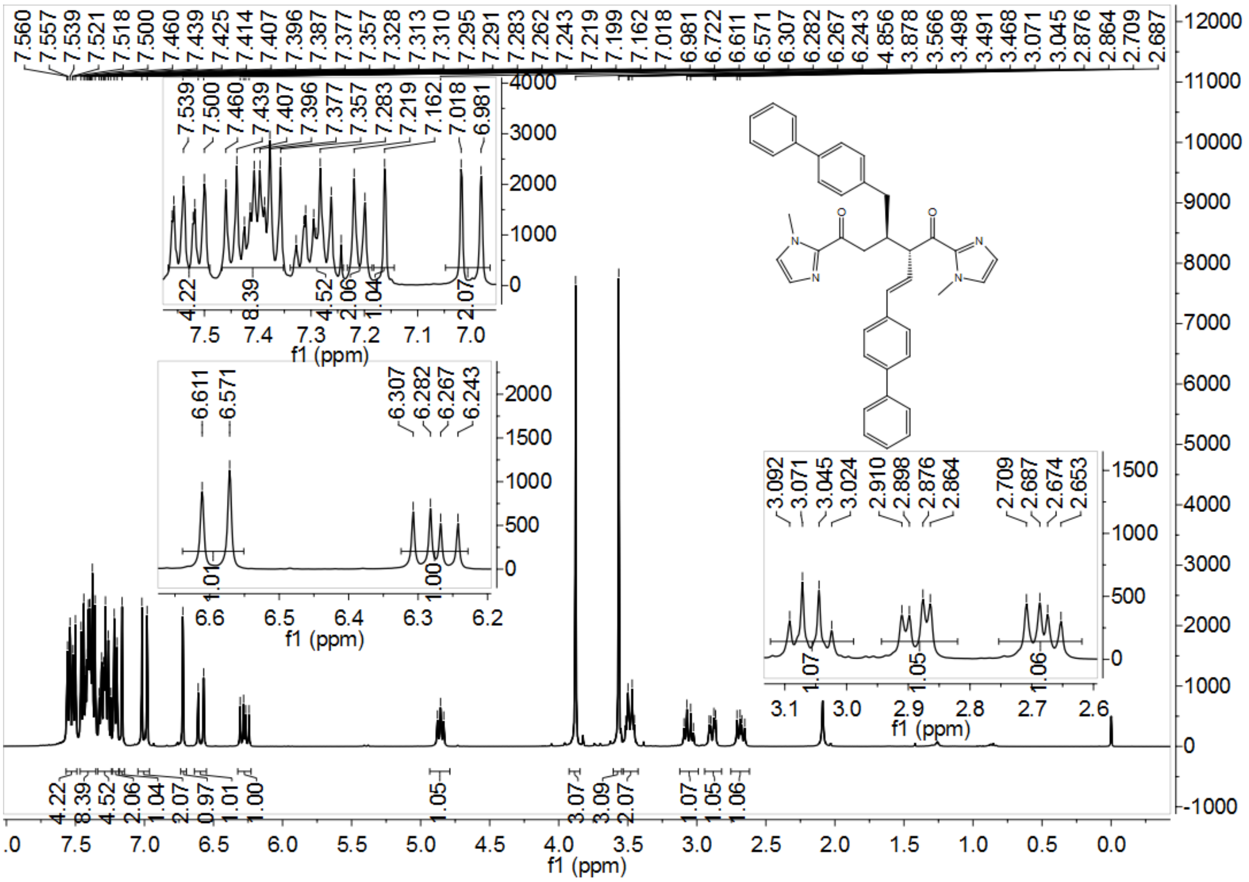

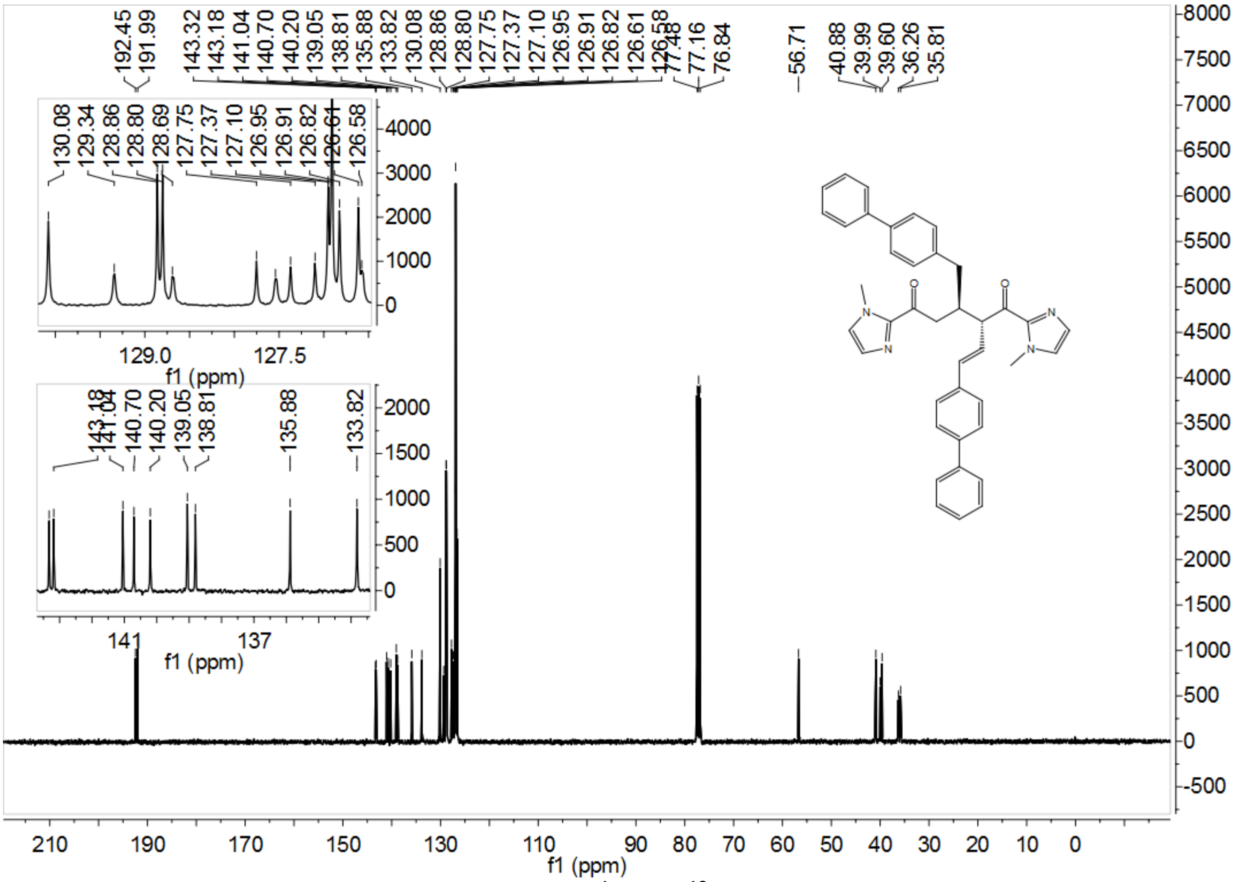

Supplementary Figure 40. ^{1}H and ^{13}C spectra for product **2e**

2f

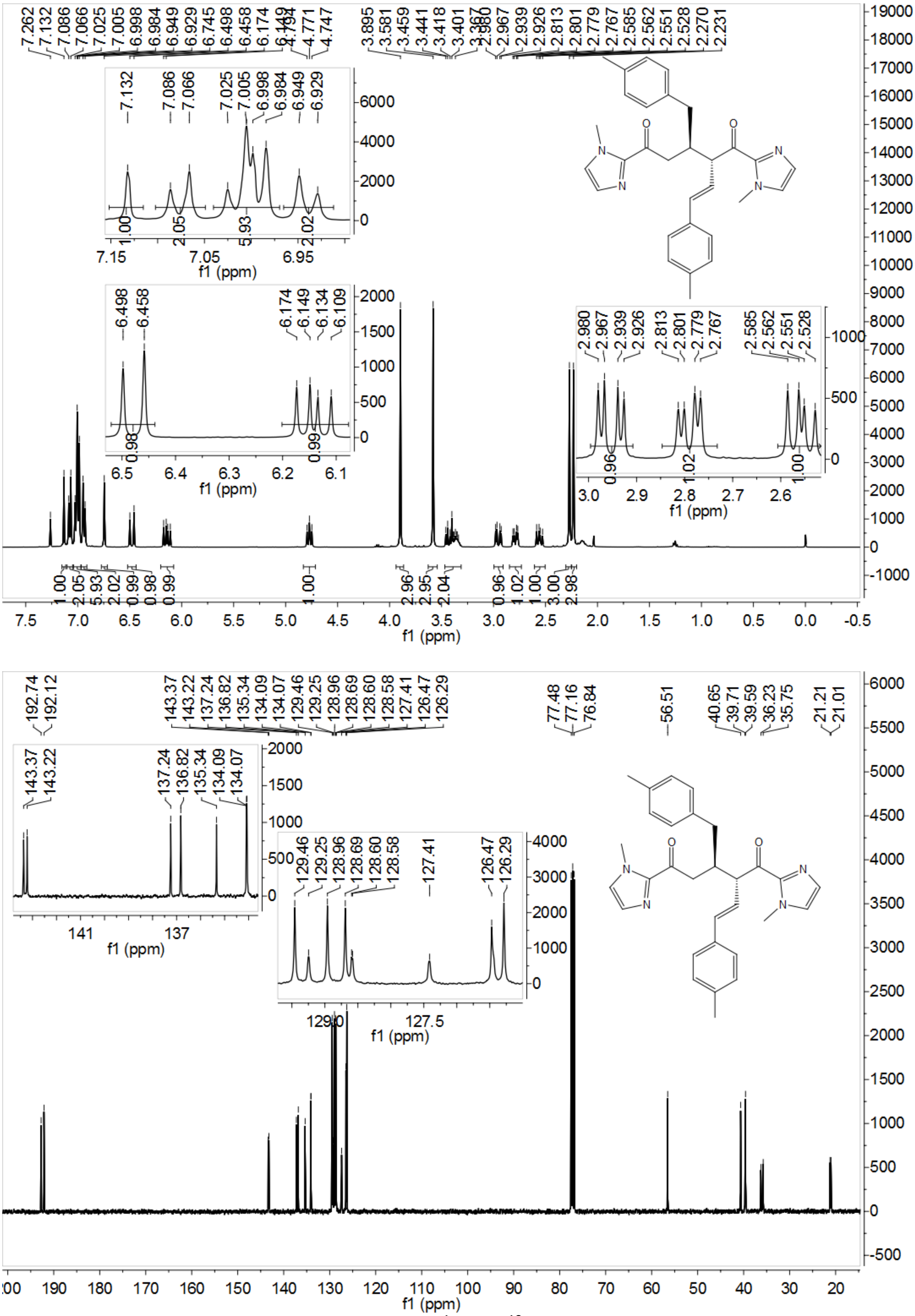

Supplementary Figure 41. ^{1}H and ^{13}C spectra for product **2f**

2g

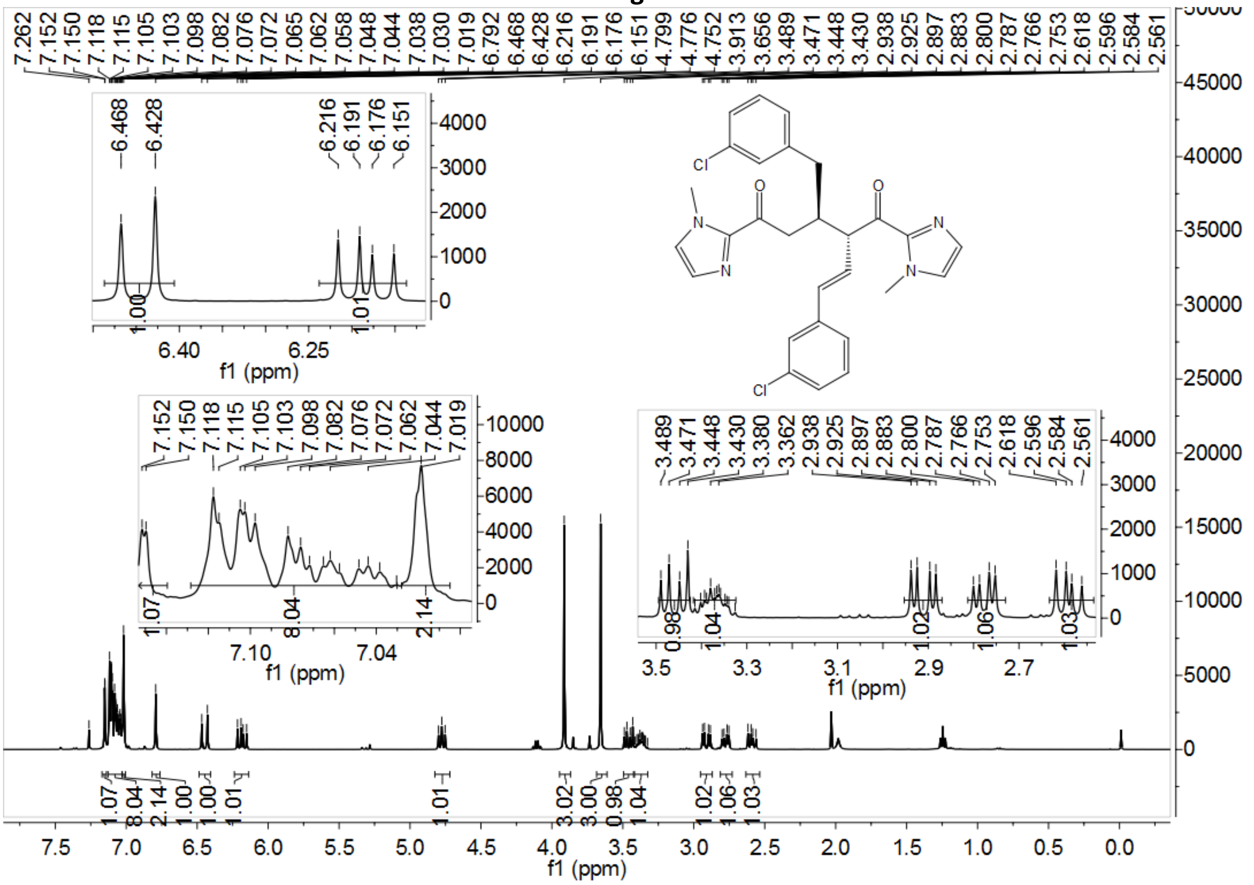

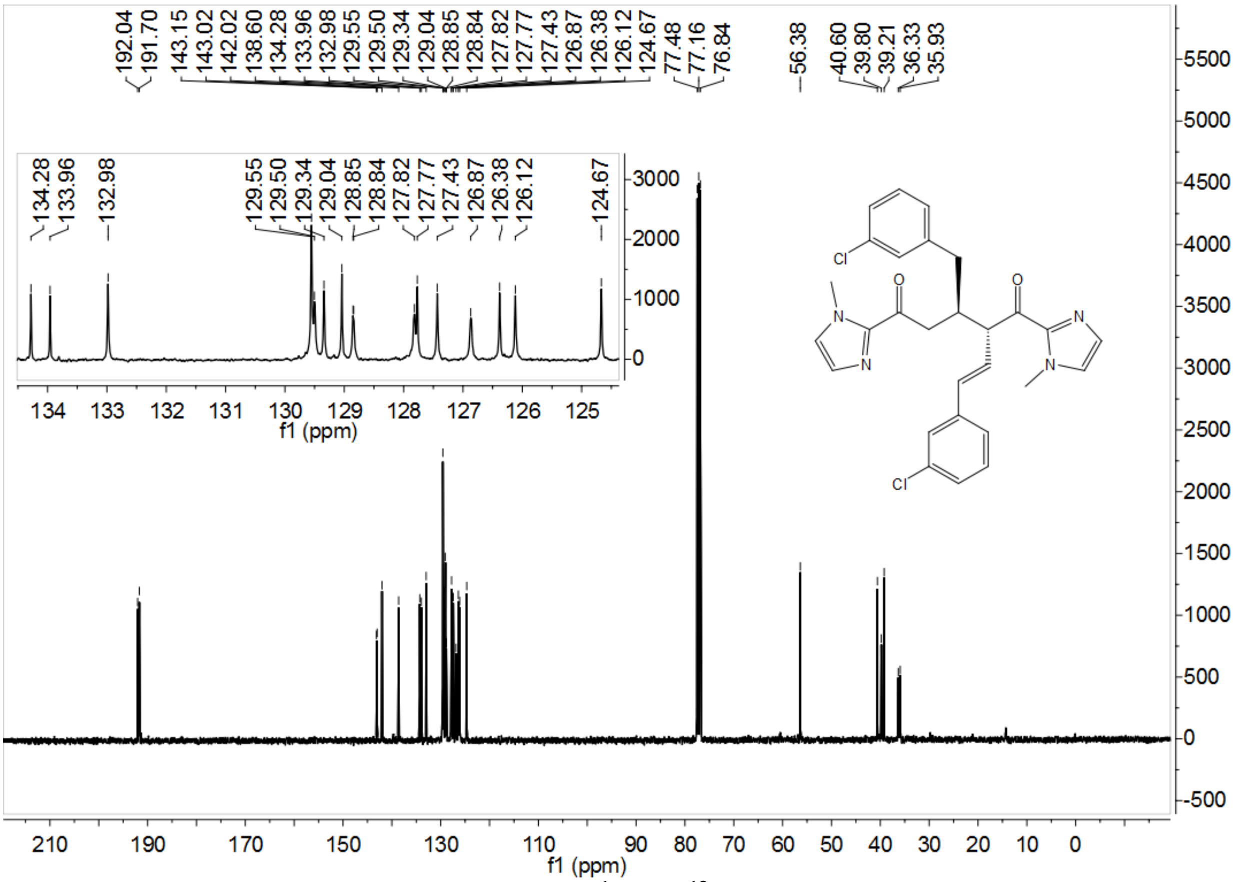

Supplementary Figure 42. ^{1}H and ^{13}C spectra for product **2g**

2h

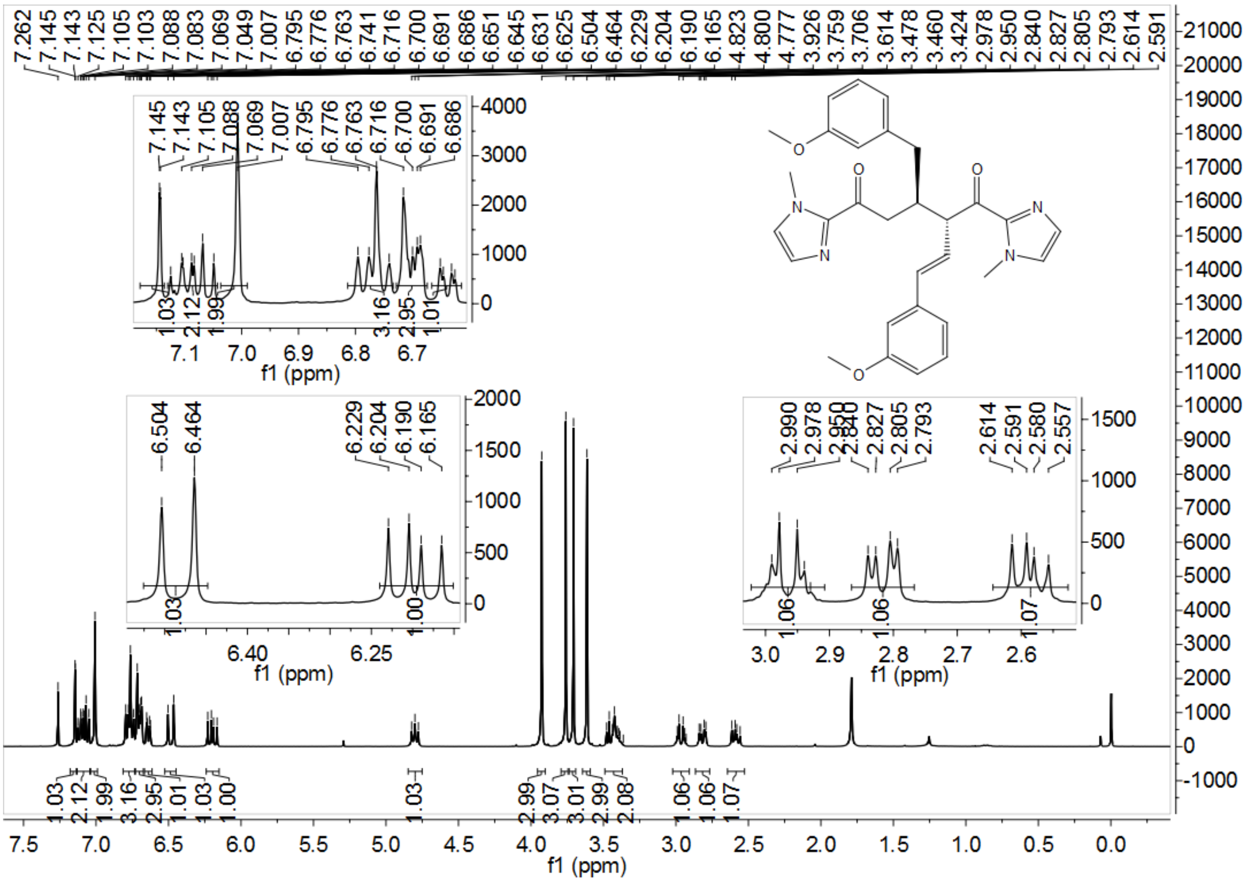

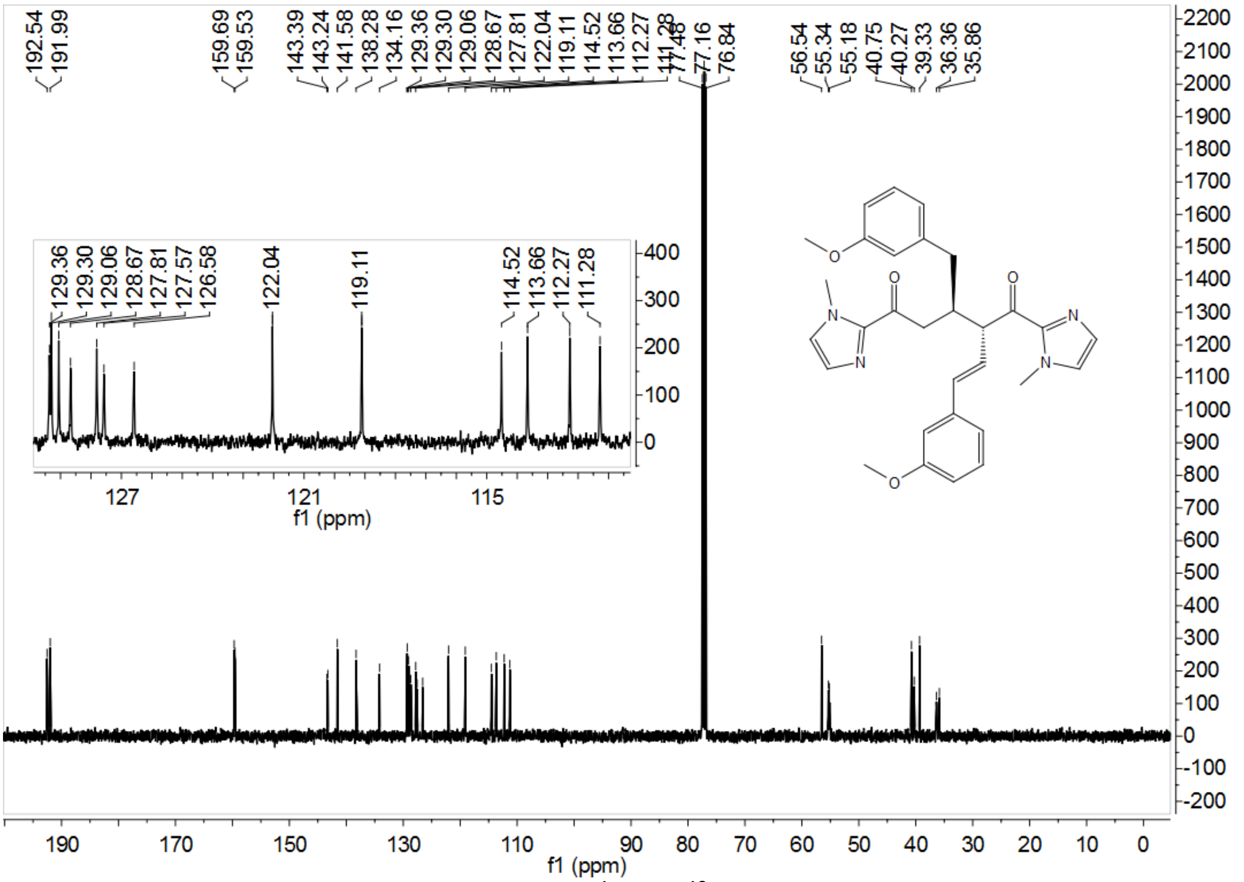

Supplementary Figure 43. ^{1}H and ^{13}C spectra for product **2h**

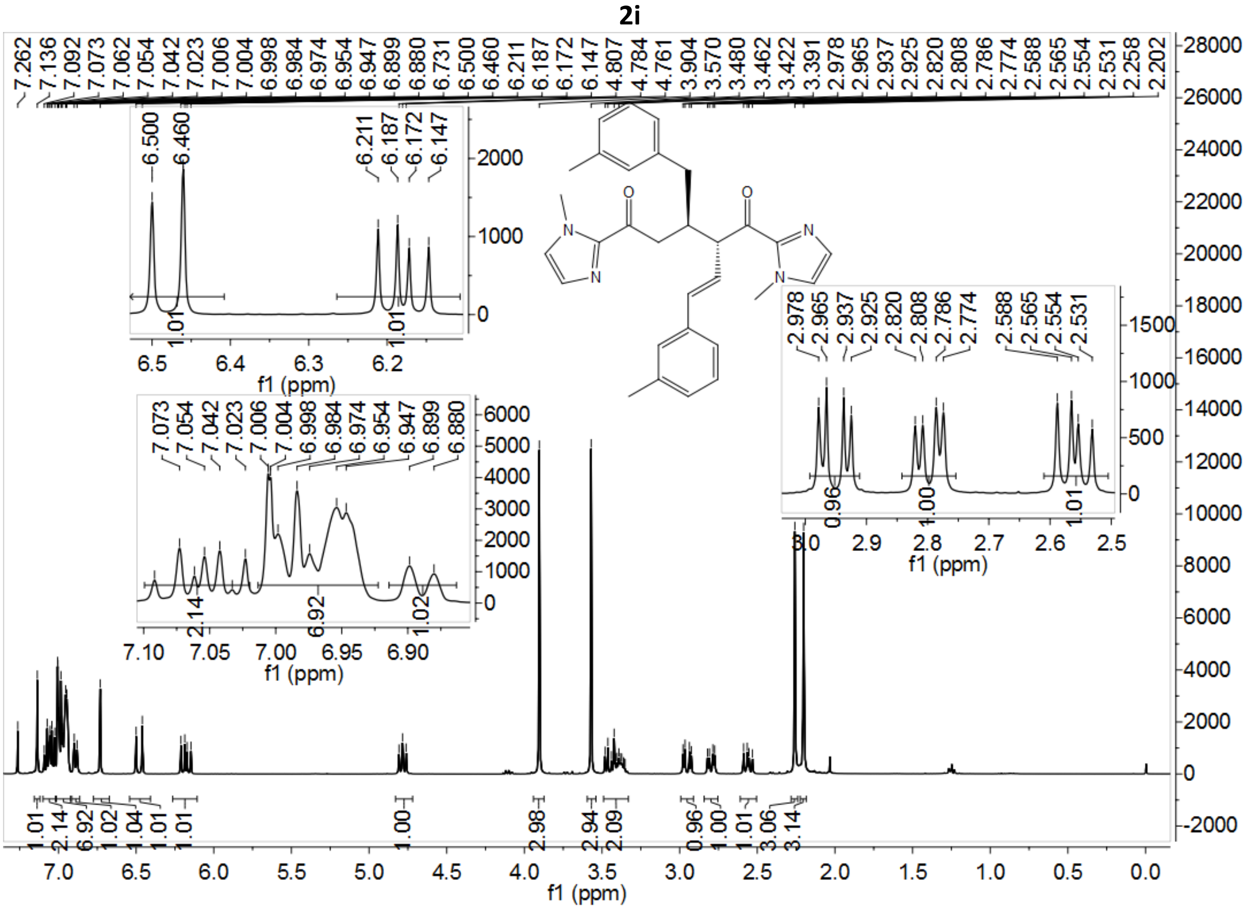

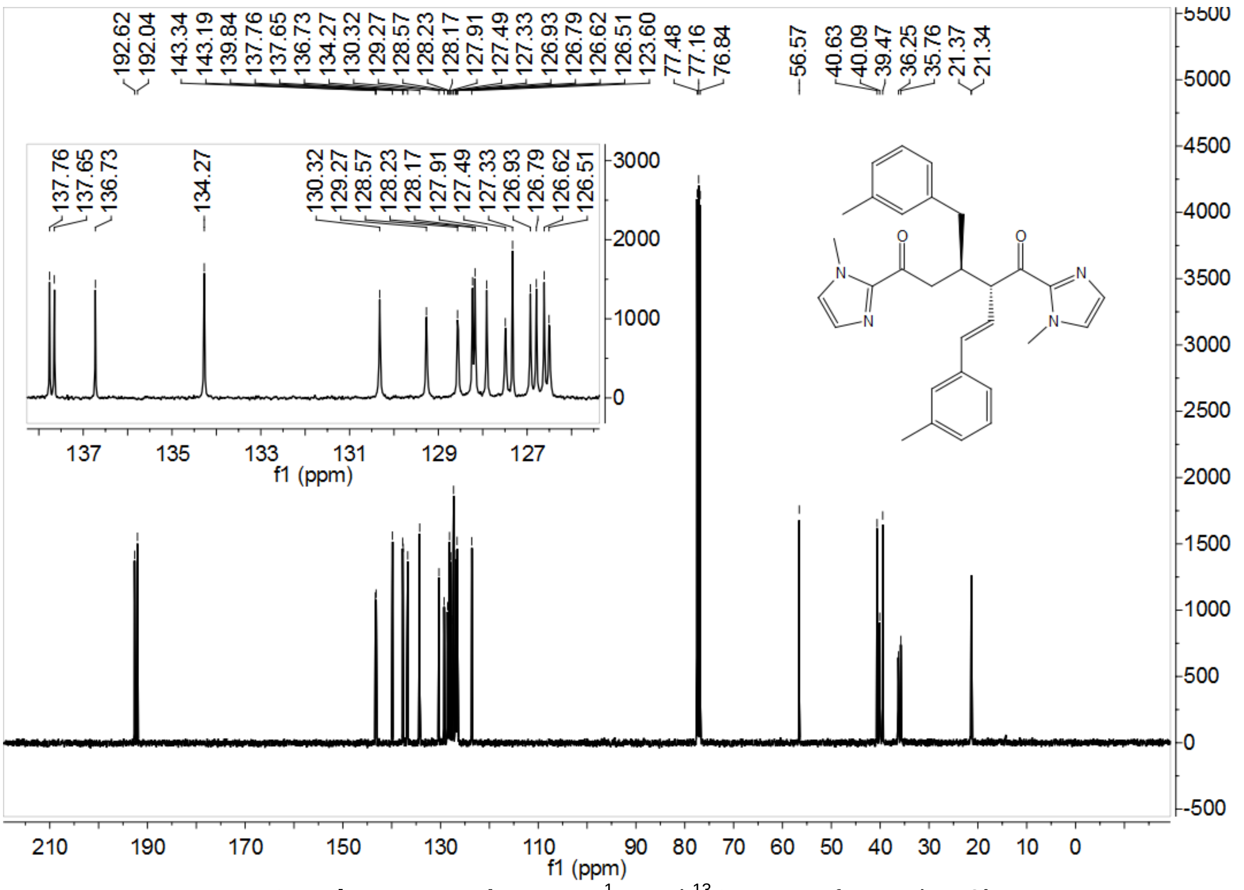

Supplementary Figure 44. ^{1}H and ^{13}C spectra for product **2i**

2j

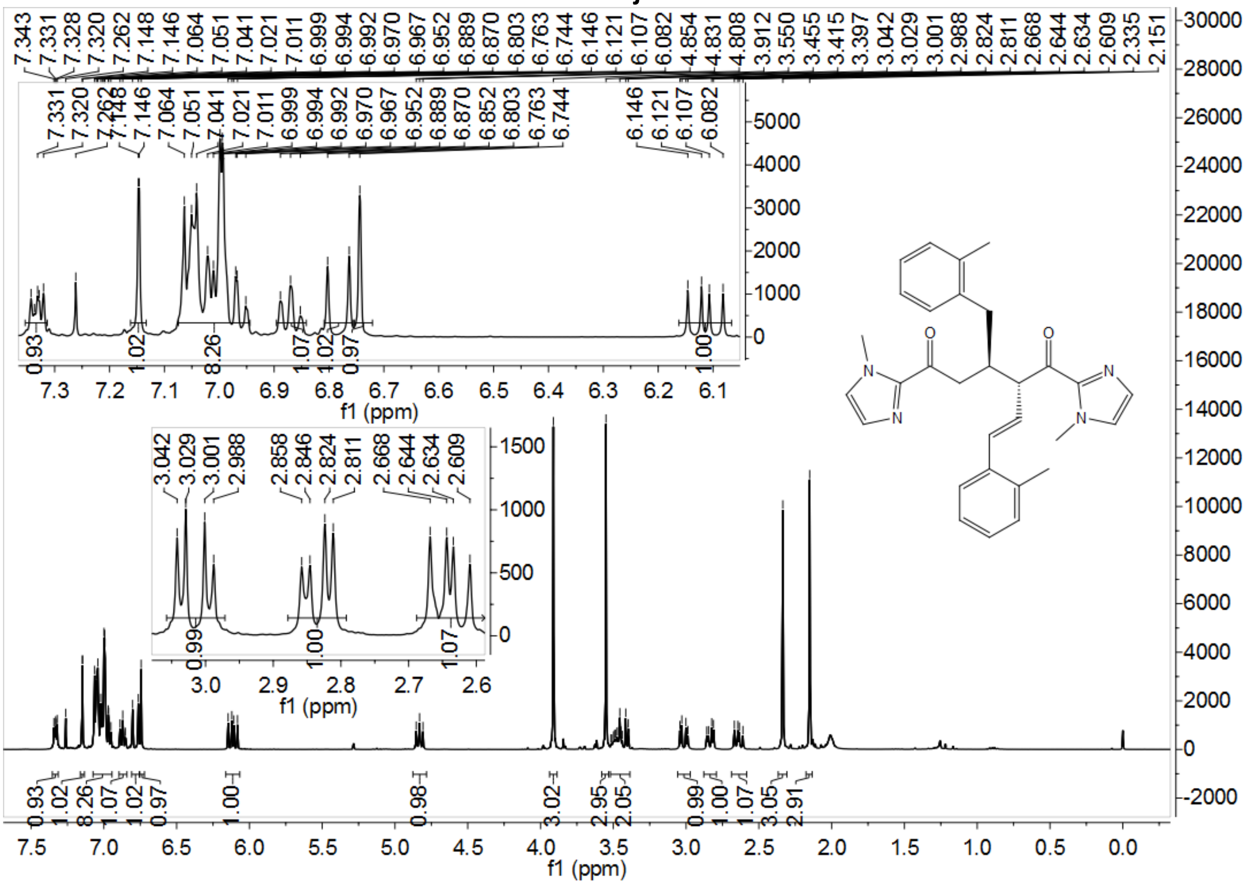

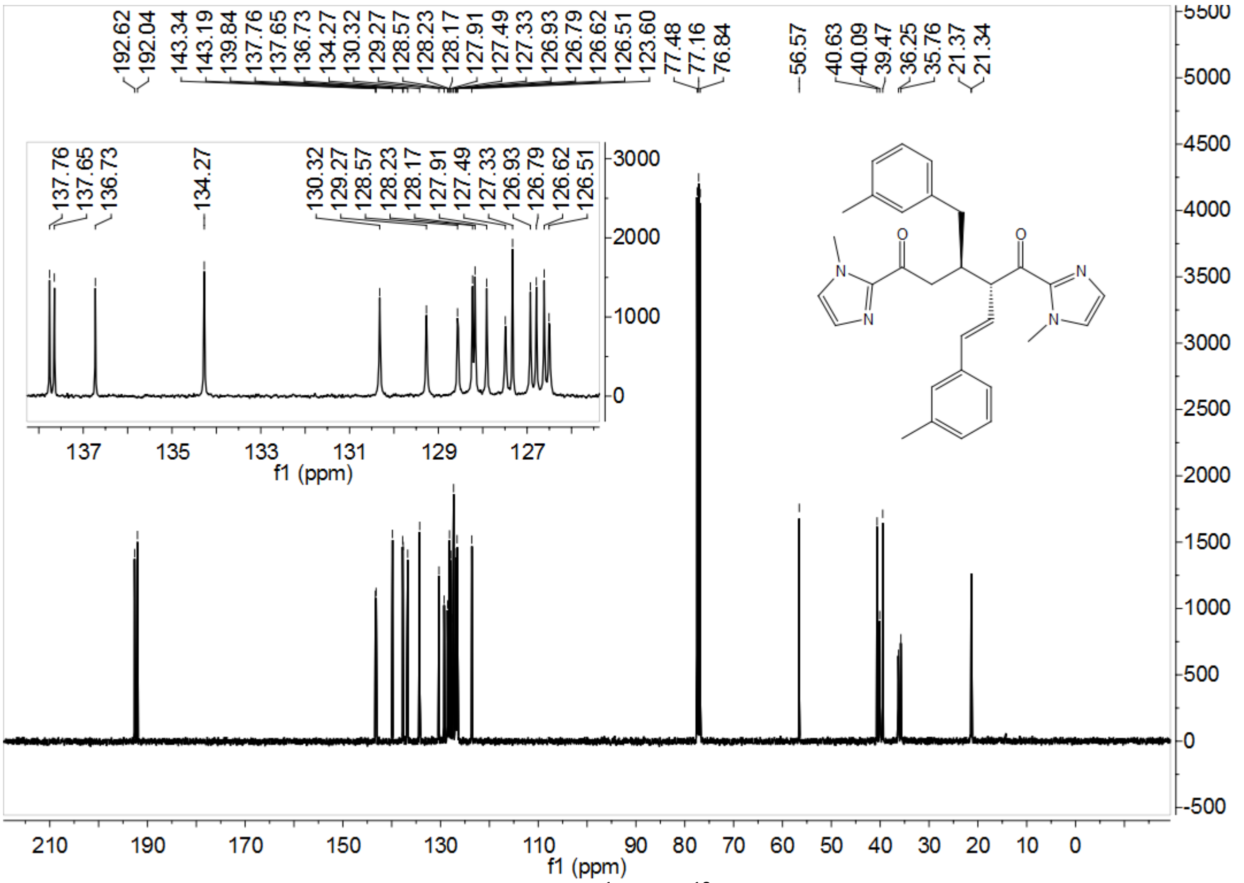

Supplementary Figure 45. ^{1}H and ^{13}C spectra for product **2j**

2k

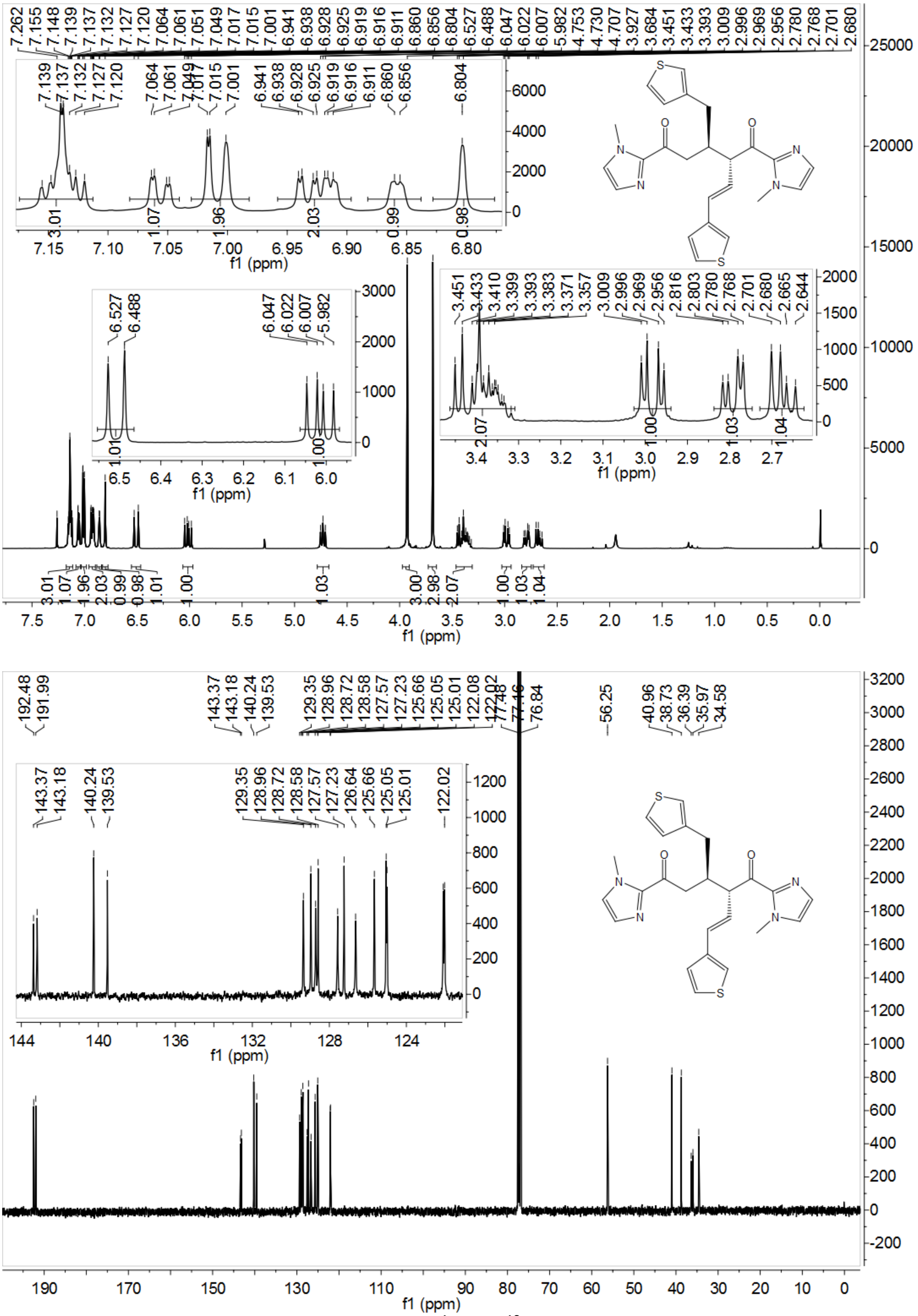

Supplementary Figure 46. ^{1}H and ^{13}C spectra for product **2k**

2l

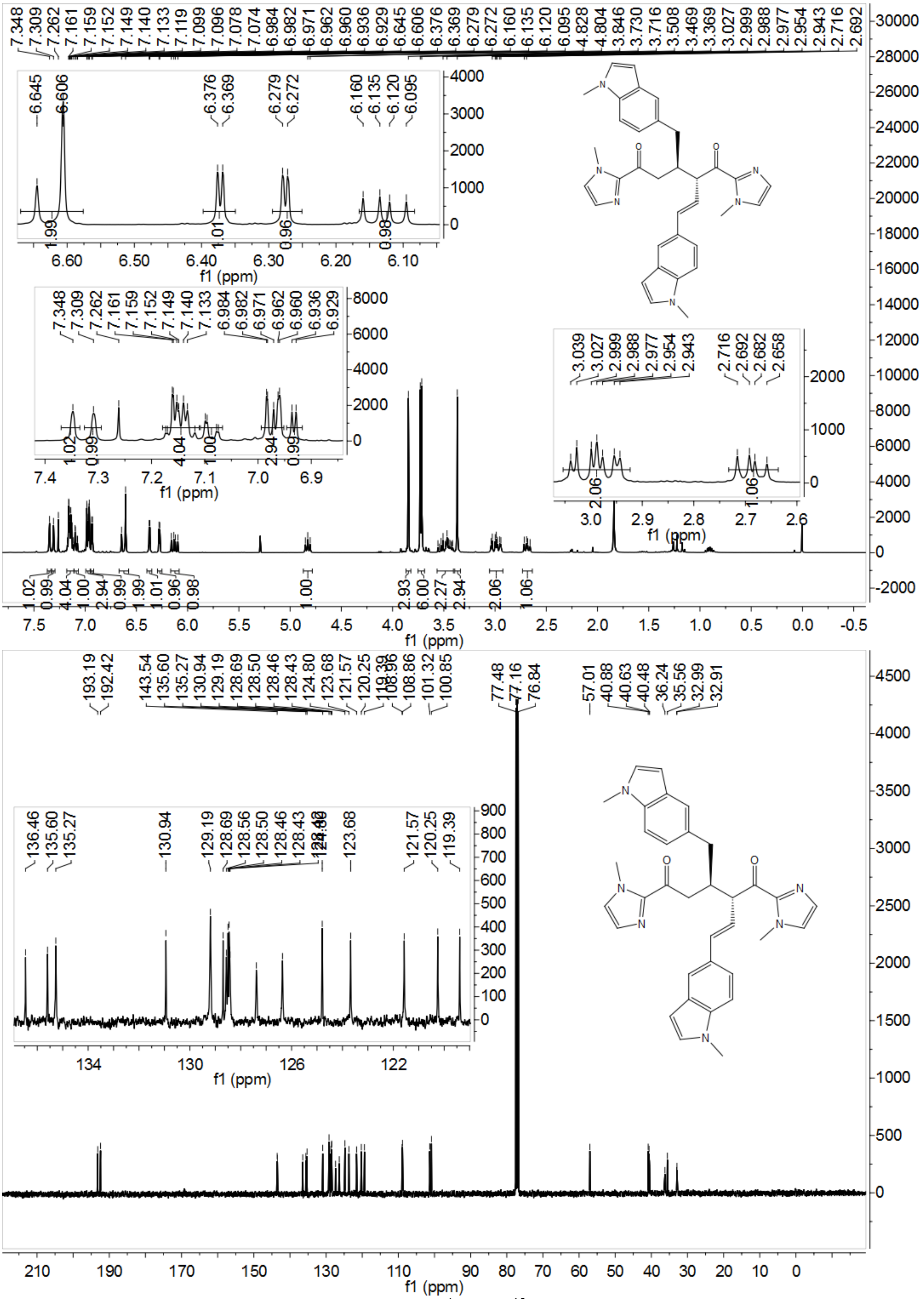

Supplementary Figure 47. ^{1}H and ^{13}C spectra for product **2l**

2m

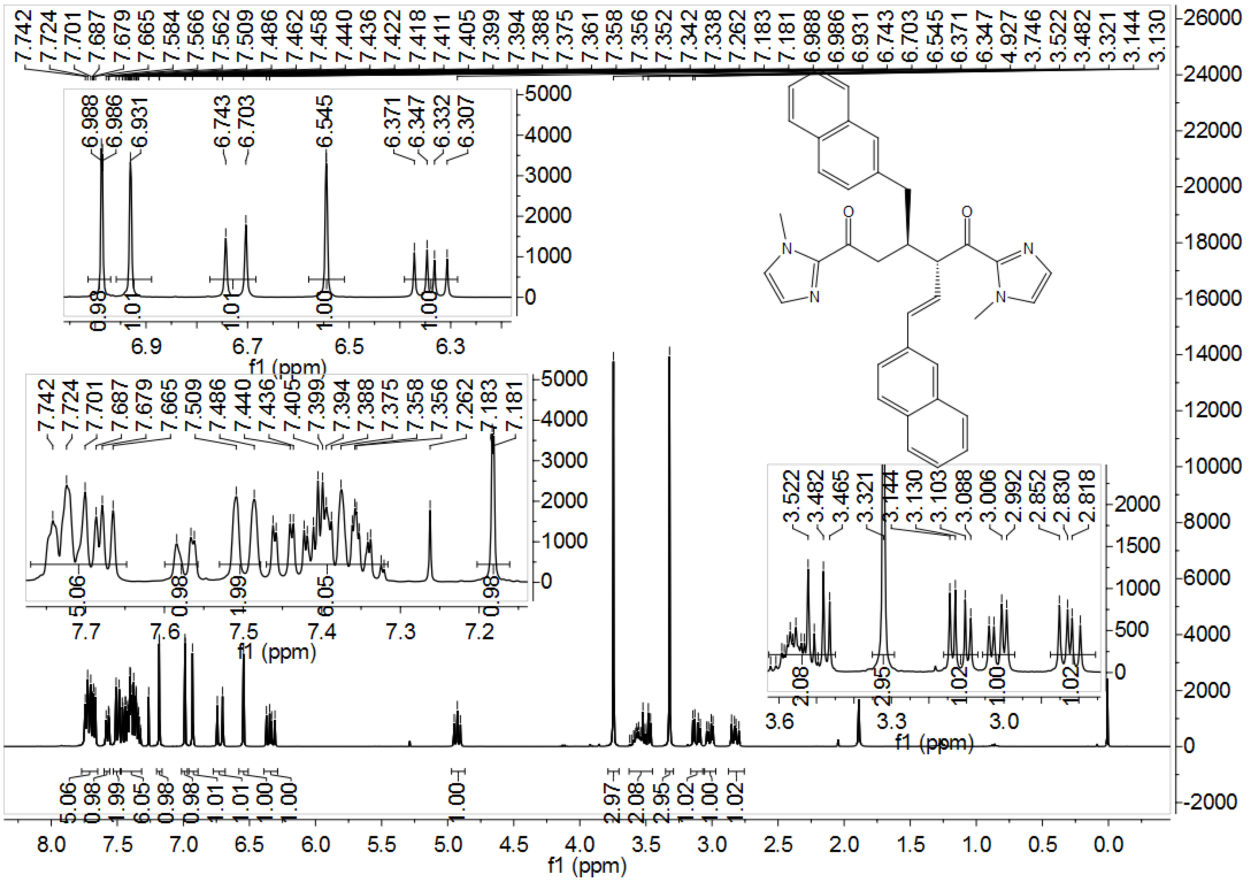

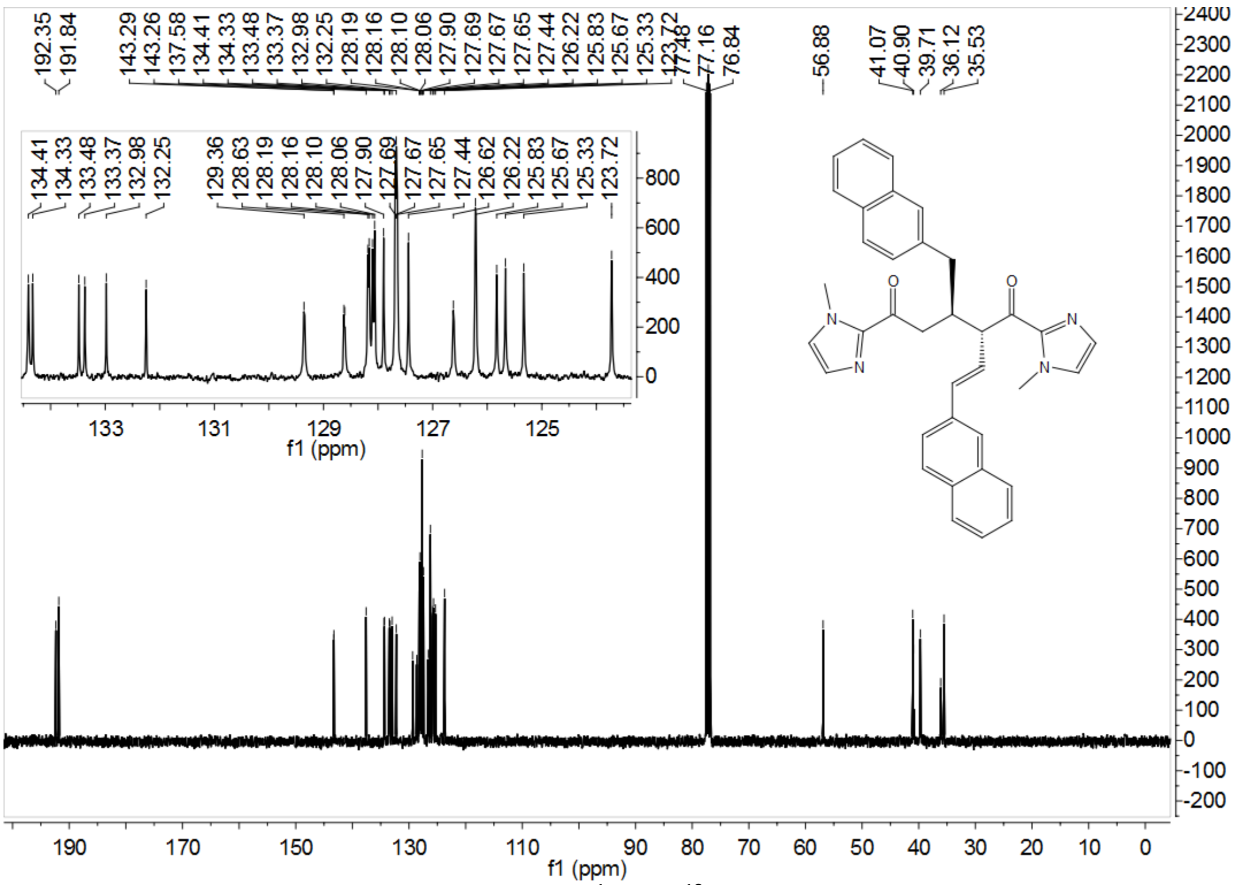

Supplementary Figure 48. ^{1}H and ^{13}C spectra for product **2m**

2n

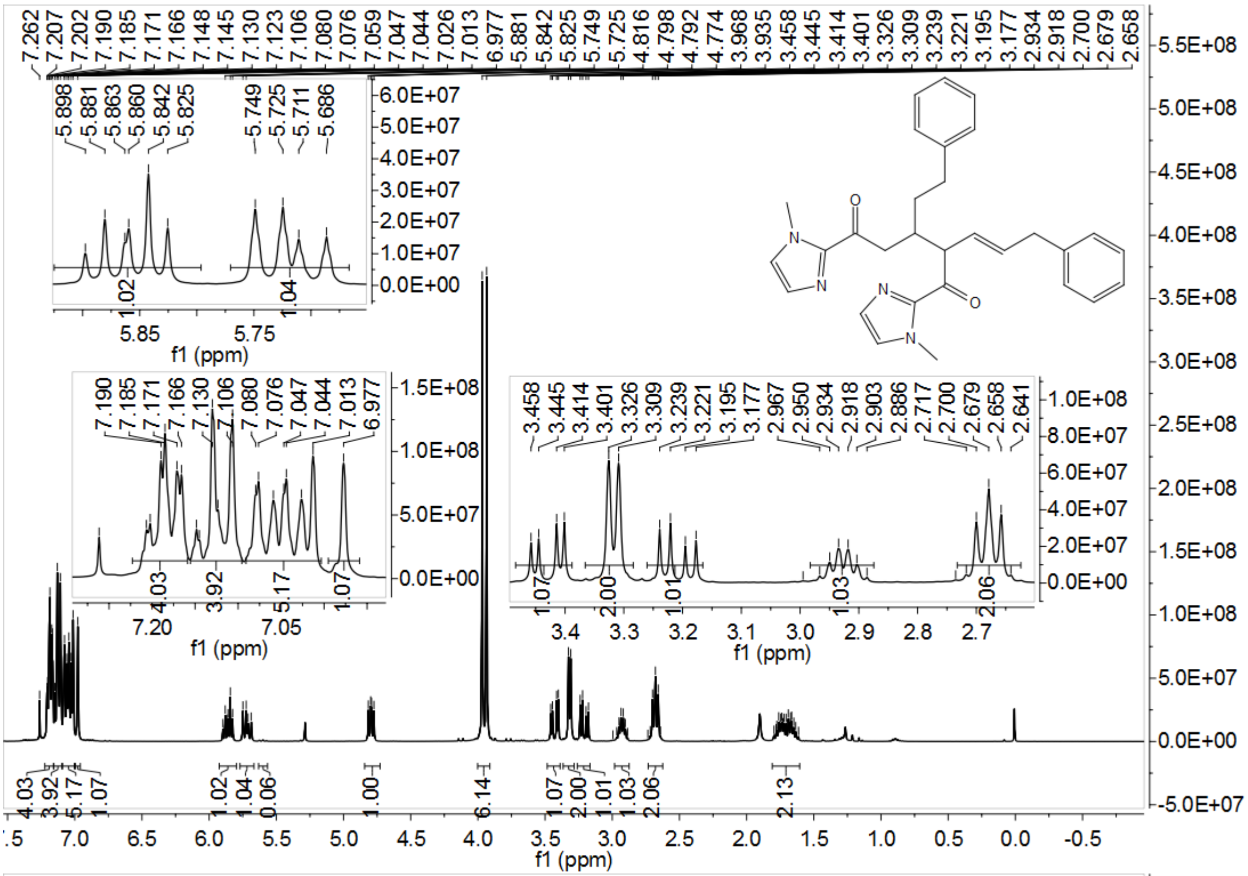

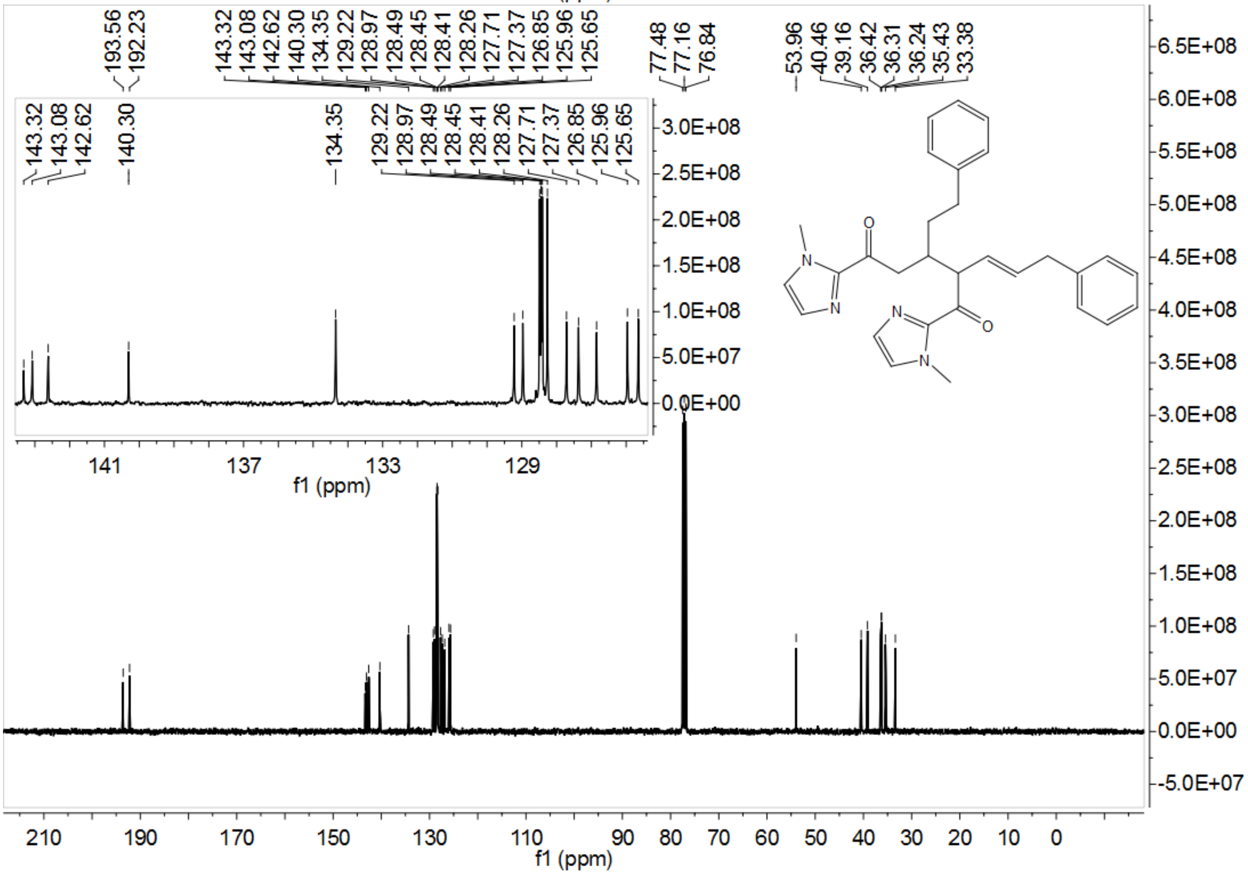

Supplementary Figure 49. ^{1}H and ^{13}C spectra for product **2n**

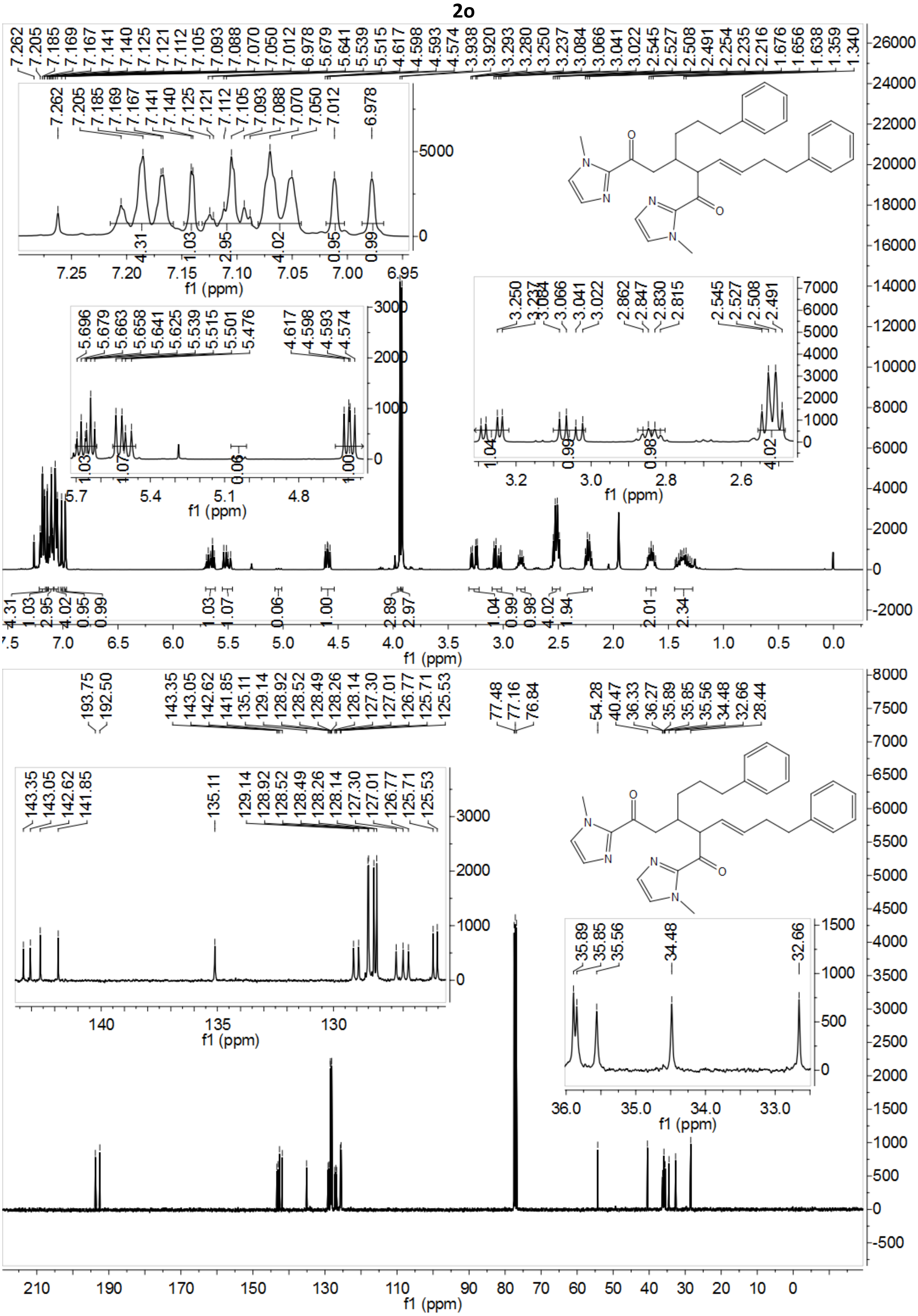

Supplementary Figure 50. ^{1}H and ^{13}C spectra for product **2o**

2p

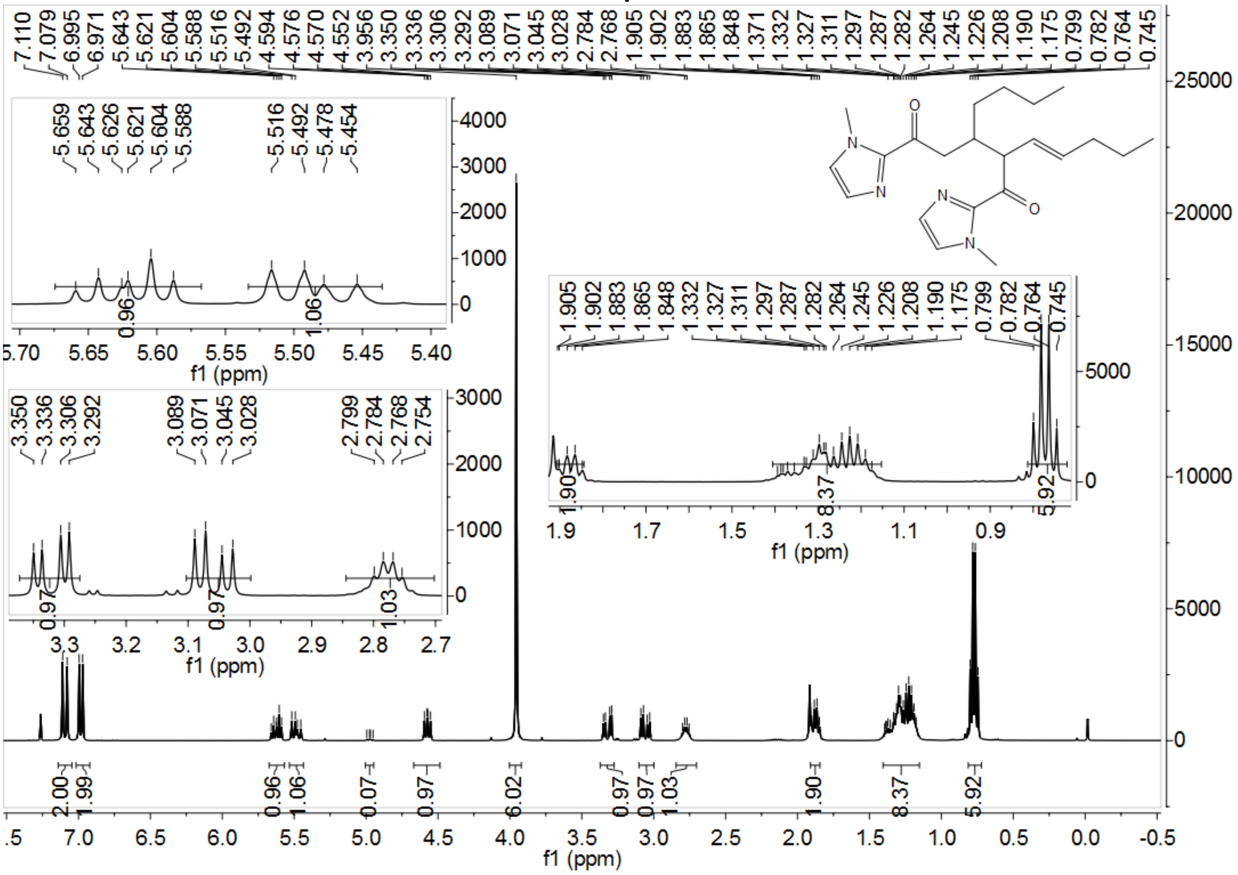

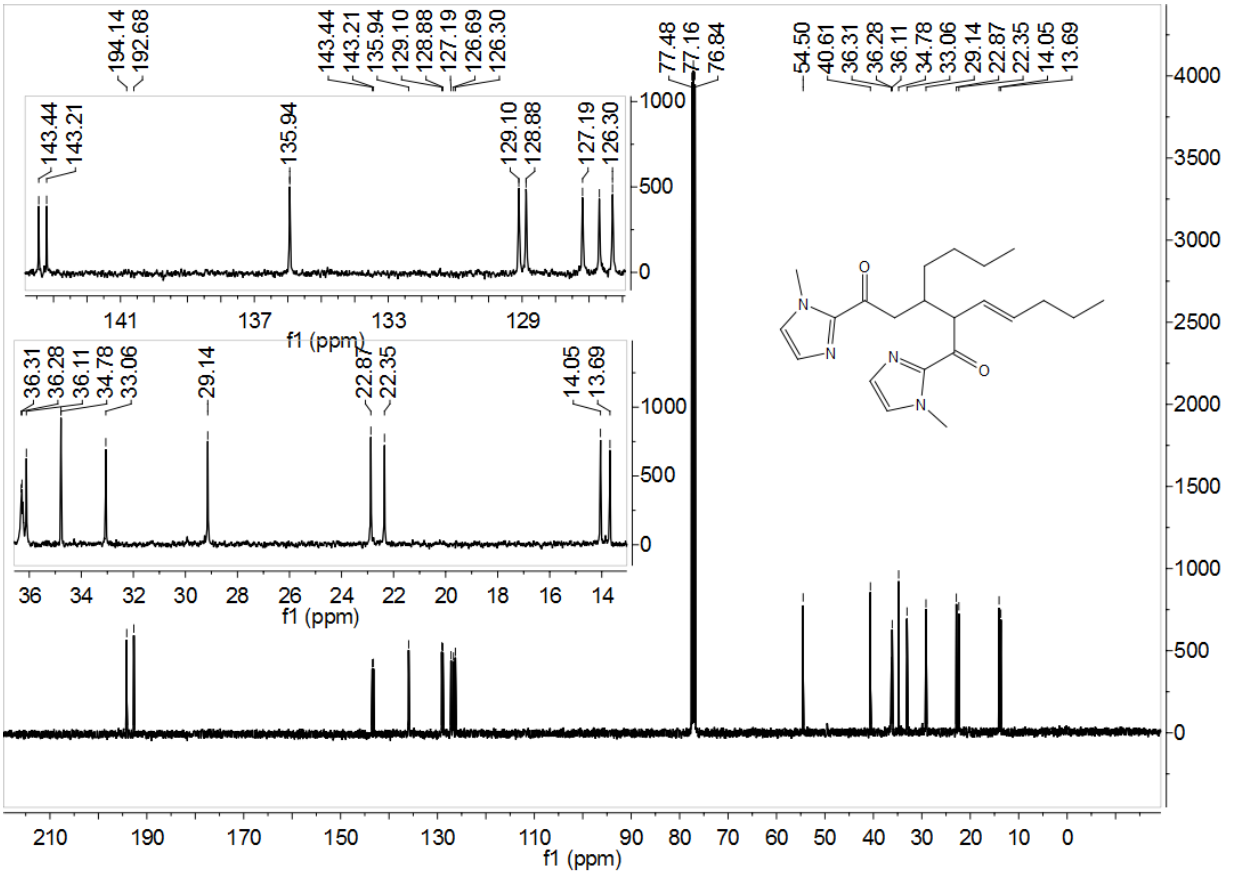

Supplementary Figure 51. ^{1}H and ^{13}C spectra for product **2p**

2q

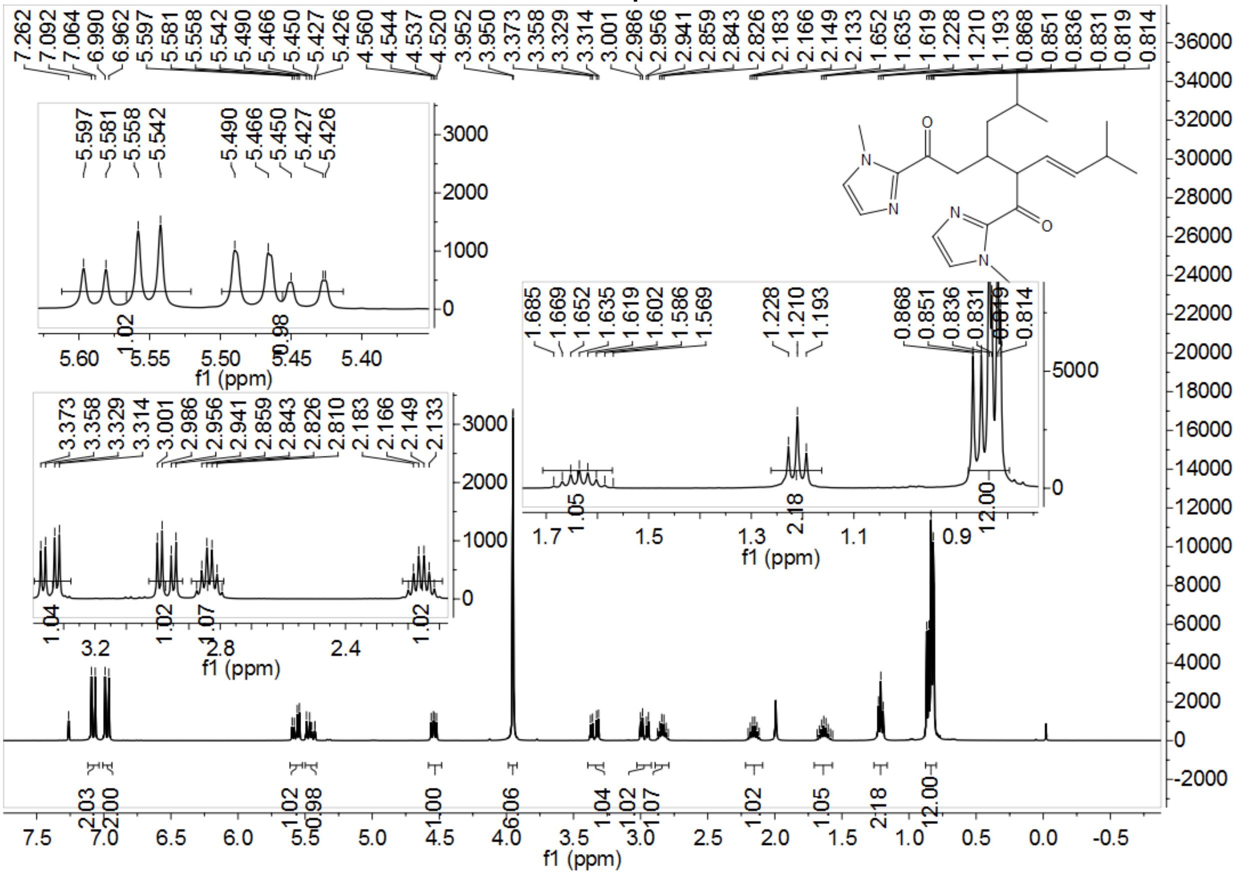

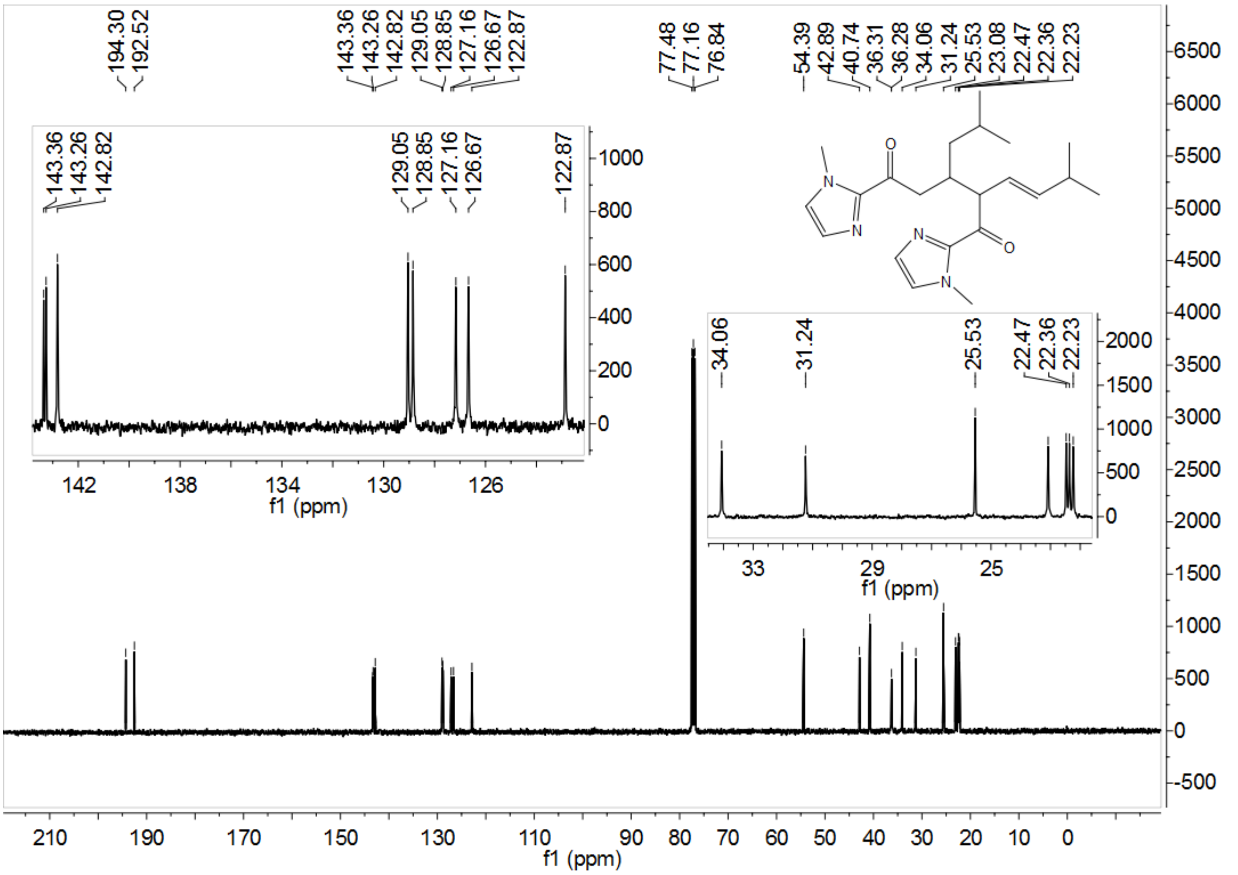

Supplementary Figure 52. ^{1}H and ^{13}C spectra for product **2q**

2r

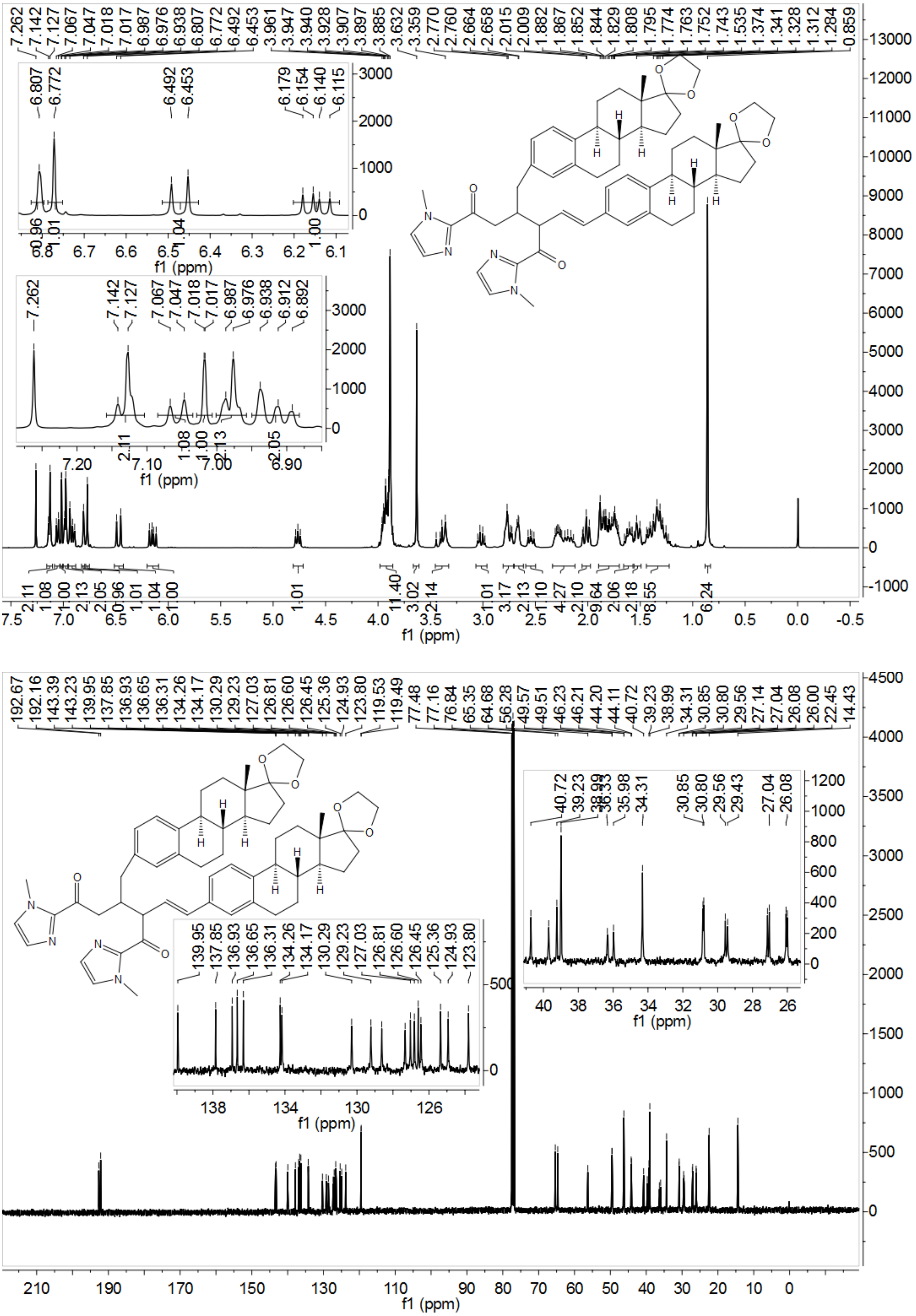

Supplementary Figure 53. ^{1}H and ^{13}C spectra for product **2r**

2s

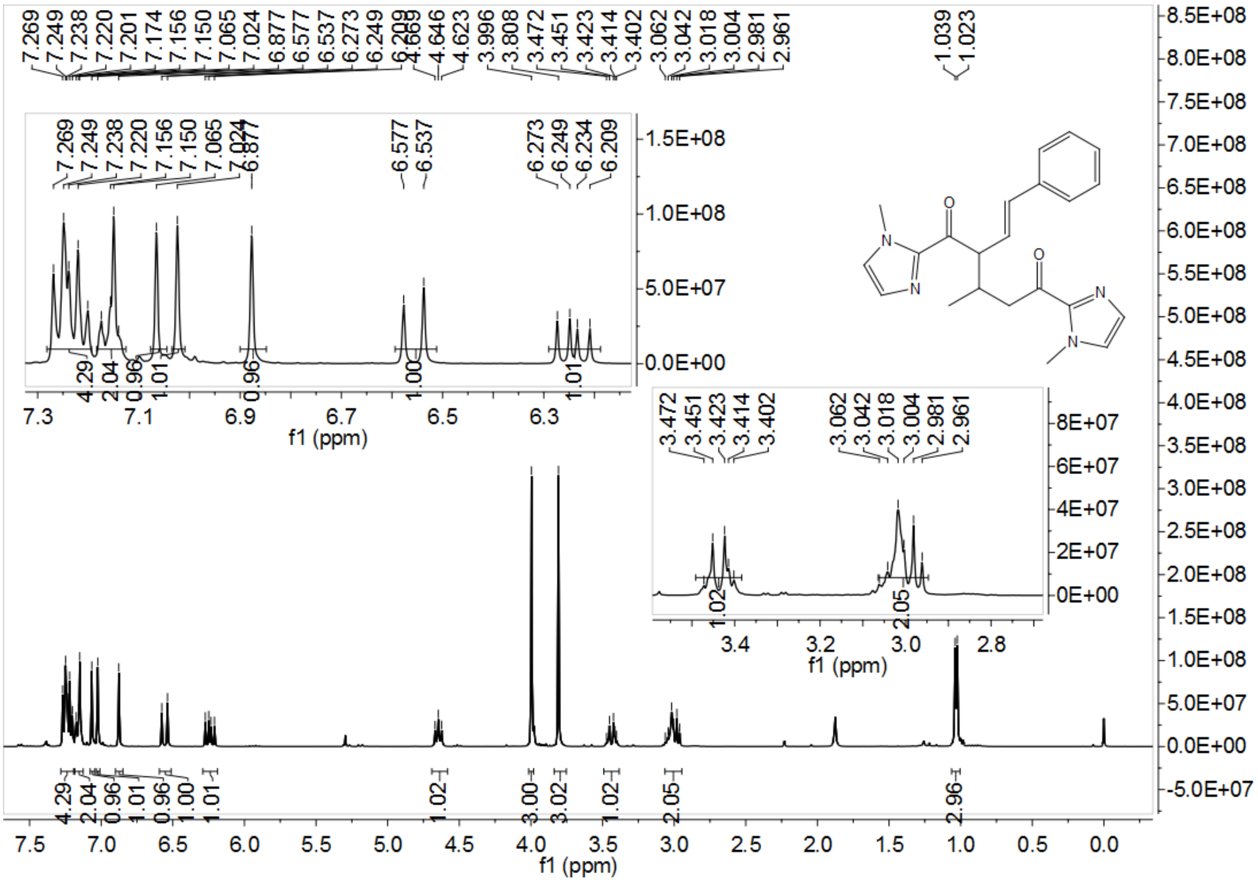

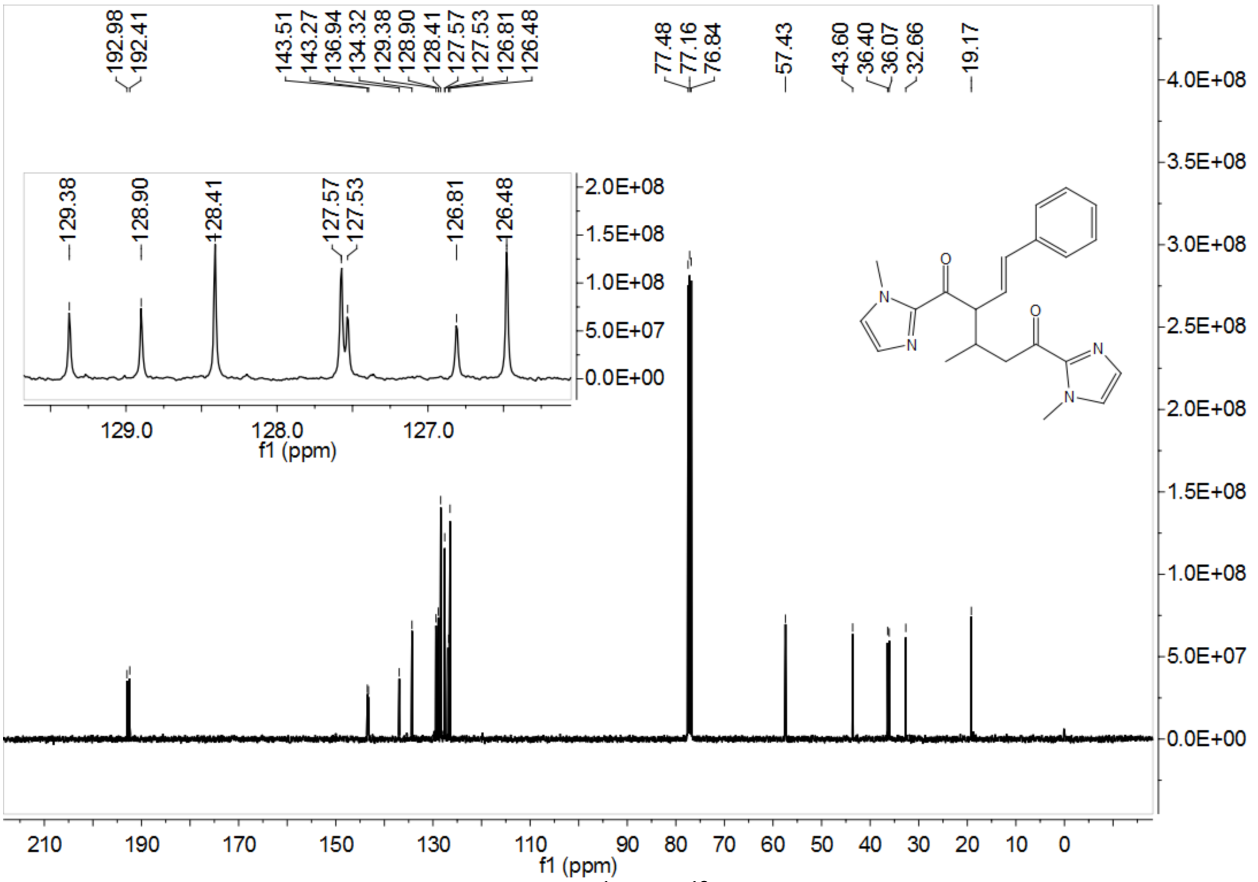

Supplementary Figure 54. ^{1}H and ^{13}C spectra for product **2s**

2t

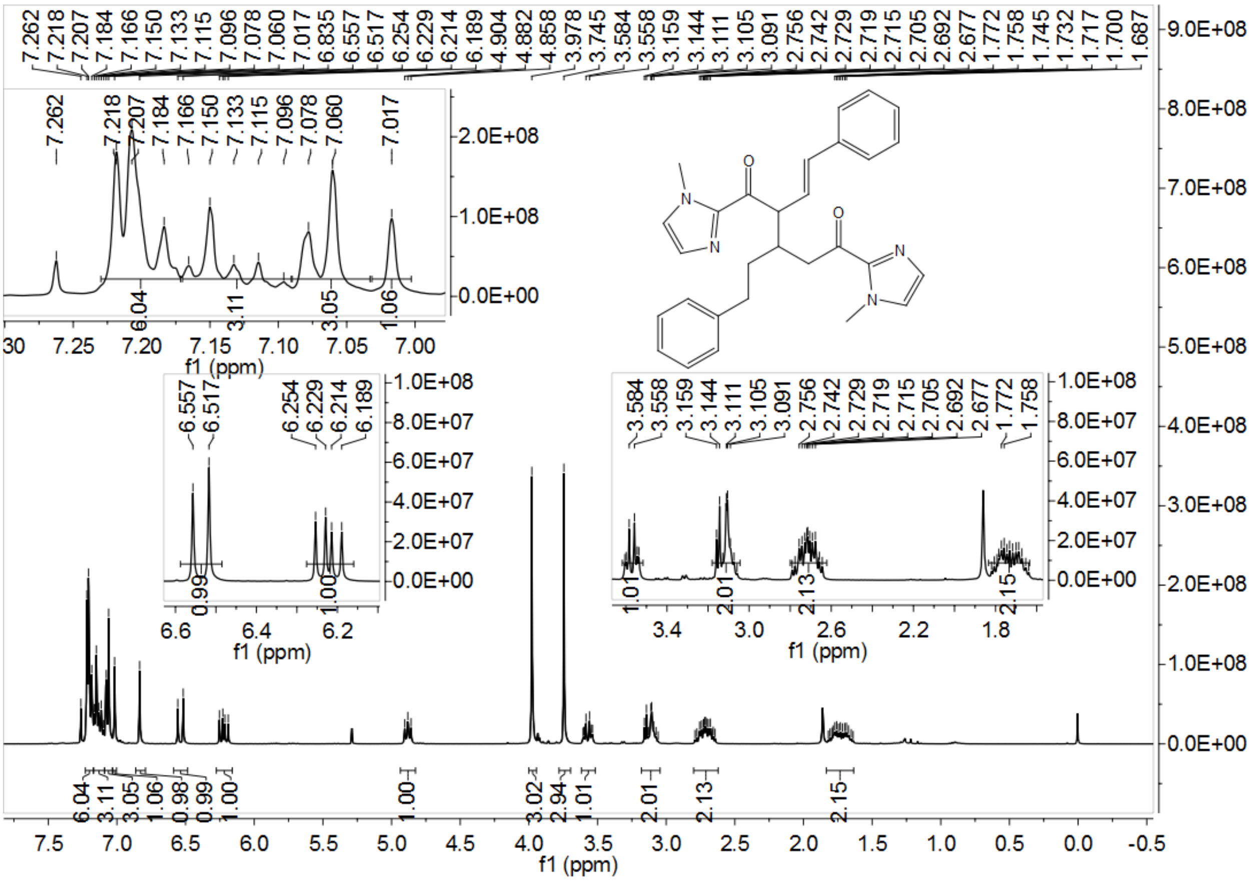

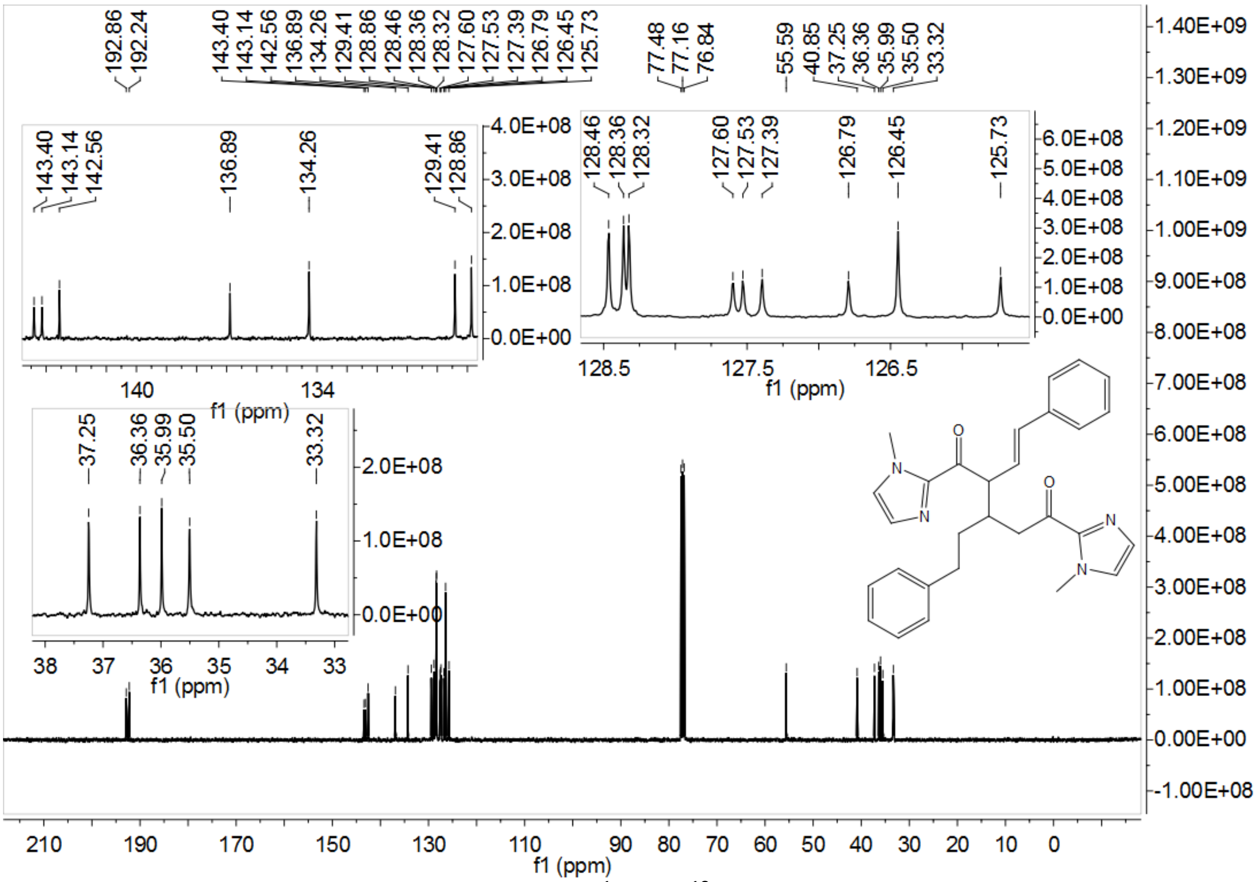

Supplementary Figure 55. ^{1}H and ^{13}C spectra for product **2t**

2u

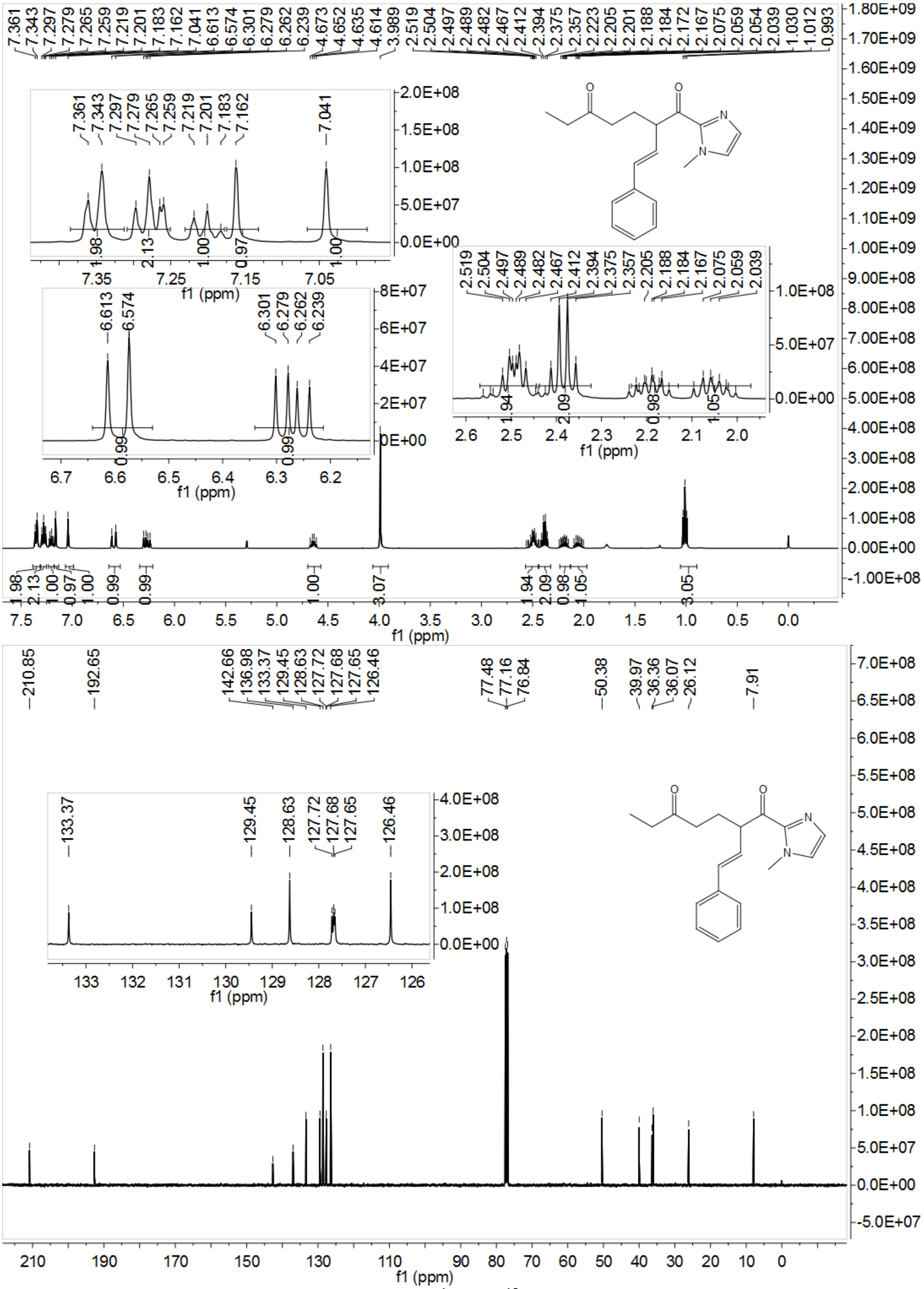

Supplementary Figure 56. ^{1}H and ^{13}C spectra for product **2u**

2v

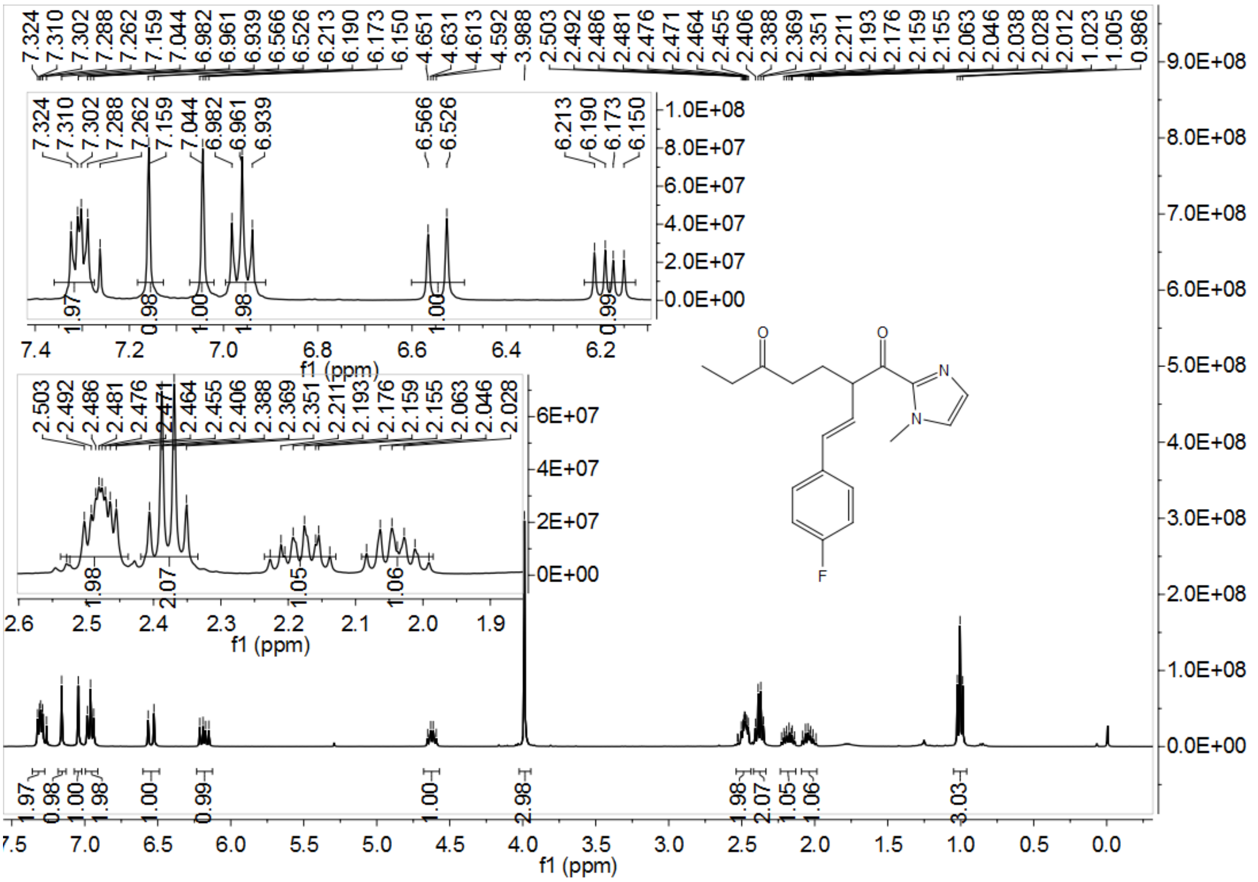

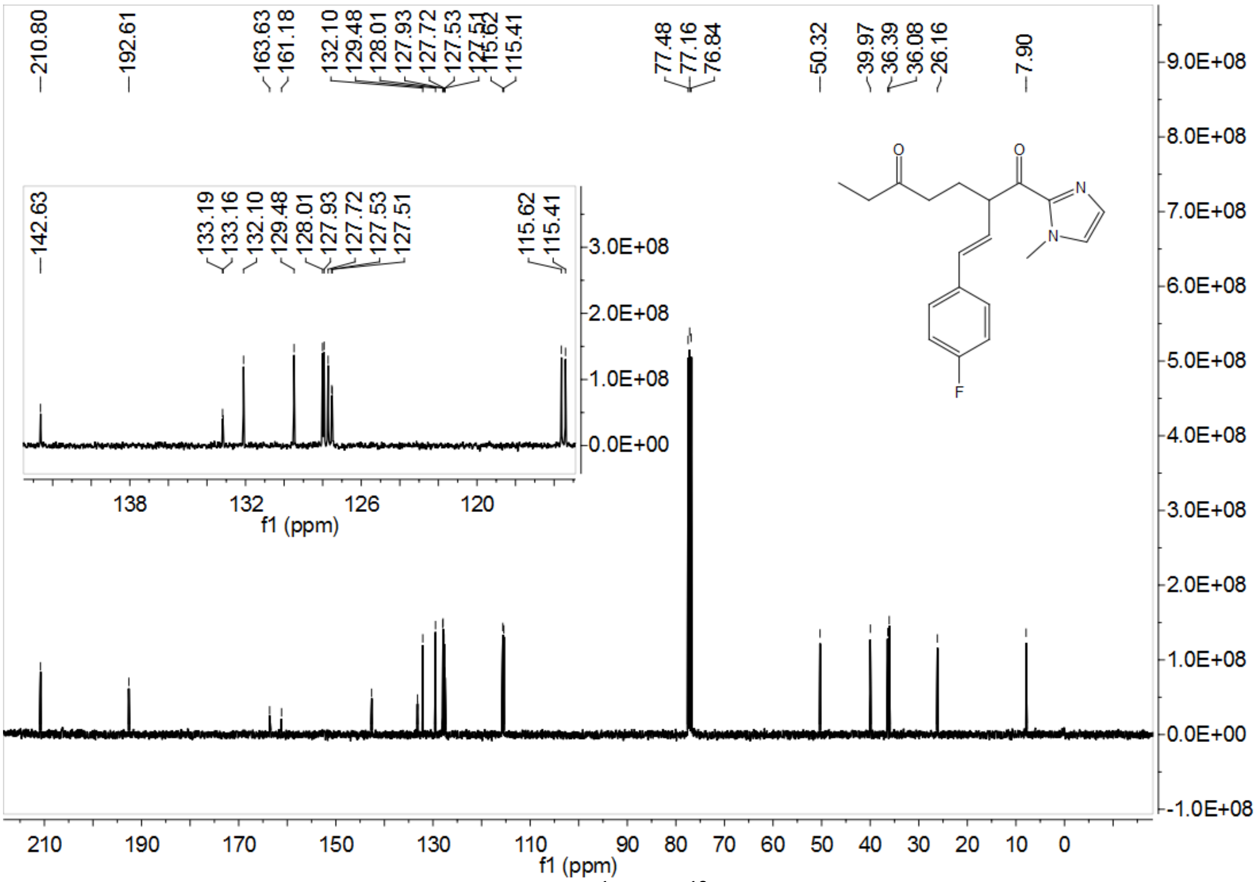

Supplementary Figure 57. ^{1}H and ^{13}C spectra for product **2v**

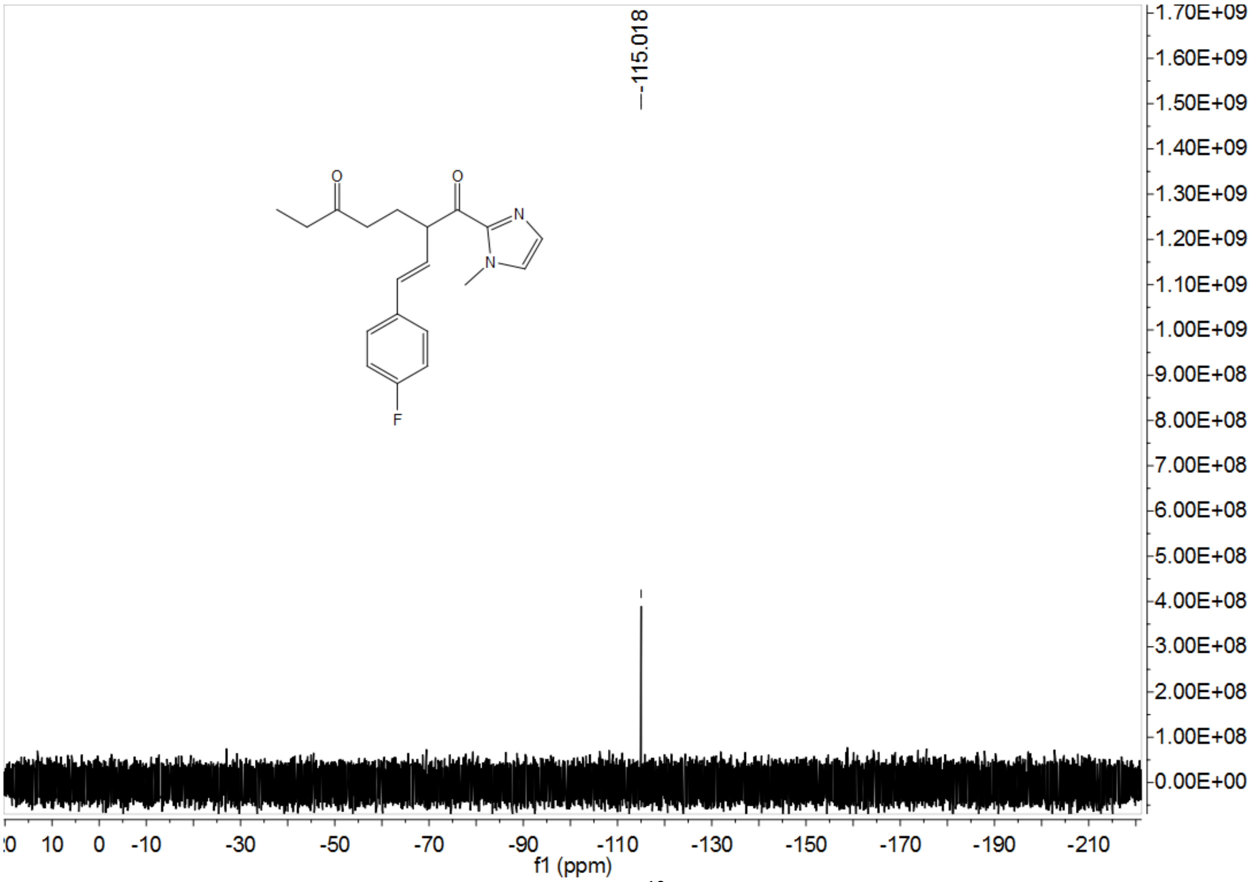

Supplementary Figure 58. ^{19}F spectra for product **2v**

2w

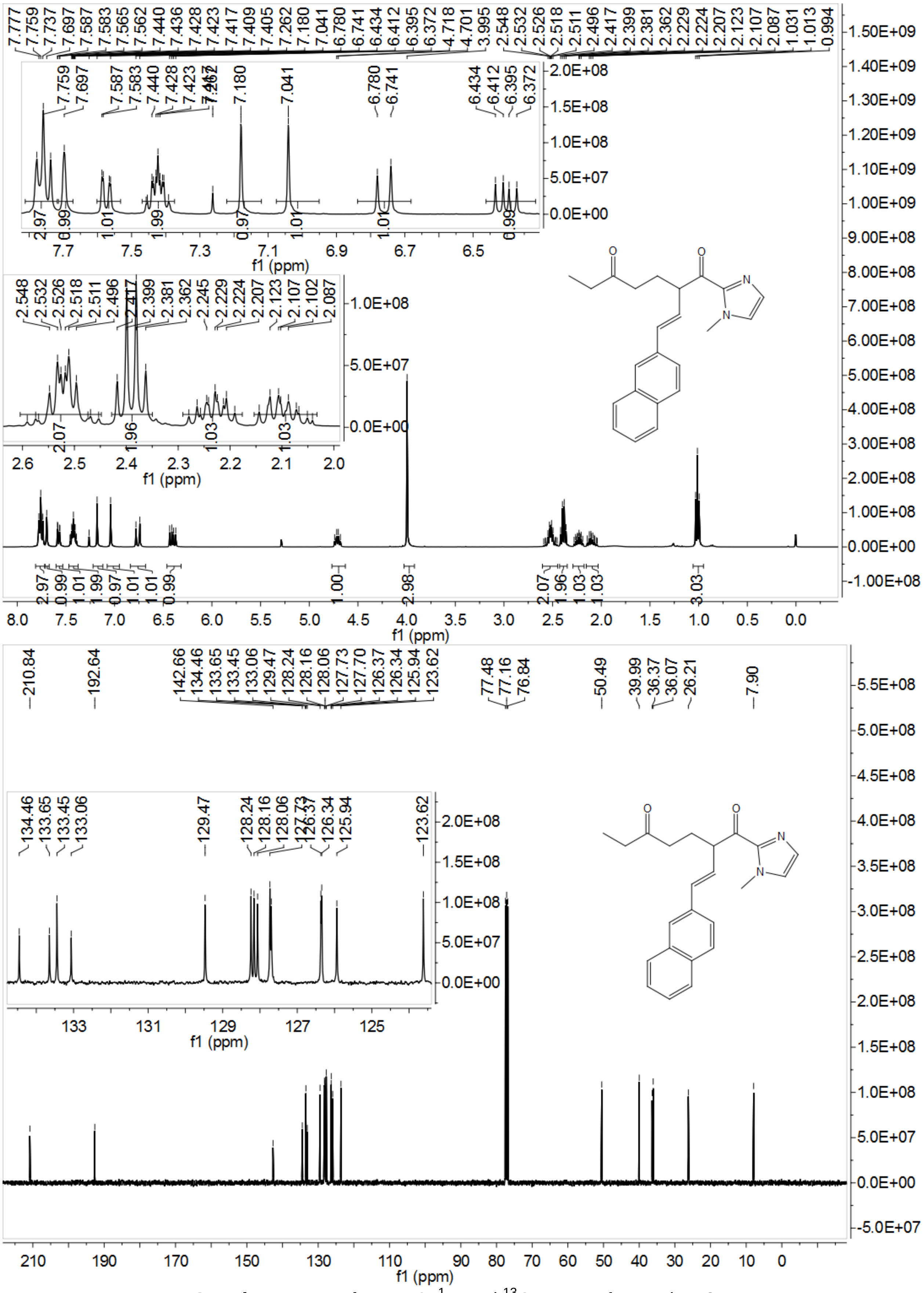

Supplementary Figure 59. ^{1}H and ^{13}C spectra for product **2w**

4a

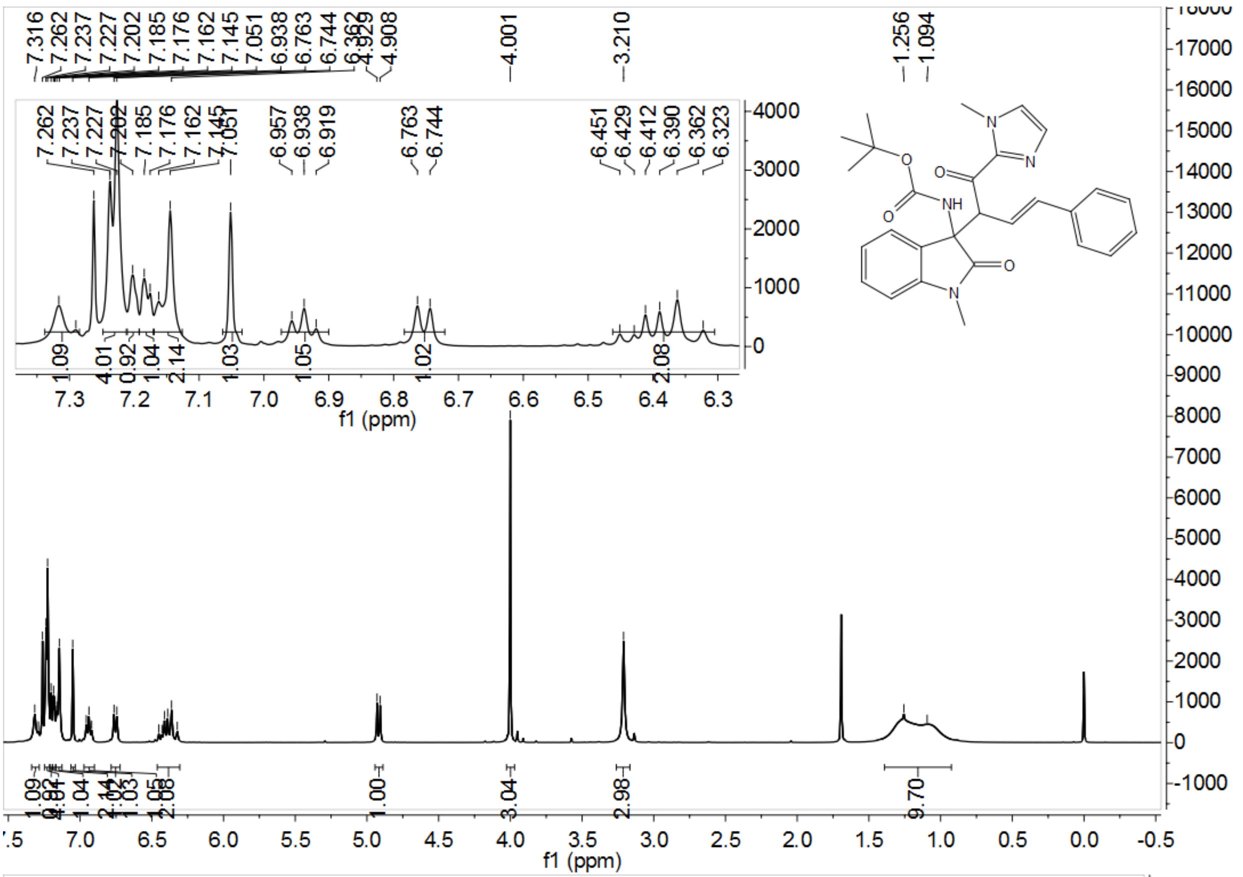

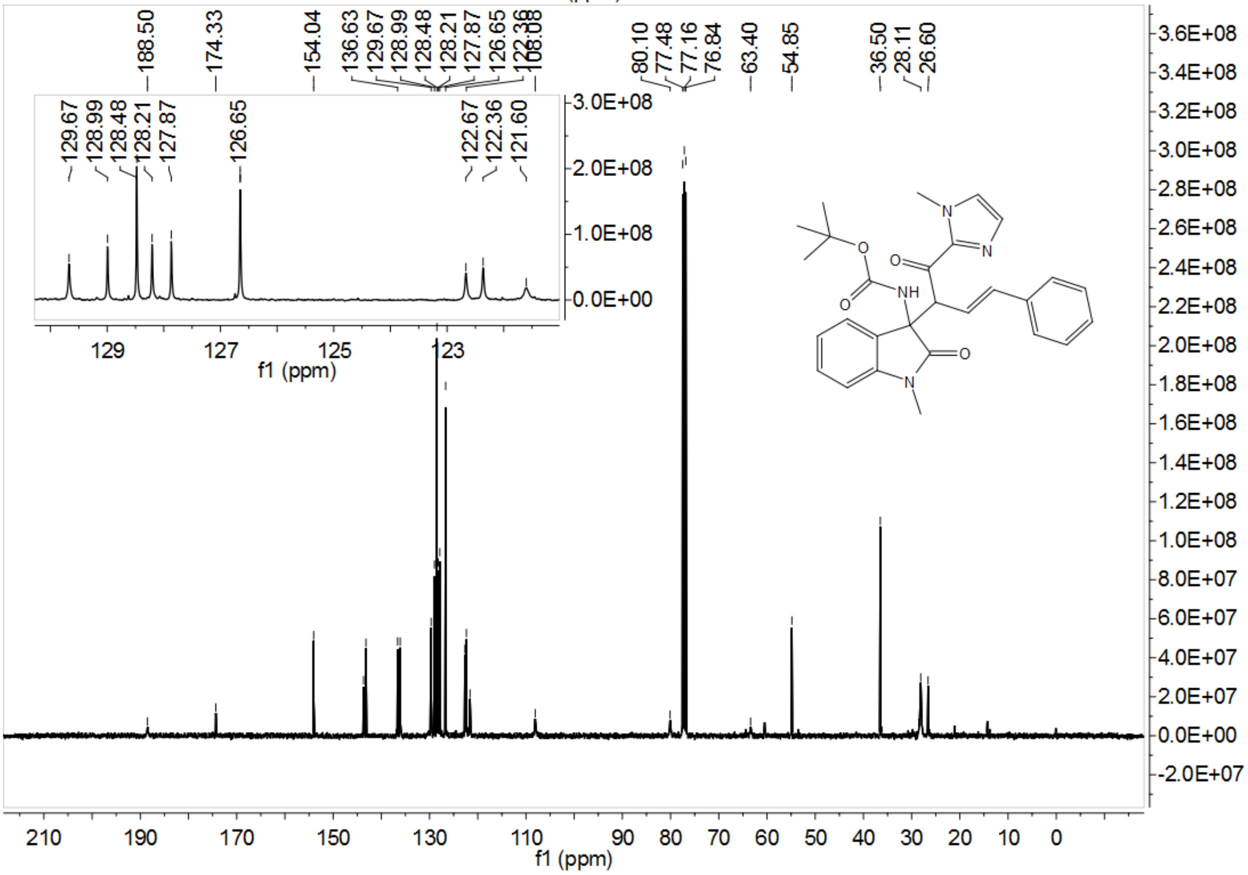

Supplementary Figure 60. ^{1}H and ^{13}C spectra for product **4a**

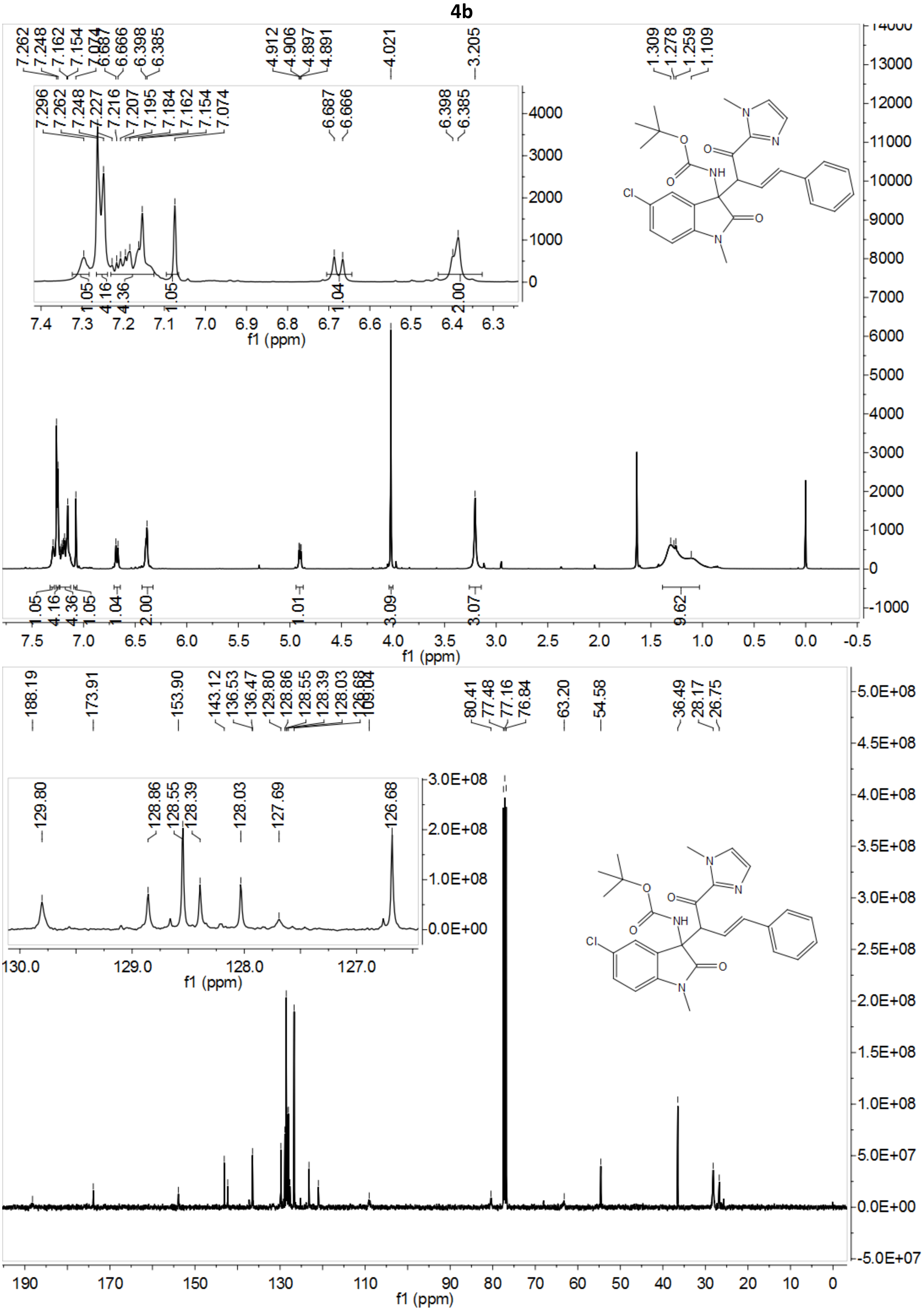

Supplementary Figure 61. ^{1}H and ^{13}C spectra for product **4b**

4c

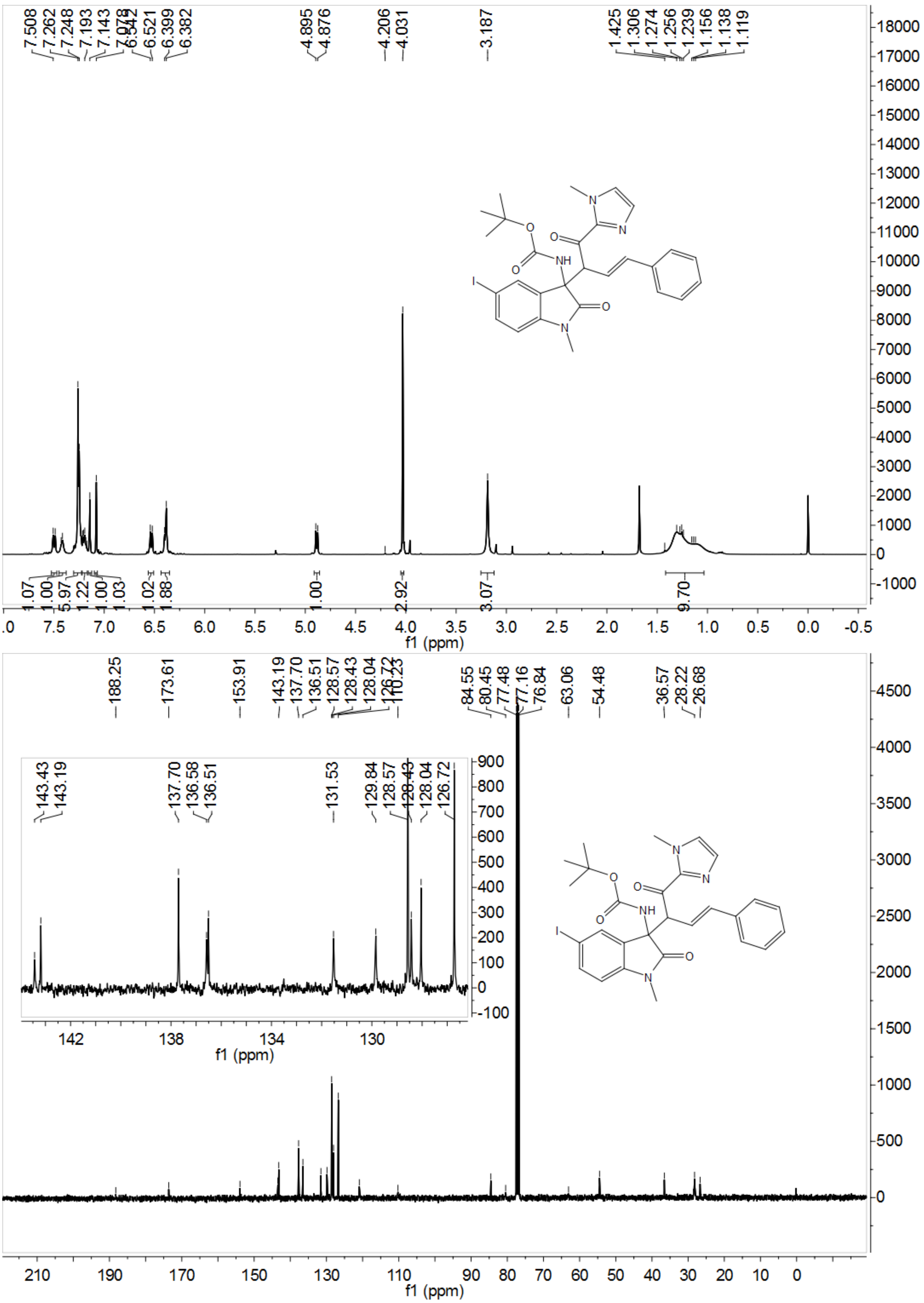

Supplementary Figure 62. ^{1}H and ^{13}C spectra for product **4c**

4d

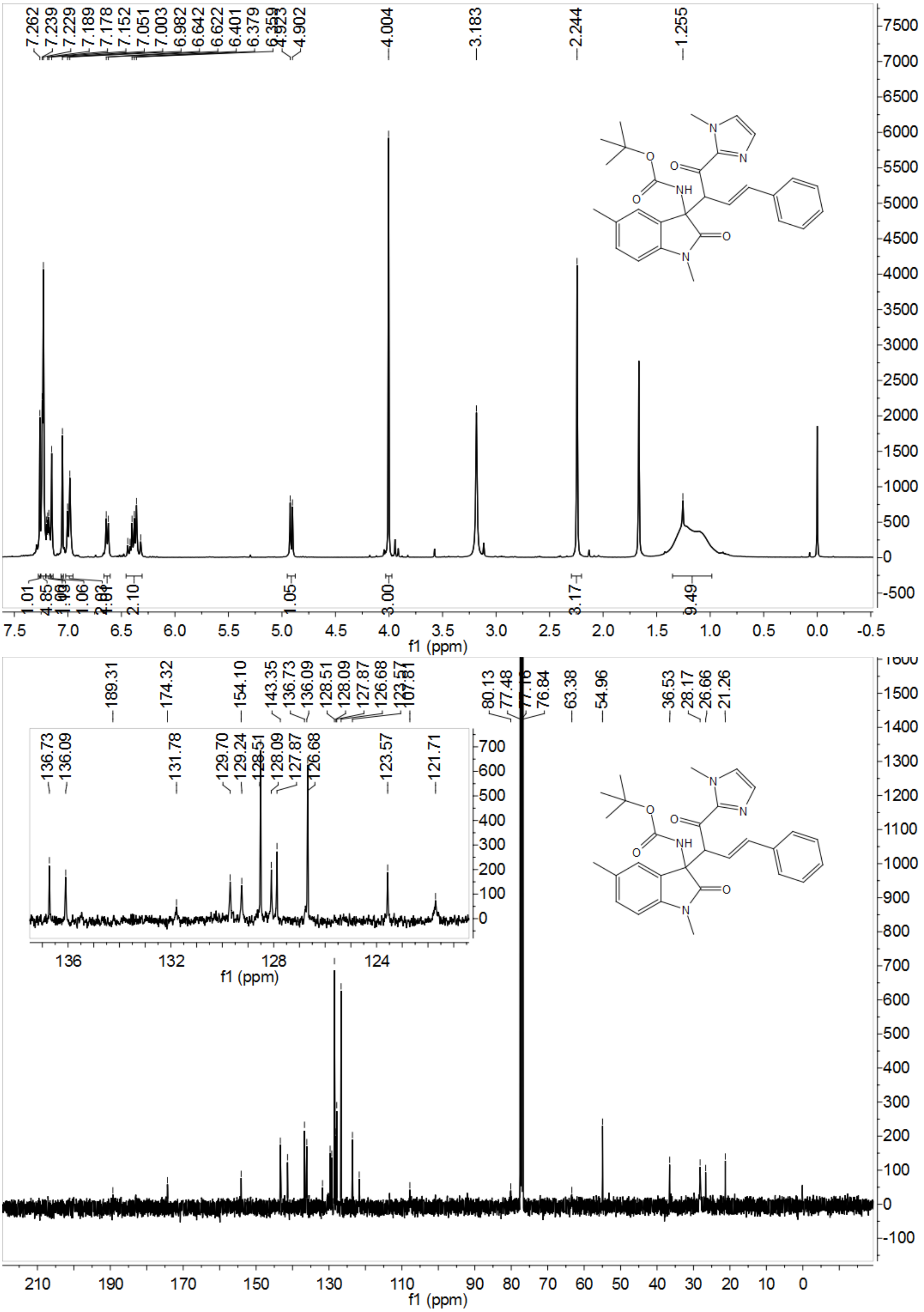

Supplementary Figure 63. ^{1}H and ^{13}C spectra for product **4d**

4e

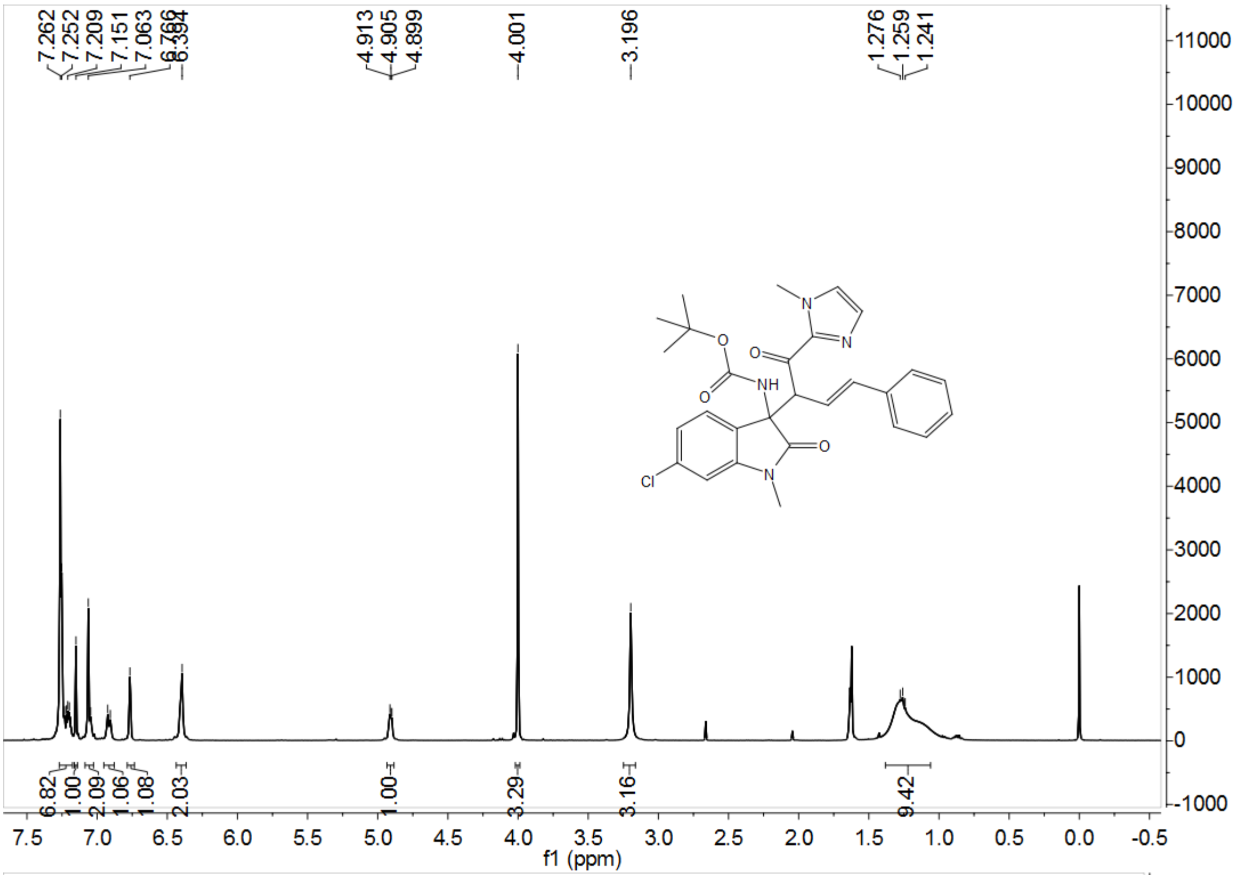

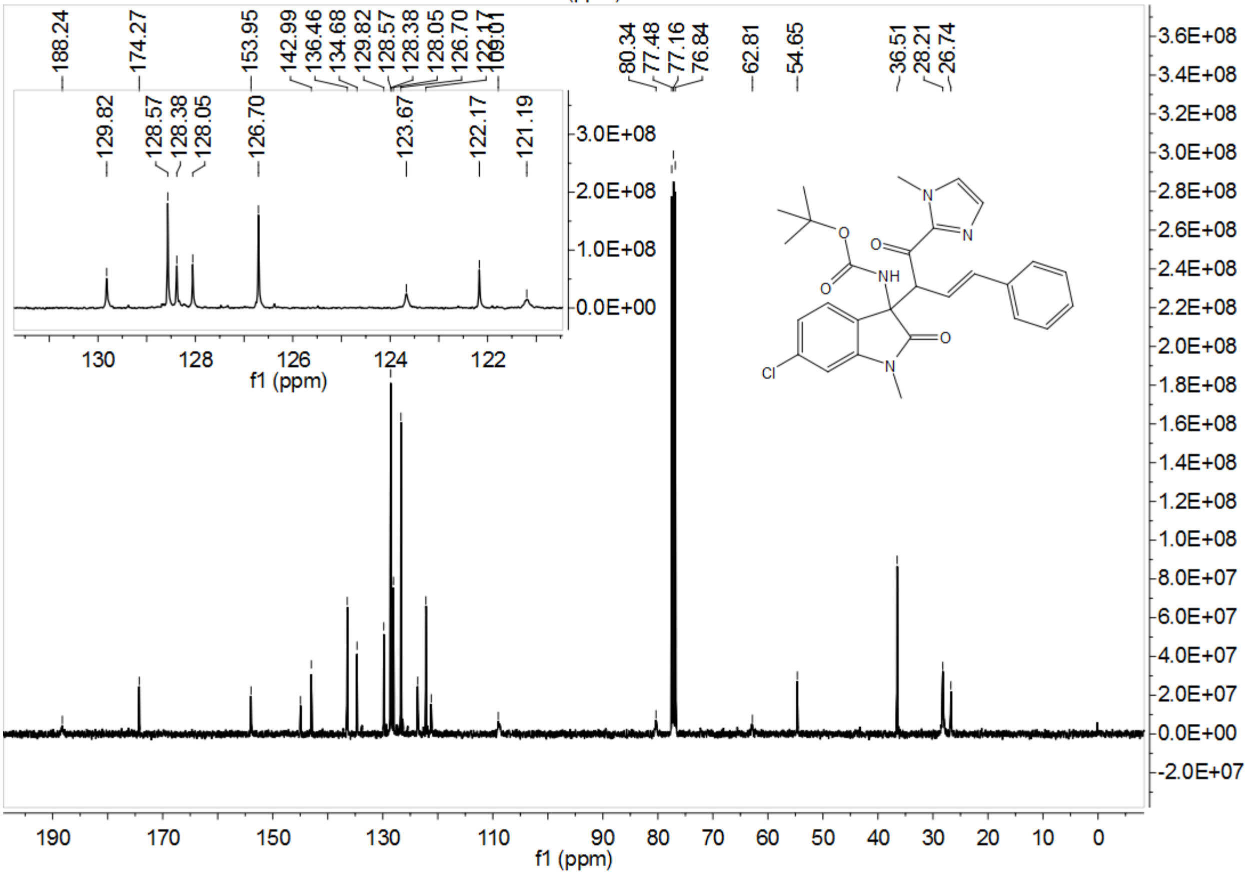

Supplementary Figure 64. ^{1}H and ^{13}C spectra for product **4e**

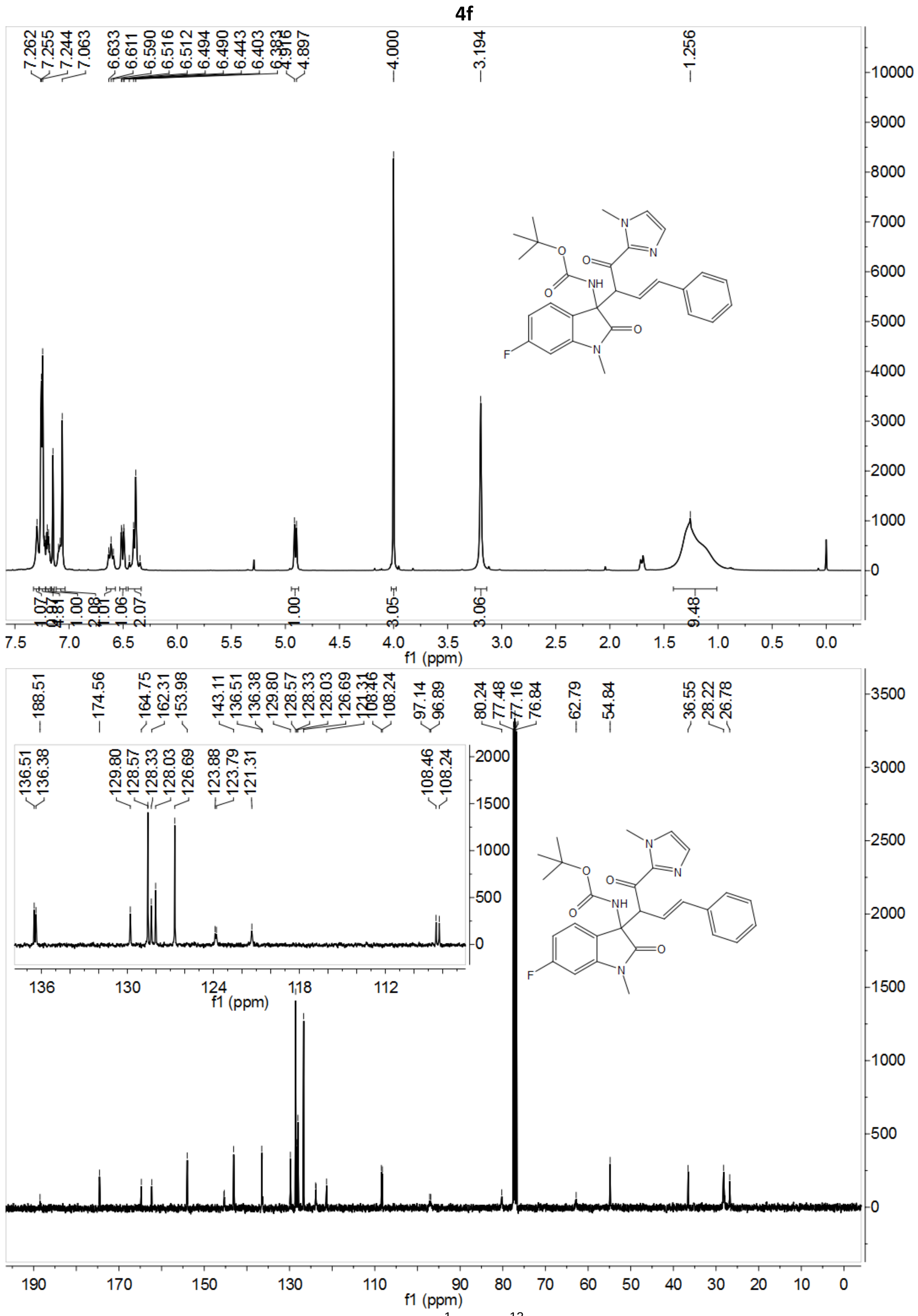

Supplementary Figure 65. ^{1}H and ^{13}C spectra for product **4f**

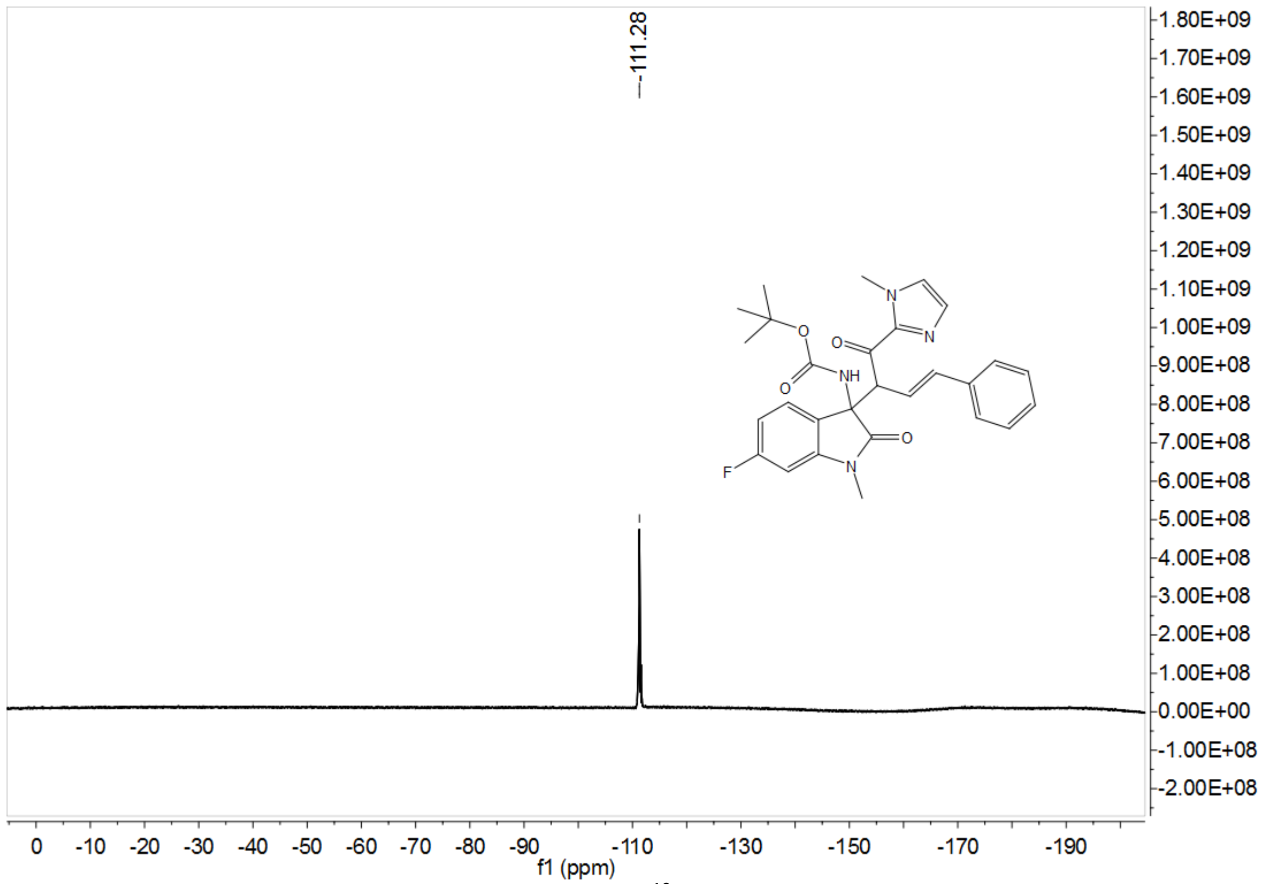

Supplementary Figure 66. ^{19}F spectra for product **4f**

4g

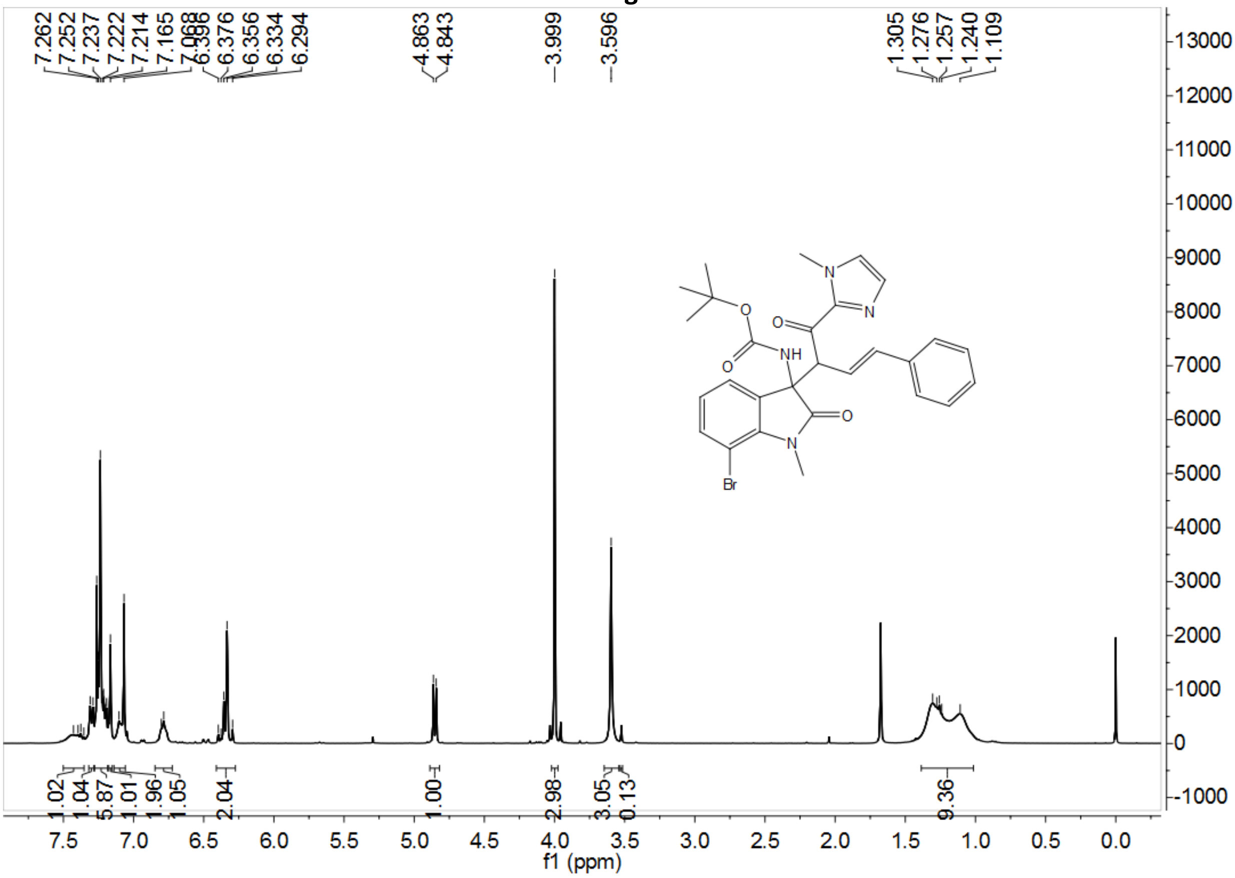

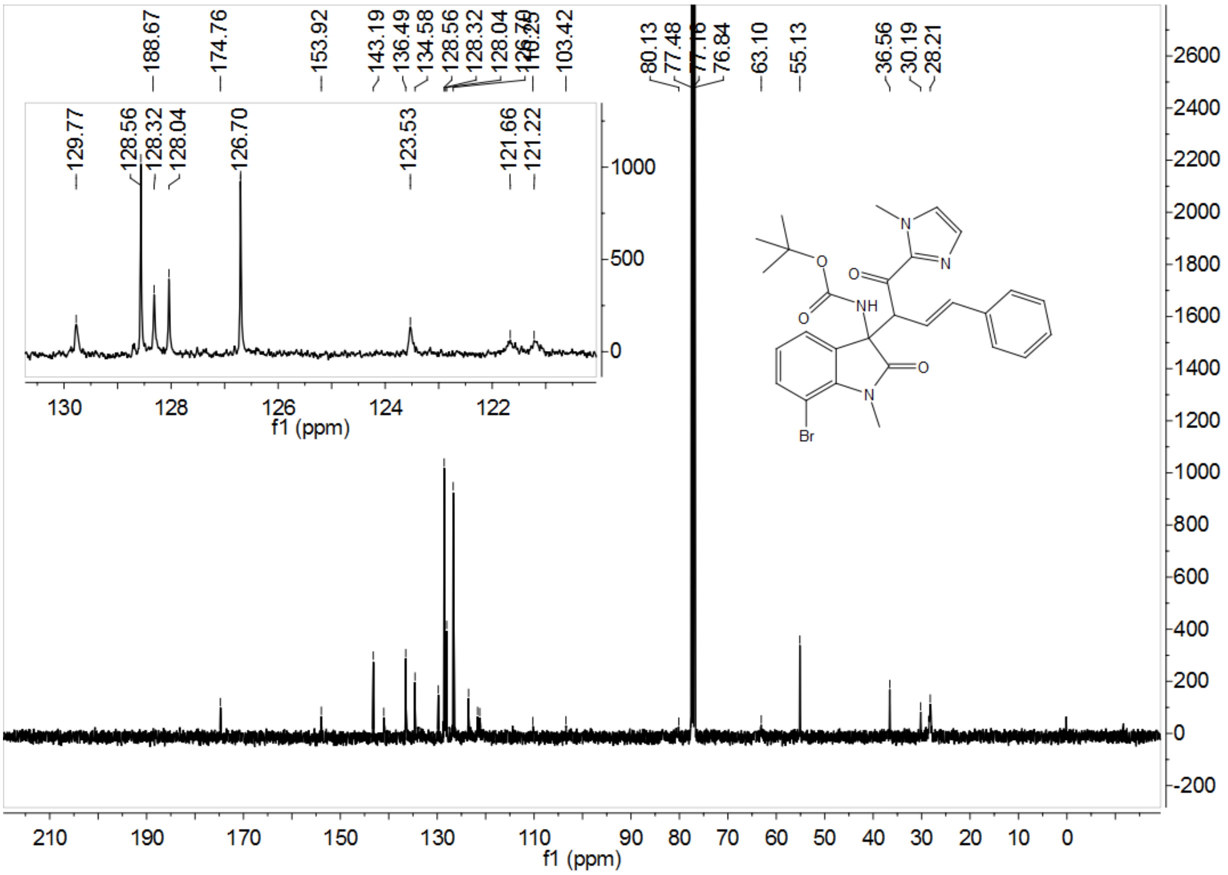

Supplementary Figure 67. ^{1}H and ^{13}C spectra for product **4g**

4h

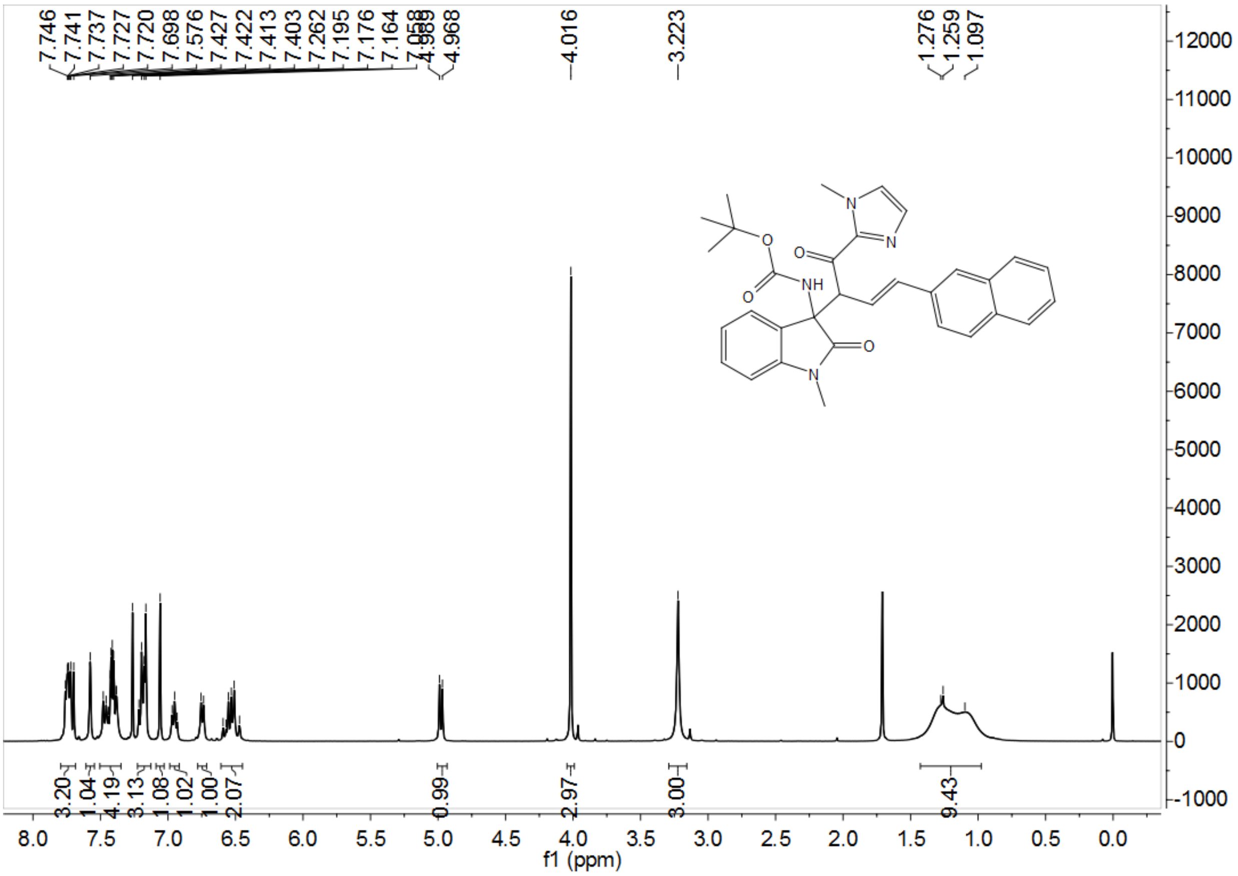

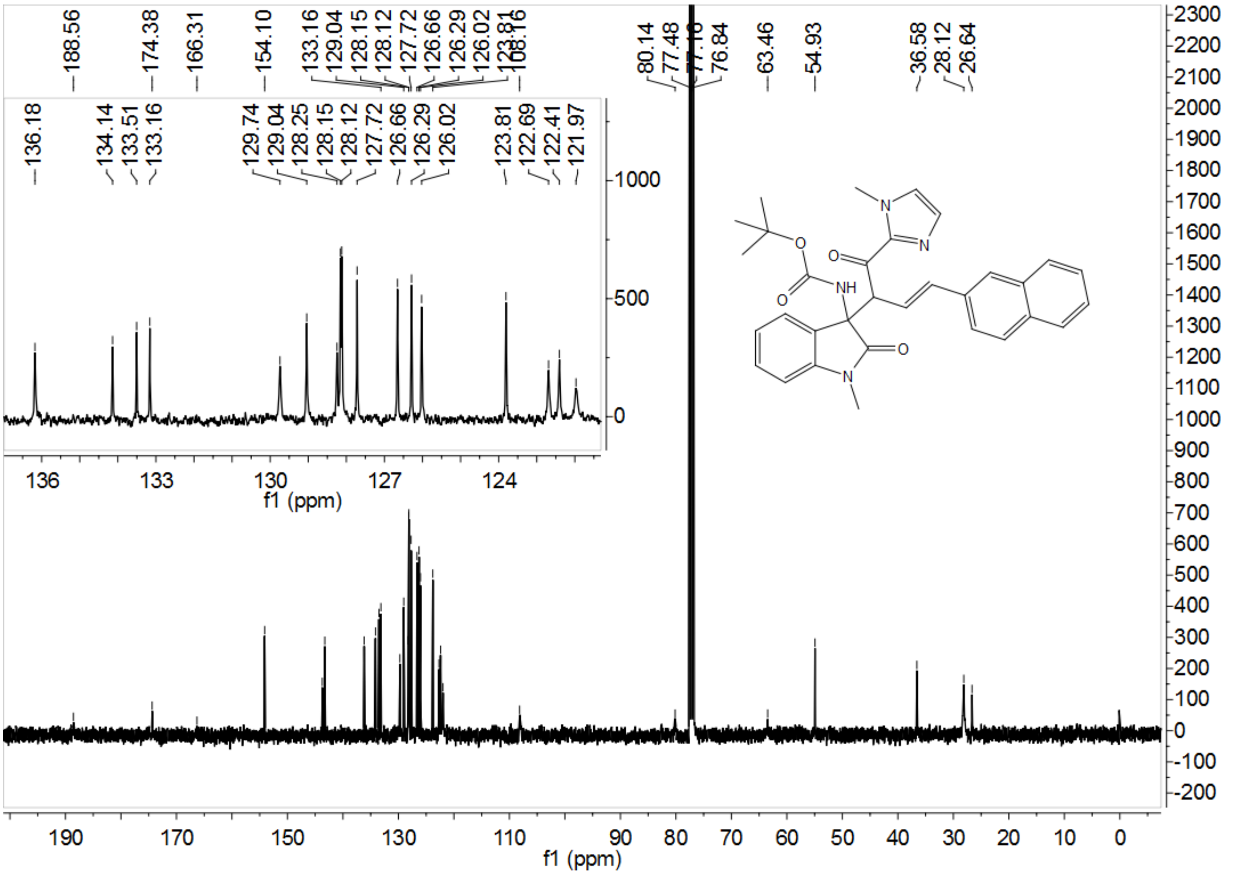

Supplementary Figure 68. ^{1}H and ^{13}C spectra for product **4h**

4i

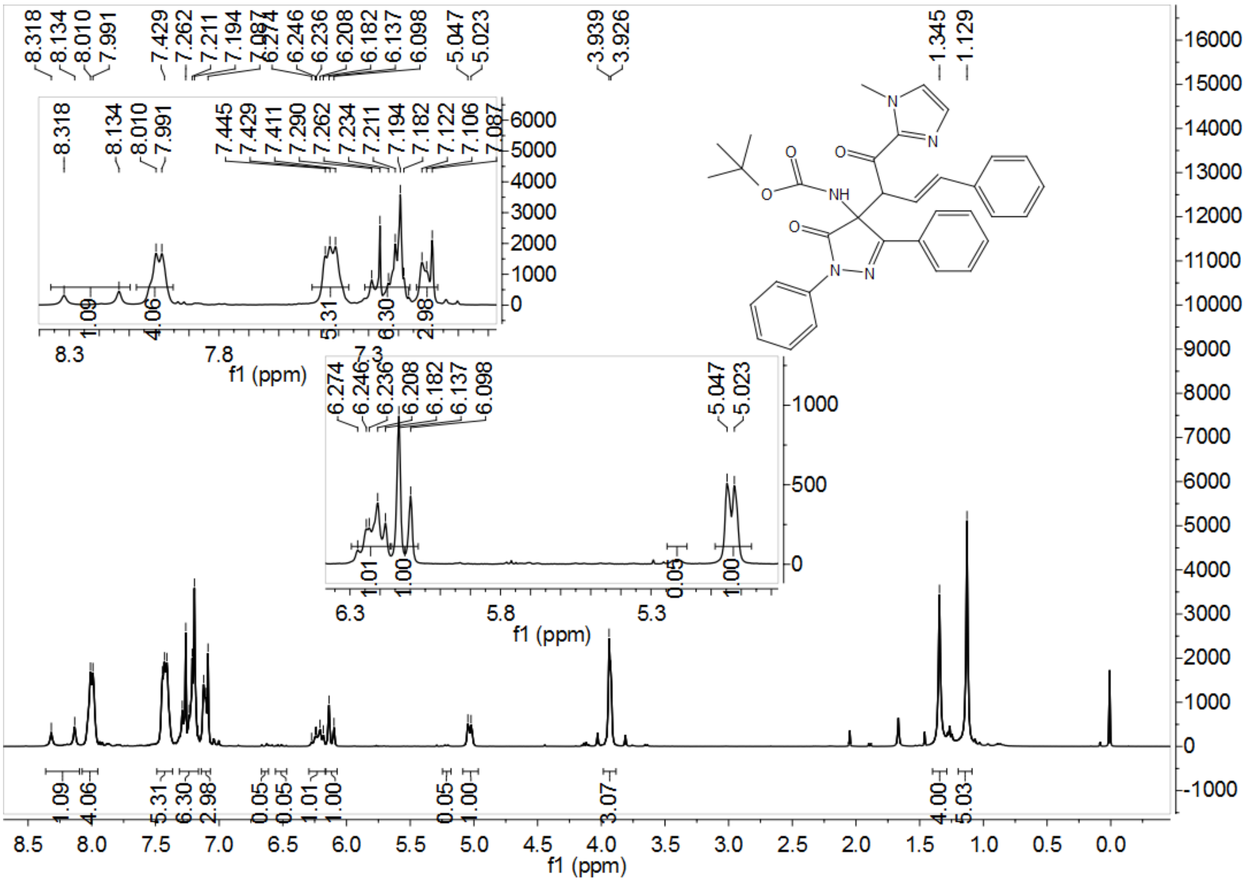

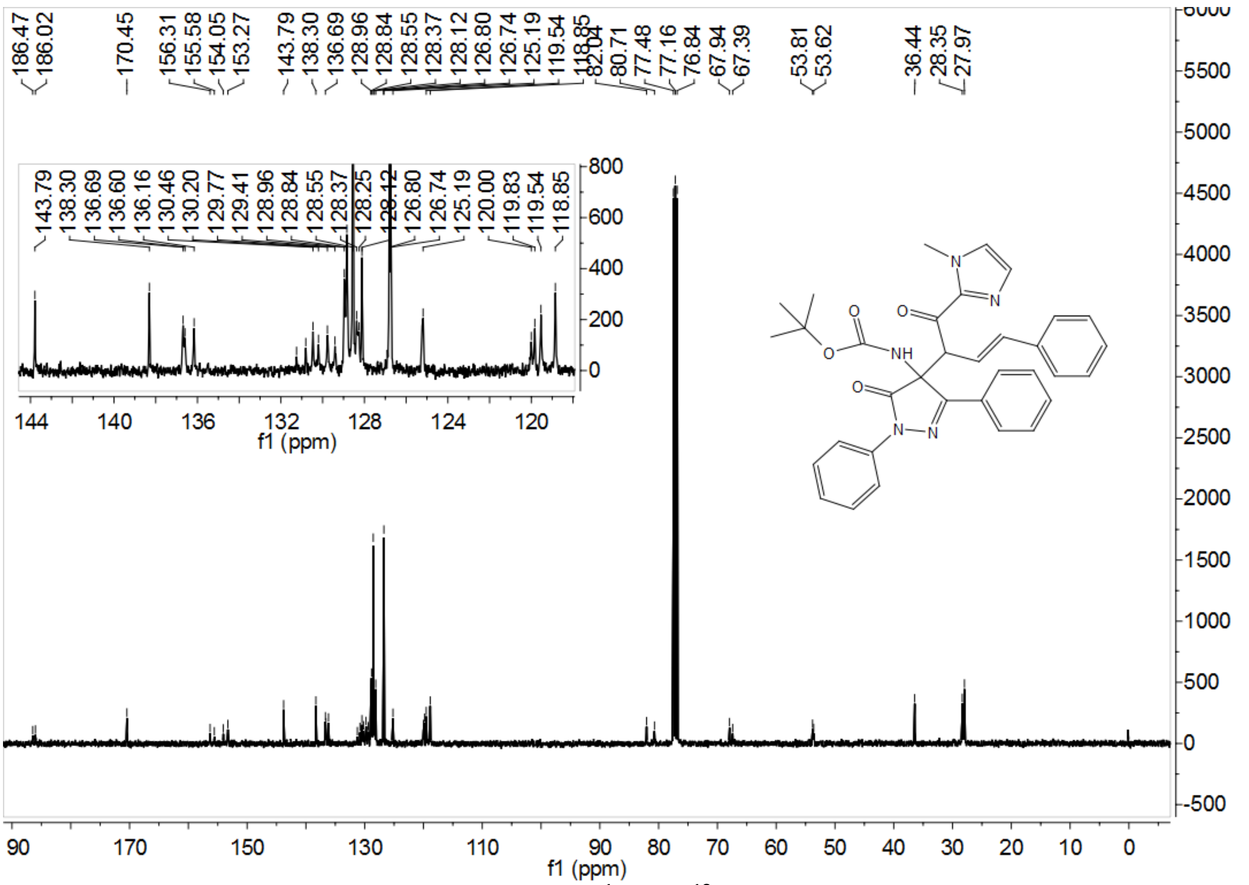

Supplementary Figure 69. ^{1}H and ^{13}C spectra for product **4i**

4j

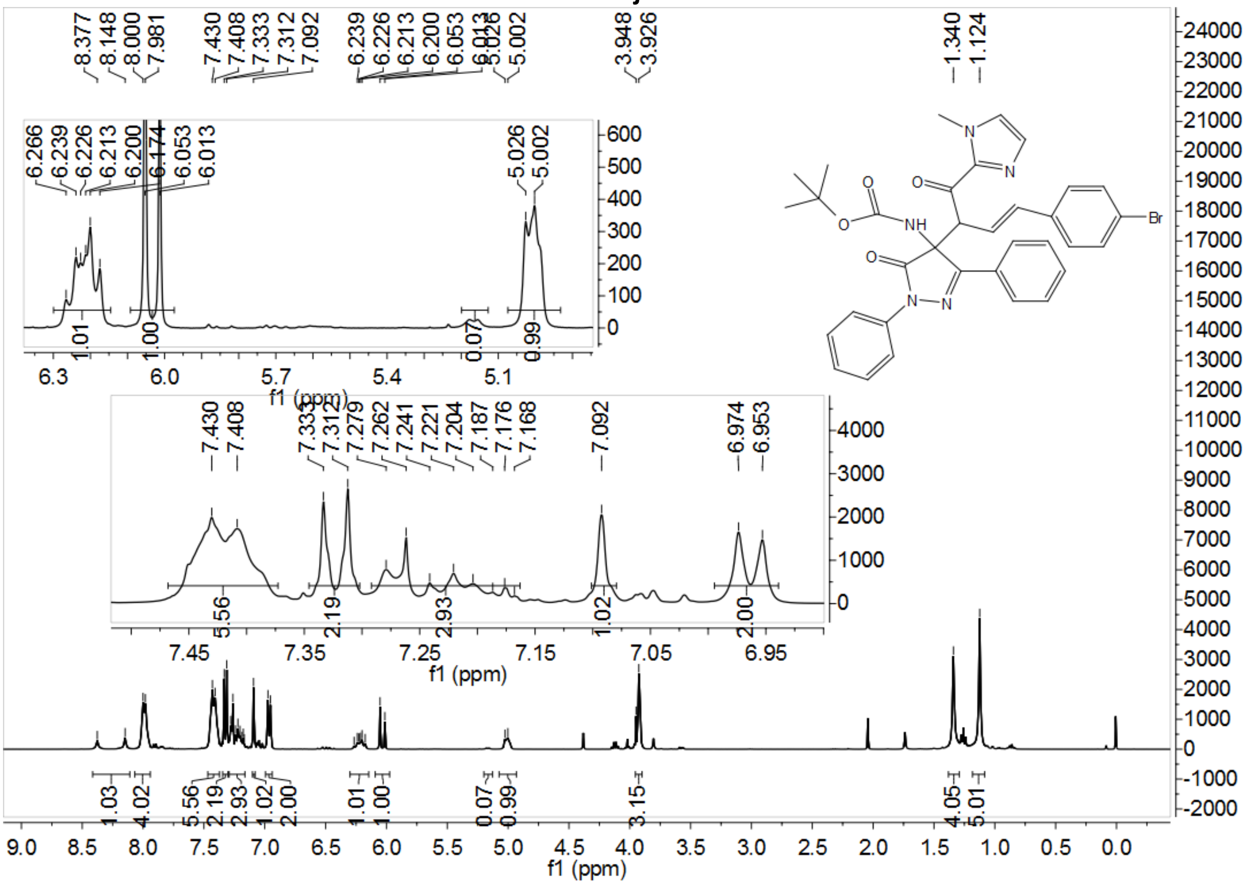

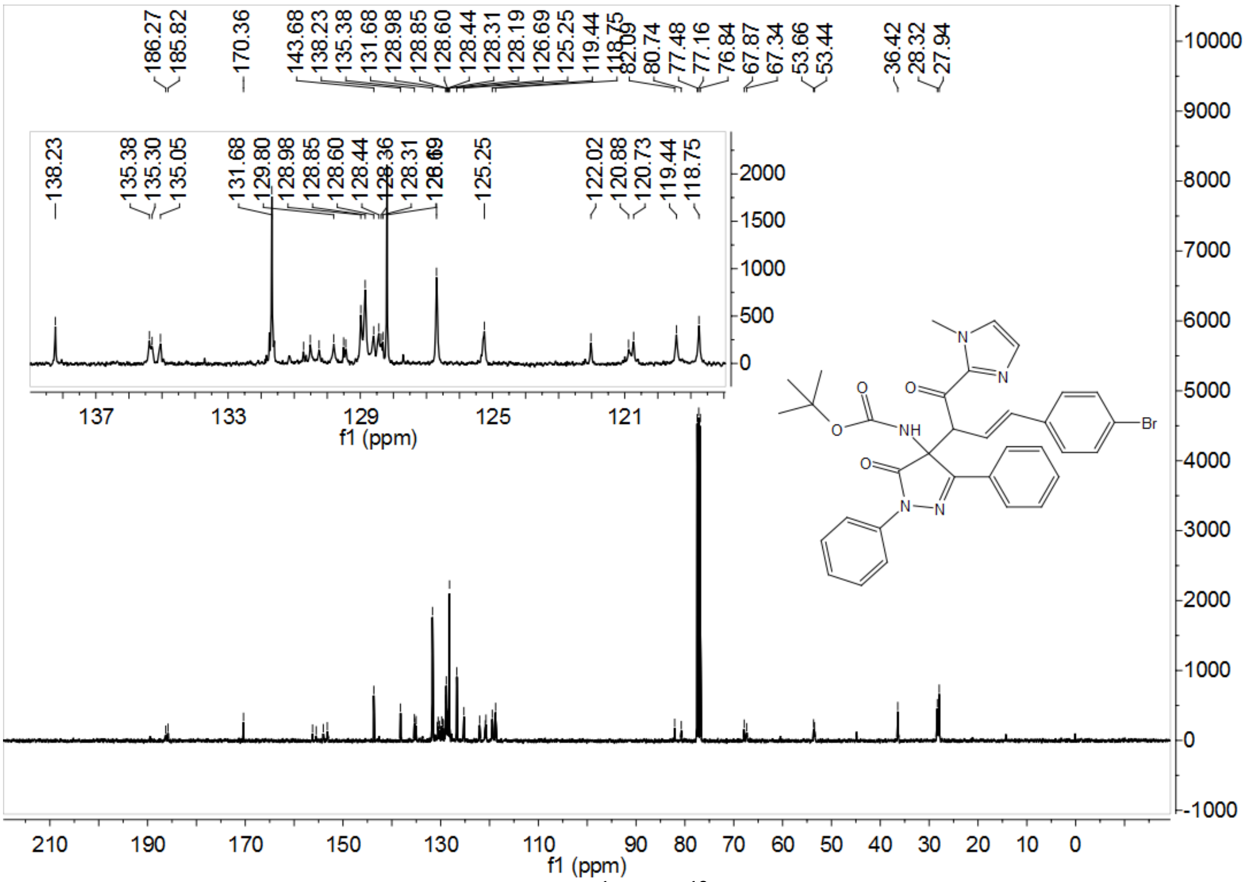

Supplementary Figure 70. ^{1}H and ^{13}C spectra for product **4g**

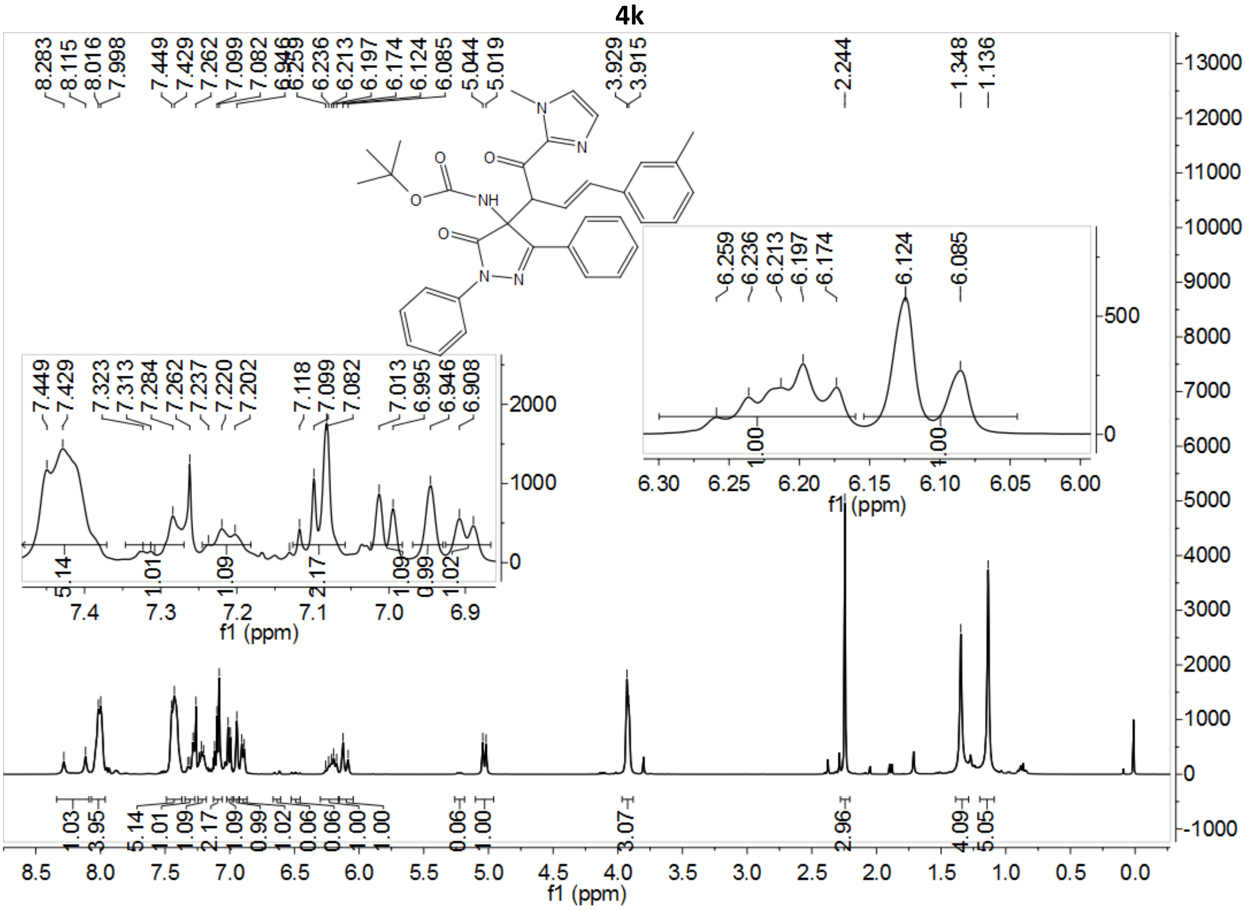

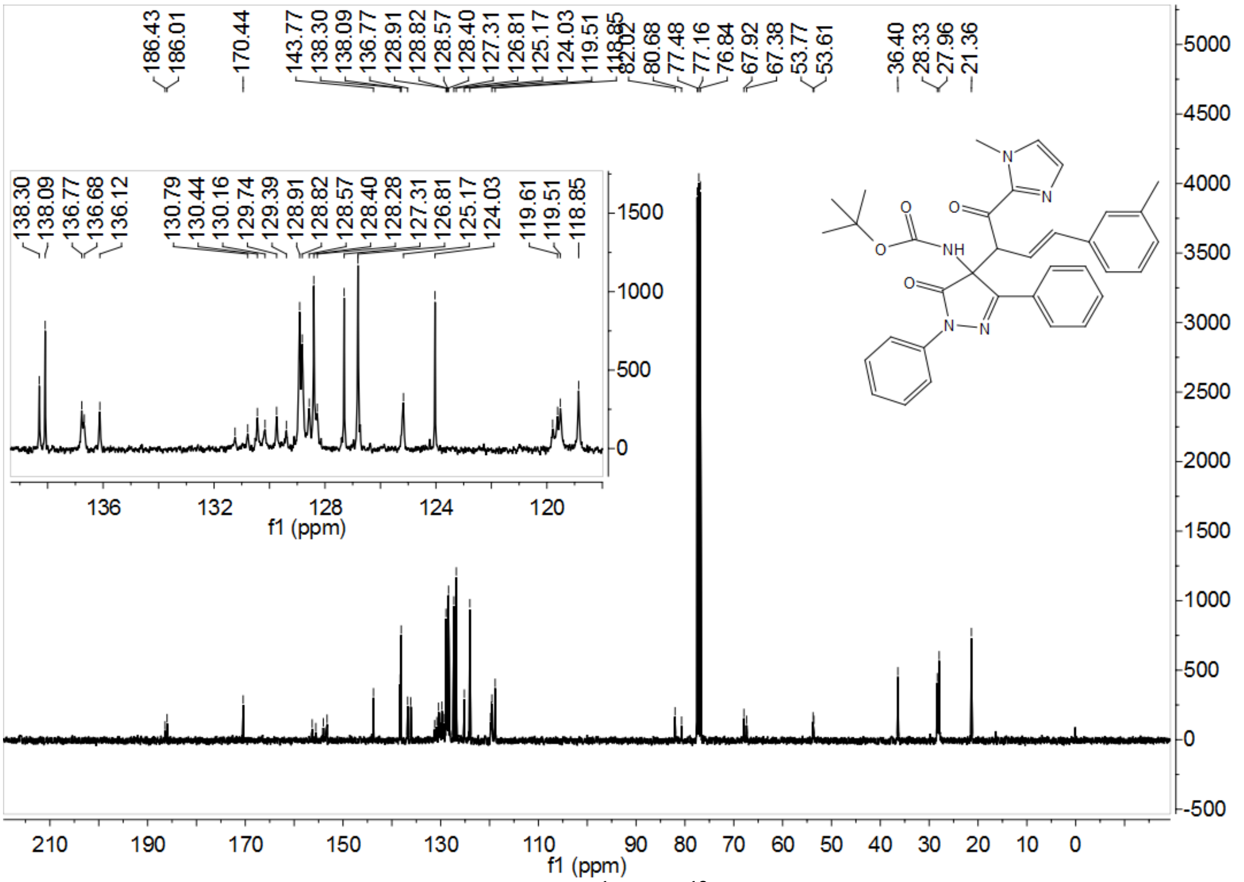

Supplementary Figure 71. ^{1}H and ^{13}C spectra for product **4k**

4l

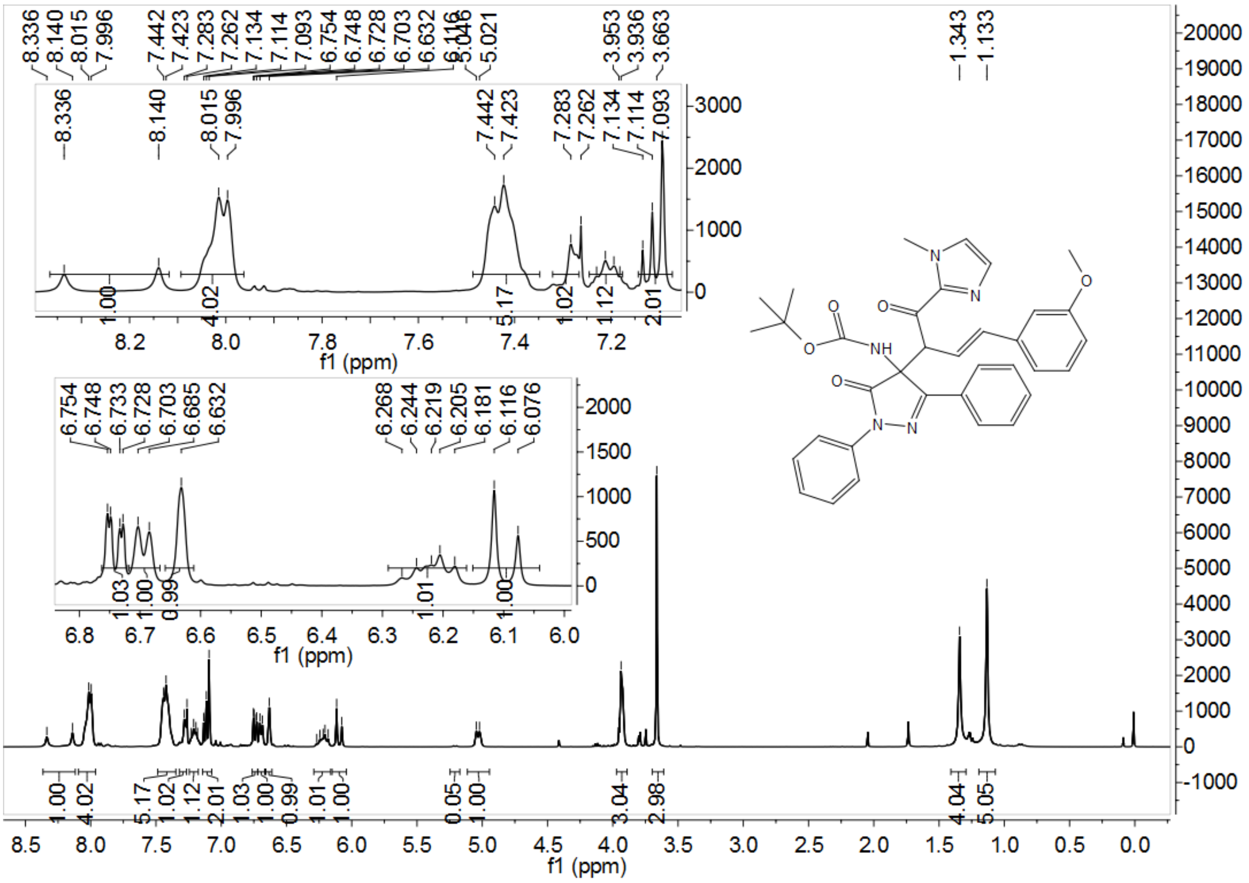

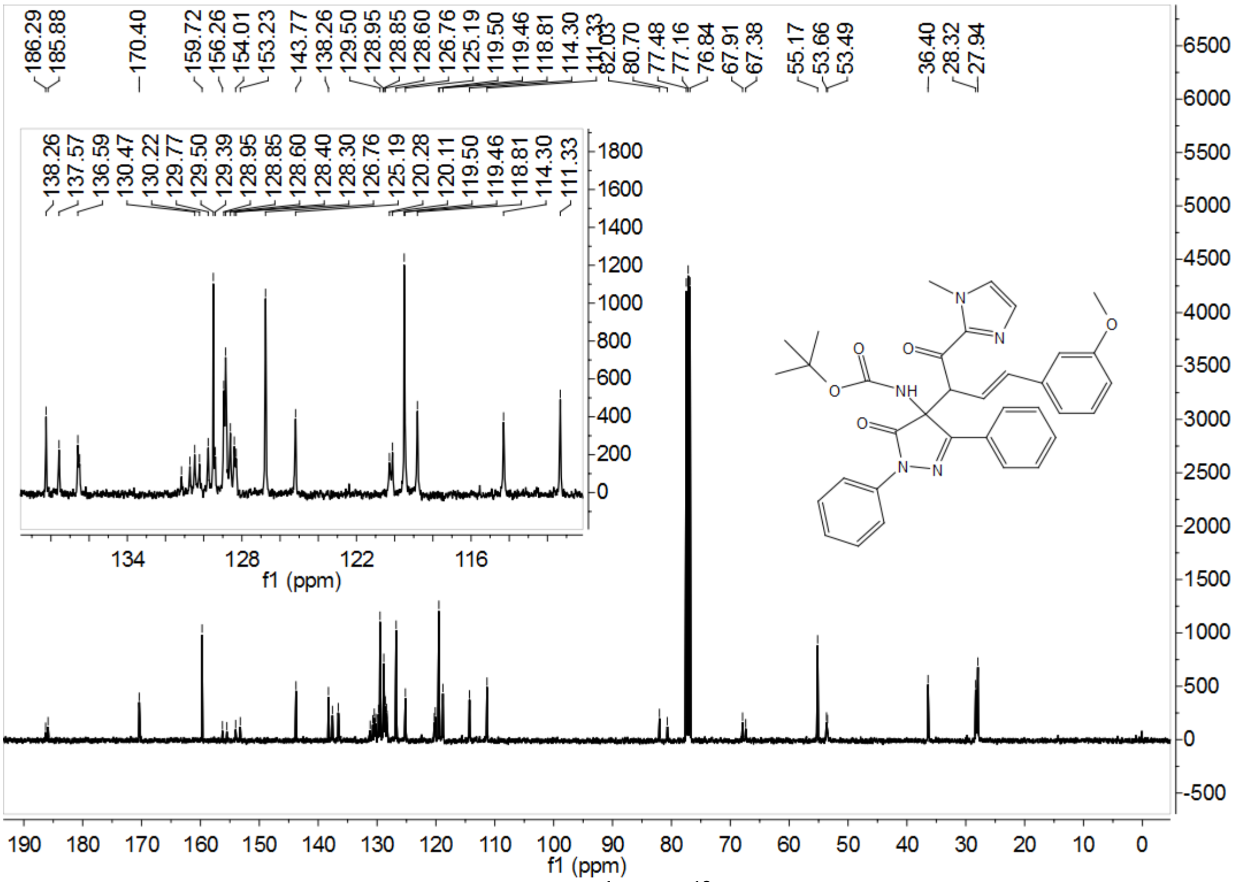

Supplementary Figure 72. ^{1}H and ^{13}C spectra for product **4l**

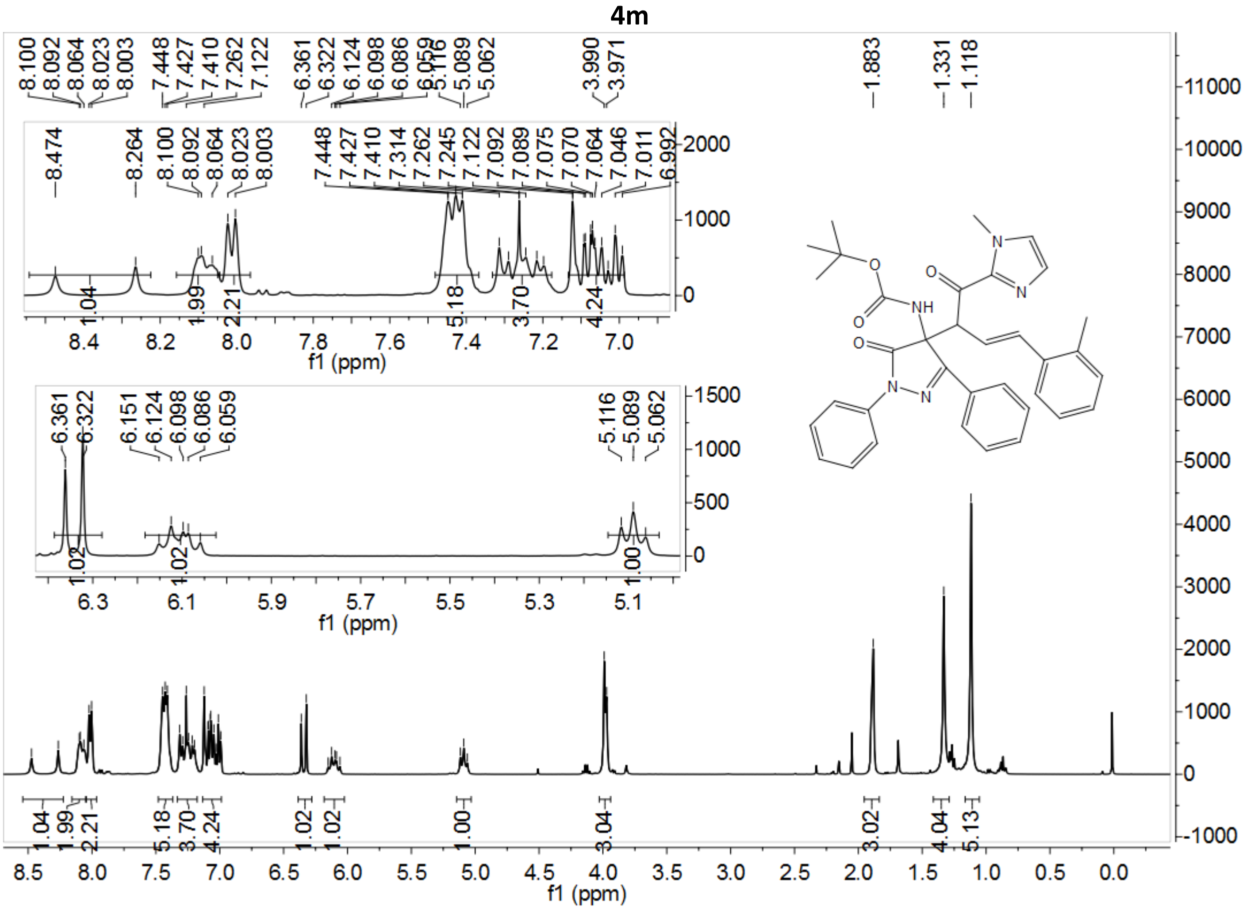

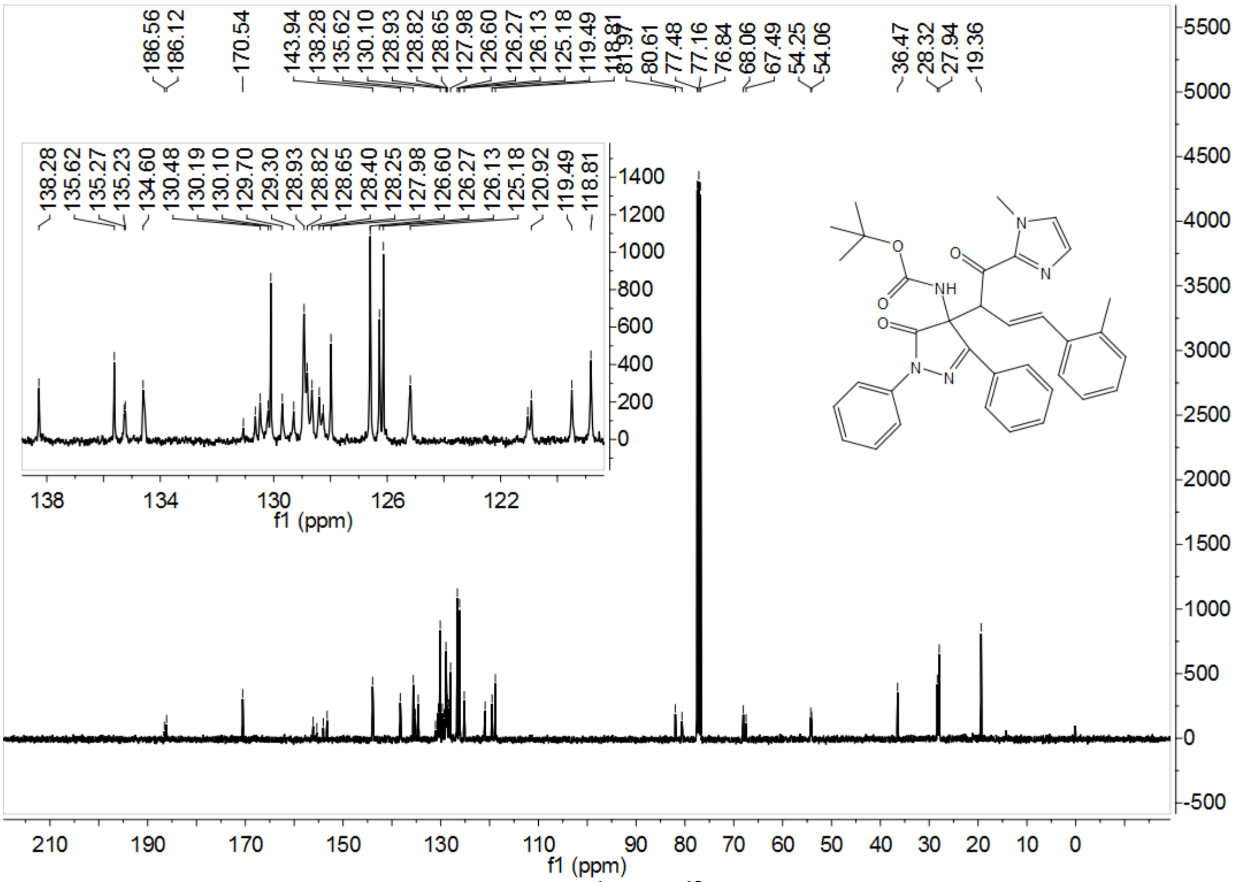

Supplementary Figure 73. ^{1}H and ^{13}C spectra for product **4m**

4n

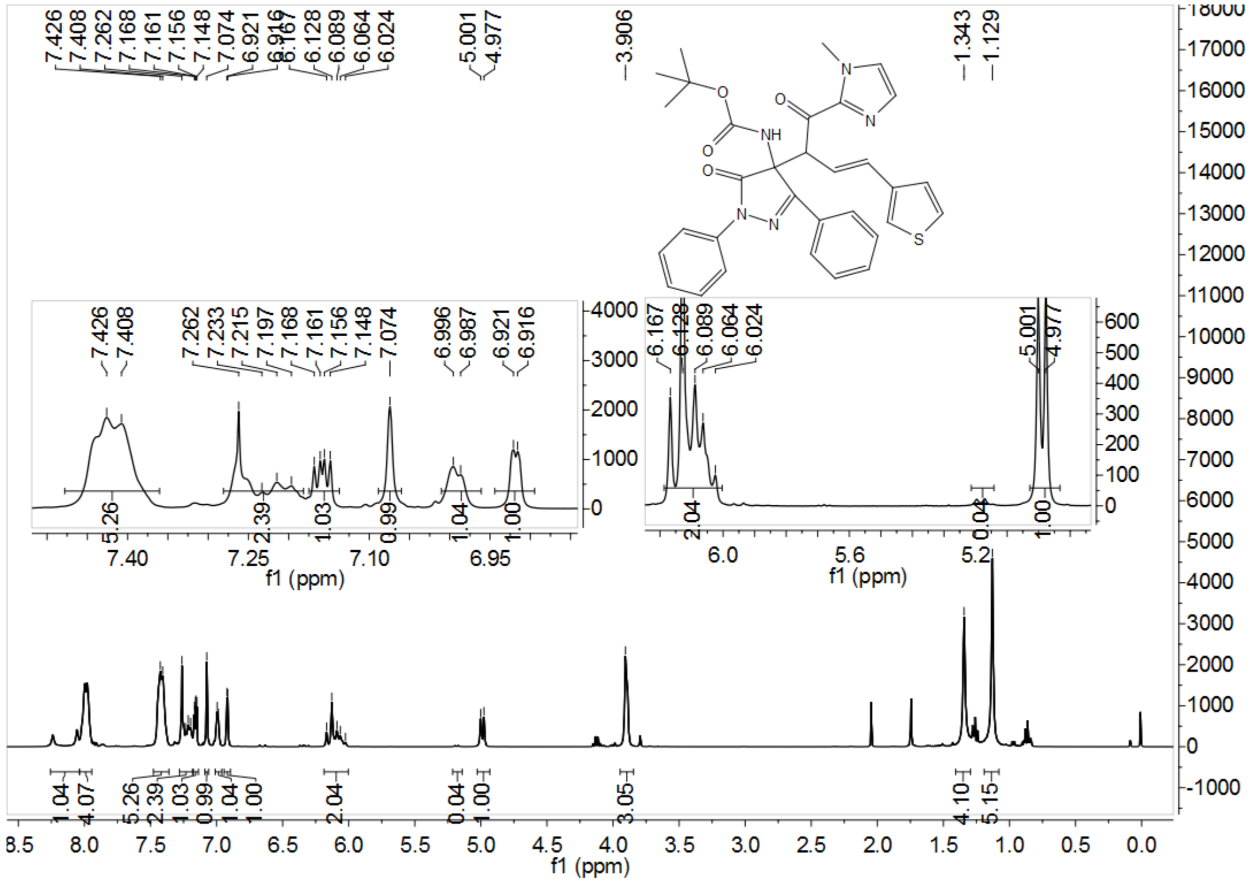

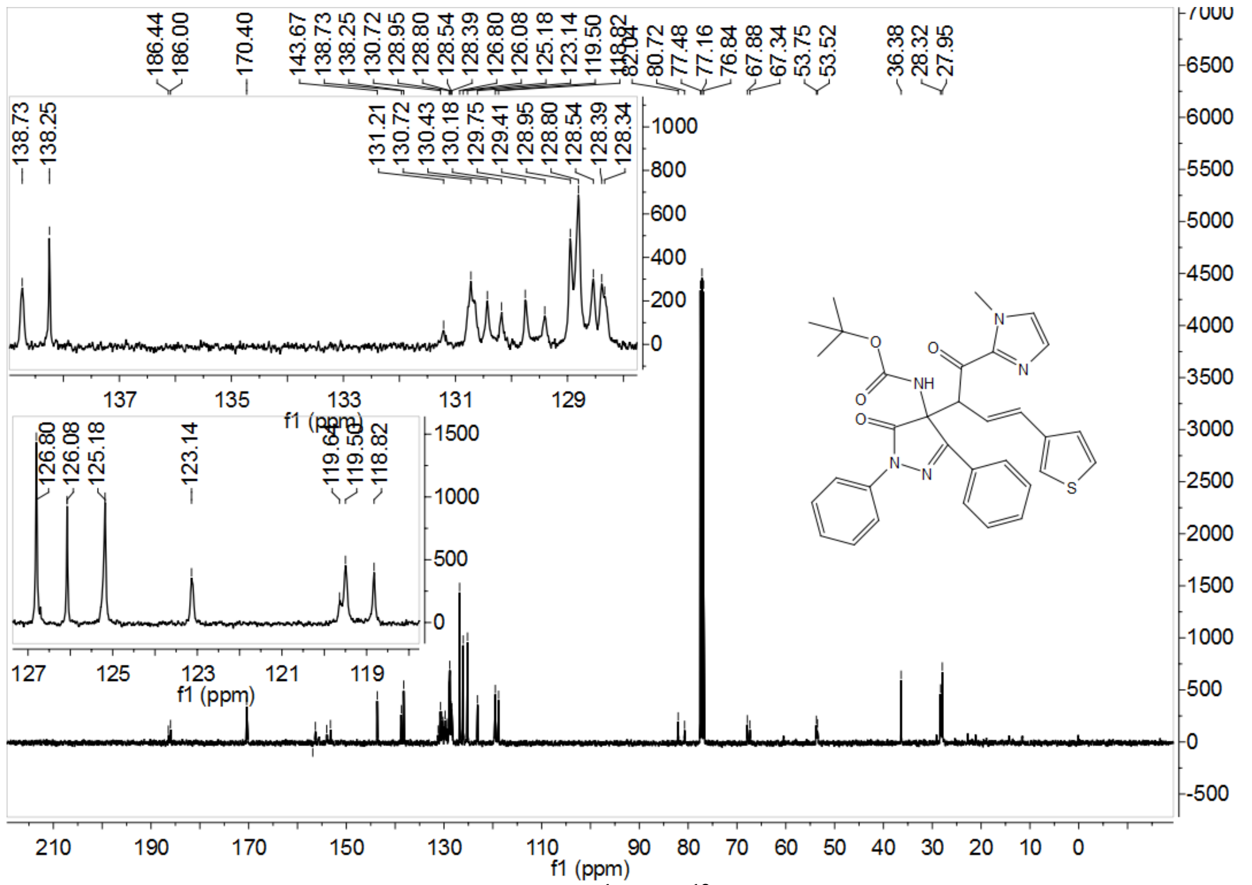

Supplementary Figure 74. ^{1}H and ^{13}C spectra for product **4n**

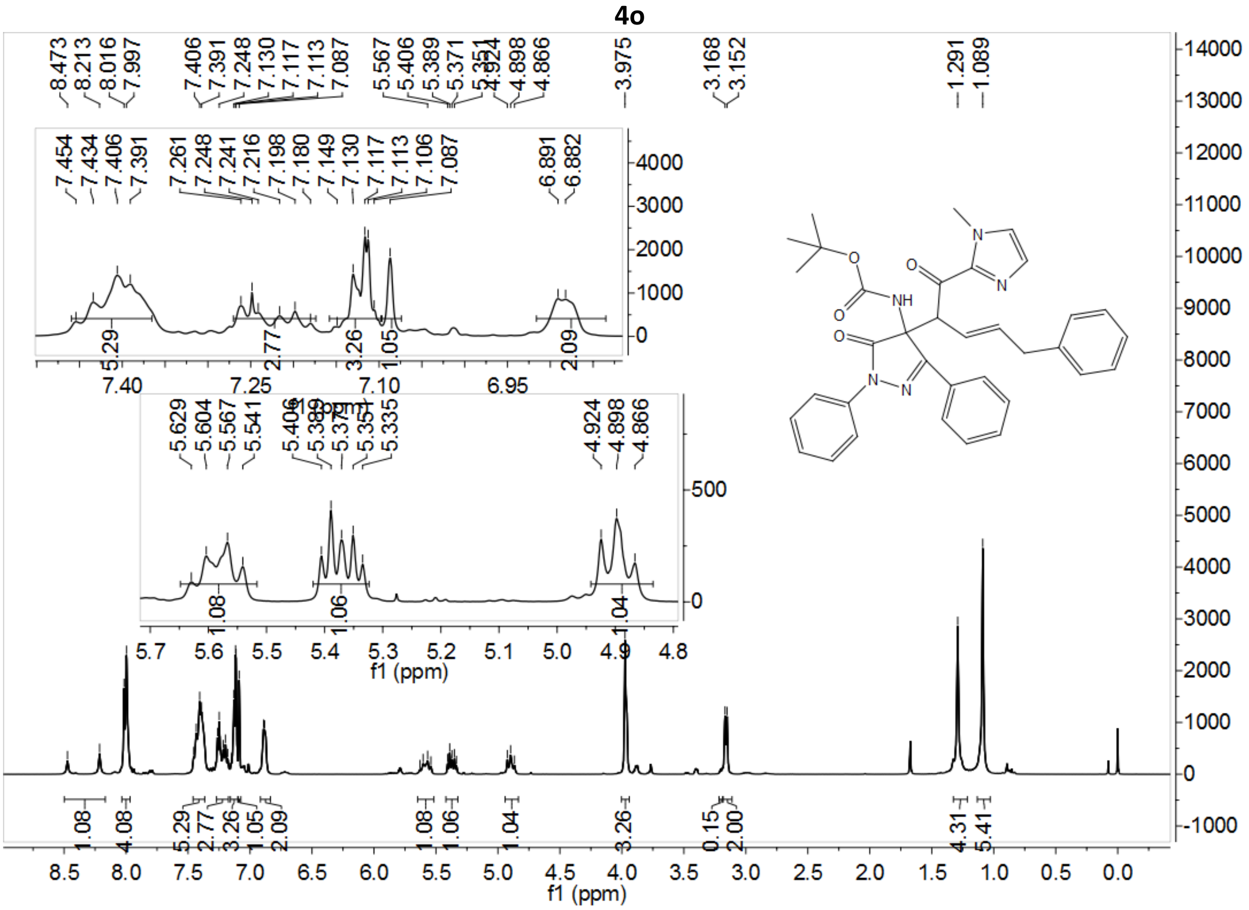

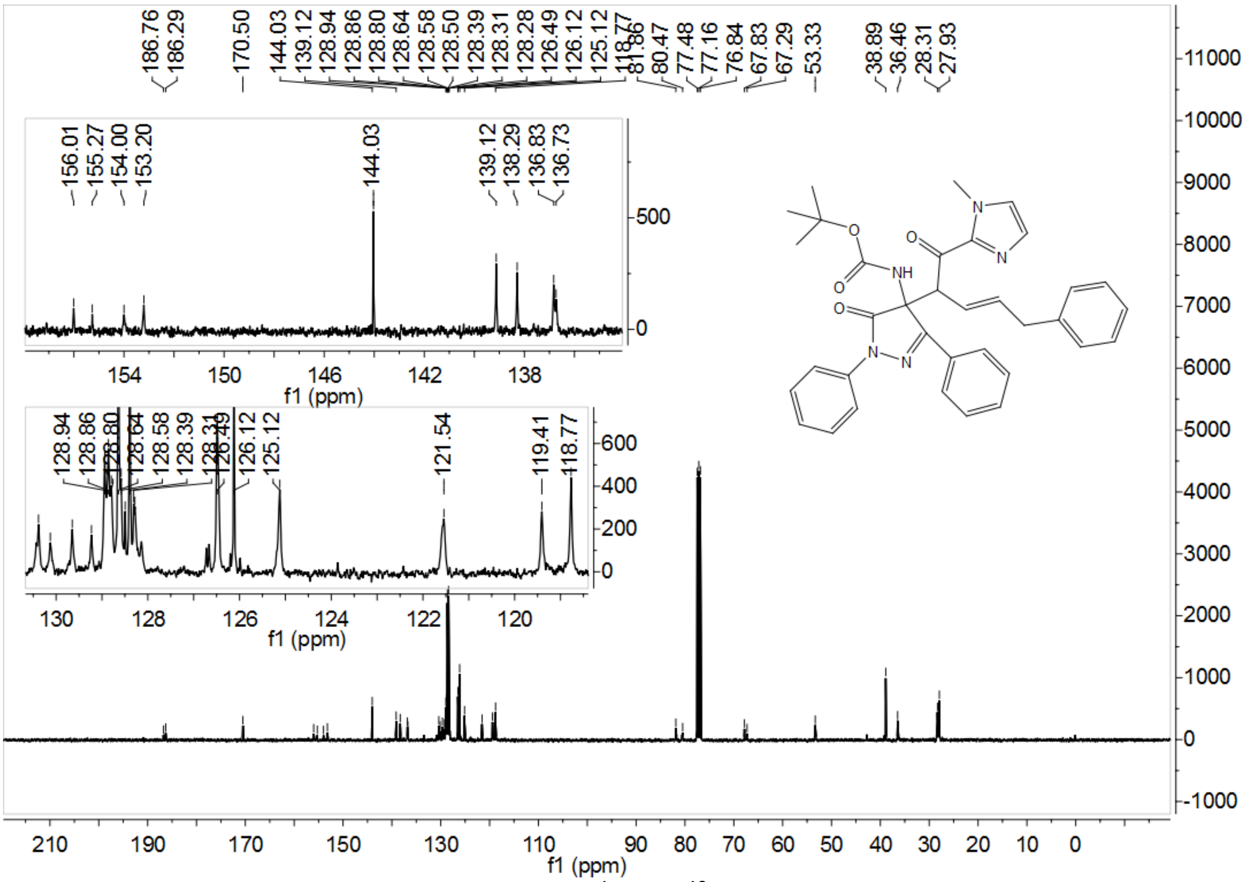

Supplementary Figure 75. ^{1}H and ^{13}C spectra for product **4o**

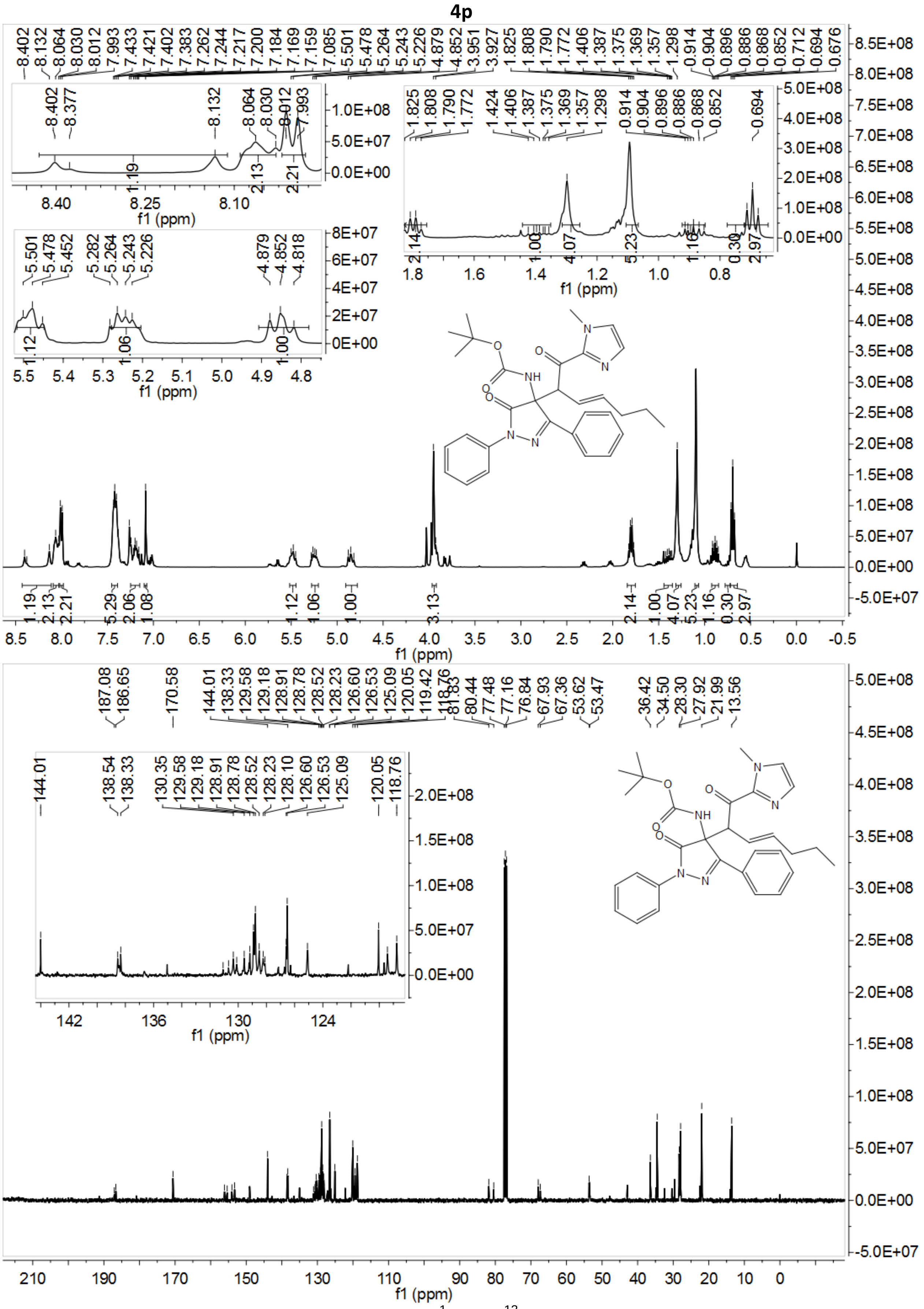

Supplementary Figure 76. ^{1}H and ^{13}C spectra for product **4p**

4q

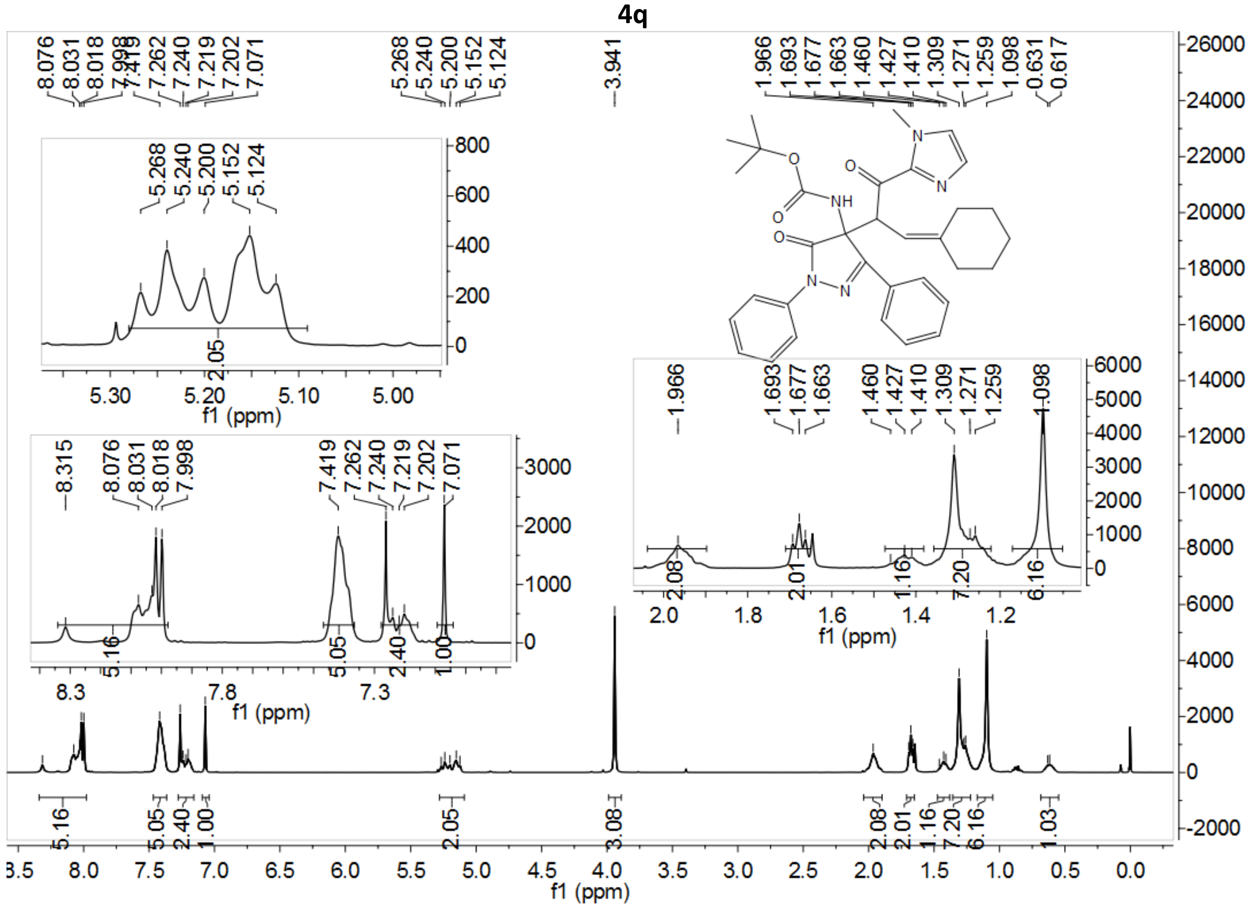

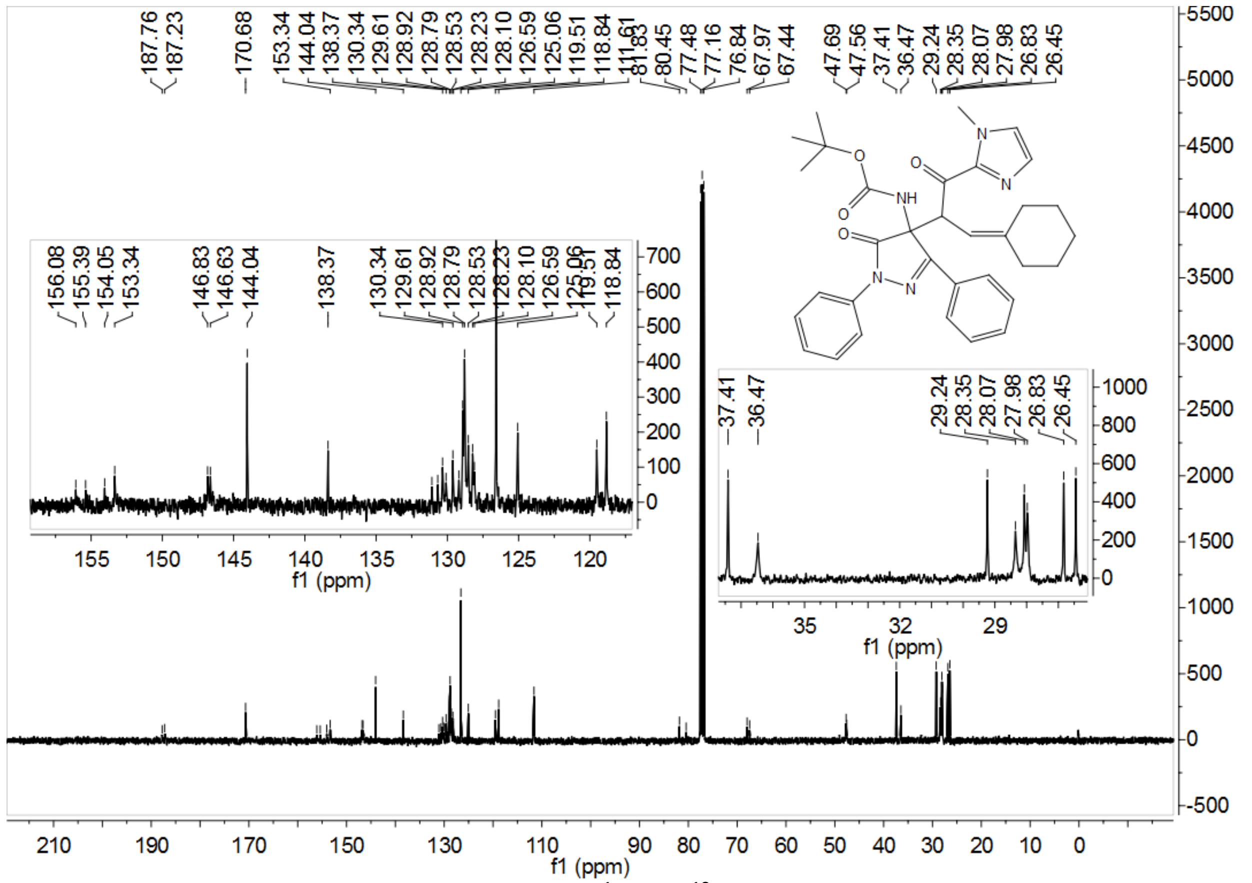

Supplementary Figure 77. ^{1}H and ^{13}C spectra for product **4q**

4r

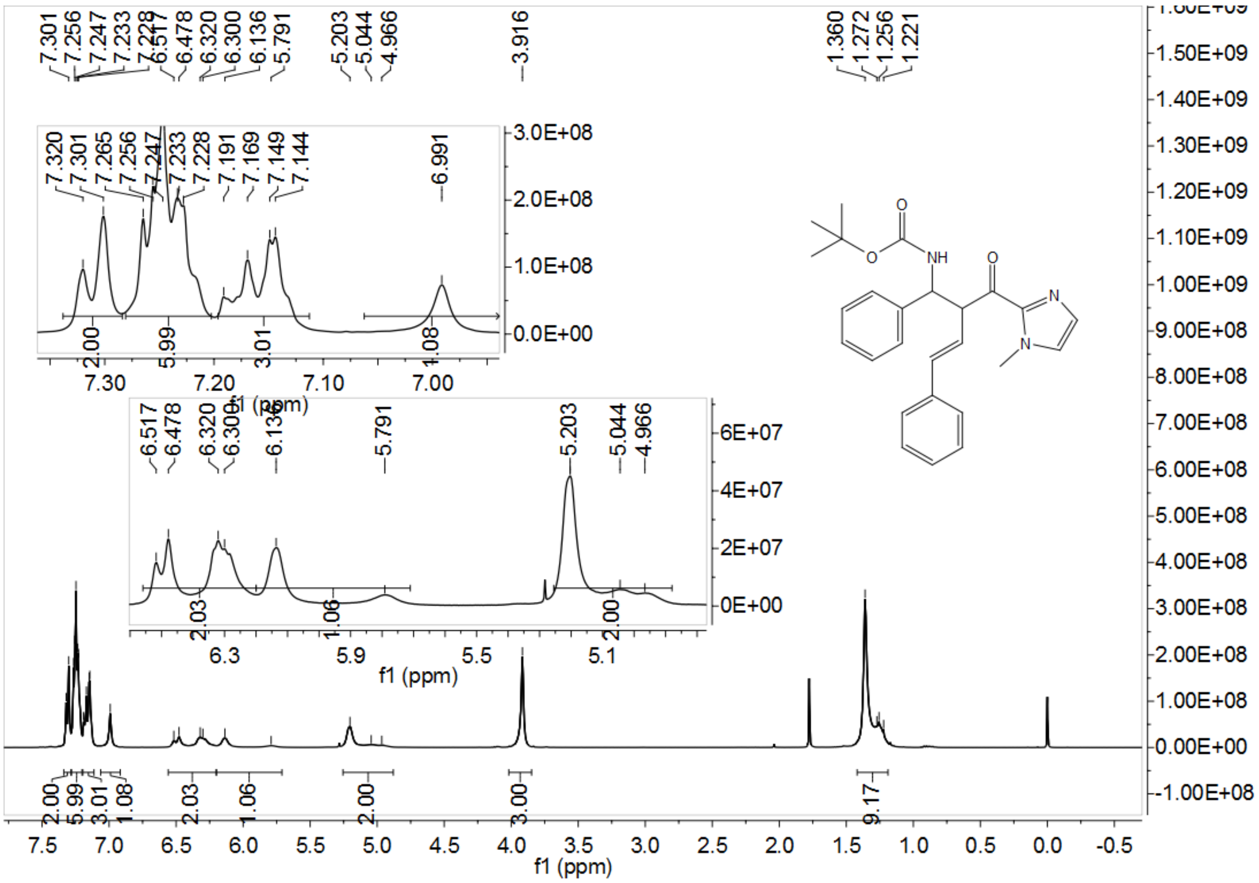

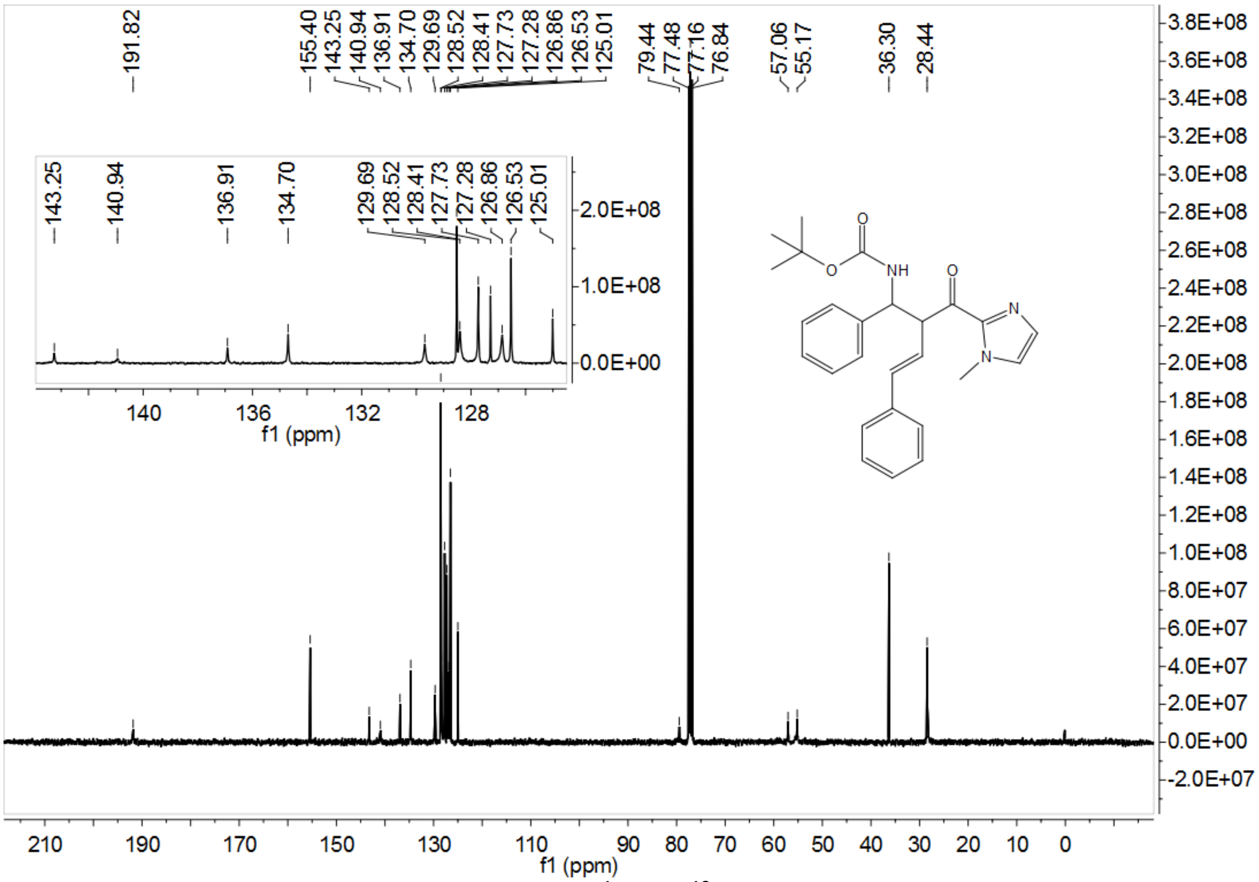

Supplementary Figure 78. ^{1}H and ^{13}C spectra for product **4r**

4s

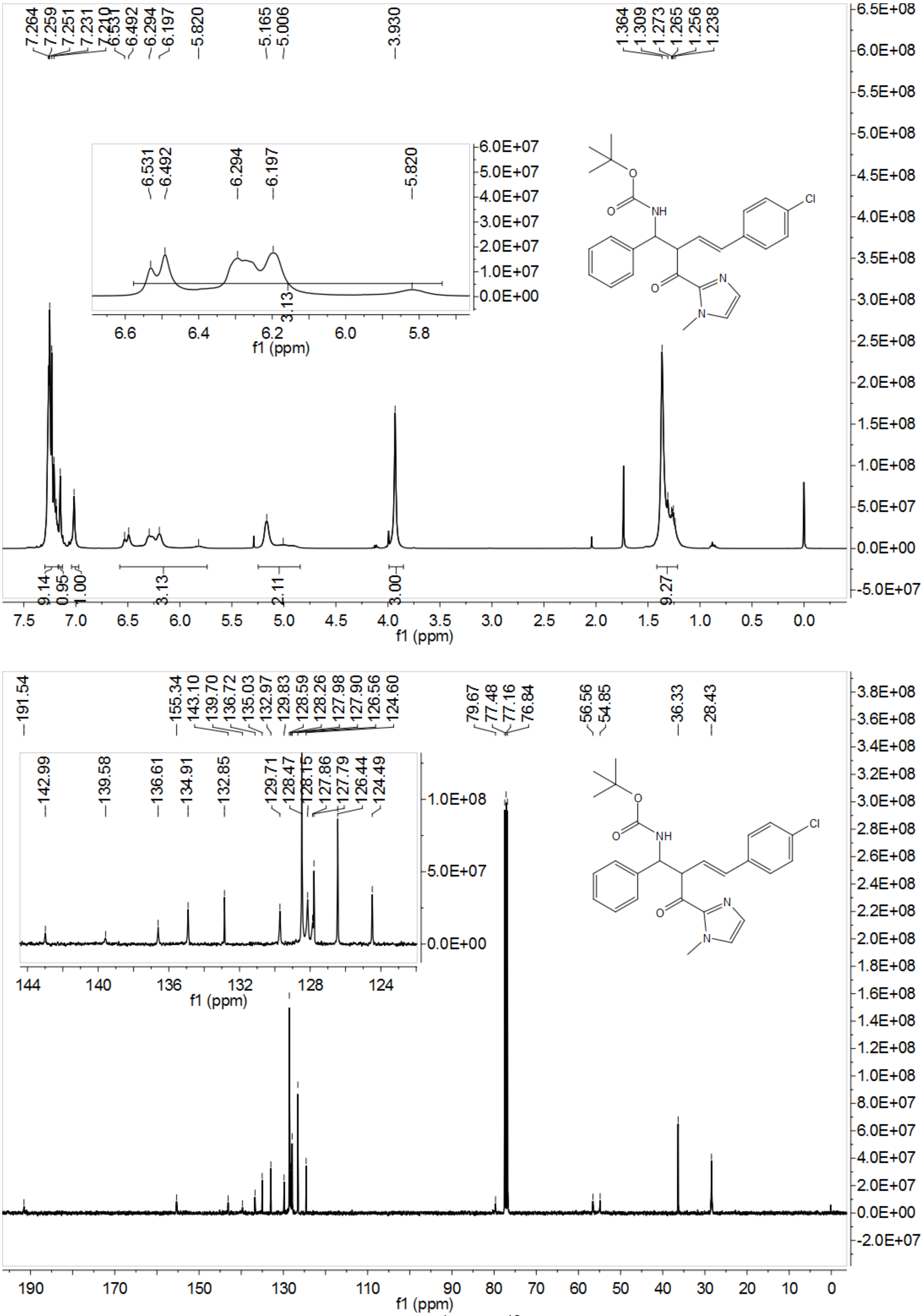

Supplementary Figure 79. ^{1}H and ^{13}C spectra for product **4s**

4t

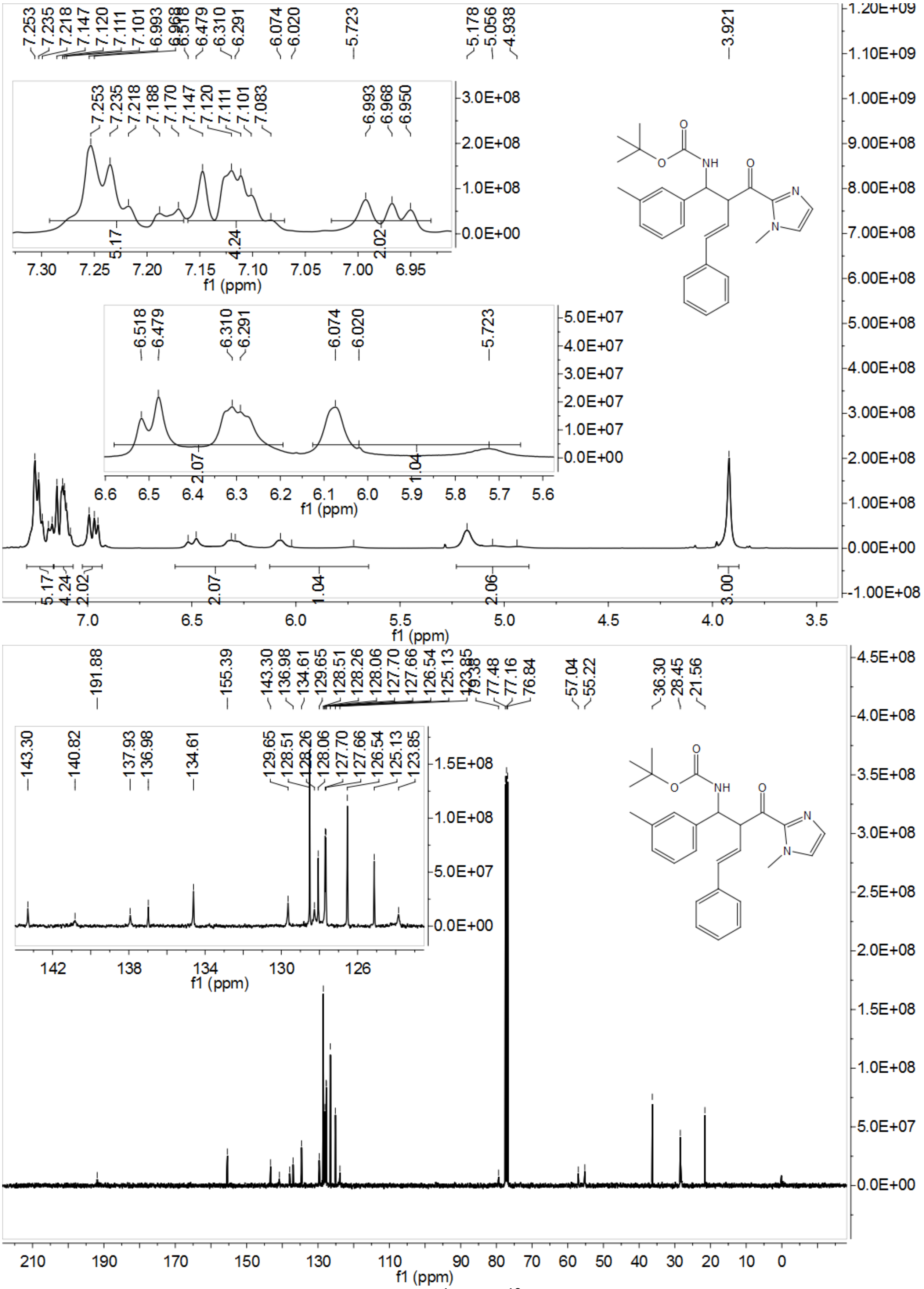

Supplementary Figure 80. ^{1}H and ^{13}C spectra for product **4t**

4u

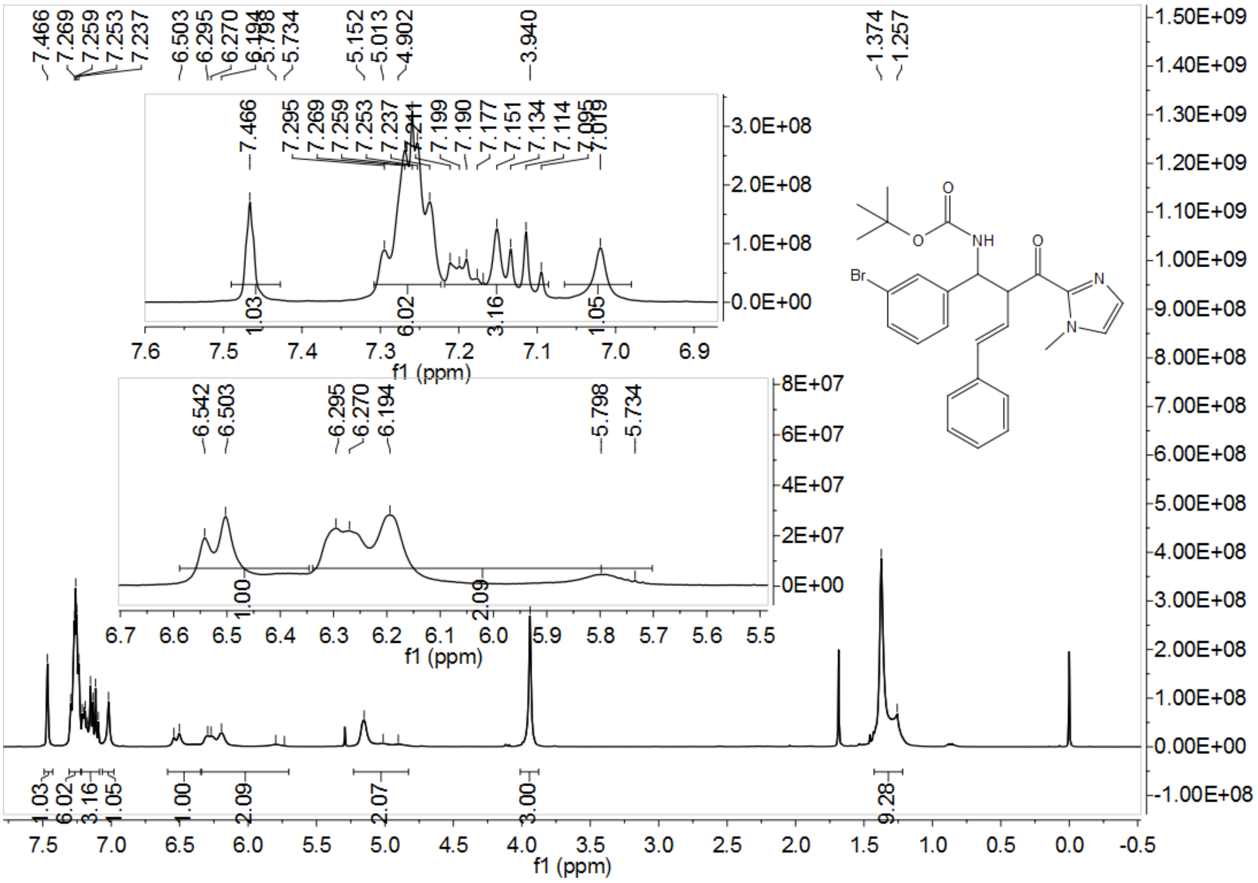

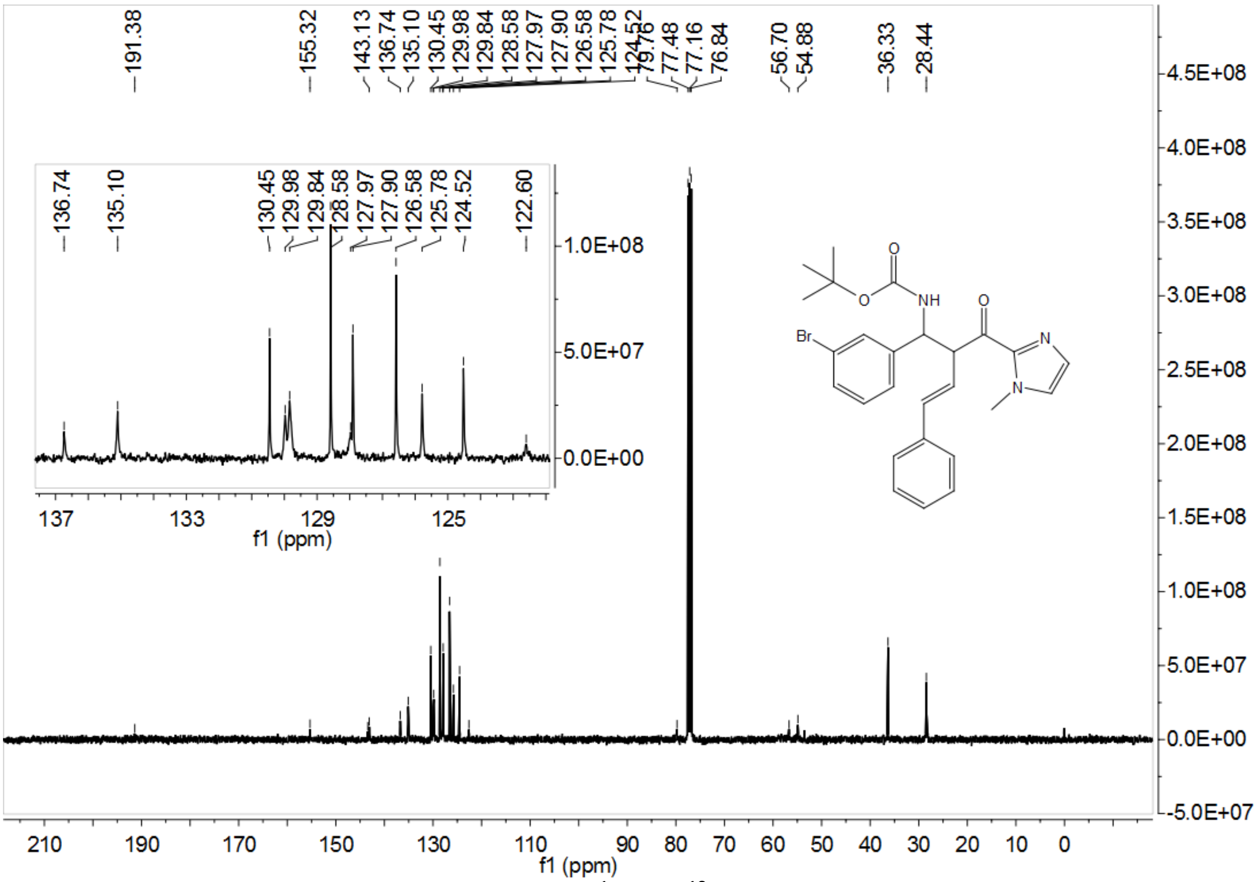

Supplementary Figure 81. ^{1}H and ^{13}C spectra for product **4u**

4v

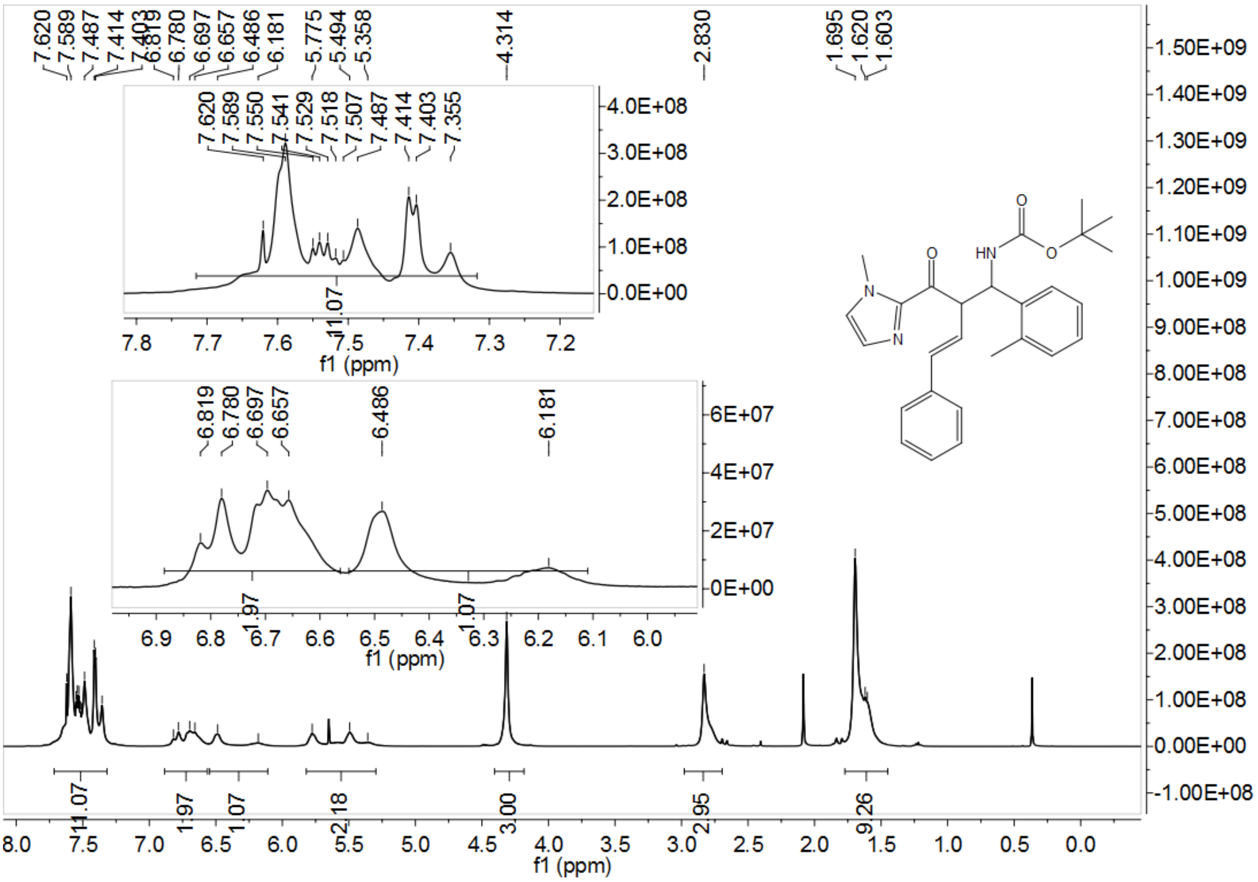

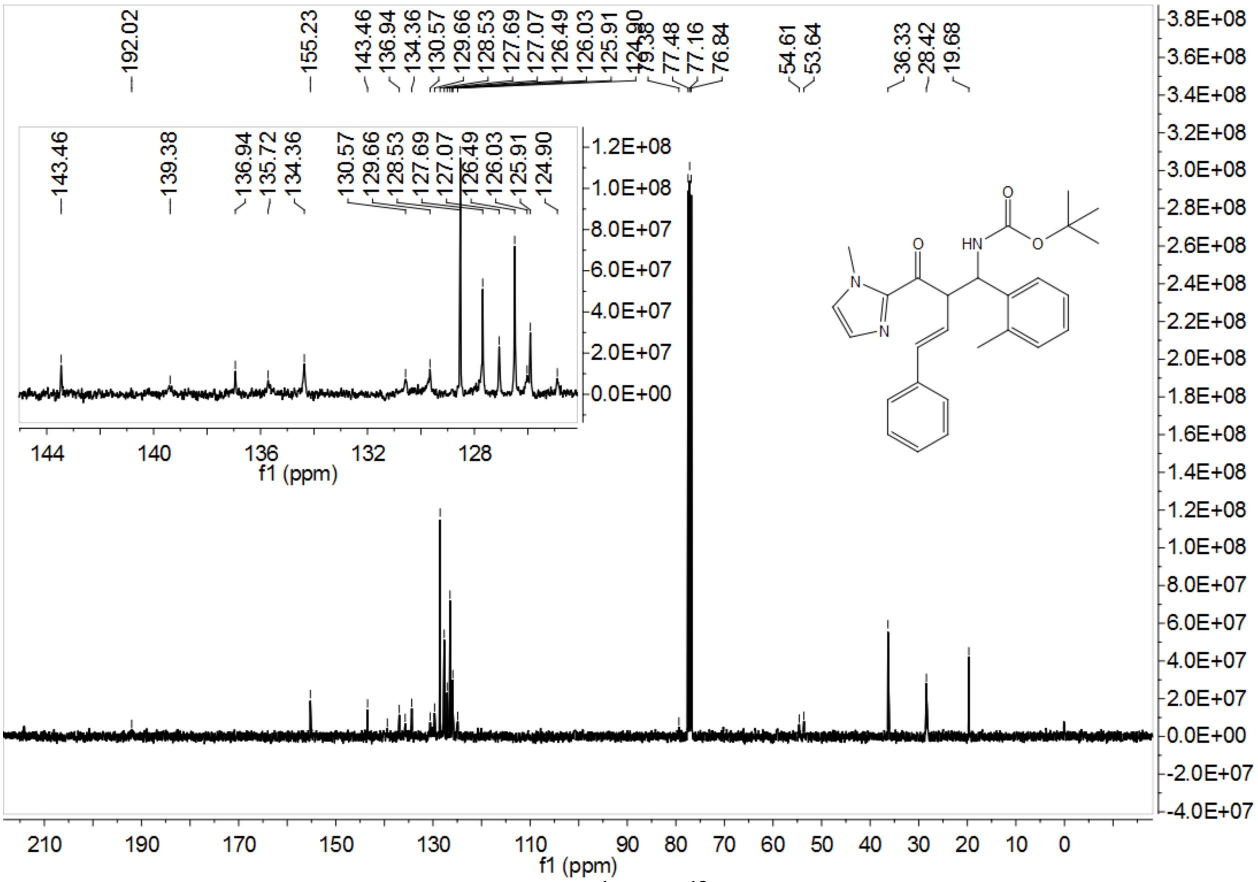

Supplementary Figure 82. ^{1}H and ^{13}C spectra for product **4v**

4w

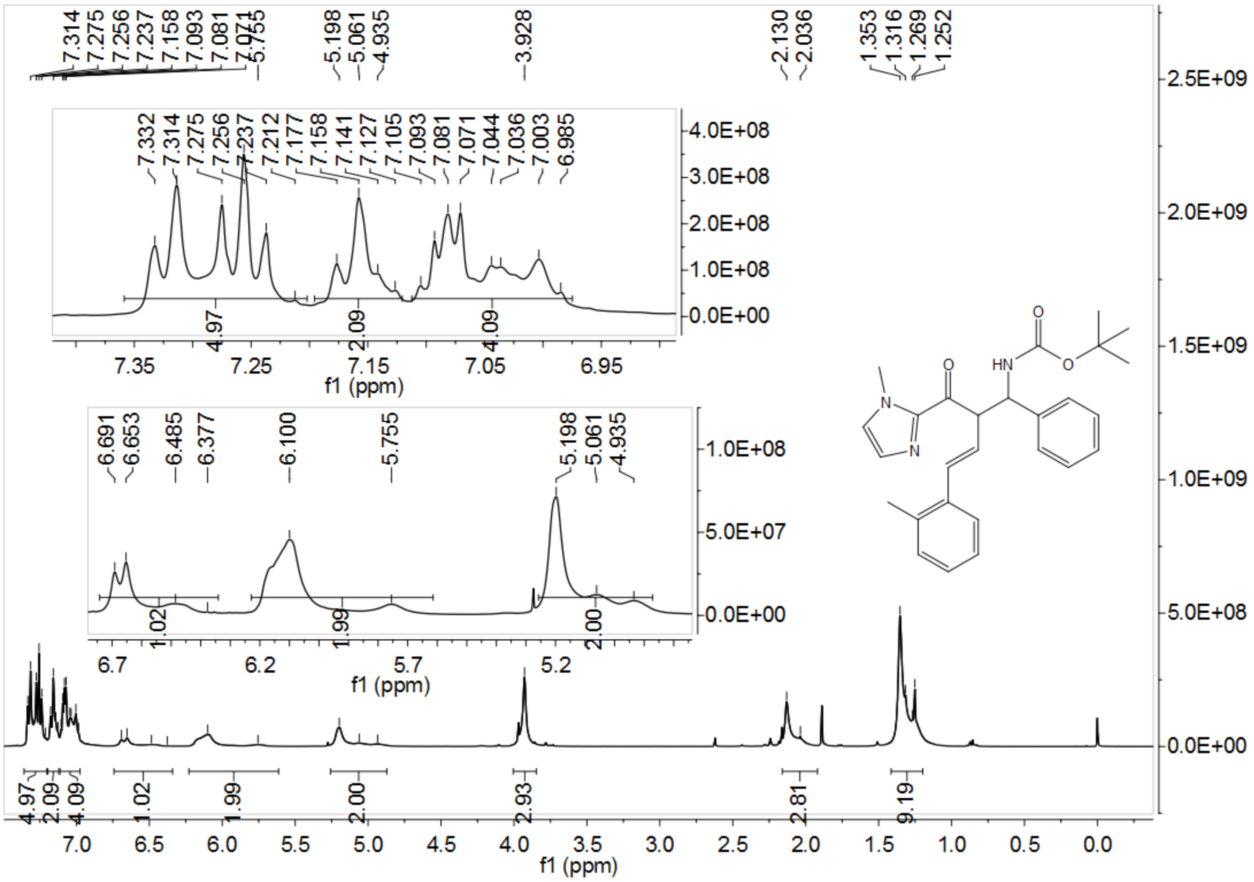

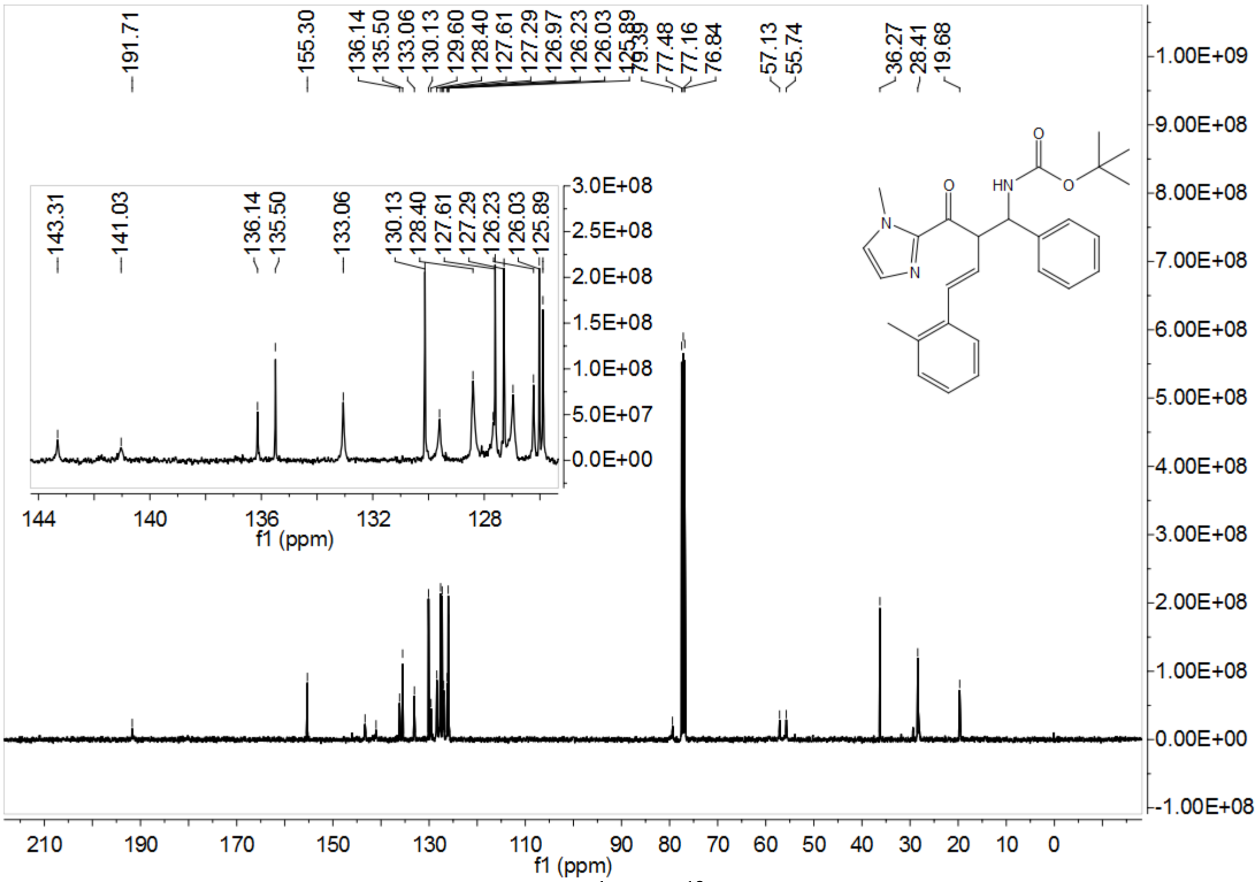

Supplementary Figure 83. ^{1}H and ^{13}C spectra for product **4w**

4x

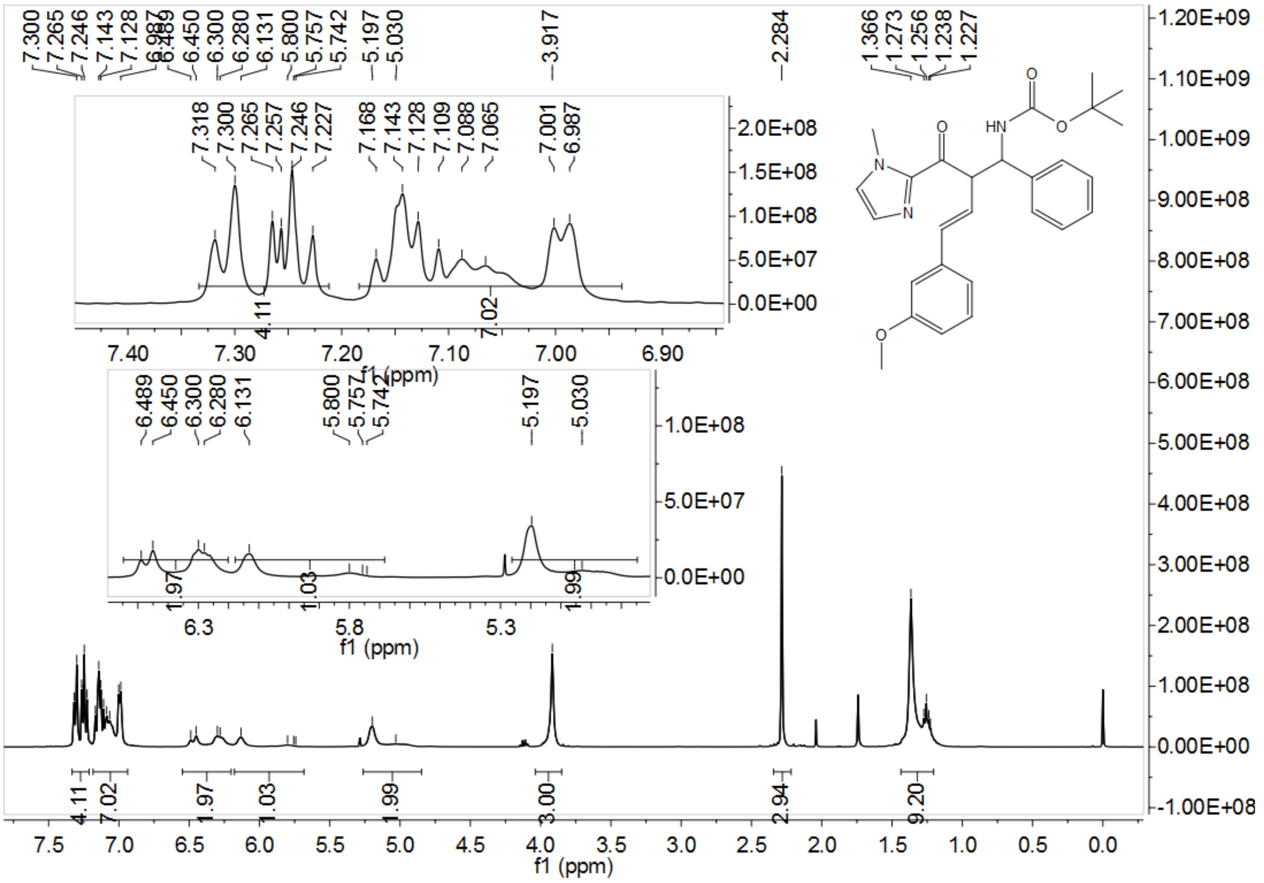

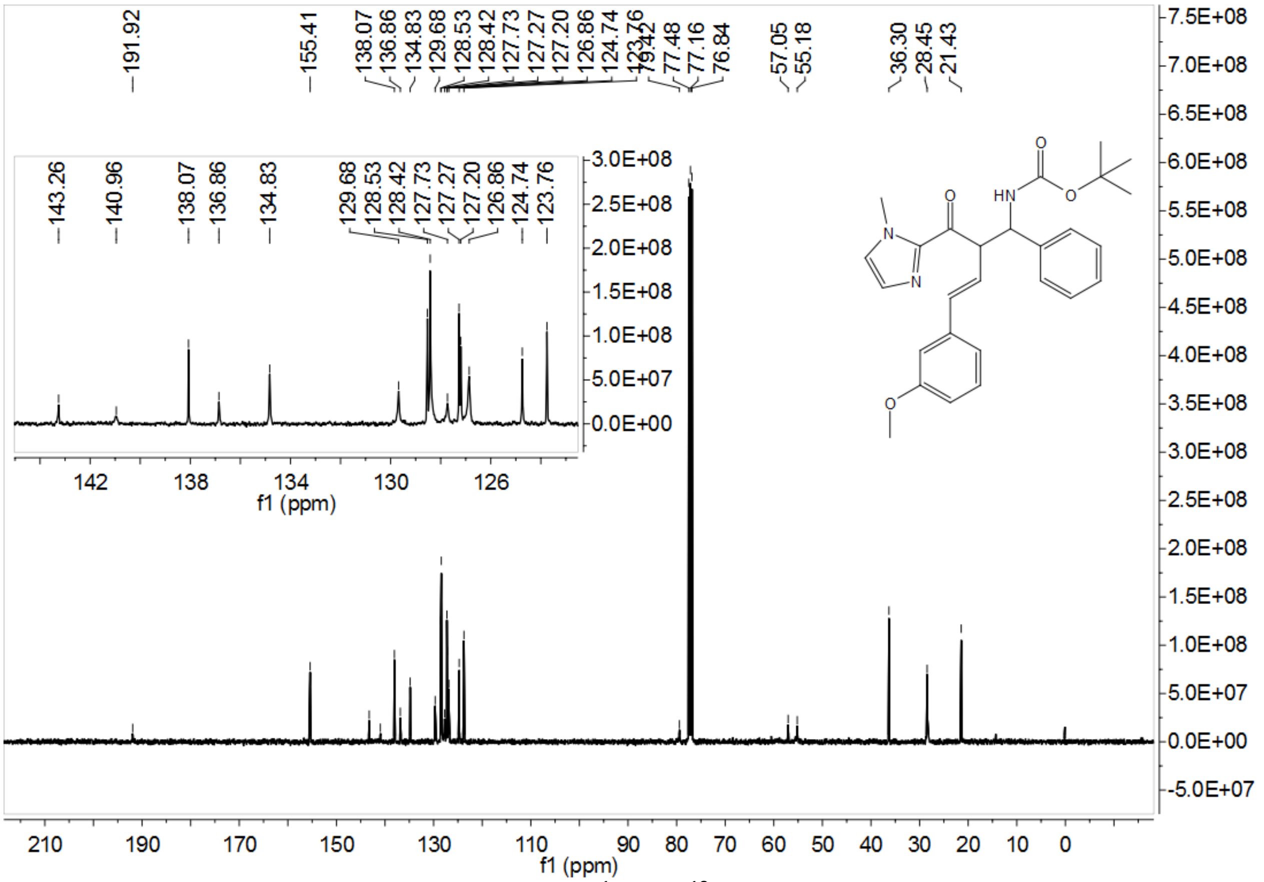

Supplementary Figure 84. ^{1}H and ^{13}C spectra for product **4x**

4y

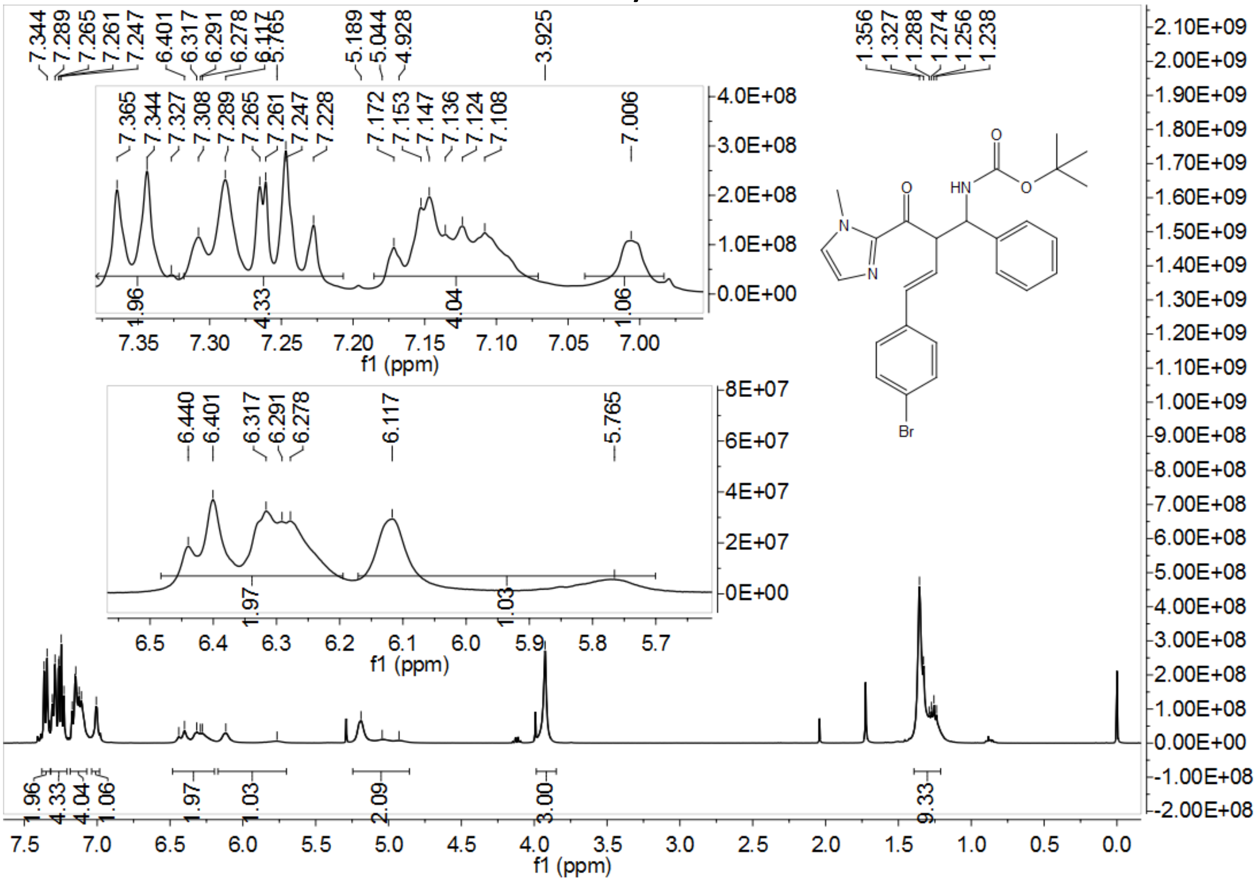

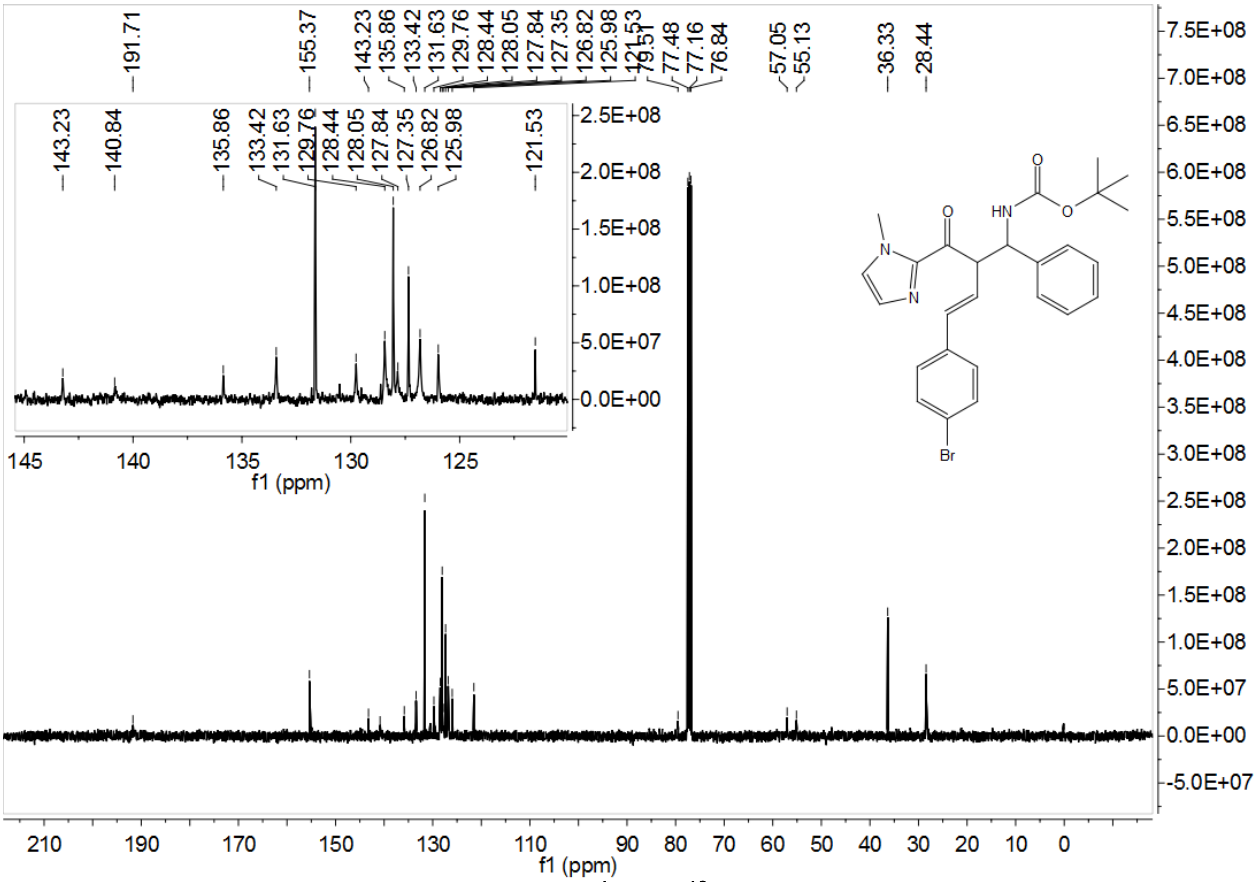

Supplementary Figure 85. ^{1}H and ^{13}C spectra for product **4y**

4z

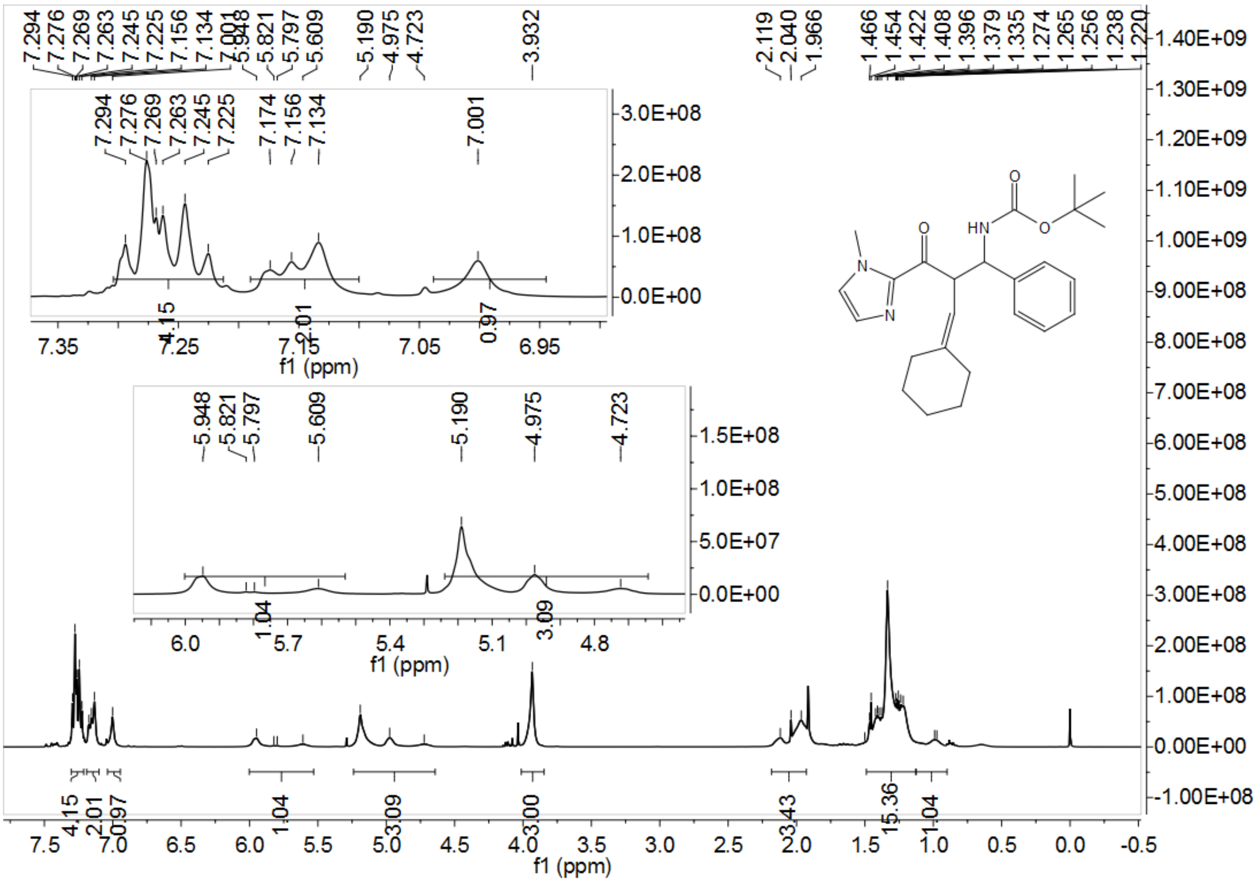

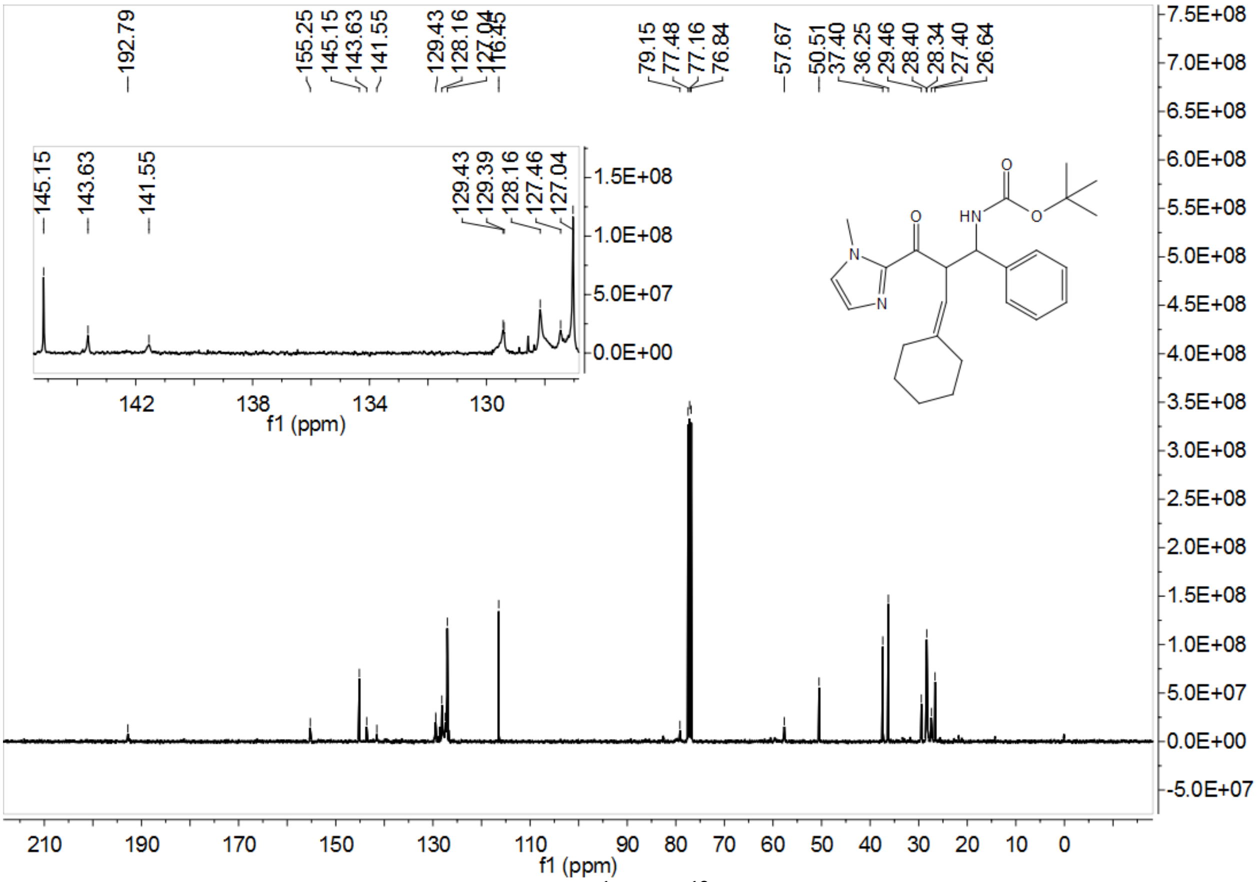

Supplementary Figure 86. ^{1}H and ^{13}C spectra for product **4z**

6a

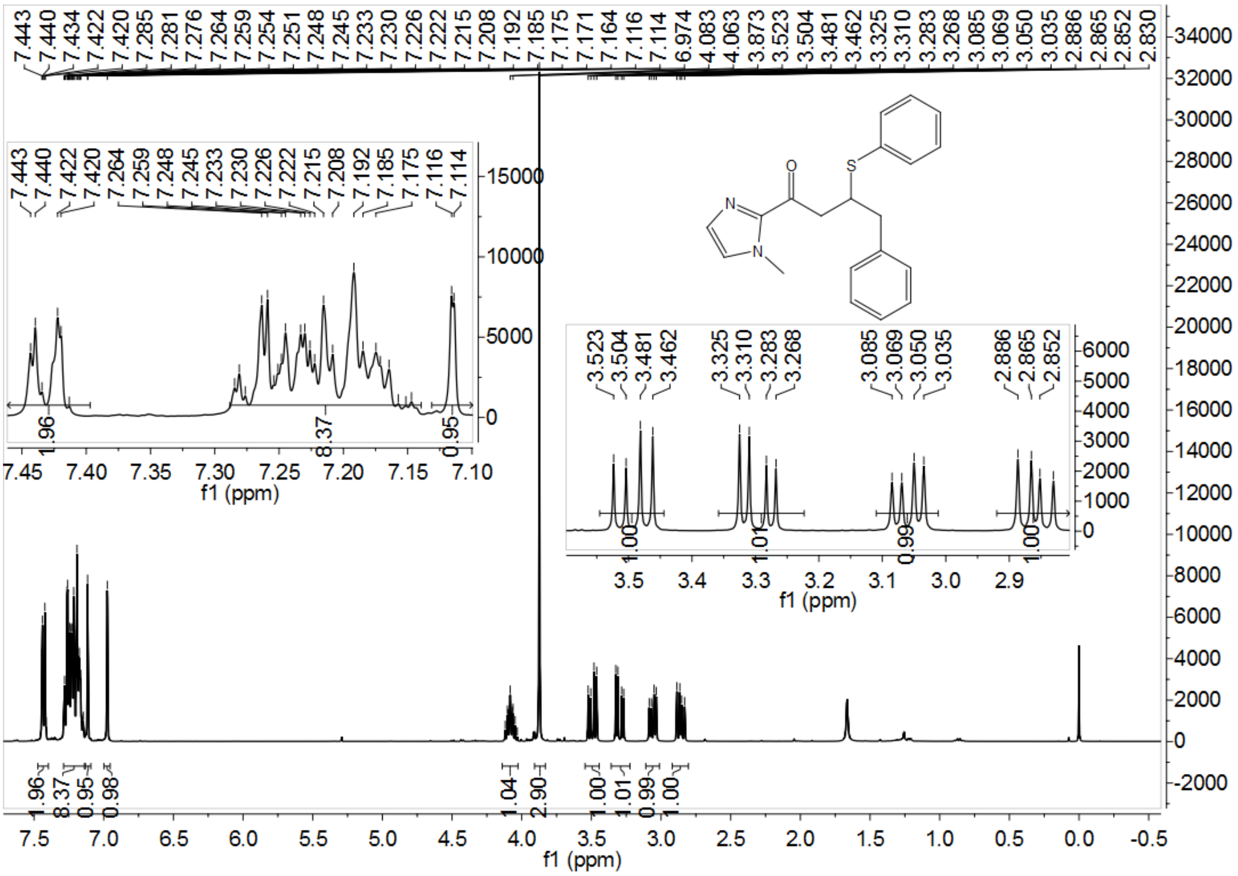

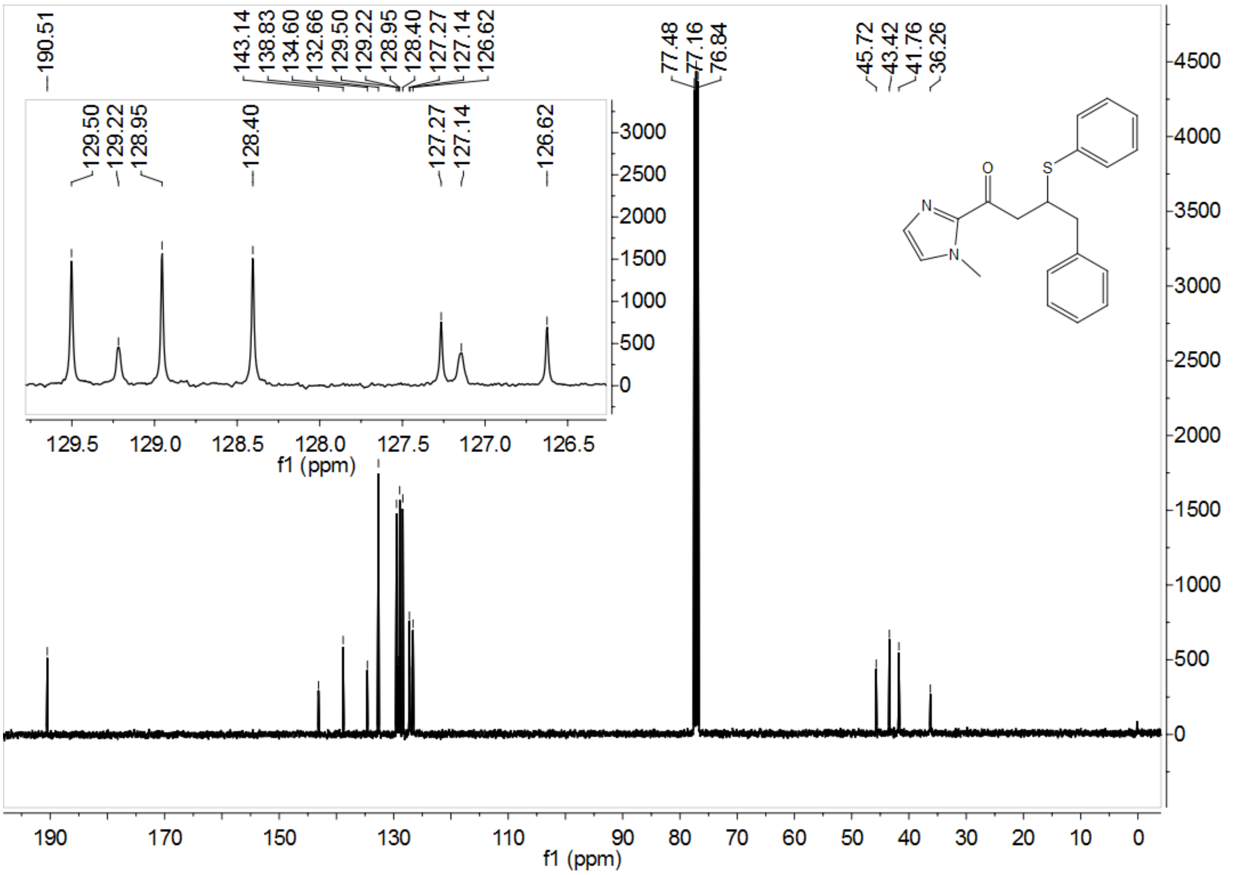

Supplementary Figure 87. ^{1}H and ^{13}C spectra for product **6a**

6b

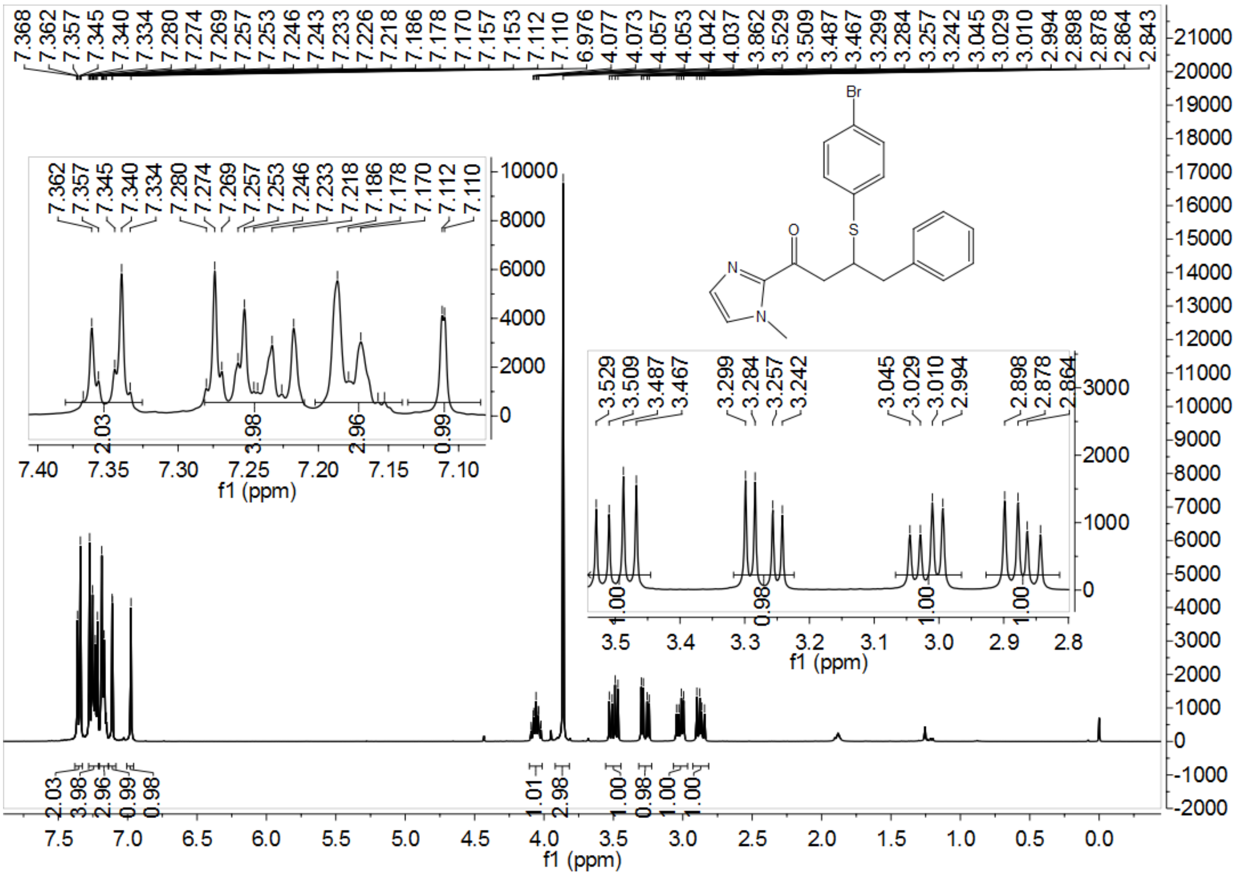

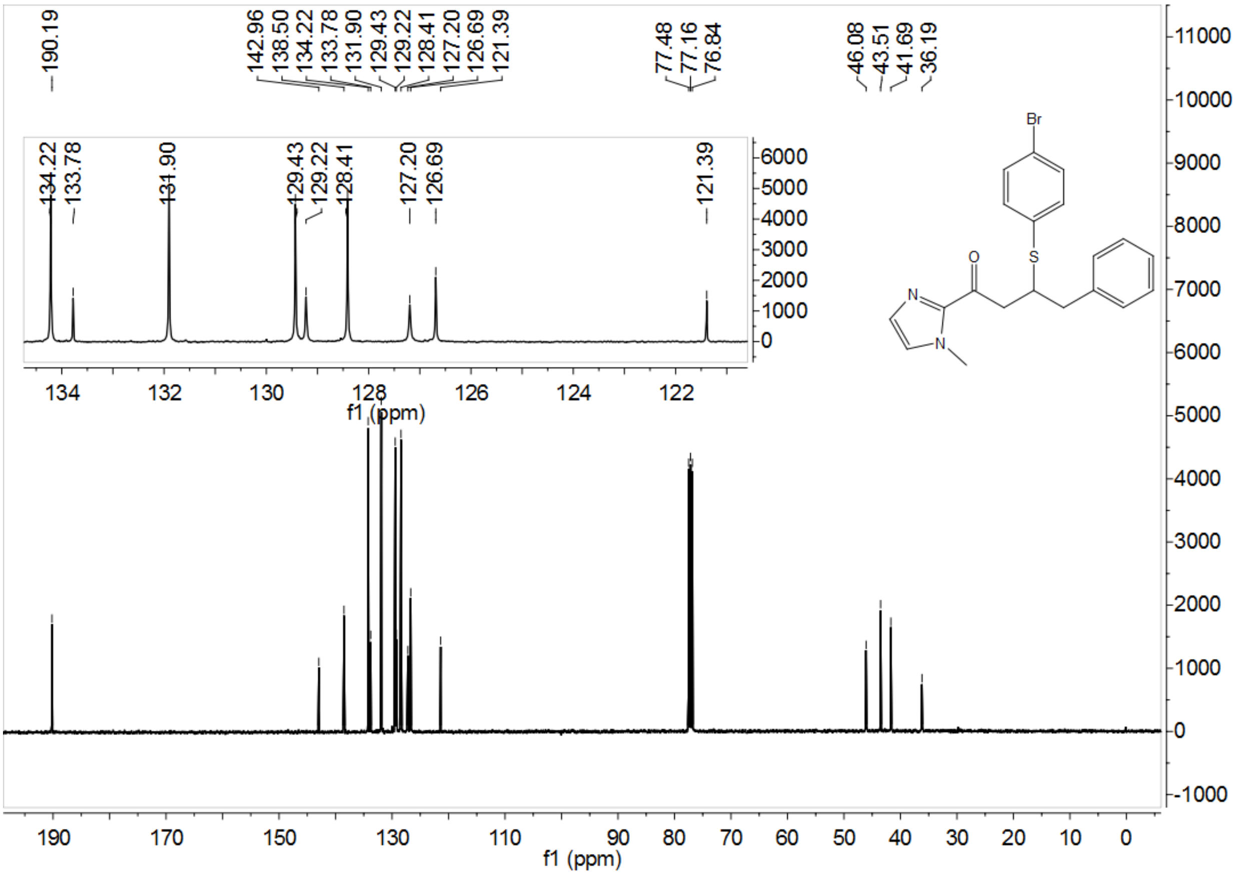

Supplementary Figure 88. ^{1}H and ^{13}C spectra for product **6b**

6c

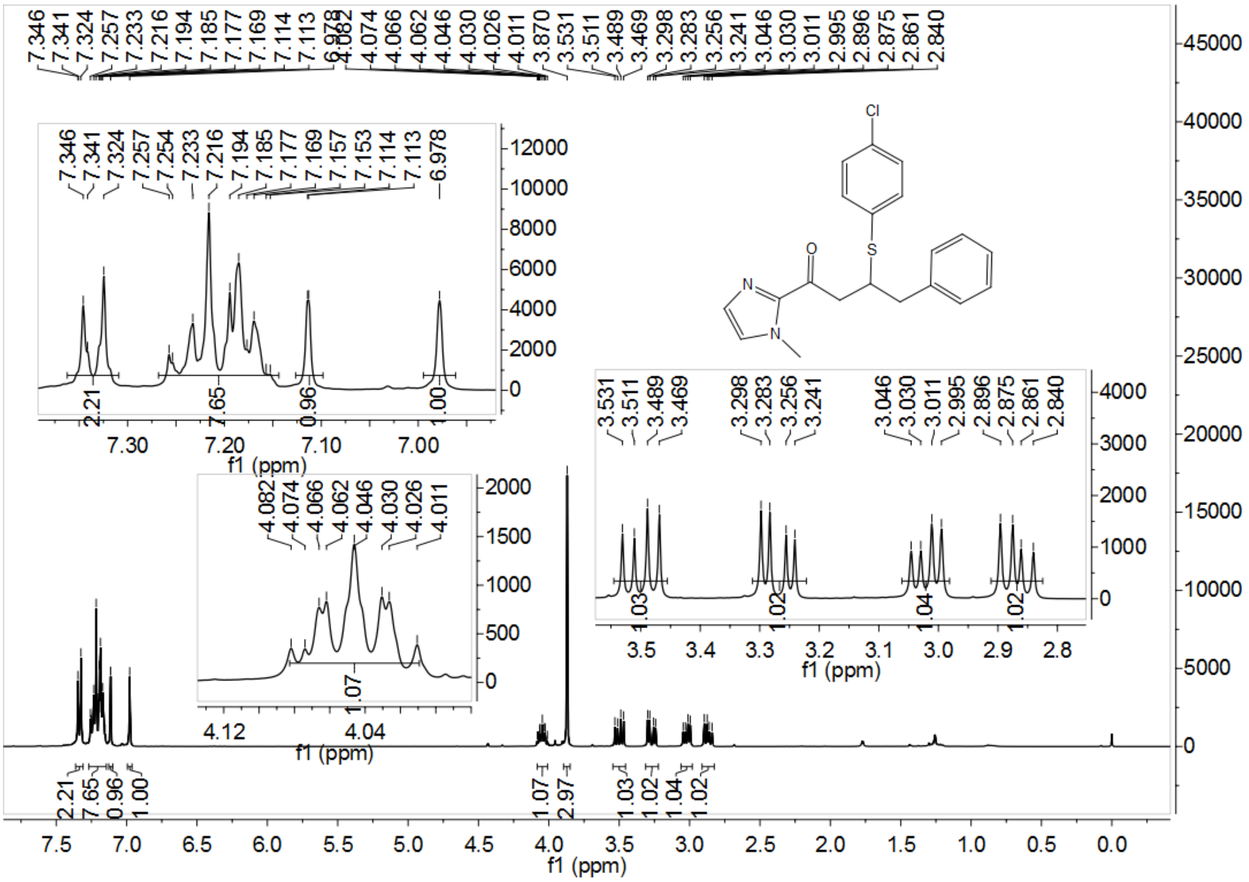

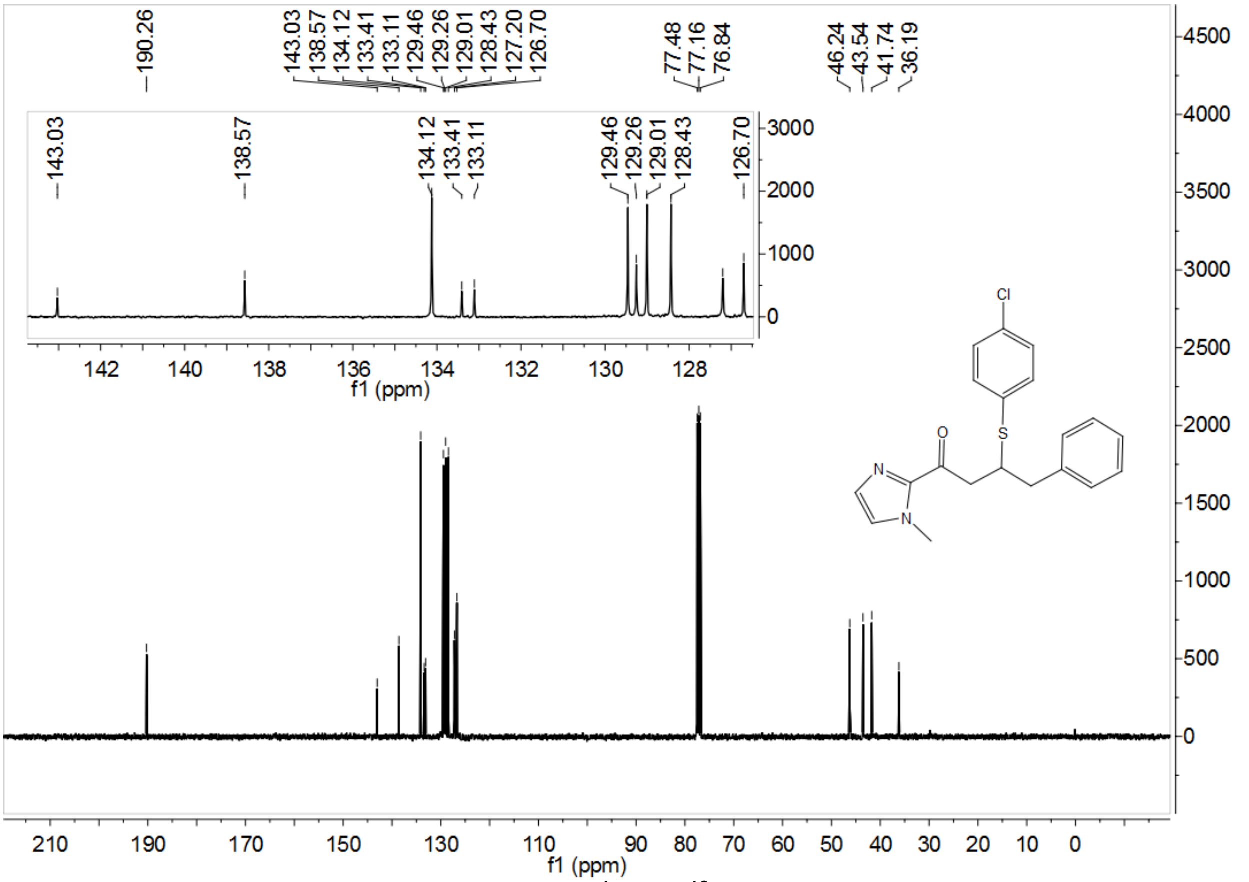

Supplementary Figure 89. ^{1}H and ^{13}C spectra for product **6c**

6d

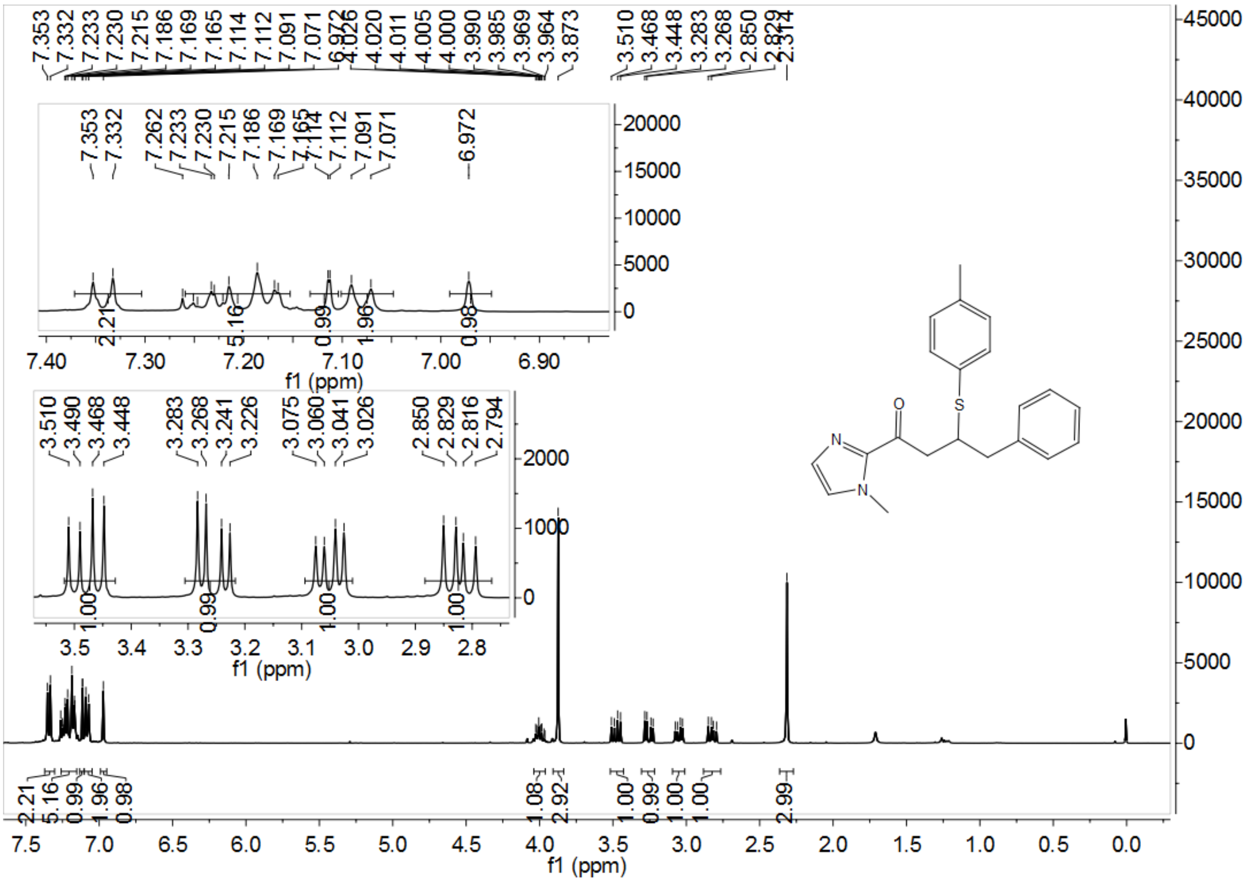

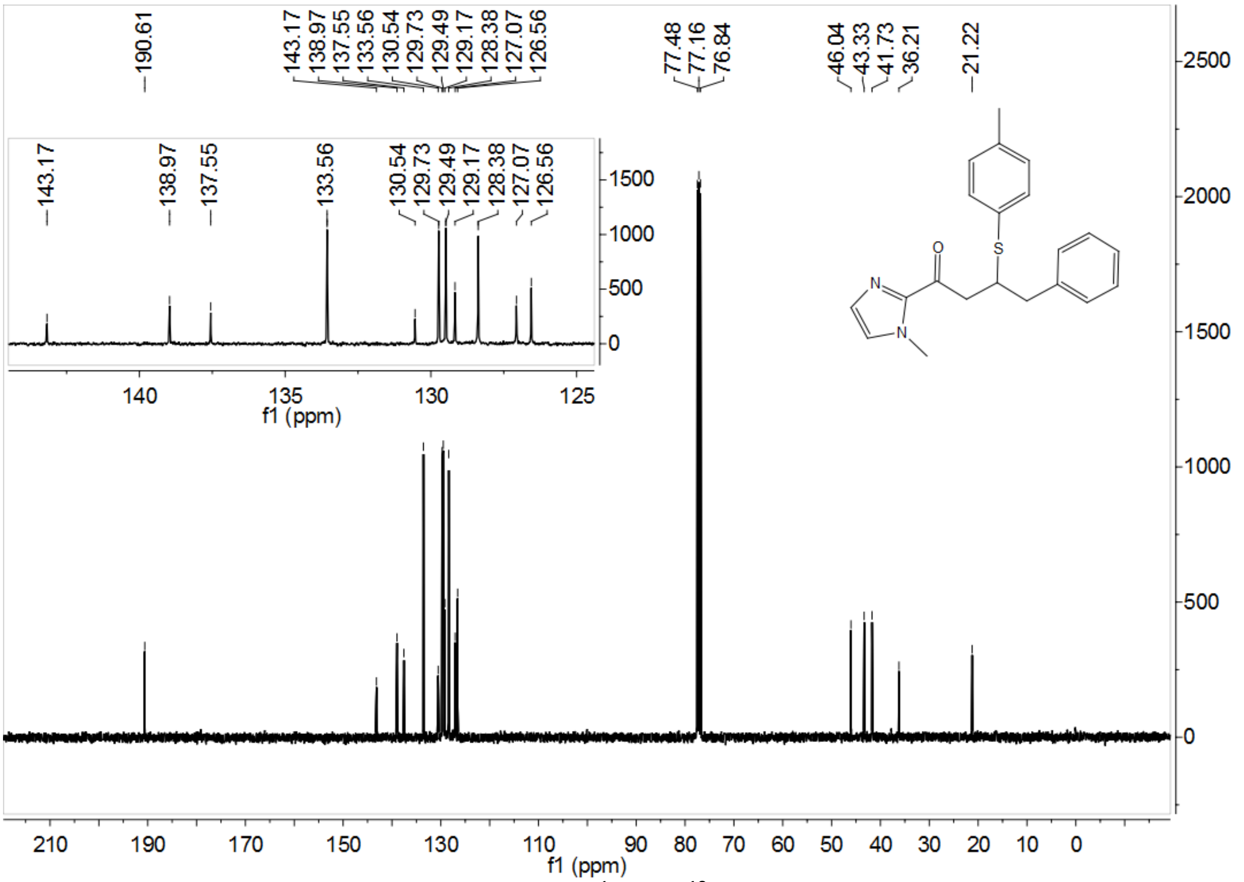

Supplementary Figure 90. ^{1}H and ^{13}C spectra for product **6d**

6e

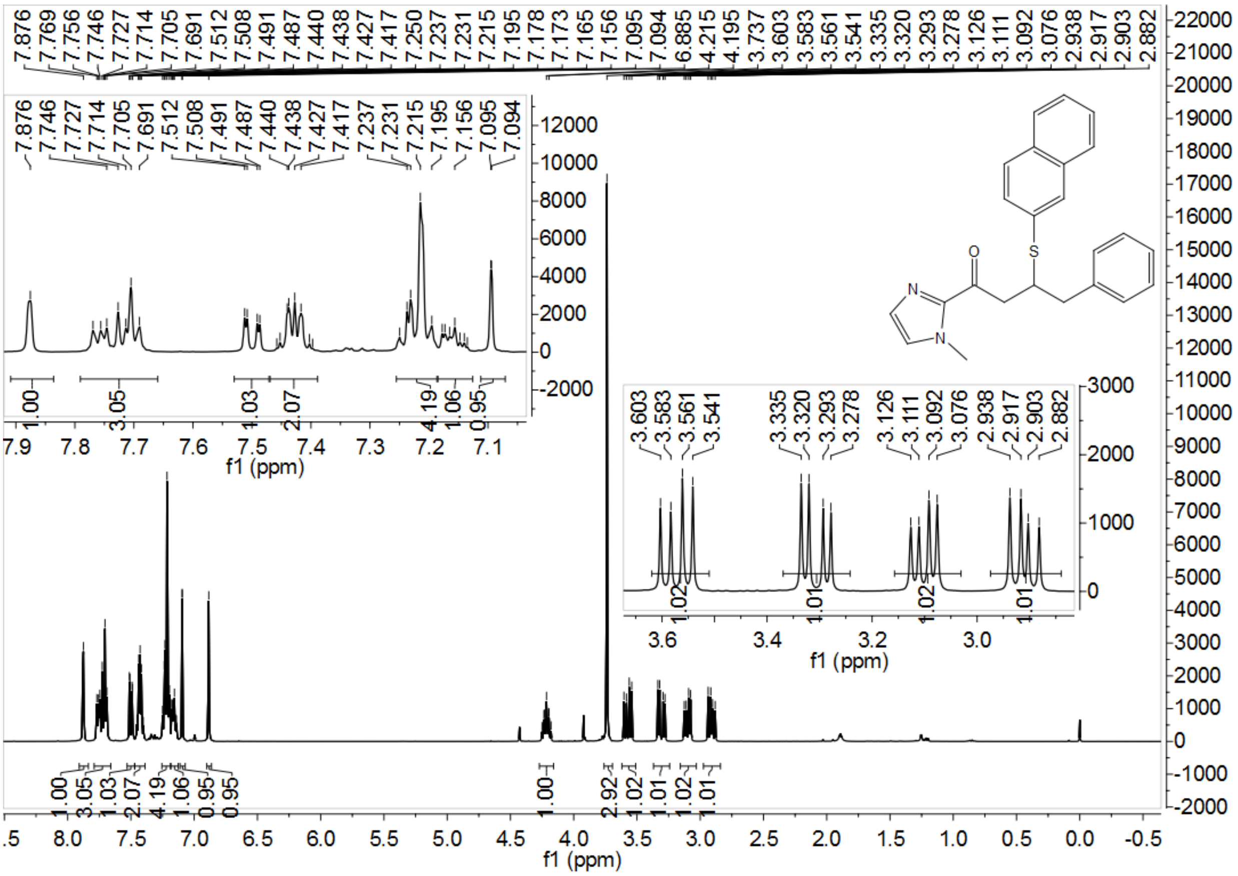

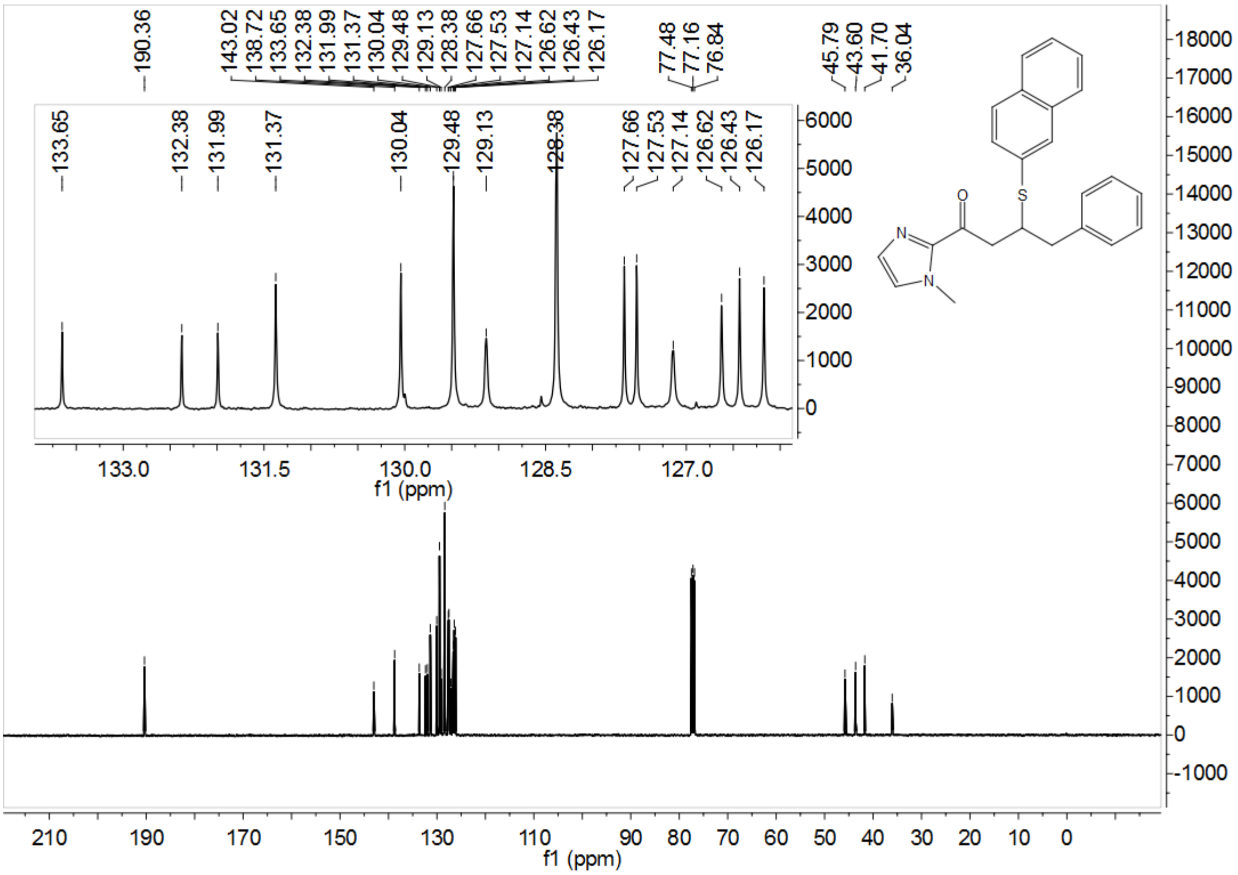

Supplementary Figure 91. ^{1}H and ^{13}C spectra for product **6e**

6f

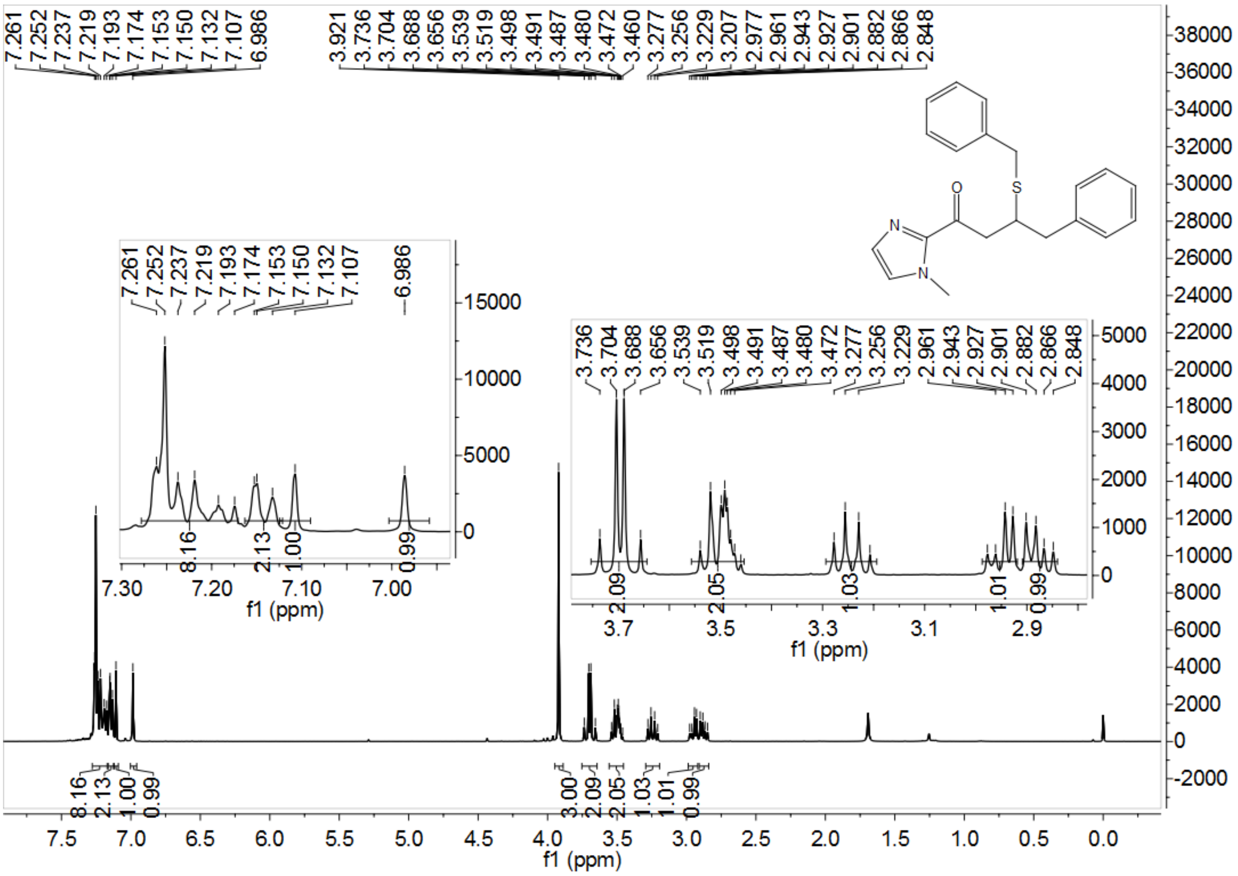

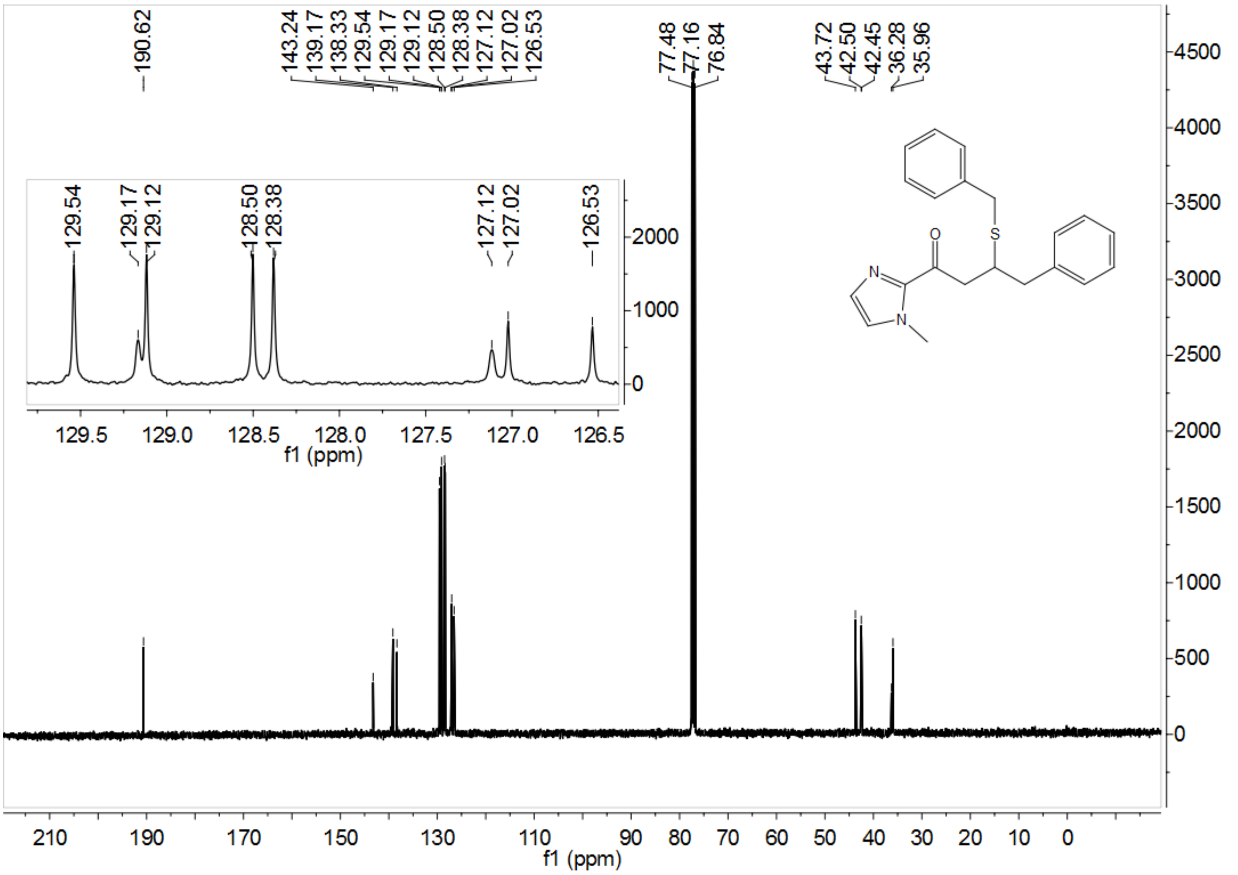

Supplementary Figure 92. ^{1}H and ^{13}C spectra for product **6f**

6g

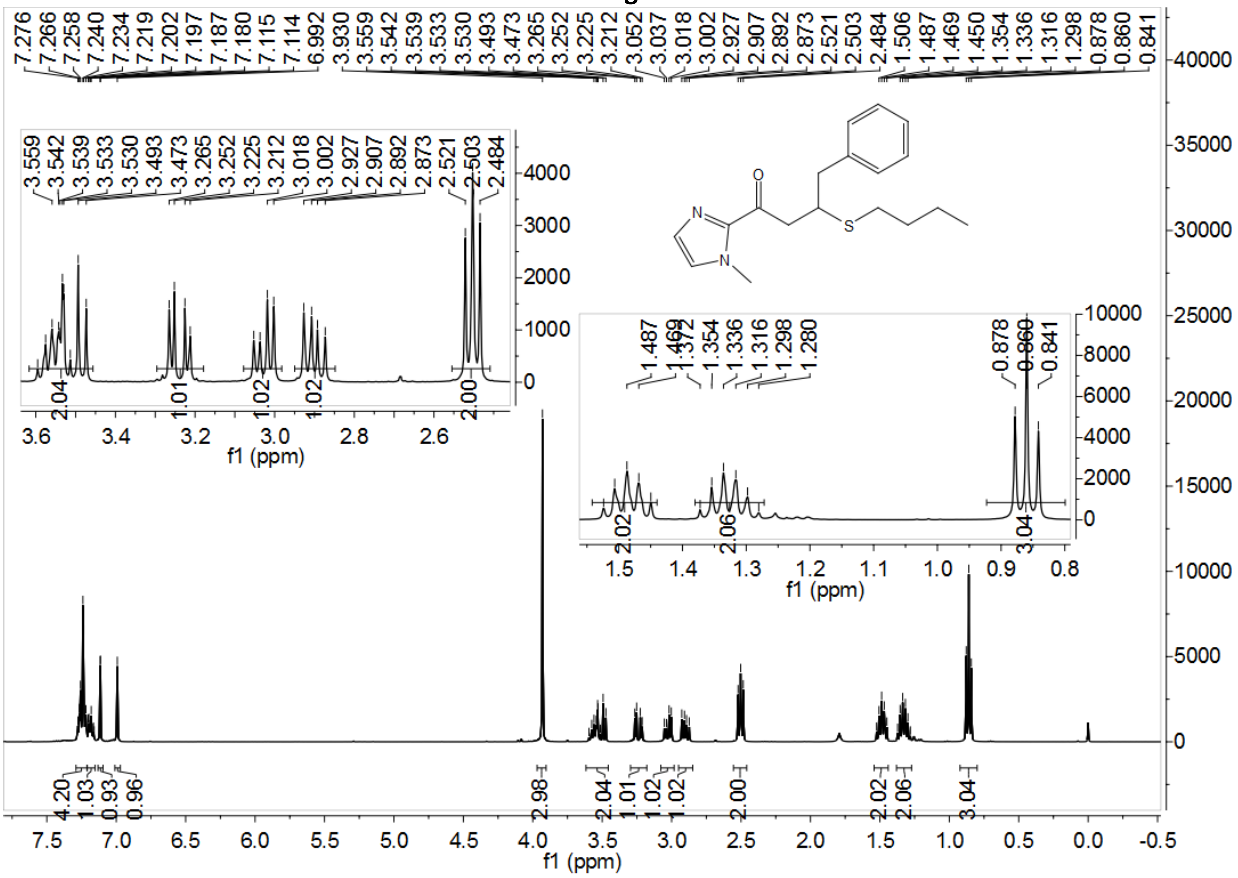

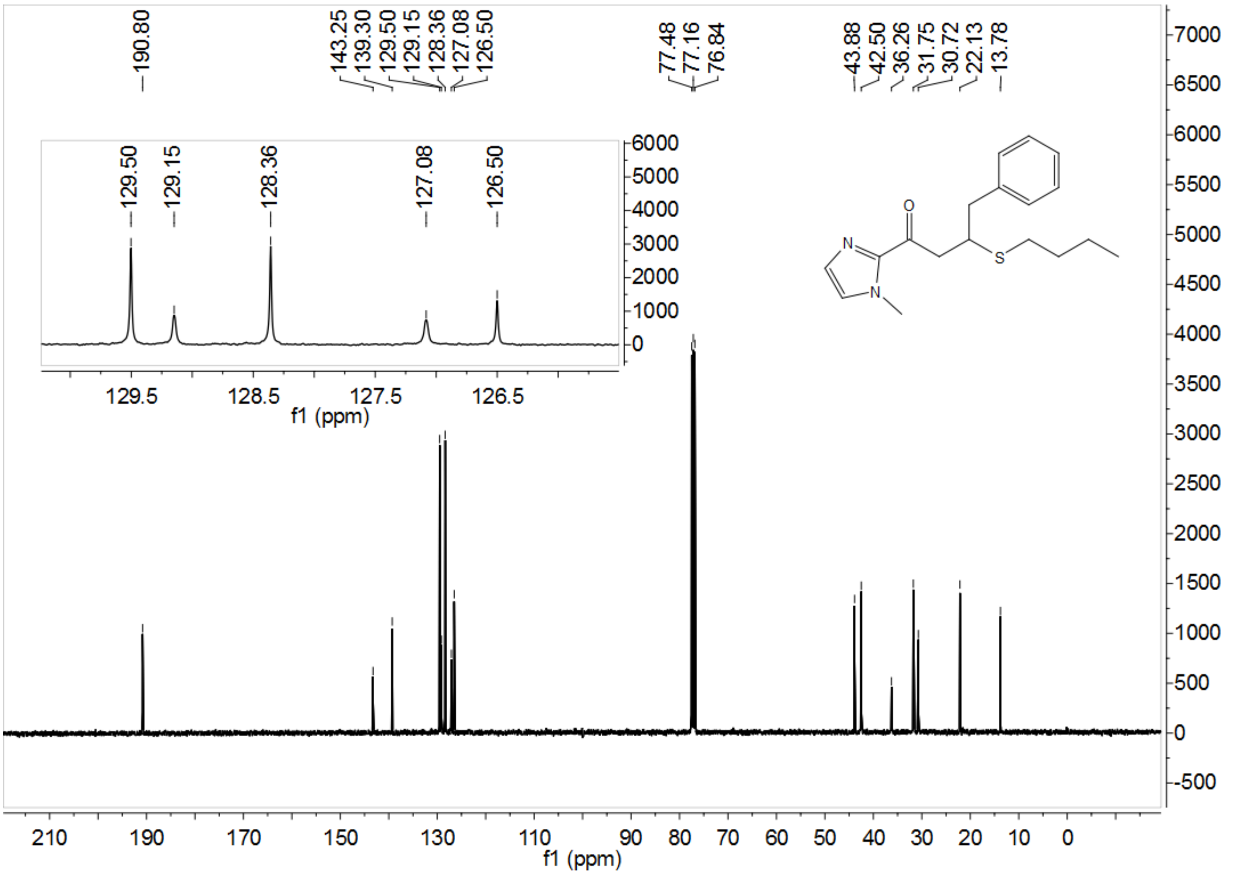

Supplementary Figure 93. ^{1}H and ^{13}C spectra for product **6g**

6h

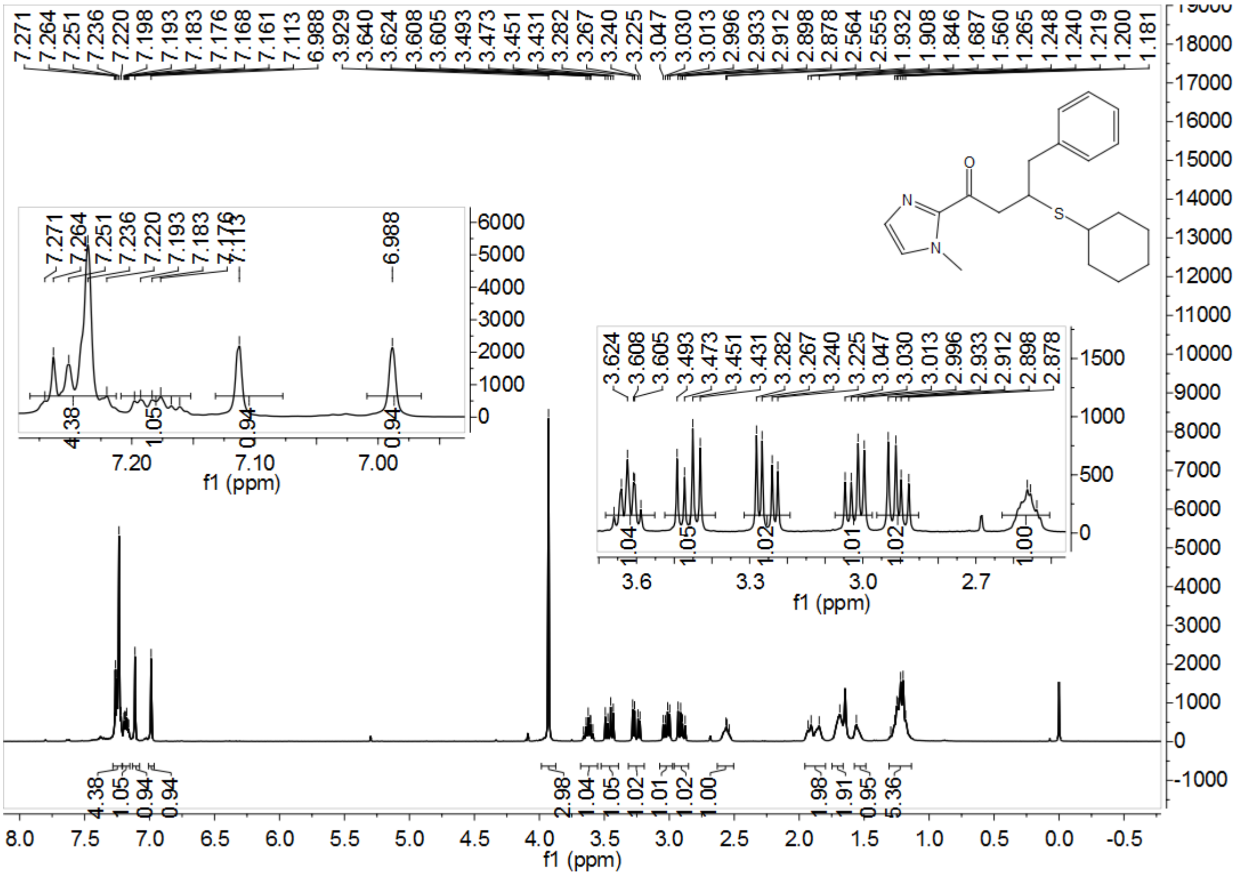

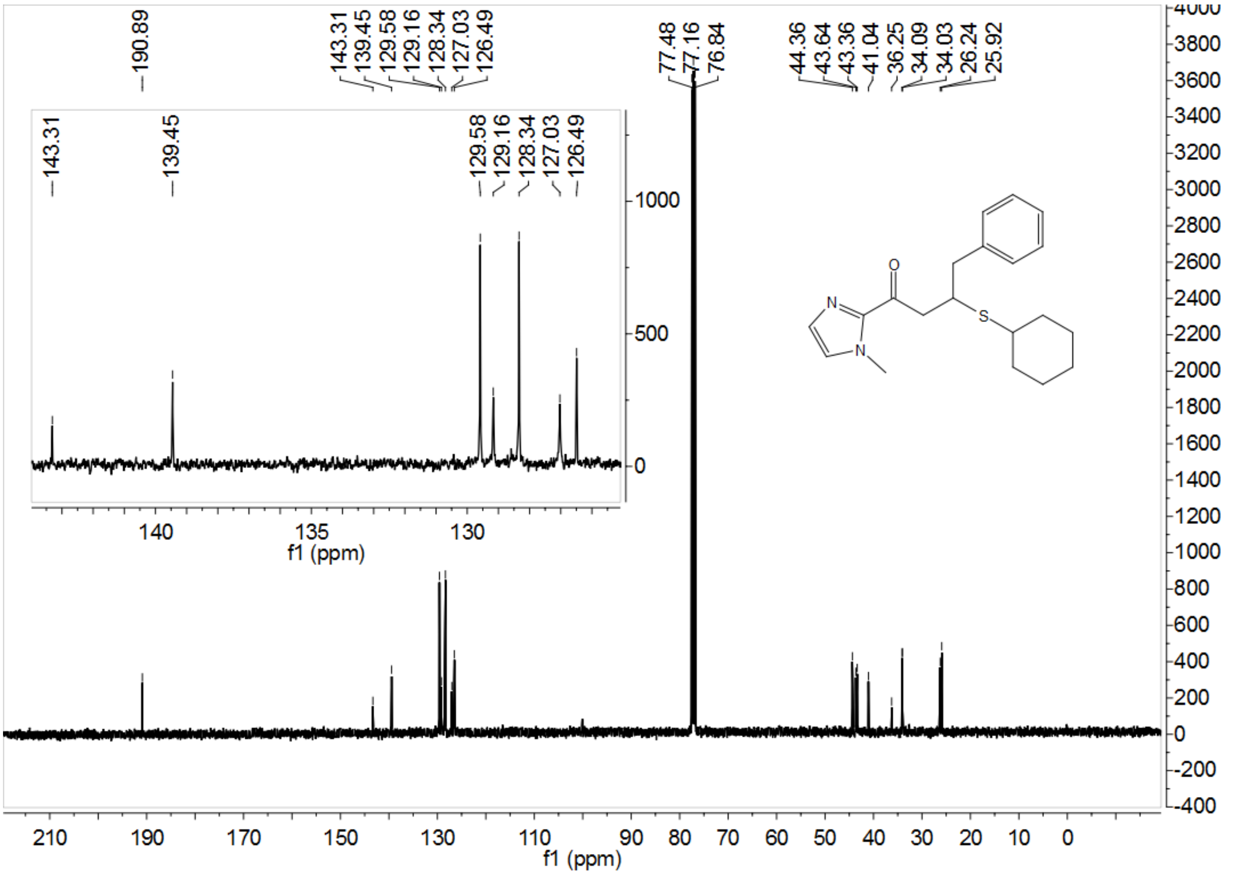

Supplementary Figure 94. ^{1}H and ^{13}C spectra for product **6h**

6i

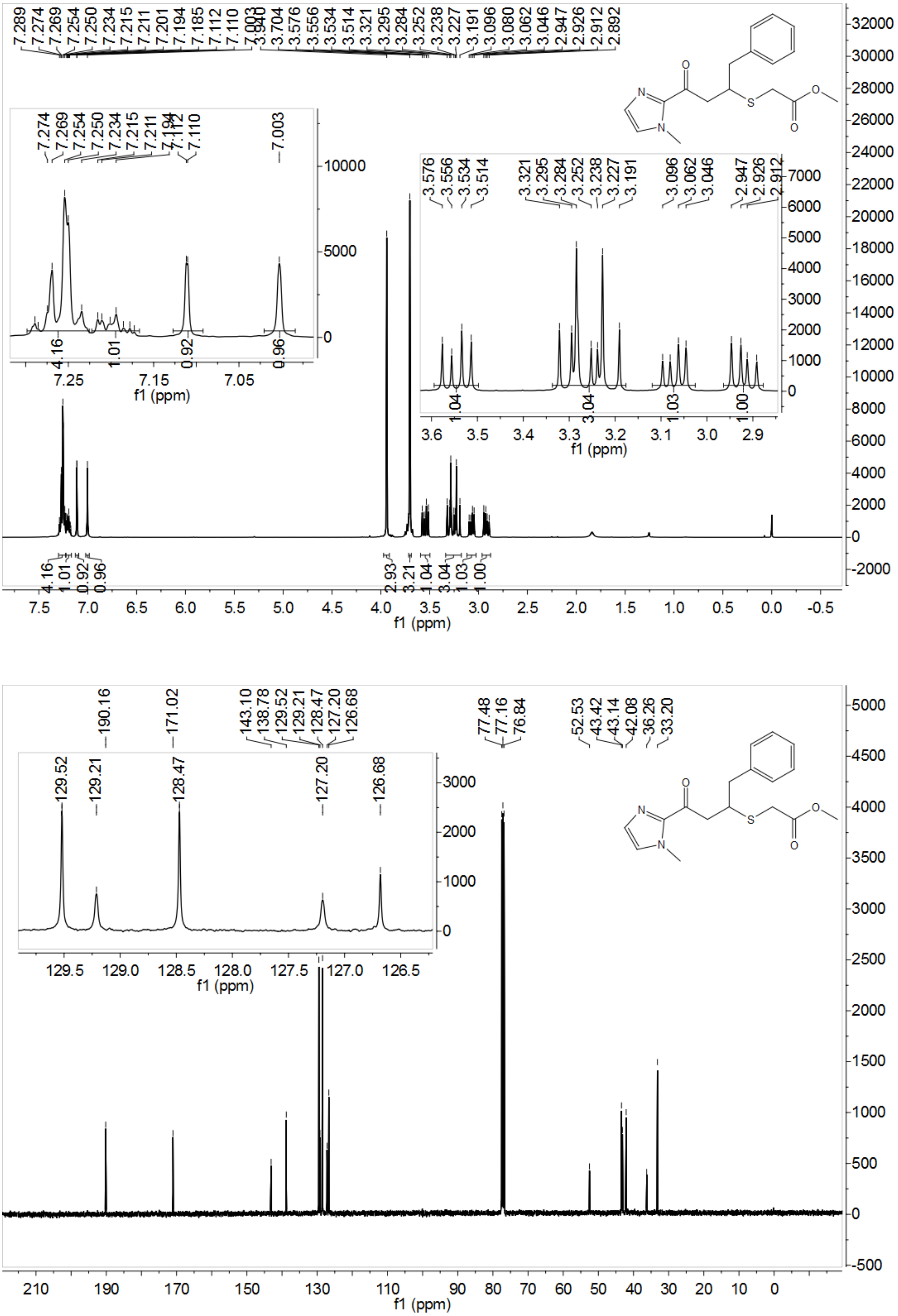

Supplementary Figure 95. ^{1}H and ^{13}C spectra for product **6i**

6j

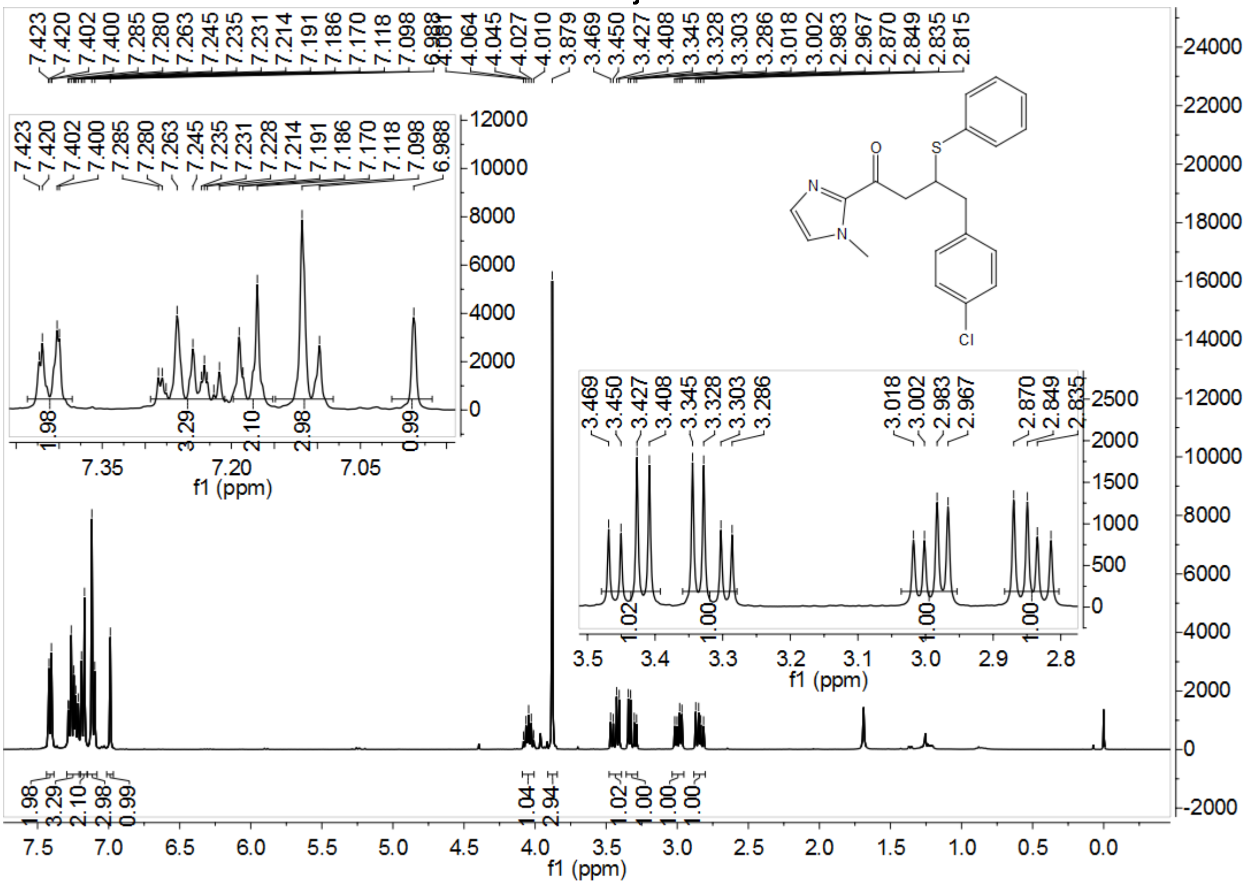

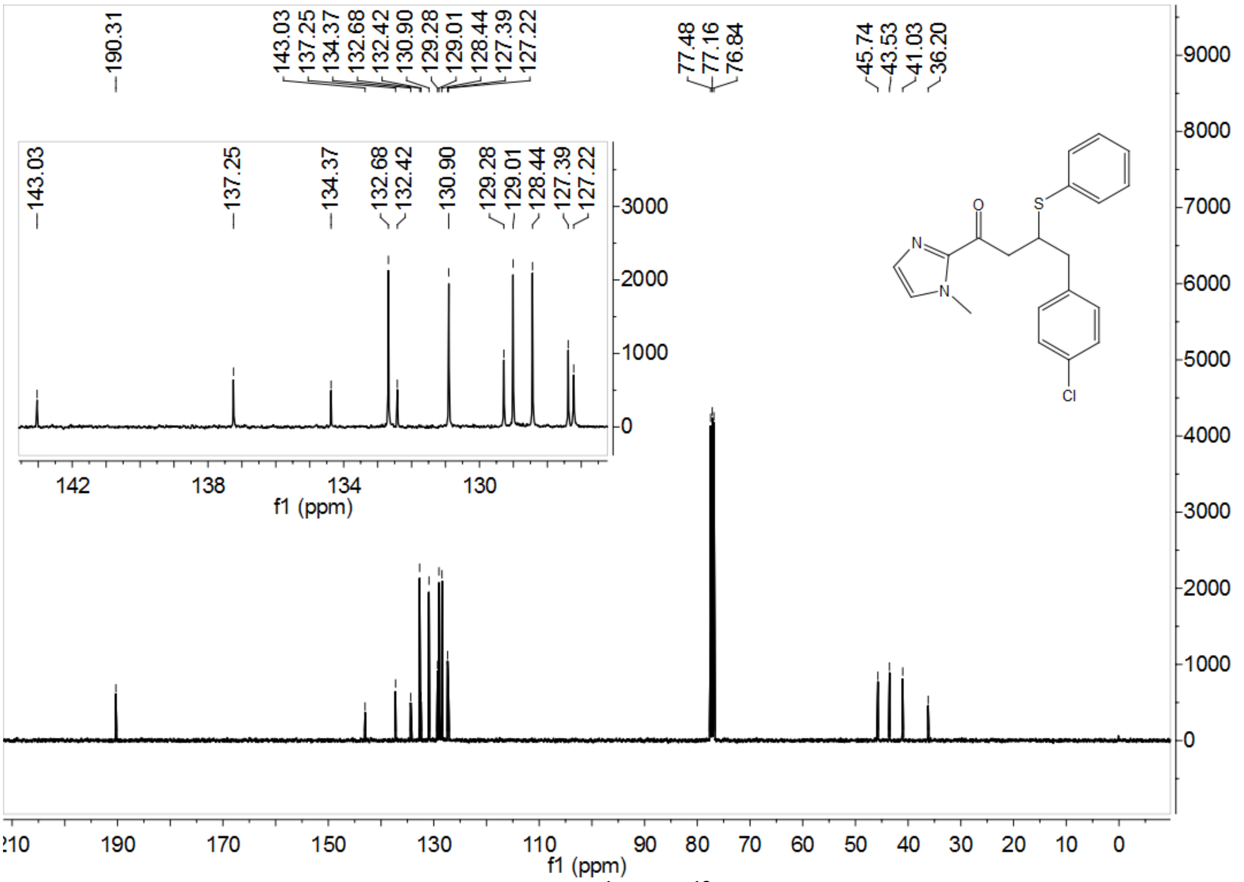

Supplementary Figure 96. ^{1}H and ^{13}C spectra for product **6j**

6k

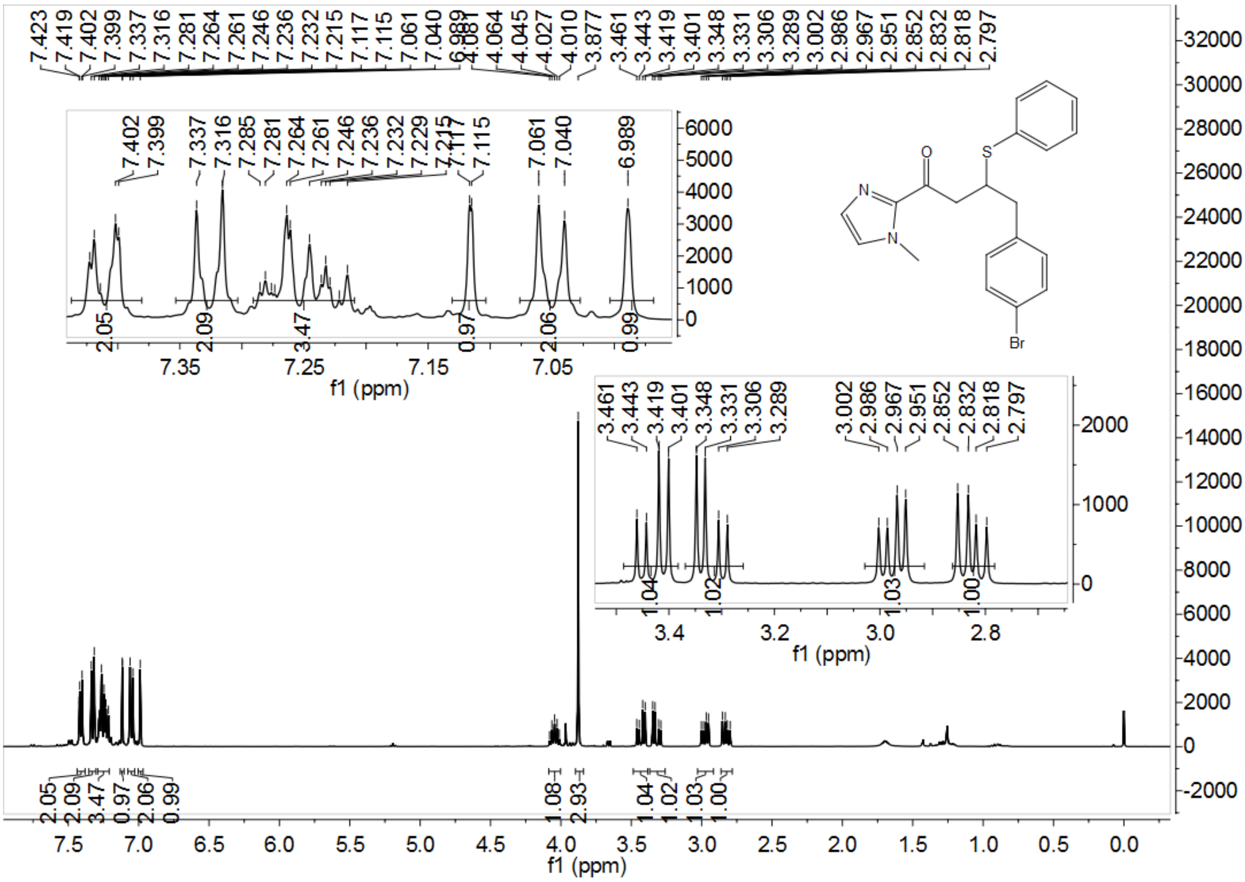

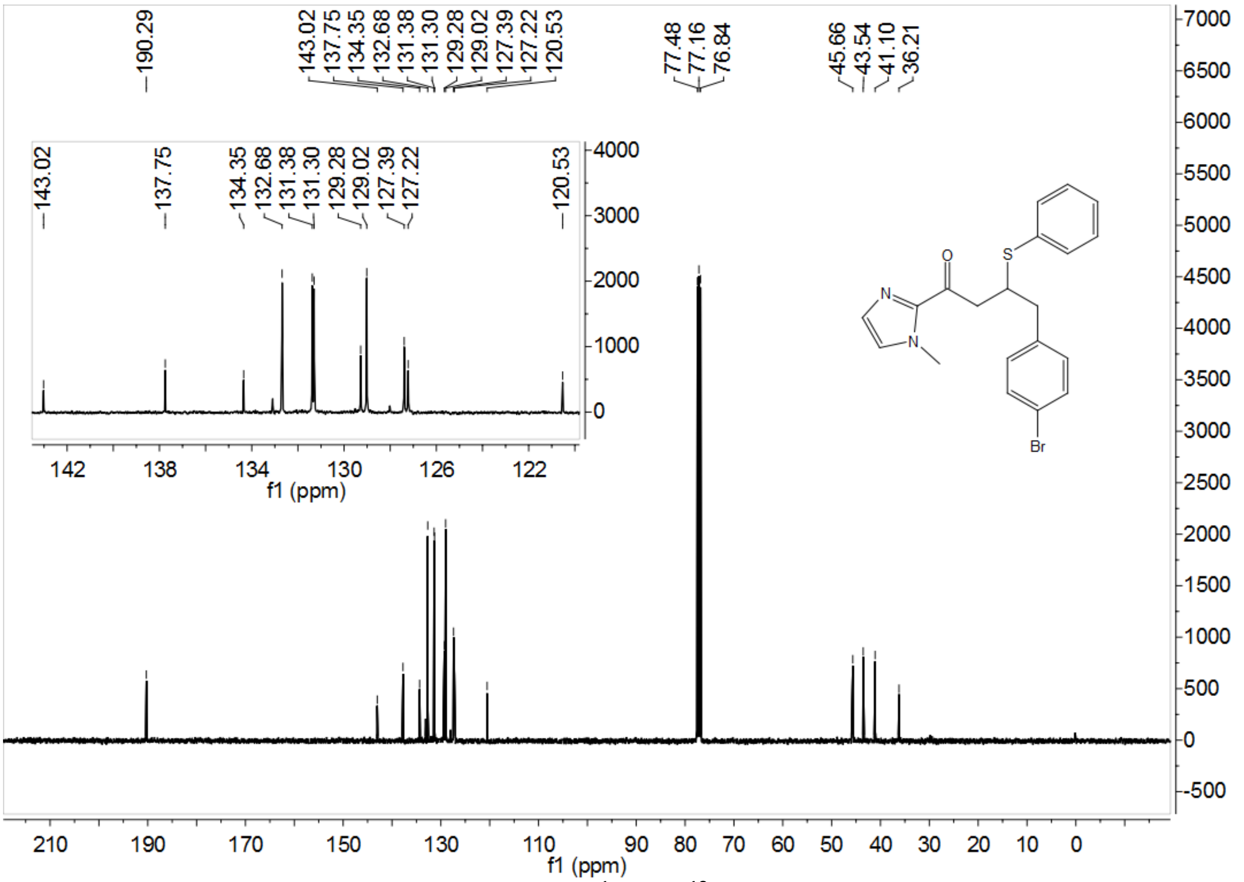

Supplementary Figure 97. ^{1}H and ^{13}C spectra for product **6k**

6l

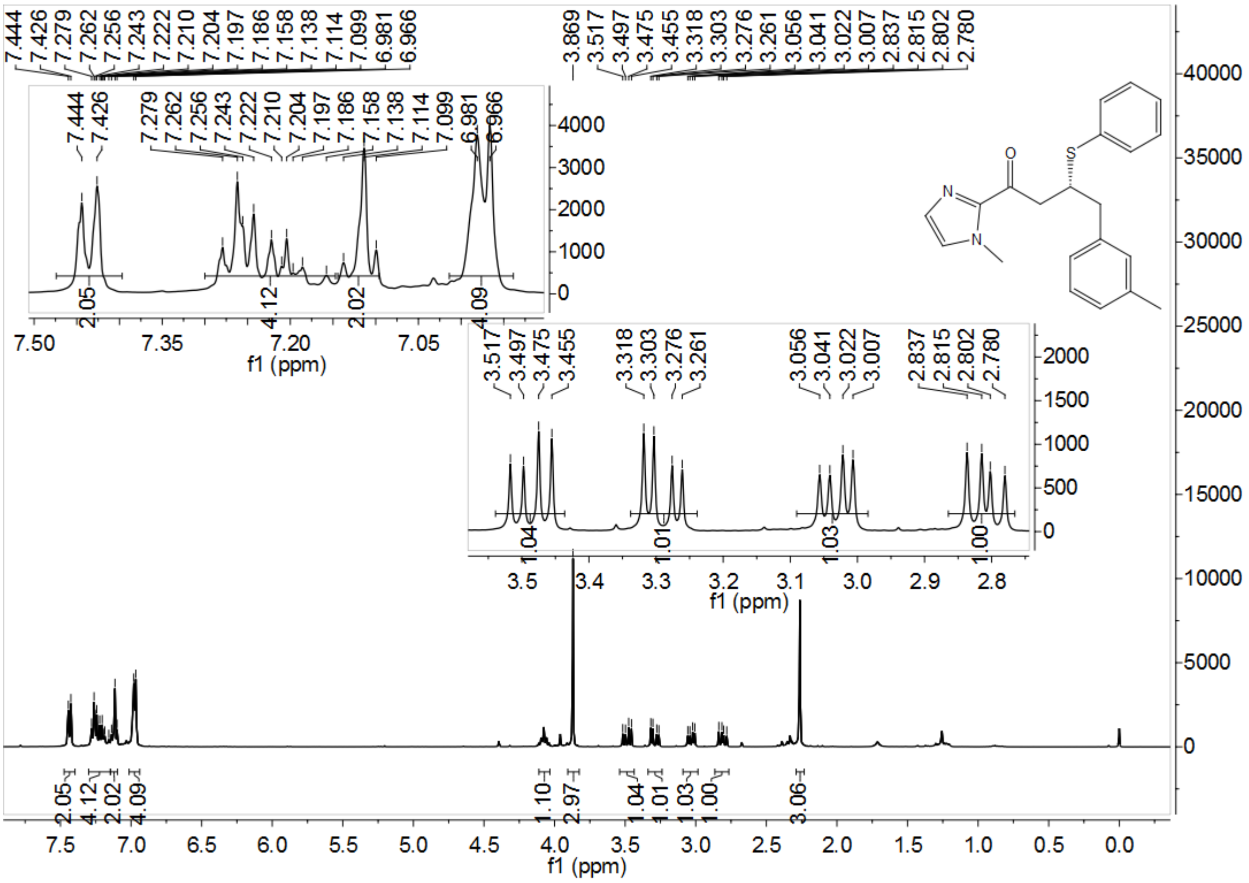

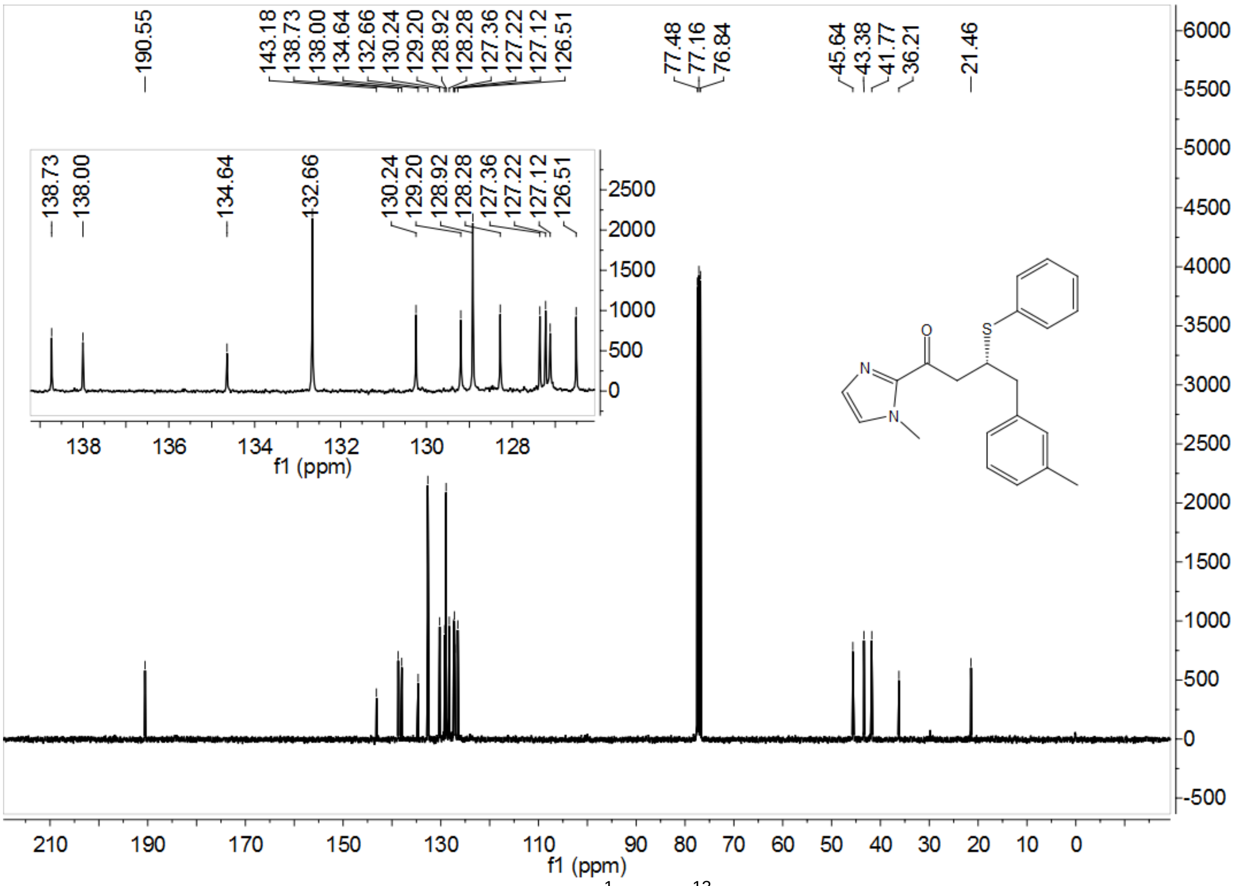

Supplementary Figure 98. ^{1}H and ^{13}C spectra for product **6l**

6m

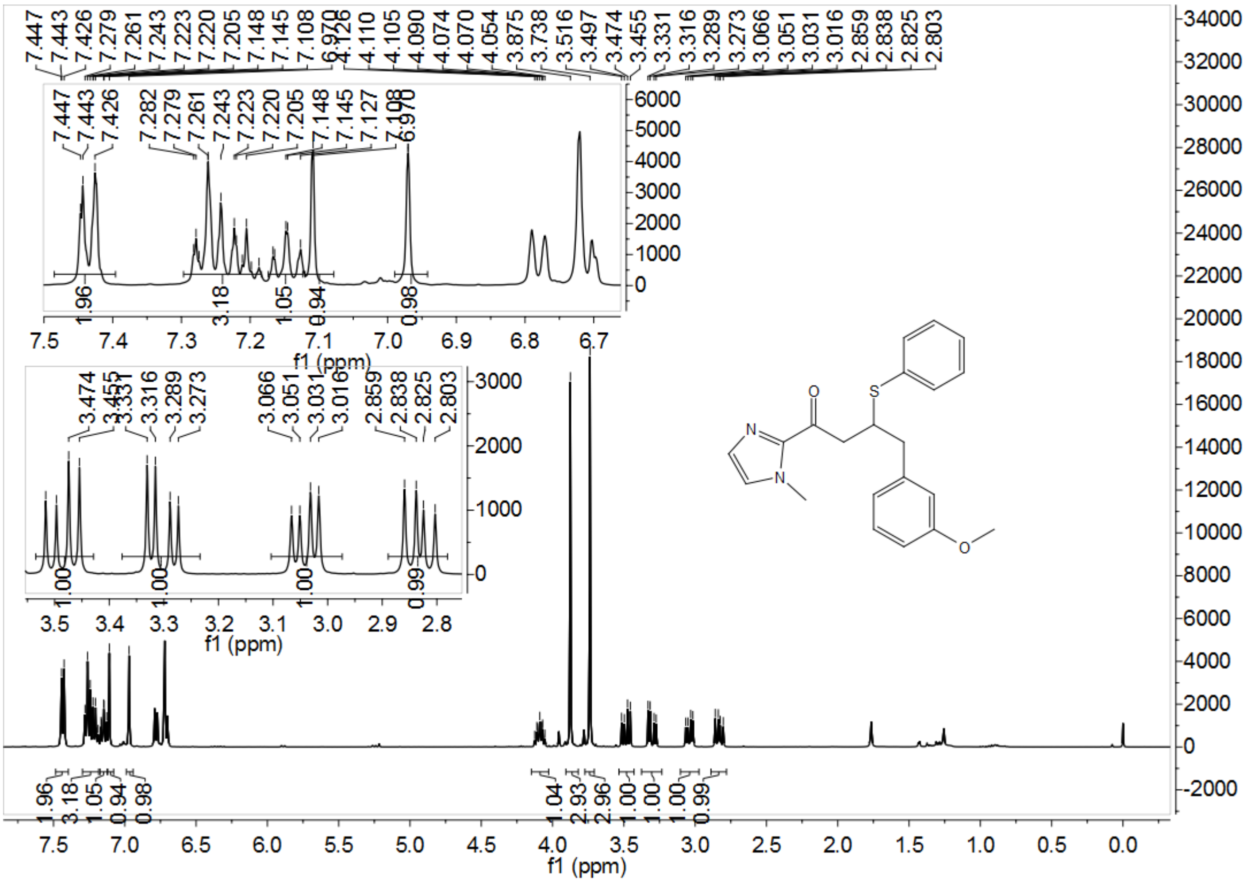

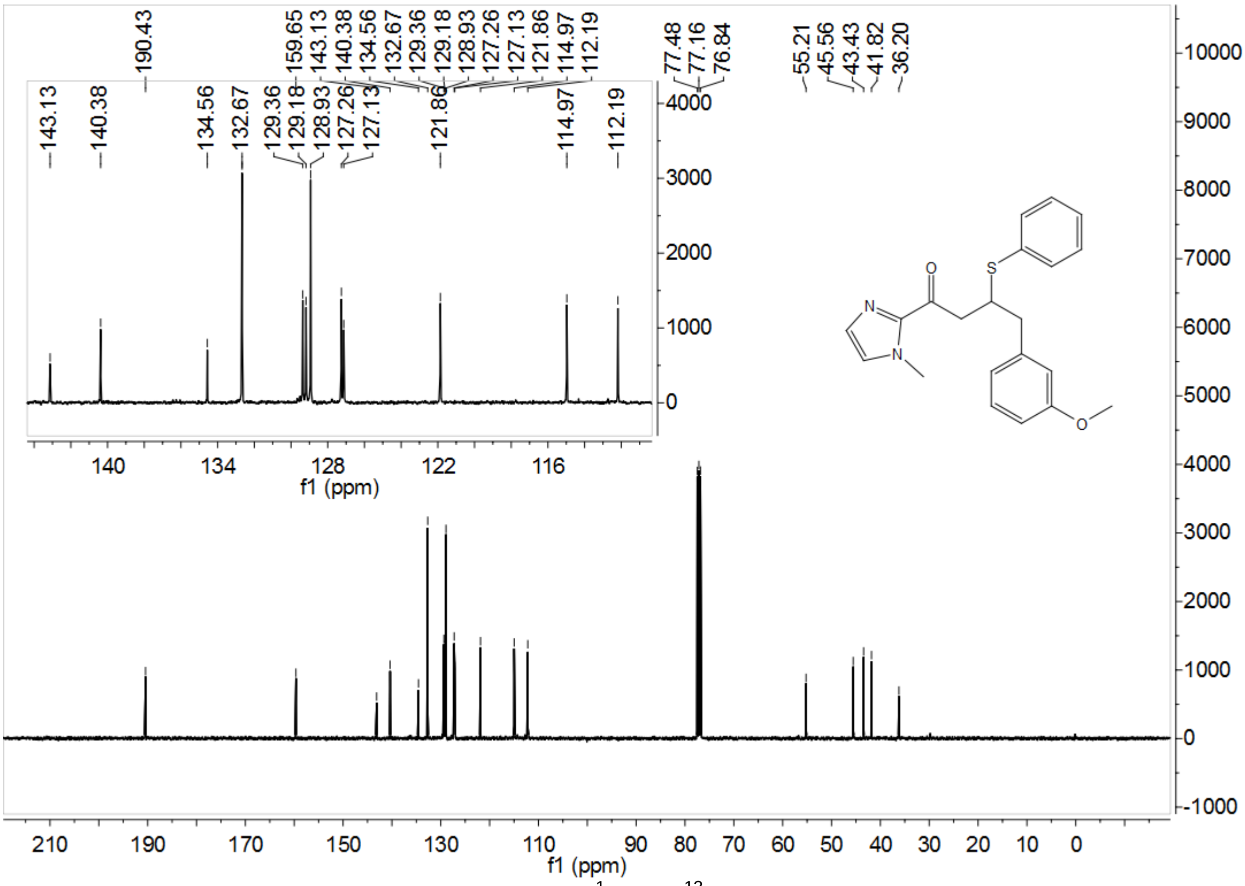

Supplementary Figure 99. ^{1}H and ^{13}C spectra for product **6m**

6n

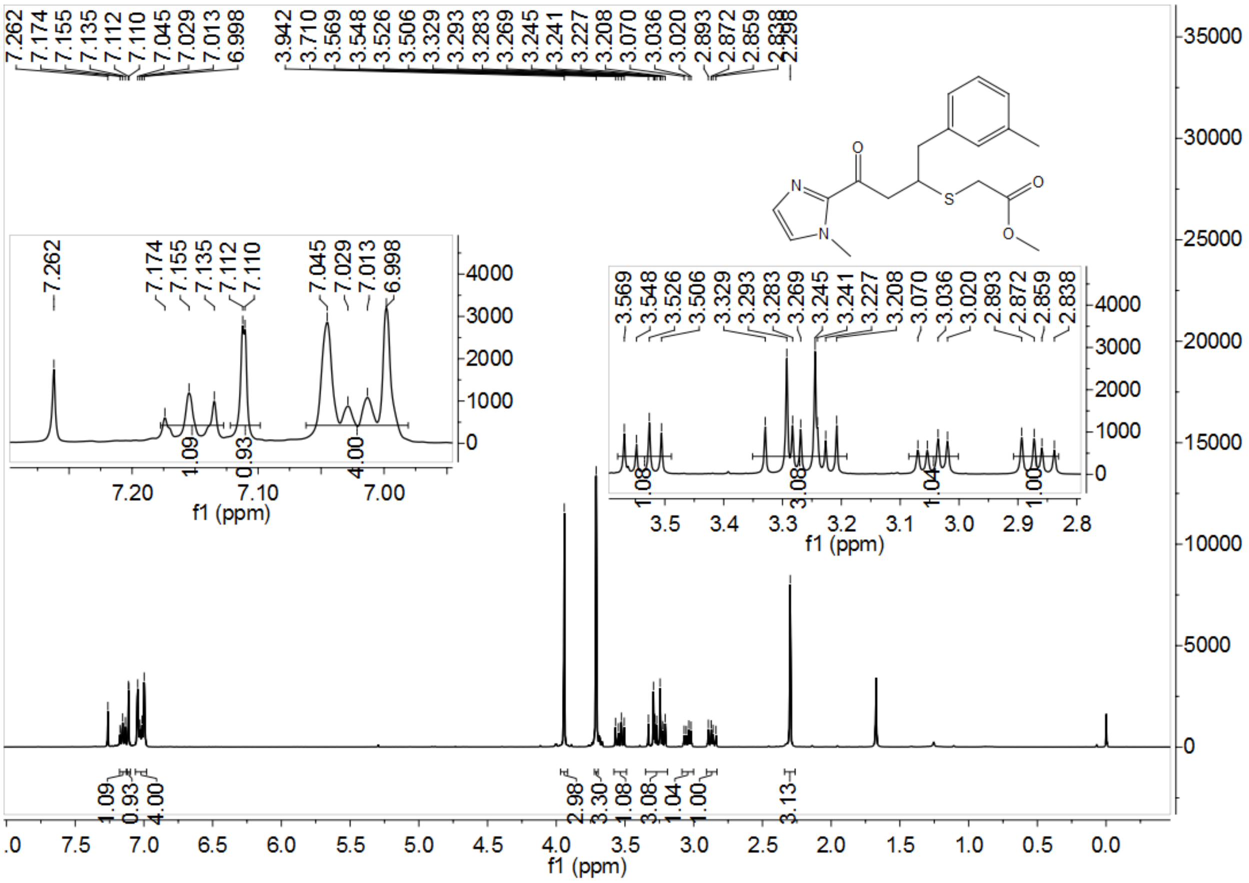

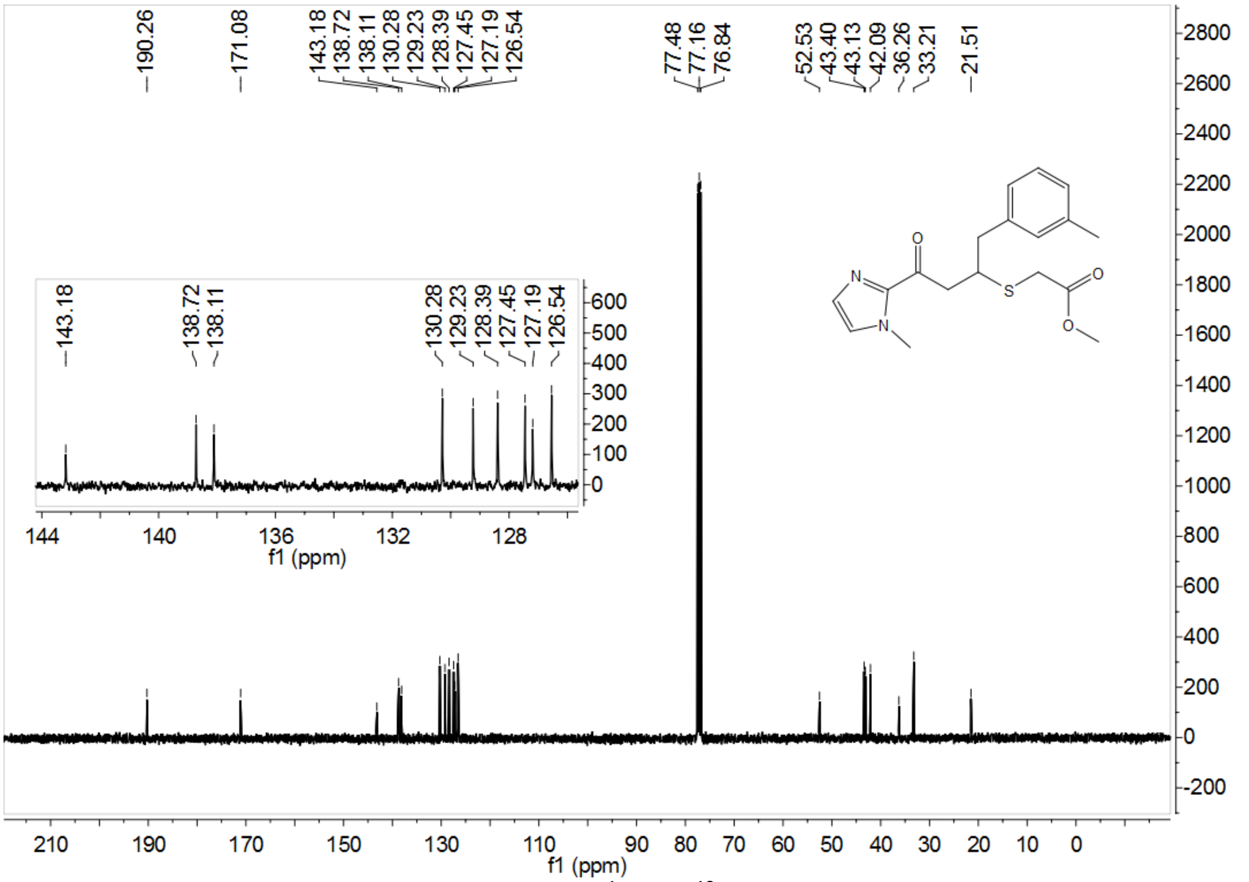

Supplementary Figure 100. ^{1}H and ^{13}C spectra for product **6n**

6o

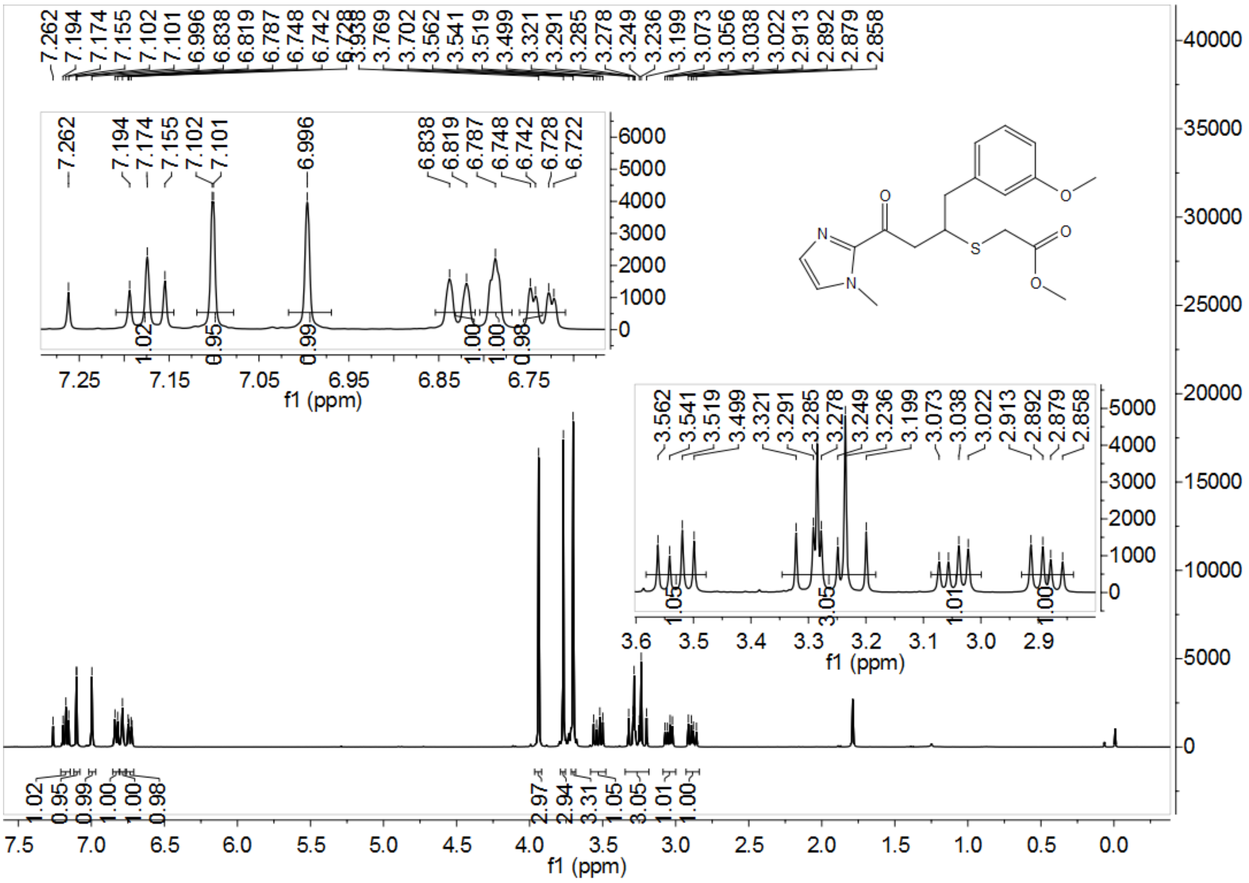

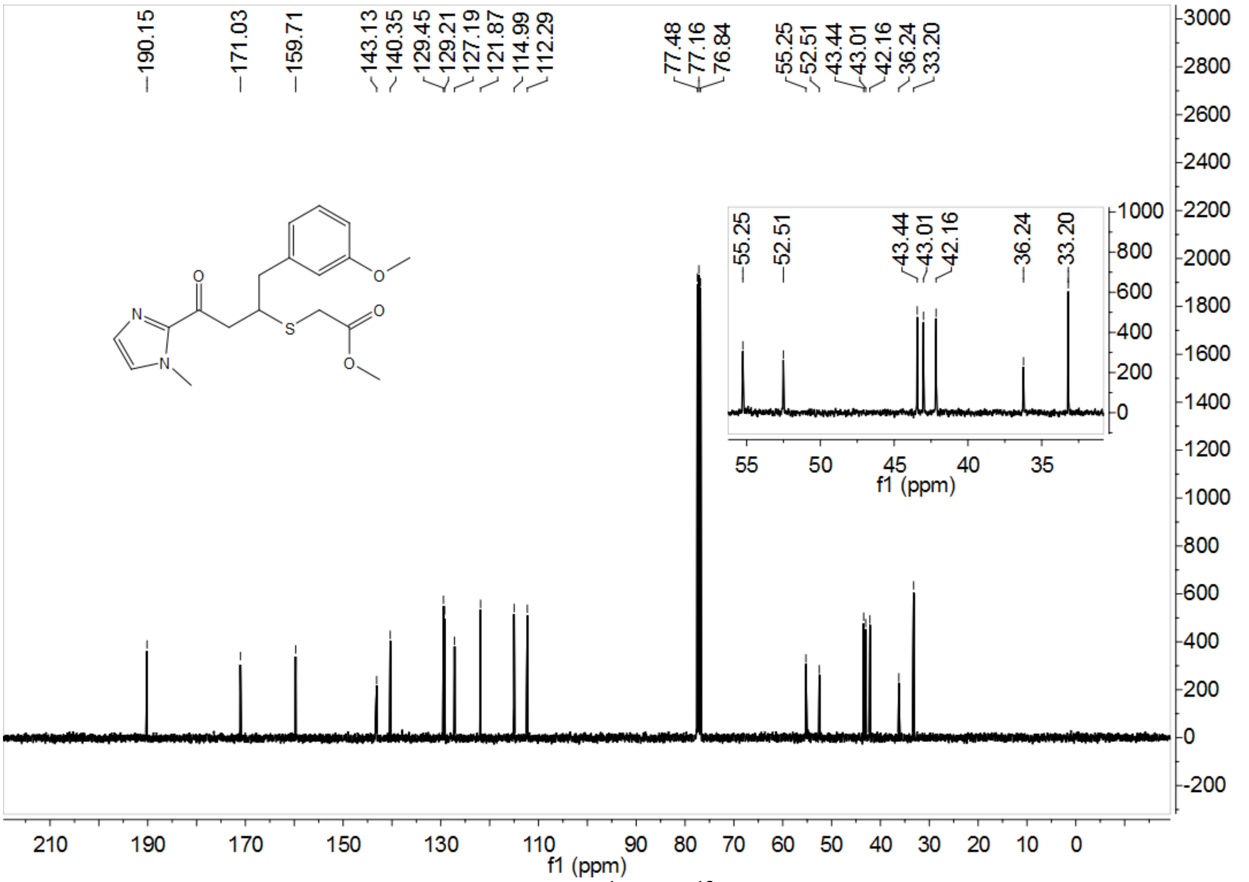

Supplementary Figure 101. ^{1}H and ^{13}C spectra for product **6o**

6p

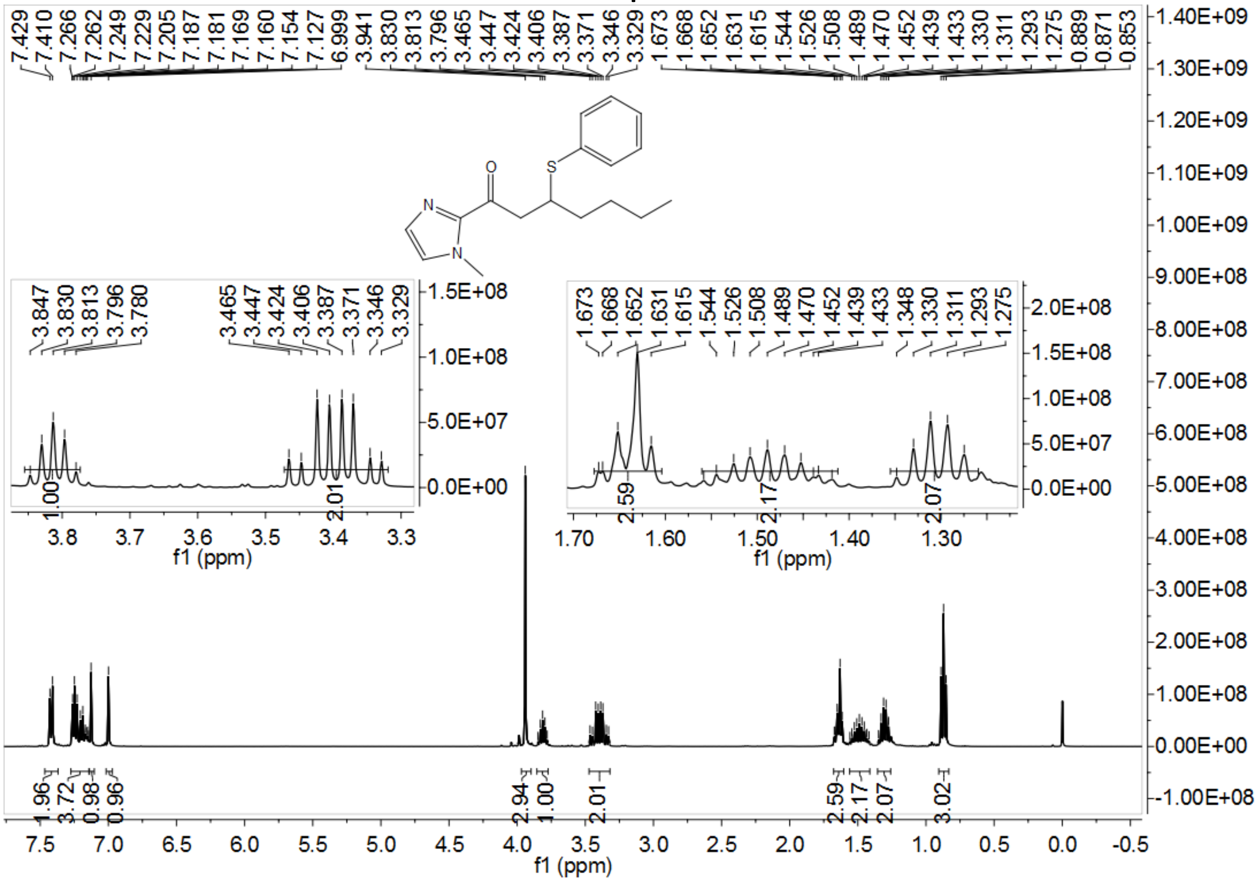

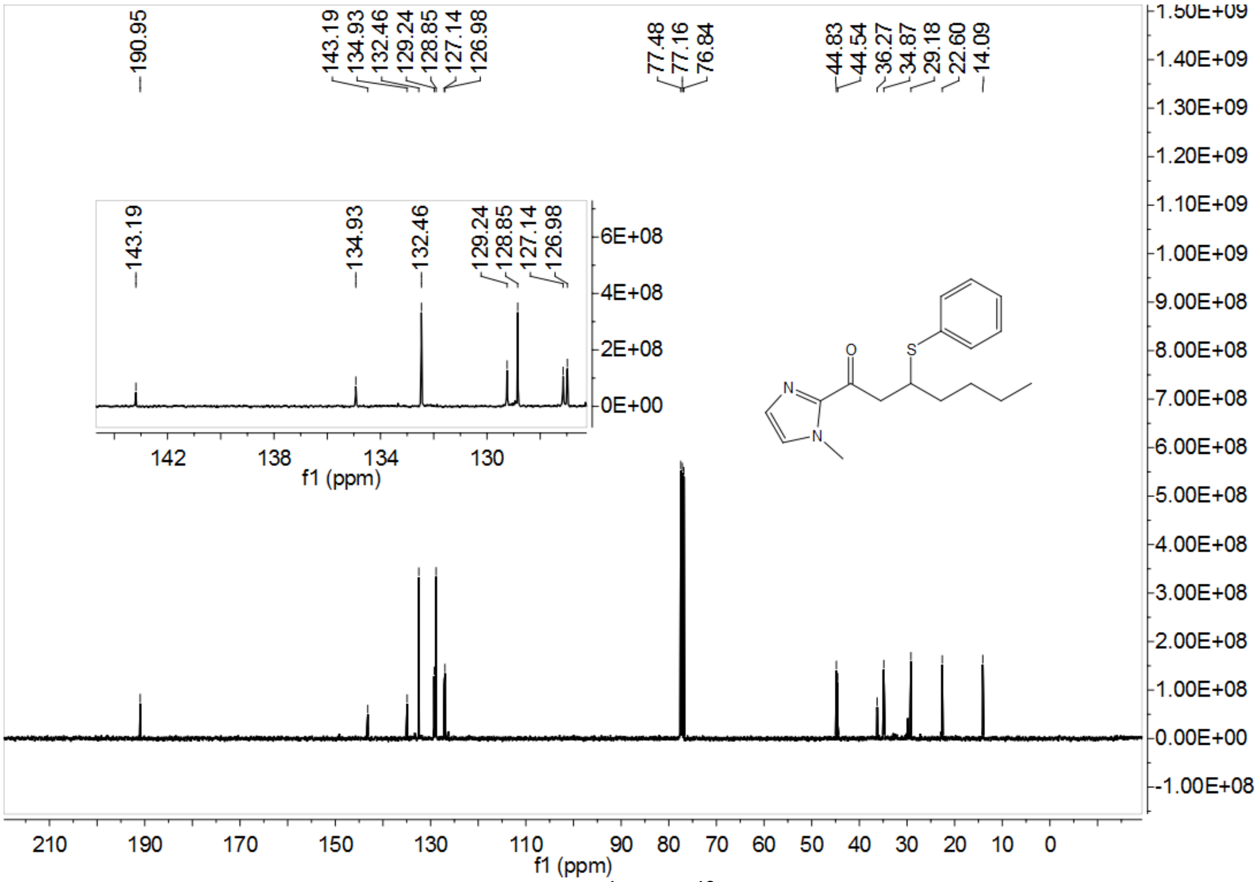

Supplementary Figure 102. ^{1}H and ^{13}C spectra for product **6p**

7

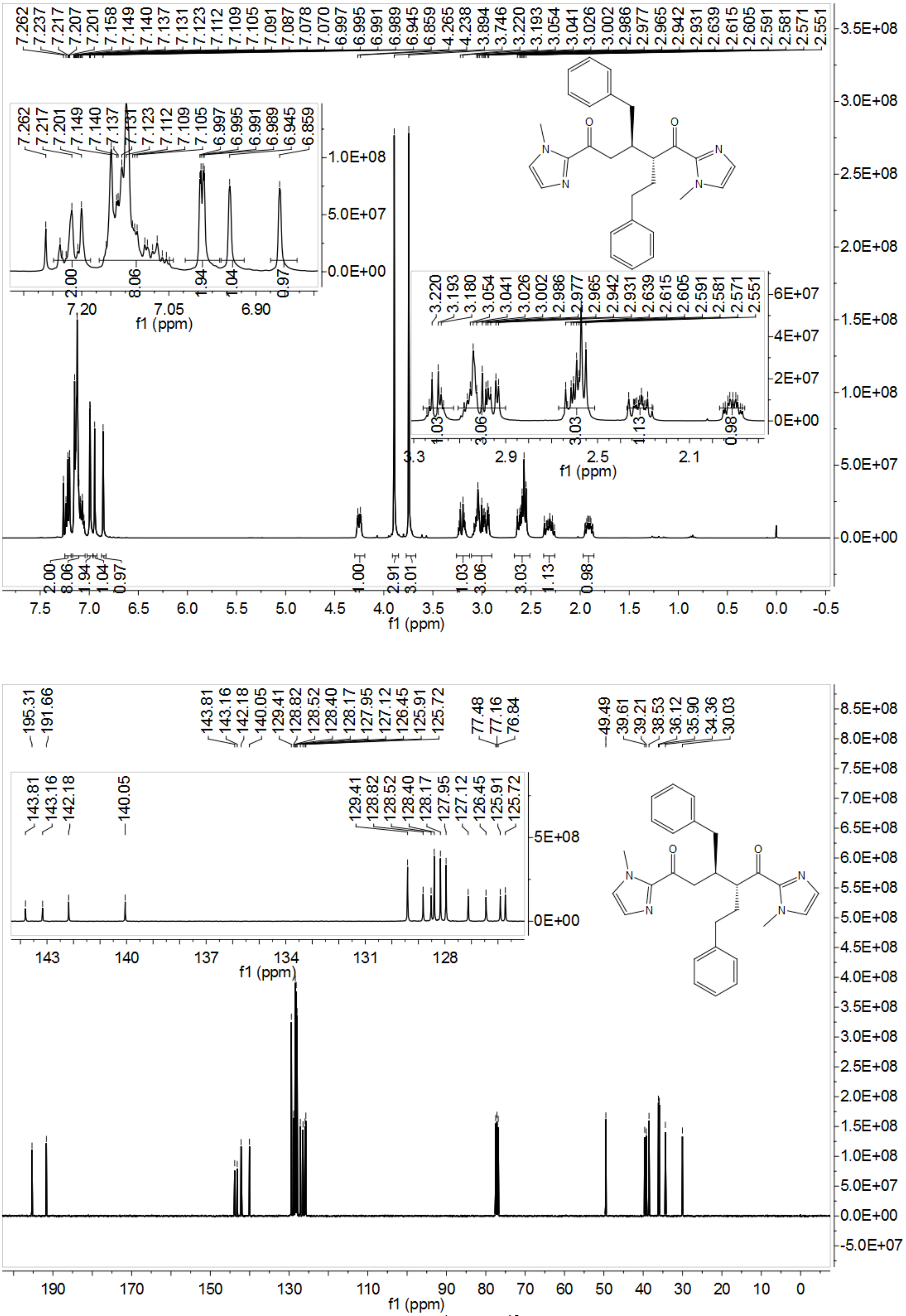

Supplementary Figure 103. ^{1}H and ^{13}C spectra for product **7**

8

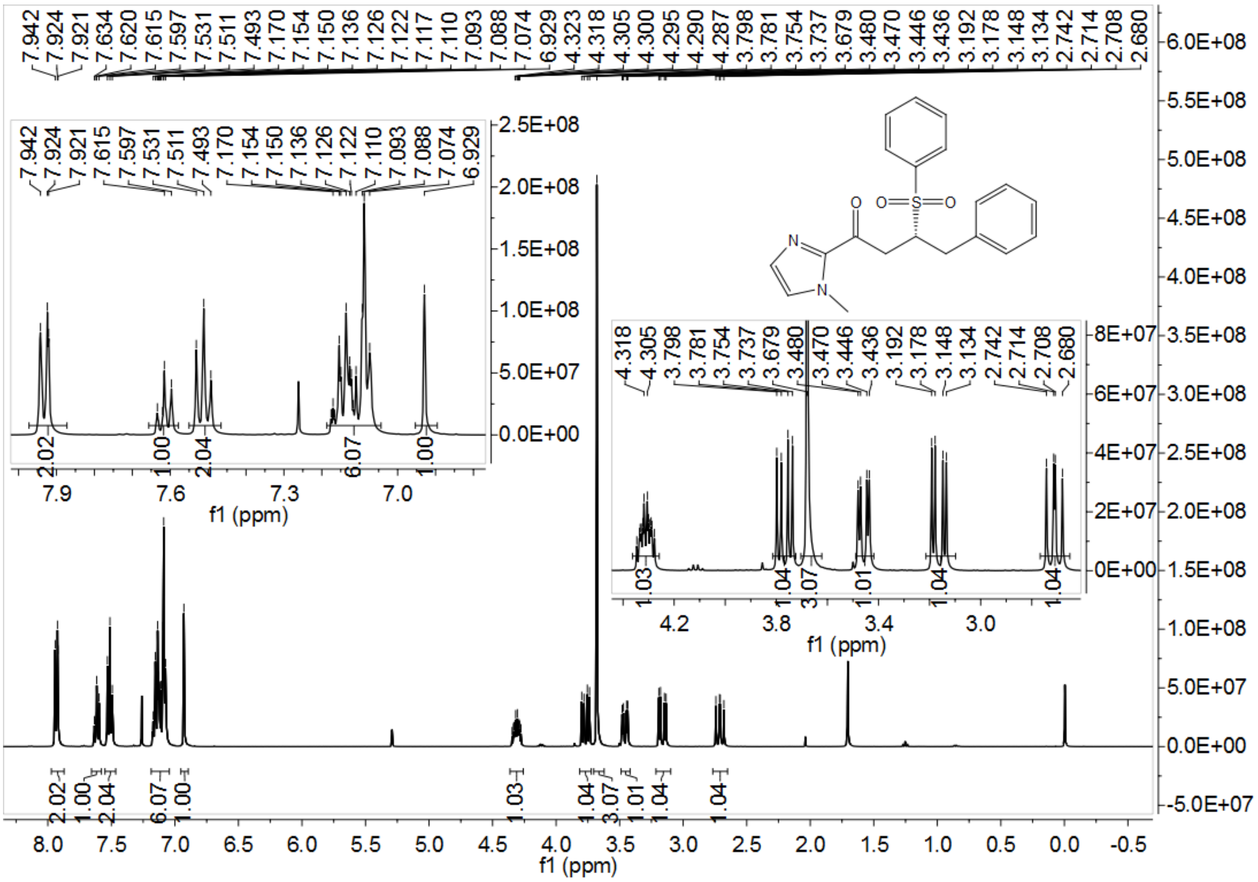

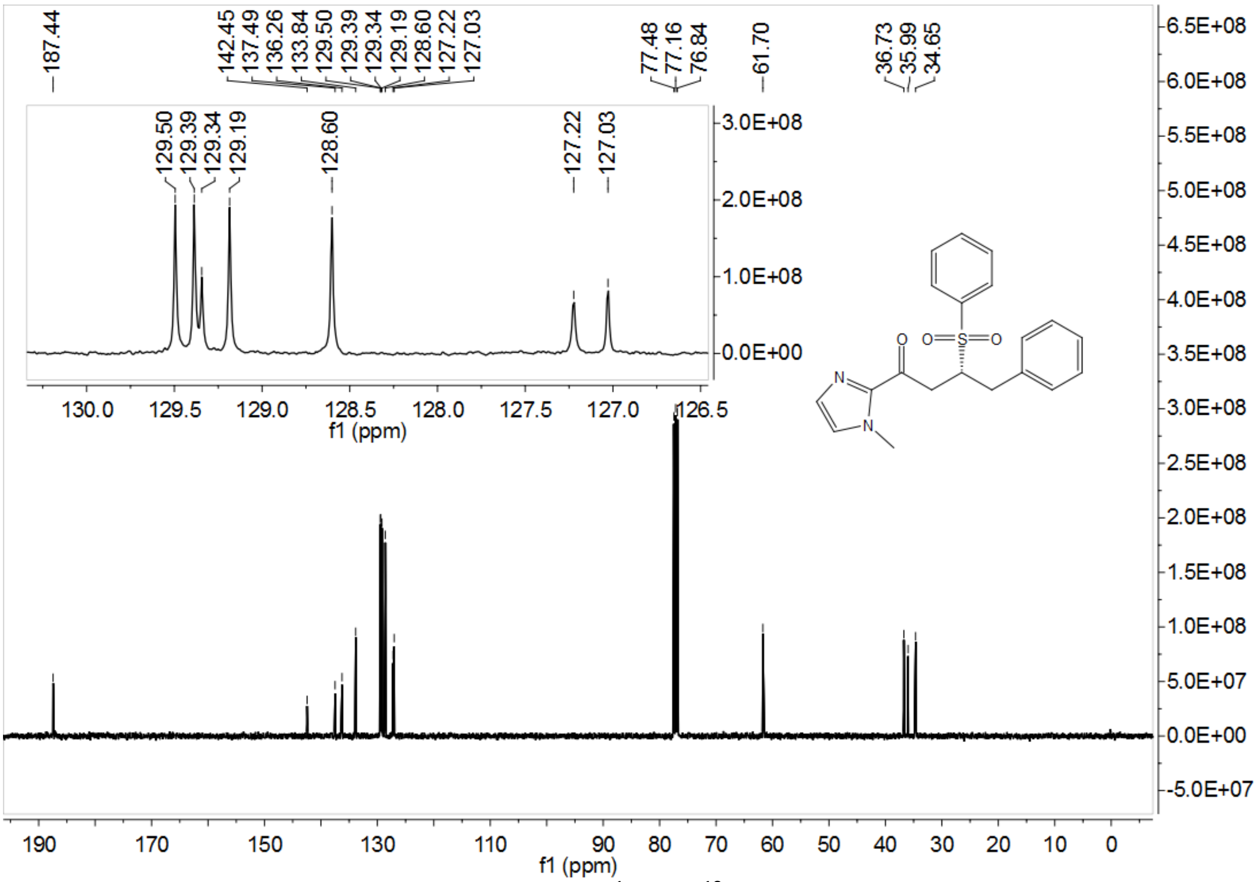

Supplementary Figure 104. ^{1}H and ^{13}C spectra for product **8**

9

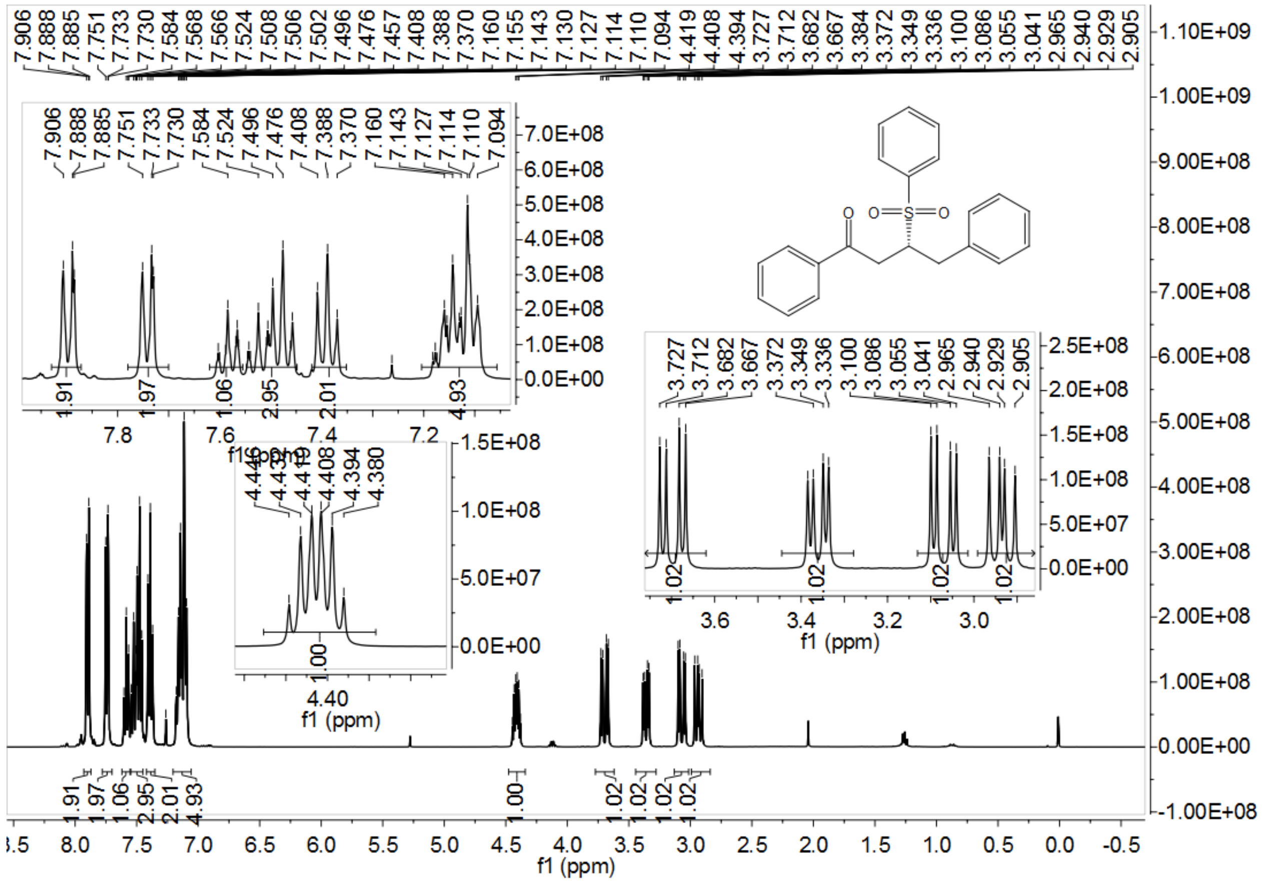

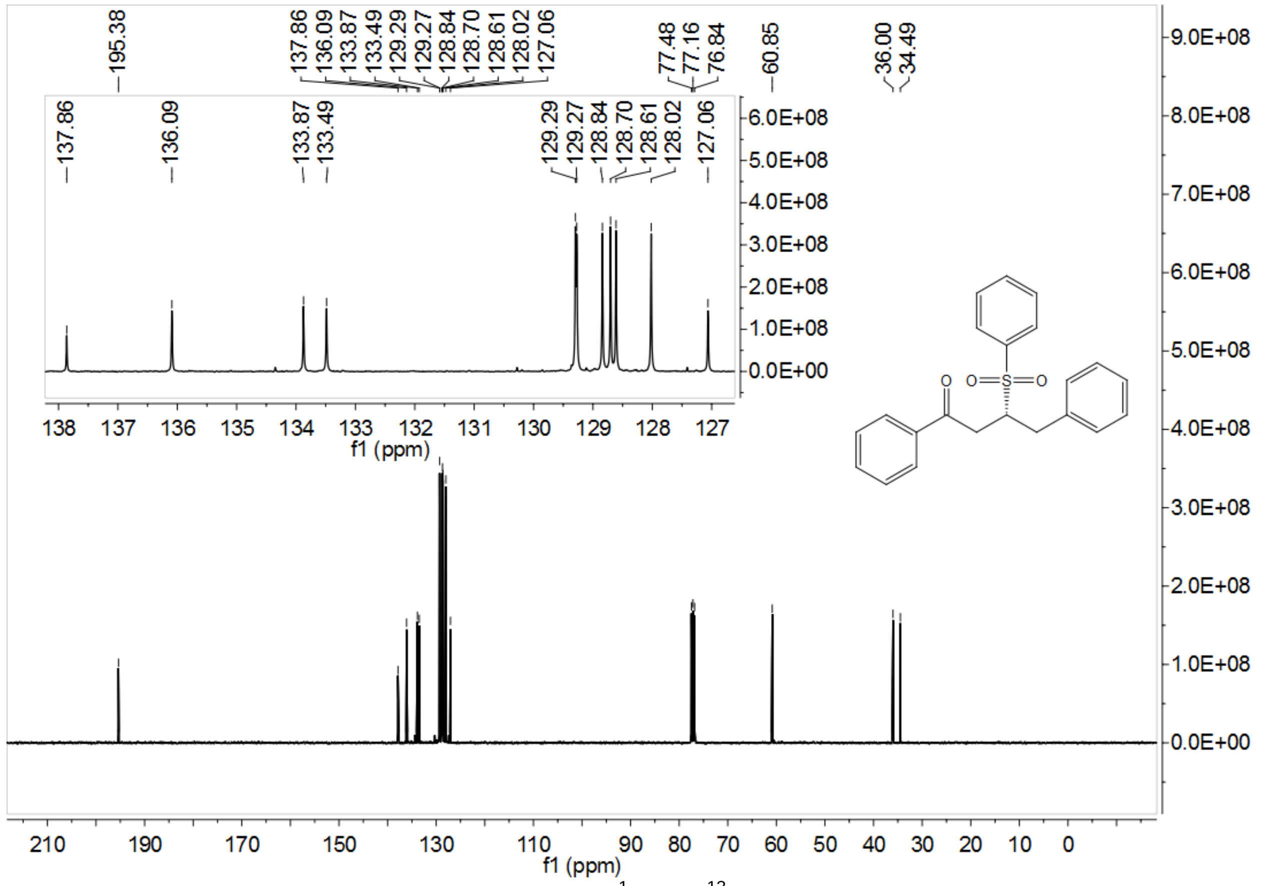

Supplementary Figure 105. ^{1}H and ^{13}C spectra for product **9**

11

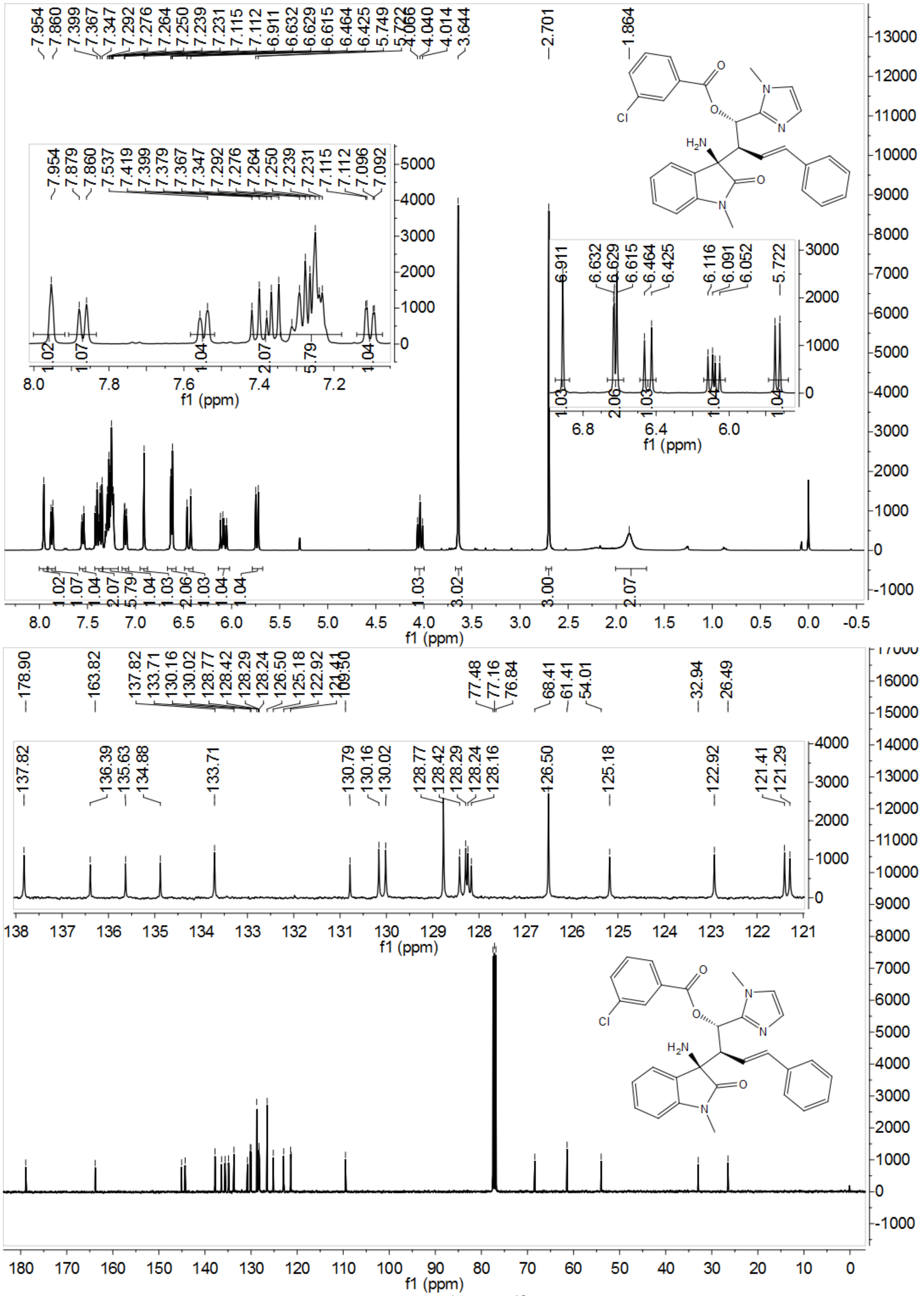

Supplementary Figure 106. ^{1}H and ^{13}C spectra for product **11**

Copy of CD spectra in MeOH

2j

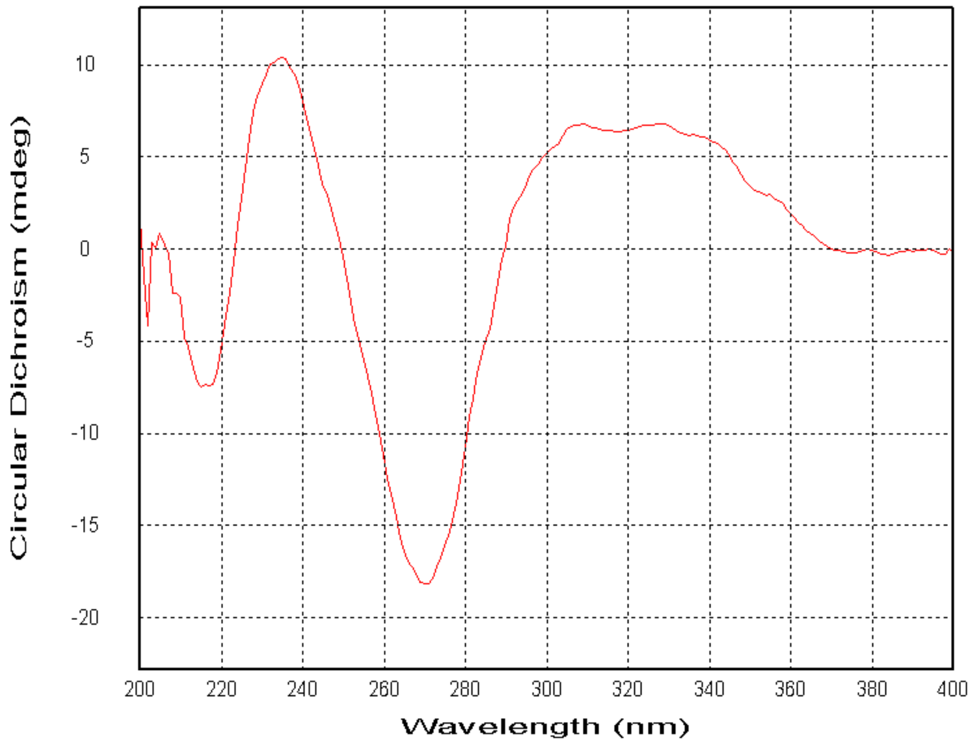

Supplementary Figure 107. CD spectra for product **2j**

2a

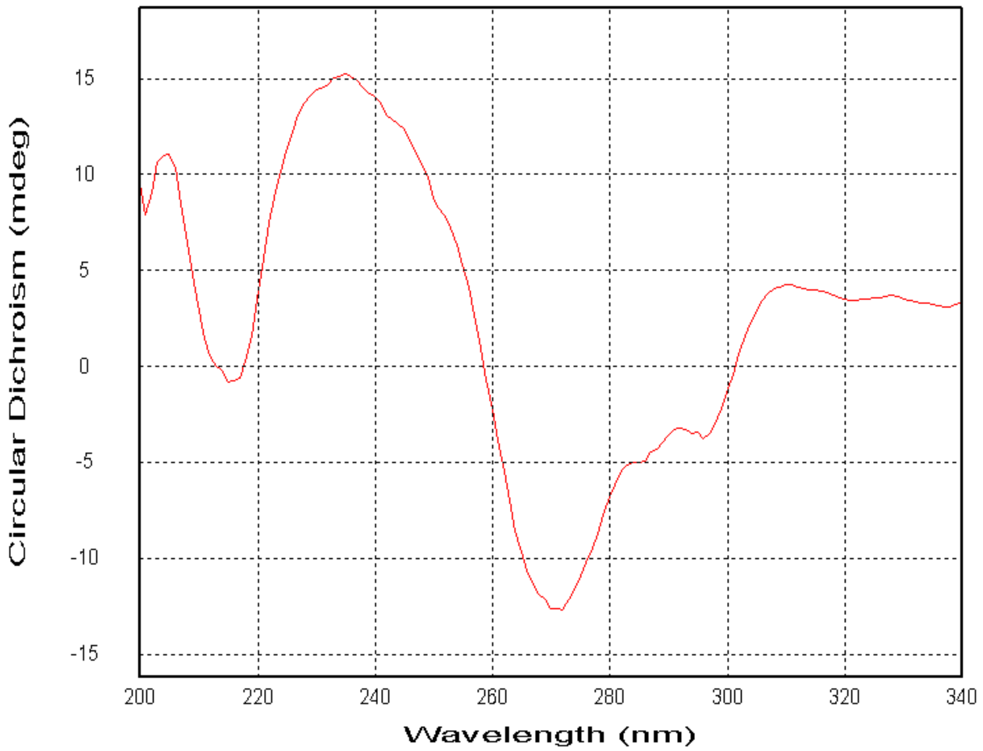

Supplementary Figure 108. CD spectra for product **2a**

2b

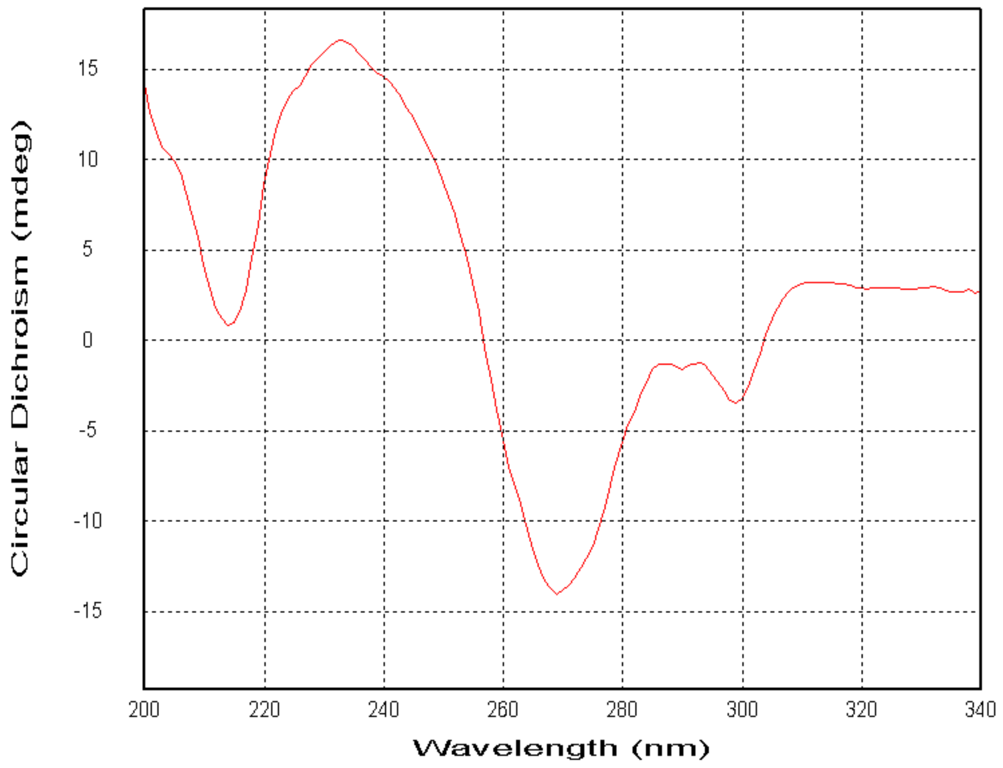

Supplementary Figure 109. CD spectra for product **2b**

2c

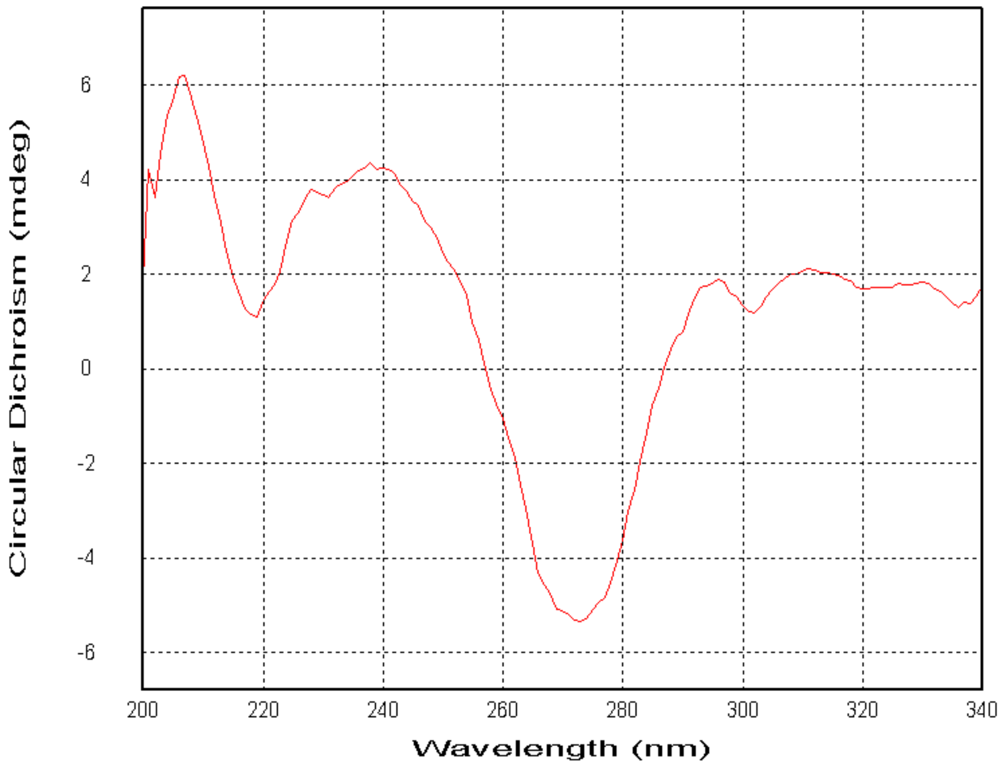

Supplementary Figure 110. CD spectra for product **2c**

2d

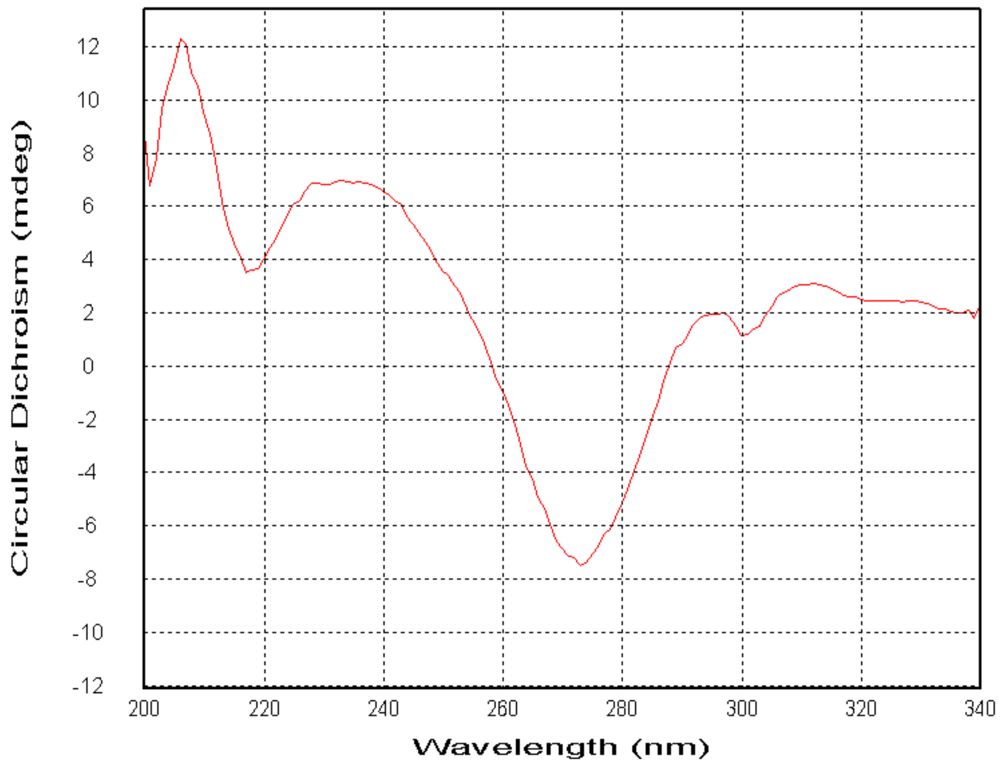

Supplementary Figure 111. CD spectra for product **2d**

2e

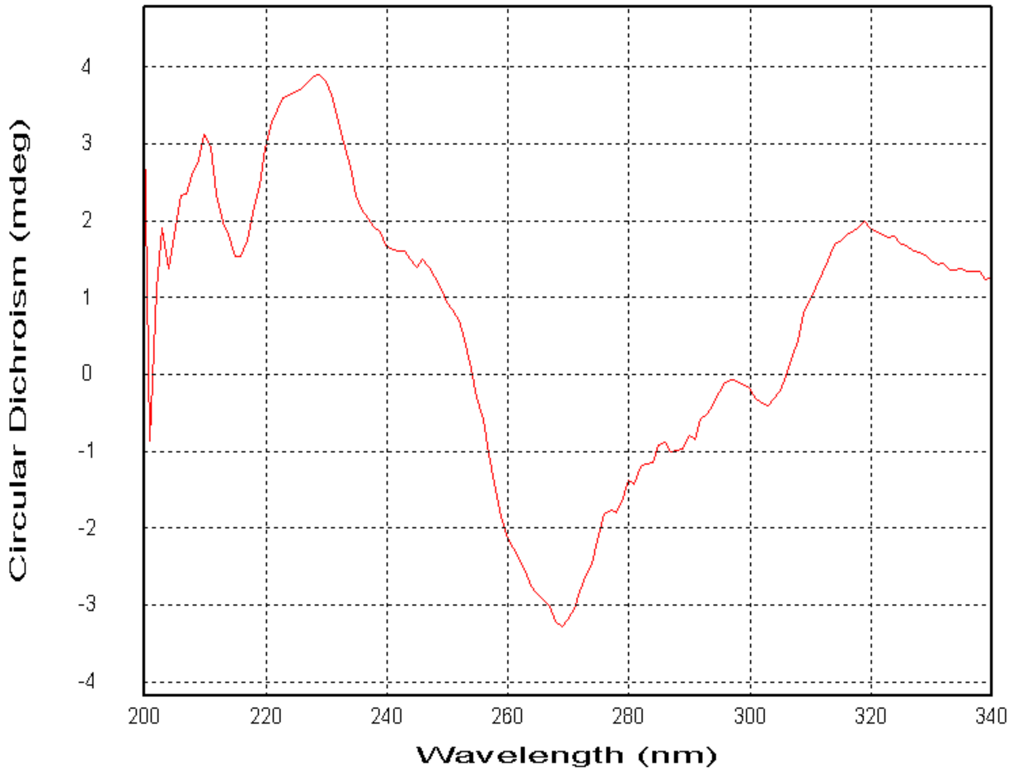

Supplementary Figure 112. CD spectra for product **2e**

2f

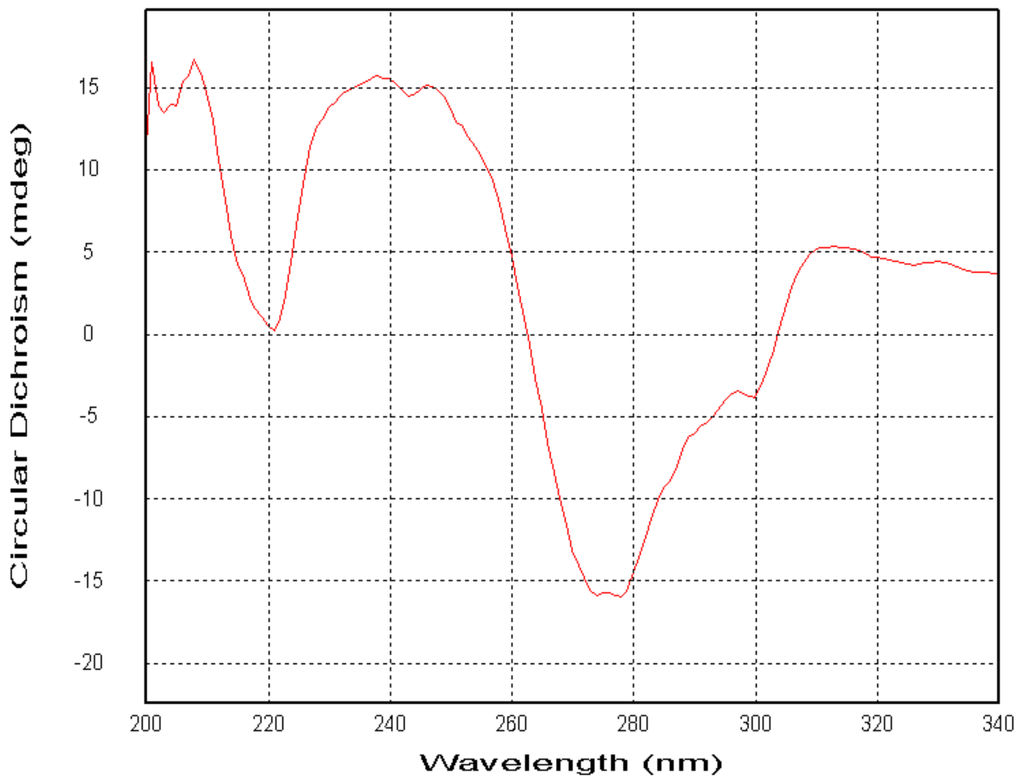

Supplementary Figure 113. CD spectra for product **2f**

2i

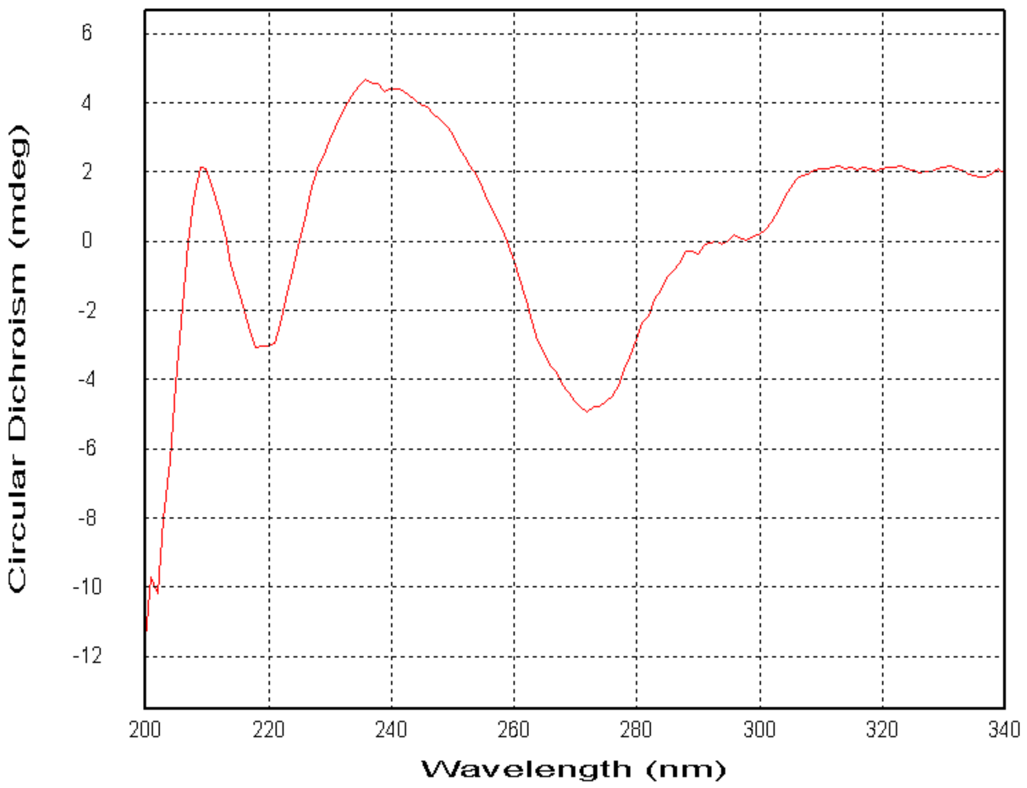

Supplementary Figure 114. CD spectra for product **2i**

2k

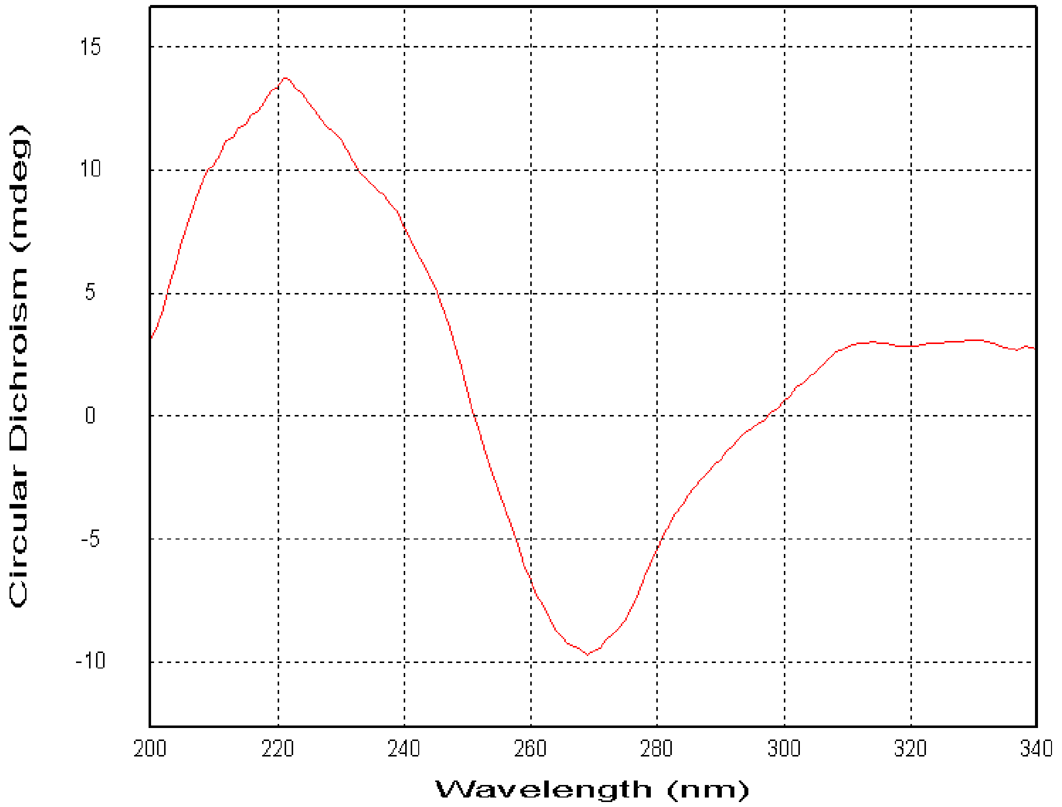

Supplementary Figure 115. CD spectra for product **2k**

2m

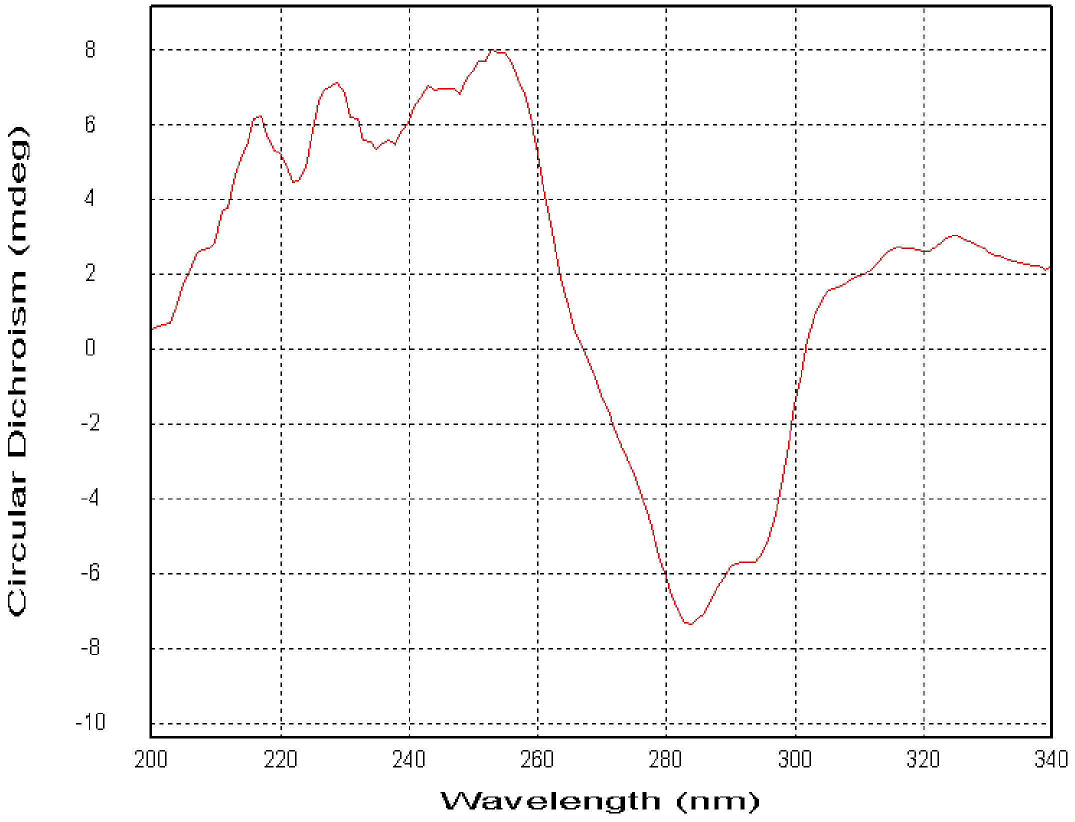

Supplementary Figure 116. CD spectra for product **2m**

4e

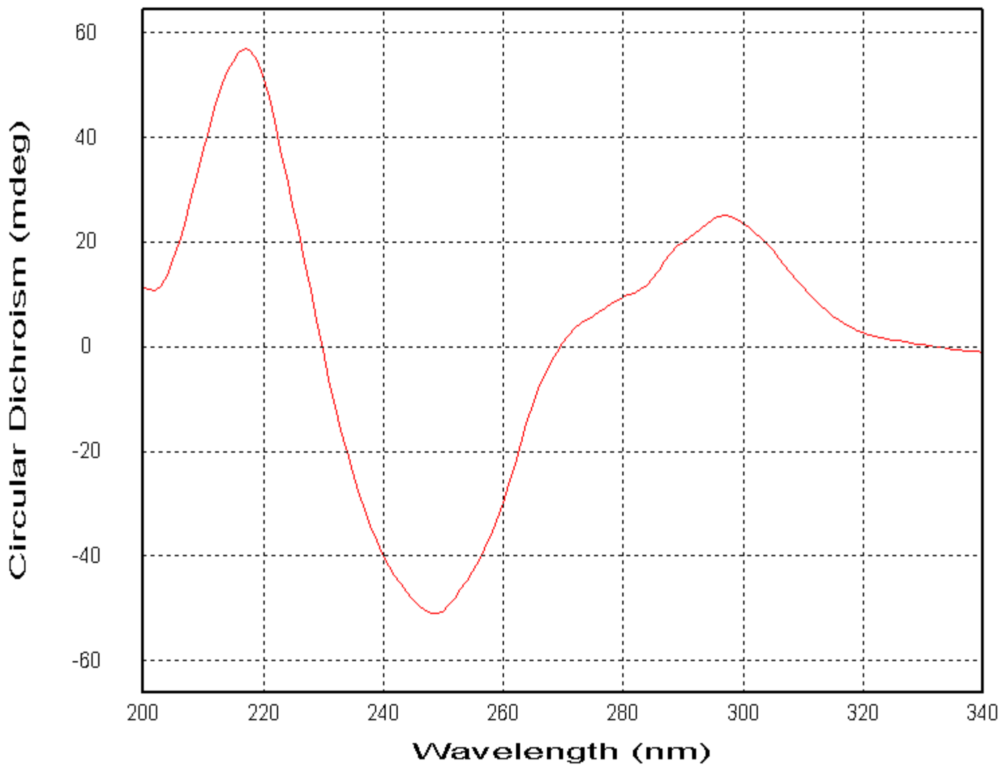

Supplementary Figure 117. CD spectra for product **4e**

4a

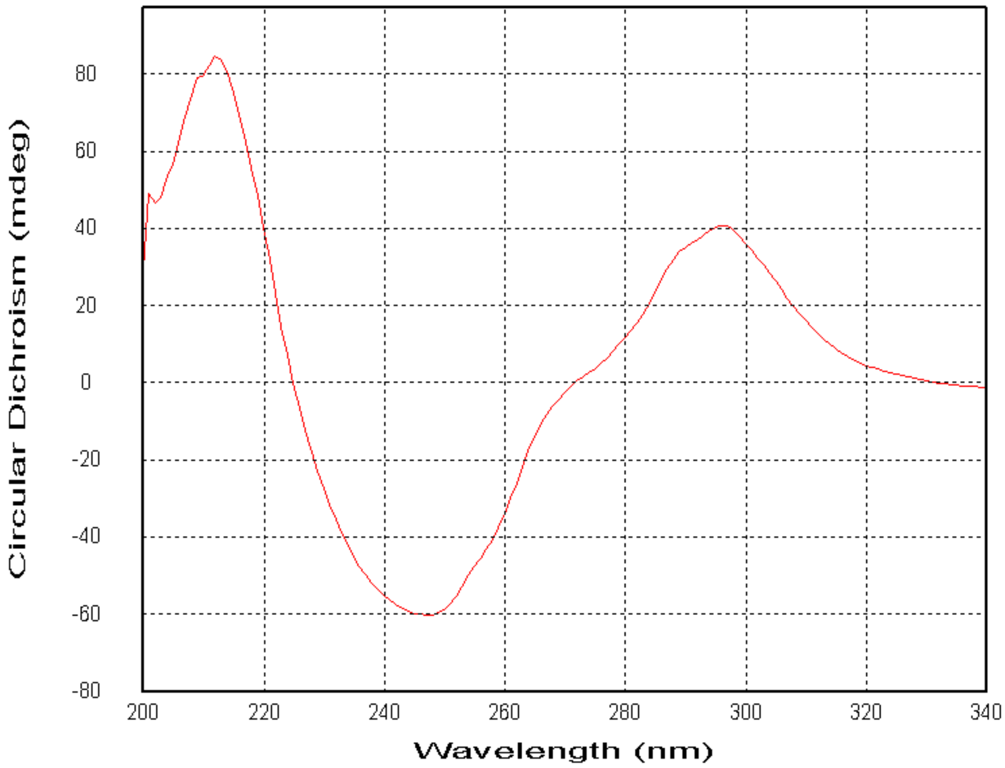

Supplementary Figure 118. CD spectra for product **4a**

4b

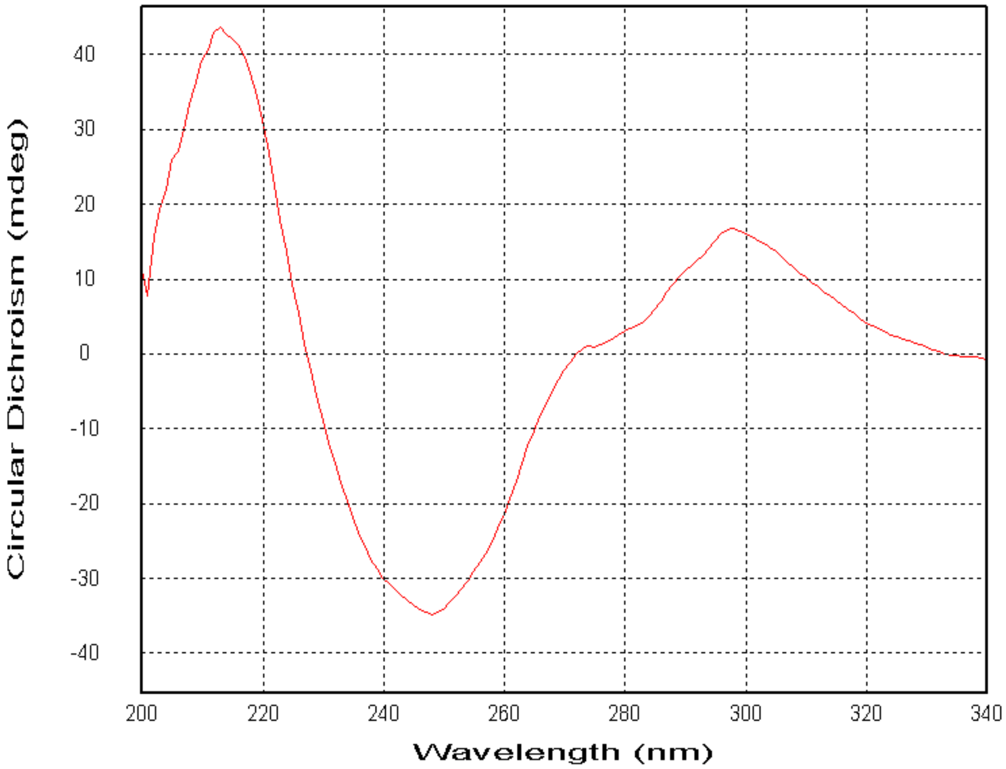

Supplementary Figure 119. CD spectra for product **4b**

4d

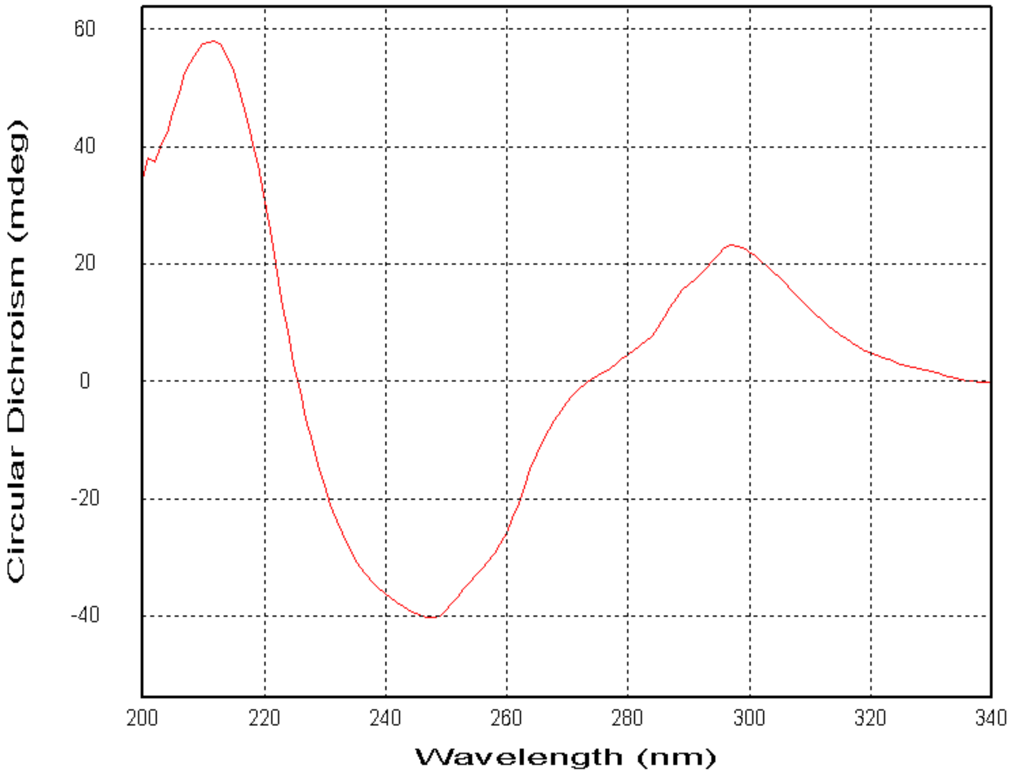

Supplementary Figure 120. CD spectra for product **4d**

4f

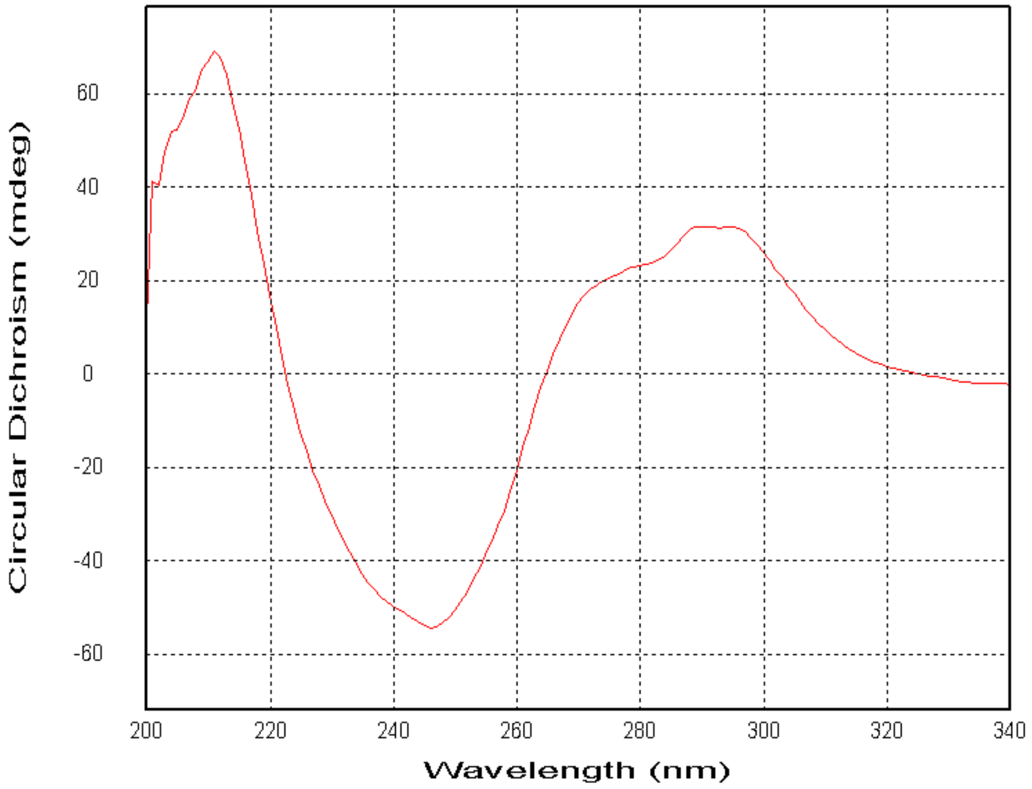

Supplementary Figure 121. CD spectra for product **4f**

4g

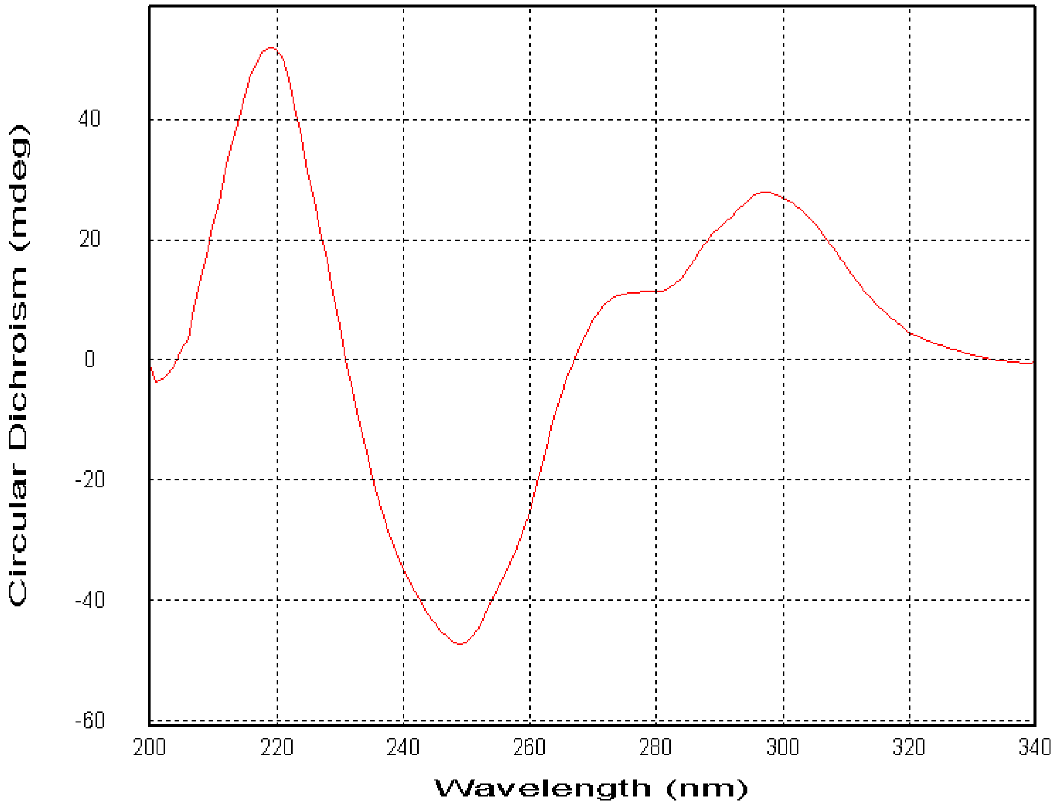

Supplementary Figure 122. CD spectra for product **4g**

4h

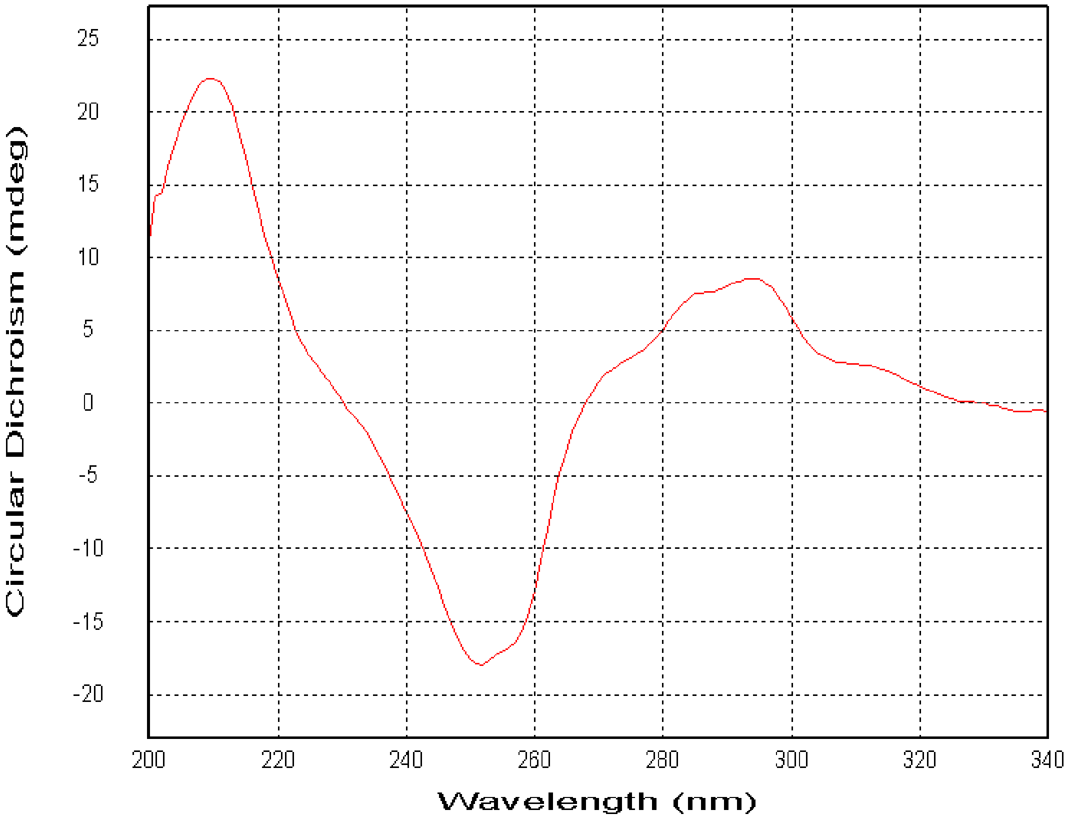

Supplementary Figure 123. CD spectra for product **4h**

Supplementary References

[1] Akula, P. S., Hong, B.-C. & Lee, G.-H. Catalyst- and Substituent-Controlled Switching of Chemoselectivity for the Enantioselective Synthesis of Fully Substituted Cyclobutane Derivatives via 2 + 2 Annulation of Vinylogous Ketone Enolates and Nitroalkene. *Org. Lett.* **20**, 7835–7839 (2018).

[2] Wang, S.-H., Tu, Y.-Q. & Wang, M. *t*-BuOK promoted coupling of alkynes and aldehydes: a concise synthetic method of β,γ-unsaturated enones. *Tetrahedron Lett.* **47**, 8621–8623, (2006).
